# Genome-wide analysis of MATE transporters and expression patterns of a subgroup of *MATE* genes in response to aluminum toxicity in soybean

**DOI:** 10.1186/s12864-016-2559-8

**Published:** 2016-03-11

**Authors:** Juge Liu, Yang Li, Wei Wang, Junyi Gai, Yan Li

**Affiliations:** National Key Laboratory of Crop Genetics and Germplasm Enhancement, National Center for Soybean Improvement, Key Laboratory for Biology and Genetic Improvement of Soybean (General, Ministry of Agriculture), Jiangsu Collaborative Innovation Center for Modern Crop Production, Nanjing Agricultural University, Nanjing, 210095 China

**Keywords:** Abiotic stress, Aluminum toxicity, *cis*-element, Duplication, Expression analysis, MATE, Phylogenetic analysis, Soybean

## Abstract

**Background:**

Multidrug and toxic compound extrusion (MATE) family is an important group of the multidrug efflux transporters that extrude organic compounds, transporting a broad range of substrates such as organic acids, plant hormones and secondary metabolites. However, genome-wide analysis of MATE family in plant species is limited and no such studies have been reported in soybean.

**Results:**

A total of 117 genes encoding MATE transporters were identified from the whole genome sequence of soybean (*Glycine max*), which were denominated as *GmMATE1* - *GmMATE117*. These 117 *GmMATE* genes were unevenly localized on soybean chromosomes 1 to 20, with both tandem and segmental duplication events detected, and most genes showed tissue-specific expression patterns. Soybean MATE family could be classified into four subfamilies comprising ten smaller subgroups, with diverse potential functions such as transport and accumulation of flavonoids or alkaloids, extrusion of plant-derived or xenobiotic compounds, regulation of disease resistance, and response to abiotic stresses. Eight soybean MATE transporters clustered together with the previously reported MATE proteins related to aluminum (Al) detoxification and iron translocation were further analyzed. Seven stress-responsive *cis*-elements such as ABRE, ARE, HSE, LTR, MBS, as well as a *cis*-element of ART1 (Al resistance transcription factor 1), GGNVS, were identified in the upstream region of these eight *GmMATE* genes. Differential gene expression analysis of these eight *GmMATE* genes in response to Al stress helps us identify *GmMATE75* as the candidate gene for Al tolerance in soybean, whose relative transcript abundance increased at 6, 12 and 24 h after Al treatment, with more fold changes in Al-tolerant than Al-sensitive cultivar, which is consistent with previously reported Al-tolerance related *MATE* genes.

**Conclusions:**

A total of 117 MATE transporters were identified in soybean and their potential functions were proposed by phylogenetic analysis with known plant MATE transporters. The *cis*-elements and expression patterns of eight soybean *MATE* genes related to Al detoxification/iron translocation were analyzed, and *GmMATE75* was identified as a candidate gene for Al tolerance in soybean. This study provides a first insight on soybean MATE family and their potential roles in soybean response to abiotic stresses especially Al toxicity.

**Electronic supplementary material:**

The online version of this article (doi:10.1186/s12864-016-2559-8) contains supplementary material, which is available to authorized users.

## Background

Multidrug and toxic compound extrusion (MATE) family is the most recent categorized multidrug efflux transporter family, which is a secondary transporter family that couples the translocation of substrates with an electrochemical gradient of cations (such as H^+^ or Na^+^ ions) across the membrane [1, 2]. The X-ray structure of the MATE transporter, NorM from *Vibrio cholerae*, reveals a unique topology of its predicted 12 transmembrane (TM) helices, which is distinct from any other known multidrug resistance transporter [3].

MATE transporters are widely distributed in bacteria, fungi, mammals and plants [4]. Hvorup et al. [5] found 203 sequenced proteins in the MATE family, which could be divided into 15 subfamilies. Omote et al. [4] identified 861 MATE transporters from Archaea, Eubacteria and Eukarya and classified them into three large subfamilies comprising 14 smaller subgroups. There are 56, over 40 and 53 putative MATE transporters in *Arabidopsis thaliana* [6], *Medicago truncatula* [7] and *Oryza sativa* [8], respectively.

The bacterial MATE transporters can export organic cations for multidrug resistance [9, 10]. In yeast (*Saccharomyces cerevisiae*), the Erc1 MATE-type transporter confers resistance to ethionine [11]. The mammalian MATE transporters can excrete the metabolic waste and xenobiotic organic cations in the kidney and liver [12, 13]. The plant MATE family transports a broad range of substrates such as organic acids, plant hormones and secondary metabolites [14–16]. Recently, the functions of many MATE transporters have been illustrated in plants [17], including transport of secondary metabolites such as alkaloids [18], flavonoids [7, 19], and anthocyanidin [20–23], detoxification of toxic compounds or heavy metals [6, 24], regulation of disease resistance [25–27], efflux of plant hormones such as abscisic acid (ABA) [28], iron translocation [29–31] and aluminum (Al) detoxification [32–35], which indicates MATE transporters play important roles in a wide range of biological processes in plants.

Al toxicity is considered as the main factor limiting crop yield on acidic soils [36]. Under Al stress, root exudation of organic acids, such as malate, citrate, and oxalate, is an important mechanism in plant resistance to Al toxicity [37, 38]. The genes controlling organic anion efflux from roots have been isolated from several crop species [39, 40]. MATE transporters have been shown to mediate the citrate efflux to confer plant tolerance to Al toxicity [41]. The MATE transporters involved in detoxification of Al were first identified from sorghum (*Sorghum bicolor*, SbMATE) and barley (*Hordeum vulgare*, HvAACT1) by map-based cloning, respectively [14, 32]. Later study found that the function of HvAACT1 protein is to release citrate to facilitate the translocation of iron from roots to shoots, and the 1-kb insertion in the upstream of the *HvAACT1* coding region in the Al-tolerant barley variety enhances and alters its expression to root tips, which is important to detoxifying Al and adaptation to acidic soils in barley [33]. Overexpression of *HvAACT1* increases citrate efflux and Al tolerance in wheat and barley [34]. BoMATE from cabbage (*Brassica oleracea*) requires Al^3+^ to activate citrate efflux, leading to enhanced Al tolerance in *A. thaliana* [35]. Several other MATE transporters, such as EcMATE1 (*Eucalyptus camaldulensis*), OsFRDL4 (*O. sativa*), and ZmMATE1 (*Zea mays*), are found localized to plasma membranes in the root tips and related to plant tolerance to Al toxicity [42–44].

Compared with other plant species, little work on MATE transporters has been done in soybean (*Glycine max* (L.) Merr.), which is an important oil crop worldwide. To date, only one MATE transporter, GmFRD3b (*G. max* ferric reductase defective 3b), was reported to play a role in iron efficiency in soybean [45]. With the public available whole genome sequence [46] and RNA-seq Atlas [47] of *G. max*, it is possible to identify the genome-wide *MATE* genes in soybean and investigate their expression patterns and possible functions. Plant MATE transporters have been shown to be involved in diverse functions including Al tolerance. By comparing the sequences of soybean MATE family with the known MATE transporters from other plant species, the possible roles of soybean MATE transporters could be proposed and help us to further test their function. In this study, we performed a genome-wide search of all putative MATE transporters in soybean. Their chromosomal distribution, gene duplication, phylogenetic relationship, structures of genes and proteins, and expression patterns were analyzed. Soybean *MATE* genes related to Al detoxification/iron translocation were further investigated by promoter analysis and differential gene expression analysis between the root tips of Al-tolerant and Al-sensitive soybean cultivars in response to Al. This study would provide useful information on the research of MATE transporters in soybean.

## Results and discussion

### Genome-wide identification of soybean MATE transporters

A total of 117 genes encoding MATE transporters (Additional file 1: Table S1) were identified from the soybean whole genome (see details in Methods), which were denominated as *GmMATE1* - *GmMATE117* according to the soybean nomenclature based on their physical locations [48]. The genomic sequences, coding sequences, and protein sequences of these 117 soybean MATE members (Additional file 2) were downloaded from Phytozome (http://phytozome.jgi.doe.gov/pz/portal.html) [49].

The details of all 117 soybean MATE proteins, including the length, molecular weight, number of TM, isoelectric point (pI), and predicted subcellular location, are listed in Additional file 3: Table S2. The Soybean MATE proteins consist of 80 to 593 amino acids, containing 2 to 13 TMs, whereas in Arabidopsis, the lengths of MATE proteins range from 400 to 700 amino acids and most with 12 TMs [6], indicating there are more variations within the soybean MATE family. The predicted molecular weights of soybean MATE proteins range from 8.71 to 64.28 kDa, and the predicted pI values are between 5.13 and 9.70. Their predicted subcellular locations include plasma membrane, chloroplast, cytoplasm, vacuole, endoplasmic reticulum, and extracellular, with 82.91 % (97 out of 117 MATE proteins) located in the plasma membrane, 7.69 % (9 out of 117) located in chloroplast, 5.12 % (6 out of 117), 2.56 % (3 out of 117), 0.85 % (1 out of 117) and 0.85 % located in cytoplasm, vacuole, endoplasmic reticulum, and extracellular, respectively.

### Chromosomal locations and duplication patterns of soybean *MATE* genes

**Fig. 1:**
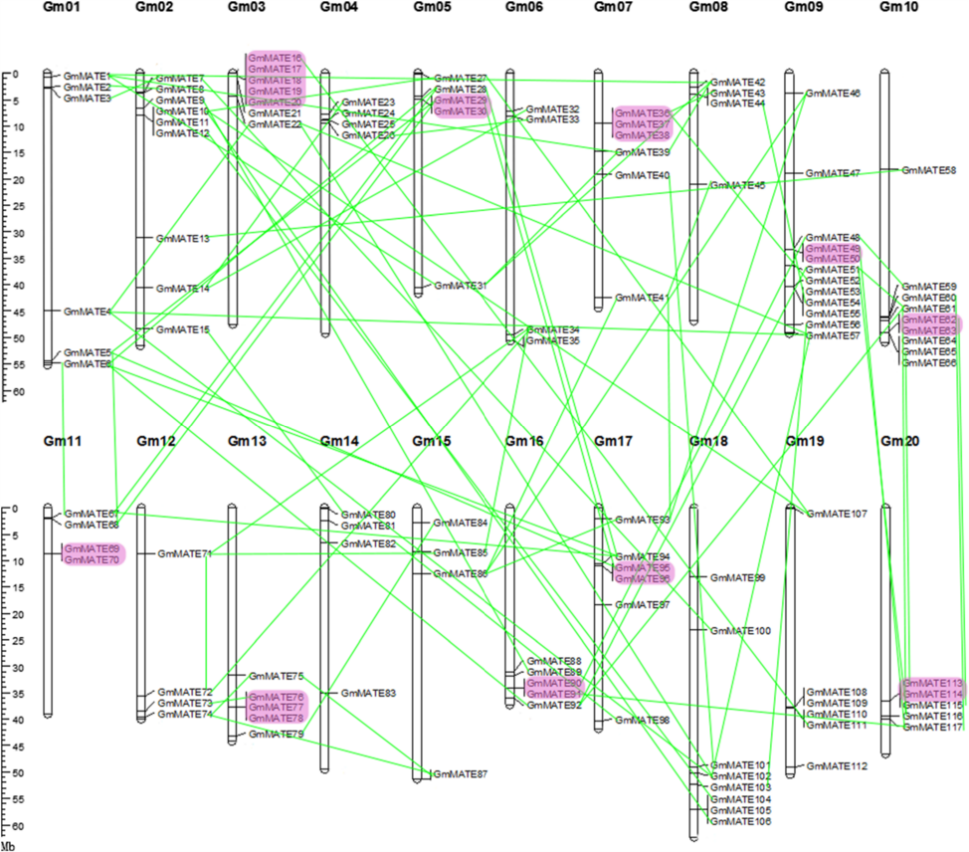
Chromosomal locations and duplications of soybean *MATE* genes. The chromosome number is indicated above each bar and the scale on the left is in megabases (Mb). The chromosome size is indicated by its relative length using the information from Phytozome and SoyBase. Tandemly duplicated genes are shown in purple boxes. Each pair of segmental duplication is indicated by a green line

Based on the physical positions (Additional file 1: Table S1), these 117 *GmMATE* genes are unevenly distributed on 20 soybean chromosomes (2n = 40, Fig. 1). The number of *GmMATE* genes on chromosomes 1 to 20 ranges from 4 to 12. Chromosome nine contains the highest number of *GmMATE* genes (12), whereas chromosomes 4, 6, 8, 11, 12, 14, and 15 contain fewest *GmMATE* genes (four on each). Majority of these *GmMATE* genes are located on the chromosome arms (Fig. 1), which are associated with high rates of recombination [50].

Compared with the number of *MATE* genes in *A. thaliana* [6], *M. truncatula* [7], and *O. sativa* [8], which contains 56, over 40, and 53 *MATE* genes, respectively, MATE family in soybean is remarkably large with 117 members, which might result from two whole-genome duplication events in soybean [46]. We further investigated the duplication patterns of soybean MATE family. The duplication analyses showed 96 out of 117 *GmMATE* genes (82.05 %) were present in duplications (Fig. 1; Additional file 4: Table S3), which indicates duplications contributed largely to the amplification of MATE family in the soybean genome, supporting the previous observation that duplications play important roles in the evolution of large gene families [51]. We observed 25 (21.37 %) *GmMATE* genes with tandem duplications (on chromosomes 3, 5, 7, 9, 10, 11, 13, 16, 17 and 20), and 71 (60.68 %) *GmMATE* genes with segmental duplications (Additional file 4: Table S3). Duplication events of *GmMATE* genes were found on all 20 soybean chromosomes.

### Phylogenetic analyses of the soybean MATE family

**Fig. 2:**
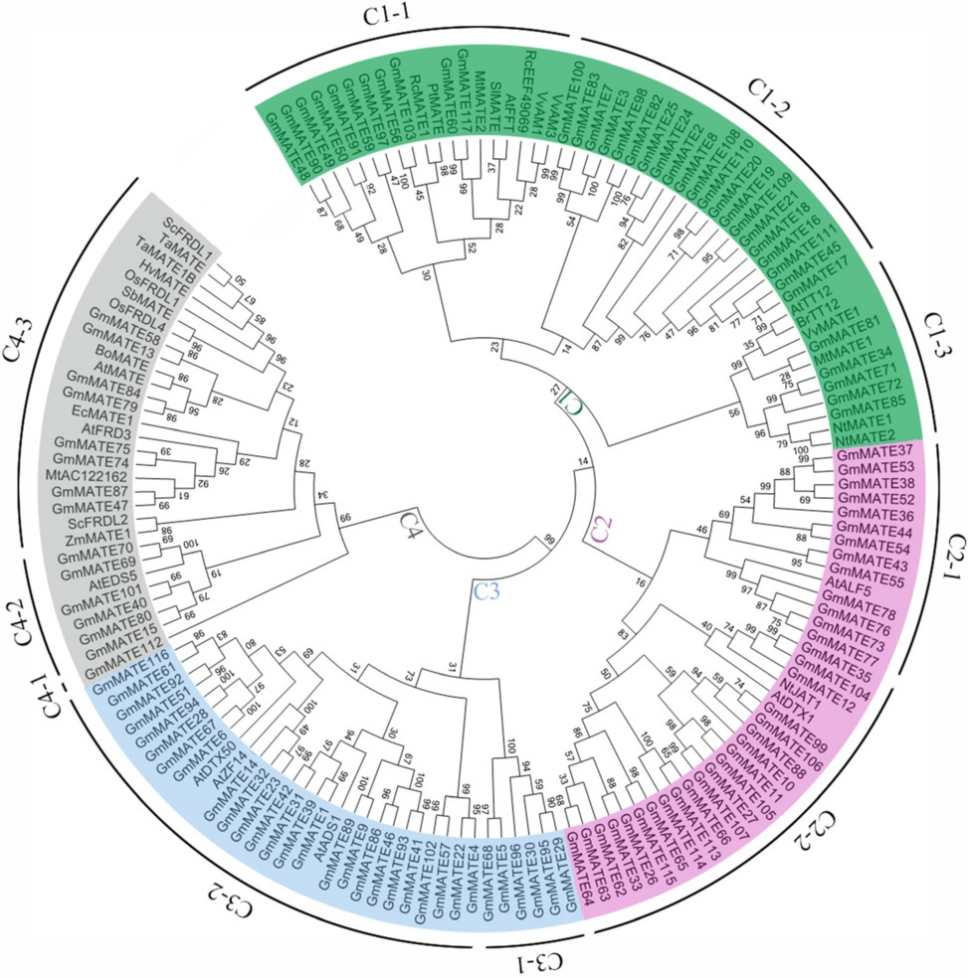
The phylogenetic tree of soybean MATE family. The phylogenetic tree was constructed by MEGA 6.0 using the Maximum Likelihood (ML) method. Bootstrap values in percentage (1000 replicates) are indicated on the nodes. Different subfamilies are highlighted using different colors (C1 in green, C2 in pink, C3 in blue and C4 in gray), and subgroups are marked with black arcs outside of the cycle tree

There are 35 plant MATE transporters that have been reported previously (Additional file 5: Table S4), including MATE transporters with known functions and few sequences that were reported by Zhao et al. [52]. Using the full-length protein sequences of the 152 MATE transporters, including the 35 previously reported plant MATE proteins and 117 soybean MATE proteins, we constructed a maximum likelihood (ML) tree (Fig. 2). These MATE proteins could be classified into four primary clades (subfamilies) comprising ten smaller subgroups (Fig. 2), including subfamily C1 (subgroups C1-1, C1-2, C1-3), subfamily C2 (subgroups C2-1 and C2-2), subfamily C3 (subgroups C3-1, C3-2,) and subfamily C4 (subgroups C4-1, C4-2, C4-3). The functions of soybean MATE proteins could be inferred from the known MATE transporters according to their phylogenetic relationships.

Subfamily C1 contains three subgroups, C1-1, C1-2 and C1-3. C1-1 consists of 19 sequences, including 11 MATE proteins from soybean and eight previously reported MATE transporters, such as AtFFT (*A. thaliana*, flower flavonoid transporter) [16], SlMATE (*Solanum lycopersicum* MATE) [23], VvAM1 and VvAM3 (*Vitis vinifera* anthoMATE1 and 3) [21, 22]. AtFFT is a flavonoid transporter that affects flavonoid levels in Arabidopsis [16]. SlMATE encodes a putative anthocyanin permease, which is co-regulated with ANT1 (anthocyanin) transcription factor, indicating it may function as an anthocyanin vacuolar transporter in tomato leaves [23]. VvAM1 and VvAM3 were found to be involved in the transport of acylated anthocyanins into vacuoles in grapevine [21, 22]. There are 21 soybean MATE proteins in the C1-2 subgroup, with no previously known MATE proteins. There are 11 proteins in the C1-3 subgroup, including five soybean MATE proteins and six previously reported MATE transporters such as AtTT12 (*A. thaliana* transparent testa 12) [19], MtMATE1 (*M. truncatula* MATE1) [7], NtMATE1 and NtMATE2 (*Nicotiana tabacum*, MATE1 and 2) [18], and VvMATE1 (*V. vinifera*, MATE1) [20]. Arabidopsis TT12, the first MATE transporter found to transport flavonoids [19], was originally isolated during screening of mutants with altered seed coloration. MtMATE1 from *M. truncatula* was a functional ortholog of AtTT12 and localized in the tonoplast [7]. NtMATE1 and NtMATE2 were suggested to transport alkaloids from the cytosol into the vacuole in tobacco [18]. The grapevine MATE transporter VvMATE1 was involved in the accumulation of proanthocyanidins [20]. The functions of the known MATE transporters in this clade suggest the MATE subfamily C1 might be involved in (vacuolar) transport and accumulation of flavonoids or alkaloids in plants.

There are 34 soybean MATE proteins in subfamily C2, which are divided into two subgroups. Subgroup C2-1 has 13 soybean MATE proteins and a known MATE protein AtALF5 (*A. thaliana* aberrant lateral root formation 5), in which mutation led to defects in lateral root formation and increased sensitivity of roots to various compounds, therefore it is thought to confer plant resistance to toxins [24]. Subgroup C2-2 contains 21 soybean MATE members, as well as AtDTX1 (*A. thaliana* detoxification 1) and NtJAT1 (*N. tabacum* jasmonate-inducible alkaloid transporter 1) [6, 53]. AtDTX1 was found to mediate the efflux of plant-derived antibiotics and other toxic compounds, and was also able to detoxify the heavy metal, Cd^2+^ [6]. Tobacco NtJAT1 showed nicotine efflux activity in yeast and was suggested to function as a secondary transporter for nicotine translocation [53]. Therefore, subfamily C2 might be related to the efflux of various compounds.

Subfamily C3 could be further classified into two subgroups. None of the known MATE proteins appears in subgroup C3-1 (six soybean MATE proteins). Subgroup C3-2 contains 29 soybean MATE proteins and three known MATE proteins, AtADS1 (*A. thaliana* activated disease susceptibility 1, which is also known as Arabidopsis abnormal shoot 3, ABS3) [25, 54], AtDTX50 (*A. thaliana* detoxification efflux carrier 50) [28], and AtZF14 (also known as bush and chlorotic dwarf 1, BCD1 or Arabidopsis abnormal shoot 4, ABS4) [15, 54, 55]. Previous research indicated that AtADS1/ABS3 is a negative regulator of plant disease resistance [25]. AtDTX50 functions as an ABA efflux transporter, which regulates ABA sensitivity, stomatal conductance and drought tolerance in Arabidopsis [28]. Overexpression of *AtZF14/BCD1/ABS4* increased leaf initiation rate and it is also involved in iron homeostasis [15, 55]. Recently, AtZF14/BCD1/ABS4 and AtADS1/ABS3 were also found to regulate hypocotyl cell elongation [54]. The functions of MATE subfamily C3 seem diversified and need further investigation.

There are three subgroups in subfamily C4. Subgroup C4-1 only contains one soybean MATE protein. Subgroup C4-2 has six soybean MATE proteins and AtEDS5, which is essential for salicylic acid (SA) dependent disease resistance [26, 27]. Subgroup C4-3 contains 22 members, including eight soybean MATE proteins (one of which, GmMATE47, is the known GmFRD3 that has been reported to play a role in iron efficiency [45]), and 14 previously reported MATE proteins from other plant species that are all related to Al detoxification and/or iron translocation (Additional file 5: Table S4), indicating these eight soybean MATE proteins in subgroup C4-3 might be involved in Al detoxification/iron translocation in soybean.

### Gene structures and protein motifs of soybean MATE family

**Fig. 3:**
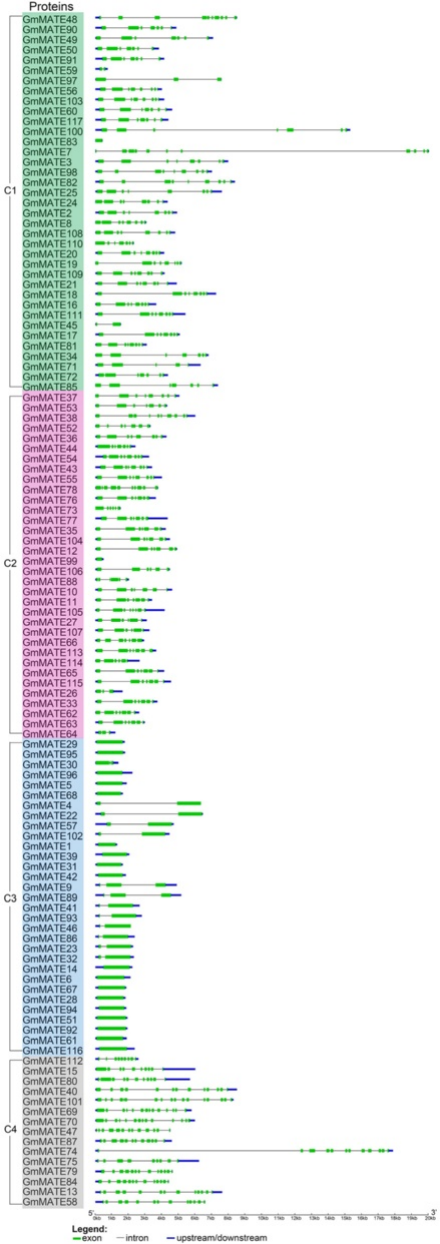
The gene structures of soybean MATE family. The structures of 117 *GmMATE* genes were plotted using green boxes representing exons (coding DNA sequence, CDS), black lines representing introns and blue boxes indicating upstream/downstream sequences. The scale on the bottom is in the unit of kilobase (kb). The genes are listed according to the order of subfamily C1 to C4 from the phylogenetic tree, and different subfamilies are highlighted in different colors (same as Fig. 2): C1 in green, C2 in pink, C3 in blue and C4 in gray

In order to better understand the characteristics of the soybean *MATE* genes, their structures were analyzed by comparing the genomic DNA sequences with their corresponding coding sequences (Additional file 2). Their intron-exon structures were plotted along with the order of subfamily in phylogenetic tree (Fig. 3). The *GmMATE* gene structures including length and number of exons and introns are more similar within the same subfamily (Fig. 3). Most genes in the largest subfamily C1 have 5–10 exons, except that *GmMATE45*, *GmMATE59* and *GmMATE83* have only 1–2 exons. Most genes in subfamily C2 contain 6–11 exons, except for *GmMATE26, GmMATE64* and *GmMATE99*, which only have 1–3 exons. The *GmMATE* genes in subfamily C3 contain only 1–3 exons. The *GmMATE* genes in subfamily C4 have 9–17 exons.

**Fig. 4:**
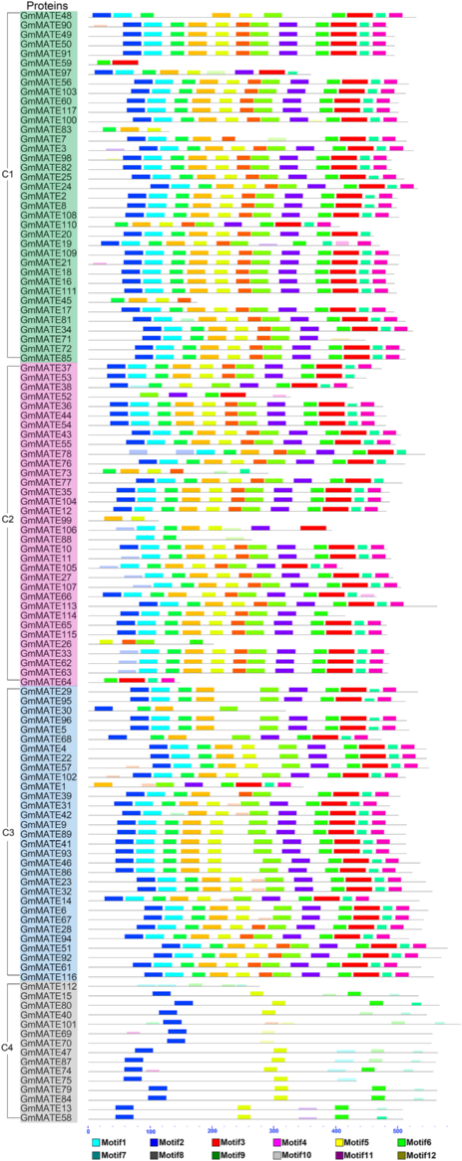
Protein motifs of soybean MATE family. The motifs of soybean MATE proteins are shown as colored boxes. The scale on the bottom may be used to estimate the length of motif (unit: amino acid). The GmMATE proteins are listed according to the order of subfamily C1 to C4 from the phylogenetic tree, and different subfamilies are highlighted in different colors (same as Fig. 2): C1 in green, C2 in pink, C3 in blue and C4 in gray

Next, motifs in soybean MATE protein sequences were identified using MEME (Fig. 4). The types and sequences of the motifs are similar among the first three subfamilies, C1, C2 and C3, but significant different with the fourth subfamily C4 (Fig. 4). The MATE proteins in subfamily C4 generally have fewer motifs than the first three subfamilies.

### Expression patterns of *GmMATE* genes in different soybean tissues

**Fig. 5:**
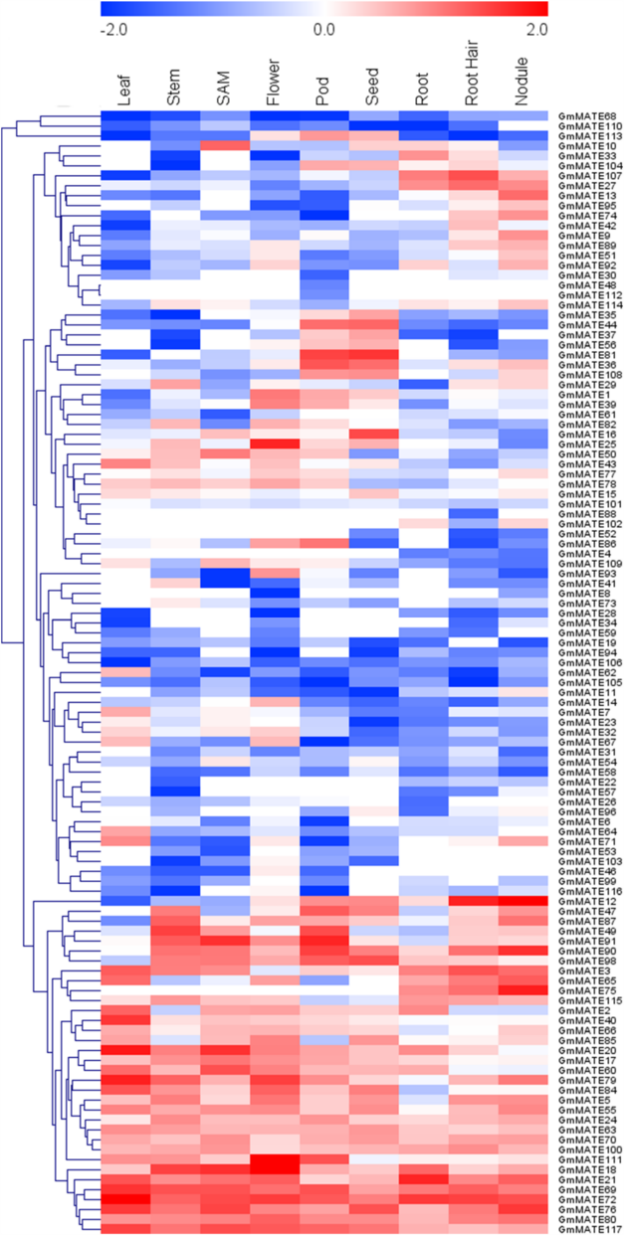
Heat map of the expression profiles of *GmMATE* genes in nine soybean tissues. The heat map with hierarchical clustering of 113 *GmMATE* genes was constructed using MeV 4.9 software by average linkage with Euclidean distance. Color key represents the relative transcript abundance of the *GmMATE* genes in nine soybean tissues. The FPKM (fragments/kilobase/million) values were log10 transformed and mean centred by genes using the MeV 4.9 software. SAM: shoot apical meristem

The relative transcript abundance of the 117 *GmMATE* genes in different soybean tissues was searched from Phytozome v10.3 [49]. There are four genes having the relative transcript abundance as 0 across all tested tissues and therefore were excluded for further analysis (Additional file 6: Table S5). A heat map with clustering of the rest 113 *GmMATE* genes (Fig. 5) was constructed using MeV software [56]. Most *GmMATE* genes showed specific tissue expression patterns. Some genes (e.g. *GmMATE107* and *GmMATE27*) showed higher expression levels in root/root hair/nodule while lower levels in above-ground tissues (Fig. 5). Some *GmMATE* genes such as *GmMATE44*, *GmMATE81* and *GmMATE36* were mainly expressed in pod and developing seed, suggesting their putative roles during seed development. Some *GmMATE* genes (e.g. *GmMATE1* and *GmMATE39*) showed higher expression levels in flower and pod. The expression levels of some genes (e.g. *GmMATE62* and *GmMATE7*) were higher in leaf but lower (or zero) in other tissues. There are also some genes (e.g. *GmMATE69, GmMATE72, GmMATE76, GmMATE80* and *GmMATE117*) expressed in all tested tissues (Fig. 5). The relative transcript abundance of the 117 *GmMATE* genes was also searched from the soybean RNA-Seq Atlas [47] in SoyBase [57]. There are 21 *GmMATE* genes have no data and 19 *GmMATE* genes have the relative transcript abundance as 0 across all tested tissues (Additional file 7: Table S6). The tissue expression patterns of most genes with non-zero relative transcript abundance from SoyBase were consistent with the Phytozome.

**Fig. 6:**
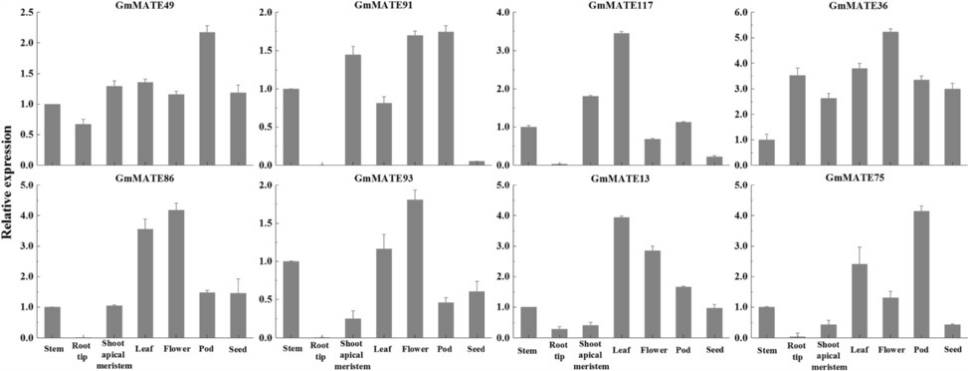
Relative expression levels of the representative *GmMATE* genes in seven soybean tissues. Eight *GmMATE* genes representing four subfamilies were randomly selected to validate their relative expression in different tissues by qRT-PCR. The relative expression level in stem was set as one and the soybean *GmEF-1*α gene was used as the internal control. The error bars indicate the standard deviation from three replicates

To cross-validate the expression patterns of *GmMATE* genes with the RNA-seq data, we performed quantitative real-time PCR (qRT-PCR) for eight representative genes randomly selected from the four subfamilies (Fig. 6). The relative expression of three genes (*GmMATE49*, *GmMATE91*, and *GmMATE117*) from subfamily C1 is higher in flower, leaf, pod, and shoot apical meristem (SAM) than root tip, which is consistent with Fig. 5. *GmMATE36* from subfamily C2 expresses in all tested tissues by qRT-PCR, which in general agrees with the RNA-seq data. *GmMATE86* and *GmMATE93* from subfamily C3 show high relative expression level in flower in both qRT-PCR and RNA-seq data. *GmMATE13* and *GmMATE75* from subfamily C4 express higher in leaf or pod by qRT-PCR but show higher expression in root hairs and nodules in RNA-seq data, which might be due to the difference in the sensitivities of two methods, RNA samples from two different cultivars (Williams 82 for RNA-seq and KF for qRT-PCR), or different growing environments.

### Characterization of putative *cis*-regulatory elements in the promoter regions of subgroup C4-3 *GmMATE* genes

**Fig. 7:**
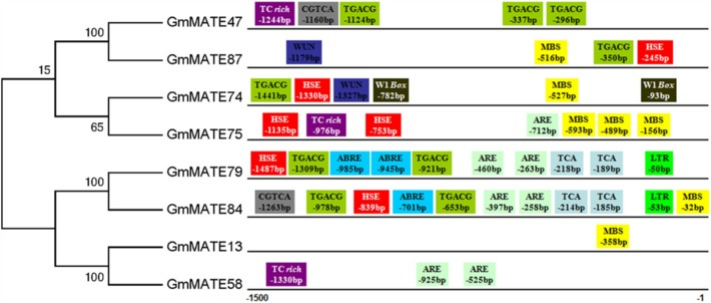
Stress-responsive cis-elements of the eight *GmMATE* genes in subgroup C4-3. The phylogenetic tree (maximum likelihood) on the left was constructed using MEGA 6.0 with the full sequences of the eight soybean MATE proteins in subgroup C4-3. The *cis-*elements in the 1500 bp upstream regions of the eight corresponding *GmMATE* genes were predicted using the PlantCare database, and shown in colored boxes with their names and positions (relative to the start codon) inside

Based on the phylogenetic tree, eight *GmMATE* genes were classified into subgroup C4-3 (Fig. 2), together with the previously reported genes that are all related to Al detoxification and/or iron translocation. The *cis*-acting regulatory elements in the promoter regions play important roles in plant response to stresses. Using the PlantCARE database, we identified 11 putative stress or hormone-responsive *cis*-acting elements in the 1500 bp upstream (Additional file 8) of these eight *GmMATE* genes (Fig. 7, Additional file 9: Table S7), including ABRE (ABA-responsive element), ARE (anaerobic-responsive element), CGTCA-motif, HSE (heat stress-responsive element), LTR (low temperature responsive element), MBS (MYB binding site), TCA-element, TC rich-repeats, TGACG-motif, WUN (wound-responsive element) and W1-Box. *GmMATE13* contains only one *cis*-acting element (MBS), while the other seven *GmMATE* genes have more than one predicted *cis*-acting elements. Two genes, *GmMATE79* and *GmMATE84*, contain ten and 11 *cis*-elements, respectively (Fig. 7). Seven *cis*-elements, ABRE, ARE, HSE, LTR, MBS, TCA-element and TC-rich repeats, are stress responsive. ABRE element is important in ABA signaling and plant response to drought and high salinity in Arabidopsis [58], which is present in two C4-3 *GmMATE* genes. ARE element was found both necessary and sufficient for induction of gene expression by low oxygen stress [59], which is present in four C4-3 *GmMATE* genes. HSE has been found to be consistently conserved in the regulatory regions of many heat induced genes [60], which is present in five C4-3 *GmMATE* genes. MBS *cis*-element was reported to bind to MYB transcriptional factors involved in stress signaling [61], and five C4-3 *GmMATE* genes contain MBS. LTR element is important for the induction of cold regulated genes [62], which is present in two C4-3 *GmMATE* genes. TCA-element mediates SA-signaling pathway and is sufficient for the response to stress [63], which is found in two C4-3 *GmMATE* genes. TC-rich repeats element is involved in defense and stress-responsiveness [64], which is present in three C4-3 *GmMATE* genes.

Another *cis*-acting element, GGN(T/g/a/C)V(C/A/g)S(C/G), has been identified as the DNA-binding sequence of ART1 (Al resistance transcription factor 1), which regulates the expression of 31 genes (including *MATE*) to confer Al tolerance in rice [65]. This element, GGNVS, was found in all eight C4-3 *GmMATE* genes, with different numbers and positions (Table 1). *GmMATE47*, *GmMATE75*, *GmMATE79* and *GmMATE87* contain more ten GGNVS elements, while only five GGNVS element was found in *GmMATE58*.

**Table 1:** Number and position of GGNVS element in the promoter region of eight C4-3 soybean *MATE* genes^a^

Genes	No. of GGNVS	Position of GGNVS^b^
*GmMATE13*	7	-639,-943,-944,-1149,-1150,-1398,-1451
*GmMATE47*	12	-232,-541,-548,-670,-769,-834,-1026,-1043,-1044,-1183,-1417,-1457
*GmMATE58*	5	-42,-122,-133,-1447,-1481
*GmMATE74*	7	-85,-287,-768,-1210,-1354,-1386,-1390
*GmMATE75*	12	-383,-609,-611,-612,-616,-884,-885,-1002,-1003,-1015,-1351,-1449
*GmMATE79*	15	-139,-143,-144,-260,-489,-513,-560,-590,-637,-724,-725,-990,-1139,-1283,-1446
*GmMATE84*	9	-271,-772,-893,-1150,-1177,-1287,-1443,-1444,-1445,
*GmMATE87*	11	-141,-376,-515,-516,-636,-671,-813,-1010,-1162,-1235,-1491

^a^GGNVS is the abbreviation for GGN(T/g/a/C)V(C/A/g)S(C/G), a *cis*-acting element (target DNA-binding sequence) of ART1 (Al Resistance Transcription Factor 1)

^b^The position is relative to the start codon of each gene

### Expression of the subgroup C4-3 *GmMATE* genes in response to Al toxicity

**Fig. 8:**
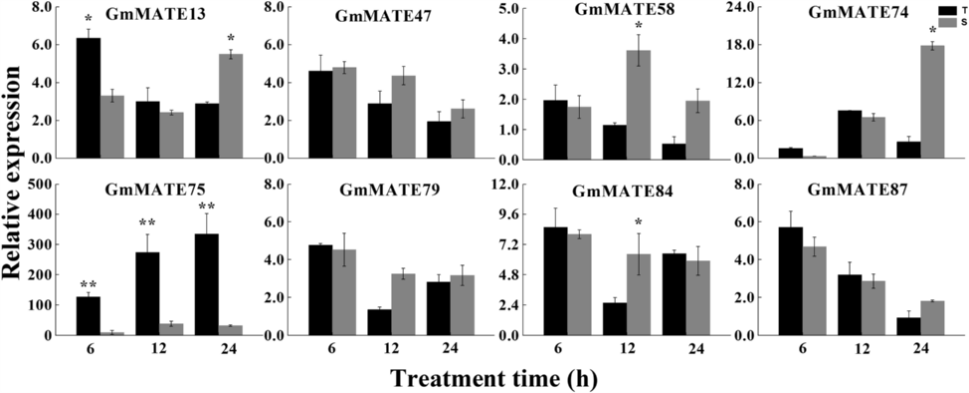
Relative expression of the eight soybean C4-3 *MATE* genes in response to Al toxicity. The relative expression of the eight *GmMATE* genes in the soybean root tips (0–2 mm) in response to Al stress (25 μM AlCl_3_) was quantified by qRT-PCR, using soybean *GmEF-1*α gene as the internal control and 0 μM AlCl_3_ (control) as reference. The error bars indicate the standard deviation from three replicates. T: aluminum-tolerant cultivar, KF; S: aluminum-sensitive cultivar, GF. * and ** indicates significant difference in the relative expression level between T and S in response to Al stress for each time point (student’s t-test) at *P* < 0.05 and *P* < 0.01, respectively

Ten out of 14 (71 %) MATE transporters from other plant species in subgroup C4-3 (Fig. 2, Additional file 5: Table S4) have been shown related to Al detoxification, therefore the expression patterns of all eight subgroup C4-3 *GmMATE* genes in response to Al toxicity were analyzed by qRT-PCR. Intraspecific variation in Al tolerance is striking in many crop species [14, 66, 67]. Previous studies showed that Al-tolerance related *MATE* gene expression in plant root tips is up-regulated by Al and is significantly higher in Al-tolerant genotypes [43, 44]. In this study, two soybean cultivars, KF (Al tolerant) and GF (Al sensitive), were treated with 0 and 25 μM AlCl_3_ for 6 h, 12 h and 24 h, respectively. The relative expression levels of the eight C4-3 *GmMATE* genes in the root tips of soybean seedlings after Al stress treatment are shown in Fig. 8. Among these eight genes, only one gene, *GmMATE75*, showed a significantly higher relative gene expression in the Al-tolerant cultivar KF (T) than in Al-sensitive cultivar GF (S) at 6, 12, and 24 h after Al stress treatment. *GmMATE13* showed a significant higher relative expression in KF (T) at 6 h but significant higher in GF (S) at 24 h after Al stress treatment. *GmMATE58*, *GmMATE74*, and *GmMATE84* showed a significantly higher level of relative expression in GF (S) than KF (T) after Al stress treatment. *GmMATE47*, *GmMATE79* and *GmMATE87* did not show significant difference in their relative expression between GF (S) and KF (T) during Al stress treatment. Therefore, *GmMATE75*, which showed higher elevated transcript levels after Al treatment in Al-tolerant (T) than Al-sensitive (S) cultivar, would be a candidate gene for soybean tolerance to Al toxicity. The expression patterns of *GmMATE75* could also be visualized by semi-quantitative RT-PCR (Additional file 10: Figure S1). Under normal growth conditions without Al treatment, their expression levels were very low and no difference was observed between KF (T) and GF (S). However, under Al treatment, their expression levels increased significantly, which was consistent with the qRT-PCR results (Fig. 8). A previous microarray study reported Gma.8768.1.A1_at, a putative *MATE* gene, was up-regulated (approximately 23-fold change) by Al treatment in an Al-tolerant soybean variety Jiyu 70 [68], which is the same gene as *GmMATE75* we identified in this study. In our study, the relative expression of *GmMATE75* under Al stress was further compared between Al-tolerant and Al-sensitive varieties. *GmMATE75* was highly up-regulated by 127, 274, and 335-fold in KF (T) while 10, 39, and 33-fold in GF (S) after 6, 12, and 24 h Al treatment, respectively, suggesting its role in soybean tolerance to Al stress.

## Conclusions

A comprehensive genome-wide analysis of MATE family is performed in an important legume species and oil crop, soybean. A total of 117 MATE transporters were identified in soybean and could be classified into four subfamilies, C1, C2, C3 and C4. The soybean MATE family displays great variation in gene structure, protein motif, and tissue expression pattern, which indicates their diverse functions. The expansion of soybean MATE family was largely due to segmental duplications. Seven stress-responsive *cis*-acting elements and the *cis*-acting element of ART1 (GGNVS) were identified in the upstream regions of eight *GmMATE* genes in subgroup C4-3, which contains previously reported *MATE* genes related to Al detoxification and/or iron translocation. One gene from C4-3 subgroup, *GmMATE75*, showed differential relative transcript abundance between the root tips of Al-tolerant and Al-sensitive soybean cultivars in response to Al treatment, indicating its potential role in soybean tolerance to Al toxicity. This study provides a foundation to further investigate the functions of soybean *MATE* genes including the candidate gene for Al tolerance in soybean.

## Methods

### Identification of MATE transporters in soybean

A total of 57 MATE (Pfam: PF01554) protein sequences in Arabidopsis were collected from Phytozome v10.3 [49] (http://phytozome.jgi.doe.gov/pz/portal.html). Soybean putative MATE protein sequences were retrieved by BLASTP searches against the target (*Glycine max* Wm82.a2.v1) proteome at Phytozome v10.3 using 57 *A. thaliana* MATE protein sequences as queries (E-value was ≤ 1e-7). These putative MATE sequences were filtered by the presence of conserved MATE domain (Pfam: PF01554) using the Pfam (http://pfam.xfam.org/) [69] and the Simple Modular Architecture Research Tool (SMART, http://smart.embl-heidelberg.de/smart/batch.pl) [70], and a total of 117 soybean MATE proteins with MATE domain were identified. Data files containing the information of the final 117 soybean MATE (including their corresponding physical locations on soybean chromosomes, genomic sequences, coding sequences, and protein sequences) were downloaded from Phytozome (http://phytozome.jgi.doe.gov/pz/portal.html) [49] (Additional file 1: Table S1 and Additional file 2). Theoretical isoelectric point (pI) and molecular weight (MW) of soybean MATE proteins were computed by ExPASy Compute pI/Mw tool (http://www.expasy.ch/tools/pi_tool.html) [71–73]. The subcellular localizations of the MATE proteins were predicted using WoLF PSORT (http://www.genscript.com/wolf-psort.html) [74] and the numbers of transmembrane helices were predicted by TMHMM Server v. 2.0 (http://www.cbs.dtu.dk/services/TMHMM/) [75].

### Phylogenetic and structural analyses of MATE transporters in soybean

The full protein sequences of 117 soybean MATE (Additional file 1: Table S1 and Additional file 2) and 35 previously reported MATE from other plant species (Additional file 5: Table S4) were used for multiple sequence alignments by ClustalW in MEGA 6.0 [76]. The unrooted phylogenetic tree was then constructed by MEGA 6.0 [76] using the Maximum Likelihood (ML) algorithm with 1000 bootstraps, where the amino acid substitution model was equal input model with uniform rates among sites, using partial deletion (95 % site coverage as cutoff) for gaps and missing data. Gene structure analysis was performed using the Gene Structure Display Server (GSDS) program with default settings [77]. Motifs in MATE proteins were statistically identified using the online tool of Multiple EM for Motif Elicitation (MEME) [78] (http://meme-suite.org/) with default settings: Motif Width: between 6 and 50 wide (inclusive). Site Distribution: zero or one occurrence (of a contributing motif site) per sequence. The maximum number of motif was set at 12 [4].

### Chromosomal locations and gene duplication analysis

The chromosomal locations of *GmMATE* genes were illustrated by MapChart [79]. Segmental and tandem duplication events of the soybean MATE family were identified using the Multiple Collinearity Scan toolkit (MCScan) [80] from the Plant Genome Duplication Database [81] with default settings: BLASTP was used to search for potential anchors (*E* <1e-5, top 5 matches) between every possible homolougous pair, and these pairs were used as the input for MCscan. Syntenic blocks were identified using the *E*-value ≤ 1e − 10 as a significance cutoff. Tandem duplication was defined as homologous genes with less than ten gene loci in-between and >50 % similarity at protein level on a single chromosome [82].

### Characterization of putative *cis*-elements in the promoter regions of subgroup C4-3 soybean *MATE* genes

The 1500 bp upstream sequences (Additional file 8) relative to the translation start codon of the eight C4-3 subgroup *GmMATE* genes were downloaded from Phytozome [49]. The *cis-*elements in the 1500 bp upstream regions were predicted using the PlantCare database (http://bioinformatics.psb.ugent.be/webtools/plantcare/html/) [83].

### Tissue expression patterns of *MATE* genes in soybean

The RNA-seq data of soybean *MATE* genes (Additional file 6: Table S5; Additional file 7: Table S6) were downloaded from Phytozome v10.3 [49] and RNA-seq Atlas [47] of *G. max* which is available on SoyBase (http://soybase.org/soyseq/) [57]. Since the RNA-seq Atlas on SoyBase was released in 2010 where the RNA-seq reads have been mapped only to the initial soybean genome assembly (Wm82.a1.v1.), the 21 *GmMATE* genes that were uniquely identified in the later assembly Wm82.a2.v1 are not represented in this dataset, and there are 19 *GmMATE* genes have the relative transcript abundance as 0 in all tested tissues. Therefore, we used the RNA-seq data from Phytozome to investigate the tissue expression patterns of soybean MATE family. The heat map with hierarchical clustering of 113 *GmMATE* genes from Phytozome (excluding 4 *GmMATE* genes with the relative transcript abundance as 0 across all tested tissues) was constructed to visualize their tissue expression patterns, using average linkage clustering with Euclidean distance by MeV 4.9 software [56].

Eight *GmMATE* genes from four subfamilies were randomly selected to be verified by quantitative real-time PCR (qRT-PCR). Soybean tissues were collected according to the developmental stages described by Marc Libault [84]. The seeds of soybean cultivar Kefeng-1 (KF) were germinated in moist sterile sand. Root tips of 3-day-old seedlings were harvested. The seedlings were transferred to the glasshouse under long-day conditions (16-h day/8-h night) at 26/24 °C temperature circulations. Shoot apical meristem (SAM) from V2 stage plants, first trifoliate leaves, stems, and flowers from R2 stage plants, pods from R4 stage plants, and seeds from R6 stage plants were harvested and immediately frozen in liquid nitrogen and stored at − 80 °C. The experiment was performed in triplicates.

### Al treatment and RNA isolation

The seeds of Al-tolerant soybean cultivar KF and Al-sensitive cultivar Guangfengmaliaodou (GF) (obtained from the National Center for Soybean Improvement, Nanjing, China), were germinated in moist sterile sand and grown under a photoperiod of 14-h day/10-h night and 26/24 °C (day/night) temperature circulations for three days. Then the seedlings were transferred to 0.5 mM CaCl_2_ (pH = 4.3) for 24 h before Al treatment. The seedlings were then exposed to 0.5 mM CaCl_2_ (pH = 4.3) solution containing either 0 μM AlCl_3_ (control) or 25 μM AlCl_3_ (treatment) for 6 h, 12 h and 24 h, respectively. The root tips (0–2 mm) were collected and immediately frozen in liquid nitrogen and stored at − 80 °C. The experiment was performed in triplicates.

Total RNA was extracted from all samples using TRIzol according to the manufacturer’s protocol (Invitrogen, USA).

### Real-time PCR

The first-strand cDNAs were synthesized using a PrimerScript First Strand cDNA synthesis kit (TaKaRa, Japan) following the manufacturer’s protocol, in a total of 20 μl reaction volume including 1 μg of total RNA, 4 μl 5X PrimeScript RT Master Mix, and RNAase-free ddH_2_O. The Semi-quantitative RT-PCR was performed in a final volume of 20 μl containing 2 μl of diluted cDNA, 10 μl 2X Premix Taq version 2.0 Mix (TaKaRa, Japan), and 200 nM of forward and reverse primers (Additional file 11: Table S8). The thermal cycling conditions were set as follows: different cycles of 95 °C for 30 s, 54.5–55 °C for 30 s (54.5, 55 °C for *GmMATE75* and *GmEF-1a*, respectively), and 72 °C for 45 s. The number of cycles is based on the genes and designed primers, which is labeled in the results. The housekeeping gene *GmEF-1*α was used as the internal control [85]. Electrophoresis was performed using 1 % agarose gels.

### Quantitative real-time PCR (qRT-PCR)

Gene-specific primers were designed using primer primer 5.0 (Premier Biosoft International, USA) and synthesized by Invitrogen (Shanghai, China). Quantitative real-time PCR was performed on a Roche 480 Realtime detection system (Roche Diagnostics, Switzerland) following the manufacturer’s instructions. The qRT-PCR was performed in a final volume of 15 μl containing 2 μl cDNA, 7.5 μl 2X SYBR Premix Ex Taq (TaKaRa, Japan), and 200 nM of forward and reverse primers. The amplification program was set as follows: initial denaturation at 95 °C for 5 min; 40 cycles of denaturation at 95 °C for 10 s, annealing at 58 °C for 20 s, and extension at 72 °C for 20 s. The amplification efficiencies (E) of primer pairs for 15 genes (including the housekeeping gene *GmEF-1*α) were estimated by qRT-PCR using 1X, 5X, 10X, 20X, and 30X dilutions of cDNA, according to the equation: E = [10^-1/slope^]-1 [86]. Primers and amplification efficiencies of qRT-PCR reactions were shown in Additional file 11: Table S8. The amplicon specificity was verified by melting curve analysis (Additional file 12: Figure S2) and agarose gel electrophoresis. Each experiment was performed in triplicates. The relative expression values were calculated by 2^−ΔΔCT^ method according to Livak and Schmittgen [87]. The housekeeping gene *GmEF-1*α was used as an internal control [85] and its invariant expression under our experimental conditions were shown in Additional file 13: Table S9 which showed relative constant Ct values across all samples. The relative expression level of *GmMATE* genes in response to Al stress (treatment, 25 μM AlCl_3_) was in comparison to their corresponding samples under normal conditions (control, 0 μM AlCl_3_) at each time point.

### Availability of supporting data

All supporting datasets of this article are included as additional files and available at doi: 10.6070/H47M05ZF that were deposited in LabArchives [88].

Phylogenetic datasets have been deposited in TreeBase and are accessible via the URL: http://purl.org/phylo/treebase/phylows/study/TB2:S18947?x-accesscode=5794eb5d85e615eed0fe24f0500a289b&format=html.

## Additional files

Additional file 1: Table S1. The nomenclature and physical locations of 117 soybean *MATE* genes. (XLS 39 kb) Additional file 2: The genomic sequences, coding sequences and protein sequences of the 117 soybean MATE members. (DOC 852 kb) Additional file 3: Table S2. Details of the 117 MATE proteins in soybean. (XLS 42 kb) Additional file 4: Table S3. Duplication analysis of the 117 Soybean *MATE* genes. (XLS 29 kb) Additional file 5: Table S4. The 35 known MATE transporters in plants. (XLS 51 kb) Additional file 6: Table S5. RNA-seq data of 117 soybean *MATE* genes in nine tissues as shown in fragments/kilobase/million (FPKM) from Phytozome. (XLS 43 kb) Additional file 7: Table S6. RNA-seq data of 117 soybean *MATE* genes in 14 tissues as shown in reads/kilobase/million (RPKM) normalization of the raw data from RNA-Seq Atlas on SoyBase. (XLS 44 kb) Additional file 8: The 1500 bp upstream sequences of the eight soybean C4-3 *MATE* genes. (DOC 43 kb) Additional file 9: Table S7. Details of *cis*-acting elements in the 1500 bp upstream of the eight soybean C4-3 *MATE* genes. (DOC 33 kb) Additional file 10: Figure S1. Semi-quantitative RT-PCR of the candidate *MATE* gene for Al tolerance in soybean. The semi-quantitative RT-PCR was performed using the RNA from soybean root tips (0–2 mm). - represents control plants (0 μM AlCl_3_) while + represents plants treated with 25 μM AlCl_3_. *GmEF-1*α was used as the internal control. T: aluminum-tolerant cultivar, KF; S: aluminum-sensitive cultivar, GF. The number of PCR cycles is shown on the right. (PNG 569 kb) Additional file 11: Table S8. Primers and amplification efficiencies for qRT-PCR and RT-PCR in this study. (XLS 22 kb) Additional file 12: Figure S2. The amplicon specificities of 15 pairs of primers for qRT-PCR in this study. (DOC 220 kb) Additional file 13: Table S9. The Ct values of the housekeeping gene *GmEF-1*α by qRT-PCR across all samples in this study. (XLS 32 kb)
