# Supplementary material for: Genome-wide analysis of MATE transporters and expression patterns of a subgroup of MATE genes in response to aluminum toxicity in soybean
Source: BMC Genomics. 2016 Mar 11;17:223. doi: 10.1186/s12864-016-2559-8 (PMC4788864; doi:10.1186/s12864-016-2559-8)
Supplement: Additional file 2: — The genomic sequences, coding sequences and protein sequences of the 117 soybean MATE members. (DOC 852 kb) [file 12864_2016_2559_MOESM2_ESM.doc]

**Additional file 2. The genomic sequences, coding sequences and protein sequences of the 117 soybean MATE members.**

**Note:** The genomic sequences are listed on pages 1-238, the coding sequences are listed on pages 238-318, and the protein sequences are listed on pages 318-345.

**The genomic sequences of 117 soybean MATEs**

>Glyma.09G134000 | Chr09:33326265..33334730 forward
ATGACGGTTTTACAATAAATCGTCGTTGAACACAACTATCTAAGACGATTTTTGTAAAACCATCGACGTTGAAGTTGTCTATATTACAAAATTGCACTGC
TCTAACACGGTTTTACAATGAAACCATGATGGTTTTACAATAAATCTCTATGTTAAGTTTGTTTTGTGGACAGTGACAGTGAAGATATGGAGAGTAGCAT
TCCCAATGGCATTGTTTGCACTGCTCTAGATTCTTTCAACCTCCTTAACTTCCATCTATGCTGGTCATATTGGAGACATTGAACTCTCTTCTATAGCCCT
TTACCAAGGTGTCATGAGTGCCATCTATTTTTACTTGCTGGTTGGTTAGCACACTTTCACATCTTCTATTCATCATAAAATTGATATGTAGTTCATTAGA
AATAAGCTCTATGAGTAGAGTACTTGCACACTATCATTGTATATTTTATCAACATCAATCGATTTCACTATGGGTTACTTTTATAGTACTCCTCTCGTTC
TTTTAAGATCTATGATCTCAGGCCACACCAATTGGGTTTTACTTGCATATCTCAATTTTTCTAATAGTTACCAAATCCATTAATGAGTAATTTAATTATG
CCAAAATGACAACTAACACTAGCAAGTAATTCCATCTCTTGTAAACTTGAGGCACATAAATGTTTTGGAAATATCTGAAGTTGTAATAATGATGAAGAAA
GTTCCCAACATTTTTTTTGAATGCCACTTTTTTTCTAGTGTATGATATGCTTGTTGTAGGTGGTTGTGGTGGTGGGAGTGGGGGGGGGGGGTGACAAATG
AGACTCATTCCCATTTAATACAATCTGTTTGGCAATAAGTGACAATTTAGAGAGAAAGAAATGATTGTATCTTTAGAAGAATAGGGTATTGGATGTAGAG
CATATTGTAGATGTTCAACAATTCCATTGTAGTGAAGGCCTGCAGGGGTAAAAAGAAGCATTCTATTATCGGGATCTTTTGCACAGCCATGGGGGGTTTC
AGTTTGTGGTGGTTTTCATGAAATATTGGGATGCAAGAGCCTTTTTATTTTGGAAAATTATGTGGTACCAGGGGTAACTTTCAGGCCAATACCATGTATG
TCTGGTTAACTTGACGGGTACATATGCTTTTGCATAAAGAACCTACTTAACCTGTTGTATTGTTTTTGTGAGCCCTTAGTTAGACACATCTTGTGTTTAA
CTTAGGCTGTAATAAATATTTTTGGCTTATAAAAAAGAAGAAAAACTAGCAAGTAAATGTGTGTTTTGATTTTATTTATTTTCTCATATGTGGAAAATTT
GGTGTTACCATGTACGAAAGAACTATTATTAGTGTATCATTGCTATTTCTCTATATGTGACTTTTTGATTTTTTTTATCTTATCTATGATTTGTGTGCAA
AATTGCAGTTTGGTATGTCATCTGCACTGGCAACACTTTGTGGCCAAGCTTTTGGAGCAGGACAAATTCAATCTACTTGTATTTATGTTCAAAGGTCATG
GATTATACTTACTGCCACTTGTACAATCCTCTTGCCTATCTTTGTATATGCCACACCAATCTTAAAGTTGCTTGGCCAAGATGAAGGAATTGCTGAACTT
GCTGGAAGATATTCTATACAAGTAATTGTTAGGATATGGGACGTGGTTATGCCTTTTTTCTATGGAAAGATATGGTAGAAATGTATGATAGAAATGATGG
GAGTAGGAAGAGGATGAGTTTTCAGAAAATTGACACCTTAACTTGAATTGTGCTTATTAGCTTCTTGACATACATTACATAGCTTGCTACAACTTATGCA
TTTATATAGGTTACATAGGTGATTTCTACACACTTCTATACTACTTAGTCCTAGAGTCTTCTAGACTCATCTATCATCTACAATTCATCTCTATAATGTT
CTAGGTGCTTCTATGACTTTAGATAATAAGATATTTTTCTACATCCATCAAGAAGTTTCTACACACTACAACATCTCCATAGGTGTAGAACTCTCTAGAT
AATGGACCACCAATTATACATCTACACTTTTATAGATTAATCTTCAACACTCCCCCTTAATCTAGAAAAGTCTTGAACTCCAAGCATGTCTCTGAATTTG
ATGAACTTCTATGTTGCAATTGGCTTGGTGAAGATGTCTGCTAATTGTTCTTCAGTCTTGCAGAACTCCATTTTGATTTCTCCATGTTCCACGAGCTCTC
TGATGAAGTGGTATCTAATCTCAATGTGCTTTGTACGGCTGTGGAATACTGGATTCTTAGTCATTGCAATAGCTGACATATTATCACAATGAATAACTGT
TGGAGTCTTGGAGTCTTGTTGTACGTCTTGCAGAATTTTTCTGAGCTACGTCGCTTCACACGCAGCGCTTGTTGCAGCCATGTATTCCGCTTTTGCTGAT
GATAGAGCAACTATCGATTGCTTATTTAACACCCATGATATTGCTTTGTTGCCTAGAAGAAACACATATCCACTTGTGCTTTTTCTATCATCTGTGCTTC
CTGCCCAATCGCTATTTGTATAACCTGTCAAGTTAAAGTCTCTATCTGCCTCGTACAAAATACCAAAATCCTTTGTGCCTTTGACATATCTTAGGATTCT
CTTGGCTGCTGCAAAGTGTGCTTTACTTGGGTTACTCATGAATCTAGACACGATGCTAACTGCATGTATGATGTATGGCCTTGAGTTGGTTAAGTAGATT
AGCGAACCGACTAGGCTTCTATAAACTGTTGCATCAACTTTGTTTTGCCCATCATCTTTTGAAAGCTTCTCGTTCATCGCCATAGGTGTGGCAAGTGGTT
TGCAATCTTTCATGTTGAACTTATTCAGTAAGTCATTCGCATATTTTTTTTGTGAGATAAATATTTGTCCAGGTCTTTGCTTGACTTGCATGCCGAGAAA
ATATCTCATGAGTCCAAGATCGATCATTTCATACTCTTGCATCATAGCCTTTTTGAATTCTTCCATCATCTGTGAGTTGTTGCCGGTGTAGATTAAATCA
TCTACGTATAGGCACAAAATCAAGAAATGACTACCTTGCATCTTCACATATAGTGACGACTCACTTGGACTTTTGAGGAATCTATGCTCGACGAGGTATT
TGTCTATTTTGTTGTTCCATGCTCGGGGAGCTTGTTTGAGACCATAAAGTGCCTTTTTCAAGCAATATACTTTGTCTTCTTCTCCTTGAACTTTGTATCC
TTGTGGTTGCTCCACGTAGACTTCTTCTTCAATCTCTCCATTCAAGAAAGCTGATTTTACATCTAGCTGGTAGACTTGTAGCTTCAACTGTGCAGCTAAG
GCTAGTACAGTTCTGATTGTTTCCATGCGCACAACGGGAGCAAATGTCTCATTGAAATCAACTCCTGGCAGTTGTGAATAGCCTTTAGCGACAATTCGTG
CTTTGTGTTTCTGGATTGTTCCATCCTCATTATATTTAATTTTGTAAACCCATTTCAGGCCAATGATCTCCTTGTCTTTTGGTTTTTGCATGAGCTCCCA
TGTATGATTTTTCTCTATCATTTTGATTTCCTCGTCCATGGATTTTCTCCAAACTTCTTCTTTCGATGCTTCCTCAAACTTTTGAGGTTCACAGGCAAAA
AAATCCAACATTTGCGTACTTTGGATTCGGTTTGTGTGGCCTTCCTGATCTTCGAAGTTGTTGTTGTGGGACTTCTTCTACTTCTTCTTGTTGGTGTTGT
TGGTCATGCTATGTTGGTGATGGTGGCAGTGTGCACGTGCTTGGATTGGTTACTTCTTGCATCTCATTGACTTCAGTACTCCATTGCCAGCTTGCTCCTT
CGTCAAAGATGACATCTCTTGAAATGATCAACTGCTTCGAATCTGGTTTGAATAATCGGTAACCTTTAGATTGGTCACTATAGTCGACAAAGATGCACTT
TTCTTCTTTTTCGTCGAGTTTTTCTCGATTCTCCTTTGGAATGTGCGAATAAGCAACACATCCAAAAACTTTAAAATGATCAACTATTGGTTTTCTGTTG
AACCATGCTTCATATGGCGTCATATTTAAGACTGCTATAGTTGGAGAACGGTTGAGGATGTACACTGCTGTGCTAACAGCTTTTGCCCATAGATTCTTTG
GTAGGTTCTTGTGTTGTAGCACCGATCGAGCCATTTCCACAATGGTTCTGTTTTTCCTTTCAGTGAGACCATTCTGTTGTGGAGTGTGACGAACAGTTAG
CTCCTTCTTGATGCCTTGATCATTACAAAAATTGATGAATATGTGCCCATTAAATTCTCCCCCGCGATCTGTTCTCAAGATTTTGAGGGAATGCCCGCTT
TGCTTTTCCACTAGGGCCTTAAACTCCTTGAAGCAAGAGAATGAATCGGATTTTTGTTGGATGAAGTACACCCACATCATTCGAGTGTAGTCATCAACGA
AGAGGAGAAAATATCTTCTTCCACCAAAGCTGGGGGTACTTGATGGTCCCCAGATATCAGCATGCACCAATTGAAGTGGAGCTTTAGCTCTCCAAGATGC
TTTTGGAAATGGTAACCTGTGCATCTTGCCATAAATGCAGCCTTCACAAACTTTAAGGTTAGATGAAATAAAAGGAAGCCCAAGCACCATATTTTTTTGT
TTTAATAATTTGAGGCCATTAAAGTTTATATGACCATACCTCAAGTGCCACAGTATGGACTCATCCATATTCTCACATTTCAATGCATTGTTTTGCCCAA
ATGGCATAAATAGAGGAAATACTTTATTAGGAGCCATTTTGATAGAAATAATTTGGCCCTTTCTTTTATCAATGATATTACATTTATCATCATCAAAGAT
GACTTTATAATTTTTATGAATAAGTTGACCAACACTTAGAAGATTTTGAGTAAGACCTGACATATAAAAAACATCATGGATATATTTTTGGCTACCATTT
TGAGTTTTAACAGCAATAATGCATTTTCCTTCAACTGTTTGAACATTTCCATCACCAAGTGTAACTTTGGATTTTATTCTCATCTAGTTGCACAAAGCAA
CTTTTGTTTTTTGTCATGTGGTTTGAACAACCGCTATCAACATACCATACATCCCCTGATTCTTTTTGTCTGTAAAAACAATCTTTTTCGTAGTGTAGGT
GCCTTTTGCACTTTTTGCATTTGTATCGACAATCATTTGTATCATGATTATTTTTCTTGCATAATTTGCAATAGGAGCTTGAAGCATTATTACTCCATTG
CTTTCCGCTCCTTTTACCACGATTAGTAAATGCTCCTCCTCTTTTTTTAGGAAATTTACTGTGGTTATTTTTCTTATCTCCATCATTTTTAGAGATATTA
ATTTTAGATTGGAATGTTTGCTCTAGTGGCTGCTCAAATCTTTTCATTCTAGCTTCATAAGCTTCTAAAGAGCCCATTAATTCTACTAGAGTGAGTTTAG
ATAAAAAAAATTATTCTTCTATGGTAGCCACAATATAATCATATTTTACGGGAAGACTTCTTAATATTTTTAGTATTATTTTCTTGTCTTCAATCGTGTC
CCCGTAACTTCTAATTTGATTGAAGATATTAAAGACACGAGAGAAGAAAGATTGTATAGATTCATCTTCCTGCATTATTAATGTATCAAAATCCTTCAAC
AAAGATTGAAGTTTAATAGAAATTATCTTGTCTGTGCCTTTAAATTCATTCTTCAGAGTCTCCCACGCCTCCTTTGCATTTTTTGCTTTCATGATTCTTA
GAAAGATCTCTCTACTAGCTCCTTGTTGGATGAATAGCAGAGCCTTAGCATCATGTTTCTTGTTTTGCTTGTACTCCTTTTGCTTTGTTACTGTCCACGT
AGCCAATTCTTCTTGGCTTTCTGGGACTGGATAGTCATTCTTTATAACATCCCACAAGTCTTGTGAGATGAATAGAGTTTGCATTTGGATGCTCCAATAA
TCATATGCTTCCCCATTAAATATGAGAACTAAGGTTTGGTTATAGTTGAAGGTTGGTGGGCTAGGTGATGTAGCAAAGGCCATAGGATCAACACTGCTCT
GATGCCATTGTTAGGATATGGGACGTGGTTATGCCTTTTTTCTATGGAAGGATATGGTAGAAATGTAAGATAGAAATGATGGGAGTAAGAAGAGGATGAG
TTTTCAAAAAATTGACACCTTAACTTGAATTGTGCTTATTAGCTTCTTCACATACATTACATGGCTTGCTACAACTTATGTCTTCATATAGGTTACATAG
GTGATTTCTACACACTTCTATACTACTTAGTCCTAGAGTCTTCTAGACATCTATCATCTACCATTTATCTCTATAATGTTCTAGGTGCTTCTATGACTTT
AGATAATAAGATATTTTTCTACATTCATCAAGAAGTTTCTACACACTACAACATCCCCATAGATGTAGAACTCTCTAGATAATGGACCACCAATTATACA
TCTACACTTTTCTAGATTAATCTTCAACAGTAATTCCCTACATGTTTTCTTTTGCTATAGCTTTTCCCGTCCAGAGATTTCTTTTGGCCCAGAGCAAGGT
TAAAGTAATTATGTGCATAGCATTTGTGGATTTGCTTATACAAAATGGGTTGCCTTACATATTCATAAATGTATTTGGTTGGGGTGTAACCAGCTTGGCA
ATAACGGCTAATATTGTAGGGTGGTTGTATGCTGTGGCTTTGGTTGTGTATACGATTGATTGGTGCAGAGAAAAATGGAGTAGATTTTGATGGATGGCAT
TTAGGGATTTGTGGGCCTTTGCTAAGTAAGCCTTCAATCATCTGTAATGAATTGCTTAGAGCAATGGTATATTACATGCATTATGCTACATGCTGGTCTG
TTAGACAATCCTGTGATTGGTGTTGGTTCCTATTCTATTTGGTAAGATGAATTTTTTTTTTTATAAGATGAACTAATTTGCAGCTGTTTAGATTGGTTAT
ATAAAACACACTTTTTCCATGACTTCTTTTTTCGACTGAATGAATTCAATAAATCTCTTTACATTTCTTGGTGCTCTTCATATATTTACTTTTGTATTTA
TAGTTTCAATATTCAGGGTTGGGACGACATGCTGCTCCTTGGAATAAATACAGTAATATGGTGAGTGACTATAAAATAAACACATGACAACATCAGTAAT
CAGTTTTAGGTTAAATGAAATCCAACTCAATAATCATATTTTTCATGTATTTGTTTCATTCTTACACTGAGTATTGGATCAATACCTTCCAAGTGTTCGT
GTCTCCAATACGCTTGGCAAGTCGCAGCCATATACTCTTTCTGCATGAAAATGTTCTTGTCTCTCCTCCTTGGTATTCTTTTCATGACTGTCATTTTCTT
TAGTAAAGATGAGTTTGCCAAAATCTTTACCGACAGTGAGGATATGATACTAGCTGATTCTGATTTAGCACACCTTCTTGGTGTAACAATTGTTCTCAAT
AGTGCTTCACAGGTCATGTCAGGTGATTGTTTTCTTTGGCAACTTGAAACATTCTTTTTGTAACATATTTTCTCCAGCTCATGAGTGTTCTGATATTGTA
CCACTAATTCTATTTTTCTATTTCTTTTAACATTTCCATAGGTGTGGCCATTGGAAGTAGATGGCAAGTTATGGTTGGTTACATAAACCTGGCATGTTAT
TACATTGTTGGACTCCCTATTGGAATTTTCCTTGGTTTCAAGCTGCATTTAGGGGTCAAGGTAACAATGTTATTGCTATTGGAAATTGGAAATGTTTCTT
TACTTCAATGTCAAATGCTGCCTTGTGTTTGTATCTTTGATCTCCAACTTATCAATTATGACTTTTCTTTCCTTCAAATTTATACTTAAAATGGTTTTGT
GGTTTAGGGTCTCTGGGGAGGCACAATGTGCAGCAGTATTCTTCAGATTTTAGTCCTCTTTACGATTATTTTGAAGACCAAATGGTCCAAGGAGGTAACT
CTATAGGATTTTTTCAATAAGCAGCTGTTTTTCGGCACTAATTCCAATTTTTTGCAGTGATTTTCTGTATCTTAATAATCATACAGAAATGTTCGATTTG
TATATATGCTAATTATTCTACCAAGTTATGCTATTAATGAGTTTTTGTCTTTAACATGCATCATGACTTCGAAAAATAAAAAAGGAAGCAACATGTGATT
TTGGCTAGAACTTGTATATAAGCAAGAAAGGACAGACACGACAAATCTATCAGTTGCTCTTGTGAAGCCATGTCAATGTTGTTTGTTTGTTTTCTATATA
TTTCAAAATATTTTTTGGTTTAAATCTGGCTAATTTGCAATTTAATACTCATAATTATAACTCACTTGAGTGTTTCAATATGCAACAGTGTTTGCCATTT
GCATTTGCCGGCTTTATTTTTTCAATGTCTGGTATCTTACGGTTACGGGGAATGTATGATGCAGATGGAACAAACAGCTCATCGCATGCGATTATGGAGC
AATAACATAACAACCTCCCCTTCAAAGAAAATGTTATATGAGAGAGAACAATGTGGTGTTATATGA

>Glyma.16G180000 | Chr16:34064572..34069421 forward
CCATTTAGTCCATTCTTCTTCCTAAAACGTAAGTGACTGTGTTGCATTCCATCTACTAACTGTAGCAGCTAAAATCTTCTGTCACAGTCGCACATCAAAG
GCAAAGCCTTCGTGCTTACTCATTTGCGTCAAAGCTTTACATGATCTGTTTAAGATTTTGGCTTTTATGATTTGAAAGTTATTATTTTTATTTGATTGAC
AAAAGTTAATTGTTAATTTGTTAGTTTTTGTTAGCAATCTCTCTCACCCTTCTGCATTCAACAACCAAGCCATCCTTATGACTCCTTGTTGTATGATTGG
AAGTTTATAACAAACTTCAGTTAGAAAGAAACTGAGTGTTGAGGGAAGAAAAAGAACTCCTCAAATGGAAACCCCATTAGTGATCCAAAAGCATACTTCA
GAACCCGACTATTTGCCGGTGGAAAGCTTGAAGGATGTTATGTTTGTTTTGCGAACGGAGACAGTGAAGATATGGAGAGTAGCACTCCCAATGGCTCTGC
TTGCACTGTTTCAGCTTCTCATGGACTCCTCAACTTCCATCTATGCTGGTCATATTGGAGACATTGAACTCTCTTCTATCGGTGTTTACCAAGGTGTCAT
CGGTGCCATCTATTTTTACTTGCTGGTTAGCACATTTTTACCTCTTCTATTCTTCATAAACTTAATATTTTTCTTATCATTTGATTTCTAAACACCGAAA
AATGAAGTTGTTGTTAAGAGTAGTAGTATTAACTCGTTAGAAATAAGCTCTATAAATAGAGTACTTGCTCACTGTCATTGTATCTTTTATCAAAATCAGT
CAATTACACTATGAGTTACTTATATGGTATGGTACTCTTCTCTTGTTCTTTGAGATCTACGGTCTCTGGCCTCACCAATTGTGTGTTACTTGCATATCTC
CTCAGTTTTCTTATAGGTAACCAAATCCATTAATGAGTAATTTATCTGTCTTTGTGCCCATATGACAACTAACACTGGCAAGTAATTCCATCTCTTGTAA
ACTTTTGTAAGTATCTGAAGTTGTAATAATGATGAAGAAAGTTCCCAACATTCTTCTTTGAATGCCACTTTTTTTTGCTAGTGTACTGTATGGTATGCTT
TTTGAGGAAAACTATGATGGACATGATGAAAGTGGTGATTGGAAGAAGGATTTATGAGCAGCAGTGGAGGATTTATTGTCTTTCACAAAAAAAGAACAGT
ATTAAAGATTCGGGAGAACAGGTTTTTATAGATTCTAACTCAAAAGAAGTAAATGTAAGGGAAGATGAACATCTTGAGCCTATGAGATGAGAAATTTGGA
CTGGACGGAAATTGAGAGTTTAAGTTGCAGACAAAATGGGTCAAGGTTGATTCCGACATTTGCACAAGCAGTAGATAGGCAATTCGTGAATTGAAGAGGA
TAAGGATTAATTGGTAAGGGCCTTGAGCCAGTTCACATACCAGTCACCCAGAACAGTTTAGAAGCATGCACTTGCTAATTGTTATTAGTTGTCATTAGTT
TTTTGGGGAGGAAATGGCAGAGTTCAACATTCCATTGTAGTGCAGGCCTGCAGGGGTAAAAGGAAGCATAGTACTTTGTGGTGGTTTTCATGATATATTG
AGATGGAAGAGCCATTTTTGGAAAATTATGTGGTACCAGGGGTAGCTTTCAGGCCAATACTGTGTATGTCTGGTTAACTTGACGAGTAGATATGCTTTGC
ATAAAGCAGCTACTTAACCTGCTGTATTCTTTTTGTGAGGCCTTAGTTAGACACATCTTGTGTTTAAGTTAGGCTTTAATAAATTTTTTTGGCTTATAAA
AAGAAAGAACAAAAACTAGCAAGTAAATGTGTGTTTTGATTTTATTTATTTTCTCATATGTGGAAAATTTGGTGTTTTAGTCCTTGTATAATTGAACAAT
TATTATTGTATCATTGCTATTTTGTCATATGTGACTTTTTGATTTTTTTTTATTATCTTATCTATGATTCATGTGCAAAATTACAGTTTGGTATGTCATC
TGCACTGGCAACACTTTGTGGCCAAGCTTTTGGAGCAGGAAAAATTCAATCTACTTGTATTTATGTTCAAAGATCATGGATTATACTTACTGCCACTTGT
ATAATCCTCTTGCCTATTTATGTATATGCCACTCCAATCTTAAAGTTGCTTGGCCAAGATGAAGGAATAGCTGAAGTTGCTGGAAGATATTCTATACAAG
TAATTCCCTACATGTTTTCTTTTGCTGTAGCTTTTCCCATCCAGAGATTTCTTCAGGCCCAGAGCAAGGTTAAAGTAATTATGTGCATAGCATTTGTGGA
TTTGCTTATACAAAATGGGTTGCTTTACATATTCATAAATGTATTTGGTTGGGGTATAACCGGCTTGGCAATAGTGACTAATATCGTAGGGTGGTTATAT
GCTGTGGCTTTGGTTGTGTATACCATTGGTTGGTGCAAAGAAGAATGGAGTGGATTTTGTTGGATGGCATTTAGGGATTTGTGGGCCTTTGCTAAGTTAA
GCCTTGCATCATCTGTAATGAATTGCTTAGAGCAATGGTATATTACATGCATTATGCTACTTGCTGGTCTACTAGACAATCCTGTGATTGCTGTTGGTTC
CTATTCTATTTGGTAAGATGAACTAATCTGCAGCTGTTTAGATTGGTTATAATACAAACTTTTTCCATGATTTTTATTTTCTACTGATTGATTCAGTAAA
ACTCTTTACATTTCTTGGTATTCCTCATAGATTTACTTTTGTATTTTAAGCTTCAATGTTCAGGGTTGGGACGACATGCTACGCCTTGGAATAAATACAG
CCATAAGGTGAGTGACTATAAAATAAACACATGATAACATCAGTTTTGGGTAAATGACATCCTACTTAATTTATCATGGGACTACAATTCTGGTGAAATA
TTATGTCAATATATGGACTGTTATCAATTACAAAGGAAACTAAATGTAAGAGGTTGATTATATATTTTTGGTTAATATGCTTAGTAGTTCCAACAAATAT
GGTGAATTTTAATTTTTTTCATTGCACTCCTTTGTTAGTTTTGATGCCAATAAGTTTTAAAAATAGTTCATTGGGCCATATGACGTGCATCTAGACATGC
AACCTCCACATGCCATTTAAGATTAATCACTGACATGCCATGTCAAGATACATGTTACGCCATCAAAAAAGATAATAGTGATCAATTTAAATTTATAAGA
ATCAAAACTAATAACAGTTTTCATAAACTAAAACCAAAATTTCCATATTTATCGGAACTAAGACATAGACATCTATTTTTCATGTATTTGTTTCATTCTT
ACACTGACTATTGGTTCAATACCCTCCAAGTGTTCGTGTCTCCAATACGCTTGGCATGTCGCATCCAAGAGCAGCCATATACTCTTTCTGTGTGACAATG
TTCCAGTCTCTCCTCCTTGGTATTCTTTTCATGACCGTAATTTTCTTTAGTAAAGATGAGTTTGCCAAAATCTTTACCGACAGTGAGGATATGATACTAG
CTGCTGCTGATTTAGCATACCTTCTTGGTGTAACAATTGTTCTCAATAGTGCTTCACAGGTGATGTCAGGTAATTGTTTTCTTTGGCAACTTGAAACATT
CTTTTTGTAACATTTTCTCCAGCTCATGAGTGTTCTGATATTGTACCCCTAATTCTAGTTTTTTTTTTTTTTTTTTAACATCTCCATAGGTGTGGCCATT
GGAAGTGGATGGCAAGTTATGGTTGGTTATATAAACCTAGCATGTTATTACATTGTTGGACTCCCTATTGGAATTTTCCTTGGTTTCAAGCTGCATTTAG
GGGTCAAGGTAACAATGTTACTGTTATTGGAAATGTATCTTTACTTCAATGTCAAATGCTGCCTATTTTTGTGTTTGTATCTTTGATCGATCTCCAACTT
ATCAATTATGAGTTTTCTTTCCTTCAAATTTATACTTAAATGGTTTTATGGTTTAGGGTCTCTGGGGAGGCACAATGTGCGGCAGTATTCTTCAGACTTT
AGTCCTCTTTACAATTATTTGGAAGACCAACTGGTCCAAGGAGGTAAGCTATAGGATTTTTTCAATAAGCAGCTGTTTTTTTGGCACTAATTCCAATTTT
TTGCAGTGATTTTCTGTATGTTAATTATCCAACAGCAATGTTCGATTTGTATATATACTAATTATCCTACCAAGTTAAGCTATTAATAAGTTTTTTTTTT
TACATGCATCATGGCTTAAAAAACAAAAAACAAAAAACAAAAAACAAAAAGGAAGCAACATGCGATTTTGGCTAGAACTTTTATATAAGCAAGAAAGGGA
AGACAGGACAAATCTATCGGTTGCTCTTGTGAAGCCATTGAAATGTTGTTTGTTTGTTTGTTTTCTATATATTTTAAAATATTTTTTTGGTTTAAACCTG
TCTAATTTGCAATTTAATACTCATAATTATAACTCACTCGAGTGTTGCAATATGCAACAGTGTTTGCCACTAGCATTAGCCAGCTTTATTTTTTCAATGT
CTAGTATCTTACGGGGAATGTATGACTGCAGGTGGAACAAACAGCTCATCGCATGCGATTATATGTGGAGCAATAACGGGACTAGTCCTGTGTGAAATAC
AAATTTGGGTTCTTTTTTATATTTAAGTAATATATACTGTCTCAAGCTCATAAGTGTTCTTCGCATACCATTAATTTTCTCTCTCTATATATGAATGTCA
GACACAAGAGAACGTTATTTTACTCAATAAGCCAAATGTAGAAGTAAAACTAAGTAGAAGTAAAAACGCAGACATTGTACGTTATTGGATGTGATCCATG
ATAACGACGGTCGAATTGTTTCAAAATAAATAAAACTAAAATTCTAGAGT

>Glyma.09G134100 | Chr09:33354120..33361171 forward
GTTTATAACAAAATTCAGCCTCAGAAACTGAGTATCAAGGGAAGAACTCCTCAAATGGAGACACCATTAGTGGTCCAAAATTTTACTTCAGAAGCTGACT
ATTTTCCAGTGAAAAGCTTGAAGGATGTTAAATTTGTTTTGTGGGCAGAGACAGTGAAGATATGGAGAATAGCATTGCCAGTGGCATTGACCCATCTATT
CCAGGTTCTCACAAACTCCTCAACTTCCATATATGCTGGTCATCTTGGAGACATTGAACTCTCTTCTATCTCTGTGAGCCAAGGGGTCATGAGTTCCATC
TATTTTCAGTTGTTGGTTAGTACATTCTAATCTCTCCAATATTAATGTTTTTTTTTTTCATTTGAGATCTAAACACAGAAAAATGAAGTTACCGAATCCA
TTAATGTGTAATTTAATTGTCTTTATGCCCAAATGATAATTAACACCAGCAAGTAAATGTGTTATTTTTTTCCTCTTATGAAGCACAGATACGATACATA
TCCAATACTGTGATACGATTATGTTAAATTTATTGGATACAATACGTGTAAGATACATGTTTAGAAATATATAAATTAAAATCTATAAATATAATAAATA
TATAATTCTAATTCTGCAGCTAGATCCAAATTAAAACATACTTCTTAAACATCACTGAATAAAAAATTATAAATCATTATCATCATTAATTAATTTCTAT
TATTTACACATAGAGAGGAACTGCCAATCTAATTCTGGAAATCATTTATAAAGAGAATAATTTTATTTTTGGTTTATCAAGGGATAATGTTTCTATATTA
TATATTATATATTATACACGGATTTCATGCAGTTGTATTGGATTTTCAAAAAAAAATGTTTTAAAAGCTTGAACACTTTACCAACACATATCAATGAAGT
ACCCAAAGTATATCGATATACGAGAAACAAATTGAATTATGGATGCTTCATAAGTTTGGGTACACTACTATATATCTTATCTAACTTTAACAACTTTTTT
ATTGTTTAATACTTAATAAAAAATATTATCAAAAGTCTTTTGATCAAATTCAAAAAATGTTGTTAAATTATAAATAAATGTTACTGATATTTTTTTTAAT
ATTTTATCAAATATTATATATAATCTGTTACTAAAATTTAAAAATATCAAAAAAAATTTAACAACATAAGATATATGAAACATTTCATAAATATCACAAA
AATATATTAATAACATTTATTCATAATTTTGTAAAAAAAATTAAATGTCGTCAAAAGCTTTTAGTAACATTTTTATTTAGTGTTGATAAAAAAATATTAC
TAAATATCATATTTTTAGTAGTAATACTTCAACAATATCTATATATACGTAAAGTATCCAATTAAGAGTATTAGTATCGTAAACAAATCGAATACGGATA
GGCAAAGCAAATTGAAGTATTGGTGTTTCATAGATTTTCTCATATGTGGGGAATCTGGTGTTTTAGTCTATAGAAGAACGAACAATTATTATTGTTATCA
TGGCTATTTTGCCATGTGACATTTATTTTTGATTTTTTAATCTTATCTATGATTCATGTGCAAAATTGCAGTTTGGTATGTCATCTGCACTGGCCACACT
TTGTGGCCAAGCTTTTGGAGCAGGACAAATTCAATCTACTTGCATTTATGTTCAAAGGTCATGGATTATACTTACTGCCACTTGTATAATCCTCTTGCCT
ATTTATATATATGCCACTCCAATCTTAAAGTTGCTTGGCCAAGATGAAGGAATAGCTAATCTTGCTGGCAGATATTCTATTCAAGTAATTCCCCACATGT
TTTCATTTGCTATAGTTTTTCCCACCCTTAGATTTCTTCAGGCCCAGAGCAAGGTTAAAGTTATTATGTGCATAGCATTTGTTGTTTTGCTCATACAAAA
TGGACTGCTTTACATCTTCATAAATATATTTGGTTGGGGTATAACCGGCTTGGCCATGGTGAGTAATATTATAGGGTGGTTGTATGCTGGGGCTTTGGTT
GTCTATACCATTAGTTGGTGCAAAGAAGAATGGAGTGGATTTTCTTGGATGGCATTTAGGGATTTGTTGGCCTTTGCTAAGTTAAGTCTTCAATCATCTG
TAATGGGTTGCTTAGAGCAATGGTATATGACATGCATTATGCTACTTGCTGGTCTTCTAGACAATCCTGTGATTGCTGTTGGCTCCTATTCTATTTGGTA
AGAGTATCGACTTCCACTAATTGGCAACTGTTTAGATTGGTTAATTAAAGCACTCAATGTTTTCAGGATTTTCATATTTAATTGAATGAAACTCTACATG
GTAATTTAAAAAAAAAAAAACTGAATAAAACTCTTTGTTGTTTGGTTTTCCTCATTTATTTACTTTTGTATTTTTAGCTTCAGCGTTCAGGGTTGGCACT
TCATGCTGCTCCTTGGAATAAGTACGGCTATAAGGTGAGTGACTATAAGGTTAAAATATATTTATAATTCCCCATCAATACTCCAATTTTGTGTTTCATC
TCTTATAATTTTTTTTCGTTGAATTCTAATAAAACAATATTTTTAATTAGATTCTTGACATTTTTTAATTTTTAATAAATTAACAAATTTTATGTTTGAT
TTTTAATATTTTTTTCATTTGATATTATTTATTTTTAAAAAGACCGATGATAAATGAAAATATTTATCTAAACACAAAATTCACATATTTGTTAAGGATT
AAAAAAAGTGTAAAAAACCAAAAATAATTTCTTTTATTAGAAAACAAAAATAAAAAAATATATATTAAAAAATTAACAAAAAATTAAATTATTTATTAAG
GATCATAAACAAATTTTAGTCTAAAGTAAAAAATACACGACGACAACAGTTTTAGGTCAAAGAAAACTTTCTGATTTAAGACTACAATTCTGGTGCAATA
TTATGTTAATATATACACTATTATTGATTAATTTAAGGAAAATAAATGTTAGAGGTTGATAATATATTTTTGGTAATATGTTTACAGTTTCTATAAATAT
GGCAAAATTTAATTTTACTTTATCTTTTTTTGTTGATTTTGATTTTATAAATATTTAAAGTTTTAAAAATGTTTTTTTTTTAAATCATGTGACATGCATT
TAAGTAGACCATATTACTTGTAATCATGGTTGTTAAACTCGTGATTCTACGTAGAATCGTGGAGGTTCCGTAAACTCGACTCGTAGAATCGAATCGTAAA
CTCGTAAGAGTTTACTCAAAAGGACAGCAACAGAATCCACTACCCAAAAGGCAGCAACAAAACCCACTACTCACCCTTGATGAAGCCAGAATTTCAATTA
ACAGATAATTTGTCCATGTTCGAAGGACCACCTTAGCATTGCCAGAATTCAACACTAAGCATCGTTTCAAACTTTCAATTAACAGAGATTTCAATTAACA
AATACTCCCTTAATTTAGCAATAGAACCCAAAACTCAACACTACAATGAAGCTAACAATAGTCTTGCTAGAATTTCAATTAACAGATACTTCCTTAATAA
GCATCATTTTAAACTTTCAATTAACAGAGATGAATTAACAAAGCTTTGAGAAAGAAAACACCTTAGCGTCGTGGAGAAAAGCTTGGAGCTAGGTCAGGAA
ATGAGGGCTTTCGATGAGTGCGGGCTTTTAAGTTTCAAACAATGATGAGGAACACCACGACGCGATTAACTCAGCAGTCCAGCACTGCAGCACGACAGCT
ATGACGACAACCACGACGACGCGAGAAGCTCAACGAGAGTCACTTTGGGGGGTTAGTAGCTTGAACAAGATAGAGTGAGTGAGTGGAAAGAGTCGCGAGA
GTCATTTCCGAGGGTTGTTGTACGACGCCGTTTCGGGTTTTCATAAACTCGTGGACTCGGAGCAGACTCGCGAGTCTATCCAAACTCGTCCGAGTCTACG
TAAAATCGGACGAGTCTACTCCGGTTTCGATTCTACTTCCGATTTAACTCGCCAACCCTTAGTAAAATCGTAAACTCGTAAGATTTTACGATTTAAATCG
AGAGTTTAACAACCATGCTTGTAATCTCATAATGGTTAGACATTTATCTAAATACATGTTACACATTATCAAATCATTAATGAAGGAATTAAATTTATAA
ATATCAAATCCAACAATGTGGAAGTTGCTTATGATTGTAGCAAAGACTTTTAGTGCAAGATGGTTGTGATTTTAAAAATTGAGGCATGATTATATCAGGA
TGGTATATATGGACAGCTTCGTGGCTTTCTACCAAAGACATATCGCAGCCATTGGATGGCAGCATTGGTGCATTATAATTTTTGTTTTGATATCCATGTG
GTGGCAAGGGTTTATACTTTCTTGTGCCTTGTTTGTACTGTTTAAACCTAGGCACTGGTGGTGATATATTTTTTTCTCTCTATAAATAAATATATTTTGC
TTATAAATAAACAATATTTTTTGCAGATACTAAATTCAAAGTTCCTGATATTTTATACAAATGAAAAACATATATAAACCTATATTACTTTTCATGTACT
CCTTTCAATTCTTATCATTAAATATTTGTTCTTTTCATGTATTCATTTCAATTCTTAACATTGAATATTTGTTCTATGCCCTCCAAGTGTTCGCATCTCC
AATGCGCTTGGCATGTCGCAGCCAAGAGCAGCCAAATACACTTTCTGTGTGACCATGTTCCAGTCTCTCCTCCTTGGTGTTCTTTTCATGAATGTTATTT
TCTTGACTAAAGAAGACTTCGCCATTATCTTTACCAATAGTGAGGATATGATACAAGCTGTTGCTGATTTAGCATACCTTCTTGGTGTAACCATGGTTCT
CAATAGTGCTTCACAGGTCATGTCAGGTGATTGTTTGCTTATGTTCTCCATCTCATGAGTATTCTATTATCATTCATTAATTCAATTTCAACATTTATTT
ATTGACATTTCCATAGGTGTGGCCATTGGAAGTGGATGGCAAGTTATGGTTGCTTTCATAAACCTTGCCTGTTATTACATTGTTGGACTGCCAATTGGAT
ATTTCCTTGGTTTCAAGCAGCATTTAGGGGTTAAGGTAAGAATCTTCTTGTTATAGAAACCATTATATATATATGACAAATTGTAATTAGTGTTCCTAGA
GCCTTGATTAAAAAATTAATTTTTTTATTGAAATATATAAAATTATATTGCTGATGACTTTTTATACTTGACAGTATGATCTTTGTAATAAATATTATAT
CGTTTAATTCCTTAATCAATGTCCTAATAAGACTCATATTTAAAGTGCAATTTGTCTTTATAACTTAAGTTATATATCTTGGAAAAGCATCATGGTTTGA
TCTCCAACTTAATTTTTATCATGATTTTCCTTTTCTTTAAATTTACACCTAAATGGTTATGGATTAGGGTCTGTGGGGAGGCACAATGTGCGGCAGTGTT
CTTCAGATTTTAATCCTCTTACTGATAATCCGTAAGACCAACTGGACCAAGGAGGTAACCAATTAACTGACATTTATTGAATAAGCATTTATTTTTTTGG
CACAGAATTCCAAATTTTTGGAGTGGGTGTTCTATATGCTAATTATCCTATAACAAAGTTATATTTGTTTATATACCAATTATTCTACCACTACAACACA
TATGATCATTAGTAACATTTGTACTATTTAATAAAAATAATACTAAAAATCTTTAGTAATAATAAAAAAATATTAATAAATTATGAATAAATATTATTAA
TATATTTTTATGATATTTATGTGTATAATTGATGTTGTTAAAATATTTTTTTAATATTTTTGTTTTAACAACATAGGTTATATATAATATTTGTTAAAAT
ATCATAAAAACATATTAGTAATATTTATTTATAATTTGACAGTATTTTTGAATGTTTCTAAAGGTTTTTAGTAACATTTTTATTAAGTATGAAAAAAATG
TTATTAAACATGATATGTGTAGTAACATACCAGTTAAGCTATTAATGTTTTGTTTTTAGCATGCATTTATGAAGCGACATGTGAATTGTGATCTTCGCTA
GAACTCGTATATAAGTAGCACAGGGTAGATGGAACAAAATATATCACTTGCTCTTGTTAAAATTTTAGCTTAATTGTACTTTTTGGTATTTTATTAATTG
TTGTTATCTTGCCAATTTAATCCCTCTTTTTATTGTTTTACAAAAAATGATCTCTTATTATTTTTATTGTGAATTTTTGTCCATCTATTAACTTGAAATA
TAATATTTCACGTAAATGTTGTGATATTACAGTGTAGAATATCATGTTATTATAGATTTACTTACATGGTTGGGCAATAGAGTGTTATGGCAGCTTTTTT
GTTAAATTGTGAATGTTATGTTAATGGAAAAACAAAAGTTGTACTATGTGAATATTGGGGTGATTAAATTGATGGAAAAATAAATAGGAGAACTAAGTGA
AACGAAAATAATAGGGTGACTAATAGAATAATTAATCATAATATTATTTGTAAGATTAAATTATGTTAACACCTCTAATTAAGATTTTGTCAAATTTCCT
CTTACATCTTTTTCTTGTTTAATTTTATATTAACTCTCATAATTATTTGAAAAAACTTTATATTGTTTCATTCTATACATTAATATTGACATACTTATCA
CTCAAGATCGAAATGCATTACACTGTGAAGTCCCAGAGTTATTGTAAACAACTAAACATTAAATAAGTAAAGAATCATGTAAAATATCATCTCTCTGAAA
ATTTGCCTGATGATTTGATTATGACTGCAGGTGGAGCAAACTGCTCATCGCATGCGAATATGGAACGTTAACAATTTCCGCTCAGACTTGGCAGAAAATG
TTACATGAATGAGAAGGAAAAAAAAACAATGTTAACAATGCACTTTTTAATCAGCATCTCTTTTGGTAATTTGGTTTGTTTGGCCACACATCTAGTGGTC
TGACAAGTTTTGTGGTAGTAAAATTTGTTTTTTCTTTTCAAAAAATAACAATACACTTTTTAATATATTTTTTGACACTATCTTTATTGTTAATTAAAAT
AAAATGTTTCTAGTTCATTTTTTTTCATCAAACTTGATTTTGATTTCTGTAT

>Glyma.09G134200 | Chr09:33368982..33372795 forward
TCAGTGAAAAGAAGCCTCCACTAATTAACGGCATATAAGGTAACCGTGTCCATATTTGTAGGATAGGAAGTTTATAACAAATTTATTTTCAGTCTCAGAA
ACTGAGTGTTAAGGGAAGAACTCCTCAAATGGAGACACCTTTAGTGACCGAAAAGTTCACTTCAGAATCTGACTACTTGCCGGTGAAAAGCTTGAAGGAT
GTTAAGTTTGTTTTGTGGACAGAAACAGTGAAGATATGGAGAATAGCATTCCCTATGGCACTGTCTGCACTGTTTCAGTTTCTCACAATCTCCTCAACTT
CCATCTATGCTGGCCATATTGGAGACATTGAACTCTCTTCTATCTCTGTTTACCAAGGTGTCATCAGTGCCCTCTATTTTTACTTGTTGGTTAGCATCTG
TCTCACCTCTCTCATGCATCGAAAGCTTAGTTGTTGTTTTATTTCTTTTGATTTCTAAACAAAAAAAAGAAAATGAAGTTACCAAATCCATTAATGAGTA
ATTTAAGTCTCTTTATGGCCAAATTGCCAAAACACACACTACCATGTACATTCTATTTTATTTTTTTTCATTTTCCCATTTGGAGAAAGTTTGGTGTTTT
GTCCATATGAAAGAACAAAATTATTTCTTATTTGCCAGATATGATTTGTTGATTTTTTTTTTATCTTATCTAATATCTATCTTTCATGTGCAAAATTGCA
GTTTGGTATGTCATCTGCATTGGTTACACTATGTGGCCAAGCTTATGGAGCAGGGCAAATTCAATCAACATGTATTTATGTTCAAAGATCATGGATTATA
CTTACTGCCACTTGTATAATCCTCTTACCTATTTATGTATACGCCACACCAATCTTAAATTTTATTGGCCAAGACCAAGAAATAGCTGATCTTGCCGGAA
GATATTCTATACAAGTAATTCCCTACATGTTTTCCTGTGCAATAGCTTTTCCCTTTCAGACATTTCTTCAGTCCCAGATCAAGGTTAAAGTTATTACTTG
CATAGCACTTGCTGTTTTGGTCATACAAAATGTGCTGCTTTACATCTTCATAAATGTATTTGGTTGGGGTACAACTGGCTTAGCCATGGTGACTAATATC
ATAGGGTGGGTTTATGCTGCGGCTTTGGTTGTGTATACCATTGGTTGGTGTAAGGAAGAATGGACTGGATTTTCATGGATGGCATTTAGGGATTTGTGGT
CATTTGCTAAGTTAAGCCTTGCATCATCTGTTATGAGTTGCTTAGACCAATGGTATAGTACATGCATTATTCTCCTTGCTGGTCTACTAGACAATCCTGT
GATTGATGTTGGTTCCTATTCTATTTGGTAAGATTATCAACTTTAACTAATTTGCAGCTGTTTAGATTGGTTCCTACTACACACTTTTCTCCATTTTTTT
TCTTCATTTTCCATTGAATTCAATAAAACTCTTTCTATTTCTTGGTTTCCTCATATATTTACTTTTGTATTTTTAGCTTTAATGTTCAGGGTTGGCACTC
GATGCTGCTTCTTGGAATAAGTGCTGCCATAAGGTGAATTCGAATGACCGACCATAAAGAAAAGAATAAATTACACTGATCATCCTTGAAGTTCCATGTA
ATTATATAAACTTTTCCTGCTTTTTCTATCCCTACACTAACCCTTTAATTGTTTGAATAACATTAAAACCTTATTCCCATGTACGAGAGGTTGTTGTAAA
TGTTAAAAAGCAGGGAGTCTTTGTGTATTTTAACAAAACCGCAGGATGATTTGTGTAATTTACTCTATATAAAATAGTCATATGGGTGCAATGTTATGTC
AATATATATGAATTTTTATTGATTACAAAGGAAACTGGATGTAACAAATTGCTTATATATTTTTCAATTCTTACACTGATTATTGGTTCTATGCCCTCCA
AGTATTCGTGTCTCCTATATACTTGGCAAGTCGCATCCAAGAGCAGCCATATACTCTTTCTGCGTGACAATGTTTCAGTCTCTCCTCCTCGGCATTGTTT
TCATGACTGTCATTTTCTTGAGTAAAGATGAGTTTGCCAAGATCTTTACCAACAGTAAGGATATGATTCGAGCTGTTGCTGATTTAGCATACCTTCTTGG
CGTATCAATGGTTATCAATAGTGCTTCACATGTGATGTCAGGTGATTGTTTGCTTTTACAGCTTGCAAAGTTCAATTTGTAACGTATTGTCTCTATCTCA
TGATTTTTCTTCAATTTATTACCATTAGGCGTGGCCGTGGGGAGTGGATGGCAAGTTATGGTTGGTTACATAAACCTTGCATGTTATTACATTGTTGGAC
TCCCCATTGGAATTTTCCTTGGTTTCAATCAACATTTAGGGGTCAAGGTAATAATGTTCTTGTTATTGATAATGTTTCTTTACTTTAATGTCAAAGGCTG
CTTATATTTAAGATAATTTGCAGTGTAATTTGCCAAATTTGTCTTTACTTTTAACAATTTGATCTCCAACTTATCCATTATGATTTTCCTTAAAATTTAC
ACACCTAAATGGTTTTGTGGCTTAGGGTCTCTGGGGAGGCACCATGTGTGGCAGGATTCTTCAAATGTTAGTCCTCTTAGTCATTATTTGGAAGACCAAC
TGGTCCAAGGAGGTACTCACTTGGATGTTTTACGATAATTAAGAAACTTTTTTTGACACCCAATGCCAAATTTTTGCATTGATGCTTTGTAAGCTAACTG
TGCTAATTATTGTGGCATGATTAACTATTAAGCTTTTGTTTTTAATATGCATCAGAACTTGCGTATAAGCCAAACAGGGCAGGCAGGACAAATCTGTCAC
ATGCTTTTCTAGAAAATAAAAAATAAAACTATGACTGATAAGCCAGATGATTTGATTTGAGGTTCTGGTATAAATTATTGTACAATATCTTTAGTTGAAT
TTTATTTTTAACTTGTGAAGACATGGAAATGTAACAAATTTTGTTTTTCTATAAAGTTCAAAATCTTTTCTGGTGTAAAATTGTTCTAATCTGCAATTTA
ATACAATAATCACAACTCACTTAAGGGTGTTTGGTAGTGTGAAAGAGAAAAAATATAAGATAATTGAGAAAAAAATATTTGAATCAAAATAGATAAAAAA
GGTGTAAAACTCATTATTTTTTGAAACTTTTCTTATGAACAATACTTTCTTTCTCACAACCAAACCAAGACTAAATGTTTCAATGCAAAACTGTTTGCTA
GCATTTGCCACCATTATATTTTCACATAAATATCAACTTTAACATCTATTTCCATTTCCTTTACTTCTCCATTTTTGTCCTATCTCTGTCTTTCTTATCA
TATCACTTATTATATCAATCACCTTTTCTCTCTTCTTTCTTCTATCCTCTTTTTATCTCTCTGTCTTTCATCCATGCCTTGTGACTTATGAGGAATATGA
CTGCAGGTGGAGCAAACAGCTCATCGCATGAGAATATGGAGCATCAACAACCTCCACTCGGATGCCATGGGAAATGTTACATGAGAAATAGCAACGTTTC
ATTTTACTGAAATTAATAAAATTATGTAAAAAATTCATTTAATAAGAAATGAATCTTACATAATTTTATATTTTCTTCTAAACTTCAAGATAATAACATA
GAGTATGTTTAAAAGAATATGGTAAAAGAGTGTGCAGTTAAATCCATTTATGGTTTACATATGTCCCTTTACTTTGGTTGATTGATCTAGTAGAACTGTA
GAAGACGACTTGTACACAGTCACTATGTAGAGTTAATGGACCCACCAAAAAAAAAAAATGTAGAGTTTATGGAGTCACGTATCAATAAACGTTCATCTTT
GCCTTTCTTTTACA

>Glyma.16G180200 | Chr16:34077613..34081734 forward
TGAATACTGTTTATCAATGTATGTCATGGGCTCATGGCTACCTAAAATACTGTTACAAAACAAAGCTTCTTGCTACTTTAAATCGAGTGCAGATACTATT
GAAAAATCTTATACTGTTACAAAGCTTCTGCCAATCCAACTTTTGTGGACTAGTAAAGTATTTTCCACTACGCTCTAAACTTTTCATCTATGATTCTTTA
TTTAAAATATAAATGATTTATTCCTGTGCGCACATGAAATTCTCGTTTTTATGCTCCACATTGAGGACCATAATAAAACAAGAAAGATAATTACATTTTC
AGTGAAAAGAAGCCTCCACTAACGCCATATAATGTAATCTTGTCCTTATTTGTAGGATAGGAAGTTTATAGCAAACATTTCAGTCTCAAACGGAGTGTTA
AGGGAAGAACTCCTCAAATGGAGACACCTTTAGTGATCCAGAAGTTCACTTCAGAATCTGACTACTTGCCGGTGAAAAGCTTGAAGGATTTGAAGTTTGT
TTTGTGGACAGAGACAGTGAAGATATGGAGAATAGCATTCCCTATGGCACTGTCTGCACTGCTTCAGTTTCTCACAATCTCCTCAACTTCCATCTATGCT
GGCCATCTTGGAGACATTGAACTTTCTTCTATCTCTGTTTACCAAGGTGTCATCAGTGCCATCTATTTTGACTTGTTGGTTAGCACCTCTCTCATTCATC
AAAAGCTTAGTTGTTATTTGTTATTTGTTTGTTCTTTTGCTTTTAAACACAAAATGAAGTTACCAAATCCATTAATGAGTAATTTAATTCTGATTTTCTT
TCATTTTCCCATGTGGGGAAAAGTTTGGTGTTTAGTCAATATGAAAGAAGAATTATTTCTTGTTCCTCTTCTTCTTATTATTATTATTTGCCTGATATGA
TTTGTTGGACTCTTTATACCTTATCTAACTTTAATGTGCAAAATTTCAGTTTGGTATGTCATCTGCATTGGTAACACTTTGTGGCCAAGCTTTTGGAGCA
GGACAAATTCAATCAACTTGTATTTATGTTCAAAGGTCATGGATTATACTTACTGCCACTTGTATAATCCTCTTGCCTATTTATGTATGTGCCACTCCAA
TCTTAAAGTTTATTGGCCAAGACCACGAAATAGCTGATCTTGCTGGAAGATATTCCATACAAGTAATTCCCTACATGTTTTCCTGTGCTATAACTTTTCC
CTTCCAGACATTTCTTCAGGCCCAGATCAAGGTTAAAGTTATTACGTGCATAGCACTTGCAGTTTTGGTCATACAAAATGTGCTGCTTTACATCTTCATA
AATGTATTTGGTTGGGGTACAACTGGCTTAGCCATGGTGACTAATATCACAGGGTGGGTTTATGCTATGGCTTTGGTTGTGTATACCATTGGTTGGTGTA
AGGAAGAATGGACTGGATTTTCATGGATGGCATTCAGGGATTTGTGGTCATTTGCTAAGTTAAGCCTTGCATCATCTGTTATGAGTTGCTTAGAGCAATG
GTATGGAACATGCATTATACTACTTGCTGGTCTACTAGACAATCCTGTGATTGATGTTGGTTCCTATTCTATTTGGTAAGATTATCATGTTGAACTAATT
TGCAGCTGTTTAAATTGCCTATAATACTCACTTTTCTCCATTTTTTGGTTTTCCTCTTGTTATTTCTTGGTTTTCCTCATATATTTACTTTTGTATTTTT
AGCTTCAATGTTCAGGGTTGGCACACGATGCTGCTCCTTGGAATAAGTGTTGCCATAAGGTAAATGACCATATAGAAAATAGTAAATTACACGGATCATT
ATTGAAGTTCCATGAAATTATACAAACTTTTCATATTCTTTTTTTACCCCTATACTAACCCTTTAAATGTTTGAATAGCATTAAACCTTTTTCCCCTTCC
TAGATGTTATTGTAAATATTACGAAAGTAAGGAGTCTTTGTTTATTTTCACGAAACCTTAGGGATGATTAATTAGTGTAATTTACTCCATATAAATATTT
CATATGGGTGCAATGTTATGTCAATTTATGAACTTTTATTGGTTACAAAGAAACTGAATGTAACAAATTGCTTATATATTTTTCAATCAATACATCGATT
ATTGGTTCTGTGCCCTACAGTATTCGTGTCTCCAATACACTTGGCATGTCGCATCCAAGAGCAGCCATATACTCTTTCTGCGTGACAATGTTTCAGTCTC
TCCTCCTTGGCATTGTTTTCATGATTGCCATTTTCTTGAGTAAAGATGAGTTTGCCAAGATCTTTACCGACAGTGAGGATATGATACGAGCTGTTGCTGA
TTTAGCATACCTTCTTGGTGTATCGATGGTTATCAATAGCGCTTCACAAGTGATGTCAGGTGATTGTTTGCTTTTACAACTTGTAAAGTTCATTTTGTAA
TATATTGGCCCCATCTCTTGATTGTTCTTCTATGTTTACCATTAGGTGTGGCCGTTGGGAGTGGATGGCAAGTTATGGTTGGTTACATAAACCTGGCATG
TTATTACGTTGTTGGACTCCCTATTGGAATTTTTCTTGGTTTCAATCAACATTTAGGGGTCAAGGTAATTAATAATGTTCTTGTTATGGGAATTGTTTCT
TTACTCCAATGTCAAAGGCTGCTTATATTTACATAATTTGCACTGTAATAAATTGCCAAATTAATTTGATCTCCGACTTATCCATTATGATTTCCCTTGA
AATTTACACACCTAAATGGTTTTGTGGCTTAGGGTCTCTGGGGAGGCACCATGTGTGGTAGGATTCTTCAAATGTTAGTCCTCTTAATCATTATTTGGAA
GACCAACTGGTCCAAGGAGGTACGTACTCACTTGAATTTTTCCAATAAGCAGCTTTTTCTTTTTGCACTCAATTCCAATTTTTTGCATTATTGATGTTTT
GTATGCTATCTGTGCTGATTATTCTACAAAGATAAACTATTACGTACGCTTTTGTTTTAATATGCATCTTGAAGCGCCATGTGATTTTTGGGTTTTAATA
TTGTTTAGGTGTAATTTTTTCCAAATTTACCGATTTGGGGAATTATTATTCTTTGGAATAAGTCGTAATGGGTTTTACTTTTTTTTAAAAAAATTTAAAT
ATTTTTTATTTAATTATTTAATAAATACATGTTTCTTTTTAATTTTATTTAATATTTTGTATATATTTTATATGATTTTATTTAATATTATATATTTTTT
AATATTATTTTATATTTTTTTTTACTAATTTTAAAGAATTTGATTTGATTTGTAAATGAAAAAATAATATAAAATACTTAAATCAAATAATGAAAAATAA
CATAATCACTTTAGCCACAGAACAGAGGTACTTGCTTTGTATTCCCTTGTAAACAATTTTCATAAAAGTGTGTCTAGCCTGCTGTGCAAATCGCCAGTGA
GACTGACAATATTCATTGCTGGTCAAATCGCTAGCTGCAGTGGCGAAACCACTGGAATCGTCAGTGAGAGTGGCGTTTTTGTTGCTGGAATCGTCAGTGA
GAGTGGCGTTTTTACCACGTATTACACGCAGGAGTTTCTCGCCAGTATCATTGGCGATTTACATGTCTTTTAAGTGAAATAACAGACCCATCGTATAAAT
TGTTTAGAAACAGACCCATTGTAGGAAATAGTTTTTAAAAAGAACCCCATTAGCATAATTTGCCCTATAAATTATTGTACAATATCTTGGCAAATCTATG
TCTTTCTTTCTTATAATATCATTCACTTTCTCTCTCTCTCTCTCTTTTTTTTTTTTTCTTCTTTTTATCTTTCTCTTTCTCTCATCCATGCCTTGTGACT
TATGAGGAATATGACTGCAGGTGGAGCAAACAGCTCATCGCATGAGAATATGGAGCATCAACAACCTCCACTCGAATGACATGGGAAATGTTACATGAGA
AGGAGCAATGCTTCATTTTTTAATATATTTATTTACCACTCTCAAATTTGTTTTTATTTTCACAACTAAGAAAATAAACTAAAACCAAACAAGTTTTTAT
TTTGGCTCCGGCATTGATCGTTTTTCAAAACAGTGCTGCTACATGGTGTGAATGGATGGAGTACCAAACATGAATTAATCTGCCTTATTATTACTCCACA
ATAAAAAGGTGACGTGTTATTG

>Glyma.10G231800 | Chr10:46138896..46139641 reverse
ATTTTATTATGTTTCAAATGGTGGTGAAGTCCTTAGTCCATGTGAACATATCAATTTAGATTATGTTATTGAATACAATGAAACTCAACGTAAATACACC
CTGGTTGCTCTTAATGTTGATTTCCTTGTGCTAAAATTAATTTTGGAATTGTGTACCCTCTCAGTGTTCGAGTCTCCAATAATCTAGGAATGCAACATCC
TAGAGCAGCCAAGTATTCTTTCTTGGTGACAATGTTTCACTCTCCTCTTTGGGGTGGTGCTGATGATTACTGTTTTCTTAAGTAAAGACAATCTTGCTAT
TATCTTCACAAGCAGCAAGGATATGCAATTAACTGTGGCTGATCAAGCATACCTTCTTGGTCTAACGATGATTATAAATAGTGCTGCACAATTGATGTCA
GGTATCAATCAGCTTTGGCTTCTTCAAAACTACTTTATTTTGTTCAACTTCATAGTCTGCTGCATTAGTGTTATTTTAGTAGCGTTTGTTTTGGTGTAAT
TTCTCTAGGTGTTGCCATTGGAAGTGGATGGAAACTGACGGTGGCTTATATTAATTTAACCTGCTATTATATGGTTGGACTCCCTCTTGGATTTTATTTG
GTTTCACATTGAATTTAGGGGTCAAGGTAAAATGCTGTATTTCCAACTCACTTTCATGTTTATTTTCTTTTGATCTAAAGACTTCCTCTTCTTTTGATAA
AAAAGAGATATATTCAAAGGAGAAGATGGTTCAAAAATTATACTAT

>Glyma.17G175100 | Chr17:18294542..18302075 forward
ATGGGGCTTATCACATATGGAAAGTCAGCAATATCCACGTATTTCTTAGGCAAACTAAGCAAAGAGGCATTAGTAGGTGGAAGTCTAGCCATTGGAGTGG
CCAACATAACTGGTTATTCTATCATTTCTAGCCTTGCAACAAGCATGGATGGAATATCCTCCCAGGCTTGTGGGGCCCAACAATGGACCCTTATTGGTCA
AACTCTCCAATGTTCCATCATGATTTTGACCCTAACTTGCATTACCATTTCAATCTTATGGCTCAACATTGAACCCGTCCTCCTCTTTTGTGGCCAAAAC
CCCACCATATCATCTATCGCCACCACCTACCTTGGATTCTCTCTTCCTGACCTTATTTTCACATCCTTAATCATCTCTTTCAAGATTTTCTTGAGAACTC
AAGATGTGACCCTCCCTTTCATGTTTAGTGCAACTTTGGCACCATTTTTACACGCCATCATCAATAATGTGGTCATTCACACTTTTGGCCTGGGAATTCA
AGGGGTTGCCTTGGTTGGATCCTTTACCAATATAAAATTTCTTATTATCCTTTTGCTTTACCTATGGTTCTCGAGGAATTGTTCTAACTCCTGGCAAGGT
TGGTCATATCAATGCTTCAAGCAATGATGGCCTATTCTTCGTCAAGGAATCCTAAGTTGTGTTTCTGTTTGCTTAGAATGGTGGTGGTATTAGGGGTGGG
TAAGCGGGTCGGCCTGCCCCGCATAAGGCCCTCCCGCCCTACATTCTTACATGAACCAAATAAATTGGTCTGCCCTCGCCCCGCGGACCCGTGGGTCAAA
CAGACTTGTCCGCGGGCCTAGTTTTAAAAGAATTTCAATTTCAACGAAAATACAATACAATCAAATTAAGTTCAACACAAATGTAAATAAAATATTAACA
ATTAGTCAATTACATCAATAAAATAAATAATGTCTTAACAAAATAAAATCCAAGCAATAAATCTAAAATATGAATGTTAAACATCTCCAACAACAAATGA
TTCCATATTTGAAGTAGTTTGAGCCTTCTCCATCTCCACATCTTCATCTTCTTTTCCTAAAACTCAAAATAATAATAAATATTAGAATAAACTCAAGTTA
AATGATTAAAAGACAAAAATTATTACACATTATTTACCTTGCATATCAAATCCCAGTAACCAATTTTTAGTACATATCAAGGCTTGCACGTTGTCAGCAA
AAAGCGTAGTTCGATATTTGTTAAGCAACCGTGAGCCAATACTAAATGTTGATTCGGAAGCCACCATTGTAATTTGGATGCTTAAAATATCACAAGCCAA
TAATGAAAGATCTGGAAATCGAGCTTGATTGTCCTTCCAATATTGCAAAACATCAAGATTGGGATGATATTTATTTGAAAGATTTGCCTCTTCCAAATAT
GTATCTAGTTGAGATTTACCCACTTGCGACATATCTTCATCTTCAAATTGTATAAACTCCTACATTAATTCCACAAAAATAAAAAGTAGTTAGTCAAAGT
AAATAAGAATAAATGATTGGCAGTTAAAAAAATTAATAGAAACCTTGACTTACATCCATAACATCAACTTGAGCAATACTAATAGTACTAGTAGTAGCCA
TAGTCTCTTGTGATGAACCTTGACTTAAACCAAAATTGCTTTTTGTTTCTTTGGAATACATTTGAACATACTCATTATATAGAGTATACAACTTGTGCTC
CACAACTTTCAACTTTGCTTGACAAGATATTGGATCAAGACCAAGTTTTGAATAACAATACTTCAGAAGTTTGATTTTAAAGCGTGGATCTAGAATGCAC
CCAAAGGAAAGTACATTGCTATAATCACTCCAATATTTATCAAACTTTGTTTTCATATCAATTTCCATTGTTCTAATCAACTCATCTTTATTACTCAAAT
TTTGAAGCAATAAACATTCAATTTTCCACACTTGCATGAAATACAAATTAGATGTTGGGTAAGTGGAACCAGATGTCAACTATGTGATTTGAAAAAAAGG
ACGCAAAAAATCACACATTTTGTGTACTCTCTCCCATTCTTCATTAGTAGGACAACTTGAATAGCTCCTATCATCAAATGCAAGACTACAAAAAACATGT
CGATATACAAGGGCACTCTTAAGCATCAGAAAAGTAGAATTCCACCTAGTAATGACATCTAAGTGCAAACCCATCTTTAAAAACTTTCATTCTACCCTCT
GATCCCTTAACATACTTAATGCTTTCTCTAATTTTGTTTATAGCAAGATTGGCTACTTTCAACCCCTTTTGAACAATAAGGTTTAAAATGTGAACACAAC
ATCGGATATGAAAAAATTCACCACCACTTACTACACCATTAGTATGCAAAAGAATTCTTTCCTTCAAATAGTCTTGCATTTTATCATTGGAAGAAGTATT
ATCTAGAGTTAATGAAAAAAATTTATGCTCAATCCCCCATTCTTCCAAAAAACCATATATAACTTTAGTCATCTCACGCCCCAAGTGTGGAAGAGGAAAA
TGAGAAAAATTAAGTATTTTACTATTCAACTTCCAATTTGCATCAACATAATCTGCAATTAATGAAATATAACCTGCAGAAGTACAAGATGTCCACACAT
CAGATGTCAAGCTTATTTTGCTAGGGACTTTAGACAACATGCATTTTATTTTTTTCTTTTCAGAATCATACAAATTGTTCACATTCATAGTAGCAACACG
CCTAGAGGGTACCTTCACATCAGGATGCAAATATTGTAATAACTCTTTAAACCTTCTATGTTCAACAAAAGAGAATGGAAGATCATGCTCAATAATCATC
ATAGATATCATCTCATGTACCACACTTTGATCTATTTTTTTTATTTCTTAATTTCCCAGCATGATCGAGAATAATATTTCCAACATCTCTATTAGAATGC
GTCTTCAAATATACATCACATTTCCCCATATGATGTTGTAAGGTTGAAGTCCCATTCTTATTGTCACCGCCCACATAATCTTTCAAACAATATTTGAATT
TACTCCTCACTTTTCCATCACTATGCATAACAGGTTTCTCAAAAAAATTCCAAACATTTGATGAATAATTTCTAGCCCTCTTTTTTGACATTTTAGACTC
AATATGTTCATCTTCATGAATAACATTTTCATGCACATCCACATCAATACTCTCATTGTCATTCTCATCAACATTAACTTCAAAAGAATCCATATTTAAA
ATTTGAAATAATAAAATTAAGTGAACTCAATTCTAAATTCAAATAAATCACACAAAAACTCAATTCATCATGTTTATAACTTGTAGATCAAGAGTTTCGT
GGTTCATTCCCTTATCCCATGATTTTATCATATAATAATAATTAAAGAATCACTACTAATAGAGATACAAAATACAAATTGTTTCCGCAATACTATGTAA
AATTTCCAATTATATAAATGAAAATATCACTAGTAATAGAGATACAAAAGACAAAGTGTTTATCCACCCTGAATTCTAAGAGAACATATTAAAGAATCAA
CTAAGTATAATTCAGTTGATCTCTATGTAATATTGTAGAAATAGAAAGGAAATAAATAATTGACAAGTGAAGTGTGATGGGTGTAGTAAGTTTGTGAAGA
CAAAAGTATCTCTGCAACAAAATTGACAAAGAAGGCATAGAATTGAAGAAGAAAGTACTTGAAAGAAAGGAAGTGTAATAGTAGGGGCCTACGTTTGTAT
TTTGTATCTCTACTAGTGATATTTTACATAGACTGTGAACAGCTATACATGCTCAATGATGGAATAAAATTGAATGTTATTATTCTCATTTTGAAGAAAA
TTGATTACACTATAATTTATTAAAAGAAGATATTTTGCCATGAATCACAAATATTAAAAGCAGGAAAGTGAATAGGATAGGATAACATCACAAACAACAG
AGAAAGGAAGAGAAAAAAAGGAAAAAAGGTGAGTATGAAAGCAATGAACCTTGAAACGGAAACAATGCCAACAAAGGGAATGAAGCAATGAAGCCAAGGG
TTAAAATAAAAAAAAAAAAAGAGGTGTGAGCAACGGACCAACAGTTATAGATTTTTCACATTTTTCTTTTATTTTAAGAGTACAACAACAATGAATCAGA
CCCAATTTTGGAAAAAAAAGCCCCAACAAAAATAGGATAAAAACAAATTCTAGAGAGAGAAGAGATGTACTTTGTGTGGTGGCGCGGTGGTGGGCCGAGG
TTGTGTCGCTGTGGTGTGCCGCGTGACTGCGTGAGCATGAGTCGCATTTTGTTTTCCTTGAAAGGGAAAGAGTCGTGTGACTACGCGCATCACGTGAATG
CGTGTGGAGAAGAAATGTTTTGCTTCAACCGTGAGGAGAAAAAGATGAAAAGAAGTGTTGTTAGCCTACAACCATGAGTCCGTGAAGAGAAGAAGAAAGA
AAAGTTGGGGTATTGGTCAAGTGGTCCTTAAGTGAAAGAGAGAAAATCAAACCTAATGTGGGTCACACAAAATAAAACAAGGTTGTTGCTGTAGATGTGA
TGTGAGATTCTTAAATGGATTAAGCGGGCTTATGGGCAACCCATGGACCCCCCAGACCAAACCTGCATAGCCCACAGATTAAGCGGGATGGCCTAAAAAT
ACAACATAACCATGGTCTGTTTAAAAAAATCGGTCCGTTACCCGTACAGACCGCGGGCCTCGCGGGTCAGTCCCATAAACCTAAGTCCATTTACCCACCC
CTAGGTGGTATGAGTTATTGGTACTCTTTTCTGGGGTCCTACCTAATGCAACTAAAACAATAGCCACCTATGGAATAATAATTCAGGCAACCTCACTTAT
CTATAATTTTCCTTATGCATTAAGTTTGGCTGTTTCACCAAAGGTAGGGAATGAACTTGGAGCTAATAGGTCTGACAAGGCAAAGGCATCATCATTCTAT
GCATTACTATGTGCTTTCATCACGACCATTGTAGCTACAATATTGACAGTAAAGTATGACTTATGCCTATGGACAAATGTCTATTGAATATGAAGCTATT
CTTTCTCTCACTGCAACAACATTGCCCATTGTGGGTCGGGAGTTGCTAGGTGCACCCAGCAATTATGTGAATGGACAAAACTGTCCTTGTTTAAAAAACG
TGATTTTCAACTGCGGAAGAACCTTCTTCCGTGCCATCTCGCGGAAGAACTTCTTTCGTAGGAAGCCACGGAAGAAGGTTCTTCCGCAGTTGAAAATAAC
AACAGCAGAAGAACCTTCTTCGGTGAGTTAATTTGTTTTTTTGATTTTTAAATTTTTGGATTATTTTGTATTTTACTATTACAAAATTTAATGCATATTT
AATTCGGGTTATTATTACAAAATAAAAAAAATATTTATTTAATATATATTAAATTTTGTAATAGTAGAAGAATTTGTGATAGTAAATATTTTTTATTTTT
AAAAAATATATGAATTAAATATTTTTTATTTAATTTGGGTTATCATTATAAAATTTAATGTATATTTAATTTGGATTATTATTACAAAATTTAATGCATT
AAATATTTTTGTAAACGTAACCCGAATTAAAGCATTAAATATTTTTTATTTAATTCGGATTACTATTACAAAATTTAATGCATTAAATATTTTTGTAATA
GTAACCATAATTAAATATGCATTAAATATTTTTTATTTAATTTGATTTACTATTACAAAATTTAATGTATATTTAATTCGGGTTATTATTACAAAATAAA
AAATATTTATTTAATATATATTAAATTTTGTAATAGTAAAAAATTTGTGATAGTAAATATTTTTTATTTTAAGAAAAATATATACAAATTAAATAATTCG
TATGATTTATATCAAAATTAATTATCATAAAATTTATTATTTAAATTTACATGTGCATTAAATATGCATTAGAAATTGTAGTAATATGTATAGTAACATT
ATTTATTTTATAACCTAATTGACTCATTATTCCAGTTGATTATAGCGTTGTGCTAATAACCTATATATTGTTTAATTTGTATATTTTTTAAAAATAAAAA
ATATTAACTATCTCAATTTTTTTTACTATTACAAATTTAATGTATATTAAATAAATATTTTTTATTTTGTAATAATAACCCGAATTAAATATGCACTAAA
TTTTGTAATAGTAATTCGAATTAAATAAAAAATATTTAATGCATATTTAATTATGGTTACTATTACAAAAATATTTCATGCGTTAAATTTTATAATAGTA
ACCCGAATTAAATAAAAAATATTTAATGCATTAATTCGGGTTACTATTACAAAAATATTTAATGCATTAAATTTTGTAATAGTAATCCAAATTAAATATA
CATTAAATTTTGTAATAATAACTCAGATCAAATAAAACATATTTAATTCGTATATTTTTTTAAAATAAAAAATATTTATTATTACAAAATTTAATATATA
TTAAATAAATATTTTTTATTTTGTAATAATAACCCAAATTAAATATGCACTAAATTTTGTAATAATAACCCGTATTAAATAAAAAAAATTTAATGCATAT
TTAATTATGGTTACTATTACAAAAATATTTAATGCATTAAATTTTATAATAGTAATCCGAATTAAATAAAAAATATTTAATGCTTTAATTCGGGTTACTA
CAAAAATATTTAATGCATTAAATTTTGTAATAATAATCCAAATTAAGTATACATTAAATTTTGTAATAGTAACCCAGATTAAATAAAAAATATTTAATCC
GTATATTTTTTTTAAAAATAAAAAATATTTACTTTCACAAATTTTTTTACTATTACAAAATTTAATATATATTAAATAAATATTTTTTATTTTGTAATAG
TAACCCGAATTAAATATGCACTAAATTTTGTAATAGTAAAATACAAAATACAAAATAATATAGGCAAATATAAAATAATCCAAAAATTTAAAAACCAAAA
AAATAAATTAACTCATGGAAGAACCTTCTTGCATGTCATCTCGCGGAAGATTATATATAATCATAATTCATGTTAATTCAATCATAATTAGCATGAATTA
TGATTATATATAGATATATCCTCTTTTAGGGAATTATGATTGATTAACATAATTGATTGATTAGCATGAATTATGATTATGATTGATTTATTAGGATGAA
TTATGATTATGATTGATTGATTAGCAAAATAAATATCTTTAAATATCTTGTATAATTAACAAAATTTTTATTTTTCCTCTTACGGATCAAGTTGATCCGG
AAGTTACGGATCAAGTTGATCCGTAAGAGAAAAACCAAGGACAATTTTGTCATTTTATTAGAATGCTGGGTGCACCAACAATAATGCTGGGTGCACCTAA
CAACACTCGGTACTAAGTGGGAGTGCTAGACCTACTTTGGGTGCCAAGATAAACTTGGTCTCCTTTTATGTTGTGGGCTTACCTGTTGCTCTTTTGATGA
GCTTTGTATTTGATTTGGGCTTATTGGGACTTTTATTGGGCCTGTTGTTAGCTCAAATAGTGCGTGCTAGTGTGATGACCATTGTGTTGGCAAGAACAAA
TTGGGGAGCAAGCACAGAGGGCAAGGGAGTTTAA

>Glyma.09G257400 | Chr09:47641273..47645269 forward
TCTGCAACCAATGAAACAACAAACGAAACTTGCTCAAAACAAACAAATAAGGAAGGGAAAAAAAATAGAAACAAACAAAAACCACTTTTCTCCTTGTTTT
CTTAATTTTTTCTTCTCTTGCAAGAGTTAAGCTTTTTCATGCAATTCTAACACCTGAAAGAAAGAAAAAAGGGTTCAAAGAAAATCTGCCAAAATAGGTA
AAGGAAGCAAGGAACAGAATGGAGGAGGAGAGAGCAGTAGCTCATCCTCCTCTGATGAAAACTCTCCTAGGAAAGGCACAACAAAGATGGAGACACCATT
AATGTTCAACAGTGATCTCAATTCCACGGGGACCACAGAGCTCCAATATGCTCCTGAGGGTCTAGTTGACACAAATGGAGGAGACTACACTGAAATGAGT
GGCCTTGCAGATTTCAAAAATGTGTTCTCTGTGGAATCCGTAAAGCTCTGGACAATTGCTGCACCCATTGCCTTCAGCATACTATGCAATTATGCTGTCA
ATTCCTTCACAACTATCTTTGTTGGCCATCTTGGAGATTTAGAACTCTCTTCAGTTTCACTCTCTCTCTCTGTCGTTTCAAATTTCTCTTTTGGCTTCTT
GGTTAGCTTCTAACTTCTATCAACTCTTGGATTTTTCCCCATGCTTTTTATATATACAGCAATATAGACTATTCATGCTTGGTTTTGCTTTTTTTTTTTT
TTGCTTGATTTCTTGAATGGTATACGCCCATGAATACGTGACTTCCACTTGTTACTAGACAATAGACATATTCTAGTTGAATTATAGGAAAACCTTCCCA
ATTGACCAAAATTTTGACTTCAATTAGCAAATCAACCAAGGAAAATATTCAGCATATATACGCCCATTTTCTTTTCAGCTTATTTGTATAGACATGCACT
TTTGAGGTCCAAAGTATTGTCACTTTCTGGGAACAAATTGTATATTTTAACCTCTTTTTGCTCATGTTTAAAAAATGGGGCATTGAATTTTTGCATCAAA
TTTGCAGCTTGGTATGGCAAGTGCACTAGAGACTTTATGTGGGCAAGCATTTGGTGCTGGACAAGTAGAAATGTTAGGGGTCTACATGCAACGTTCTTGG
TTAATCTTATTGGGTGCATGCATCTGCCTCACACCAATTTACATTTATGCTGAGCCAATCTTGTTACTCCTTGGACAAGAACCTGAAATTGCAGAGTTAG
CTGGTGTATTTACCATTCAATCCATCCCTCAGATGTTTTCTCTAGCCATCAATTTCCCCACCCAGAAGTTCTTGCAGGCACAAACCAAAGTGGGATTTCT
TGCATGGGTTGGTTTTGGAGCCTTTATTTTTCACATTATACTTCTATGGATTTTACTTAAAGTGCTTGCATTGGGTACAACTGGTGCTGCTGTAGCCTAT
AGCACAACAGCTTGGGTTATTGCTTTGGCTCAAACAGCTTATGTGATTGGTTGGTGCAAAGATGGGTGGAGAGGCTTCTCGTGGTTGGCATTCAAGGATC
TTTGGGCCTTTGTGAAATTGTCTGTTGCTTCAGCAGTCATGCTATGCTTAGAGGTTTGGTATTTTATGATCTTAATTGTGCTCACTGGACACCTTGACAA
TGCAGTTATTGCTGTTGGTTCTCTTTCAATATGGTATGATATCAAATCCAAACTACTAATACATTTCCCCCCTATCTTCTTAACGAACCTTGAATTGAAA
ACAAATCTAGGACTTAACAAGTTCTTTTCGCTTCGTTTTCTGTTTTCTAATATTTGTACGAGAAATAATGAAAACAATAAATATTATACATAAACTAATT
TTAAACTTACACGGGAGAAGCTGATTCACTTTATTTTTTTATTTTTCTCTTGTAAGTACTTATTGATAAGTTTATCCAATTACGACTCCAGGACGACGTG
GAACAATATTAATTATGTTATTTTCTCTTAAATAGTCTTGGTGCTGATTAGATTTTTGTTCTGCTTATCCTCTTTCAGCATGACTATCAATGGATTTGAA
GGCATGTTATTTATAGGGATCAATGCAGCAATTAGGTAAATTCTTGATTTATGGCTGATTTTTTTCCCCATCTTAGTGTAAAGGTTATTTTACAACTTCT
ATAGGAATGATGTATTTTTATTTTTATTTTCACTTTCTCAGTGTGAGGGTTTCCAATGAGCTTGGATCAGGACGCCCAAGAGCGGCAAAATATTCAGTCA
TTGTCACAATTATTGAGTCCCTCGTCATTGGGTTAATTTGTGCAGCCATTATTTTAATAACAAAAGATCATTTTGCCATCATTTTCACTGAGAGTAAAGA
GATGATAAAAGCAGTTTCTAAATTAGCAGGCCTTCTTGGGTTAACCATGATTCTGAATAGTGTTCAGCCAGTTATATCAGGTAAGAGGATCTTAATAACT
TCTATGATATAAGTTGAGTTGAGATTGCCTAGACTTGCTCTACTTTTTCAACGTTTAAAGCAACCTCTCTTATAAGAACGCAGTATATTTTTTAAAAAAT
AATTAGAGGGATTAGTATATATAATGTATAAGAGAGATCCGTGTAATGTTTTTCAAACAATTAAAGGAATTAATGTAAGTGTAGAAAAAATGGAAAAATT
TTGTACAATCTCACAAAACTTCAGGAATATCAATATAATTTACTCTATAAATTATTACTATGTAATAAGCATAATTGTGAAATGTTTCTATATTCTTGCA
GGTGTTGCTGTGGGAGGAGGGTGGCAGGCTTTGGTGGCTTACATCAATCTATTTTGTTATTACATCATGGGACTCCCTCTTGGCTTCCTTTTGGGTTACA
AGTTGGGTTACAGAGTGGAGGTAAAGTACACCATTATCACTCAAACCAAAACTAACCAAAAGATATCAATTTTGTTTTCATTCTCCCTTGGGGTGAAGGG
CATCTGTCATCCTGCTGAAATTTCATCAATAAGTTTAGAGTAAAATATCAATTAAATCCTGAAATTAGAATCACTGGCCCATTTGATCCAATCAAATCAA
ATTTATTTCTTGACTTTGGACATTACATAAAACTAATTAATAAATTATATACGTGACATTGTAAGTTTATTAATTTTTATAATAGTCTTTTTTTGATAGT
TTCTGATTAGTTGTCGTTCTAAAAACTTTTACACTCGTAAAACATGTTTAATTGATTAAATCAAACTAATTTGATCCTTTACTTAGACCATTATCATTCA
ATTTAGTTTTTGGATTTAATGTACATTAATTAGTCAATTCGATTCTTATTATTAGTGACCAAATTAGGTTATGGTTGTCAACTTAAGGAACTAAATTGAG
TGATTTTGTAATATTAGATACTAAATTGACTATGGGTTGTTTCAGGGTATTTGGGTTGGAATGATTTGTGGGACAATATTGCAAACGCTCATCTTGTTGT
ACATTGTCTATAAAACAAACTGGAACAAGGAGGTGAGAACCAGTGCTTTTCTGGGTAACCATGAAATTAATATTTCATCAACTAATATAATTGACTAAAA
AATGAACAAATACAAGTATATATTGCATCTTCTTCTTTTCTTTCCTAATTAAGCTCAAATTTCAGGTTGAGCTAGCATCAAGTCGAATGCGGAAATGGAC
CGGACAAGAAATTGAAATTAATTTGTAAAATTAACGAAACTCAAACCCTTTTATAATTTTGTGTATATTTGTTATGTGACAAAGATGTTCATGTCTTAGC
TATAGCAGAAGTTTCTTATGTTTTTGTTTGTTTTTCTAATGAAGGGCAAATTCTCAGCAACCAGTCACACATTAATCTCTTTCAAACCAAGAGATGGCTG
CATAGAGATGGCTGAAGTGGACTTTATCTCTCTTAACGAATATTTTTTCATATATAGTCAGTGATCGATATGCTCAAGAAATTTAATCACCTGCAAATTT
TGTTAGACTTTTTTGGTGGACCAGCTTTCTTTTCTTATGTGATAATTATCACAGTTTGTATTCTTATCTAAAATTTTTATAGGTTCTTTGAATACTC

>Glyma.18G235200 | Chr18:52365873..52369870 reverse
AGGCACCTTTTTTTCTTCTCTTGCATGAGTTAAGCTTTTTCATGCAATTCTAACACCTGAAAAACAACAAAAACAAAAAAGGGTTCAAAGAAAATCTGCC
AAAATAGGTAAAGGAAGCAAGGAGCAGAATGGAGGAGAGAGCAGTAGCTCATCCTCCTCTGATGAGGCACCATTCATGTTCAACAATGGTTTCAATTCCA
TGGAGACCACAGAGCTCCACCATGCTCCTGAGGGTCTTGTTGACACAAATGGAGGAGACTACACTGAAATGAGTGGCTTTGCAGATTTCAAAAACGTGTT
CTCTGTGGAATCTATAAAGCTTTGGACAATTGCTGCTCCCATTGCCTTCAGCATACTATGCAATTATGCTGTCAATTCCTTCACGACTATCTTTGTTGGT
CATCTTGGAGATTTAGAACTCTCTTCAGTTTCACTCTCTCTCTCTGTCGTTTCAAATTTCTCTTTTGGCTTCTTGGTTAGCTTCTAACTTCTATCAACTC
TTGGATTCTTTTCCCATGTTTTTTATATACACAGCAATATAGACTATTCATGCTTGGTTTATTTTTTCTTGATTTCTTGAATGGTATGTCATGTTTACCT
TTTTTAAGAGCCAAACTTATAAACTCCCCACATGAATATGTGACGTCCACTTGTTACTAGACAATAGACATATTCTAGTTGAACTATAAGAAAACCTTCC
TAATTGACCCAAATTTTAACTTCAATTAGCAAATCAACCAAGGAAAATATTCAGCATACACCCATTTTCTTTGCACCTTATTTTTTTTTCTTATGGTACT
CTTCCTTTTGTATCACATAACTTATTATCGTATCTATCACTTTCTTTCTCTTCCTTTTCTTTTTTTCTTTCTATCACTATAACTAAAACCACTCCATTTT
GGAGTGGACATTTTATCATTCCTCATCAACCAATTCCCCTTGTGTTGGAGGGTCATGTTTTCTGTTGCACTTTCTGCTCTCATATCCAATTTGATCCATG
GAAACATTAACAAGTTCATTTGTATAGACATGCACTTTTGAGGTCCCAAGTAACACAATTTGTCACTTTCTGGGAGCAAATTGCATATTTTAACATCTTT
TTCTCTCGAGTTTTAAAAAAATGGGGCATTGAATTTTTGAATCAAATTTGCAGCTTGGTATGGCAAGTGCACTAGAGACTTTATGTGGGCAAGCATTTGG
TGCTGGACAAGTAGAAATGATAGGGGTCTACATGCAACGTTCTTGGTTAATCTTATTGGGTGCATGCATCTGCCTCACACCAATTTACATTTATGCTGAG
CCAATCTTGCTACTCCTAGGACAAGAACCCGAAATTGCAGAATTAGCTGGGGCATTTACCATTCAATCCATCCCTCAGATGTTTTCTCTAGCCATCAATT
TCCCCACTCAAAAGTTCTTGCAGGCACAAACCAAAGTGGGGTTTCTGGCATGGCTTGGTTTTGGAGCATTTATTTTTCATGTTATACTTCTATGGATTTT
ACTTAAAGTGTTTTCATTGGGTACAACTGGTGCTGCTGTAGCCTATTGCACAACAGCTTGGATTATTGCTTTGGCTCAAACGGCTTATGTGATTGGTTGG
TGCAAGGATGGGTGGAGAGGCTTCTCATGGTTAGCATTCAAAGATCTTTGGGCCTTTGTGAAATTGTCTGTTGCTTCAGCAGTCATGCTTTGCCTAGAGA
TTTGGTATTTCATGATCTTGATTGTGCTCACTGGACACCTTGACAATGCAGTTATTGCTGTTGGTTCTCTTTCAATATGGTATGATATCAAATCCAAACT
ACTACACTTCCCCCACCTTGTTGAATTGAAACAAATCTAGGACTTAACAAGTTCTTTTCGCTTCGTTTTCTGTTTTCAAAGATTTCTATTGAAAATAATG
AAAACAGGAGATGAAAAAATAGAAAACAATAAATATTATACATAAACTAATTTTAGACTTGCAGCAGAAGCTAATTCACTTTACGTTTTTTATTTTATTT
TTCTCCTATAAGTACTTATTGATAAGTTTATCCAAATAGGACTCCAGGAGGACAACGTGGAACAATATTAATTATGTTATTTTAACGTTGTTCTCTTAAA
TAGTCTTGGTTACTGATTTGATACTTGTTCTGTTTCTCCTCTTTCAGCATGACTATCAATGGATTTGAAGGCATGTTATTTATAGGGATCAATGCAGCAA
TTAGGTAAATTAATTTATGGCTGATTCTTTTCCCCACCTTAGTGTAAAGGTTATTTTTCAACTTCTAGGAATGATGTATTTTTTTATTTATTTTTTTCAT
TTTCTCAGTGTGAGGGTTTCAAATGAGCTTGGATCAGGACGCCCAAGAGCAGCAAAATATTCAGTCATTGTCACAATTATTGAGTCCCTCATCATTGGGT
TAATTAGTGCAGCCATTATTTTAGCTACAAAAGATCATTTTGCCATCATTTTCACTGAGAGTAAAGAGATGATAAAAGCAGTTTCTAAATTAGCAGGTCT
TCTTGGCATAACCATGATTCTGAATAGTGTTCAGCCAGTTATATCAGGTACTAGGATCTTAATAACTTCTATGATGTAAGTTAGACTTGAGATTTCATAG
ACTTCTTCTACTTTTTCAACGTTTAAAGCAACCTCCCTTGTAAGAACACAGTAAAAAAAATTTAAAATAATTAAAGGGATTAGCGTAATGTATAAGGGAG
ATCCGTGTAATGTTTTTCAAACATCTAAAGGAATTAATGTAAGTGTAGAAAAAATAGAAAAATTTTGTACAATCTCACAAAACTTCAGGAATATCAATAT
AATTTACTTTATAAATTATTACTATGTCATAAGCAAAATTATGAAATGTTTCTATATTCTGGCAGGTGTTGCTGTAGGAGGAGGGTGGCAAGCTTTGGTA
GCTTACATCAATCTATTTTGCTATTACATCATGGGGCTCCCTCTTGGCTTCCTTTTAGGTTACAAGTTGGGTTACAGAGTGGAGGTAAAGTATCAATTAA
AACTTGAAATTAGAATCACTGACCCATTTGGTCCAATCAAATTAATTTTTTTTCTTAACTTTCGACATTACGAAAAAGTAATTACTAAAATATGTATTAA
CACTCTATAATAAGCTTGTTGATATTTATAATAATCTTAAAAACTATATATCTTCTGATTAGTTGAGGCTATAAAAACTTTTACACTAGCAGTACATATT
TATTAAACTAATCTAACTTGATACTTTACCAGGACAATCATCATTCAATTTAGTTTTTGGCTTAAATGTTTATTAATTAGTTAATTCGATTCTTACTATT
AGGGGCCAAATTAGGTAATGGTTGTCAACTTAAGGAACTAAATTGAATGATTTAGTAATATTAGAGACTAAATTGACTATGGAAACTGTTGTTTCAGGGT
ATTTGGGTTGGAATGATCTGTGGGACAATGTTGCAAACGCTCATCTTGTTGTACATTGTCTATAAAACAAACTGGAACAAGGAGGTGAGAATCAGTGCTT
TTCTCAATCATGAAATTAATATTTCATCAACTAAAATAATTGACTAAAAAATGAACAAATACAATTATATATTGGGCCTTCTTCTTTTCATTTCAAATTA
ATCTCAAATTTCAGGTTGAGCAAGCATCAGAACGAATGCGGAAATGGACCGGACAAGAAATTGAAATTAATTTGTAAAATTAACAAAACTCAAACCCTTT
CATAATTTGTGTATATTTGTTATTTGACAAAGATGTGCATGCCTTAGCTATAGCAGAAGTCTCTTATGTTTTTGTTTGTTTTATTAATGAAGGGCAAATT
CTCAGCAAACATTCACTCGGTGATCAATATGCTCAAGAAATTTAATCATCTGCAAATTTTGTTAAGACACTTTTGGTGGACCAGCTTCCTTTTCTTATGT
GATAATTAGTACAGTTTGTATTTGTAATTATATTCATACTATATTCCTTTTCGTTTGTACACGTGTTCTTCTAGGTTCTTTGAATACACTTTGTAAGG

>Glyma.10G232200 | Chr10:46170280..46174862 forward
ATTCATAGCCATAGCCCATAGGTCACCGTCACAGTATCAGAATCTCATTTTCATTTCAAAACCAAGTTAAAATAAAAATAAAATACGTGTACATTCTACG
TTCGTTGTATGAATAATAATAATAATAAATACTGAAGAAAAGAAAATTGCACTGGTGGAGTGGTGGATGTGCATATGCAGTTGCAGGATATAAGCCCGTG
AGAGTTTAACGTCGAATCAGAGGCGTAGACGTGTAGGGTGACTGAGAGATGGACGCTCCCCTGCTGCTGGCCAAAGGCGAGGGGGCGGCGCTGGTGACGG
AGAACGGCGACTACGTGGCGGTGAGGGAGCTGAAGGAGGTGAAGAAGGTGTTCTGGATTGAGACGAAGAGAGTGTGGCAGATTGCGATGCCCATTGTTTT
CAACATATGGTGTCAGTTCGGTGTGAATTCCGTGACCAGCATGTTCGTTGGACACCTCGGCGACATTCAGCTCTCTGCTATCTCCCTCATTAACTCCGTC
ATTGGCACTTTTGCCTTTGGTTTCATGGTAAAATCTCCTTCTCTATCCCCAACGACCAAAACTTTTCAATCATTCTTATATATATATATATATATATATA
TATATATATATATATTAATTTGTTGCTACAGTGATGTTCGTGAGTAACTAACACTCGTTGCCAAAGATTCTTGTTAATTTTCTCTGTTTTTGTCTGTGGA
GTTAGGCAAATGTTTCCTATTATTTTACGGACAAAACTTCGATGCAGTTTTTTTTTAGATGCGGTTAGAATTAGAATTGAAGTATACAGGACAAAAAAAG
TAAGGACAAAACTTAGTTTTAGTTTAGTTTAGTTTTTTTAGATGTTGTTCTGATTAGAATTGAAGTTTTATTTTGCTAATTTACATAAAAAAAGTGTTTA
CTTTAAAGGTAAGTGTAGGATCTCAAGATGAAACTTCAATTCTGATCAGAGAACAACATGAAAAATTTCTTAAGTTTCGTCCTTATGTTAATCGAATGGA
TTTTAATTGGTTCTAGCTAAATACAGAAGGCGGCTCCTTTGTTCTCACCGTTCCTAATTGAATTTTCCTTCGTCTTACAAGGTTTTCGTAATTTTAATCA
TTTTCATTTTCTTAAAATCAGAATTCATAAGCAAGAACAAAGCCTTGTGCTGTGTATTTCTCAGAGGATATTGCAGGAAAACTTGCTTAGAGAATAGAAA
CCAACTGGTTTCACCAGTATAAAGTGAAGGGAGTAGTACACTTTGAAGATATACAATAATAACTATTAATTAAAAAATGGCCAATTAAGAATTAAATTAA
TTATAGAATTTGTTATACATGTATTTGATTTTATTACCATTTTAAAATGTTATATATGTATTTAGTTTGAGAGTGTATTTGCATGTCTTCAGTTTAAAGA
ATACAAATTCAAAAGGGAAGTTGTAGAAATTGTACTTGTTAGTCGTCTGTTGTTTTTTTAAAGAAAGAAAATCTAACGATACTCTATAAAACCTGTAGCT
TGGGATGGGAAGTGCAACTGAGACGCTTTGTGGACAAGCTTTCGGAGCTGGGCAGGTTAATATGCTTGGTGTTTATATGCAACGCTCATGGGTGATATTA
TCCGTGACCAGTATTTTGCTCTTGCCAATATACATTTTTGCTGGTCCAATTTTGAAGTTTCTTGGTCAACAGGAAGATATAGCTGATCTTGCAGGGAGTT
TCTCTATTTTAGTAATTCCACAATTTCTTTCACTTCCCTTCAATTTTCCAACGCAAAAGTTCCTTCAAGCTCAGAGCAAGGTTAACATTATTGCATGGAT
TGGGTTGGTGGCTTTAATTCTGCACATTGGGATGCTCTGGCTCTTAATTTATGTGCTAGATTTTGGCTTAGCTGGTGCAGCTTTGGCGTTTGATATCACA
AGCTGGGGGATCACAGTGGCTCAACTTGTTTATGTTGTGATCTGGTGTAAGGATGGATGGACTGGATTGTCATGGCTGGCTTTTAAGGATATTTGGGCCT
TTGTTAGGCTCTCTCTTGCATCAGCTGTAATGCTTTGCCTTGAAGTTTGGTATATGATGAGCGTTATAGTTCTTGCTGGCAACCTTGATAATGCATTGGT
TGCTGTTGATTCCCTCTCTATATGGTAAGATCATACTTTCATCCCCACACAGGCAAAAAAAGCCATGTAGTCCCAGCCAATTGGAGTGGCATATGTATGC
TGCACTTTGCATAGATATAAATGGAAGGATATGGATTAAATGGAATAAGAAATCTTAAATAAATTCTCTCTGCTAGTTCTTTGCAACTAAGAGCTTGAGA
TTGTCAAGGGAACATGTCAAGTGCATATCAAGACCAAAAAAAAAAAAAAACTATCATGCAATCAATATCAAGTTAAATAACTAGTGAGTTAATTTGCTTC
CAAGTGTCTCAGATTTGATACTGCCATTTTCCTAAAACTATTTTGTGAAAAGTGAGTGAGTCAGAGTAGGGTCAAAATTAAAATTAAGGAATATAATAGA
GGGAATAGAACATTATGGCGGAAGTTCATCCATGTAGCCGACCCCACCTAGTGGGATAAGGCGTTGTTGTTGTTGTTGTATAATAGAGGGAATAGACTCA
GTTACAATATCTCTTCAGTGATTTTGTTGGATGCATTGCATATACTTTGAATTCATTTAACCTCGTAACTCATTCTGCCATCTAATTAGCAGTCCGTAGT
TTTACATTTGCATCTAATTATTCGAATAATGGATGTATGGTAACCTTTTTGCTAATACATTCAATGATGTTTAATAGCATGAATATCAATGGGTGGGAAG
CCATGCTCTTCATTGGAGTAAATGCAGCTGTCAGGTATGCAACCAAAATTACTGTCTTGCTGTTATTTATCCCATTGTCTGTCTACTCTGGCAAATATGT
TGACAATTTCACATTGTTAGTATTGGTTGCTTGCAATGCAGGCTTTTCTCTTAGCCACGTAATGTTTGTGTTTTTCTCATTTTCAGTGTCAGAGTTTCCA
ATGAACTTGGGCTTGGACATCCAAGAGCTGCCAAGTACTCTGTCTATGTGACAGTCTTTCAGTCGCTTTTCTTGGGAATCTTTTTCATGGCTATTATTTT
GGCGACTAGAGATTATTATGCCATCATTTTTACAAACAGTGAGGTTTTGCATAAGGCTGTTGCGAAACTAGGATACCTCCTTGCTGTGACAATGGTTCTA
AACAGTGTTCAACCAGTGGTTTCAGGTATGAATTTATCACCATTACCCTTGTCTACTTTATTATGGATAACAGATCATCCCCTTTATCTTATCCTATTTA
CAATTTTGAAATCATTTTTGCAGGTGTTGCTATTGGAGGTGGGTGGCAAGCCCTGGTGGCCTACATCAACATAGGTTGTTATTACTTATTTGGGCTTCCA
CTGGGGTTCCTTCTTGGCTATGAAGCAAATTTGGGGGTTGAGGTAATATACCACTACTATTTGTATTTCCATTGAAGTCTCCTTTGATAAAATTCATTAT
TTTAGTGAAATGTGTTGAGTTTTCTCTATTTAGATCTGCAAAAATTAGAGAAGGAAAATAACAAAATGGCTTGTTGCTGAACTATTGTTTGCTTTTGAGT
GAAAATGTATATGTCTATGCTGATACCTGCTTAAATTTCTGTTTTTGATAATAGACTTTATTCAAGACTCGTTAACTCTTTTATAGATAATAAATGAATT
TGCTAAAAGAAGTAGAGAGAGAGGAAAAGAAAAAGACAACTCTCCCTAATGATTCAGATTTTTTCCCCATTTAGGGACTTTGGGGTGGTATGATATGCGG
AATTGTTATCCAGACGTTGCTGCTCTTGTTGATACTTTACAAAACCAATTGGAAGAAAGAGGTAGGCAATGGTTCTCATTTTCTCTGAAACTTTTATAGG
CATTTTACTTCTAGTACTACAGAAAAGTGATACTTTGCTTCTACAATAACTTTTTGCAATATTAGAGGGGGCAACTATTTAAATTTTTAGTGCAGCTGAT
ACTAATTTAATTTCAATCAAGAAAACCAAAAGCAAATTGCCACACGGTGGAGGGTATTTCCTGCTTTACATTTTATATTGATAAGTTTAGAAATACAAAG
CTCCATAGTTAACTATTCTTTCTTATCCTTAGTCTTTCCCCTGTTTTATAATTTGCTTGAATGAATTCAGATACTGTAGTTGCTTTTAGTCACTTAACTC
TTCTCAATTGTAGGTGGAACAAACAACTGAACGCATGCGGATATGGGGAGGACAAGACATTGGAGTTGATAAGATAGTGGCTTCTACATAATCCTGTATA
TCTTCCACCTCCACTCGAGTCAAATTCTCAAAGTCTTTTGTTTTTTGTTTTTTTTTTTATGTGTTCCTTCTCTGTCCATTTTACACGTAATGTTGCTCTA
TTCCAATTAGGTACAGAACATAGTTTAGCCGTGGTGTCTGATTAAGATAAAAAAACTTGGTTATTTTTCCAATTTAATTTTCATTTTTTAGTTATTGAGC
ATCTCAGTGAAAAATGAAAAACATCGATATTTTCTTTTACTCTGACATAGAGATGAGCTAATATATCTCTATGCAGGACTAGA

>Glyma.20G162000 | Chr20:39966692..39971061 reverse
TAAGCTGTCCATTTAATCTAACGTATATATTGATCCTTTCACACGGTGCGTAAAAAGTTTACGTACGCACCCTAGTCTTAGCCATTCTCATGATGGTTAG
AGTATCATATTAAAATACGTGTACATTTTTCGTACGTTGTATTGTGAAAAAAAAGAAAAAAGAAAAATTGCACTGGTGGTGGTGGTGGATGCATGCTTAC
CTAATTTGTGCATGCTGTTACACGATATAAATGAGTCTGTGAGAGTGAGAGTGATTCACTGTTTTGTGTATGCAACTAACGATTTCTTTTGTGCTGAGTT
TGACGGTGAATCAGAGGCAGGCGTACGTATACGTGTAGTAGGGTGATTGAGAGATGGACGCTCCCCTGCTGCTGGTGAACGGCGAGGGGGCGGCGCTGGT
GGCGGAGAATGGTGACTACGTGGCGGTGAGGGAGCTGAAGGAGGTGAAGAAGGTGTTTTGGATTGAGACCAAGAGAGTGTGGGAGATTGCGATGCCCATC
GTTTTCAACATATGGTGTCAGTTCGGTGTCAACTCCGTCACCAGCATGTTCGTTGGACACCTCGGCGACATTCAGCTCTCTGCTATCTCTCTCATTAACT
CCGTCATTGGCACTTTTGCCTTTGGTTTCATGGTTAGTGCCTTTGTATTGTTTTTTAGATGTTGTTCTGATTAGAATTTAAGTTTTATTTTGATAATTAC
ATAATAAGTGTTTACATTAAAGGCCAGCTTAGGATCTCAAGATGAAAATTCAATTCTGTTCTGAGAACAGCTTGAAAAATTTCCATATAAGTTTCTTCCT
TATTTTAATCTTATGGACTTTACTTGGTTCTAGCTAAATACTGAAGGGGGTTCCTTTGTTCTCACCGTCCCTAATTGAATTTTCCTTCATCTTACTTGGT
TTTTAGAATTTTAATGATTTTAATTTTCTTAAAATCATAATTCATAAGCAAGATCAAGAGGATCTAGCTCGGTTGATTGAATAATGTACTTGAGTTGTTG
TGAACTCCCTAGTACCTAACTGAGTTCAATTTCTACGGATAAAAAGAAAAAAAATCATAAGCAAGAACAAAGCCTTGTGCTGTGTATTTCTCAGAGGATA
TTGCAGGAAAGCTTGCTTAGAGAATAGAAACCAACTGGTTTCACTAGTATAAAGTGAAGGGAGTACACTTTAATTATATATGATATATAACTACTAATTA
AAAAATGCCCAATTAATGTATTTGATTTCATTACCATTTTAAAATGTTATATACGTATGTAGTTTGTGAGTGTATTTACATGTCTTCATCTTAAAGAAGA
CAAATTCAAAAGGGAAGCTGTAGAAATTGTAATTGTTAGTTGTCTGTTTTTTTTTTTAAAGAAACACAAAATCTAATGATACTCTATAAAACCTGTAGCT
TGGGATGGGGAGTGCAACTGAGACACTCTGTGGACAAGCTTTCGGAGCTGGGCAGGTTAATATGCTTGGTGTTTATATGCAACGCTCATGGGTGATATTA
TCGGTGACCAGTATCTTGCTCTTGCCTATATACATTTTTGCCGCTCCAATTTTGAAGCTTCTTGGTCAACAGGAAGATATAGCTGATCTTGCAGGGAGTT
TCTCCATTTTAGTAATTCCACAATTTCTTTCACTTCCCTTCAATTTTCCAACGCAAAAGTTCCTTCAAGCTCAGAGCAAGGTTAAGGTTATTGCATGGAT
TGGGTTGGTGGCTTTAATTCTGCACATTGGAATGCTCTGGTTCTTAATTTATGTGTTAGATTTTGGCTTAGCTGGTGCAGCTTTGGCATTTGATATCACG
AGTTGGGGGATCACAGTGGCTCAACTTGTTTATGTTGTGATCTGGTGTAAGGATGGATGGAATGGATTGTCATGGCTGGCTTTTAAGGATATTTGGGCCT
TTGTTAGGCTCTCTCTTGCATCAGCTGTAATGCTTTGCCTTGAAGTTTGGTATATGATGAGCGTTATAGTTCTTGCTGGCCACCTTGATAATGCAGTGAT
TGCTGTTGATTCCCTCTCTATATGGTAAGATCATCTTTCATCCCACACACTAGCAAAAAAAGCCACGCAGTCCCAGCCAATCGGATTTCTGTAGATATAC
ATGGAAGGATATGGTTTAAATGAAATATGAAATCTTAAATAAATTCTCTTCGGTTATTCTTTGTAACTAAGTTTGAGATTATCAAGGGAACATGTCAAGT
ATGTAATCAAGAAAAAAAAACTATCATGCAATCAATATCAAGTTAAATAACTAGTGAGTTAATTTACTTCCAGATGTCACAGATTTGCCACTGTCATTTT
CCTAAAACTATTTTGTGATAGAGGGAATAGACTCAGTTACAATATCTCTTTAGTGATTTTGTTGGATGCATTGCATATACTTTGTTCATTTAACCTTGAA
CTCATTCCTCCATGGCTCCATCTGATCAGCAGTCCGTAGTTTTACATTTGTATTTAATTATTCAAATAAGGGATGTATGGTAACCTTTTTGCTAATACAT
TCAATGATGTTTTATAGCATGAATATCAATGGGTGGGAAGCCATGCTCTTCATTGGAGTAAATGCAGCTGTCAGGTATGCTACCGAAATTACTGTCTTGC
TGTTGTTTATCCCATTGTCTGTCTACTCTGGTAAATATGTTGACAATTTCACATTGTTGGTATTGGTTGCTTGCAATGCAGTTTTTTCTCTTAGCCACGT
AATGTTTGCATTTTTCTTATTTTCAGTGTCAGAGTTTCCAATGAACTTGGGCTTGGACATCCAAGAGCTGCCAAGTACTCTGTCTATGTGATAGTCTTTC
AGTCACTTTTCTTGGGAATCTTTTTCATGGCTATTATTTTGGCGACTAGAGATTATTATGCCATTATTTTTACAAACAGTGAGGTTTTGCATAAGGCCGT
TGCGAAACTAGGATACCTCCTTTCTGTGACAATGGTTCTAAACAGTGTTCAACCAGTGGTTTCAGGTATGAATTTATTACCCTTTCCCTTGTCTACTTTA
TGAAGGATAACAGATCATCCCTTTTATTCATCCTATTGACAATTTTGAAATCATTCTTACAGGTGTTGCTATTGGAGGTGGGTGGCAAGCCCTGGTGGCC
TACATCAACATAGGTTGTTATTACTTATTTGGGCTTCCACTGGGGTTCGTTCTTGGTTATACAGCAAATTTGGGGGTTGAGGTAATATACCACTACTATT
TGTATTTTCTTTAAAGTCTCCTTTGATAAAATTTATTTAGTGAAATCTGTTGAGCTTTCTCTATTTAGATCTGCAAAAATTAGAGAAGGAAAATAACAAA
ATTGCTTGTTGCAGTACTATTGTTTGCTGTTGATTGAAAAATGTATATGTCTATGCTGATGATACCTGCTTAAATTTCTGTTTTTGATAATTGACTTTAT
CCCTTTATCCAAGTCTCATTAACTGTTTCATAGATAATAAATGGAATTGCTAAAAGAAAGGAGGGAGAGAGAGAGAGAGAGAAACATAACTCTCCCTAAT
GATTCTGAATTTTTTCCCCATTTAGGGACTTTGGGGTGGTATGATATGTGGAATTGTTCTCCAGACCTTGCTGCTCTTGTTGATACTTTACAAAACCAAT
TGGAAGAAAGAGGTAGGTTCTCATTCTCTCTGAAACTTTTTTTAGGCATTTTATTTCTAGTACCACAGAAAAGTGATAATTTGCATTCACAATAACTTTT
CGCAATATTAGAGGGGACTATTTAAATTTTTAGTACAGCTGATACTAATTTTATTGCAAGCAAGAAAACCAAAAGCTAATTGCCAATGCTTTACATTTTC
TATTGATAAGTTTAGAAATACAAAGCTCCATAGTTACCTATTCTTTCTTATCTTCTTTCCCCTGTTTTATAATTTGCTTGAATGAATTCAGATATTGTTG
TTGCTTTTAGTCATTTAACTCTTCTCAATTGTAGGTGGAACAAACAGCTGAACGCATGCGGATATGGAGTGGACAAGACATTGGAGTTGATAAGATAGTG
GCTTCTGCATAATCTTGTATATCTTCCACCTCCACTCGAGTCAAGTTCTCAAAGTCTTTTTTTCATGTGTTCCTAGTCTGTCCATTTTACATGTAATGTT
GCTCTCTTCCAATTAGGTACAGAACATAGTTTAGCTGTGGTGTCTGATGAGATGGTGAAGATAAAAAAACTTCGTTATTTTTCCAATTTAGTTTTCATTT
TTTAGTTATTGAATATCTCAGTGAAAAATGAAAAACATCAATCTTTTCTTTTACTTTGGCCTAGAGATGAGTTGATATATCTCTATGCAGGACTAGATTA
GTTAATAAAAATTATTTATAGCATATAATAGTGTCATACGAATTCTGCTATGCTAACTGTTTTGAATCTG

>Glyma.18G143700 | Chr18:23063131..23078420 reverse
CAAAGGCTAAAACTATCTTTTAAATGACATGAACAATACTTTTTCTTACATGAGAACCGGACAATTGCGAATAAAATATTTTATAGCAACTATGGCGTTT
CAACTATAACCCTAAACTCTGGAAAAAAAGTTATAACCCTAAACTTTAAAATAATTTTATTTTGTCACTATGTTTTTTTATCTACTTTCCGTGACATTAT
GATTGATTTTTCTCCCTATCCTTTTTACTTTTATCTCCTTTTCCGAACCGATGACGGAGTGTGGCATTACATTGGGTAACAGTAAAAACCTGCTCTGAAG
GCCGGAACTTTTTCTTTATAAAGTTGTTCTAGAATTTTCAATTTTTTCGTATCTCTGATTCATATCACGAATTCACGATGGAGGACCATGAGGACCATAC
TCACCCACTTCTCACGCCGCGACACCACACACCGGACCCCGCCGTCGTCTTCACGGCGAAATCCGATGACATCGCTCCGATCGCCGGCGCCGGCGACTTC
GCCAGAGAGTTCTTCGCCGAGTCGAAGAAGCTCTGGTACCTCGCCGGCCCCGCCATCTTCACCTCCGTCTGCCAGTACTCCCTCGGCGCCGTCACCCAAG
TCTTCTCCGGCCACGTCAGCACCCTCGCCCTCGCCGCCATCTCCATTGAAAACTCCGTCATCGCCGGCTTCTGCCTTGGCATCACTGTACGTAATCTCAC
CGGGTTATACACATATACTCATTTGGTCTCTTCACACGTACAATTTCTAAGTTTTAATTCTACACATACACACTTTTTTAACTTTTATATGTACTTATGC
GTACACTTTTTCATTTAGTCTTTAATTTTGAATGACAGATATTAAAAACAAAATAAAATGTATATAAAATAAGTAATTTCTAAGTATAAAATTGTATTAT
AGAGGTCAAACGAATATATTATGACTGTAATTCATGACAACTGCAATTGCGTCTGTATTTAAAATCCTATACTTTACATACCCATATAATTGATTCTTGA
ATTTTCAAAACTACCTAAAGTGTTTTAATTTGAACCATTGCAATTTGACTCCCAATGTCAAATGTCAGCGACAAAACCACAACCATGATTCAACTGAAAT
TTAGAATTATGATAATATATCTAAAGTTTAATTATTTATTTAGGGTTTAGTTTATAAACTTATCTATTATCTATTTTAGTTATTATAATTAATAAGTGAA
TTTTTAGTGATGAATAGAAGAATTCTCAAAAACATTTATACCTTATATCTAAAATAGACCCTTAACACCTGATTAAATTAAACTACTTATTACTCACTTG
TGGACTTTGCGCGCAGTTTGGAATGGGAAGCGCGTTGGAAACGCTATGTGGACAAGCGTACGGAGCGGGGCAAGTGCACATGCTGGGCGTGTACATGCAG
CGCTCGTGGGTGATCCTGAACGCCACCGCGATCCTGCTGACGCTCCTCTACATCTTCGCGGCGCCGCTGCTGCGGGCGATAGGGCAGACGGAGGCGATTT
CGGCGGCGGCGGGGGATTTCGCGGTGTGGATGATCCCGCAGCTGTTCGCGTACGCGGTGAACTACCCGGCGCAGAAGTTCCTGCAGGCGCAGAGCAGGAT
CATGGTGATGGCGTGGATTGCGGCGGCGGCGCTGGTGCTGCACACCGTGTTCAGCTGGCTGCTCATGCTGAAGCTCCGGTGGGGGCTCGTCGGCGCTGCC
GTGGTGCTGAACGCGTCGTGGTGGTTCATCGACCTTGCGCAGCTCGTTTATATAATGGGTGGGGCCTGCGGGGAGGCCTGGTCTGGCTTTACCTTCAAAG
CCTTTCACAATCTTTGGGGCTTTGTTCGTCTCTCTCTTGCCTCTGCTGTCATGCTCTGGTTAATTATCTTCTTCTTTTCTTTTTCTTTCCTTTTCTTATT
TAAATTATTTTAAATTCTCTTATAGTATTTTAATATTACTTTTTATGAATTAAATTAAAATCATTTTAAATAGTGTTATAATAATTTTTTATTTACTCGC
CGGAGGCTTGTTTTAAAATATTATATTATATATCCAGAAATCATATTTTCAAAAAATAATTATGCCTAAAAATATTCTTTTTACAAAGATGTTTAGTTTA
CATTTAATATTTTCTTAAAAAGTATATATAATTAAAAATTCAGAAAATAAAGAAATAAGTGTTAAATAATAAATTGTAAAGTGGTATTATTCTAAAAAGT
ACGTGTTATAAGGTTGGGTTAGAACGGGTATAATCAAAATTTTACAAGTTTGATTGGATTGGATTTGTATAATTTTTTATAAAAATTGAGTCCAATCTAA
CCAAAACATAAATGGGTTAATTTAATTGGGTCTAAATATTTAAAAAGATATTTTTTATTTGACAAAAATAAAATATTTAGAACTATAATTTGTTTTTTAT
AAAATTTAGTAAGAATATAATGAATAAATGTATTCAAAAGAAATCAAATTATAACAATATTTAACTAATAGAATTAACAATTTTAATGTTAATACAAGAT
TTTTACTTTAAATATAAATAAGATATTCTGTTAACATAAGAAAATATAAACAAAATCAAAACAGAAATAAAAATAATAATTTAAGAAAATCATAAGTAAT
TTTATTTAATAAACTATATATATGAACTAAAAATTAATGTATTTTATATTTAATAATTAATTTATATACAGTAATAATTTAATTATATGATATGAATTCT
GTCGGATTCAAAAAATAAAACATGTTATACAGTTTATTTATGATGCAAACATTTAAAACATCTAATCCAATACAATGGAATGGGATCATATTGAATCACG
AGTAGATCTGAACTACTGGGTATCCTGCTAAAATGTAACTTCAATCATAATGAGAATATAAGATATATCCACGTGTCATTATGGAAATTAAAAACAAGTT
AAATATACAGATTTTATATTTATAAGAAACAATAAACAATTTTTGGAAATATTTTTTCAAAACTTGAATTAAATATAAATATGTGATATTGTCATGTTCG
GCTTTTCTTGAATTTTTTTATAATACTTTGAAACAAATGTTTCTTGAAAAATTAATAATATATGATTCATTTTATATTTTCAGTGTAATATATGTAAATT
TTTTATAATTTTTATTTTCTTAAATAATATTTTAAGAACAAATATTAATAATATTCGCATAAGAAAATTAATAGAATTAAAGATGTAATATAAATAGTAC
TTCCTTCCTCTCAAATAAGTGTCTTCTTTTAAGTTATTTTAATATTGATTTTAAACTTATCTTTTGAGATGGTGAACTTTTAGAAGAATTTATGAAAACA
ATTTATAATTTATATAAAAATAAGTTTAGTTTATTTTTTAATTGTAAAATAAGTTATACATAAACATTTATTACTGATATGATAAATACATATTTAATAA
GTGTTTAATTTAGTAAAGATTTTTATTTTTAATAACTTAAATATTGATTTAATAAAATATTTTTCTATTTGGTTGAAAAATCATGTGCAGCCTTGAAGTG
TGGTATTTTATGGCCCTCATCCTCTTCGCTGGATATTTAAAGAATGCAGAGGTTTCGGTCGATGCCTTGTCCATCTGGTGAGTAATTCATATATTATATA
ATACTGAGTAGTACTGAATACTGATCTTAGTGAATGATGACTGGCGACTGCCTGTTAACATCAATGGTCGGTTTGGTTGGATAAAGAAATACTAAAAAGG
AAAAAATTAATAAGGAAAAATTGAAAAAATAAAACAAAAAATGGATTTTTGTATGTTGAAGTTAATTTATTTTTTTTCTACTTTCTCTCTAATTAAATAA
AATATTATTATTCATGTTTTTTCTTCCTCTCTTTATTGTTTCTCCACCCAACAAACTATCTAAGCATATCATAACTGCAATTCAAACCCCCAGTTTCATT
ACCCATTTTATAATGTCTTTCTTCATTTGATCCTTTTTTTATACCTCTATTTTTACATTTATTTTGTAGTTTCTTTATATAGTTTTCTCCTACTTTTTTC
ATTCTTTTTTCCCTTCTTTATTGCCACATTTTAAAGATTTCAGAAAATCATTTATTTTTCATTACAAACTAAAAATCATTCATTTAACTAAAGTTTTATG
AGTAGTAAGGCAGAAGGCAGAAAGCTGGATCGAGCGCACAGAAGCTCTTTAATGACTAGTTGGTGAAGCAATGTTTTAGCTTCGTATCAATATTTATATG
AATACAAATATATCTCTCTATTATTCATTAAACACAACTAATTAATGTATTAAAGTTAATGTTAAATTATTTAGATAATATTTAAATTTAATCTTTAATA
AAAATAATTATTGATTAAATTAGATTAGTCAAAAAAAATTTCTTCCTAATAAATCAGAAGATTAAAAGAAAAAATAGTATTTCACTCCACCTAACACTTT
GACAAAATTAAAAAAATAGTTATACATTTAACATATATATATATATATGTATATATATATATATATATATATTTCAAATTTAAATTGTAGCGCTCAAACC
AACCCAATACTTCTCTCACGAAATGTTCAATTACTTCGGAGATTATTTTTAATTGCCAGAACCTCGTACACTTCGTGGCACAAAGTTGAAAAAGAAATGG
ATAGCATCCTTTTGATTTACTGCTGTGTCTTTTCCAAATAGACAAATTGGAAAACATTATTACTTTGTGTATCAGGAAATATTTTTAATGATTTTTTTCG
CTATAAGATATGTTTTGATATAAACTTTATATTTAAGAATAATTTATATATAGTAATTATATTTGATAATATAAATTTAATTTTATTAAAATTAATTGTT
TTTAACTTTAATTGTTTCTTTTTTATTTTATGTGTAATTCAAAAAATTTCAAAAAAAATCTCTCTCAATGTTTATCACTTGGTGTCCTTCCTCATCCATA
TTCTAATGTCAATTAATCGAGGACTCGTGCTACTCGTGTCTATATAATATTGGAAAGTTGTTATCTACCTATATATTATTATATTAACGGCATGCACTTG
CCATTGTGTTTGTCTGACTATTTACATATAGTACAAAAGTTCCATTTAACTTTAATACCCCCCACCCCCCGTGTTTGTATGACTAGTCCACTTTAGAGTT
CAGACATGATTAGTTGGCACTTGGTACTATGACTTCCCGATTTTAGTGGATATAATTCTTCATAGACAAGTATATAAGATGGATTTTCATCTAAAGCGAG
TCACACGTGTTTATAGGTTTCATATAATTTTGTTTTGAATAAATTTCTATCTATATTTTTCTTTCTTCTGAAAACCGTAAACTTGAATCAACCACGGTGC
TGACGTTCATATATCAATTCTAACTTATATTAGCACTAGCTAAAACAAAGTACAACAAGCTCATATATATAGAATCCGACGACGACTTATAATAAACGCC
TCTACTGGGTGGATTAGAGTGGAAATAATAGAGAAAGATAAAAGATGCAGAAAAGAAATAATAGATTTTTTTATGGACAAATGTTAGATTTTATTAGTAG
AAAGAATTAAATATGTAATCTTTTTATTTTTTTTAACCATCCAACTAGTCTTATATTTCCATAAATAATAGATGTAAATAATGATAACAATGAAAAAAAG
ATGAGACAGGTACTAAGAAAATGATGTAGTCATGTAGAAATGAGAAGTAATTTAGGAAATGAGTCTGTGTTTACAATTTTTCATTTTTTTACAGTTAAAA
TAATGTCCTTTGTTATATTTAAAAATATTAATAATACTCTCCCTATATATATAGTCTTTTATTAAGTCAAATTTGTTTATGTATGTTATAATTTAGTTAG
TTAATTAGGAGTTAGTTAGTGATTTATTTTGAAGTTAGTTTGACAGCTATAAAGTTTGTTATAGTTACATGTGCAATGTGTATAAAAATAATAATGATTA
TCAACGAGAAGTATCAAAGTGCAGAAGTTTGCATAGTGTGAGTGTGTGTGAGAACTTTATTGTTCCAACAGACTGGTATTCAGAGCTGAGGTTCAAGATC
CCTGGATATCGAAATTGAGATGACTTCCTCGAATGGAAATTTTTCAGCATCTATATCTGTTCTCATAGGTAAGAACTATGATGATTGGTGTGCTCAGATG
AAGGTAATCTTTCGATTTCAAGATGTGACAGAACTGGTGCAAGAAGGAGTTCAAGAACCTGAAAAGAACCCAACTGAGGCACAGAAGGTGGCTCGCCGTG
ATTTGATGAAAAGAGATGCAAAGACATTGTTCATTATTCATCAGTGTGTAGTGTAGATGCAGATAATTTCTAGAAAATTAGATCTGCTGATACTGCAAAG
AAAGCATGGGATACTCTAGAGAAATCCTATGCAGGGGATAACAAACTCAAGAAGGTGGAGTTGCAGACCTTGAGAAAGAAGTATAAACTTTTACAAATGA
GTGATCAAGAAAGCATTGGTGAGTTCTTTTCTCGAATCTTGGCAATTACAAATCAAATGAATGCTTATGGTGACAAGCAATCATACTTGGGGATCATTGA
CAAGGTATTAAGAACCTTGACACCAAGATTGGAATACTTGAAGCTTAAGAGATGAGGCTCAATGAAAGAAACTCACAAATATCAGCTGACAAGCTATGCA
AGCCCAAACAACCAAAGGGAACAACTATGATGGTGGCAAGAATAAGAAGGGAAAGGGAAAGTGGAAGAACAATAAGTGGAAGGGGTTAGGTGAGGGCTCC
AGCAGTTCTGGAAATCATAACCAGAATGAAGAAATTGACAAGAAAAGTGGAAGGAATCACAAAGTGGGCAAGAAGAAATTCAACAAGAAAGGGATTCAAT
GCCATAACTGTCAGAAATGGAGACATTTTGCAGATGAATGCAGAAATAAAAGGGTTCCAAGAAATGCAGATGAGGCTCAATTGGCACAAGATGAGGATGC
TGACTCTGATAAAGTGTTACTAATGGCAACTACAAACTCAGAAGAAGACAGTGTTAATTTGTGGTATCTTGACACAGGTTGTTCCAATCACATGACTAGA
CATAAAGAGTGGTTTGTAAACATTGATGATAAGGTGAAGAGCAAGATCAAGTTTGCAGATAACAGTTTTGTAACTGCAGAAGGCATTGGAAAAGTGATGA
TTCAGAGGAAGGATGGACAACACTCATTTATCAATGATGTGCCATATGTTTCCAATATGAAGAATAATTTGCTGAGTTTGGGACAGTTGCTAGAAAAAGG
CTACTTAATGCAGATGGAGGACAGTCAACTGAAGATGTTTGATAGCAATAGGAGGTTGATTTTAAAGGCCCCTTTTTCAAGAAACAGAACATTCAAGATT
GGAATTCAGATTGCAAAATTTCAATGCTTGGTTGCTTCTATAAGTGATGAAAGCTGGATGTGACATCACAGGTTTGGTCATCTAAATTTCAGGAGTTTAA
GTGAATTGAAAAGTAAGAAAATGGTTCATGGTCTCCCTCAAATTGAGATACCAAAACAGTTGTGTGTTGAGTGTTATGTGTCAAAGCAACCAAGGAATTC
TTTCAAGTAAGAAATTCCAATCAGATATAAAAGAAAGCTTGAAGTGATCTATTCTGATGTGTGTGGTCCTTTTGAAGTGAAATCCCTAGGAGGTAACAGT
TACTTTGTGTCATTCATTGATGAATTTTCTAGAAAAATGTGGATCTATCTCATTAAGCAAAAAAATGAAGTGTTTAACATATTTAAGAAGTTTAAGTTGT
TGAGTGAAAAACAGAGAGATAAGGTAATCAAAGTGCTTAGAATAGATGGAGGTGGTGAGTATAACTCACATGAATTTCAAGTATTTTGTGATGAAGAGGG
AATAATTCATGAAGTGACATCTCCCTATACACCTCAGCATAATGGTGTTGCTGAGAGAAGAAATAGAACCATTCTGAACATGGCCAGGAACATGATGAAA
GGGAAGGGAATGCCTCATTACTTCTGAGGAGAGACAACTTCTACTGTTGTGTATATTCTGAACAGATGTCCTACTAAGAGATTGCAGGGATACACACCTG
AAGAAGCATGGTCAGCAAAAAAGCCTAGTGTGAGTCATTTCAGAATATTTGGTTCACTGTGTTTTAAGCATGTGCCTAAGCAGATTAGAAAGAAACTTGA
TGACAAGGCTGAACCAATGATACTTATTGAATATCTTCCAATCGATGCCTACAAGTTGTATGATCCTAGAATGAGGAAGGTTGTGATTAGCAGAGATGTC
TTGATAGATGAAACCAAGGGCTGGAATTTGGAAATAAATGCTGCAGACAATGGGGAAAGAAATGTGTTTGAGTTACAGTCAAATCATTTGGATCAATTCC
AGTTATTTTATCATATAAATCGAGTCTTAAATTAACTCATTTGTAAATGTTTTCAAGAAGATCTTTTAGAGTATAAATTTTATCCTAATTAATTTTTAGT
GTAAACATGCATTTTGTGCACTTTGTTCTACTACAGCATTGAGAGTAAAAGAATTATGATGAAACCTCACAAAGTTCAGATTGGAGAACAGCTATGGAAG
AAGAATTGAGACCAATTGAGAAGAACCAGACTTGGGAGTTAGTCCACTTACCTCAAGGAAAAATACCAATTGATGTGAAGTGGGTCTTAAGACTAAAGTA
AAGCCTAATGGAGATGTATCCAAGTATAAGGCAAGATTAGTAGCAAGGGGATTTCTGCAGAAACATGGCTTGGACTACAATGAGGTTTTCGCTCCAGTTG
CAAGACTTGAAACTGTTAGGCTCATTGTGGCAGCTGCTAGTAACAGAAACTGGTCTCTATATCAACTTGATGTGAAGTCATCATTTTTTAATGGGCCATT
TGAAGAAGTCTACATAACTTAACCACTTGTTTATGTAGTGGCAGGTCAAGAGGATAAAAGTTTACAAGTTTAATAAGGCACTCTATGGCCTCAAGCAGGC
TCCTAGAGCCTGCAATATGAGAATTAATAGCTTCTTAGTCCAACAAAATTTCACCAAGTGCACTACTGAGCATGGAGTTTATGTCAGAAACACATATTTT
GGTAAGTCTTTGATAATATGTCTTTATGTAGATGATTTGCTAGTGACTGACAGTAATAAAGAAGATATGAGAGTGTTCAAAGGAAGAATAATGGAAGAAT
TTGAGATGTCTAATCTTGGTGAACTATCATACTTCCTGGATATTGAATTTGTTTCTACTAGTAAAGGAATTTTCATGCATCAAAAGAAGTATGCAGAAGA
CATTCTGAAGAGGTTCAATATGATAGAGTACAATTCTGTGATCACACCAACTGAAACTGGAATTAAGCTGCAAATAGATGGGGATGAGAAAGAAGTTGAT
CCTACTTTGTACAAGCAAATTGTAGGCTTATTGAGGTACCTATGTAACACCAGACCTGACATTGCCTATTGTGTTGGGTTGATAAGCAGGTTTATGGAGA
AACCAAAGAAACCTCACTTCCTGGCAGCAAAAAGGATTCTGAGGATGATACGAAAAGCACTGCTGGGTATGTTTTCAAATTTGGAACAACACCAATCTCT
TGGTGCTCAAAGAAGCAGAGTGTAGTTGCTTTGTCAACATGTGAAGCAAAATATATTGTTGCTGCTATGGCAGCTTGTCAAGCTCTATGGCTGGAAGCTT
TAATGGAAGAACTAAACATGAGAGATTATATTCCTATGAGGTTGTTGATTGATAACAAATCAACAATTGATTTAGCTAAGCATCTTGTGGCACATGGTAG
GAGTAAACATATCGAAACCAAGTTTCATTTCTTGTGTGATCGTGAGTAAGGAGAAGCTTGAATTGGAGTTTTGTAGGTCTAAAGATCAAGTTGCAGACAT
ACTAACTAAGCCATTGAAGTTTATCAAGTTCAAGAAATTGAGAGACAAGTTAGGAGTGACATCCTTGACAAATCTAAATTAAGGAGGGATGTTGATGTAT
GCTGTAATTCAGTTAGTTAGTGATTCATTTTGAAGTTAGTTTGACAACTATGAAGTTTGTTATAGCTACATGTGCATTGAGTATAAAAATAATAGTGATC
ATCAATGAGAAATATGAGAGTGCAAAAGTTTTTTTTTTTTCAGAATTCTTAGTCTAATCCCCTCTATTTTCTCTCAATCATCTTCTCTTATTCTCTAACA
TAGAGTGACAGTGGGTGCACAGTGTGAGTGCGTGTGAGAACTTTATTTTCCAACAAAATTTATGTGAATCTTATTAAATATGTAAGTCTTATTTCCATTT
TAGTGGAATTTGTACATCAATTTCAACTAATATGAAAGAATGCTTTAGAAATAACATGTGCATATTCGTGCTATAATGATTCTTCTTGTTGGATGGGGCT
CTGTTTAAATGCAATCTATGCATTTTGATTTTTGACTCTGCCTTCAAACTTTGCACAAATGCTATGTTTGGACTGTTTTAATGATATGGCTTTTCATGTC
AACACAAAAAACAAAAACTCCTTATTCCTAAATCCAAAATCCTAATGTTTTCTCTTATCCAGAGTCATATCATAGTCATAAGTATCCTTTTATAAATATA
ATTTTTTCGAGTCAAAAACACTCATTGTGTAATGAGTGTATGGTGGGTCTAGCAAGCTCTTCCAACTAGAATTTTATTTTACACACAATTTATGTTTAAA
GTCTTACTAGTACTTAAGAAGATCGAATCAAACATGTATAATGTATCACATGAACAAACCAATAGATGATGTAAGAATTTCTAATGTAAACATTAGTAAT
GGGCAGGCACCCATATATATTAATACATCATTAATATAAATGAATATAGTACAACAATCTACACGCCAACACTATATAGCACCAAGGAAAAAATATGTCA
GAATTTTTGCCAAGTAGTGCAACCAACCTCTCCTTTTTCACCTTGTTTGGTTTTGATATTAAACAATATTGTGCCTCATCTTGTGGTTTATATTGAATTT
GCAGCATGAACATATTGGGATGGACCATCATGGTATCCTTTGGAATGAACGCAGCCGTAAGGTTTGTTGTTGTAGCACGCACCCTTCATCTGAACATTCT
TCTAGCTACCATTGTATTGTATATACCCAAGTAACTAACATATATTATATATAGTTTTTAAGAGTTCATCTGCTGTGCCTATGCACTATGCAATGTTCGT
GTAAAAGATATTTATACTATTAATTAAAAATTATTATTTACAATGACTTTTAAAATAAGTATTAATTAAAAAGTTAATAAATTTATTATATATAATAATT
TGTAATTAGATAATAATGTAAAACTATTTTATTTTATATTGTTAGTTTATAGTTTGTATTCTCAAATTATTTTGGCTAGCTGTTTATAGTATGAGAAAAT
AGGCTATATGCATTGTATTAAGAAAATAGACTCTATATCACTTTTATCTAATCACCAACAGTTTTACATAATAAATTTATTAATTTTTATAATAATTATT
TTAAAAATTATATTATCAATGATTTATTATTAGTTAACAATAGTATAGACTGTTGTACGTTATTGTGCATAACCATTAAAACTTGAATGATTTACCTAAA
TCAGTCAGTGACCAAAAATTACATTTGCAGTGTGAGGGTGTCAAATGAACTAGGAGCATGTCACCCAAGAACAGCAAAGTTTTCACTTCTTGTTGCTGTG
ATTACTTCAACTCTGATCGGTGTCATGCTCTCGATGGTGCTGATAATCTTCCGGAACCAGTACCCTTTCTTGTTTTCAAATGATTCAGAAGTGAGAAAGA
TTGTGGTAGAGCTCACGCCCATGTTGGCCCTTTGCATTGTCATCAACAACGTGCAACCCGTTCTCTCAGGTGTTGCTGTTGGCGCAGGGTGGCAGGCTGT
GGTTGCTTACGTGAATATTGCTTGTTACTACTTTTTCGGTATTCCTCTGGGTCTTATTTTAGGGTACAAGCTTGACAAGGGGGTCATGGTGAGTCATTTT
CTCTTTATCTTTCTTTCATTCACAAATAATTAATAAGGTTAATTAAATTTTTAATCCCTTAAATTTTTTAGCCTCCAATTTTTAATCTCTTAAATTTTTT
TTATAAATTTTAATCTTTCATTAATTTTTTTATCACTTTACATCTTCTAAATTATTTTATCTATTTTTAATTCCTCATTTATTTTTTATTATTCAAAATT
AATCTTAAATATAAAATAAACGAGGAATTAAAAGTGAGCAAAAGAGAATAATTTTAAATAATAAAAATGAAAAAAATATAGTGATTAAAAATATTAACAA
AAAATTAATAAAGGACTAAAAATTGAAATATGTAAAATTTGAGTAACTAAAAACTTAATTAATATATCCATTTACTATATTTTCATATAAATTTCTTCAC
TATTTTATAATGTAAGTATTTAATAAAAATAAATATGCTTAATATATCGAAAATATATAATACGTTTTATACTTATTATGAAAATGAATCTAATAGTAAG
AACTGCATTTTTTTTTACTTTCCTTTGGTAAAAGGATATAGAATTATTCCTCTTCCATGATTTACCATTTTTTTTCCTTCCTTCCTAGTTCTAGTAAAAT
ATCATTTGGCATTGGGAAAAGTCAAAGGAAATATAAAGTTCTACAAAACTTAGACACGCCCAAAAAGTGCATCTATACATTTGAAACTATAACTAGCAAT
AGAAAATTAACAAGTAACTTTGGCACGCTGCATAATTCACATGATTGGGCTTTAACCTTTTGAAACGGGGTAAAACACCATTTTAGCAAATAATAAAAGT
ATAAATTATTATCATATTAGTATCTGAAATTAAAGAAAATTAAAATAATTTTTTAAAATATTAAATAAATATCTAAAATTAATTATTTATTATCATTCTA
ATCCTTAAATATATGTTAATACTATTATTCAAGTCACACATTTGTTTTATTTTAGAGTAAAGTAATCAACAAATTGTATTTTCAAATTGTTTATATTACT
TTTCTTTCTTTCTCCGGGGACTAATTTTAGTATTGTACACTTTTAGGGAGCAAAGAGTGTTTTACCCTTTTAAAATATTTGCATTAAAGTCAAACAAGAG
AATAAATAAAGGGGATTTAGCCTTATAATGGGGATTGAATTGAGTTTTGTATATTTTTATTTCAATCTATATAATTAAACTTAGGCTCTTGCTATTTATT
TTTCTGTTTTTCTAATTACATTCCCATATTTTCTTTTTATCTCTCAATTATTTATTATACTTTCTTTTTAAAAAAAGAAATACACCTATTTTACTTCCGG
AAATGTATTTCTGGAATTAGATATATCTATTCCAAAAATACATCTCCAAAACTATTATTCATAGTTTTAAAAATATATTTCCAGATATATATTTTTTAAT
AAAATATCACCTAAGAATTAAGATGGTTGACAAGAAATGAAAAAATGATATATGTTTATCTTATATTATGTTTATGTTTGCTAGTCTTATTTTTATTCTA
TCATTAATGTTTTTTAAATCCTATTTAAAATCTTATTTTCGTCTTATCTTTATTATTCTTTAAACCCTAATTTTAAACTATCTTCACCATATATTTATTA
TATAATACTATGTTTTCATCATTATTATATAATATTTTACGTTTTCCTTCATGTGATAATGCATTTCAAGGTACGCTCATTCATCACTAATTGCATGTGA
CATTGAATCGTTGACATAGACTTAGATTAGAAATTGAATGAACACACTTAAAGGAAAGAAATCATCGTCAAATGCTTTGCTTTGTAATTAAGATATATAA
TATTTGAAGTTAGTGATTAACATACCTTGAAACATAATGTCACGTGAAGAAAAACACAAAATATTATATAATAATGATGAAAATGCAATATTATATGATG
AATATATGGTGAAGTTAAAATTTGGGTTTAACGAATAATAAAGATAAGGTGAAAATAAAATTTTAGATAGGATTTAAAGAACACTAATGATAGGATGAAA
ATAAGACTAACAAACACAAGTATAAGATAAACATTTATCATTTTTTTTATTTCTCATCAACCATCTTAATTCTTAAGTGATATTTTATTAGAAAAATATA
TCTATTTTGGAAGTATATATCCAGAATAGATATATGTAATTCCGAAAAAACATTTCCAAAATACATTTCCAGAAAGTAAAAGAAGTATATTTCTTTAAGA
AAGAGGGTATAATGGGTAATTGAGAGGTAAAAGAGGATACAAGAGTATACTTAGGACAATGCAGGTGTAAATAACAAGAGTCTTAAACTCAATTGGATTA
GATTGAGTTTATCTTTTTTTTTTAATTCAATCCAACTTTAATTGGATTGGATTGAATTGGTTATCGAGTTGGATCATTAAAAATTGTTACCTCTTCAAGT
CTTGAAAAAAAATTTAAAAAATTCTCAAATTCAAAAGTAATAGTAAGGATTTTTTTGACAAAATAGTAAAGATATTTTAAAATCAATATTCATAAAATAT
TTTACTATTCATATGGTAAACAGATAAAAAAAACCATAAATATACACTCACAGTACCATCATAAAAAATGTATTACTGAATAATAAAAAATATAATTTCA
AAATAATGTATACCTGAGATAGATGTGCTTAATTTAAAGTTTTTAATTTTTAATGGTATGCGGATTGGGTTGGTTTGGTTGGATCCAAAACTCAAAATCC
ATCACTCAATCCAAATCTAATGAGACTCATTAAACTAAAATCAATTTAATCCAATGACTCAATCCAATCCTTAAAAATCTAGTTGGATTGGGTTGGATTG
AGCAGGTTCACATATTTAATTTGATTCACTTACATCCCTATTTTATGATGATTTTCAGGGGATTTGGTCTGGAATGTTGTTAGGAACTATCTTACAAACT
TGTGTTTTATTCTTCATGGTCTATAGAACAGATTGGAATAAAGAGGTATTTTTTTTTTGCAATGTTATTAAGGTTGCATGTATATACTATAAGTTCATTA
CATGCTAGGAAATATTTTAAGGTTGTATATATACACTATAAGTCTAGTAAATATGTTTAAGGTTATATGTATATACTATAAGTCTACTAAATATATGTTC
ATTACATGCTAATTATATGGGTAAAATGACAGGCATCCCTTGCGGAAGATAGAATAAAGCAGTGGGGTGGGCATGAAGATTCAAAAACGCATGCTATAGC
GAAGAATGATCAAGAAACATGAAAATTTGAAAATTCTAGAAGCAGGGTGTACCTTCTTTTATTGATTTTTTTTCAAGCCATGACTTAGTTGTTTGCTGTT
TCTCTTTCCCATGGATCAGGTGGACGATCCTGCATTCATTTTTTTCCTTAAAAAATAAATGAGTAATGTTATGTTTTTCTTAATAATTCT

>Glyma.14G158100 | Chr14:34998078..34998467 forward
ATGTTGGGAGTGTACATGCAGCGCTCGTGGGTGATTGTGAACGCCACCGCGATCCTGCTGTCGCTCCTCTACATCTTCGCGGGGCCTATGCTTAGAGCTA
TAAGGCAGACAGAGGCGATTTCGGCAGCGGGGGGGGAGTTCGCGGTGTGGATGATCCCGCAGCTGTTCGCGTACGCGGTGAACTACCCGGCGCAGAAGTT
CCTGCAGGCGCAGAGCAGGATCATGGTGATGGCGTGGATCGCGGCGGCGGCACTGGTGCTACACACTTTGTTCAGCTGGTTGCTCATTCTGGAGTTCTGG
TGGGGTCTCGTCAGCGCCGTCGTGGTGCTGAACGCGTCGTGGTGGTTCATCGACATTGGACAGCTGCACACTTTTTCTTCAATCTTATAG

>Glyma.02G038600 | Chr02:3594440..3614434 forward
ATGCCTAGGTCGCGCCGCTCAAACCCAAGGCTCGCTGAGAGAGGTTCGCAATAGAGCTCAGAGAAAGTGTTCGACGAGAGGTTGAGGGTTTTGAGGCTGA
GAGGGAGTTCGTCGGGGATGTTGCCGAAGATGTGGTTCTGCACGACGTTGAGAATCTGAAGGCTCGTGAAGTTCGCCATCTTCGCAAGAAGGTTGCAATA
AAATGAATTCTTCTGCAAGAACAATGATTGCAGGAGCGTGCATTTGGATAGCGACGAAGGAGTTTGAACGATGTTTTAAATACACAGCTCTGTGACTTCT
TGACAAGGTTCTTTGTGATCTTTTCTTCTCGTTTTGCATCTTTCTTTTGGTTTTTTATGTCTATTTCCTTTTTCTCAGTTTTCTCAGCAACCAAAACTGG
AAGGTTAGATTATTTGGTGAATTTGTATATGCATTCATTGAGGTGAGTTTCGTTCTTTAAATCTAGAAAAAGAAACATCGCGCAAGATCTACTCTACTCT
CCTCATATCTTTTTCTTTTCTTTTTCTTTGAAATTAAAAAGTTGTTAGAAGGTTGTGCAAAATAATTTGATTGTTTAAAGGTTCTTTTTTGTGTTTAATT
ATTGTTTCGCAGGTATGAATTGGTTGAAGAGTGTGTTGATGATTACGATGCGAAACCTGGAATCTGTGAGTTGAGGAAGGCAGTTCTAGAAGGTTTTGCA
ATATAATTTGATTGTTTAAAGGTTCTTTTTTGTGTTTAATTATTGTTTCGCAGGTATGAGTTGGTTGAAGAGTGTGTTGATGATTACGATGCGAAACCTG
GAATCTGTGAGTTGAGGAAGGCAGTTCTAGAAGGTTTTGCAATATAATTTGATTGTTTAAAGGTTCTTTTTTGTGTTTAATTATTGTTTCGCAGAACTTG
GAATCTGTGAGTTGAGGAAGGCAGTTCTAGAGGAGCTGAAGATGCACAACTGGCTGGTACAGGTGAGACGATTTTCCATGAGCTAGATCCGGGTCCTGAG
ATCTTACTCCTTAAAAAGATTCTGTAACTTATATTTGTCTATTAGGACTTTAATTATGAAGTATTCCAGGGGAAGAATGTAATTTGCCATGATTTTCAAC
TTGTAAAGGAAATAATATCGCAGGCTTTGTATGCCATTAGTGAAGGAAATAAATATCTTCTATATGCTGATTGGATTTGGATCCCTTGTTTAAAAAACTC
GAGCAATTTGTGTGCACAATAATGAAGTTTTATATGTTAGAGCTTGATCACATGGGACATCCATTCCAATGAAACAGGGGAATGAGTGCTTTTAAATTAT
TTCTGGGAAGGAAAAAAAATGTATGACTTTTGCCTGAACTTCACCTCAGTCAGTCTACTATTATTATGATAGTATGTGTGAGATTAAGCCAATCTTTAAA
CTTCAAATGTATGTCACATATGTATCATATTGTGCTAACTTCATGAGATTTTCAAACAGCCAATTTATACTTCACATGGTTAAATTTATTTTCCTTTTAC
CAGTCATTTAGCCTATATTATTTTGGCTTTTTGCTAATGGCGTGTAGGAAAATCAACTCTTTTAGCAGCAATTCTCAGAGAACCCCATCAAATCCAACCA
CAACTGGGATCCACCGCAGTTTTCATCGCCGGCACCCCTGACATAGCTCCGATCACCAGCACTGGCGACTTCTATAGGGAATTCATGGTGGAGTCCAAGA
AGCTCGGGTACCTCGCCGGCCCCGCCATCTTCTCCTTCGTCTCAAAGTACTCCCTCGGCGCCTTCACTCAAATCTTTGCCGGCCACGTCGGCACCATTGA
CCTCGCCGCCGTCTCCGTCGAAAACTCCCTCATCGCCGGCTTCTCCTACGGCATAATGGTAAAATCCTACTCTAAGTAATCACACCATTGACCGGTCAAA
TAAAAAATACATAAAAAAATCTAATTGATTTGGTTGAAAAGAATGTGCTTGAGTTGATGTAAATTTTTCTTAAAAAAAAATGTGTTTCCGAACAGCTAGG
GATGGGAAGCGCGTTGGAAACGCTGTGTGGACAAGCGGTGGGAGCAGGGAAGCTTGACATGCTGGGAGTGTACATGCAGCGGTCATGGGTGTTACTTCTC
AGCATGGCTTTTGTTCTGTGGCCTATGTACATCTTCGCGGGACAAGTTCTGAAGTTCATAGGGCAAGACACCCAAATTTCGGAGGCTGCGGGAACCTTCG
CCATTTGGATGATTCCGCAGCTGTTTGCGTACGCGCTGAATTTTCCGGTGGCGAAGTTCCTGCAGGCGCAGGTGCTGAGTTGGCTGCTCATGGTGAAGCT
CGAGTTGGGGCTGGTGGGCGCCGCCGTCGTGCTCAATGGCTCGTGGTGGTGGCTCAGTTGGTTTATGTGTTTGGTGGGTGGTGTTGGCCTGCGTGGAACG
GCTTCTCGGGGGAAGCGTTTCGGAATCTCTGTGGATTCTTTCGCCTCTCTCTTGCTTCTGCTGTTATGCTTTGGTGAGTGCTTCCAAAACACTCTCATGT
CACATGTTTTATCATTCTTTTACTTTAAAATATGTAACCTTAATTTTATAATTAAAAATTTAATAAATGTATGATATGATATAGTAGAAAAAAAAATATT
AAAGTGATATATCACTTAATAAGCATACTGAATTTCTAAAAATAAATAATTTAAATTTAATTCTCACAAAATATATTATAGAAAAAGTGAGGAAAAGTGT
AATTAAGTCTAAAGATGATTAGTGACATAATCGGTCTTTACTAATGCTTATTGGAATATTGTTTTTTTATATAAAAAGATATAAAAAATAATAAAGTGTT
AACATTGAGATCGATTATTAAAATAGGTAGGTGTCCATTAATATTCAATAGTATAGTACTATTTTGACTACTAGTTTAGATTGTAGTATGTACGTACACC
ACGTGATGATTTTGACTACTAGTTTAGATTTTGACTACTAGTTTAGATTGTAGTATGTACGTACACCACGTGATGCACTTAAAAAACAAGTGCAGGGAAT
CTGGTCTGATACCTACGCACATATTTTCATTTTATTATAAAAATTAATAAATATAAAAAATAATAAGCGTTAATAATGAGGTGTTTAAAATAGATAATTT
TTCACCATTAGTTACTTCTTTGACGTTACTACTAGATTATATTGTAGAATGTACAGCACTTGATGGCTTTGATGCACTTAAAAAAAAACAAGTGCAGGGA
ATCTTGGTTGGTCTGATACCTGTACTTATATATATATATATATATATATATATATATATATATATATATATATATATATATATATATATATATATATATA
TATATATATATATAGTATTTTATATTTTCTTCTTATTATAAAAATGAACGAAAATAACATTATTTTTATATAAAAAAAGATATGTAAGAGAATAAAAGCA
AAGTGTGTAATAAGAAGAGACAAGGAAACTCATAAACATTTGAGTGTTGATATTTCAGTATAAATATTTCTAAAGAAATCATATAATAGAAAGGTAACAA
AAAAAGAAGTAAGAGTACATATAAGATAAATAATGTGATGGAAAATGATGAAGAATAAAAATAATTACAAAATATTATAAGAATTTGTTATATGCGTATC
ATTATTTTATATTTTTCTCTTAAAAAATAAGAATAGTCTAATGTCTTATTTGAAGATTATAATTTAATTATTATAATAAAGAGACTTGTAATCTTCTGCA
TGTGTGTTATCAATAGATTTGTAATTATTCATGTGTGTAATTTAAATCTTTTATGATACAAATCACATGCAAAACAATTATCAAAAGCTTTTAGTAGGTT
TAATTTATGGTTAATGATGATGTATTATGTGTAATATGACGAACCAATTATACACAAACTAAATTTACTTTATTTTGTGTTGAAAATTTGCTTTTATTTA
AAAGAATGAAAGTACAAGATAGACTGTATGGTATTTGCTTGTACTCTTCATAAGATATTATGTCAACTACATAAATTTAAGTTATTAAATAAGTGTGAGC
AGGTAAATAAAATATCTAACATCAGTGTTATTGAACCAAAAATTAAATAATATAAACATAACTAAAGTACGTGTGATGCAATTTAAAAATAATCAATGTG
AGGTAAGAAATCCTGTTTGACTAGTATTCCTGTTTCATGTTCGATGGATATCCATTTTTTATATCTTAAAAACAAGACTGGAATATTTAATTTTATGTAA
TTAAGGAAGTTATGTATATTTTCTCTCATGCATGATGTGTGCAGCTTGGAAACATGTACTTTATGGCTCTGATTTTGTTTGCTGGATACCTCAAGAATGC
ACAAGTCTTTGTAGACGCCTTCTCTATCTGGTGAGTGTGAGTGAGTAATAACAAGGAGTAATTTTCGGTACTATGACTATACTATATGGTGTGTTTTCTT
AAGCTCTTGAACATTGGTTAATTAGAGTATTTTTTATGATTTTCTTTAATTTTATTTCAATTTGCTACAAAACTAATAATCTAGATTTTGACAAATTGTA
AGAATTAAAAAAAAAAATAAAGTGATAGTTTTTTTACTTATTCAAGAGTTAAAGAGCATGAAACATTTTTTTAAAAAAAGGAAAAAAATCTGTTGACATC
CAACACCAACACCAATTGACAACTTGAGAAAAAATAAAGACAAGAGGTAAAAAGATTAATATGATAGATAATGTGATGATATAATAAAAAAAGATAGATA
AAAATAGGAATTATTAGTTGTGTGGAAAATAAAAAATTGATAAAATATTAACATCCTTTTAAAAAAATCAGAAATTATTTGGTTGAAGAGAAAAATATAT
ATATTTAAAAATGTGGAGCCTATAGAAAAATTTGAAAATTTATTCTCCATTTCCCTCTATTTTTTATTTCAACCAATCCAAGGCTCAGCCTACAAATTGC
CCAACTTCATAGCTTATATTTCTCCGACAAAATTATTATCTACGAGACTACGATAGTGGCTGATCCACTAACCAGAAATGTCCAAACAAAATTATTATTT
TATTTTGTATCTTTCACCATTTGTTTTATTCACATTGCTTGTGTTCTTACTTAGTTTTTATTTTATTTTTACTGACACTTAACTTATGCTATTCACCATT
TGTTTTTGTTTTTCTTATTAACAATCATATTTTTATTTTTACTTGGCAGCATGAATATATTAGGCTGGACCATCATGGTATCTTTTGGGATGAACGCCGC
CACAAGGTTTGCTTTCGCATTACCTACTTTATGCTAATGAGAAATTAATTGTATATTAAAAGACTTAAATATATGTCATTATAAAAAAAACTTAAATATG
TTTTGATATATGATTAACACTGGATTTTTATTTTGTTCTCCAATATTTTTTAATGTTTTGTCCCTATTAATTATTTCATTTTATTTCTCTGGTCCTCACA
TTTGTTTAGGTAGCGTTTTGAAAATTTATTTACATATGGCTTAAGGAATCCGAAACAAAATAATTTTTTTTATTAGGAATCACAATTTTTTTTAAAAAAT
ATTAATCATTTATCAATAACTAAAAATATATTTAAGTGGTATAAAAATTAAAGATACATTATCAACACAATGTGTAACACTTCATATTTTTTACCGCTGA
TTTAAAATTATTAGACATATTGAAAATTTTTATGAGTTTTACTTCTTAATTATTTAATAGATCTCATTAACAAATTTATATTTTTAATAGTAGAGTGTAT
TTGAAAGAACATATTAAAAAGTATATTCTTAGTAGTCCTCTTAAATTTATACAATATCTTCTATAAAAAAAAATAAGGATAAAAATATAAGAAAATGTAT
AATAAATAGATTATGTGATTAAAAGATAATATAATATCCATATTTGGAAAAAAATAATATCCTTTAAAAAAAGATAATACAATAGAAAATATTGTAAAAT
TTGTATGGATAAGTTATTCAAAATATATTTACTAATCCATATTTATAATGTCATGATATCAAATTAAAAAAACAAGGATTGTACTATCATGATAAATATA
ATTGGTGGCAAAATGACCTCAAGGGTTTCTTTTAGAAACTTATTTCCAATAAGGGTCTTGGAGGTTTGTTTTGTGTAAGGGTTTTGTCGAGGAGTATGGC
GAAACACGTATTTTGCCATTCCTGTCGGTGAAATAGTGCATGCATGTCGCCATTAGGTGTGCCAGCCTACAGCAGCGTAGGGTTCATGTGCACGAAACAA
TGAGTGCATGTTGTCATCCACACTGGCAAAATACCAGTGCATGTCGTCATTCTCAGTGGCGAAACACATGTAGTGCATGTCGTCATTCTCAGTATCCGGC
ATAGATCTATCAGTATCCATTCAAAGGTCATCAAAGTGATACTTCTGCAAGCGAACATTCATTAGGTGGTGTTGCGGAGACATATCCCAATTTTCTATGG
CCTACTATGACTCCTTCACAACAACATGATGCTCCAATTCCAACACCTAATGTTGCGTTAGGTACTTAATGGAATGTACCCGGAGCAATACCTAATATGG
ATGAATTATTAGGTGTTGATTTACGTCACCAATTTTATGCTGAGGCGGACCAAGTTGAAGGGAGGAAACATCATGGCAGAAGAAATCTTGATAGACAAGC
ACGAAGATAGGAACGCTCATGCGGCACATCCTTACGTCATCATGACGATTGATTTTCGCATTACTATTTAAATATTTTATTGTTATGTTTTTTAATTTAA
ATTTTAGACTTCTATTATCGTTTATGTTATGAATATTTAGTAATATATGCAGTTTATGTCACGTACAAAAATATAATAATAAATAATTTTAAAAACAACT
TGATCACGAATAACTATCATTTCATTGAAGAAGACAAACAAAATACATAAATCAATTAATTTAACAACTTCCATATTCAGATTGCATTGGACATCGACGT
CTGTTGTGTCCTTCTCTTTCATATCTATTACATTTTTTTTTATGTTCAGATAGTTCGAGTCAATCCATCTCATTCCTTATCCCTGTTAATTTTGGTCGAC
CTTTCGCACGAATTGTACTTAGATCAGAGATAAGTGTCCATGAGTCACTAGAAGGAGGAATTATCGTTTCATTCCCAAGAGACCACTATTGTGCGGAGTA
AGCTTTTAAGACGTGGTCATTTGTGTAAACAACATCTATATATTGGAAATAGTTCATGCTCACGTAACCACATGTAGCAATAATGTGTGAACATGGATAG
TGAAGTGCATGATACTTTCCGCATTGACAACAATGGTCATTCAAGTTAACTGTCCACTTTTGTCCGCCACGTTGTGTTATAGGATTGAATATCTTCTCTA
CTTCAAGTCGAGTGGATATCATAGATGCAGACGATGTGCGAACAAACTTGTTCTTGATTTTTTTGAAATTCTTTAACAACCTTAGAACAATACACTTGTC
CTTCATTTAATTGTTTTTGGGTTTGGCGACCACGATCAACAAAGTACTTTTGACATCGACTATATGTTGATTTCACCAAAGTTGTTATCGGTATGTTGCG
ACAATACTTCAATACCTTATTTACACAGTTTGAGAGATTGATTGTCATGTGACCATATCTATGTCCTTCTTTATCATAAGCCATGATCCATTTTTCTTTT
AAAATGCGATCAATCTATGTTGCTATGGCTAGACTCAATTGACGAAATTTTTCTAAATTTTGATCAAATGTATGCTTGCAAGGAGTGTAGGTTGCATACA
ATTAGTTAGCAACAACAATTTTAAGTATATATGATACTTAAATTAACATCATATTATAAATTAAATCTTATCCAATTTCATCAACATTTCTTTTTGTTTG
ACATTGTTGAATTTGCGATTGAAATTGTTTGTTAAGTGTTGGACGCAGTAAACATGATAAGCGTGGGGAGGTTGCCAACCTAGTGCTTCGTTAGCAACAA
CAGACTTTATACTCGCGTGACGATTAGATATGAAACAAATACCATTTTTATCTGTCACGTGTTCACGCAAGTGTGTCAAAAACCATGATCATGCTGTTAA
CGTTTCACTTTCGACTACGGCGAATGCTAGAGGAGGAACACCACCATTTCCATCTTGTGATGTGGCCATTAACAGGGTCTCACGGTATTTCTCGTATAAA
TGTGTGTCGTCAACTTGTATGATTGACTTACAATAATTAAAAGTCTCTTTACATCTGAAAGTCCAAAATACTCCATGAAACTGACGATGTTCACGACTCA
CCCTATTCCTAACAATAAAATCGTCATGCAGTATTTGAAAATAGGATTTAGGAGAATTATTTTACATATGTTTTAAACACGATGAAAGTTTGGTATATAA
CTTTTCCCAATCGCCATATTCAATTGCAATCTCTTTTTGTTTCGTCATCCATGTTTTTTTTTGTACAAAACTTTATAGGCAAATTCAGTGTTTATCCTTT
CTTGAATCAAAGAAACTTTTATTGATGGATCTTTTCTAATCATGCCCGTTAATTAAATAAAAAAATTTAAATAACAAAAAATGCAAAAGTGGAATAATAT
GAACATTAAAAACGTACCTATTATACAAGTGACAATTAAATCTGAATCAAGCTTGTCATGATCTTGTGTCACACTCATATTCAGACATGTGTGTGGTCCA
CTCTATTGTGTCACTTTCCATGAATTAGCTTTTTTAGATAAGATTAACCTCATATAAAAAGGGCAAGTACACTATGAGATTTTATTCTGGCAACAAACGA
TATATTTGTGCGATTTGGTTTCAAGAACTTTAAAACTTTGATGCATCTTTATAATATATTGTTTCAGTGCATTTTTTTACAACATCTTTACTATCAAAAT
GCATACCAACATATAATTCTTGCCCAACATTAAAACTCGATGACATCTTCAAACCGCAAATATCCTCCTCATCAGGATGACACCAATTGATATTGTTATA
ATACAAAACATCATTTCAAAATGAATTTTGAATTCCTTCTATACCTAATACTTAAAAAAATAAAATTCAGGTCAATATCACAAAAATATTTATTTACCAT
TAAATATAATTGTTATAAAATGATTAATGCATTCAACTATTTTTTAATTTAATTAAATATACCTTCTCTTGGTTGAACAATTGTAACTGGTTCAACCATG
TGGGCGGCTTCATCATCTGTATCAGATATACCATCAAAAATATCATCTTCATCTAAAACTCTTCTACCTCTGAATTCAAACACTTTGCAAGCATCACCNN
NNNNNNNNNNNNNNNNNNNNNNNNNNNNNNNNNNNNNNNNNNNNNNNNNNNNNNNNNNNNNNNNNNNNNNNNNNNNNNNNNNNNNNNNNNNNNNNNNNNN
NNNNNNNNNNNNNNNNNNNNNNNNNNNNNNNNNNNNNNNNNNNNNNNNNNNNNNNNNNNNNNNNNNNNNNNNNNNNNNNNNNNNNNNNNNNNNNNNNNNN
NNNNNNNNNNNNNNNNNNNNNNNNNNNNNNNNNNNNNNNNNNNNNNNNNNNNNNNNNNNNNNNNNNNNNNNNNNNNNNNNNNNNNNNNNNNNNNNNNNNN
NNNNNNNNNNNNNNNNNNNNNNNNNNNNNNNNNNNNNNNNNNNNNNNNNNNNNNNNNNNNNNNNNNNNNNNNNNNNNNNNNNNNNNNNNNNNNNNNNNNN
NNNNNNNNNNNNNNNNNNNNNNNNNNNNNNNNNNNNNNNNNNNNNTTTTGATCAAAGTTTCTCAACAACAAAAATAAATGTCAGTTATAAAATTGTACAT
AGAAAAAGATGTTGCTGGTGGTTTTACGTTTCATTCAGCAAATTCTGATAAATCATGTGAAAATAATTTATCTAATAATGAGTCGAAATCGTCAACAAAT
GTAAACAATTTAAATGATGATGCTGATTATCTTGTGTCTAATTCATACGTTGAAGAGTTTTTAGATGAAGATGATAATATTGATGATATATCTGATACAG
ATGATGAAGTCATTCACATGGTTGAACCAGTTATAATTGTTCAACCAAGAGAAGGTATATTTAATTAAATTAAAAAATAGTTGAATGCATTAATCATTTT
ATAACAATTATATTTAATGGTAAATGAATATTTTTGTGATATTGACCTGAATTTTATTTTTTTAAGTATTAGGTATAGAAGGAATTCAAAATCCATTTTG
AAATGATGCTTTGTATTATAGCAATATCAATTGGAGTCATCCTGGTGAGAAGGATATTTGCGGTTTGGAGATGCCATCGAGTTTTAAGGTTGGGCAAGAA
TTATATGTTAGTATGCATTTTGACAGTAAAGATGTTGTAAAAAATACACTGAAATAATATGTTATAAAGGTGCTTCAAAGTTTTGAAGTTCTTGAAACCA
AATCGCACAAATATATCGTTTGTTGTCCAAATAAAAGCGCATAGTGTCCTTGCCCTTTTTATATGAAGGCATTTTTATCTAAAAAAATTGATTCATGAAA
AGTGTCACAATGGGGTGGACCACACACATGTCTGAATATGAGTGTGACACAAGATCATGACAAACTTGATTCAGATTTAATTGCTACTTGTATAGAAGGT
ACGTTTTTAATGTTCATAAAGGTGCATTAAAGTTTTAAAGTTCTTGAAACCAAATTAGACAAATATATCGTTTGTTGCTCGAAAAAAACACAGGGTATCC
TTTCTCTTTTTATATGAAGACAATTTTATCTAAAAAAACTGATTCATGGAAAGTGACACAGTGGGATGAACCACACATATCTGAATATGAGTGTGACACA
AGATCATGACAAACTTGATTCAAATTTAATTGTCACTTGTGTAGTCGGTACATTTTTAATGTTCATATTATCCCACTTTTGTGTTTTTTGTTATTTAAAT
TTTTTTAATTTTATTAACAAGCATGATTAGAGAAGATCCATAAATAAAAGTTTATTTGATTCAAGAAAAGATAAACATTCATATTATCCCACTTTTGTGT
TTTTTGTTATTTAAATTTTTTTAATTTTATTAACAAGCATGATTAGAGAAGATCCATCAATAAAAGTTTCTTTGATTCAAGAAAAGATAAACAGTGAATT
TGCCTATAAGATTTCGTACAAAAAAGCATGGATGGCGAGACAAAAAGCGATTGCAATTGAATATGGCGATTGGGAAAAGTCATATACCAAACTTTCATCG
TGGCTAAAACACATGCAAAATAATTCTCCTTGGTCCTATTTTTAAATACTGCATGACGATTTTATTGTTGGAAATAGGGTGAATCGTGAACACCGTCAGT
TTCATAGAGTATTTTAGACTTTCGGTCAATGTAAAAAGGTTTTTAATTATTGTAAGCCAATCATACAAGTTGACAACACACATTTATATGGGAAATACCA
TGAGACCCTGTTAATGGTCACATCACAAAATGAAAATGATGGTGTTCCACCTTTAGCATTCGTCGTAGTCGAAGGTGAAACGTTAACAACGTGATCATGG
TTTTTGGCACACTTACGTAAACACGTGACAGATAAAAATTGTATTTATCTCATATATGATCGTCACGTGAGTATAAAGTCTATTGTTGCTAACGAAGCAT
TTAGTTGACAACCTTCCCACGCTTATCATGTTTACTGCGTCCAACACATAGCAAACAATTTCAATCGCAAATTCAACAATGTCAAACAAAAAGAAATGTT
GAAGAAATTGGGTAAGATTTAATTTATAATATGATGTTAATTTAAGTATCATATATACTTAAAATTGTTGTTGCTAACTAATTGTATGCAACCTACATTC
CTTGCAAGCATACATTTGATCAAAATTTCGTCAATTGAGTATAGTCAGTGCAACATGGATTGATCGCATTTCAAAGGAAAAATGGACCATGACTTATGAT
AAAGAAGGACGTAGATATAATCACATGACAATCAACCTCTTAGAATGTGTAAATAAGGTATTGAAGGATTGTCGCAACATACCGATAACAACTTTAGTGA
AATCAACATATAGTAGGTGTCAAAAGTACTTTGTTGATCATGGTCGTCAAACCTAAAGACAATTAAATACAAGACAAGTGTATTGTTCTAAGGTTGTTAA
AGAACTTAAAAAAAAATCAAGAACAAGCTTATTCGCACATCGTCCGCATCTATGATATCCACTCGACAAGGTTTGAAATAGATGAGACCTTCAATTTTAT
AACGCAACATGGCGAACAAAAGTGGACATTTAACTTGAATGACCATTGTTGTCAATGCGAAAAATATCATGCACTTCACTATCCATGTACACACATTATT
GCTGCATGTGGTTACGTGAGCATGAATTATTTCCAATATATAGATGTTGTTTACACAAACGACCACATCTTACAAGCTTATTCCGCACAATGGTGGTCTC
TTGGGAATGAAGCTTATCCCTGATCTAAGTATAATTAGTGCACGAAAGGTCGACCAAAATCAACAAGGATAAGGAATGAGATGGATTGACTCGAACAATC
AGAGTACCGACAAAAATGTAGTAGATGTTGATGAGAATGACACAATAGGCGTCAATTTCCAATGCAATATGAATGTGGAAGTTGGTAAATTAATTGATTC
ATGTATTTTGTTTGTCTACTTAAATAAAATGGTAGTTATCAGTTTGTTTTTAAAATTATTTATTATTATATTTTTGTGTGTGACATAAACTGTATCCATT
ACTAAATATTCATAACATAAACGATAATAAAAATCGAAAATTTAAATTAAAACATACAATAAAATATTTAAAAAGTAATACGAAAATCAAGCGTCATGAT
GTCCGTGAGGATGTGCCGTATGAACATTCCCATCTTTGTGCTTGTCTATCCATCAGGATTTCTTTTGTCACGATGTCTCCCCAATTCAACTTGGTCTGCC
TCGGCAGAAAATTGGTGACGTAAATCAACACCTAATAATTCATCCATATCAGGTATTGCTCCGGGTACATTCCATTGAGTACCTAACGGGGCATTTGGTG
TTGGAATTCGATCATCATGGTGTTGTGAAGGGCTCATAGTAGGCCATGGAAAATTGGGATATGTCTCCGCAGCACCACCTAATGAATGTTCGCTTGCAGA
AGTATCACTTTGACCTTTGAATGAATACTGATACATCTGTGCCGAATACTGAGAAAATGCTGGTGGTGTGTAATACATTCCATGACCACACTCTACCATC
TGTGGGTACAAATATGGTCCCGCTTCAAGAGTCTCTCTTTGTCTGGCTATGCCTTGAGTTTCAACACTTGATTGGAGAATATTAAACTCTTGTGCTGAAA
ATTCATGTTCTGTTATCCTGTATTTTTTTAGAATAATCCTTGCCACTGATTTTTTATTCAACGGCTACAAGATCTACAATTCCGTGAGATTGGGCTTCTG
CTGCTGACATAAAAACATCCATTTCCATGTCTTCGGATATAACCCATAAAGGTTTGCCCGTTCTTTGTACATAAACCCTTGTGATAGTTTCACTCATTTT
CAGTAGCTCTTCCGCTTCCAGGATAAATTCTCCCGTTTGTGCCTCATAAAAAGAACTAGCGGGTTGGTGGATCATTACCCTGATTTGGCAAATTTACGTA
ACAGGGTTCCATTTAGAAACTTTTTACATAAATGGGTCTGTTTTCAAACAATTTGCACAAGGGGTCTCTTATTTGCATGGAAGCCGTTGTTGGCTTTGGC
GGTAAAGCATGACTTGCCAGTGGCAATGGCGATACAGGTTGATCCCGCCATTGGCACTGGTAATACGAGCTTATGTGAACACTAAACCGCTAGTCCCATT
GGCGATACGAGCCATGTGGAGTGCTTGCCACAAGTCCAATTGGCGATACGAGCTGGAGAGAGGTTGCCGCAAATTCCATTGGCGATATACAAGGGTGCTC
TCGCCTCTCTCATTGGCGATATACAAGGAGTGCTTAAGGTTGTTTTTGTTTTCGGCATTTTTTATGTAGTCTAAAGGTGATGCTGGTCTCGTGCTCATAT
TTAATTTGGAGACTTTTGCATTTTCAACACGCGCAATGAAAATTTTTATCTGAGATATTCCGTATAAGCTTCGCACGTAAGATCATTGCGTCTAGAGAAA
ATATCCATTAATAAAATAATGTTGAAAAATATATTTGTAAATTTACCGAATCAATAAATGCTATGAAGGGTACTATTTTTTTATGATTTTGTAGTTCATG
ATTTTGCTCAGGATTATATTTTAAATGCTTTAAGAATGAATTCTGATTCTATTCTTTTCCTAGAGGTAATTAGTTCTCTGGTTGGTTTATCACCAAAAAA
AATAAGAAATCAAATTTATAATTTTCAAAATAGCATAATACATTCCGTATAGATTATAAAAATATATAATCGGAAATAATATTGGTCATATCTTTTTTTG
TCATGTCTCTTTTATTTTGGCAATTACATTATTGGAAATCATTAATTATTAATTCAATACAAGATCATATTTCATGTGTCTCTATTATATAAAGATTGAC
TAAAATAATCCTTAATTTAACAAAAAAGGTATTTTTTTCTCTGATTTTTCAAAAATTATCCTTACAATGCATTTTGATTTTATAATATTTATGAAGAAAA
AATATTTTTCTTTTTTCAAATCAAATAATACTAATGAATTTCTTTTCTTTTCTTGTTTTGTTCATTTTGTCTATCTTGTTTTTTCTTCTTCGCCGAATAT
GCCAATCACAAATAAAAGAAATCTACTCCTAACTAAAAATGGAAACAAAAAAAAAAATATGTGCATGACGGTGACGATATGATGGAAATTGTTGGAAAAA
TATAATCATCGAATGAAATAGGACACGTGTGAAACGTAAATAATGAAGCGTAGACGCGAAACAATATATAATCATTTTATCATTATATCATTTCATAGTA
GTTAACGCTAAAGCAAGAAAACGTGTACACAAGCAAGCAAAGTGGCAAACATTGGCAAGTATACGGTTTTTATTTCATTGGCGAGAGCACCCTTGTATCG
CCAATGGAATTTGCGGCAACCTCTCTCCAGCTCGTATCGCCAATTGGACTTGCGGCAAGCACTCCACGTGGCTCGTATCGCCAATGGGACCAACGGTTTA
GTGTCCACGTAAGCTCGTATCGCCAGTGCCAATGTCGGGATCAGCCTGTATCGCCATTGCCACTGGCGGGTCATGCTTTACCGCCAAAGCCAACAGCGGC
TTCCATGCAAATAAGAGACCCTCTGTGCAAATTGTTTGAAAACATACCCATTTACGTAAAAAGTTTCTAAATGGAACCCTGTTACGTAAATTTGCCACCC
TGGTTTTATAACTTAATAAGTATTTCCCTTATCTTGATAAAGATTTTGATAATGATTTCTCCTATATTGGTAAAGATGATCCTTATTCCCAAATAAAGGT
AAAAGGGATAAATAACCATGTAACAGAAGTTCAGTGATTATCTCTTGCTCTTCAGATAAAATGGTTATTTTCTCCACATGCGGCACTAGATCATCCACTG
TCCATGTATTTATCCCTTGTGGAGACACCATGTACTGTAATGTCTCTGCAACTTCAGCCCGCAATTATTAGGTGTTCAAATTAATAGACTAAAATTTCAT
AAATAATTTGAACTTAAATATTAAGAAAAAATTGAAAAATATCAATGTAGTTGTGTTTGCATTCTTAGGGTCAACAAACATATTCGTTTTTTTGCCTATA
TCACACCATATAGTCGGAGTTAAAGCTCAGTAGGCCTTTTTGTCAAGGATAATCGTCAATCCTAAAATCAGCCCGATTGTTCCATTGATGGATCATTGGG
CCTAACAATTGCCCCCAATTTTCATCATGTTTTCCTTTCAATGTTATGTCATGAATATTAAAGGGGTGCAAAGGACACCGTGAAATTGGTTGTTGCATTC
CAAATTGTCTCACTCTATCAAGTTGGTGCCACTCAAGAACTTGGAAACAAATTAGTGGCACCACCGCGCACCACGCTACATTTCCAACTAAACAAAACAT
TTGTTGTGTAAGGCTCTCACACTATAATTTCAATGTTGTTTATTTTCAGTGAAATATGACATTATTTATTATTTAAGAAATACATTTAAATGTTCTTATC
TCATGTCGTTTCATAATATCTAATTTGCGACGAAAAAGTATCAAATCATCATTGTCGATATGTTGATTTTCACGTCGTAAGCCACCTAGAAAAAAATATA
AATTAATATAAGTTACATTATTAATTATTTTGAAATGAAATCTAATTTTTTAAACGATTAACCTGTGTCCAAGTGATTTATTTTCTATTAGTGAAGGAGT
CATCTTTGGAGTCAAACTTGTACATCGTTTTCATGTCCACATTTGTATTAAGACGCATGTCCACATTTGTATTAAGACGTACATACCTCCAATTGAATTA
ATTTTATAATCGGTGGCGGCGCTGCACATCTCTATGTATAAATAAGCAAGTACGACAGCTCTCCATGCATGCGTGCTGCACTATCTCTAAAGTCCCGTAA
AAATTGCTGGTACCTTAGTGAAACTTTGCTACTACCTTTGTCAACAAATAAGACACCTTCAATGAATCTTAGGATCCATGCACATATAAACCTTTCTATT
TGTTGTAATTTTCCGTCATGGTTATTTAATTCTAGAAAATGCTAAGCCCGCCAACTTAATTTAATCATACTACCTTGAAGTTCATCTTCTACTGACTAAC
AATTCTCCACATAAATAAGCTCAATTAAGGTTTGTTGAACCAATTAATGGTGACACATCTACACGAAGACCTAATAAAACAGATACATCTTGAAGAATAA
TAATGTACTCTCCGCATCTCATGTGAAAAGTATATGTTTTTGGTCTCCATCTTTCTATCAAGACGCTAATTAATGCCATATTTATTTTTAAATATCTCAT
TTTCATAATCCAGTAAAAATCAGATTGCTGAAGTAGAGAAATAATTTCGCCTGATATTTCTTCTTAACCCTGATATATGGGCACAACTCGTCTAAAATGT
AATTTTCTATCTTATTTTTCATTGCAAATCCGTTGTGAAACATGTTATCTTGCATTCATAACACGTCATCATCAGCTGAACCAAATTTAAGTTTTATATT
TGATGAAAATGATGAAGAAAATGCCATAAATACTATAAAATATAATATAATATAATATGTGACAAAAAATTTATAAAATATTTGTACATAATATAGTAAA
ATTAAGTACATTTATAAAAATAAAATTAAATATATATAAAAAATATTAATGACGCGATAGATATATTATACAAAATAAATTAAAATACATACAAAGAAAC
TAAACAAATAATAACACAATAAACATTATAAAAGTTAAATTCAATATATGCACAAAATTAATATATATATTAACAGAATAATAACTTAGCATAAATACTT
TTATAATATTAACATATTAATAATAAACTAATACCAAAATATATATGTAAAATAAATATATTATACACCTCTATAATACCAAAAACAAAAAAATTTAAAT
ATCAACAAAACAAAAAATACATAAAAAAATTGTTAGGATGAAATATTACCAAAGTTTCACTAACACCAACAACATAAAAAATGACTGCAGAATAAATGTT
TTACACACATATATGTATAAAAACATATGGTGAATAATTTCGGTAACAATAATATAAAAATTGTAGACTACAAATATATATATATATTTGTAGTGACTGA
GGGGGCGTTCGTAGTGACTGAGGGGGTCAAAGACCAAGAAGTGAGTTATTTATCAGCCAAGCATTCTATTCTTCTTATGGCTAGATCCAATCTCCTGGTC
CCTGTGGAAAGGAAAAAGAATTTTTCACGTTCTTCCTTTCGGGAAGGAAGGATTAGTAAAATCCTATTGATTGCAATTTTCTCCAGACCCCCGGGAAAAG
CATGAAAAAAAGGCTCGAATGGTACGATCCCACCGTCACCCCAGAACGAAAGGGGCGATCTCGTAGTTCTTGGTCTGTGAAGATACGTTGTTAGGTGCTC
CGTTTTTTTCCATTGAGGCCAAACCTAAACCTGTGCTCGAGAGATAACTGTCCATATACTGATAAGGGATGTATGAATTCTCGAGAAGAGAGGAGCCGTG
GTGGTCCCCCCCGGATCGCCCGGATCCCACGAGTGAAGAGAAAGTTGGATCAAAGACCAAGAAGTGAGTTATTGCCGGAATGAAGGGTTTTGCCACCACC
ACCAACGGAATAATTTGCTTTGAAGGTAGAAAACATAGAAAATCTTGAAGGCAGTGTGTGTATTCTGGACCACAAAAGGATCTTATATAAAGGGGAGGGG
GAAAGGGTGGAGGCCGAAATTTAAATGGAGTTGGTCGCTCCCACCTTCAACGAAACAATGGCTATGCCTGCAACCACGCTTTTGAAAAGGCTACTGCATG
GTGTTTCGCCGACATGGATGGCGAAACGCATGCACGTGAAAATATGCATTGGTGTTTCGTCAATGTGGATGACGACATGCACTGCATGTGTTCCGCCACT
GAGGATGGCAACATGCACTCACTGTTTCGTGCACGTGAACCTTGCGCTGCTGTAGGCTGATACACCTGATGGCGACATGCATGCACTGTTTCGCCGATAG
AAATAGTGAAATACGTGTTTCGCCATGCTCCTCGACAAAACCCTTACACAAAACAAACCCCCCATGGAAAATGTTTTACCCTTATTGAGAATTAGTTTCT
AAAAGAAACCCCCCTGAGGTTATTTAGCCATAATTGGTTTATGTAAGAATATAGAATGAACTAGGGGTGCGCCACCCAAGAGCAACATTGTTTTCCCTGG
TGGTTGCGGTGATTACTTCGGTTTTGATCGGAATTTTACTCGCTATTGTTTTGATGATCTTGCGGAATGAGTACCCTTCCCTGTTTTCAAACGACACAGA
AGGGCAAGATCTAGTTAAGAACCTCACACCATTTTTGTGCTTCTGCATTGTCATTAATAATGTTCAGCCTGTTCTCTCAGGTAACAAAATTAATTAAGAT
TTTCAATTTCTTATCTCACATTCTGTAATTAACTCAAACATTATTTTTCTTTTGATTACTAATTAAGAGTTCAATCCCTATTATTTTACACTATCATCCA
ATTATAAATCATCATATGTAATGTTGCAATTGGGTGATTGTATAAATTTTTTTATAACGTTAATGCATAATTTTTTCCATTAAAATTAATAGTAGGTGTG
ACATTTTAAATTTTGATTTTGTGCAGGAGTTGCCATTGGTGCGGGATGGCAGGCTCTTGTTGCCTATGTAAACATTGCATGCTACTACCTTTTCGGAATT
CCTGTGGGTCTTGTATTGGGTTACAAGCTTGACTGGGGAGTCAAGGTATGTCATTCTCAATTCTCTACTTAATTTATACGAATACTTTTGATGTATAATG
AATAAGTATACGGATACTATTTAGTGAAGGAGAAATTAACCAATTAGAGGTATGTATTTTAAGTTAATTGGAAGAGAGAATTGATTTTAGAAGAATAGTT
AATTAAGTCTTCCAAATATATATATACTGTTAATCTCACTGCTTATCTTTTAATAGGACTAACATATATAATTCATTTAATAATGAAAATGATAAGTGAT
AGGCTTCAATTTTTGTATATTGATTGGTGGATGAATGAATACTAAAATTTCAGCCATAGCAGAACACATTTAAATGTCAAGTAATTTTTTTTAAATGACT
AGATATAAAGGTCAAGTAAAGCCTAACATGTTCTTCAAATTCAAAAGGCAGTACGCAGTTCTTTAATATTGTTGATAATTGTTTGTTTTCAGGGAATCTG
GTTAGGAATGATAGCAGGGACTATCCTCCAAACGTGTGTTCTTTTGGTGCTGATTTACAAAACTAATTGGAACGAAGAGGTAAGATATTATTATTCAGTA
TTCTCTCATGTATATGAACAAATTGCAAGTTCAAGACGATGTTAAATTGCTTTCAAGCATACATATAATTAATTATTAAGTGGTGACTAGCACACAAAAC
CGCAGGCCTCCCTTGCCGAAGATAGGATACGGACTTGGGGTGGACATGAAAAAGCGACTGTGAAGGACACAAAAAACACAAAAGAAGAAACATGA

>Glyma.01G026200 | Chr01:2747392..2755356 reverse
ACACTTTTCTCTTTTACATGATCTCTTTCTATTAAATAAAATTAAAATGTTGATAAGCCAGGTCATATATATAAATTAACCTGGTCTGTAACACTTGCTG
TTGAGCCTATCAAAAAACTCTGCTCTGTCATTTCACTCCAACTCTCTCTCTCTCTGCAATATGAAACACTCAGATGGCAACTCCTCCACACAGCCACTTC
TCACGGCCAGGGCCGACGAACCCCATCAAATCCACCCTCACCCAGCAGAAACAACCGCAGTTTTTAGCGCCGGCACCCCTGACATAGCTCCGATCACCGG
CGCCGGCGACTTCTATAGGGAATTCATGGTGGAGTCAAAGAAGCTCTGGTACCTCGCCGGCCCCGCCATCTTCTCCTTCGTCTCAAAGTACTCCCTCGGC
GCCTTCACTCAAATCTTTGCCGGCCACGTCGGCACCATTGACCTCGCCGCCGTCTCCGTTGAAAACTCCCTCATCGCCGGCTTCTCCTACGGCATAATGG
TAAAATCCTTCTCTCCATAATCACATAACTACGGTTCATGATTGATGAAAGAGGCTACATCATATACACACCCTGATACACATGAATACTAATAGTATCC
GTGTCTAGCTAGCAGCATTTTATAATTCTACATTTTTATTTTTTAACAGTACATATTAAAATACTATGATACACATGAATACTTCTATAGTTTTTATTTT
AATTTTACACGATATTTTTTATCTTTTCTTAACACTTACAGATGCAAAGTGTTTGTTGAGTAGTATAGGCCAACAATTACATGGTACAATGTGTTTATTA
ACGATACAGTATTTTCTTACAAAAATCATCTTTCTATTTTCTGAATATATATATATATATATATATATATATATATATATATATATATATATATATATAT
ATATATATATATATATATTAAAATAAACAGTATCTTAGTTTTGGCTTTCTTTTATCATGACCAAAAATATTCCGTATCTTAGTTGTTTCACTGGCTGGCA
TAGTGGACCTAGTACTACTCCTTGTAGGTAGTATCATTAATTACTTGTTTCATCACTAGATGTAATAATGCAGTTTTGATTAGTCAAATAAAAAAAAATA
CATAAAAAAATATAGATCAGTTAATTAAAGAACACAAAACATATGGGGTGTTTGGTTTTATTATTTTGTGTTTTTATTTTCATTGGGAGTAAAAAATATG
ATAGAAATGTGTTTTTAAAATATTTTCTCAAACGAACTAAAAATTGAAAACAATAAAATCTCGTTTTTAGTTAAAATCAGGAACCTCATTTTGGATTAAA
TAAAAATACGATGATAAAAAATGTAATTTTAAACAAATCTAAAAATACATTTTTTTTAAAACACAATTTACAGCATTTTTATTTCTTGAAAGCAAAAAAT
AAAAAGTCAAACTAAACATATTTTCAAAATTCTAATCTTTTGATAATCAAAATAATTTTCAGAAAATAAAAACAAAAAATAAAAATAAAAAATAAAAATG
CAAACTGAACACATCCTAAATTATCATAAATATATTTTGATGTTTATTTTGGATAAAAAAAAATGTGATTCCGAGCAGCTAGGGATGGGAAGCGCGTTGG
AAACGCTGTGCGGACAAGCGGTGGGGGCAGGGAAGCTGGACATGCTGGGAGTGTACATGCAACGATCATGGGTGTTACTCCTCAGCACGGCCTGTGTACT
GTGCCCTTTGTACATCTTCGCGGGACAAGTTCTGAAGTTGATAGGGCAAGACACCGAAATTTCGGAGGCTGCGGGGACCTTCGCCATTTGGATGATTCCG
CAGCTGTTCGCTTACGCGCTGAATTTTCCGGTGGCGAAGTTCCTGCAGGCGCAGAGCAAGGTGATGGTGATCGCGGCGATAGCCGGGATGGCCATGGTGC
TGCACCCGGTGCTGAGCTGGCTGCTCATGGTGAAGCTGGAGTGGGGGCTCGTGGGCGCCGCCGTCGTGCTCAACGGATCGTGGTGGTTCGTGGTGGTGGC
CCAGTTGGTTTATGTTTTCGGAGGGTGGTGCTGGCCGGCGTGGAACGGGTTCTCGTGGGAAGCGTTTCGGAGTCTTTGGGGGTTCTTTCGCCTCTCTCTT
GCTTCTGCTGTTATGCTCTGGTGAGTGCTTGCAAAACACATCCACGTACGTGTTTTTTTTTTTTTTATCATTCTCATCATCTCTTTAAAATATGTAACAT
TAATTTCATAATTACAAATTTAATGAATATATGAAATAAGAGGATGAATTAAGAAAATAAATGAGTAATTAATTCTTTTTAAGTTATTTTTCATTCTTAT
TTTTATATTTTTAACATTTAATATTTATAATGAGTAATTAATTTTGATTATTAGATTTTAAGAAAATAAATAATAAAAATAGAAATAATTTTCTTAAAAT
TACTTTTGAAATAACTTAATTTCTTCCCATGAAATAGTAAATTAATATTCCAATAGTGACTCAGTTAACTACAAATGTTTAGTTTGTAAGAAAAAAATTT
AGTGATAAACGTTAAATGTTATTAAACTTGAAATGATGATTATTAGTCATTATAATCCGTCTAAAAGAATAAAAATTATAAAAATATTTTATTTTATTTA
CCAAAATAAATAAATAAATAATAGAATAGAAATATTAAAAAATGATAAGTATTAATGTTGAGAGCAATGTTTAAAATAGAGTGTTCAACAATGGTGTACT
ATTTTGACTACTAGTTTAGATTGTAGTAGTATGTGCACCACTTGTTGCACTTAAAAACAACAAGTGCAGGGAATCTGGTCTGATACCTGCGAACATATTT
TCATTTTATTATTAAAAATTTAACTAAAATATATGAGATAATAAAATGACATGATTTAATTAATAGGAAACAAGAAATATAAAAAATAATAAGTGTTTAA
TAATGAGATGTTTAAAATAGATAATTGCCCATGGCTAGTTGCTCTTTTACCTACTAGATTATATATTGTAGAAAGTAGAATGTACAGCACTTGCTTTAAA
ATAAACAAGTGCAGGGAATCTGGTCTAATACTTTTTAAAGCAAGTGCAGGGAATCTGGTGATTCTGGTCTGATACATGTACACATATTTTGTTTTTTCAT
TCAACAAAAAAAGAAGCATTCCTACTATGATACTAGTATATTGTTACCACAGCCGTTTCCAAAAAAAAAAAAAAATTATTGTTACCACATAAGGTATTAT
TTATGTTATGTTATATTGGGAAATAAAACAAAAACTTTTGGATCTTTGAAGACTGAAGTTACAACCAACAAAAGCCAAGTGGTGTGGACGTGAAAGCTAC
AGAACAGAGAAAAGATAATTATATTATCTTTTAAGTTTTTTTATCATAGCATTTATACTACTTTTTAGTTTTTTTCTCATTAAATAATGAATGAGAATAT
TATTTTTTAGTTTTCTTAAAAAAAAAAAAATTAAAAGTAAAAGTGTGTGATAAGAAGAGACATGAAAAGTTATAAACATTTGCGTATTGATATTTCAGTA
TATTTAAAACAACATTATGTAAGAGAAAGATTAAAAAAAGGAAAAAGTGTGATATAAGATAAATAATGTGATGGAAAATGGTAAAGACTAAAAATAATTA
TAAAGTATTATAAGAATTTGTTATATATGTATTATTATTTTAAATATTAATTTCTTCCTCAATTTTTCTATTAAAATAAGAATAGTATAATTTCTTATTT
GAAGAAGATCATAATTTATTGTAATAAAGAGATCGATTTGTTATCTTCTTTGTTATCAATAGATTTTTAATTATTAATTTGTGTAATTTAAATCTTTTAT
GAAACAAATCACATGCAAAACAATTATCAAAAGCTTTTAATGGGTTTTATATGGTTAATAATGCTGTATATATGTGTAATGAACCAATTATACACAAAGT
AAATTTACTTTATTTCTTATTGAGAATTTTCTTTTATTTTTAAAAGAATGAAAGTATATATAGACTGTATAGTCTTTGTAACTCTGATAACATATTATAT
GTCAACTACTTAACAAATTAATTTAAGTTATTAAATAAGTGTGAGAAGGTACATAAAATATCTAATATCAGTTATTGAACCAAAAATTAAACAATACTAT
AAACATAACTAAAGTACGTGTGTGATGCAATATAAAATTAAATCAATGTTAGGTAAGAACATCCTATTTGACTAGTATTCCTGTTTCATGTTCGATGGAT
ATCCATTTTTTTAATCTTAAAAGACTGGAATATTTAATTTTATGTAAGAAAGTTATGTATATTTTCTCTGATGCATCATGTGCGCAGCTTGGAAACATGG
TACTTTATGGCTTTAATTTTGTTCGCTGGATACCTCAAGAATGCACAAGTCTCCGTAGACGCCTTCTCTATCTGGTGAGTGAGTAATAACAAGTTGTGTT
TTCTTAAGTTCTTGAAGAACATGCGATAGAGTATATTTATTTCCATTTGTCAAGAAAAAAAAACACCCTAAAAAACTAATGATCTAGATTTTGATAAATT
TTAAGAATTAATAAAAGAAATAAAGTGATAGGATTTGTTTGCTAATTCAAGAGTTAATTAAAGAGCATAAAACATTTTTCTTGTTATAGATAATTCAGCC
TGCAACTATACAAGCTTATATTTCTCAGACAAAATTATTAGTGGCAGATCCACTAACCAGAAATGTCCAAAGAAAATAACTCTTTTTGTATAACTGGGGT
TGGGTATTTATTATTTTATTTTGTGTCTTTCACCATTTGTTTTATTCACATTATTGCTGACTTTGTAGCTTTTGTTATAATGTTATTCTCTTTTGTTCTC
ACTTAGCTTATGCTATTCATCATTTGTTTTTGTTTTTCTTAGCAACAATCATATTTTTATTTTTTACTTGGCAGCATGAATATATTAGGCTGGACCATCA
TGGTATCTTTTGGGATGAACGCTGCCACAAGGTTTGCTTCCGTATTACCTACTTTATGCTAATGAGAAATTAATTGTATATTAAAAGACTTAAATATGTT
TTGATCTATGATAAAGACTGAATTTTTATTTTGGGCTCCCATACTTTTTTTAATATTTTGTCCCTAATAGTTTTTTATTTATTTATGGTCCTCAAATTTG
TTTAAGTAGCGCTTTGAAAATTTATATATATACGGCTTAGGGAAAACGAAACAAAATAAAAATATTATTAGGAATCAAAAAGTTAAAAAAATTATTAGGT
ACCAAAGAAAAAATGATCATTTATCAAGAATTAAAAATATATTTAAGACAGATTAAAATTAAAGATACATTAACAAAAACTCTAAAACACTTAATATTTT
TTTTCATTGATTTAAATTTATCGGAAATATTGCAAAATTTTGAGTTTTACTTATTTAACTAATCTTATTAACACTTTTATAATTTTTAATATTAGAGTGT
ATTTGAAATAATATATTAAAAAGTCTCTTCTTAATACTCCTCTTAAATTTATACAACATTTTATATAGAAAATATGGATAAAAATAAAAGAAGATGTGTA
ATAAAATAGATGACGTCATTAAGAGAGAATAAAAGACAATACAATAGAAAATGTTGTAAAATCTGTATGAGTAATTTATTCAAGATATATTTATTAATCT
ATATTGATAAAATCATGATATACAAATTTTAAAAAATAAATAAAAGGATTGTACTATCATGATAAAGACTATTGTTTTTTTATGTAAAGTTTGGATGAAA
TTTCAGTGTGAGAATATCGAATGAACTAGGGGCACGCCACCCAAGAACAGCATTATTTTCCCTAGTGGTTGCCGTGATTACTTCGGTTTTGATCGGAGTT
TTACTCGCTATTGTTTTGATGATCTCGCGGAATGAGTACCCTTCCCTGTTTTCAAACGACACAGAAGTGCAAGATCTAGTTAAGGACCTCACACCATTTT
TGTGCTTCTGCATTGTCATTAATAATGTTCAGCCTGTTCTCTCAGGTAACAAAATTAATTAAGGTTCTTAATTATTTCATATATTCTTTCAAGTTACTCA
GTCATTTTTCTTTGATATGAGAGTCTAGCTTAATTGTTTGAGTATAACTTGTGAATTATTATAAATCTTTCTATATTTACCTTTTATTACCAAGAATAAA
AAAATATCATTTTTCTTTTAATTATCAATTAATTTTATGTATGACATTTTTAAATTTTAATTTTGTGCAGGAGTTGCCATTGGGGCGGGATGGCAGGCTC
TTGTTGCCTATGTAAACATTGCATGCTACTACCTTTTTGGAATTCCTGTGGGTCTTGTATTGGGTTACAAGCTTGACTGGGGAGTCAAGGTATGCCATTC
TCAATTCTCTCCTTTATTTATAAGGATACTAATTAGAATGAATTATATCTTCCAACAAATTTTAGTGAAGGAGAAATCAAGACCAATCTTAATACTTGTG
GTGCCCACCAACAACACTGTATCAGAATCTTTCCTATCATTATGAGCAAGATTTGCTTCATCATTCAATCTTTCCTTTCCTCCTTTGTTACTTCTACATT
CATCTTCAAAGTGCCTCCACTTGTTAAAGTTGTAGCACTAAATCTTCCTCTTATCAAACTTCTTTTTCCAACTCTTATTTTTCTAGATTGGCCACCATTT
GTTTTATTTGAGAATTTTCCTTCATCATGATCGTACTAATCTTGACCATTTGTTGATTCACTGCTCCTTCCCCCTTTCCATTTGCTTTTTCCTTTCTTGT
TCCATTCACCTGCACCTTTATTCTTGAAATTGTTGAGCCTGCAGAGCTTGTTCTTGAGTCTTTTCATCATCTCCTCTCATTGATTCTCTGCACATCTTCT
TCAAGAGAGTTTTGCAGCTCCTCCACCTTCATTAAATTAAGATCTTTTTATTCTTCAATTGCCACTACAACATGATCAAACCGAGGTGTCGAGGTTCTTA
GCACCTTGCCAACAACTTGTTGATCTGTCAACTCTTCTTTGTAGGCCTTCATGGCATTGACAAGCATTTGAACTTGGCTGAAGTAGTTAGCAATGGACTC
ATGATCAGACATAAACAATAATTCATATTGCCTTCGAATAATTTGTAGCTTCACCTTATTTGTCTTGCCAACCCCTCCATATGTTTTATTCAGAATATCC
TAGGCTTTTTTTGCTATAGTAGCTTTGGAAAATTTTCAAAAGATTGCAATATCAACACACTGATGCAGAATATTCCAACTCATTATACAACAAACACTAA
TTGACTAGATGACACACCTACAATCAGAAAAACAAATTATTATTATTCTTGAGAACAGACGAGCTAATTACTTAACGACACACCTACAATCAGACTGTAA
AATTATTATTATTTTAAAATGGCTAAATATAAAGGTCAAGTAAAACCTAACATGTTCTTCAAATTCAAAAGGCAGTACACAGTTCTTTAATATTGTTGAT
AATTGTTTGTTTTCAGGGAATCTGGTTAGGAATGATATCAGGGACTATCCTCCAAACATGTGTTCTTTTGGTGCTGATTTACAAAACTAATTGGAACGAA
GAGGTAAGATATTATTATTCAGTATTCTCTCATGTATATCAACAAATTGCAAGTTCAAGACAATGTTAATTTGCTTTCAAGCATATATATAATTAATTAT
GAAGTGGTGACTAACACAAAATTAAATCGCAGGCCTCCCTTGCCGAAGATAGGATACGGACTTGGGGTGGACATAAAAAAGCGACTGTGAATGACACAGA
AAACACACAAGAAACATGAAATTGCAGTAGGAGAATTGGTACGATATGAAGTATGATACTTGAATTGTTGACAGAAAAGCTTTAGTTGCTTTAGAATATT
CTATAAAAAATTGTTGCAAGGCCATTATTCAACTTCTTATTTATTTTAGAAAACACAGAAGAAGGGAACATGAAATAGTAGGAGAATTTCTTTTTGGTTA
ATAAATCGCAGAAAAAGAAATGCATGGTACCATACAACATCACGACGTACTATATTTGAATTGAG

>Glyma.17G247400 | Chr17:40283791..40290776 reverse
TGTCTGATATTCAAAGGCCACTTGAAATTCTCGTTTCTTAAAAAAAAAAAAAGAAAAGGGTATTTGAAATTGATATTAGTAGCACAAGTAAGTAGGTGTC
TCATTCAGTGCCAAGTAACCTACTAACCAAGCCAATGGATTCTCCCTTACTAGAAAACATTGACAACTCTTGTTCTAATGTTGAGGAGGAGAAGACACCG
AACACTGTTGTTAAGAGATTTGGGTTTGAGTCGAAGAAGCTATGGAAAATAGCTGGCCCTGCTATCGTTACCTCTATATGTCAGTACTCACTTGGTGCAC
TCACTCAAACATTTGCTGGCCTTGTTGGTGACCTTGATCTTGCAGCAGTTTCTGTTGAAAATTCTGTTATTGCTGGCCTCGCCTTTGGAGTCATGGTACT
CAAACAATTTGCTTTCTTTAACTCAATTGTTTGAAATCATGTCATGAAGAAATTTGATGCTTTGATTCAATTCATCACTTTAATTTTTATGCATTGTCAG
TATAAACGGTTAATCATTCAGAAATCATCACTGACATGACTACTAAGATTGTCATTGTAAAATTCAATAGCCGTATCATACATAATGCTTTGTAGTTGAA
TGATAATGTAAAAACTTTGATATTACCCATGCAAAAGAGGCAGAGGTTTTGAGAAATCAACATGAAATCATTCACATTTTTATTTGATAGCTTAAACAAA
CTGTTGGACTGTTGTTGGATAATGTACTCATTCATGTCCATGGCATATATATGTAAAGTTGATGCATTGATGTCTATGGCATGTTTTTGTTGAAGAGGTA
AAAATAAATGGGCAGAACATAGTGGGAAGGAATGGGATCCCTGCAGTTCATATTTTAAGTGCAGAGGATCTGCATTGCTAGTGAGAAGCAAAGTTATATT
TTTCATTCTGCAATGCTAAATTGTATTTCCAAAGTTAAAATTGGTTTTAGCACCAAGGTTAGATTTTAAGTACAAGAAAGGTTTTAGCACAAGTGGTCTT
CATAAAACTGGTTTAATTATAAATTAAGAAAATACTATTTTTTTTATTTATTAATAAGTGTAAAAGCTATTCTGAGTTTATTTTATATCGTTTTAAATAT
TAATTATACAGAAAAGTGAAAAAAAAGTAATAATTTGGAAGAGTTGATTAAGATGTGAAATTGATGGATTATGTTTTTGTTGAGATCAGTTGGGAATGGG
AAGTGCCTTGGAGACTCTGTGTGGGCAAGCATACGGTGCGGGACAGATCAGGATGTTGGGTGTGTACATGCAGAGATCATGGGTCATCTTGTTCATCACT
GCCCTTATTTTGTTACCTCTTTATATTTGGTCACCTCCCATCTTGAGGCTCGCTGGACAGACGGCTGAGATCTCAGATGCTGCTGGTCAGTTCTATACTT
CACTTCAAATCATTCTATATCTTATAATAATTCTCTTACATTTACACCGACCATACAAAAAAACATTCAATAAAACTTCAAATCATTCTAAACTTAATTT
AGGAGACAAAATACTAGCAATATATTTTTTCTTTATTTATAAAAAATGAGCTCAAATACCAGAATATAATACAGCTAGTTTGTGTAGTGTCATTTAGTGT
GTGATCACCTTTTCAAAAGAATTCTAAATCCCTGAAATAATGTTATTTATTTACTTTAAATAACTATTGAGGATTCAAAAAATTGTTTTTATTTTGAAGG
GATAAAATCCAGAGACTTTTTGTTTTTAAATGACTAAAACTAAAATTTGAAAATGGAGATCCTCAGTCTATATGATAAGTTTTTTTGCAATTTGATTATA
TGGGTTGGGAAGAGATTTTTGAACCGAAGTAAAAAAATAGATCTAATGACTTAAAAAAATTAAAAGAGAAGTCATATGAAATAAAAATATCATATATAAT
AAAATGACAATAAAGGTGACTTATATGTCAATTTTTCAATTATTACACCCAAAAAAATCAAACTCTTATAAATAGACCAAATTCAATAGTTTCAGACTTT
AAAAGAAAAGTATACAGATTTATGACAACTAAAATCATATTTTAAGTCATATAGTATTAAATTACTCGTGAAAAATAGAATCCAAGGTACACATTATTCT
ATCGGTGGACCACAGTCCTCTTGGAACTTGACAATTATTTCCTCCTAAATTTCAACAGTGGGGACTACCGGAACAAGTTGTCATAAATTAATGTTAGGAA
AATAGGCAACCGCGCATGTGTACTATGCAGCAGGTATAAGTAGAACGATAATTGTATATACTAATCCTACTAAATGAGAATGAGACCTTTTCATATATTT
TAGGATAACTTTAATTCAAAACTACTTAAATATTGCATAATTTAAATATATTTTAGGATAACTTTAATTCAAAACTACTTAAATATTGCATAATTTAAAT
AAATTTTTAAAATAGAGCTAGTCATTATGAATAACGATTTTTAAATAGAAAATGTATATGTGAACATAACTTATTACTTTAAAAATAAAATAACTGTAGA
AAAAAACAAATCATAAAATAAATAATAAATAAATGTTTAATAATACTAATGAATTTATTATTATTATATATATATATGTGTGTTTATATGCATGTGTATG
TATGTGTGTTCAGACATGTATCTCTTTGTCTATGTGTATGAAAGTGTAAAAGTTTGAAATATGATATTTTTCTAAACTTTTTTAAAATGTATCTCACATA
TATATATGATTTACAATTAATTCATTTTTTAAAATTTCTTATTTTTTATATAAATACTTATAAATTAAAAAATATCCTCCAGAGATCAAAACAATAAAAA
AAATTATAGGAATAAAAAAAATGAATATGTAATATAAGAGTTAGAAACATATTTAAGTTAAGTAAGTGCATAAATTCTTTTTTTATTTGGTTGGATAGAA
AGTAGAAAAAAAAAGAGAAAAAAGTTGACTAAAAAAACAAAAATTTGAATTTTTTCTTTTATTTCCTTTCCAAATCAACTTTCAAATTTTTCTTTTCTTC
CAATCAAACGGACCATTAAGCTTCAATATTATTTCTGCTGAGTGGTCAGTGGTCAGTACTCAGTTCCCTCCATCCATCCATTGGCTCTTGTCTATCCAGA
AAAAAGAGTCTGGATTGGAAAATGGGTCAATTACTGGTGACCAGTGAGCATCACCTCTTGCACTTTCCTTTTACAGAATAGTAATTAATTTTCCCTGGTG
TAAGCTGAAAATAGTTGAGTATTTACTGGTTTACTTGTAATTAATAGTCGATGTGATTTTTTTTTTAAACATCAATAGATAATCTGATTATTTTGGTCTG
TAATAATTTTTTAAGAGTTTTACATTATTCCATATAATAAAAAAATAAGTAGATAAATAATTATAAAAAACATATATATATATATATATATATATATATA
TGTGTTAGAAAATTGTTTGATATTCTCTTTTCTTTTTAATATTAATTGGTGGAAATTTATTGAAAATCATAAATTTAGTGGATATTACATCTCATTTAAT
GATTAGCTCTCCTAGTTTAGATTGAGATCCACCAAAATTTTATTGAAATGCTTACACATTAAAAATTATTATTATTATTATTTACTTGTACTTAATTTTT
GTAGGAAAGTTTGCTGTGTGGATGATACCTCAGTTGTTTGCATACGCTATAAACTTTCCAATAGTAAAGTTCCTACAGGCACAGAGGAAAGTGCTGGTGA
TGTTATGGATATCAGTAGTTGTTTTGGTGCTGCACACATTTTTTAGTTGGCTTGTTATATTTAAGCTTGGATGGGGCCTAATTGGAGCAGCAGTTACTCT
CAATACATCATGGTGGGTGATTGTGATTGCACAATTGTTGTACATTTTCATCACTAAGTCAGATGGAGCATGGAGTGGATTTACATGGCTAGCATTTTCG
GACTTGTTTGGTTTTGTAAAGCTTTCTTTGGCTTCAGCTGTTATGTTATGGTATTTTTCTCAAGCTAACTTCATTTTGCATTGGACCTTGAGTGTATATA
TTCATATTTGCTGAAAATAAATATGGTGTTAATTAAATGTGAACTCTTGCAGCTTGGAGTTTTGGTATTTGATGATACTGGTGGTGATAACAGGGCGTTT
GGAAAATCCTCTGATACCTGTTGATGCCATATCTATCTGGTAAAAAAAATTCTCTACCCTCCCTCCCCCTCAGTCCCTTTTAATTCTCTTATAATGGCTT
AAAAATGGTTTCTGACCCTTTTATTTTTAATTAGTTTCTATTTAAATTTTGTTTGTTTTAAGTCTTTAAAAATTACAAAAAATTGTGTTTGTACTTTGTA
ATCCTTGTTCAAAGATTACAAATGCATTGTATTTTTTTTATTAAATATGAAGACTGGTAACATTAAAATTTAAATAAAACAGCTGAAGAAAATGACATGT
ATTCATATTGCAAAACTATAAAGTTAATTTAGTTTCTGAACTCATTGCCACTTTCTTTCTTTTTTTACTGGATTCATTGTCACTTTAGTTTCTAGAAATA
ACGAAATTATATATAAAAAAAAGTCTCCTTGTCACCCACTTTAATCTCAAATTACATACAATGTGTGACTTAAGGGATTATTAAATTTAGGAATTTGATG
AAATCGAAGGACTAGAATAAGAGCGAATTACAATGTGCTCTTATTTTACAATGTGTCAACATATCAAAATCTAAGATCATTACTGATAACTAATACTTGT
TTCCATGTTTGCAGTATGAACATAAATGGTTGGGATGCCATGATTGCGATTGGGTTCAATGCTGCTATAAGGTGATCATCTCTCTCTCTTCTTTTTCATA
ATACAGTTCTAGTCACTCCATATATAGAAAACCTCTATGTTCCCATAACCTAATGAATAGTTGGTTTTGAAATTTGGATTGATTTTCCATGAATCACATT
GTTTAAACTTATTTTTTCAAAATAACTTTTTTTAATAAAATAAATAATTTTTTATTTTTCTGATGTGTTTGTCAAAATTATTTTTATTTAAATTAAACAA
TTTTTTTATTTTTAAAAAACAAATTTTATCTACTTAAAAATACTTTTTAAAAAACACATATTTTAAAATTATTTATTTTAAATTTAAACAAACTCATCTA
ATTCCTCATTAATCATGTGGGGACATTATATAGGGGTGACTAATGAATATTATTCTGAAGTAGTACTTGATTTAATTTATGGACAGTGTGAGAGTATCAA
ATGAACTTGGAGCTGGTGATTTCAAGGCTGCAAAGTTTTCAGTGTGGGTGGTTTCCATCACATCAGTGTCCATAGGTGTTGTTGTCATGATCGGAGTGCT
ATTAACAAAGGATTACTTTCCCTACTTGTTTACCACCAGTGTTCCAGTTGCCAATGAAACAACTAGGCTTTCTGCTTTGCTAGCTGTCACAGTGCTTCTA
AACAGCCTTCAACCAGTCCTATCAGGTTAGTCCCACAAACAATGAACTCAGGCAAATTACATTATCCATTCAAGGATTACATTTTCTTTTTTACAAGTTA
AATGCAACTTTTTAAGTACTCTTAGTGAGTTAGTGTTGGCTTTAAGGAGAAAACTTACATGTAATTTTAGGGGTTCTAAGTTGGATTTGATGGTGAATGA
AAAAAAATTTAGAAAGATTATGAGTTTGAATATTTCTACTAATTAATAACTGACAATTAACATGGATCCATAAAAAAAGTTGTTCTATATATGTTGTTGA
TATTGTTCTTATATTAGAATGGAGGGACCCATTAAAAAAATAATAGAATGCAGGTTTCATCTAACTATTTTTTTGTACTGTTATTGTAGATTATCAAAAG
AAAACTTCAAATAGGATTGAAGAACAACACTAACAATACTTATAGAATTATATCTAAGTTTGTCTTATTTTTAATTTGAATTAGAGTTTTCTCTCAATAA
CTTATGTAGATCTTTAATACAAACTTTTATCACGTAAATTAACAAGTTGAAACTTAAATTTTAATTACAAAATAACACTAAAAATGTTTAAAGAAATGCA
TTTAAGTTCTGCTTTTTTAATACTATTTTATGCCTTTGTGGTGACTGGTTTTAGGTGTTGCTGTTGGGGCTGGCTGGCAATCTCTAGTAGCATACATCAA
CATTGTGTGCTACTATCTAGTTGGATTACCGGCAGGGATAATATTGGGATTTAAACTTGGTCTTGGAGCAGAGGTGATGTACCATCACTACTTTCTCTCG
TCTTGAAATTATACACCAACAGATTTGGAATAAAGTGCAACAATTATAAGAAGGGATTCATAATTCATAATCAATAACATTATCAATCTTAACTGTAACC
AATAAAACATTATCAATCTTAATTGTTTGCAATTTTATTTATTATTGTATTAGTAGTGTACTTTTTATTCTTACAGCTCTTTAATCTTTATGCCTGTGTA
ATTCAGGGTATTTGGTCAGGGATGATTGCAGGCATAGTTTTGCAGACTACAATCCTTATAATCGTCACTTCAATACGAAACTGGAAAAAAGAGGTTGGTA
CTTTACACATTTATTAATTTCTCTCTATTTAATTTAATTCTGCTAGAGTTTAAGGAAGTTCCAAAAGTAAATGTTCCAATTTAGAGTTAAGGTGATGGGA
ATTAGCAATCTTCTTTTTCTACAAATAAGTAACATGTTATTTGTTCAAATTTAATTTGTTGCCAATGGTTTTCACAGGCTGAAGAAGCAGAAAGCCGCGT
GAGAAAGTGGGGAGGAGCAATTTCATATGATCAATGATTTTTTTTTATCATAGTAGAGAATTAGACAGAGACTCTATTGAGTGGCACCAATAAATGAAGA
ACTTTAAATTTTTCGACATGTATTTGAGTGAGGACTTGGAAATTTAATTTTAGATTATAGGCCAGCTTGTTATGATTTCTACATGGATATTTCACATGAT
TGAGCACCATTTGGTTGAGCACTAATTACAAGTGTTTAACTAAGTTAGTAGTATTTAAACAAAAGAATCTCAATCTTATCTATAAA

>Glyma.14G078000 | Chr14:6600960..6609330 forward
TAATTGCAACTCGTGCTTCGACCTCTTCCTCACTCTCACTCACCATTCCCATATTCATACCCCATTTCTCATTTCCAACACAGTGTAGTATATAATAAGC
TAAATCAATCTTTGTCTGATATTCAAAGACCACTTCAAATTCTCATTTCTTAAAAAAGGGTATTTGAAATTGATATTAGTAGCAGTAGCACAAGTAGGTG
TCCGATTCAGTGCCAAGTAACCTACTAACCAAGCCAATGGATTCTCCCTTACTAGAAAACATTGACAACTCTACTACTTATGTTGAGGAGAAGAAGACAC
TGAACTCTGTTGTTAAGAAATTCGGGTTTGAGTCGAAGAAGCTATGGAAAATAGCAGGCCCTGCTATCCTCACCTCTATATGTCAGTACTCACTTGGTGC
ACTCACTCAAACATTTGCTGGCCTAGTTGGTGACCTTGAACTTGCAGCAGTTTCTGTTGAAAATTCTGTTGTTGCTGGCCTCGCCTTTGGAGTCATGGTA
CTCAAACTTTGTTTTCCTAAACTCAATTGTGTTTGAAATCATGTCATGAAGAAATTTGATGCTTTGATTCAATTCATCAGTTTAATTTTTATGCATGGTC
GGTATAAAGGGTTAATCAATGAGAAATCATCGTTGCCGTGACTACTAAGATAGTCATTGTATAATTCAATAGCCGTATGTTACTTATCATGCATTTGTAA
TTGAATGATATTGTAAAAACTTTGTATATTATCCATGCATAGAGTATTTACTCTACATTTATTAGCAAAAGAGGCAGAGGTTTTTGAGAAATCAACATAA
AATCATTCACTTTTTTATTTAATAGCTTAAACTATCGATTAGATGTTGTTGTGTTGTTGAGTTATGTGTTCATTCATGTCCATAGCATATAGATGTTAAG
TTCATGCATTGATATATATATATATATATATATATATATATATATATATATATATATATATATATATATATGTATGTGTGTGTATTAATTATCAGCAATG
CTGAATTTTACTTGTACCAAATATCTTCGTAAAACTGGTCTAATTATATATTTAGAAAAGTTAAAACTTGTCAGAAAATAAAGTTGAGTATTGATATTTA
CTTATTTAGTTTTCTTCTTCATAAAATTGGTTTAAATTAAGAAAAGTAAAAAATTGCCGGAAAACACAGTTGAGTATTGATATTTATTTCTTTAGTTTTC
GACTTTTCGTTGCCTGTATATTAATAACAATTAGCTATTTTTATTTATTAAGACTTAGTGCTATTTATACAACAATTTACATAATATTTCATTTATTTAT
TACATCTAATATTTTTCTCTCTTTTCTCTTTTGCCTTTTCTTTTCATGGTATATATAATTTATTGTATGAGTTATTTTAACTATAACATTTCTCTTTTAC
TAACATTATCTTCGGACTTACTAATGTTTAAGAAAATACTATTTTTTATTCATAAGTATAAAAACTATTCTGAGTTTGTTTTTTATTTCAATATTTATAC
AGTAAAATGAAAAAAAAAATCATTTGGATGAGTTAATTAAGATGTGAAATTGATGGTTTATTTTGTAATTAATTAATTATGTTTTTGTTGAGATCAGTTG
GGAATGGGGAGTGCCTTGGAGACTCTGTGTGGGCAAGCATATGGTGCGGGACAGAGCACGATGTTGGGTGTGTACATGCAGAGATCATGGGTCATCTTGT
TCGTCACTGCCCTTATTTTGTTACCTCTTTATATTTGGTCACCTCCCATCTTGAGGCTCTTTGGACAGACGGCTGAGATCTCAGATGCTGCTGGTCAGTT
CTATGCATCACTTCATATCATTCTAAACTTATTACTTTAGGAGACAAAAATACTACTCCCTCCTTCCACATTAATAAACGTGTTAAATTATTTTACTCAG
ACAAAACAATAATAGATGAGAATGATAATTTTATAAAATTAACCTTATATTATTATTATTTTATTTATAAATTTTATTACTCATCATTAATATATAAGAA
ATCTGAGTGGAAAATATAATATTATATTAAAAAAGTTAAAATGAGAGTTATTTTAAGACAATTTTTTTTTACATCATAATTATAATAGGATCGAGAAAAT
AACAATTATTTAATTTTCTTTATTCATGAAAATTGAACTCAAATATATTGAGGATTTGAATCTTGCATCTCCCAATTTTTTAAAAATCCTAAATTTCTCT
TTTCATATTATATCATGTGGAATGGGGTTTCATAATAATAAAAATTAGTTTTCAGAATAAGTGTTTCATAACAGCAAAAATAACTTTTGAAATTGACGTT
CTGTACTAGTAAAAAAAATAGCTTCCGAAATAAATGTTATATAACAATAAAAAATAACTTTCTCTTATGAAATAAACATTCCAGAATAAATGTTTTATAA
CAGTAAAAATTAACTTTCGAGATAAACATTCCACATCAGTAAAAAAGATAACTTCCAAAATAAATATTTCATAACAATAAAAAAATAATTTTGATTTTTA
AAATACGTATCCCATAATAATAAAAAATAACTTTAAGAATAAACATTGCAATAGTAAAAAAAATAGTTTAACATTTTCAAATAAAAATGAAAGTGCAGGA
TACACATGAGAGTACAGCATTCAAATCCCAATACATCATTTTATGTGGCCCACAGTCCTCTTGGGCCTTGGCAATTATTTCCCCCTAAATTTCAACAGTG
CGTCGTATACAAGAAAAATTAATTTGTTATTACAAGTAGAACGATAAATGCATGTATGTACTAATCCTACTACATGAGAATGAGACTTTTTCATATAGCG
TAGGATAATTTTAATTCAAAACTACTTAAATATTGGGGAGAGTAAGTTATTGTCAATGAAGGGGTTTTTAAATAGAGTAAGTTACTTTTTTCTTTAATTA
AATCGAAATCTTAGCTAATTTCATTCATAATGATAGAAACAGTACATGAAGGAATTTGATAAATAAAAAAAATAAAAGCATGGTTGTTAGCATGAACCAT
GATGCTCCTGGCAGATGATGAGTAACATGATTGGTTTGTCGCTTTAATTTACAAAATTCTCTTCTGAGTTTGGAAAAAACTTTAGACAATCTTTATATTT
ATCTAAGAAATTTTTTAAACCAAAGCTGTTTTTACAATCAAGAATATGTCTCGATCGTCTCTAATACAGATGCCTACACCAAAACCTTGATGTTTTGGTT
GAATAATGTTGCGTTGATGTTGCATTTTACTCACCTCGCTTTGGTTTGAACCAATGATTGAGAGTTGTGTTCTCACCACCTTCAAGAATATAGAGTAAGT
TATTGATAACAATAACAACTTATTACTTTAAATATAAAATAAGTGTAGTAAAAACAGAAATGATAAAATAAATAAATAATATTAATTACTATTATATTTT
ATTTTCCTTAAAAAATTATAAATGCACCTGACATATATATGATTTATCTTTAATTCATTTTTAAAAAGTTGAAATTTATTTTAGTTTCTTATTTTCTTCT
TACATAAATACTTATAAAAAAAGTTATCCAAACATAATGTAAGCCTTAATTATTTCTGCTGAGTTGTCAGTACTTAATTCCCTCCATCCATCCATCGGCC
CTTGTCTATCCAAACATAATGTAAGCCTTAATTATAAAAAAAGTTATCCAAACATAATGTAAGCCTTAATATAAGCCTTAATTATTTTCTTCTTAATTAT
TTTTTTAAAGATAGAATAATAATAATTAATTTCCCTTGTGATTGGCTGAAAACAGTTATAGGTACTTACTATGATTTACTTCTAATTAATAGATGAAGTG
GATTTTTTTATACATCAATAGATAATGTGATTATTTTAGTTTTTAATGAACTTTTAAGAGTTTTACATTATCCTATGTAATAAAAAAAAGTAGATAACAA
AAAATAATTATAAAAATCATATATGGTAAGTTAGAAAAATATTAGCCACATTTTCTTTTCTTATTAATATCAATTAGTTGAAATTTATTGAAAACCACAA
ATTTAATAAATACTACATCTCATTAAATGATTTACTTTGGTGGTTTAGAGGAGAGATTCACCATAATTTATTGAAATGTTTACACATTAAAATTTCTTTT
TATCATAATTATAATTATTATTATTATGAGCACATTGTATTTATTTACTTGTACTTTATTTTTGTAGGGAAATTTGCTCTGTGGATGATACCTCAGTTGT
TTGCATACGCTATAAACTTTCCAATGGTTAAGTTCCTACAGGCACAGAGGAAAGTGCTGGTGATGTTATGGATATCAGTAGTTGTTTTGGTGCTGCACAC
ATTTTTTAGTTGGTTTCTTATATTTAAGCTTGGATGGGGCCTAATTGGAGCGGCAATTACTCTCAACACATCATGGTGGGTCATTGTGATTGCACAATTG
TTGTACATTTTCATCACTAAGTCAGATGGAGCTTGGAATGGGTTTACATGGCTAGCATTTTCGGACTTGTTTGGTTTTGTAAAGCTTTCTCTGGCTTCAG
CTGTTATGTTATGGTATTTTTCTCTAGCTAACTTCATTTTGCATTGGACTTGATTATATATATTTATATTTGCTGAAATTAAATATGGTGTTAAATGTGA
ACTCTTGCAGCTTGGAGTTTTGGTATTTGATGATACTGGTGGTGATAACAGGGCGTTTGAAAAATCCTCTAGTACCAGTTGATGCCATATCTATCTGGTA
AAAATTTCTCTACCCTCCCTCTTCAGTCCCTCCAAATTCACTTTGTGTGGAGAAGTTGAAACTTGAAAATATAATAGACTAAAAGTTTTTTTTACCCTTT
AGTTAGTTCCTATTTAAATTTTATTTGTTTTTAGTCTTCAAAGATTACAAAAATGTTGTGTTTTGAGTCCTTGATCAGAGATTAAAAATGCATCGTTTTT
TTTATTCAATATAAAGATTAAAAAATAAACAAGGACTAAAACAAATGAAGAAAATGACAAACATTCATAATTTCATATAGCAAAAATATAGAATAAATGA
TAAATTTAATAATTTCATATAGCAAAAATATAGAATAAATGATAAATTTAATTTTTGATATTGTGATTTGTTGTTACTGTAGTCTTCAAAATTAACCAAA
CTAAAAAAAGTCTCAAACTATTATATGTCACTTAGTCTAAAGTTACATGTACCAAACGATTAAGTCTAATATCAGAGACTTTTTTAATAATCAAAATGAT
GATTAAATTAAGCAACTTAAACAAAAATTAGGGGCCAAAATGCTGGTTAAAATTAGGAATTTAAATGGAACATTATAATTTCAGTTTTTTTTTTTAAATT
TTGTTAAATCAAGGACTAACTTTTAATTTAGTGGGCCATGAGAAGGTGATATTCACTTTCTAGTTTGGGCTTATGGGCATGTCACTCATCTACAGTTTTG
CTGTGCTTGCAAGTTGCAATTTCATAACCGAGCTAGTGAATTGCAATGCTTTGCTTAGTCAATGCCAACATATCATTCCAAGTGCCACTATATTGAGTGA
ACATAACAAAATCTAAGATCAAAATCGATTACTAATACTTGTTTCCATGTTTGCAGTATGAACATAAACGGTTGGGATGCCATGATTGCAATTGGGTTCA
ATGCTGCTATAAGGTGACCATCTTTCTGTCTGTTCCTTTTCACAATACAGTTCTAGTCACTACATATATAGAAAACCCCTATGTTCCTATAAGCTAATGA
ATAGTTGATTTTGAAATTTGGATTGATTTTCCATGAATCACATTGGGACATAATTTCTCACTAATCATGTGGGGACATTATATAGGGGTGACTAATGGAA
TATTTCCAAGTAGTAGTAATGATACTTTTACACGCCCTTCGGTTTAAAGGAAAGAAAAAGATGAGAAAGTAAATTGAGAAGAAAAAAAAAAGGATTAAAT
TATTCATTTGGTTTCTAATTCTTACTTTTTTAATTCCTAGTTTGAAAATAATGTTTTTATTTTTTATTGTTTACATTTTAATTCTCTTTTAGTCTATGTA
ATTTAAAAATGTTCTTTTTAATCCTTATAGATTGTATTTTTATTCTCTTTTAGTCCTTTTAATTTGAAAGTGATATTTTTAGTTCATATAATTTGTATTT
TAATTACCTTTCAATCCTTACTACAAAAAAAATATAAAAATAATTAATTACAAATTAATATTTTTTTATTACAAATTATCTTACGATAAATTAGTTACAA
ATTACTTGTTAATATTTTTTTAGTTAATTGTAATTGATAATATTACTCATATTTTGATGTTAAGGACTAAAATAAAATTAAAATATAAATAATAAGGATT
AAAAAGACCACTTTCAAACTATAGAGAGTAAAACAAAATTAAAATACAAATTATAAGAATTAATTTTAAAATTATAGGGACTAAAAAAGAATTAAAATAT
AAACTATAATAACTAAAAAATTATTTTCAAACTATAAGGATAAAAAAATAAAAATTATGAAACTATAAAAACAAAATAAGTTAAAAAAAAATATTTCAAT
TAAAATAGAATGAAAAGGTATGAGAATACACATATTTTTAAAACTTCATCTCAAATATACTCAAGAGTAATTAAACCAAATACTATGTTAATTTATGGAC
AGTGTGAGAGTATCAAATGAGCTGGGGGCTGGTGATTTCAAGGCTGCAAAGTTTTCAGTGTGGGTGGTTTCCATCACATCAGTGTTCATAGGTGTTGTTG
CCATGATCGGAGTGCTATCAACCAAGGATTACTTTCCCTACTTGTTTACCACCAGCGTTCCTGTTGCCAATGAAACAACTAGGCTTGCTGCTTTGCTCGG
TGTCACAGTGCTTCTAAACAGCCTTCAACCAGTCCTATCAGGTTAGTTCCACAAACTATGAACACAAGCAAAGTACATTATCCAATCGAGGATTGTATTG
TTTTTTATAAGTTAAATGCAATTTTTTAAGCACTCTTAGTGTTGTTTTTAAGGAGAAAACTTACATGCAATTTTATAGGTGATTGTATATATAAAAGTTA
TAAGATTGATTTGATGGTGAAAGAAAAAAAGAAAATTGTGGATTTGTATATGTTATTGATATTATTCTTATATTAGAATTGACGTTTCATCTAATTAATT
TGCATAATAAAAAATTTCATTGAATGTTAATGTAGATTATCTAAAGAAAACTTGAGTTATGATTGAGGAACAATACTAACCATACTTATAGAACTGCAAT
TAAGTTTGTCTTGTTTCTGACTAGAATTAGAATTTCGTCTCAATAACCTATGTGGATCTTTAATACAAACTTTTATTACATAGATTAATTAGTTGAAACT
TCAATTTTAATTATAAAACAAGAATAAAAATATGTTAAGAATTACATTTAAATTCTCTTTTTTTTTCTTTCTATTTTATGCCATTGTGGTCTCTGGTTTT
AGGTGTTGCTGTTGGGGCTGGCTGGCAATCTCTAGTAGCATGCATCAACATTGTGTGCTACTATGTTATTGGATTACCGGCAGGGATAATATTGGGATTT
CCACTTGGTCTTGGAGCAGAGGTGATGTACCATCACTACCTTCTCTTGAAATTATATACCAACAGATTTGAAATAAAGTACAACAATATATTAGAAGGGA
TTCATAATCAATAGCATTATCAAACTTAATTGTTTGTTAGTGACGATAAGTTTGCAATTTATTATTGTACTTTTTATTCTTACTCTTTATGCCTGTGTAA
TTCAGGGTATTTGGTCAGGGATGATTGCTGGCATAGTTTTGCAGACTACAATCCTTATAATCGTCACTTCAATACGCAACTGGAAAAAAGAGGTTGGTAC
TTTCCACATTTATTGTTTTCTTTCTATTTTATTTAATTCTGCTTTAAGGAAGATCCAAAAGTAAATGTTTCGGTTGTGGGTCCGAGGGAAGCAAACATTA
ACTAGATTTATATGAATGGAACATTAGCAAAACCTTAATATTTATATTTATGAGTATTTTATTTATTTATATGATGTCTTGTCACACTTGCATTTCAACA
TATTTTAATTTAGAGTTAGGGTGATGGGAATTACCAATTTTCTTTTTTACAATTTACAAATAAGTAACCTGTTTTTAGTTCAAATTTAATTTGTTGCCGA
TGGATTTCACAGGCTGAAGAAGCAGAGAGCCGCGTGAAAAAGTGGGGAGGAGCAATTGCATATGATCAATGATGACTTTTTATCATAGTTGAGAATTAGG
CAGAGATTCTATTAAGTTGTATCAATAATGATTGAAAATTTTTAACATGTATTTGAATTTTAGATTATAGGTCAGCTTGTTATGATTTCTACATAGATAA
TTCATATGATTGAGCACTGATTAAACAAGTGTTAAATTAAGTTATTTAAACAAAAGAATCTCAATTATAAA

>Glyma.04G097900 | Chr04:8857667..8865249 forward
CAGCAGTGTTGTTGAAGGGATTGAAGAGTAAACATAGGATCCACGATCAACTCGATTTTATACTTTTGTTTCTATTTCTCTGGAGGCAGCTAGCTAGGGT
CATACTCATGGGGAGCTTGAAAGGAGACAGTGGACATGCCAATGGCGAAAATCTTACCGAAGCTCTTTTGCCAACACGAGATGCACAACAACAACATCAA
ACAGACGACGAGGAACAAAGATTTGGTGACAAGCTCTGGCTTGAAACCAGGAAGTTATGGCTCATTGTGGGGCCCTCTATATTCAGCCGCCTCGCGTCGT
TCACCATGAATGTTGTCACCCAAGCCTTCGCCGGTCACTTGGGTGATGTCGAACTCGCTGCCATTTCTATCGCCAACAACGTTCTTGTTGGCTTCAATTT
CGGCCTCCTTGTAAGTCTCTTTCTTTTCCCTCACTAACAAAAACTCCATCTCATGCTAATTCATGGTTAGAATTGGTTTAGTTTAAATATATAATTGCGA
TAAATAATAAAAGAGAAAATGTTTGTCTCTTATAACAACATATTTTTATGAGCGTTAAGGAATATATTTTCTGATACTCTCTTAAAATTTATCAAAAAAT
ACAACTTGAAGAAAGGCTAATCGACATTTTTATTATTAAAAATAAATAATTTGTAATTCTTGAATGCAAAAAGAAAAGTAATCTTGTGTGGTAATAGTTG
GGGATGGCGAGCGCGTTGGAGACACTGTGCGGGCAAGCGTTTGGGGCGAAGAGATACCACTTGTTGGGGATATACATGCAGAGGTCATGGATTGTTCTGT
TCATGTGCTGTTTCCTGCTGTTGCCGTTTTATGTTTTTGCAACGCCGCTTCTGAAATTCCTAGGACAGCCAGATGACGTGGCAGAGTGGAGCGGGGTGGT
GGCAGTGTGGCTCATTCCCCTGCACTTCAGTTTCGCGTTTCAGTTTCCTATGCAGAGATTCCTACAGTGCCAGCTCAAGACAGCAGTTATTGCTTGGGTT
TCTCTGCTGGGTTTGGTGGTTAATGTGGTCACAAGCTGGCTCTTCATTTATGTCTGGGATTTTGGACTTTATGGCGCAGCTATTTCTTTGGACATTTCTT
GGTGGGTTTTGGTTTTTGGCATGTATGCTTACATTGCCTATGGTGGCTGCCCTTTGACTTGGAATGGTTTCTCCCTTGAAGCTTTTTCTGGACTCTGGGA
ATTCCTCACACTCTCTTCCGCTTCTGGGGTCATGTTATGGTATTATCATTTTTCATTTTTTACTACTATTATTTTGTTTTTTTTTCTCTCTCGTCATATT
TGTGTATAGTATTTATTAGTTTTATTAATAATTCATCTTTAATAAAAAAATCCAATTAATTATTTTTAGTAAATAAATTTTCTTTAATTTCATTTTTTTC
AATTGAATTCTAAATCTTACTTAATAGAAATAAATTTCAATACCACTTGTACTTGTTAGTCTACTATTGTTATTTATTAACAAAATTCTAACTTATCTCG
AATGAAAATGAAACTAGACAATCAATTTTATACATAATTGGTGCAATTAATGAGAAGGACATTTGGGTACTTGGTTGGTGACACTTGAATCTTATACGTT
GATGCAGCTTAGAGAACTGGTACTACAAGATATTGCTACTTATGACTGGCCAGTTGGAGAATGCCACTATCGCTGTCGATGCCTTGTCTGTATGGTAAGT
TATTTTACATCCTTTTTTTTCTGTATTTTACAGCCGTTAATTATCATGAGTTTTTTTTTCTCATTTTAATATAAATTTTTAAAATTGATTATTTTTTATA
TTTCTAAAATATTTAATCTATTCACTTTTTTGTTTTATACTTTCAAATTTCAATCCTTGACATCATTTTAAAAAATTATTCTAAAGACTATAAAAATTTA
TTCTAAAGGTTATTCCGCACTTTTTATGATCTTTAACTGGTTAGTTGCATTGTATTTGGTTTTGCAGTATGACCATCAACGGGTGGGAAATGATGATTCC
CCTGGCTTTCTTCGCGGGTACTGGGTACGTTGTTGTTTTTCTTTTTTCCTCTTTTAATATTCTTCATTTTATTTATTTTAAGGAAATATATATCCTTTGT
TTACTGTTTGAATAAAGAGTTTAAAATTCATGTACCGAAAGTGTAAAGAAATTATAAATTAGCTAATTACAAATCATATTAGAAATGACTTTTAAATTAG
TTATTATTAAAAAACAACAACAAATTAGTTACTATATATGATTATTTCTATATTTATTAATTATGTAAAACTCTTCACAGTGTATGTATTGTTTTCTTTT
AAATACTAAACCATCCTTTTCCACTTTCTTCCTTGAACTCTTTGCCTTTTATTGTAGATTAGATTAAAATTAATTCTACTAAAGAGTAAAAGTGTAATAT
TACCTGTGGGCTGTGGATAAATCATGCCCCCAAGGCCCCAACTGGCATTATGCTTATCTTATCTCGCCAGGCGTAAATTTTCCTTCCATTAAAAAATATT
CTTTAATTATTGCTAGTCACAATTATTTACATTCCTGGTACTTTTTTAGTTCTTTTCTCCTTATTGTGCTCGATTGAAAATATCATTTTCTAAATTTATC
AGTAAAAAGGTAAATTTTTTTTTTAATTTAAAAATTATACCTTTTAACTAAATAATACTTTTATTTTGTTAATATTATATTTTATTTAATAAAAATATCA
ATAATTTAAAATAAATTTTAAAATAATTGATGTCTTTTTTGCTATTTAAAGTTGACAACAAATGAGACATGTTTTAAAAAATGGGTAATACTTTAAAAGG
AATGAATAATATTTTCACATATTATTGAATAATATTTTTTGGACATCAAATATTAAGTTTTCTTTAACAATAAACAAGTTGCATTATTAGAGAAAAAGAA
ACAAAGAATCAGGAGTAATAACAATAAATTATAGATGTGATACAAAAATAAAAACACAAAGAGAATAAAAAAATTTGATTGTTGATGATGTATGCTATAA
TTCAGATATTTAGTTAGAAGTGAGTTACTGACTCATTTTGAAGTTAGTTTGACCGTCATGAAATTTGAAATTTGTTATAATCATATATGCATCGTGTATA
AACATAATAGTCATAATTAATGAGAAGAATGAGAGTGTAAAAATTTTAATTTAGAATTTTAAGTCCTCTTTATTTTCTCTTAATCAATTCTTTCATTATT
TTTTTTTTATTTTCTAACGTAGAGTGAATGAATGTATAATGTGAATGTGTGTGAGAACTGTATTATTCCAACAGAAATCTTATGTATTCTTGTATTGTTG
TTGATATATAAAGAATTTAGGAGTAAAAGTTTGGTGAAAAAATAATTTTCCTTCTGGGTATCAAATAATTAATGACTTTGAGATTCTTCAACATGTAGCT
TTAGAACATCATCTTTATCTTTAATTTCTTTTAGAATTCTTTATCTTTTTTTTTTTTCTTTTCTCCTCTTTTTGGGCATGAATTTTGAATTATATCACGT
GGGCAAGTAATTTACCACTCTTTTATATCTGTTCTTGTTGATAACGTTCCAATGTAAATGCTACTCGTGGACGGCATGAACCTTTCTATCCCGAGAAAAC
GCTTTGTGTGATCATATTCATTTCCTATAGGCTATTATTGGACTTGATTGAATAAAAAGAAATAAAGTAAAACGACATAAATTTTTCTTCTGCAAACAAT
CACTCGGGAATGGGGGATTGATTCGATTGAACCCACAAAAAGAAAAAAGTCTTGTTTGAATTTTTTAAAATGAAACAAAACAAATTAAATGAAATTGATT
TAATAAAAGAAAAACTTTACTTCAGACTGTACTTTATTTTTCTTCATCTTAATTTAAATTAATATGATTTAGAACTAATTTATAACATAGGATAAACTAT
TTTTTTATATTTAACTAGGATAAATAGTATAAATTATTTCATACTACATTATTTCTCACTTTTACAGATATTATTAGCGAGATATTAGGCGGCAGTATCA
TGATTAAATGGTTCTCCTGTCTTCTCAGATTGAAATTGGATACGGTCAAAAGAGAAAGATAAGGACAAAGAAGAAGAAAACATCTTCAAACTATTGAATT
GACCTCTATTTTTCCAAGACGTTCTTTTGCGTTCTCATTGCTCAAAATGATATTGCATACATACGAACTGTAGATAGAGAATCCTGATCCTCTTTCCAAC
CTTAACTGTTGCATTGGAGTACTTTTTAAATGAAAGTTAATTATCGACAGTCCCAATTTTATAGTAGAACAAGTCCTTTCCCTAAACTTATTCTAAAAAA
AATAGTCATGTTTAATTAACGAAAGCATGATTATGTTAATTACAGGGTAAGGGTGGCGAACGAGTTAGGCGCAGGAAATGGCAAAGCAGCAAAATTTGCA
ACGCAAGTATCAGTGGCACAATCAACAATCATTGGGCTTATATTTTGCGTTCTGATAATGATATTCCATGAGCATATAGCATACATATTCACCACCAGCA
CTTCCGTCCTACAAGCCGTTGACAACATGTCATTACTCTTAGCTATCACCATTCTTCTCAACAGTGTACAACCCGTTTTGTCAGGTACGTATACAAATTA
TATTAGGATTAATTAAGTTTTTAGTTTTTTTATAGGGATTTTAAAATATTTGATCCTTAAATATTTTTTTATTATTATAAATAGTTTTTATAGTAAATAA
TTGAACTGTTTATTTAAATGATTACTAATTACAAAAATTGTAGATAAAAATAATGGATTGTTGCTTGCTTTGCAACGTAGATTTGCAACGTAGATTTTCT
TATGGGTGATCATGAATACTTGAAACGTTAGGGTTCAACAATGACAATTTCTCTTTTATATTTTAATAACATAATATTATAATTAGGTTTAATTACGTTT
TTATAGTTTTATCATTTGTATCTTTTAGTCTCTATAGTTTTAAAGTGATTTTTTTAGTCCTTATAATTTATATATTAATTCTCTTTTAGTCTCTGTGAAA
GTGATTTTCTTAGTTCTTATCATTTACATTTTAATTCTCTTTTAGTCCTTTTAGTTTGAAAGTGATATTTTTAATTTTTATATTTTATATTTTAATTTTT
TTTAGTCTTAATCATCAAAGTATGAGTAATATTATCAATTACAATTAAATACAAAATAATAGAAAGTAATTCGTAACTAATTTATCGGAAGATAATTTAT
AATAAAAAAATTTGATAATTTATAATTAATTTTTTTATATATTTTTTTGTAATAATGATTAAAAGATAATTAAAATATAAATCATAGGAACTAGAAAGAT
CACTTTCAAACTATAAGGACTAAAGGAGAATTAAAATACAAATCATAAGAACTAAACAAATCACTTTCAAATTATAGGGACTAAAAGAGAATTAAAATGT
AAATTTTAGAAACTAAAAAAATCATTTTCAAACGATAGAAACTAAAAAGTACGAATCGTGAAACTATAGGAACCAAAAGAATAATTTAACCTTATAATTA
TTTACCTAAATATAATAAAACTTATGTGTCACGTATATGGTATACATGTGTCATTTAATTTGTTTTGTTTTATGACATTTTATCATTTAACAAAAAAACA
TTAACGACAGAGATGATTTAATAACAACAAATGCTTTAGGGACCATTTTTTTTAAAAATAATTTAGGGATGAAAAATTGAAAATTCCTTACAACTAAAAA
CTTAATTAAGCCATTATATTATTATGTAATAATAATAACATAACATATAATATCTCAGTCTCACTATATGCATGGATCTAACTTCTAAGGTCCACCAAGA
AAGTGGAAGAAGGAATCTAAATTTCCAAAGTTTGCGCAGCCAACGCAACCTATTAAAATATTACTGGTAATTTTAGAAGATGCATATGCATGCAAGTTTG
GAACTTACATGTGGGGTTTCAACATTTTTTATGCTATATAATTGTTTATATAGTTAAATTTTAATTTATTAGTTCTAGTTATGTAAAATTTTCATAATAT
TACTGATAACCGGATTCTGTGTATGAGAAACACTTCAAAATATCCTTTCTAATAAATATTATCTCATTACTTTGGAAATGTATAATAATTCACGATACAT
ATATATTAGGTAGCATTTTAAAAAAAATATATTTGAGTTTATTTTATTGTTGTTTTTTAAGTGTTGTCATACCAGTTAATAAATCATAATGCATGATTTA
AATGTTAACACATAGTTAAATTATAATACATTTTAATTTTTTTTACATTGTCAATGTATATATTAAATTAAATTTACTTTGATTATATAAAGAAATTTGG
TGATGCATCATGCATGCCTTATTTTTGATACAAATACGAAAGCTTTTTGTGTAATGCATATCCTACATTGATAAGTAGTTTGTTATCTTGAGGTATTGAA
AGATATAGTTGCCATCATGGTATTGATAATATTTGATATAATATTATAGGAGTGGCTGTTGGATCCGGATGGCAAGCATACGTTGCATATATAAATATAG
GATGCTACTACCTTATCGGGTTCCCACTCGGAATTATAATGGGATGGGTCTTCAAAAGTGGTGTAATTGTACGTATAGTTTACTACTTAACTATCACATC
TAGACACTAATTTTGTGTTTGAAAAGTTTTTAAGTGGTGTGCTAACAATATGGGAATGATCATGCATGGCAGGGCATTTGGGGGGGCATGATTTTTGGTG
GCACAGCAATTCAAACGCTGATACTGATCATAGTAACGATACGATGTGATTGGGAAAAGGAGGTATAGTTTTACTTTTACATGATAAACAAGCTATAGCT
AATTCCAATTCGTTAGCACTTAGCATATTGACAAAATTGAAAGATCTCACGATAAATTATATACCCATATCAATATATAGCGTATATATGCATGTTCTAA
TATTTTGTTTTAATGGCATTTTGCAGGAGGAGAAAGCTTGCTTTCGTGTAAGTAAGTGGTCAAAATCTAACTCCAATGGCAATCTGGAAGCCTCCAATTA
ACATTTGATTTAGCCAATGGATCGTGTTTGTTATTCACCAATCTGGATTCTATGAGTTTTGCCGTTGTATTGGATGCTGCATATATATATATTTCTTTCA
ATATTCACCAAGTTGTTGTTGTATTCAAAATTAAAGGCCCGGTTATTCTTTAGGATAGCTATGTTATATTACCATGAGTTGAATTTGGAGATCCCCACGC
ACCGACAATACAGTTGTAATTCCAACCTATGGTGTTACTACATATTCTTTAGTAAGACTAATTAATTAGATGTTTTAATCATATTTTAGCACAGAAAAAG
TTCAATTAATCATATTTTTCAATTTTTATTGCAGGATTGTGGAGACGGATAAGATGAGAGAAAAGTGATGAAGCTCGTGATTTACCCTGTCATCATTAAT
TTCTTTGATTCCTTGTTTTCTTATCTTTTATTGCTGGCTAACAACTAGCTGGACAAGGTTTCTTTGTTATTAAAGTGAGTGGATCATTTTTTTTTTCTTT
AAGTGTTGCGTGGATGGAAATTAGTTGAGACAGGAAATAAGTTCTATACATAATACTATAACAGTAACGGAGTGATTATGTGC

>Glyma.04G097700 | Chr04:8838031..8842355 forward
ATGGATACCAGTTGTCAACAAAAGGGATTTTTTACTTTTCTCCCTCAAAATATCACTAAAGCACCGCATTATTGTTGTCCAGTAATCGACAACCTGCGAA
GCGAAGCAAAACACATGGGAAATATTCTGAATGAAGGAGAGCATGAGCATCCTCTGATAAAGTCAAAGCTGCCACCACAACCACACGGATCCAACAATCA
CTCTCTCTTCCAACGATCATGTTCCGAATCCAAGAAGCTATGGCACATCGCTGCCCCCTCCATCTTCACCCGCCTCGCCATGTTCTCCATCACCGTCGTC
ACCCAGTCCTTAGCCGGCCACCTCGGCGATCTCGACCTCGCCGCCATTTCCATCGCCTGCACCGTCCTCATCTCCATCACCTTCGGTTTCTTGGTAACAC
TTTCCACTTACCACTTTTCATTCACTATTTCCCACTTTTCATTCACCGCATTATTGTTGTTAGTCGTTAGTTAGTTAATTTTTGTTAACGAACTTATTCC
TTTTTTAGTTGGGTATGGCGAGTGCGCTTGAGACACTATGCGGGCAGGCGTACGGCGCCGGACAACAGCGCATATTGGGCGTTTATTTGCAACGCTCGTG
GGTTGTTCTGTTCCTGTCTTCCATTCTGTTGTTGCCGGTGTTCATTTTTGCGACGCCGGTGTTGAAGCTAATAGGGCAGCCCGTCGCGGTGGCGGAGCAG
GCGGGGCTGGTGGCGGTTTGGTTGATCCCATTGCACCTGAGCTTCCCGTTCCAGTTCACTTTGCAGCGGTTCCTGCAGTGCCAGTTGAAGACCGGCATCA
TTGCTTGGGTTTCAGGGGTGGCTCTTGCGGTTCACGTGTTAGTGAGTTGGGTTTTTGTTTATAGGATGAGAATTGGGATTGTTGGCACTGCTCTTTCCAT
TGGTTTCTCGTGGTGGCTCTCTGTTTTGGGAATGCTCGGGTATACACTCTTTGGTGGTTGCCCTCGCTCGTGGACTGGTTTTTCTGTTGAAGCTTTTGTT
GGCCTTTGGGAATTCTTCAAACTTTCTTTGGCTTCTGGGGTCATGCTTGCGTTAGTCTTCTTTCTATCTTGCACTGTAGTATTGTTTGGATAAACTTGTC
CACGAGTACTAGGAAAAGAAAAGATAAAATGAAATGAATTATTTTTTTTGTTCAAGTTAAAATTAACTTGTGCAGTTCAACTCTTATAGAAGCTCTCTTA
TTTAACTTTCAAAAACTAAGCTGAATGCCTTTTATTTTCTTATCTAAATGTTCATTAAGAAAAATGTGTAAAATGGATCTTGCTAAGGAGTTGGGACTTT
TAATTTGATATTATGTGTAATTAAGGTTTGCATTAAAGGATTATGCAGATTATGGAGGTAAATCCGCAATTTTATTTTGAGAACAATACAAACACCTAGA
GAACTATGTATAAGATTTGTCCCTTACTTAGTTCTTAACTTTGAGATTGATGCGATTCTTAGTGTGTTAATGTGGCATTGCAGGTTAGAGAATTTCTATT
ACAGACTGTTGCTTATAGTTTCTGGATATATGCATAACACTGAGATTGCAATTGATGCTCTTTCCGTTTGGTAAATTTGTGCTGTGTTGGTTTTTTTCTT
TCTAATTTAGTTGTATCTTGGTTTATTGGGTTTTTGCTGAATTAAAGTCTTACCTGGCATGGCAGTGTGACTATTTATGGTTGGGAGTCCATGATACCAC
TAGCATTTTTGGGCGCAACCGGGTATGTCACTGGTTGTAAGTCTCTCTTTCTTTCTCTCCCACTATGTGGTATTATAGATTTGTTGTTTAACTATTGACA
AGTTGTTTAGCTTGGGGGTGTGGTGAGGTTGAGAATTCTGTGGTGTACTACTCAAATGGAGTCAATGTTCAGAACTGCTGTGAATCACAAGAAAATGCAG
TTGGAGATAATTGTATGTATCTGTGGTGGTGTGAGGGAACAAGGAGCGAGTTTTGTGGTAGTTGAATCATTGGACACAGTTTATATTTTGTCTACTCTGC
TATTTCTCTCTACTTAGTTGCTGACATGCTTTTGTCAAATGATGGTGATAGTTGTTACAAACTAATGAGGCGATTCATGAGATATTGATTTATCATTTAA
TTTGGTGTGAATTTTCCAGAGTGGCATTGTTTTCCTTCCCCTAAATTTTATGCAAGACCTATCATATTTAATGATTAATTCTGCTTGCTGTAAACCTGTG
ATTAAGTCTCCAAAAAGTAAGAATTACTCCAAAACATTCAGTCAGTTTGTCAACTCGAGTATATAATTTGCAGTAGTCTTGCAACAACACTTGCATTAGT
TGTTCCCTTGGAGTTTATCGTTATATCAACATACACCTTTTATTGAAAAACTGAATCATTTCCATGTTTGAGAAGTTACATAGGTCACTTGATTTAGCCT
CTAGAAAAGTAAGGAAGCTGATACTGAAGGTCGAGAACCACAGATTGACAGAAATCTACCTTCTTTCCTATTAGTTCATATGGAACTTCTAAAAATCTAC
TGATTTTATTGGAGACATTGGTCAAGGTCAAGGGAAACTGTAAAACTATGTGCTGTTATTCTCTGATGCAACATCAGCCAGTCTTGGACCTCTTGGTAGT
GCTTGTATTACAAGGCCTGTAGATACATGTAGATGTAGCTTCATAACAGCCAAACTGTGTCTGAAAGTCTGCAAATAATCTCAAACATATTTCTTTATAG
TAAAAAACTAATCTGTTTGATGTAAAGAACTGTGTCTCAGTACAAAACCAACTAATTTTTCCTCCCTTATAATCATCTAACTTCCTTCAACTCAGGGTGC
GTGTAGCAAATGAGCTTGGTGCAGGCAATGCAAAAGGTGCAAGATTTGCAACTGTGGTTTCTGTGGTAACTACTTTATTTGTGGGATTTATATTTTGGTT
GGTAATTGTGTCCTTCAACAAGAATCTTGCCTTGATCTTTACATCGAGCTCTTCCGTTATCCAAATGGTCAATGAGCTGGCAATGTTGTTAGCTTTTACT
GTTCTTCTTAATTGCATTCAACCAGTTCTTTCAGGTGAGTATCTAATAATACACTTTAGAAGCACTCAACTCAAATTTCAATTTCTGAGGTTGTCCTTAG
TGCTGGTATCTGTTGGTCTTCAGGGGTGGCAGTTGGGTCTGGTCGTCAAGCAGTAGTGGCTTATATAAACATTGGCAGTTACTACTTGATTGGAATACCT
CTTGGGGTTCTCCTCGGCTGGTTGCTTCCTTCGGGTATTGTAAGTGTACCTATCCTATAATGATGCAGACACAACTTAAATATTCCAATCCACTCTTACA
TAAGTTACAATTAAAGGTATGTTTGGATAAACTTTTTCACAAACACTTATAGAAAAAGAAAATAAGATGGTAAAATGAATTTAGCCTTTCCCATAAGCTA
ACACTAACTTATGCATCATAGTTTTTTAAGTTAAATGAAGGACTTCTATAAAAGTTAAGTATGATAAGTTGATTTTAGTTTATGGGAGAAGCTTAATTTA
TTTTATCTTATTTTTTTCTCCTATAAGTATCTGTAGAAAAGTTTACCTAAAGAAAGCCTAATTTTACGTGATCATATGCCAATTATGGACCAGCTAATGT
AAATTAGGCTAACCTTGCCATGTAAATGTGAACTGTTCAGAGTGCTCAATTGTGAAGGCAATACTTTATGCAGTGATGGTTTATTTATTTTACATGCAGG
GTATGTGGACTGGAATGATGAGTGGAACAGTGGTTCAAACATTGATCTTGGCTATTATTACGATGAGATATGATTGGGAAAAAGAGGTATGCTTTACAAA
GCGTAGCGTTTTGGCACGGAATGGAACGGTTTTGGTTTCATTTTCATCCAATAAAATTAAATTATATAATGCACTCGGTTTAGTTCTTTTCTTTACTTAA
AAAATAAACTGTCTATTACCTGTCCATTAAATAGCTAATTTGAAGTCAAATAGATTGTTTTCGTTTCTCTGATAAGTGATTGCAGAAACTTGTAACAGCC
ATCTGGTTACTTCGTTCGTAGGTACAGAAAGCCCAAATTCTTGTTGAAGAGGAAGCAACATTCAACGACCAATAACAACGGTGAAATTTGTATTTAATGA
TTAAGGTAATTTTATTCTTTGCTGCCTAAAAAAGACCACATCAATCACAGAATTGTGGTGCTTTACCATTAAATTTTTTGATCCGTCGAAGCATGTATTT
TTAACGGCTAAAACAGGATGTATATTTCACTAAGCAACTTACTACTCTTAAAGTTTGTTTTTTTTTTTTTTTGGTGAATACTCTTAAAGTTAGTTTTCTA
ACACAGGAAGCAACGGGAGCTTGTA

>Glyma.01G025200 | Chr01:2616281..2621186 forward
AAGAAAGATATGAATTTTATTGTGCATGTCATACCTTTTTTTGTTTGTTCATTTGAAAGAAACCAGACCATGCCACATCTGCTTTAAAAGCCTGTACTTT
TGAACCTCTCTCGTGATCACTCTTCTTCCTTTCTTTAAATTTGTTTCCTTCACACTTTTCAGAACACCCAGAAGCTCTAGCCTCACCAGCACCACATATT
GCTAGCTAGCGACTCCGAGATGCCAAGCAAAGAGGATTCACCTTTAGTAGAAGCAAAGCTTCCACTATTAGAAGCTCACCCTTTAACAGATGAAAGAGAA
CAAGACCAAAGTTTCCCACGAAGGTTTTGGCTTGAGTCAAAGAGGCTTTGGCACATAGTAGGCCCTTCAATTTTCAGCCGTATTGCATCCTACTCCATGT
TAGTCATAACACAAGCCTTTGCAGGCCACCTTGGTGACCTCGAACTCGCTGCCATCTCCATTGCCAATAATGTCGTCGTTGGCTTCGACTTCGGCCTCTT
GGTATAATATATAATACTATTAATCCCACTTAGCACAAAATTTAGACACAGTTACATAATGTGTTTTTAGCCATGTTCTCTAATTAGAACAATATCGAAA
ACTATTTAGCTAGGTTGAAGATTGCCCAGTACAATAACATGTATTTTAATTTAATGTTGTTCTCCGATTAGAATTGAAATTTCTAATCGTTAAATGTTAA
TACTAACTCGCATTTATCTTTTAATCAGTTGGGTATGGCAAGCGCTTTAGAAACGCTATGCGGGCAAGCTTTTGGAGCGAAGAAGTACTACATGTTAGGC
GTTTACATGCAGCGTTCATGGATCGTTCTTTTCATATGTTGCATCTTTCTTTTGCCTTTGTACTTGTTTGCCTCGCCGGTGTTGAAGTTGTTGGGGCAGC
CGGAGGAGTTGGCGGAGCTATCCGGCGCGGTGTCGATCTGGATGATACCGGTGCACTTTGCCTTTGCGTTTCAGTTCCCTCTGCAGAGGTTCTTGCAGTG
CCAGCTGAAGACCGCGCCCATAGCGTGGGTGTCTCTGGTGGCGCTCGTGGTGCATGTGTTTGTAAGTTGGTTGTTCGTGTTCAAGCTTCAGTTTGGTGTT
GTTGGTGCTGCTGCTACTATTAACTTCTCTTGGTGGGTCCTCACTTTGGGGCTTTTTGGCTACGTTGTTTGGGGTGGGTGCCCTCACACTTGGAGTGGTT
TCTCTGTTGAAGCGTTTTCTGGTCTTTGGGAGTTTCTGAAACTCTCTGCTGCTGCTGGAGTCATGCTATGGTAATCTCTATCTTATAATCTTTTTGAATA
CTCTTCAAAATCATAATCACTATCTCAAAATCACTTTTTTATTATGATTTTTTCAAAGTAGTTATGTGTAATTTTATAATTTATTTTGACTGATCATCTC
ATTATCGTTACCCTCAAACGTGTTATCTCAAAGTTTTTATTACTGTGAGTGCTTTTGTTTTTTTGTTTGTCAATTATTCTTGTCAATGTTAGGTGTGTTC
TATGATACACAAGAAATTATTATATGTTCTCAACTCCTATTCCCCAACTACCACGTGGTTAAATTTTATAATTCATGAGAAAATAAAAATAATACTAATA
CTTTGTTGTACTTAATAATTTCCCGTGTTGCATATAACATGTGTTGTTAATTCATGGGAGTTTTGAATTTTGCTTTTGCAGCTTGGAGAATTGGTATTAC
AAAATTCTGATTGTAATGACCGGGAATCTTGAGAACGCGGAGATCGCTGTGGATGCATTATCCATATGGTAATTTAGTTTAATGAGTAAACGAAATTTTC
ACTTTATGCCTAACATGATTAATTAGTGTCTTTTATTTTACTTTTTTGATCTTATAAAACTTTTAATTTAATGTTTGATTTTCTAAATGTCATTTGTTTA
ATCCTTTTTCAATTATTTCTTTTACTGACATTAATAGTGTAATGAATGAGATTTTACAATATTTTGTAAAATGAATGACCATCAATAGTTTTATTTGACA
GTATGACCATCAATAGTTTGGAGCTGATGATTCCTCTGGCTTTTTTTGCTGCAACAGGGTAATTTATCTATTGTTAACTTGATATTTTCCTTCTTTCTCC
CTCTTTTCTACTTTGCTCACTCTTTAGTTTGTTGTACAAGTCAAAAATGGAGGCCACGCCATAACCTTATATGTCTATCAATGAGGTCAGATCGATAGTC
TACAGTGTATAAAAAAAACCTGGAAACGAACACGTCCATACAAAATATTTAATCAATCATAATTCATAAATATAATTGATAAAAAATGTACGGCGAGATG
AGAACGTGGTTAGTTCATGAACTCTAGGGAACCCATCAAACATATGGCTGAGCTCAAAACATGGTGAATTATTAAACCGTGTAATAGCACATAAGTCATA
GGATAGAATAACACGACACAACAATGATTCTCTATCATAAAATATATAACCTTTGATTAAATTGCTGTGGCGATTGAGACTTTCGTACATTGTGAAGTCA
CACAAATAAACACGCAAATAAACACGACGTGGTCCTTTAATCCTCAATCATCATTCACCACCAAATTAATATGGATAACAAGAAACAAATAATTAAGAAA
ACAAATACATGTGGTTGCTAGCTATAAAAAAATATTTGGTCCTAGTCAAGTCAAACTCGTGCATTATTTTTATAGGTGAAGCTATTACTGTTTGTCGTCG
AGCTGGAAAGAGGGATCTTGTATATGTATGGTGGTGATCTATAAATTTGGATGTATATAGAGTCTAGACTGTAGTAATAAAGTATTAGTCATGCATCATG
GAACGCAATCTCACAAATATTCCTTGCTAGTTTGATTTCCTTAGTTTTTAATTAATCCTTCTTTCCCTAAGAATTTTTAATATTAATTAAATATTTGACA
TGGTCCTAGATGAGATAGCGACAACCTCTGTATACAGAGTCTTGATTGCTCCATATGGAGCCTGTGACCACGTTGCTCCAGTTGCCATATGTTGGATGAT
TCTACAATTATCTATCGGAAAACTAGAAAATTGTTTGATCACAAAAGTGTCCTACAATTGCTATCCACGGCCTTGCTTTTACAAATGCCATTTTTGTGAA
TATTTTAATTCTCATTCATTATTCCTCTAAAGTACTAGTGAGTGGAGGCAGTTAATTGATGGACCTAGAGTTACTTTCAAAGAATTAAAAATAGCAATTC
AAGAATACTGCACTCTTTTTTCTTCTTTTTTTTCAAATCACATTAGACCATCTTTAATTAACTGCTTCAGTTGTAGTGCGCTGTTTACTTTAATGATTGT
GGTTTAACTCCCATGAAATGATAAACGATGATCATTAATTTAACTAATCACTAACTCTCAAGATGCATGGATAGAGCAAGGGATGCCCTATTTGCCATAA
CCTAAGGGGAGAGAAAAGATGAAAAAAGTGAGATATAATTAATGAATAGTACTACTAGTAAAAGAAAAAAATAACAACTAATAAAACATTTTTTTTAATT
TATAATGTTATAAGAACTTGAAAAGGTACAAATACAAATACTAAAGTTGTTACTAAACATGCATTAATCTGCAGAGTGAGGGTTGCAAACGAGCTAGGAG
CTGGGAACGGAAAAGGAGCCAAGTTTGCTACTATGGTATCTGTGGTGACATCGGTTATAATAGGACTTTTCTTCTGGATGTTGATTTTGATATTGCACGA
TAAATTCGGCTACATATTCTCTAATAGCAAAGCTGTCCTTGATGAAGTAAACAACCTTTCTCTTCTATTAGCCTTCACAATTCTGCTCAACAGTGTTCAA
CCAGTTCTCTCGGGTATCACCTTACCCTTTAGTAGCTTTCATTTTTCAAATATGAGTAGTATCAACATCATACTCTTTAATACACCTTTTTAACACATTT
TTTTATTAGTTAAAATTTATGAAAAATTATAAAATTTAGTTAGACTAGTTAAATAAGAAGTAAAACTCATAAAATTTTGTAATTTTTAATAAATTTTAAC
TAATTAATAATAAAAAGTACGTTAAAAAAGTGTATTGAAAAATATGTGTTATTGTTAGAGTGATGTTCAAACATTTGATGTTAAATGACCACATATGTAC
TCCACCATATGCTATATATGTATTCCCTTTTGGATGATATGAACAGGGGTGGCAGTGGGATCAGGGTGGCAATCATATGTTGCATACATAAATTTGGGTT
GCTACTATATTATTGGGGTGCCTCTTGGAATTTTGATGGGTTGGGTCTTCAATCAAGGAGTTATGGTATGTTTTGTTACCCTCTTTGTCTTGTATCTAAA
AAAATTTGCAGTTATTTTGCCTAGAATGTGAATTTCAAATGCATTAAGACATGATTCTGACACTATTTGCTAATGAAATTGAACAAAGGGAATCTGGGCA
GGGATGATTTTTGGTGGCACAGCAACTCAGACATTAATATTGAGCCTCATTACAATTCGATGCGACTGGGACAAAGAGGTAACATCCTAGCTACTCCTCA
ACGTCAGTTTTTTTTTTCTTCTTTTTCTTACTTGTCTTAATGGTTCTGACTTTTAATAATCTAATCTTAATTGATGTATTTTGTTGGTTAAAAATTGTAG
GCTGAAAGAGCAAAATTGCATTTAACGAAGTGGACTGATCCAAAACAAGAATTGAACTAATCAAGGGATACTCGCTTTGTAACCTAAATGCCAAAGGAAT
CCGCATATTTGTTTTAGGCTTCGGTGATATTGAACGAAACTAAAATAATTATGATATATGTCATTATTAATACAATGAAATTGTTTCATGATAGTAGTAT
TAGAAAAGAAAATTCTATTTACCAATTTTCTCCAGTTAAGCTCCAGTGTTGTCTTTGATTCTCCCATCACTCCACAAGCATTTTATGAATACCCTTGATT
CTACTT

>Glyma.02G039500 | Chr02:3707201..3710252 reverse
ATGTCCAAGATGTCAAGCGATGAGGCTTTAGCATTAGAGGAAGTACACCACCCATTGTTGGAAGACTACTGCGCAGATGGAGAAGAAAAAGAATATTTTG
TTAGAAGGGTTTGGAATGAATCCAAGAAACTATGGAATATATCTGGCCCTGCAATTTTTAATCGTGTGGCCACTTTTTCCATGTTTGTGATCACTCAGGC
TTTTGCTGGTCACCTAGGCGACCTTGAGTTAGCTGCCACTTCCATTGCTATTAATGTTATTTTGGGGCTCGACTTTGGCATATTGGTAAACTTCAAATAT
TTTCTTTGATTTCAAATTTTGAGAGAAAAAATTTCATTATTATTTTTTATTGATGTGTTATATGTTTTTTCTTTTTATATATATAAGTTGGGGATGTCAA
GTGCCTTAGACACACTATGCGGGCAAGCTTTTGGAGCCAAGAAGTACTACATGTTAGGCATTTACATGCAACGTTCATGGGTGGTTCTTTCAATCACTGG
GGTTATGTTTTTGGCTTTGTTCCTCTTTGTGACACCTATCTTGAAGTTCTTTGGACAGACTTCAGAAATAGCTGAGCTAGCTGGAGTGATTTCGTTGTGG
TTGATACCTACCCACTTGGCTTATATATTTTATTTACCTATGCATTTTTTCTTGCAAAGCCAACTAAAAAATAATGTCACTACTTGGGTGTCTCTATTGG
GTTTATTGGTGCATGCATATCTTTGTTGGTTGGTTGTCAACAAGTTTCATCTTGGAGTGATTGCCCTTGTTGCCTTTGGAAACATTGCTTGGTGGCTTTT
GGTGCTAGGTTATTTTGGTTATGTTATTTGTGGTGGTTGCACCTTAACATGGACTGGTTTTTCCATCGAAGCATTCTCTGGTGTTTGGGAGTTCTCCAAA
CTCTCTACTGCTTCCGGGATCATGATTTGGTATATTTTCCTTTCTTGCAATTTAATTGCACTCCCACTAAAGTTAGCATTAACTTTTATTAGAGCTCAAA
AAATGGATCTAGCCGAGTTATGGCCTGAGAAAATATTACTTCAAATAGATTTGGATCGACTCAATGGGCCCGGATCAAATTGGACTCAATAAATATGTCT
GAATTCAATTAACCTATTTTGGTAGGATAATTTTTAATTTTTTAAAATTATATATATTTAGACAAAAATATAAAATAGATTAATATGGAGCACCAAACCT
GATTAAACTTCAAAAAATGAAATCATAATAAATATGGATTGGCCCAAACTAATCCAATTTTCATACTTGACCGCCGTTCTTTTTGCAAGCTTTAAGTTTT
ATTTGTGCAACTTTAATTATTTCTTTATGGAGTTATTATTCTATTGAAGTATCATCATAGGCTGTTGGATTTTTCTTTTTCAGCTTAGAGGTTTGGTATG
ATAAAGCACTTATGTTGATGACTGGGAACCTACAAAGCGCAAAGACTACTATCGAGGCTTTAACCATATGGTTAGTGTGAGAGAGTATATATTTTCCTTT
TAACTTAGTGCATACTCTCGCTCCAAAAGGATTTCTATTTTTTAAAATAACTAGCACTCAAATATCATTCTTTTTGTTCGATTTGTGGGCTACAATGTTT
CTTACTTCTGTCTTTTTATGGCAGTTTGACCATAAATATATGGGAGTTGATGTTTCCTCTATCGTTCTATGCTGCAACTGCGTAAGCATCTTCCATGCTT
CTATTTTTGTGCATGTACTTAAAAAGGAAAAAGAAACAGAAGAAATATTTTATAATGGATAATTGTTATAATAAAATAACATATTTATAACCAAGACTCT
ACTGGAATGATTTTGTAAAAGAGTTAAGATTTTGACATATAATAAAATCATATATTGATCTTGTGTGATGGTTTTGTAGAGTAAGGGTAGCAAACGAACT
TGGAGCTGGCAATGGAAAAGGGGCCAAGTTTGCTTCCATGGTGTCAGTGGTGACATCAATTATAATAAGCATTTTCTTTTGGTTGTTGATTATGGTATTT
CGGAGAAAGCTTGCGTATTTATTTTCTTCGAGTGAAGTTGTGATTAAAGAGGTAGACAAGCTTTCACCTTTCTTAGGCGTCACAATTCTACTCAACAGTG
TTCAGCCAGTTCTATCAGGTGATATTATCTAACTTTTACCATCCATCGTCAATTATTTGATTAGATTTAACAATGAATACTAAACTAGACCATATTACAT
ACTTTTTTTTACTGGCCAATGGTTGCCTTATTATACTTGATCAACTTATCATTTAGTTACTTCACCATTGTAATTTGTTATATTATTTAGCTATTTTTTT
AGACTTGAATATATTTTTCATACCTGATAAATTTTTAAAGTATACTAAAAGTACTTCTGTTATCTTGCATTGATATATCAACAGGGGTAGCTGTGGGATC
TGGGTGGCAGAAATATGTGGCATTCATAAACTTGGGTTCCTATTATCTTATTGGATTGCCACTTGGGTATTTGTTAGGCTTCGTCTTTCGTTTGGGAGTC
CAGGTATGTTGTGTCCATCTCTTTTTCAATAGAAAGATATCTTAAGTTTTTTTTTTAATTAAGGCAAAGATAACTTAAAATTAAACGATAAAAAGTGATT
TAAATATAATTTCGGTTGCTCGTACTTATACATCTGTGTAATTTTTGTCCTTTTACTTCTTCTAACTGACATTAAACTTATACTATAAATTAATCTTAAC
TGACATTAATTACTTACGTGGACAAACCATCAGGGAGTTTGGGCCGGGTTAATTTTTGGTGGACCGGCTATTCAGACTTTGATCCTTGCTTGGGTTACCT
CTCGATGTAACTGGGACAAACAGGTAGATAAAGCCACTTCTTCTAAAACAATTTTCACTCTTTTAGTAATATCTTTGCAAGTGCATGTGGCCAATTTTTA
TTCTTATACATTTTTTTGGTTAACAATCACAGGCTGAAAGAGCAAGGTTACATTTGACTAAATGGGACCCAAATCAAGAACTGATCAGGTAGCAGTTTCT
TTCACTTGCAATTGAAAGAAGAAGAATGAGTGGGGCTTTGTTGGGGCATGTG

>Glyma.19G120200 | Chr19:37773951..37778723 forward
TTTTCATATCAATAAGACATTGACAGCTCTATAGTAGCAATGGAAATGGAAGAGGAGCTAAAGGAGACGCTTATGCATCATCCAAACAACACTTCTTTGG
AGAGTGTTGAAAAGGAAGAGTCATTGAGGAAAAGGGCATGGGAAGAAAGCAAGAAGATGTGGGTGGTAGCAGGCCCCGCCATATTCACAAGGTTTTCAAC
GTTTGGAATCATGGTGGTTAGTCAATCCTTCATTGGGCATATTGGCTCAACTGAACTAGCTGCCTATGCCATTGTTATGACTGTCCTAGTCAGGTTTGCA
AATGGTGTATTGGTAAGTGTTTCTCTACCTTTGGATGAAATCAAATTACTCTATCCAAACAAAAACACCAAAAAAGGAATCAAATTTAAATCAAAGCAAT
TCGAATTTCGTGCATTTTTCTTGAAAATGTTAAAATTTTCCATCTAAACACATTAATTTTCTCTACACCCAATACTCTTTAGCTAGTTATCTAAAATGTG
AAATCTAAAAAATTTCAATTTCAATTGTAGATTGGAATGGCAAGTGCATTGGATACTCTTTGTGGGCAAGCATATGGGGCCAAAAAATATGACATGCTAG
GAGTGTATCTTCAAAGATCATGGATAGTGTTGTTCATGACCTCAATACTCCTTCTTCCTATTTACATCTTTACCACTCCTTTGTTAGAGGCTCTAGGCCA
AGATAAAACCATTGCTCAAGTTGCTGGAAGCATTTCTCTGTGGTCAATAGGTATAATATTTGCTTTCTCTGTCTCATTCACCAGCCAAATGTTCCTACAA
TCACAGAGCAAGAACAAGATCATTGCCTACCTTGCAGCAGTTTCAATTTCAATTCATGTTTTGTTGTCATGGGTTTTAACTGTTCAGTTCAAGTTTGGGC
TCAACGGTGCAATGACATCAACACTTTTAGCATATTGGATTCCTAACATTGGCCAGCTTGTGTTTATAATGACTAAGTGCCCTGACACATGGAAAGGTTT
CTCATTCTTGGCCTTCAAAGATCTTTTGCCTGTTATCAAGCTTTCCCTCTCTTCTGGAGCTATGTTATGGTAAGATATTAAGCTATTTTGCAACATCAAT
TCTTTTAACCAAGTAAACTTGTAAAGCTAGCTGTGCTTGTTTTGCGTTTAGCACATTACTGATTTTCTTAAATGATGCCTAATACATCACTTCTCAATTT
TGTCAAAGTTTTCACAAGCGCACTAATTTTGTGCTGCCGAAAAAACAAAAAACTATAATTTTTGTATGAATGAAAAGATCTAGAACTAACATAAATTATG
GTTCCTTTTACAGTCTTGAGATCTGGTACAACACTGTTTTGATTCTTCTTACAGGCAACATGAAAAATGCTGAAGTGTCTATTGATGCTCTGGCCATATG
GTAATCTTGTTCTTACTTTTACTTTTGCTTCTAAATTTCATAATAAGATATAAGGGACATGATATCCCTGCTTGTTCATATTGAAATTAATCTGTTTATT
TAATCATAGTATAAATGCATATTTGAATATTCTTTTGCAGCCTCAACATTAGTGGGTGGGAAATGATGATAGCCCTTGGTTTCTTTGCTGCAGCAAGGTA
CTTCTTTTTCACAATCTCTTTTTTTCCTTTCAAATGTCTGAGGAATAAAACAAAAAGGAATGGAATTTTGAGTAAAAGATATAGCCATAAATGCAAAAGA
AAAACTATTTTTACTTTATAATAAATAAAATAAGAATTTAATAGACATAGGAGTGGAAATGGATTGGGAAACAAAGTTTTATATAATTAGACCTATCTTA
TTTTAAAATATTGAAAACATAAACAGACTTGTTATTTTATTATAGACTTCTTTTTAAGTTTGACGTAACCTAATTTTTTTTAACATCCATTTTGACCTTT
AAGTCTGACTTCTTAATTGTTATAGACTACTTTTAAAGCATAATTCTGACTTTTTAATTCATAATATATATATTTTTTTACTTAGGTGTGGCTTATAAAA
ATAAAATAACTACTACTCAAGGCAGGGAACTAGGGAAAAAATAGGTACGCTAGACTTTGGGCAAATTTGTCTAAATTATTTTTTTTAAGAATGTTTTTAA
AATGTTTTTCTAAAAGTAGATAAAGTTTGTTTATTAAAAAAAAATCAATTTTCTGTTTTCTTAACAAAAAAATAATTTAGACAAACACATGAAAAGATGC
TTAATTATTTTGTTATTATATTTTTTTAAAAAAACAACTTAAACAAATGAACCTTTTATAAAAATTGAGTCTAACTTATGTTTTATAAGCTTATCTTTAA
GGTCTAATATAAGGTGAATGATAACTTATTTAAAAATTCATTTTGCAATCAGACTTTAAAATAAACATAACTTTATCCTTCAATAAACTTATAAGTTTGA
CTTTTTTATATATATCAACATTCCTAACATTTAAAAAAGTAAATAAATTTATGATTTTAATAATAATAATAATAACAACATAGAGGTTTATATAAACATT
TACACTTACATAATAAAATAATCTGTTAAGCTTCAAAATATTTTTTGAATGACTTAAGGTGTAACATTTTTAACTAGTTATACTTTTATAAAATCATTAA
GCTAGTTTATTTCCTAAAAGAAGTCTGGTATGACTTGACTGGACGTAGTCTGTGTATAAACTATTATTTACATTATCAATAAAAAAAATTATCCTTTATA
TGACTTTGTTGGTAGTTAATTAAATTACTTATCATATATTATTTGTAATGAGATAAAAGTATAAAAAATGTTACATGCATATATTATTTATTTATTAATT
ATATATCTTATTAGATTTTTCATTCTTTTTCTATATCTCTTGTGTTTAATTTGTTATAAGAATTTAATATTTATAAATAACCTTATTTTGAATAAGATGT
TTAATTTCTTTATAATAATTTTTTACGTGAAATGTTAAGGCTTAATAATTAATTTGTTCTATTGGATTTAAATATAAAATTATCTTTTAATATGAATTCC
GAGTTATTTATAATTTTTGCAGCGTCCGAGTGGCAAATGAGCTTGGAAGAGGGAATTCAAAGGCTACAAAATTTTCCATTCTGATAACAGTCCTCACATC
ATTTTCCATTGGATTCGTGTTATTCTTGGTGTTCCTATTTCTGAGGGGAAAACTTGCTTACATTTTCACTCCAGACCCTGAGGTTGCTAAAGCTGTTGGC
GATTTATCACCATTGCTCTCATTTTCCTTCTTGTTGAACAGTGTCCAACCTGTGCTTTCTGGTATTATTATTATTATTATTATAAAATTTACAATAATTC
ATGCATTCTGTTTAACTACCTGATGTAGATCACCTTGTTTAACAAATATAAGTTTTGTTTTTTTTACACATTAGTTCATTAAAAACACTACTATAGCTTT
TATTAGTAATTTTTAGGGACAAAAATGAGATTTTTACATTTCTAAATGTTAACGTGTTTATAATAAAATCATTTAGGAACAAAGTAGGTCTATACTTTAA
ATTTAATCCAATTCATTCAATTATAAAAATGTACAATAGACTGGATTGAACGGTCCGAATTTTTTATTCTCTTGGTCGTGAAGTGATCATACGCATAAAT
GTAATGCAGGTCGTCTAACCCCAAGAAACCAAAGGAATCAAGTTTAAATCATGAATATAAACAACAAATGGTCATATACTGATTTCTGCTTTAACTTCTA
AGATTATTGTTTAAAGATTAAGATTAAAAATATGGATTAATCTCGAGCAATGCATTGCCAACTGACTGGTAATGTTATTATGAATAGGAGTTTCTGTGGG
TGCTGGGTGGCAAAGCGTTGTAGCATATGTAAACATAGGCTGCTATTATCTTATTGGTATTCCTGTTGGAGTGCTGCTTGATAATCTTTTCCATTTGGAA
GTTAAGGTAATTCATTTATCTTTTGCAATTGCATGCATATATAGATGCTTGTGTCAAATTTATACTGTGAAGAATAATCAATAACTAAATATATAATAAT
ATAAATTTAGATCACAGAATAATTAACTTTACTTTTAAACTATTTTCAAAAAGAAAATTAAAGGTTAATCAATTTGAAATAGAGTAATAAAGTTCATAAA
TAATCATACTTCTTATTTTTTTTTGCTGAATGTTCATAAATCATACTAGTAGTCCATAAAGCTTGATACGATAATTTCTGAATTGTAATGCCTTCTCATA
CAGTGTCTTTTTTACTAAGTGGAGATAAGTGAACAAATCTATGTTACTTTTGCAGGGTATTTGGATTGGAATGTTGTTTGGAACATTTGTTCAAACCGTA
ATGCTTATTACAATCACATTTAAAACTGATTGGGACAAGCAGGTAATCGATTTTCTGTTGGCTCTATATATGATTGTGAAATTAATTTTCCATGTTTCAG
GGAATTAAGCTGTTAAAATTTTATATGAATGTTGCAGGTAGAGATAGCTCGAAATCGTGTTAACAAATGGGCAGTGACGACGGAGAACGAAGAATCAAAC
AGTAGATCAAGCATATCTAGCTAGGCATTTGATATTTTAGTTCTCCCCTCCATCATTTTATTTATTGAAAATTAAAACAATATATTTTATTGGATTAGAT
GTTATTTTTTCTCATTTTATTTTCTTTTCATAGGTAAAATTATTGATCTGATAGGGTTTTGTATGTACTACAACAAGGCCAAAGAAGAACGTTATTTTCC
TAAATTCGAGGCCAATAAAGATACCTCCAGAAATACGAGGGAATAAAATTTTCTAAATGATGGTCTTTTATTT

>Glyma.19G120700 | Chr19:37861157..37863438 forward
ATGTCAAGTGCGCTGTCAACACTATGTGGACAAGCATACGGTGCAAAAGAATATGCTATGATGGGAGTGTATCTTCAAAGATCATGGATAGTTATGTCCT
TAACTACACTCTTCCTTCTTCCGGTGTTCATCTTCACACGACCCATTTTGATGCTCTTAGGCCAAGATGAGATTATAGCAGAAGTGGCAGGAACCATTTC
TCTTTGGTCAATTCCAATCATATTTGCTTTTATTGCCTCGTTCACTTGCCAAAATTTCCTGCAATCTCAAAGCAGGAACACCATCATTGCACTCTTGGCG
GCTTTCTCAATAGTTATTCATGTGTTTCTCTCTTGGCTATTGACAATTCAATTCAAGCTCGAGATTCCTGGTGCAATGACTTCAACAAGTTTGGCATTCT
GGATTCCTAATATTGGTCAACTAATATTTATTACATGTGGTTGGTGCTCTGATACATGGAAGGGTTTCTCATTTTTAGCATTCAAAGATCTCTGGCCTGT
TGTCAAGCTTTCCCTTTCATCTGGTGTCATGTTATGGTAAGCTTTCATCTCTACCAACTAATGGGCTAAGCTCTTTAGAAATGCCATGACTGCCATATAA
ATTTGGATTTTCTTTATGATGAATGTAGAATACAAAGTATTAACTATCTAAATTTCTATTATGCAACTTATCCCAAAAATACATAATGCATATATGTCTT
CCTAAGTTGGAGCTATGGTATATTTTACAGTCTTGAGCTCTGGTACAACACAATATTGGTTCTCTTAACGGGCAACATGGAAAATGCTGAGGTCCAAATT
GATGCTCTATCTATATGGTATTCCTTCTTTAATGTCTCACTTTGTTAGAATTTGAAAAATGCTATATTATAGGACGAAATTAATTTGAATTCATGAAATT
TTGAGTAATACACATCAAATCGGAATGAGATTTACATTATTTTTTGAGTTCCTATAAATGTTAGTTAATAATAAAGAGTGTATTGACAAAAAAAGAAGAA
AGGAAAAAAAAAAACTGTTTTGTTTTGTGATAATTTATATGTTGTATCCACCTTAGCTACTTGGTCGTATTGGCCTAATGCATAAAAGCCTTTTCTCTTT
CGCTGCTTAAGATCAAATTTCATTTGTTTACAGCCTCAACATCAATGGATGGGAAATGATGATATCACTTGGTTTCATGGCTGCTGCAAGGTAATGGCAT
TCTTTTCTCTTCCTTTTGTTGTAGTCATTCTTTTTGCTATCTGTGAAATAGAAATTGGACCATTTGGCCAGGAGGACCGAAACAAGCACTTACGCCTTTC
AAGCTTAGCATTTGAGGGGCTGTGAATGGTTCTGACAAGAGGGTCAACCTCACAAGAACTCAACACTCATAAACCTACCAATTTTTCATCTGTTGTTCAT
TTACCATGGAATTTTAACTTTATCATTTCTCATTTGCAGTGTTCGAGTGGCAAACGAGCTTGGAAAAGGAAGCTCCAAGGCTGCAAAGTTCTCTATAGTT
GTGACAGTGCTTACATCCTTGGCCATTGGATTCGTTCTCTTCTTATTTTTCTTATTTTTAAGGGAAAAACTTGCTTACATATTTACCACAAATAAAGACG
TGGCCCAGGCTGTTGGTGACTTGTCACCTTTGTTGGCAATCTCTATTCTACTAAACAGTGTCCAACCTGTACTCTCGGGTATTTTTAAACAACATTTGGC
CTCCAAGTTGGTTTAAGACTAGAGTCTGATATCAAAATTGAATATTGAATGTGGACCAAAGAAACAAAAATTACGAAATGATGCACTTAAACATTGTAAT
TGTGTGATTTCAACAGGAGTTGCTATTGGAGCAGGGTGGCAAAGCATTGTAGCATATGTGAATATTGGGTGTTATTACATCATAGGTGTTCCTGTAGGAG
TTCTACTTGGTAATGTTCTGAATTTGCAAGTCAAGGTCGGTATTCTTCTCATTCTCAGGTTGCATACTTGTATTTGATTGGTGTTTCTCTATTGAAAAAA
TCTTCTTCTTTCTTAGGGTATTTGGATTGGAATGTTGTTTGGAACATTTATTCTAACTGTAGTGCTAATTGTAATCACCTACAAAACTGATTGGGACAAG
CAGGTATTTGATTCATGACCTCTTATTGCTTCTTAGTTCTAGATTTGCCCCGCAAAAAATTATAATTTATTATATAACGAATGAATCATTATGTTACTGT
GCAGGTTATTATAGCTCGTAATCGTATTAACAAGTGGTCTAAGGTAGAATCTGATCATGAAATAATTACATCAGATAATTAG

>Glyma.03G005600 | Chr03:504869..509004 reverse
TGTATTAATTAAAGCCATGCCAACACCTTTTTTTGGTTTAAAATAGAAAAAAATTCAGGCCTACACTCCCTCATCACTTGCTTAGTTTGTTCTTCCTTCT
ACTAGCAAAGCAAAAAAAAAAAAAAAATAGAGAAATGCAGGGGGGTGTGAAGGAGAAGTTGTTGATCAAAGAGAAAAAAACATCAGAACAAGAAGAAGAG
GAATTATCATTGGTGAAGAGGGTGTGGAATGAGAGCAAGGTGATGTGGGTAGTGGCAGCACCAGGCATTTTTACAAGGTTCTCCACCTTTGGTATCAATG
TTATAAGCCAAGCATTTATTGGTCATATTGGTTCAAGGGAACTTGCTGCTTATGCTCTTGTGTTCACTGTTCTCATACGCTTTGCCAATGGAATTCTGGT
ACGTTTTTCTAAGCCAAATTAATCTTCCTTCACAATTTTTTTTTTCCAGTAGATCTAGTCTCGAAAATAATTTTTTTTTAAATAAATAATTTTGAAATGA
AAGTGGGTCAGGGGCATAGAGCTGGGAGAGAAAGAGTGGATAACTGTGAGGGAGAAGAAAATGCCACTGAATACTTTTATACAGTATAAATCTCTGCATG
TTTAGATTATAGTTTGGGTTAAATTTCAAACTAAGTTTGCAAAATTTGAAAAAAAACAACCTTATGTCTTACGTCAGCAAAATTCAGTTTTCGGGCTAAA
TCTTTATGTGCATATATACTTTTGGAAATGACAAAATATGTCAGCAAAATGAGTTTATTAACCAAACTGAAATTTCCCAAACAGAAAATCAAACATAATC
TTAGCCTCTGACTACGTGATGTACTGTCCTAGTAATTTTTAAAATTAAAAAAAATGTTAAATAAAGTTTTGAAATTAAAATATAATTCTTAATATATATA
ATTTATTATCATTATGATTCTTAAAATTTATAATTTAAGTTAAGGGCTATTAGGAAGGACTAACATGATTAACAAATTTGAATTTCAGATATTTTTATTT
GATATATATATATTTTTTTTATTTCAGAGATTACTACGTCAAGAACCAAAATGGGGGGTTTACTCCTTTTATACATGATTACCTAATTATGAAAAGTAAC
AACTAACAACACTATAAAATGTTATCTGTGCGACAAAACTACCCTTTCCAATTTTAGGCGAGATATTCTTTCTGTAGAATGATTCAAAGAAACAGAGACC
TCTGCCATTTCCTGTGTTTCATCAATGAGTAACTTAAAGATTTCCTGAGTTTTGTTTTTGGTTCGTATGAATCTATATTAGTGTTATAGTAGTGATTTTA
ATCATTGGTGAATCAGGAGTTGTAAGAAAAACAAATGCATCAAAGAGTGAAAAGGGTTATTAAAGTAGTGAAAAAGTTGAAGCTGAAGCTTAAGCTTGTG
AATACCTGCTATGACAATACCCAACATATGCTTCCCCTACATATAATATAATATGGCTTCAAATGCAATACATTCAACAAAAATATGTTTCCACCTTATT
GTTATTGCAATTACAATATAAAATGCTATTGATGTAACGCAAGTTATTTGTGTCTGTTGTAACAACTCTAGTTAGGAATGGCGAGTGCGTTGTCAACACT
TTGCGGACAAGCATACGGTGCAAAAGAATATGTCATGATGGGAGTGCATCTTCAAAGATCATGGATAGTTATGTCCATAACTTCACTCTTTCTTCTTCCG
GTGTTCATCTTCACGAGGCCGATTTTGATGCTCTTAGGCCAAGATGAGAATATAGCAGAAGTGGCAGGAAATATTTCTCTTTGGTCAATTCCTATGATAT
TTGCTTTTATTGCCTCTTTCACTTGCCAGAATTTCCTGCAATCTCAAAGCAAAAATACCATCATTTCTTTCTTGGCAGCATTCTCAATAGTTATTCATTT
GTTCCTCTCTTGGCTATTGACAATTCAATTCAAGCTCGAGATTCCTGGTGCAATGACTTCAACAAATTTGGCATTTTGGATTCCTAATATTGGTCAACTA
ATATTTATAACATGTGGTTGGTGCTCCGATACATGGAAAGGTTTCTCATTTTTGGCATTCAAAGATCTTTGGCCTGTTGTCAAGCTTTCCCTTTCATCTG
GTATCATGTTATGGTAAGCTTTCATCTACACCAACTAATGGGTTAGGCTCTTTAAAAATGCCATGAGTTCCATGTAAATTTGGATTTTCTTTATGATTAA
TGTAGAATACAAAGTTTTGACTATCTAGATTTCTTTTACGCAACTTATCTAAAAAATTAATAATCCATACATGTTTTCCTAACTTGGAGCTATGGTATTT
AACAGTCTTGAGCTCTGGTACAACACAATATTGGTTCTTTTAACGGGCAACATGGAAAATGCTGAGGTCCAAATTGATGCTCTATCTATATGGTATTCCT
TCTGTATTGTCTCACACTTCTTTATTAGAATTTGAAAAATGCTAGCAACACACTTTGTCTCAAACATTATTTATTATAGGATGAAATTCATTTGAATTCA
TTAAATTTTGACTAATGGTCATCAAATAGGGAATGAGATTTACATTATTATGTGAGTTCCTATAAATTTTAGTTAATAATGAAGAGTGTATTGGCAAAAG
AAAAAAACCTGTGTTTGTTTTGTGATAATTTATATATTGTATCCACCTTTAGTACTTGGTAACTGTATTGGCCTAATGCATAAAAAACTTTTTTCTTTGG
CGGCTTAAGATCAAATTTGATTTGTTTACAGCCTCAACATCAATGGATGGGAAATGATGATATCACTTGGTTTCATGGCTGCTGCAAGGTAATGGCATTA
TTTTCTCTTCCTTTTGTTGTACTAATTCTTTTTGTTATCTGTGAAATAGAAAATGGATTGTTTGGTCAGGAGGACTTACCTCAACAAGTGCCAATGCTTT
ACAAGCATAGTGCTAGAGGGGTTGTGGACGGTTTTGCCAAGAAGGCCAACCTCAACAAGCACCAACACACACAGACACACATCTAAACCTACCACATTTT
CATCTATTGTTCATTTACCATGGAATACTAACTTTATGGTTTCTCATTTGCAGTGTTCGAGTGGCAAACGAGCTTGGAAAAGGAAGCTCCAAAGCTGCAA
AGTTCTCTATAGTTGTGACAGTGCTTACATCCTTGGCCATTGGATTTGTTCTCTTCTTATTTTTCTTATTTTTAAGGGGAAAACTTGCTTACATATTTAC
CTCAAATAAAGACGTGGCCGATGCCGTTGGGGACTTGTCACCTTTGTTGGCAATCTCTATTCTACTAAACAGTGTTCAACCTGTACTCTCAGGTATTTTT
AAACAACATTTGGCCTCCAAGTTGGTTTAAAACTAGAGTTTGATATCAAAATTGAATATTGAATATGGATCAAAAAAACAAAAATTACGAAATGATGCAC
TTAAACATTGTAATTGTGTGGTTTCAACAGGGGTTGCTATTGGAGCAGGGTGGCAAAGTATTGTAGCATATGTGAACATAGGGTGTTATTACATCATAGG
TATTCCTGTAGGAGTTGTGCTTGGTAACGTTTTGAATTTGCAAGTCAAGGTCGGTATTCTTCTCATTCTCGAGTTGCATACTTGCATTTGATTGGTGTTT
CCTCTATTGAACAAAACTTCTTTATTCTTTCTTAGGGTATTTGGATTGGAATGTTGTTTGGAACATTTATTCAAACCGTAGTGCTGACTGTAATCACTTA
CAAAACTGATTGGGACGAACAGGTATTGATCATTATTTCTTCTTAGTTCTAGATTTATCCCAAAAAAAGTATCAATTATTGAATAACGGACGAATCATTT
TGTCAATGTGCAGGTAACTAAAGCTCGTAATCGTATTAATAAGTGGTCCAAGGTGGAATCTGATCATGAAACAATTACATCAGATGATTAGTTTCATGTT
CCTTCTTTGATTTAGTTTTTTAAATTCAAAATTAATGAATCTAGCCACGGATTTATTTATGGCAATATATTTTCTGTTGCTAGTGTTATACAGGCAAATA
TTGTTTATTTGTATGGACGCAAATTATGTTGGATAGGATCGGATCCTTGAATTTAATTGGACATTGTTATTTAAAAAAGCAATACTCTACAACCAATATT
TTTTTTTAAGGAAGATGCTTATATTATTTCATAAAC

>Glyma.03G005500 | Chr03:495973..501101 reverse
ATGTGGGTAGTTGCAGCACCAGCCATATTCACAAGGTTCTCCACCTTTGGTATCAATGTTATTACTCATGCATTTGTTGGTCATATCGGTTCAAGGGAAC
TCGCTGCTTTTGCTCTCGTTTTCACTGTTCTTATACGATTTGGCAATAGTATTCTGGTATTTTTCCTTTTTCTTTCTTTCTTCTTATTATTTTTTTTTAC
AAATTTTCCTTCCATTTTATATTAAGCTATGAAGCTCGTCTACTTCTTTTGTTCGAACTATCATAATGTAGGACATTTTTGTACTATGACACTTTCCGAC
ACACGTGTTCAACTCTTCTAATTAAGTGTTTAATTAAAAAATTATTTTTTCTAAGATACTTAATTTATTATCTAGACACTTGAACAACACCTTTATGTGA
ATAAACAAACACTAATATTATTTAAGAATAACTTAAATAACTTTTTGTTACTTTAAAATAGAGTGTTTTTTATTTTAGTCTTTTAAAGATTTTTATTTTT
GTTTTAGTGCTGTAATTTTTTTATTTATTTTAGTATGTGCAATTTTTGTTTTGTTTTAATTCATGTTTTCAACTAACAATGTTAACTACGAAAGAGCTTA
CAACGACATGTTTTATATGTATTTAAAGACACTTCCAAACTGTCTTTAAAGTCAACGTAAAGATTTTCTACAAAAATTTCTTAAAAATCATTTTGGAAAA
ACTATCATTTTAATCGCATAACATTAACTTGTTAAGGAATGTCACAAGACCGTCAAATATTTTTTTCATATAAACCGTGCTTTTAAATCCTCATTAAGTA
TGCATTATTTTCTCTGTTCTTTTAGTGGTTTAAAGATTTTTGTTTTAATTTTATTTTTTTTTAATTTCTGTTGTCACTTCTCGTTAATTGTCGCAACATA
GAGATAAAATTCATGAAAAACTGGCAAAATTTACAACATTGTGAAACTTTTGGTGATAAAAGTGCAACTAAGCCTGCTTAATTTTATTACGATGTAATTG
ATTTGCAGACAATGAAAACACTCTTCAAAAGAATCTACCCTTATAAAATTTTAAACAAGATCTACTTAATTTTATATACCGATTCAAAGAAATAGAGATG
ATAACGATTAAATATGAGTTATTTTTTATAATAAAAATATAATGAAAATATCATTAAAAATATGTATTTAGTAGTTATTTATGCTTAATTGTTAAGAAAT
GATGTTTTTTTCATTCATGATTTACAAATATGAAAATGAGGGATTAAAAACAAAAAAAAAGACAAGAAAATACCAAAAATATGAAAAAGGTCCAACCCAA
GACGCGCTTAGCACAGCATGCGCCGGGCGTGAATACATGCACTCTCGCTAAGCGGGAAGAGGCGCATTCAGCGCAAAAACATTCAGTGGCGCTAAGCGAG
AAGAATTGTGTTAAGCACGAATATAGACACCCGCGCTAAGCGATTAACGCAGGCTTAGCATGAGTATGCAAACCCAAGTTCACTCCAACAATTATAAAAA
TGGAGTCAAACAAGGAGAAAAGACACACCGAAGTCTCAGAACACTCTAATACACACCTAAAGCATAAAAACTCTTCCATAGGAGATTCCTTCTTCTCTTC
CATTATTTTTTATTCACTTTTTTCATCTTTTCTTTTCCATCAGTTTCTAAACCCCTTTTCAAGTGTAAGACCTCTTATGTCTATGAGAGGCTAAACCCTT
AGTTAGGGTCTGACAGACCTAAAAAGCCAAAAAATATATTGTATGCTTCATATCTATCAATGCAAATAGGTGTTTTCTTTCCTATTATTCTTTCCTACTT
TAATTTCATATATCACTCATCTTTGCATTATCTTTAAGGGTTAGGTGCTCGATAGAGGATAATCCTTAATAGAAATACAAGGAAGGTCTTACATGCATCT
TTTTAGGGATTAGTTAATCGACAGAGGATAGTTCCTAATAGAACTAAAAGAAAGGGATATCTTAATAAAATCATTGTTAGACATAGAGTGATTGCATTAT
GCACATGCATCAAAGCAAGCATCTAGAATTAGGACTTCATGCATTATCTTTTTCTTTACATTTTATCTATTGAGTCTTTGCAAAAACATTTGAGAGATAG
ATAACTAAGATAGGCTTATTATCGTGAGACATTAAGGGCAAGTATTCTAATAAATATGGGTAGGGAAAATTCACCTAATTTGTAAAGAAAAATATAAAAT
AATACATCTTAAGCAAATAAAACATGTCAGGTCCTAACATTCTCATCTAATTAAATTTCTTATTCTTTCTTTTGTCTTCTATTAATAGTTATTATTTTAT
ATCTTGTTCTTTTAAATTCATCTTTCATACCTCATCTCATTTTCTATTTTTATAAATTAAAATTATCCAACACAAGTACAAAATAAAATATCTGTGAGAA
TCGACACTCGAACTTTTGAGTCTTTACTACTTGGACATTTTGGTACACTTGTCAAATGATCAACAAGAGACCTATGACCTCTCTCCTCTGTTTCATCAAT
GAGTAAATTAAAGATTTACTGAGTTTTGTTTTTGGTTGGTATGAATCTATATTGGTGTTATAGTAGTGATTTTAATCATTAGTAAATCAGGAGTTGTATG
GAAAACAAATGCATCAAAGATTGAAATGGGACTAAAGGAGCTTAAAAAGTTGAAGTTGAAGCTGAAGCTTGTGAACACCTGCTAAGACAATATTCACAAT
ATGCTTCCCTTAGATATAGATATGGCTATGTATTGGTACATTATTGTTATTGCAATTACAATATAAAATGCTATGGTAGTAACGCTGATTATTTGTGTCT
TTTAACTAACAACTCTAGCTGGGAATGGGTACTGCATTGTCTACACTTTGTGGACAAGCATATGGTGCAAAAGAATATGGCATGATGGGAGTGTATATTC
AAAGATCATGGATAGTTTTGTCCTTAACTGCACTTTGTCTTCTTCCCCTGTTGATCTTCGCAATCCCAATTTTGACTCTCTTAGACCAGGATGAGACCAT
AGCACAAGTGGCAGGAACCATTTCCCTTTGGTCAATTCCTGTCTTGTTCTCTTTTATTGTCTCATTCACTACCCAGACATTCCTACAATCTCAAAGCAAG
AACATCATCATTGCATTCTTGGCGGCTTTTTCGATAGTCATTCATGTGTTTCTCTCCTGGCTCTTGACAATGAAGTTCAAGCTTGGGATTGCTGGTGCAA
TGACTTCAACAAGTTTGGCACTATGGATTCCTAATATTGGTCAACTAATATTTATAACATGTGGTTGGTGCTATGATACATCGAAATGGAAAGGTTTCTC
ATTTTTGGCATTCAAAGATCTTTGGCCTGTTGTCAAGCTTTCCCTTTCATCTCTACCAACTAATGGGTTAATGTAGAATACAAAGTTTTTACTATCTAGA
TTTCTATTATGCAATTTATCCAAAAATAAATAAATAAATAATCATAAACAGTTACATACATGTTTTCTTAACTTGAAGTTGTGGTGTATTTTACAGTCTT
GAGCTCTGGTATAGCACAATATTGATTCTTTTAACGGGAAACATGGAAGATGCAGAAGTCCAAATTGATGCTCTATCTATATGGTATTCCTGCTGTATTG
TATGACACTTCTTTATTAGAATTTGAAAAAGGAAAATGATTCTTGGACACTCAAATAAAATTGATGATATGATGTGACAAAAGAGAGATAGAGAAATAAG
AAATGTTTGTAAATAATGGATGTTCAAATATTACCACTTAAAGAAAACTGTGATGTTTCCCAATAATTTATATACCTTATCCATCTTTGATACTTTGTTG
TATTGTCATAATGCATAAAAAATTCTTCTCTTTGGCTGCTTAAGATAAAGTTTTATTTTTCTACAGCCTCAACATAAATGGATGGGAATTGATGATATCA
CTTGGTTTCATGGCTGCTGCAAGGTAATGACATTCATTTAACTTCCTTTTGTTGTAGTCATTCTTTTTGTTATCTGTGAAAATTGGATTGTTTGGGCAGG
AGGACTGATCTAAACAAGCACCTACGCCTTTCAAGCATAGTACTCGAGGGGCTATGAATGGTGGTGCCAGGAGAGCTGACCTTAAATATAAACCTACCAA
TTTTTCATTTGTTTGTTTACCATATGGAATATTGACTTTATCTTTCCTCATTTGCAGTGTTCGAGTTGCAAAAGGAAGCTCCAAAGCTGCAAAGTTCTCT
ATAGTTGTGAAAGTGCTTACATCTTTTGCCATTGGCTTCATTCTCTTCTTTATTTTCTTATTTTTAAAGGAAAAACTTGCTTATATATTTACCTCAAGTA
AAGATGTTGCCGATGCTGTTGGAGACTTGTCACCTTTGTTGGCAATCTCTATTCTACTAAACAGTGTTCAACCTGTACTCTCAGGTATTTTTAAACAACA
TTTGGCCTCCAAGTTGGTTTAAGTGCATGTTTGTTTTCACATCCTCAAAAAATTTATGGGCATCAATTGGAAGCTAGACATTGTAGCTTCTGCTTAAAAC
ATTCAATTCCACGTGGTTTTTTCACGAGACTTGCGTGTAGGATGAAATTCCAAACATGCTGTAAGACTAGAGCTTGTTAGGTTTTGCATGACAAAATCAA
AATTGAATATGCACAAAAAAACAAAAATTACGAAAAGATGCACATGAACATTGAAATTTTGTATGATTTCAACAGGAGTTGCTATTGGAGCAGGGTGGCA
AAGCATTGTAGCATAAGTGAACTTAGGGTGTTATTATATCATAGGTATTCCGGTAGGAGTTGTGCTTGGTAACGTTTTGCATTTGCAAGTCAAGGTTGGT
ATTCTTCTCATTCTCAAGTTGCATTTGATTGGTGTTTCATCTATTGAACAAAACTTCTTTCTTCTGTTGTAGGGTATTTGGTTTGGAATGTTGTTTGGAA
CATTTATTCAAACTATAGTGCTAATTATAATCACCTACAAAACTAATTGGGATGAGCAGGTATATGATTCATTTCTAGATTATCCCCTAAAAATTATAAT
AATATATGTATAACTAGATAACTAGATGGATCATTATTTGACTGTGAACAGGTAATTATCGCTCGTAATCATATTAATAAGTGGTCGAAAGTGGACTCTC
ATCATAAAATAATTCCATCAGAAAATTAG

>Glyma.19G120300 | Chr19:37782534..37786674 forward
AATGGTCCATCTCTGGTTTATTTCAAACAAACGCCACTGTCTCTTTTTTTCTTTCTTTCTTTCTTTCACCTGAACGCAGTGCCGCAAGCCAAAAAAATGG
AGGGGCATCTAAAGCAGAAGCTGTTGAGAAACAAAGAGAGAAGAACATCATCAGAGCAAGAAGAAGAGGAGCTATCATTAGTGAAGAGGGTGTGGAATGA
GAGCCAGGTGATGTGGATAGTGGCGGCACCAGCCATATTCACTAGGTTCTCCACCTTCGGAATCAGTGTCATAAGCCAAGCCTTTGTTGGCCATATTGGC
TCTAAGGAATTGGCTGCATATGCTCTCGTTTTCACCGTTCTCGTTAGGTTCGCCAATGGTGTTCTCGTACGTTCTTTTTCATCAAACCTTGACAATTTTT
ATTTTGTTTTGTTTTGTTTAAAAAAAAAAAGTGGAACAAACCGTGCCGCATGTGTTGTATTGTATCATATTTTGACTATGTTTCTGTGTTGCTGTCTCAC
TATGTTGGGTCTAGTCTAACATGGCGAATCAAGGTTCGAATTTCTGTTGAGAGAGGCGTTTAGTTTTAGTGTTTGATAGTGTCTCAACCAGGATTCTCTT
TTAAAAAATATACATGTTGGGGAAAAGGAAAACATGTTATTCTGATTTTTTTTAATAACCCTGGTTGTTGTTCTGTATTTCTGTATTGTGACTCAAGGAA
ACAGATCTGTTTCATCAATTAAAAATTAAATATTTTTGTTCTTTTTTTTTTGGTTAATCAGAAGAAAATCTTTTGGTGTTATGGCTTGAATTATTGTGAT
TGTTTGAACTTGGGTTATAGGGAGAAATATGGATTGTAACGTCTGCAGTTGGTGAACATATGCTATCACCATTTTTAAAATTACATTACTTTGTGGGTGT
GTGTACTTTTGCATTTATTTGATTGATTTTAATTTAACCTATGTATGGTGTACAGATGTAACTTTTTGGATGAAACTGTATTGGCGTTGTGTTACAATTG
CAATTACAAATTTACAATATGATGCCTTTGATAATGACTCAAATTTATTGTTTTGTGAAATGACAATTGCAGTTAGGAATGGCGAGTGCGTTGTCAACAC
TTTGTGGACAAGCATATGGTGCAAAAGAATATGGCATGATGGGAGTGTATCTTCAAAGATCATGGATAGTTTTGTTCCTAACTGCAGTCTGTCTTCTTCC
GGTGTTCATCTTCACAAGCCCAATTTTGATGCTCTTGGGCCAAGATGAGAGCATAGCACAAGTGGCAGGAAACATTGCTCTTTGGTCAATTCCTGTCATG
TTTGCCTCCATTGTCTCGTTCACTTGTCAGACATTCCTTCAATCTCAAAGCAAGAATGTCATCATTGCGTTCTTGGCAGCATTTTCGATAGTCATTCACG
TGTTTCTATCGTGGCTTTTGACAATGAAGTTCCAGTTTGGGATTCCTGGTGCAATGATTTCAGCAGGCTTGGCATACTGGATTCCCAACATTGGTCAACT
CATATTTGTCACCTGTGGTTGGTGTTCTGATACATGGGAAGGTTTCTCATTTTTGGCATTCAAAGACCTCTGGCCTGTTGTCAAGATGTCCCTTTCAGCT
GGTGCTATGTTATGGTGAGTTTTCTTCTCAAGTGGGCTAAGTTCTTTGCAAATATTGTTGGTCTTGAATTGACCTGGATTCTTCCTATTAATAATAATAA
TTCATTTTTTGACTTTAAAAAAGAAGAGTAAACCTGGATTCCCTTCAGTCGGGGAATTAATATTGGGTGAAGATCAGATGACTCATTTGAACAATCCGAT
CTTCATCAAATGGTTTCAATTTACTTTTGTGTGAATATGTGACTTCATAGAATCCATTTCCATTTTGAGTGTCAATCTTGACATGTTCTGTCATTAATTA
TTTTTACTAGCAAATTGTGCCTCATTTTTCAGAAGACATTGCTTATTCTCCTTAACTTTTCTCCTTTCCCATTGACGAGCCCTTATTCACTTAGTGTAAA
AGGGTGTGAGAAAAAGATGTCAATGAAACACTTTTTTCTTAGAGAATCTAGATTGTTACTATGTTATTTATCGTGAACAAAATGTAGATAAATAGATACA
TGTCTTTCTAACATGAAGCTGTTGTATATTTTACAGTCTTGAGCTCTGGTACAACACAATATTGGTTCTTTTGACTGGTAACATGAAAAACGCAGAGGTT
GAAATTGATGCTCTATCCATATGGTAATCCTTCTGCACTAGAACACTTTTGATTAAAATTTAAATGCTGTGTTTCTATTGTATGTTGTTGGTTGCATTAA
CTTCATGTATATTAGGAGAATTTTTCTCTTGTACTGCTAATGGTGAAATTTTACTATTTACAGTCTCAACATCAATGGATGGGAAATGATGATATCACTT
GGTTTCATGGCTGCAGCAAGGTAATTTCACTGGTTTTTTCACCAATCTCAACCTTCCATTTTGTTTTCTTTTTCTTCTGTTGTAAGTTTACCCTGAAAAC
TTCACCTTCTAACTTTATTTTGCAGTGTTCGAGTAGCAAATGAGCTTGGAAGAGGAAGTGCCAAAGCTGCAAAGTTCTCTATTATTGTGTCAGTGCTCAC
ATCATTGGCCATTGGATTCCTTCTGTTCTTATTCTTCTTATTTTTTAGAGAAAGACTTGCATATATATTTACCTCAAATAAAGATGTGGCCTTTGCTGTT
GGGGATTTGTCACCTTTGTTATCAGTCTCTATATTACTAAACAGTGTTCAACCTGTACTCTCAGGTATTATCTGCACCATTTGCTCCCACTTGAATTGAT
TTAAGATTTGAATCCTTGTTGTTTTATATTACTAAGCGAATTAATGCATGTTTGGGTTAAAGTTTGCAACTGAGTTTGCAAAATTATCCCTCGTCTTGCT
TCCCCCAAACTTAGTTTCTAGACAAAATTTTACTCTCAAACGTACTTTTGGGAGTAACCAAACATAGCCCAAACTGATTTTACTATCAAACATTCTTTTG
AAACCATCCCATCAGAAATCCAAACATGCCCTTAATCTGTCAATGCCTTAAACTTACTACAGTTGTATGTTGAATGTGTCATTTTTCTATGTGTATATAT
ATACACTTCTGAAATGAGATTAGTGATTTCAACAGGAGTGGCTATAGGAGCAGGGTGGCAAAGCATTGTAGCATATGTGAATATGGGGTGTTATTACGCC
ATTGGTATTCCTGTAGGTATTGTACTTGGCAACGTTCTCGATTTGCAAGTCAAGGTACTGTTTCAGCCATTTTGTTGCTTTTAAATTGCAGATCACCTTT
TTGCTGCTTGATGTATTAAACAAACTTTTTCACTTTTGTAGGGAATATGGATTGGAATGTTGTTTGGAACGTTGATTCAAACTATAGTGCTAATTGTAAT
CACCTATAAAACTAATTGGGATGAGCAGGTATGCATTTGACTTAATGCTAGTATAAACTCCTAAAAAGTAATTATAGGAAAAGAAAATAAAAAACTTATA
AAAGTAGCTTACAATTCCCATAAGCTCTTTTGAGCTTATTACAATAAACATTTTGGTTCAACTTTTCCAAATAAGCTCTTTCTAAGTTATTTCAATAAGT
TTTGTGGTTAAATTTACAGCGTAAGCTCTCTTGTGATGAGATCTTACTGAATAAACGCTTAATTTTATTTAACCAAATGCTCCTTAAAACAACTCTTATA
AATTAAAATTGACTTAAAGATACTTATACTTTTGAGAAGTGTTCTCACATAACTTCTCTAAAAGTAAATTAATTTCAGCTTATGAGAAAAGTTTGATTCA
TTTTATTTTCTTGTTTTGTCTACAAGTACTTTTTGATATATTTATCCAAAACTGAAACCAAACATTATTTACCTCAGATTGCTTCAAGTTACTACATTCT
CTTGCATAATAGTTATTTTTATTGAACATGATCACATCATTTGTGCAGGTTACCATTGCTCAGAAGCGTATTAGCAGGTGGTCGAAGGTGGACAATGCTG
ATCAAGAAAATGAAGCACAAAGAAAATATGTTAGCTAATTAGTTTCACTGTCATCATCTGTGTACTATGTTGTTATACAGCAGAAAACGTTAATAGCAAT
TGTACGGTTAATCATATTTTTTTTTTGAAGAAATGGTTATT

>Glyma.03G005800 | Chr03:545102..549977 reverse
GTCAGCAAATCAAACAGAACAACAACTTGAACTTGAATATATATAATGGTTCAACTCTTTGTTTATTTTAAACAAACGCCATTCTCTCTTTCTCTCTTTG
ACCAATCCCAAACCCAAAACAAAAAATAAAAATGGAGGGGGATCTGAAGCAGAAGCTGTTGAGAAGAAGAAAGGAAGAGGAAGAAGAAGAAGAAGAAGAG
TTATCATTGGCCAAGAGGGTGTGGAATGAGAGCAAGGTTATGTGGATAGTGGCGGCACCAGCCATATTCACAAGGTTTTCCACCTTTGGAATCAGTGTTA
TAAGCCAAGCTTTTGTTGGCCATATTGGTTCTAAGGAATTGGCCGCTTATGCTCTCGTTTTCACCGTTCTCATTAGGTTCGCCAATGGTGTTCTGGTACT
TTTTTCTTCACCAAAAACCTTGCTATTTTTTTTTATTTTTTTTATATAAAAAAAGTGGAACAAACTACGCCGCATGTGTAGTATTGTATCATGTTTTGAC
TGTTTTCTTGTGTTGCTGTCTCACTATGTTTGGTTTGGTGTCTCGTAACTTGACGAATCAAGGTTCGAATTCCTATTGAGAGAGTTGTTCTCGTTTAGTT
TGATTATGTCTCAAACAGGGTTCTCTTGAAAAAGAAAAATACATGAAAGAAATAGAGGTTACTCCTCTGTTTCATCAATTAAAAATTAAATTTTTTCGTT
TAACAAAATCTTTTGGTGTTGTAGGTTGGATTGTTGCGATTGGTTGAACTTGGGTTATATGAACACATGGATTGTAACATCTGAAGTTTGTGAACATATG
CTATAACAAATACATTTAGTGTTTGATTGTAACATGAATTATAACAAAAAAAATTCGAATTTCTGTTCAGAGAGGTGTCTACGTTTAGTGTTTGATTATG
TCTCAAACAGGATTCTCTTAAAAAAAATACATGTTGGGGGAAAAAGAAAAACATGTTATTCTGATTTTTTTATTTTTAAATTTAAACCTAGGTTGTTGTT
CTGTGTTTTCTGTATTGTGACTCAAAGATACTGAGATTGCTCCTCTGTTTCATGAATTACAAATTAAATATTTTTGTTCTTTTTTAATTTGTTAATCAGA
TGAAGATTTTTTGGTAGGTTATATGAGAAACATGGATTATAACATCTGAAGTTTGTGAACATAGGCTATCACTGTATTTAAAATTACGTTACTTAGTGGG
TGTGTGTATTTTTTCATTTATTTGATTTTTACTTAACCTGTGTATGGTTTGGATGAAACTGTATCAGTGTTGTGTTACAGTTGCAATTACAAATTTTACA
ATATGATGCCTATGATAATGACTAAAATTTATTGTTTGTAAAATGGAAATTGCAGTTAGGAATGGCGAGTGCGTTGTCAACACTTTGTGGACAAGCATAT
GGGGCAAAAGAATATGGCATGATGGGAGTGTATCTTCAAAGATCATGGATAGTTTTGTTCTTAACTGCAGTCTGTCTTCTTCCTGTGTTCATCTTCACAA
GCCCCATTTTGTTGCTCTTGGGCCAAGATGAGAGCATAGCACAAGTGGCAGGAAACATTGCTCTTTGGTCAATCCCTGTCATGTTTGCTTTCATTGTCTC
GTTCACTTGCCAGACGTTCCTGCAATCTCAAAGCAAGAACATCATCATTGCGTTCTTGGCGGCTTTTTCGATAGTCATTCACGTGTTTCTCTCCTGGCTT
TTGACAATGAAGTTCAAGTTTGGGATTCCTGGTGCTATGATTTCAGCAGGCTTGGCATACTGGATTCCTAATATTGGTCAACTCATATTTGTCACATGTG
GTTGGTGCTCTGATACATGGAAAGGTTTCACATTTTTGGCATTCAAAGACCTCTGGCCTGTTGTCAAGATGTCCCTTTCAGCTGGTGCCATGTTATGGTG
AGTTTTCTTCTCAAGTAGGCTCAGTTCTTTGCAAATTCTATGATTGTTGGTCTTGAGTAGACCCGGATTCTTCCTATTAATAATAATAATTCATGTTTTG
ACTTAAACAATAAAAATAAAAAAAAGTAGACCCGGATTCCCTTCAGTCGGGGAATTAACATTGGGTCAACATCAGATGACTCATATTTTATCTCGCAAGT
CTGACTTAAAAGTTAAAATACACATCCAAAATTTGAACAATTCGATCTTCATCAAATGGTTTCAATTTACTTTTGTGTGAATGCCTGATAGCATAGAATC
CATTTCCATTTTGAGTGTCAATCTTGATGTTTTATCATAAATCATTTTTACTAGCAAATTATGCCTCATTATTCAAAAGACATTACTTATTCTCTTTAAC
TTTTCTCCTTTCCCTTTGATGTGACCTTATTCACTGAGAGTGTAAAAGGGTGTGAGAAAAAGATTTCAATGAAACACTTTTTTCTTAGAGAATCTAGATT
GTTACTATGTTATTTATCGTGAACAAAAATTGTTACATGTCTTTCTAACTTGAAGCTGTTTTATATTTTACAGTCTTGAGCTCTGGTACAACACAATATT
GGTTCTTTTGACGGGTAACATGAAAAACGCGGAGGTTGAAATTGATGCTCTATCCATATGGTAATCCTTCTGCACTAGAACATTTTTCACTAAAATTTAA
ATGTGTTATGTTATTGGTTGTATTAGTTTGATGTAAATAGGAGTTTTTTTTTTTTCTCTTGCACTGCTAATGGTGAAATTTTACTATTTACAGTCTCAAC
ATCAATGGATGGGAAATGATGATATCACTTGGTTTCATGGCTGCAGCAAGGTAATGTCACTGTTTGTTTCACCAAGCTCAACCTTCCATTTTGTTTTGTT
TTTCATCTGTTGTAAGTTTACCCTGAAAACTTGACTTTAACTTTATTTTGCAGTGTTCGAGTGGCAAATGAACTTGGAAGAGGAAGTGCCAAAGCTGCAA
AGTTCTCTATTATTGTGTCAGTGCTCACATCATTGGCCATCGGATTCCTTCTGTTCATATTCTTCTTATTTTTTAGAGAAAGACTTGCATATATATTTAC
CTCAAATAAAGAGGTGGCCTTTGCTGTTGGGGATTTGTCACCTTTGTTGTCGGTCTCTATATTACTGAACAGTGTTCAACCTGTACTCTCAGGTATTATT
TGCACCATTTGCTCCCTCTTGAATTGATTTAAGATTTGAATCCTTGTTCTTATATTACTAAAGCAAATTAGTACATGTTTGGATTAAAGTTTACTCAAAC
TTAAGTTTGCAAAAGTATCTCAAGTCTTGTTTCCCAAAAACTTGGTTTCCAGACAAAATTTTACTCTCAAACATACTTTTGGGGGTAACTTAACATAAAA
TGATTTTACTCTTAAACATACTTTTCAATCCATTCCAACGGAAATCAAAACATGCCCTGAATATGTCAAATGCCTTAAACTTACAACAGTGATATGTTGA
ATGTGTCATTTTCCAATGTCTACATAGACTTCTGAAATGAGATTATGTGATTTTCCAATGTGTATATATACTTATGAAATGAGATTGTGTGATTTCAACA
GGAGTGGCTATAGGAGCAGGGTGGCAAAGCATTGTAGCATATGTGAATATGGGGTGTTATTATGCCATAGGTATTCCTGTAGGTATTGTACTTGGTAACG
TTCTGGATTTGCAAGTCAAGGTACACTTTCACCTATTCTGTTGCTTTTAAATTGCAGACCATCTTTATGCTGCTTCCTGTATTGAACAAACTTTTTCACT
TTTGTAGGGAATCTGGATTGGAATGTTGTTTGGAACGTTGATTCAAACTATAGTGCTAATTGTAATCACCTACAAAACTAATTGGGACGAGCAGGTATGC
ATTTAACATAAGGGCTAAGGATAGTATAGGAAAATAAGATAAAAAAACTTACAGAAATAGCTTCTAATTTCCACAAGTTCTTTTGAGGTTATTTCAATAA
ACATTTTGGTTCAACTTTTCAAAATAAGCCCTTTCTAACTTATTTCAATAAGTGTTCTGGTTAAATTTACAGCATAAGCTCTCTTGTGATGAGATCTTAT
TGAATAAACACTTGATTTTGTTTACCCAAACACTGCTTAAAACATCACTTATAAATTAAAATTAACTTATCCACTTTGACTCTTAAGAAGTGTTAACTTC
TCTAAAAGTAAATTAATTTCAGCTCATGAGAAAAGTTTGATGCATTTTATTTTCTTATTTTTTCTGTAAGTACTTTTTGACATGTTTAATTTATCCATAC
TGATCCTTATTTACTGTCAGATTGTTTCTAATTAACTACATTCTCTTGCATAATAATTATAATTATCTTTACTGAACATGATCATATCATGCTGTGCAGG
TTACCATTGCTCAGAAGCGTATTAGCAGGTGGTCGAAGGTGGACAGTCCTGATCATGAAAATGAAGTAGAAAGAAAAAACGTTAGCTAATTAGTTTCAGT
GTCATCATCTATGTACTATGTTGTGATACAGCAAAAAAGTAGATAGCAATTGTACGTCATTTTTTTGGTGAAGAAATGGTTATTTATTGATCATAAAGTC
TTCAATCAAAAGTAGTGGACTTCTTTCTTACTTTTGAATTTTTCAATAGAGACATTACTTTTGCTCGTACTCGATTGACCTACTCGGTTAATGGATCTTT
ACGTGCAAGGCATTATTAATGTTACATGTTCTTAATTCTCAACTTTAACATTTTGTAATTAGTGTGGTAAGGATGGTAGTTAGTACTCATAAGGATAGAT
CAGAAAAAATGTCCAGAAAACTAGATGGTAGGAAACTATATAATACCAGAAATTAATGCAGTTGTAGATTTATTGTATATAAATAGTAACAAGCATGTTT
CTAATAATTAACAACAATCCAACTATATATATAACTAAATATGTGGTAGATATAAACATTTAATATAACAATCACA

>Glyma.03G005400 | Chr03:483748..490996 reverse
ATCTAATATATAAGCACTGGCTAGATTCACTTGTGTTTATTTTTTTCTTCCTTTTTCTCTTTTTATAATATCAGCCCCCAAACTCCCTTGTGTACAGTGA
TCGATCTCAAGAAGAAAGACTCAAAAAATGGAGGGGAATCTAGAGAAGAAGTTGCTGAGCAAAGAGGAAGTGTCAGAAGAAGATAATTTATCATTGGTGA
AGAGGGTGTGGGAAGAGAGCAAAGAGATGTGGATAGTGGCAGCACCAGCCATATTCACAAGGTTTACGACCTTTGGCATCAATGTTATAAGCCAAGCTTT
TATTGGTCATATTGGTTCAAGAGAACTCGCTGCCTATGCTCTTGTATTCACTGTCATCATACGTTTCGCCAATGGTATTCTGGTATGTTATGTTATGTTA
TCTTCTTACTCTCAAATTTTAATAATTTTCCTATGCCAGATTTTCAGAGTGCTTTAAGTATTTACTATTTTTTATACGAAGCTGTCTTCAACTCTTCATA
TCATCATATATATATTAAACTCAGGCAATGATAAATCATTTTTTCACCAATTACCAATTCTTAAACATACCTATATATACACCTTGATTATCAACTTTTA
TAGACATATAACTGAAAGGAAAAGGAAAGATATAAGATTGATTTGAAGATTAAAAAAATAAGATCAATAAAAAGTAATGAAGTTGATTTCTCTTATTAAT
AAAAATTAATAATTAACCTTTACGGCTAAAAAAGAAAAACTGAAGGAGAAGAGACGGAAAAAGTGAGAAAAGTTTTGGATTAAGCTATTAGGTTGGCCAC
TTGGCTCAATCTAATAGAAAAACGTGATTTCCGCTGATCGATGAGTTGTGTGTGGACACCACCAATTGACTTTGTGCCTACTTTTGTTTTGTTATTATTA
TGCATGAAAAGAAGAGAAAGAAATTGGATGAATAGAAAAATAGTATAAAGAAATAAATAAATTATAATATTTTTTATACAAATTAAAAATGTATTTTTTT
TAATTTATTATTTTTTCATAAAATCCCTTTAGAGCAAATTAAAGTAATATATTTCATGAGATTTTATGTAATTATACAAGTATTTTTTTTAATCATTGTA
TTCATTTTTCTTATAAGGAGTGTTAGTGTAAGATTTGAGAGAGTGCTGAAAAGGTGTTAGTGTAATATTTTAAAACTTAAAAGGAGTTATGGCAAGAAAA
ATAAAAAAGAGTAGAGTTATGTGTAAGTTTAAAGAAGTTTAGAGAGAGTATTGTTGTAATTTGTTAAATTCTTTATTATTTTTTAATTTTTATAATATTT
TAAAAAATAAAAAAATAGAAGGAAAGAATTTATTTTCCTGCCATTTTATTTCAATTTATATAAAAAAATTAAATTATTTTAGTTTCATTTTTGTACATGA
ATGCTGAGATATCGTACCTCTAGACAATGTTTAAGGCCAGCCTTGGGTTGTGATTTTTAAAATTTAAGATACCACAACAATATAATTGCCGCCAATTACT
CTTTATTTTATGGTAATCCTAATTTTAAGAATTAAAAATTAAATTATCTTTGTAATTATTTAATTTGGTTTGATGTGGTAGTTATATCCACACGTGTTAC
TTCAAGGATATATATATTAGATAAGGTCAACAAGGTTTACATTAAATTATGCACATACACCCAGCTCTCTCTTGTCTTGAGTTGAAAATATTAAAATAAA
AAGTGGAATTTTTAGTTTGCACGCAAATTACATCCAATTGTGCACCTTGGATTGCATAGGAGATGCAACAAATAAAAAATAAAGCTAACATTAAGAAGGA
GAAGTTGAGAGTCATTAATGACTTTGTCTTCTTGTTTCTTTTTTGACCTTTTTTTTTTCTTTTTTTCTAATCTTTTTTATGATTCTCAATAGAAAATAAT
GGTATATGTAAGATCATTACATTAAACTTAATGCAATGCGGCTGTTGCTCAATTGTAAAAGAACATCATACATCTAACCCATACACCCAGATTTATTAGT
TTTATACCTAACATATCAATATTAACTCAAAATTTTCGAAGGATGTTTAATAACCTAAATAAAGAAAAAAATTAGTTTTTTCTCATATACCACCTGTTAT
GAGTAGATTAAACTAATTACATTTCCTTGATTCCTATTTTTAATTCTTATTTTATTTATTAAATTTATAGATAAATTCTAATAATATATCCATAATTGTA
GTCATCATATTTTTTTTATAAAAGTATTAAAAATTTAATTAATTATTATAATTGAATTTAATATTTGCATTATTCTACAAGTGTAGTGTATCTTGACAAT
AAGGTGGACTATAAAAAAAATATTTGCAAAAAAAAGTTAGTTAAGAAGTGAATATGGTAATGATAGTTAATGCAATTAGTACATTTTAAAATGTACATTG
TAACAATAAATTTCTGTTAAATAGAATATTTTTTGTACAAACCATTGGTACCCTCGTGAAACAGCGGCAAAGTTTGGATTAGTGTTGATATTTTATGGAC
TTGAGCCAAATAACTTTTTTTTTTATAAATAAATATTAAAAAAATTGGAAAACAAGTGTTTTCCGGTAGATCTCACTGGAATAAAAAAAAATCAAATATT
TCACCTCTCAAACATATTAGAAAAATTAATATCTTTATCATGTTTATCTCATTCTTATTTCGTTCATCTCTTTTTCTTCCCAAACAAATAAGGGTGAAGT
GCAAGCCTAAGGACTTTGTTTGATATGGAAAGATCAAAATAATTTTGGGTAGATAGAAATAAGTTGGTGTCTTAATTGGTAGCCGAAAGTTAGAAGGGAA
ATAATTTTGAAATTGGTGGGACTTACTATGATATTTCTCATATATTTTTACTCATTGCTTCTTTTCTATTTATCTTCTACCAAAAATATTTATTTTTCAC
TTGTTTCCACTCTTTTATTTTATTTTCATCTACCATATTATTTCTCTCTCTACCAAACATAGGGAATAAGGGGATTGAGATGTGAAAACCCCTACAATGA
CCCCTTCCTCCCTTTCCATTTTCTCTGTAATCAAATAAGCTTTTAAAAACTAGAAAGAAGCATGTGCCCTAAGTATATAATCATTCTCTTATGTTATACC
ATATTCCGGATTTTTTACCATTCAAGGTGTAATTTTTGTCGGCTTTTTTCACATGGGGTGCTTTTCAAATTATTTTTTAAAAGGAAATAAATCATAATAG
ATGTTACGATTTATTTTATTTTTTAACAAAAGTCGTAAAATTATTTACAATTTTAATGTAATTTTTTTAAGTATTTAAAATAAAACAAATATTAAAAAAA
TTAAATATCGTTATGTTAGTTTTTTATTTCATTTACAAAATAAATAAAATTCAATAGCATTATTAAAAAATTATACAAATTTTAAATATATTAAATAAAA
CAATATTAAAAAATATATAAAATGCTTTCAATAAAACAATATTAAAAAATATATAACATACTAAATAAAATCATATAAAAATATAAACAATATATTAAAT
AAAAATAATAATAAGTAAAACTAAAAATATTTATTTAATAATTAAATAAATAAAATTTAATTAAAATTTTTTTAAAAAATTGAAAACAAAAAATTACATG
TAAATAAAAAAAATCCTAAAGCAGTAAAAAAAAATACTACGACTTCAAAAGTTACATCATTAAAAAAAACTTACAACTTTAAAGTTGTAAAATTAAAAAA
AAAATTAAAACTTTGAAGTCGTAAATAAAAAAATTATGTCAAAGTTATAAATGATTTTATGATTTCAGTTAAAAAAATAAATAAATCATACTAGATGTTA
CAACTTCTAATTAAGAAGACATAACACCTGTTCCGACTTATACTTTTTGAAAAATAATTTGAAAAACATCCTTAAAAAAGTGAAAAAAATTGCACCCGAA
TGGTTAAAAAGCCCCATATTCCCATATGGAACCTCAACTAATTAATAAGACCTCTGGGAATTAAAAGAAGACAATACAATAATCGAAACACTGCATAAAT
AGTTCCATATATATTCAAAACAAAGAAGGAACAATATCATTAATAACAAAGAAAACAAGATAAACAGATCACAGACAACCTAGAAACACAACCTCAGCAA
CAACAGGATTTGATCAGAACTATAATACACGAATATAAATACATACATAAAATAGAAATAACAACAACAATGATTCACGGATAATGGTAAGCTTCAATCA
AAATATTTGAATGAGCCTTTTAGATTTTTGACAATGTCATCTTGTATACAACAACTCAAAAAAATATTGATCTATTCTTTTAGTACTTTGTATCGTGAGA
AAACGAATTACATTTTCTGACTTTTGTTTTTGGTTTGTTTAAATCTATTGTGTTACATAGTTACATTATATAGAAGTGATTTTAGTCAATAGGGAATCTA
GGGTTGTAAGAAAAGATATGCATCAAAGCTTGATGGGATTAAACTAAATTAAAAAGTTGAAGCATATGAACATCTTCTATGACGGTATACAAAATTAATT
ACATATTTACATTGTTGATGAGGGTAGTAGCTTTAATCATATTATTTGTATATATACTTCACCTACATTTGGCTATTAGCTACCATTACAATTTATTCAG
TGAAGAAATATATATTGGCAAGGAATAACACAAATTATTTCTGTCAACTGGCAATTGTAGTTAGGAATGTCAAGTGCGTTATCAACACTTTGTGGACAAG
CATACGGTGCAAAAGAATATGACATGATGGGAGTGTATCTTCAAAGATCATCGATAGTTTTGTTCTTAACTGCACTCTGTCTTCTTCCTGTGTTCATCTT
CACAAGCCCAATATTGATGCTCTTAGGCCAAGATGAGAACATAGCACAAGTGGCAGGAACCATTTCTCTTTGGTCAATTCCTATCTTATTTGCTTATATT
GTCTCATTCAATTGCCAGACATTCCTTCAATCTCAAAGCAAGAATGTCGTAATTGCATTCTTGGCAGCTTTATCAATAATCATTCATGTGTTTCTTTCTT
GGCTATTGACTATACAATTCAAGTTTGGGATTCCTGGTGCAATGATTTCAACAATTTTGGCATTCTGGATTCCTAATATTGGTCAATTAATATTTATTAC
GTGTGGTTGGTGCGATGAAACATGGAAAGGTTTCTCATTTTTAGCATTCAAAGATCTTGGGCCTGTTGTCAAGCTTTCCCTTTCATCTGGTGCCATGTTA
TGGTGAGCTTTGTTCTCAATCAACAAAAGGAGTTAATAAGCTCTTTATAAATGCCATGATTGCTGGATAAATTTGGATCTAATTAGTGTAGGAAATTAGG
AACAATTTAACATATATGTTTCCTAACTTGGAAGTTATATATATTTTACAGTCTTGAGCTCTGGTACAACACGGTATTGATTCTTTTGACTGGTAACATG
AAAAATGCTGAGGTTGAAATCAATGCTCTCTCTATATGGTAATCCTTTTGCTCTCTCTATATGGCAATCCTTTGTACTGATAGCAAATCTATGTTGAAAT
TTATATGTTGAGTTTCTATTTTATGTTGCCGGTTTTAATGGTCAGACTTATAGTAGGCTCTTCTCGTTTGGCCTAATTACGATTAAATTATATTATTTAC
AGTATTAACATCAATGGATGGGAAATGATGATAGCACTTGGTTTCATGGCTGCAGCAAGGTAATGATGCCCTTTACCTTTTTATGGTTGTAATCCTTGTT
TTTGTCATCATCTACGAAATAAATAGTATATATATATATATATATATATATATATATATATATATATATATATATATATATATATATATATATATATAAT
AAATCACAAAAAAATCAATTAGTTGTCATTTAATTTTCTATTTATTTTTTGTTTGCTGTAGAATATTAACTTTATATTTCTTAATTTGCAGTGTTAGAGT
GGCAAATGAACTTGGAAGAGGAAGCTCACAAGCTGCAAAGTTTTCTATAGTTGTATCAGTGCTTACATCATTTGTAATTGGGTTTATCTTATTTGTTCTT
TTTTTATTTTTAAGAGAAAAAATAGCTTATCTTTTTACTTCAAACGAAGATGTGGTTACTGCTGTGGGGGATTTGTCACCTTTGTTAGCGCTTTCTTTGT
TACTAAATAGTATTCAACCTGTACTCTCAGGTATGCTATATAACTTTTGGTGTCTTGGTTTAATGAATTTGTTTAAGATTTGAACCCAATTGTTTATGCA
TGATAAAGCCAAAATTAATTTGTCAATTGGGGAGTCTGAAAGTCATGAAATTGTATGATTTCAACAGGGGTGGCTGTTGGAGCAGGGTGGCAGAGCACTG
TAGCGTATGTGAACATAGGGTGTTATTACCTCATAGGTATTCCAGTTGGAATAGTACTTGGTAACATTATTCACTTGGAAGTCAAGGTAGGCTATTATGT
TTTACTTTCCTCATTTTCAAGTTACACCTGATTCTATCACTTCTCATCCATTGGACAAACTTTCTGATTTTGTAGGGTATTTGGATTGGAATGTTGTTTG
GGACACTAGTTCAAACTATAGTCCTAACTATAATCACCTACAAAACTAATTGGGATGAGCAGGTATGCGATTCATTTTCCTATCATTGCTTCTTAATGTA
AGTTACTATTCGATGTTGAACTTGATGAAATCGTTATGTCAATGTGCAGGTAACTATAGCCCGTAATCGTATTAGTAAGTGGTATAAGGTGGAACTTGAT
CATGAAACAAGTACATCAGATAATTAGTTTCAGACTCCTATTTATCAATATCATCATTGTTTGTTTGGCTATTGTACGTTATACATTCAGGGGATTGCTT
GATAGTTATTATACTATAGTACCATCCTATCAGTATATTCTCTCTCAAATATTTCTTAAATTAATAAAATTATATAAGTAGTTGCTAAAGTATTAAATGT
TCCTGTATTTAGACCTTGGATTTATATATTTTACTAGCATTGAGGGATTATTTTGAAGAAATTTTTAATATTCGAGGTACTATGTAGCATTGCATAGTTT
TTTCTTCTGTAAAAAAAATGTAGCATAATTTTTAATACTTGAGGAACTATTTATACAGTTTCATTAGTTTAAGGGCTATTTAAAAAAGAGGATAATACTC
CAGAGACCAAATTAATTGATGGTTTTATTCAATTATTTTATGGTTATTTTTTCTTTTTTTTACTGGTTATTTGTTTTTTCATCTTAATAGGAGAACACAT
TTTACTATTTAATTATATATATATTTTTTAATTTTACTCAAGATACAAA

| >Glyma.03G005200 | Chr03:459177..462834 reverse GAGCCCCAAACTCCCTCCTCTATTCTGATCTTAAGAAGCCCCCAAAAAAAGGCTCGAAAAATGGAGGGGAATCTAAAGCAGAAGTTGCTGAGCAGAGAGA AAATATCAGAAGAAGAAGAGAATTTATCATTGGTGAAGAGGGTGTGGGAAGAGAGCAAGGTGATGTGGATAGTGGCAGCACCAGCCATATTTACTAGGTT CACCACCTTTGGCCTCAGTGTTATAAGCCAAGCATTTATTGGTCACATTGGTTCAAAGGAACTCGCTGCTTATGCTCTTGTATTCACTGTCATCATACGC TTTGCCAATGGTATTCTGGTATGTTATGTTCTTACTCACAATTTCTTTTTTTCCTAGGCCAGAATTTGAGAGTGCTTATAAAGCATTTTGTTATTTTTAA GAACTGTTCATCTGTTAAACTCATAGGCAATGAATATGATACATAATTTTGTCAACAATATTTGTGAATCCTTGTATATATTAAGGAAAATGTTTGATAG ACAGCCAAAAGCTTTCCAATACCTAGAGAGAAAGAAAAAAGAGATTTTTTTTTGGTACAATAGGATTTTCAAATGGACTAATTAAAAACAAGAAAAATAA TATAGGAACTAAAACTATATATATATATATATATATATATATATATATATATATATATATATATATATATATATATATATATCAAGAATCAAAACCAAAA GTAGTAATTTTTTTAAAAATATAGGCTAAATTTTCTTTTTCCTTATGAAATTGACAACAAGGTTTGGATTTCTCGTTTGTGCATAAATTTTCATCTTCAT AAATATAAAATTATAAGGTGTCATTTTTTTAGCCCTAAGCACAACTGAACCTATTATGATAACTTTTACATCATCGAATATTTTTTTTATAAATGTCATG TTTTTTATATGTCATATATATATATATATATATACATAAATTCGGATATCAGAACCTTAGCTACGGATCATAAAATGATACATTATAATTTATTTAGGGT TGAAGATGGATAAAATTTTTGCTGGAACAAGATCCGAACTTTGCAAATTCTAAAAGAAAGATGAGCCAAAAACATATTACAAAATTACATTGTTGTTGAT CAGTGCAGCTTTCATCATGTTATATGTATATATAATTCAACTTCATATGGCTATTGGCCAACAATACTATTTATTCAGTGGAGAAGTATAGGCAAGGAAT AACACAAATTATTTGTGGGTGTGAACTGGCGATTGTAGTTAGGAATGTCAAGTGCGTTGTCAACACTTTGCGGACAAGCATACGGTGCAAAAGAATATGA CATGATGGGAGTGTATCTTCAAAGATCATCGATAGTTTTATTCTTAACTGCACTCTGCCTTCTTCCGTTGTTCATCTTCACAAGCCCAATTTTGACTCTC TTAGGCCAAGATGAGAGCATAGCACGAGTGGCAAGAAACGTTTCTCTTTGGTCAATTCCAATCTTATTTGCTTATATTGTGTCATTCAACTGTCAGACAT TCCTTCAATCTCAAAGCAAGAATGTCATAATTGCATTCTTGGCAACATTATCAATAATCATTCATGTGTCCCTCTCTTGGCTATTCACAATACAATTCAA GTATGGGATTCCTGGTGCAATGATTTCAACAATTTTGGCATATTGGATTCCCAATGTTGGTCAACTAATATTTATTACATGCGGTTGGTGCCCTGAAACA TGGAAAGGTTTCTCTTCTTTAGCATTCAAAGATCTTTGGCCTGTTGTCAAGCTTTCCCTTTCAGCTGGTGCCATGTTATGGTGAGCTTTGTTCTCAATCA ACTAAAGGGGTTAAGGTCCTTAGAAATGCCATGATTGTGAAATAAATTTGGATTTCCTTAATTTGTATGGGAAATTAGGAATGGATTACTAAAGTTAGAT GCATCCTAACTTGGAAGTTGTTGTATATTTTACAGTCTTGAGCTCTGGTACAACACAATATTGATTCTTTTGACTGGTAATATGAAAAACGCTGAGGTTC AAATTGATGCTCTATCCATATGGTAATCCTTTGCACTATAGCATATCTATGTTGAGTTTCTATTTTATGTTGTTGATTTTATTGGCCAGACTTAAAGTAT GCTCTTCTCTCTTGGCATATTGAGATTAAATTTTATTATTTACAGTATTAACATCAATGGATGGGAAATGATGATAGCATTTGGTTTCATGGCTGCAGCA AGGTAAATTACATACTTTACCTTTCTATGGTTGTAGTCATTGTTTTTGTCATCATTTAGAAAATAGTGTATATATATATATATATATATATATATATATA TATATATATATATATATATATATATATATATATATATATATATATATATATATATATCAAATTTTCATGTTGTTTGTTTGCCGTGTAATATTAGCTTTAT ATTTCTTAATTAGCAGTGTTCGAGTGGCAAATGAACTTGGAAGAGGAAGCTCAAAAGATGCAAAGTTCTCTATAGTTGTGACAGTGCTTACATCCTTTTC AATTGGTTTTATTTTATTTGTTCTTTTCTTATTTTTAAGAGAAAAAGTAGCTTATCTCTTTACTTCAAACGAAGATGTGGCTACTGCTGTGGGGGATTTG TCACCTTTGTTAGCAGTTTCTTTGTTACTAAACAGTATTCAACCTGTACTCTCAGGTATGCTTTATAAATCTTGGTGTCTTGGTTTAAAGAACTTTTAAT AGTTTAAGATTTGAACCCTATTGTTTTTACAGTCTGAACGACATGAAATTATATGATTTCAATAGGGGTGGCTGTTGGAGCAGGGTGGCAAAGCATTGTA GCATATGTGAACATAGGGTGTTATTACCTCATAGGTATTCCAGTTGGAATAGTGCTTGGTAACATTATTCACTTGCAAGTCAAGGTAGGCTATTATGCTA TACTTTCTTCATTTTCAAATTGCTCTGGATTCCTATTTCATTGATTGGAAAAACTTTCTTCTGTAGGGTATTTGGATTGGAATGTTGTTTGGGACACTGA TTCAAACAATAGTCCTAACTATAATCACCTACAAAACTAATTGGGATGAGCAGGTATTTGATTCATTTTCCTCTCATTGATTCTTAATGTAAGTTAATAT TAGATATTTGATATAGAACTTGATGAAATCATTGTCATTGTGCAGGTAATTATAGCCCGTAATCGTATTAGTAAGTGGTCTAAGGTGGACCTTGACCGTG AAACAGTTACATCAGATAATTAGTTTCAGACTCTAATCTATATCGTCCTTATTTGTTTAGCCGGTGTTATATAGCCAAGAGATTGCTTGATAGGTATTTG AATAAACTATCAATGTAGTCTCCCAAATAGTCCTTAAATTAATTAAATTATAAATTATACAAGTAGTTCTTAAAGTATTGAATGTTCCTTAAGTTAGTCT CTGGATTGATATATTTTACTAGCATTGAGAGACTAATTTGAAGAATTTTTCAATACTTGAGAAACTATGTAGCATGATTTGTAATACTTTAAAGACTAAT ATAGTTTAATTAGTATTTTAGGATAAATGGTATAATACTTCCCGGATAATTGTTAGTCTTATTCAAAGGTGTAAAACTATGTAGCATGATTTGTAATACT TTATAGACTAATATAGTTTAATTAGTTTAAGAAGTACTTTAGGATAAATGATATAATA |
| --- |

>Glyma.19G120900 | Chr19:37879976..37885683 forward
TTCCTCTCATGCAAGAGCAACGATAAGATATATACTCATTCTTTTTTTTATTTTCTGTTTTAATATCAGCCCCAAACTCCCTTGTGAACTGTTAAGTCTC
TGATCGATCTTAAGAGTCCCCAACAACGACATAAGACACAAAAAATGGAGGGGAATCTAGAGAAGAAGCTGTTGAGCAGAGAAAAAAAATCAGAAGAAGA
AGAAGAAGAAGAGAAATTATCATTGGTGAAGAGGGTGTGGGAAGAGAGCAAGGTGATGTGGATAGTGGCAGCACCAGCCATATTCACAAGGTTCACCACC
TTTGGCATCAGTGTTATAAGCCAAGCATTTATTGGTCATATTGGTTCAAGGGAACTCGCTGCTTATGCTCTTGTGTTCACTGTCATCATACGCTTTGCCA
ATGGTATTCTGGTATGTTATCTTGTTTCTTACTCTCTAATTTTAATAATTTTCCTAGGCCAAGCGCTCTCTGTTTTTAACTATGAAGAAGTTGTTCATAT
ATTAAACTCAGGCAATGAATGAATATGATAAATACAATTTTGTCACCAATTACCAGTTCTTACACATACCATAGCCTGGATTCGGAATCTGAAGAAAGTG
TGGCTGGAATCAGATTCTGTGGTTAGTGTGGAGTTAATCAATAAGTATTTTTCCCTCATCTCATAGATATGCTTTTATTCTGAATAATATCAAGGAGTTT
ATAAGTGTGTTAGGAGGGGTTATAAGATTTGGTATGGTGGTGAAGAGAGGAGAGAGAGGAGATTGATTTGAATCTCTTTTACTAACAAAACAAATTAATA
ACTAATATTAATCTATAAAAACAAAAATCAGTATTAAGGTGTCACGTACCCATACAGAAGTTAATTCCGTCCCACTAGTTTGATTTGATTAATCACGTAA
TGAGTGCTGGAATCTGCTTTGGTCAGATTTTGTTGGAGTAAAAAATCGGATTCGTTTGTAATTCGAAGCTTGAGTCTTCAAGAGTTGATAACTTTCTATC
TATCTCTTTATATCACGTCATAACTTCTGTAATATCACGTCATAACTTCTGTAAAATCGGATTCGTTTGTAATTCGAAGCTTGGTCAGATTTTTTATTTT
TTTCTATCTCTTTATATCACGTCATAACTTCTGTAATATCTTTCTTATAAGGAAAAAAAACAAAAAAAGTTAGGTCCAACACAGGTTGGCCTGATCTGTC
AAACTGAAATACGTAATTTCCGCAGAGATTAGTACCGTGTGGACACCACCAAATGACTTTGTGCGTGCTACCCTCCTCGATACATTAATACTTTTCCTTG
TATTCGGGATGAACAGAGATAATAAAATTATGAAGAGTAAATTATAGAAATAGAGAAGATATGTTATTCAGATGAAAAGAAAATTCTTTTCCAAAAAAAG
ATGGATGGAAAATAAAAAAGGAAAGAAGAGAAGTAATTTGGATGAGTAGAAAAAAGAGAAGACAAAAATAAAATAAAAATAATATGAAATTTAATATAAT
TATTTTAAAAATCAATAAATTTATCACTGAATTGTAATTAGATGATTGTGTAAAATTCTTTTTATATTGTTAATACATGACTCTTTTTCTCTTAAAAAAT
ATGCTTTTGAATTTATTATTTTTATATAACAAAATTATATTATTTTTTTAAAAAGAATTATATTATATTTTTAAAAAAATACAAGAAAACGAAAGATAAT
ATTTTCCTCCCATTTCATTTCAATTTGCAAGAAAAGTGTTTTCTAGTAGATATCTGGGAAATAACTTCTTTCTTTGATATTTCTCCTCTCATACAAATTA
TAAAATATAATATTTTTATTCTGTCATTCTCATTTCTTTCCTCTTTATTTCCACCCAAACTAGGAAAGGGATGAGTGCAAGCCTAAGGGGACTGAGAAGT
GAGAACCTACGATGATCCTTAAAGAACTATAGAAAGTAGCATTTGTCATATGTATACAATCATTTTCTTATATTATTATACTTCTCTTATGGAACCTCAA
TTAGTAAGACCTCAAGGAAGAAGACAGTACAATAACCGAAACAGTACATAAATATTTCCAGATATCCACAACAAAGAAGGAACAATATCATTAATAACAA
AGGAAATCAGTTAAGCGGATGAGAGGCAACCTAGTGGCAACCTCAGCAACAATAGGATTTGAATAAGAACTGTAAAAACACACACATGAATACATAGATA
AAATAGTAATAACAACGGTAACAATGATCTGCAGATAATGGTAAGCTTCCATTTTTATTCGAATAACCCTTTTAGAATTTTAAAAATATCATCTTGTATA
AAACAACTGAAAGAAATAGAGATCTCTTCTTTGTCTCGTGAAAAAACAAATTACATTTTCTGACTTTTGTTTTTGGTTTGTTTATATCTATTGGGGTACA
TTATATATAGTAGTGATTTTAGTCGAAAGGAAATCTTGGATTGTAAGAAAACATATACATCAAAAGGTTAATGGGATTAAAGTAAATTAAGAAGTTGAAG
CATATGAACATCTTCTATCACAGCATACAAAATTACGTTACATTGTTGATGATCATGAGTGTAGCTTTCATCGCATAATGTGTATATATACTTCACCTTC
ATGTGGCTATTGGCTACCATTACAATTTATTCAATGAAGAAGTACTGGCAAGGAATAACATAAATTATTTGTGGGTGTCAACTGGCAATTGTAGTTAGGA
ATGGCAAGTGCGTTGTCAACACTTTGTGGACAAGCATACGGTGCAAAAGAATATGACATGATGGGAGTGTATCTTCAAAGATCATGGATAGTTTTATTCT
TAACTGCAATCTGTCTTCTTCCCCTGTTGATCTTCACAAGCCCAATTTTGACTATCTTAGGCCAAGATGAGAGCATAGGACAAGTGGCAGGAACCATTTC
TCTTTGGTCAATTCCTATCTTATTTGCTTATATTGTCTCAAACAACTGCCAGACATTCCTTCAATCTCAAAGCAAGAATGTCATTATTTCATTTTTGGCA
GCTTTATCAATAATCATTCATGTGTCCCTCTCTTGGCTATTCACAATGCAATTCAAGTATGGGATTCCTGGGGCAATGATTTCAACAATTTTGGCATACT
GGATTCCGAATATTGGTCAACTAATATTTATTACATGTGGTTGGTGCCCTGAAACATGGAAAGGTTTCTCTGTTTTAGCATTCAAAGATCTTTGGCCTGT
TGCCAAGCTTTCCATTTCATCTGGTGCCATGTTATGGTGAGTTTTTCTTCTGTCAACTAAAGGGGTTAATAAGCTCTTTATAAATGCCATGATTGCTGGA
TAAATTTGGATTTCCTTTATATAGAAAATTGGATATAGATTAATGTATATGCTTCCTAACTTGGAAGTTGTATATTTTACAGTCTTGAGTTCTGGTACAG
CACAATATTGATTCTTTTGACTGGTAACATGAAAAACGCCGAGGTTCAAATTGATGCTCTATCTATATGGTAATCCTTTGCATTGTAGCATATCTATGTT
GAAATTTATATGTTGAGGTTTTATTTTATGTTGTTGATTTTATCAGCCAGACTTATAGTAGGCTCTTCTCTCTCGACCTAATTAAGATTAAATTTTATTG
TTTACAGTATTAACATCAATGGATGGGAAATGATGATAGCATTTGGTTTCATGGCTGCAGCAAGGTAATGACATCCTTTACCAGTTTACCTTTCAACTGT
TGCAGTCATTGTTTTTGTCATCATCTACGAAATAGTAATTAAATATATCAAGCTGCCATTTAATTTTTCATTTGTAGCTTCTTTGGCGTGGAACATTAAC
TTTATATTTCTTAATTTGTAGTGTTAGAGTGGCAAATGAACTTGGAAGAGGAAGCTCCAAAGCTGCAAAGTTCTCTATAGTTGTGACAGTGCTTACATCC
TTTGTAATTGGGTTTATCTTATTTCTTCTTTTTTTGTTTTTAAGAGAAAAAGTAGCTTATCTCTTTACTTCCAACGAAGACGTGGCTACTGCTGTGGGGG
ATTTGTCACCTCTGTTAGCTGTTTCTTTGTTACTAAATAGTATTCAACCTGTACTCTCAGGTATGCTTTATAATTTTTGGTGTCTTGTTTTAAAGAATTT
GTTAACAGTTTAAGATCTGGATCCTATTGTTTTCACATGATAAAGTCAAAATAATTTGTCAATTGGGGAGTGTGTGTCAAAGTCTGAAAGACATGAAATT
GTATGACTTCAACAGGAGTGGCTGTTGGAGCAGGCTGGCAGAGCACTGTAGCATATGTGAACATAGGATGTTATTACCTCATAGGTATCCCGGTTGGAAT
AGTGCTTGGTAACATTATTCATTTGCAAGTCAAGGTAGGCTATTATGTTATACTTTCCTCATTTGAAATTGTACCTTATTCCTATCTCTTCTCATTCATT
GGACAAACTTTCTTTCTTTGTAGGGTATTTGGATTGGAATGTTGTTTGGGACACTAATTCAAACTATAGTCCTAACTATAATCACCTACAAAACTAATTG
GGATGAGCAGGTATTTGATTCATTTTCCTCTCATTATTCTTAATTGTAAGTTACTATTACATATTAGATATAGAACTTGATGAAATCATTATGTCTTTGT
GCAGGTAATTATAGCCCGTAGTCGTATTAATAAGTGGTCTAAAGTGGAATCTGATCATGAAACAACTACATCAGATAATTAGTTTCAGATTCCTATTTAT
ATCCTCATTATTTGTTTGCCCAGTGTTGTACATTAGATAAAGTATCACTCTCTTTTAAGTAGTCCTTAGACTAATCAAACTATATGAGTAGTCCTTAAAG
TATTGAATATGCATTGATTTAGTCTCTGAAATAATATATTTTACTAATTATTATTGAGGGATTAGTTTGAAGAATTTTTTAATATTTGAGAGAATATGTA
GAATAATTTTTAATACTTTAGGGACTATTATACAATATTATTATTTTAAAAGGTTTCAGGAAAGAGGATAATATTTTAGTGACCAAATTAATGATTTATT
CAATTATTTTATTATTATTTGTTAATTTTTATTGGAGAACACATTTCTTTCATTTTTTTATATTTTATTTCACCCAAGATACAAGTTTATTTATCTGTTA
TATTCCAATTGGCAAGAAAAGAAATTACTAGGCTTATTGTAATAATATACAGAATTGGTCTCTAACTACAAAACCCAAGTTGAACAATATTTGCCTTGGC
TCAATAATCAATATAAACTTTTTCACTCAGGGACTTTGTGACAAATTCAAGTAGAAATGCTTGTATTAATTGTTATTTTGATTTTCACATAAGATAGATT
ATTTAATATAAAATGGTAATGTGTTAGTTGTTACTTATTTAGTCTATTGAATTTAATAAACATGATTTATACTAACATTTCTCTCCTTGACCATTTCCTT
GCACTATCCCTAAATGGAAAGTAAGTTTTGAGGAGAGTCTAGTAAGCTACTAGGAGAATTGTCTCATGTTCGAACCCGGCCTTGCTTTATGGTATGTGTG
TGTATTCTGGTTTGGACTAAATCTACGTATTTTTTTATTTTTTATTTTTTAAGTTTTAGGACCGATATCATATATGTTTAACCAGAAATATAAAAATTAG
AAAAGGCATTGTTTCCATTAATTAAGTATGAATCCAACAATTTAAAAACTCTCAAGATAATGAATGATATGATAAACACTACAAAATAGATTATTTATAT
TTAATGAA

>Glyma.08G244400 | Chr08:21028620..21030099 reverse
ATGGCCTATGGCGGAGGAGGTTTTGCAATTAGTTATCCTCTTGCGAAGGCTCTTCACATAATGTAGGACCATGGAGGAAGAGCTCTTTTGTTTTAGTTTT
ATTTTATTATTATTGTTGTTGTTGTTGTTGTTGTTCTACATTCTGTTTTATCATGTGGCTTATGTGATTTTTTTCTACTGTACTACAACAGGTTCATGCC
CATAGCAACTTGGGTAATCTTATGAAAGCTCAAGGCTAGGTGCAAGAAGTAAGTCCTAACATTTAATAATTTATTGTCATGTTTAAGTGGTATTCTTTTG
AGTTTTGAGGTTTTTCTACGACCTATAATTTTGTTCAAGTGCTTCTTTCTTCTTCTTTTTGGTATTTGGAACTGGAAAGTGTTCATTGTACATGTCAATA
GACAAAGTGGCTCTAAATTCCTTTCAAAGGGGGCTATTGTATTTCTTTTCAGTGGTTTTGGACACAAACTGATGGAGTGGAGGAGACAAAGAGAGGTATA
GAGAGCAAGAAGGTCAAGTGCTATGGTAGATATGGATACTTTGAAGCCAATCAAGTGGTTCACTACATGTATGAACAGACTAATCATGTATTGGACCATC
AATCTAATTCTAACATGTTACAATCTGCTTTGTGTATATTGGTTGTAGGGGATAGATCTTGACATCCAAAATCCACATTCCTCTTCATTCTTGTTGTATG
ACCATAGATACAAAAGTTTCGAGTCTGCATGAAGTACAAGGGTAAAACATGGTTAAATCATTTTCCTCTAATACGCTTTTGGTAGTGCATGTGTAATTCA
GTATTTCCACCAAATTTCTTAGTATATAATGTACTGCAGTTTTTTTTAGTGTGTATAAAAATCTAAATACTTCACTTGCATATGGCTATTGGCCACCATT
ACAATTTATTCAATGAATAAGTATTGACGAGGAATAACACAAATTTTTTGCTTGTGTCAACACTTTGTGGACAAGCATACGACACAAAAAGAATATGACA
TGATGAGTGTATCTTCAAAGATCATGGATAGTTTTATTCTTAACTGCAATATGTCTTCTTCCGTTGTTCATCTTCACAAGCCCAATTTTGACTCTCTTAG
GCCAAGATGAGAGCATAACAGAAGTGGCAAGAACCATTTGTATTTGCTCAATTCCTATCTTATTTTCTTATATTGTCTCAAACAACTGCCAGACATTCCT
TCAATCTCAAAGCAATAATGTCATTATTTCATTTTTGGCAGCTTTATCAATAATCATTCACGTGTCCCTCTCCTGGCTATTCACCATGCAATTCAAGTAT
GGGATTCCTAGGGCAATGATTTCAACAATTTTGGCATACTGGATTCCCAATATTGGTCAACTGATATTTATTACATGTGATTGGTGCCCTGAAACATGGA
AAGGTTTCTCTTTTTTAGCATTCAAAGATCTTTGGCCTGCTTCCAAGCTTTCCATTTCATTTGGTGCCATGTTATCGTGA

>Glyma.03G005300 | Chr03:465179..470215 reverse
GCACTCCTCGTGGATATAATTTGAAGTTCTTGTGTATAGTATGTGTCTAGTTGCTGTCCCTCTCATGCAAGAGCAACGATAAGATACTCATTCTTTTTTC
TTTTTTTCCTTTTCTTTTGTATAATATCAGCCCAAAACTCCCTTGTGTACTGTGAAGTCTCTGATCCATCTTCAGAGGCTCCAACAACAACCAAAGACTC
AAAAAATGGAGGGGAATCTTGAGAAGAAGCTGTTGAGCAGAGAGCAAAAATCAGAAGAAGAGAATTTATCATTGGTGAAGAGGGTGTGGGAAGAGAGCAA
GGTGATGTGGATAGTGGCAGCACCAGCCATATTCACAAGGTTCACAACCTTTGGCATCAGTGTTATAAGCCAAGCATTTATTGGTCATATTGGTTCAAGG
GAACTCGCTGCTTATGCCCTTGTGTTCACTGTCATCATACGCTTCGCCAATGGAATTCTGGTATGTTATGTTATGTTCTTACTCTCAAATTTGAATAATT
TTCCTAGGCCAAGCGTTTTCTGTTTTTAATTAAGAAGCTGTTCATATATTAAACTCATAGGCTATGAATATATAATATCAATTGTTACACATAGCATAGC
CTGAAGGCTGAAAGTGTGGCTGGAATCACTTTCTGTGGTTAGTATGGAGTTAATCAACAAGCTAAGGGTCTTCTTTCCCTCATCCCAGATATGCTTTTAT
TCAGCTGATCGATAATATCAAGGAGTTTTATCAGTGTGTTAGGAGAGATATGATTATAACTTGGTGGAGGAAGAGACAAATGATAGATTTGAATTTTTCT
CGCTAACAATAAAAATTAATAATTAATAACTAATAATTAATATATACTGATAAAAAAAAAATAGTGTGTCAGATTGTCAATCACATACTCATGGAGAAGC
TACTTTCGTGACTGGTTGGTTTGATATGATTGTGTTGGAATCTGCTTTTTGGTCAGATTTTGTTGTTGTAAATGTACGGACTTGGTTGTAATTCAAAGCT
TGAGCCTTCACGAGTTGATAATATTAAAGAGTCTGGCTTGCTTTTTTTCCACTCACAAATATAAATATTTAGTTGACACTTTTCTTTTTTATGTTTCTTC
TTATATCAAATCATCATTTTCTTCCTTTTTTTCTTCCTCTATAAGGTGTCAAATATAAAAATAAAAAATTTCATTAAAAAATTGAATTAATTAAGCTTTT
AGTCCTCAAACTATGAGAATTTTGAATTTTTTATTCCTAGATTGTTTTTTTTTAAATATTTGATACCTAAAGAATTTTCAACTATTATAAATAATCCCCA
TCATTACTAATCTCTCTTTAAGTGTTGAGCTGTCTATTTAAGTGGTCATGAATATCACCATAAATTGTACATGAGAAAGATGAATTGTCATAGATTTTCT
CATGGGTAATGATTGTCCTAGGTTCATGGATGTTAGGAGTGTTAGGATTCAACAATGACTTTTCTTTTTTTCCCTTGCATTGTAATAACATAATATTTTA
ATTATTTACTTGGATATAATAAAACTTACATACTATGTATGTGACATACATGTGATATTTATTTTATCTTATCTCATGACACCTCATGATAACGAGGACA
AAGACTATTTTAAATAATAACAAATTTTATTGACACTAAATTTTTTTTAAAAATAATTTAAAATCCGAAAATTCAAAATCTCATACCAATAAAAACTTAA
TTAGTAAAAAAAAAAAAAAGTTAGGTTGTCCTCATCTGTTTGAAAGGCTTAATTTTCCTTGAGATGAGTACTATGTGGCCACCACCAAGTGACTTTGTGC
GTGCTACCCTCCAAGAAACATTAAAAACTTTGTTTGTATTTGGGATGAAATAGGGATAATAAAATATGTAGAGTAAAGATGAGAATCATAGAGATATGAA
ATAAATAAAATAGAAAAATATGTGTTATTTAGACGGAAAGAAAATAAAGAGGAAAGAAGAGAAAGAATCAGGATAAGTAGAAAGAAAGAAATAAGTTATA
TGAAAAATGTTTTTATACTAGTTATATATATATATATATATTATTTTTATATAAAAAAATTAATTATTATATTTTAAAAAATACAAGAACAAAGAAAGAA
AATATTTTCCTCCCATTTATTTGAATTTGCAAGGAAAGTATTTTTTAACTAGTAGATCTCGCGGGAATAACTTCTCTCTTTGATTTTCTCCTCATACTAA
TTATAAAATACAATATTTTTTATTCTATATTTGTCATTCTTATTTCTCTCCTCTTTATTTCTACCCAAACTAGTAAGGGATAAGTGCAAGCCTAAGGGGA
CTAAGAAGTAAGAACCTACGATGATCCTTAAAGAACTACTAGAAAGTAGCACTTGTCCTATATATATATGTATAAAATCAATCTCTTATACCATACTTCC
CTATGGAACCTTAACTAGTAAGACCTCAAGGAAGAAAACAGTACCAATAATGAAAACACTACATAAATATTTCAAAATATCCACAATAAAGAAGGGACAA
GATTAACACAAGTGATAGCAACTTATATTCTTTAACCAAATAATAAACTTCCATCAAAAGATTTGAATGACCCTTTTAGATTTTTAACAATATCATCTTG
AATACTACAACTCAATGAAATAAATAGTGATCACTTTTTTGTCTCATGAAAAAAAATTACATTTTCTGACTTTTGTTTTTGGTTTGTTTAATTATATTGG
GGTACATTATAAATAGTAGTAATTTTAGTCAGTAAGGAATCTTAGGTTGTAAAAAACATATACATCGAAAGGTTAATGGGATTAAGCTAAATTAAAAAGC
AGAAGCATATGAACATCTTGTATCACAGTATATATACAAATTATTACATTGTTGATGAGTGTAGCTTTCATTATATAATGTGTATATATACTTCACCTGC
ACATGGCTATTGGCCACCGTTACAATTTATTCAATGAAGAAGTATTGACAAGGAATAACACAAATTTTTTGTTTGTGTCAACTGACAATTGTAGTTAGGA
ATGGCAAGTGCATTGTCAACACTTTGTGGACAAGCATACGGCGCAAAAGAATATGACATGATGGGAGTGTATCTTCAAAGATCATGGATAGTTTTATTCT
TAAGTGCAATCTGTCTTCTTCCGTTGTTCATCTTCACAAGCCCAATTTTGACTCTCTTAGGCCAAGATGAGAGCATAGCACAAGTGGCAAGAACCATTTC
TATTTGGTCAATTCCTGTCTTATTTGCTTATATTGTCTCAAACAGCTGCCAGACATTCCTTCAATCTCAAAGCAAGAATGTCATTATTTCATATTTGGCA
GCTTTATCAATAATCATTCATGTGTCCCTCTCCTGGCTATTCACAATGCAATTCAAGTATGGGATTCCTGGTGCAATGATTTCAACAATTTTGGCATACT
GGATTCCGAACATTGGTCAACTGATATTTATTACATGTGGTTGGTGCCCTGAAACATGGAAAGGTTTCTCTTTTTTAGCATTCAAAGATCTTTGGCCGGT
TGCCAAGCTTTCCATTTCATCTGGTGCCATGTTATGGTGAGCTTTCTTCTCTGTCAAATAAAGGGGTTAATGAGTTCTTTATAAATGCCATGATTGCTGA
ATAAATTTGGATTTCCTTTATATAGGAAATTGGATATAGATTAATGTATATGCATTCCTAACTTGGAAGTTGTTGTATATTTTATAGTCTTGAGCTCTGG
TATAGCACAATATTGATTCTTTTGACTGGTAACATGAAAGATGCTGAGGTTCAAATTGATGCTCTATCTATATGGTAATCTTTTGCACAGTATCATATCT
ATGTTGAAATTTATATGTTGAATTCTTATGTTGTTGATTTCATTAGCCAGACTTATAGTAGGCTCTTCTCTCTTAGCCTAATTAAGATTAAATTTTATTA
TTTACAGTATTAACATCAGTGGATGGGAAATGATGATAGCATTTGGTTTCATGGCTGCTGTTAGGTAAATGACATCTTTTACCTTTCTATAGTTATTGTC
ATTGATTTTGTCATCATCTACGAAATAGTATATCAAGCTTAAGCTGCCATTTGATTTTTCATTTGTGGTTGATTTGGGGCGGAATACTAACTTTATATTT
TTTAATTTGCAGTGTTCGAGTGGCAAATGAACTTGGAAGGGAAAACTCCAAAGCTGCAAAGTTCTCTATAGTTGTGACAGTGCTTACATCATTTGCAATT
GGGTTTATCTTATTTGTTCTTTTTTTAATTTTAAGAGAAAAAGTAGCTTATCTCTTTACTTCAAACGAAGATGTGGCTACTGCTGTGGGGGATTTGTCAC
CTTTGTTAGCGCTTTCTTTGTTACTAAATAGTATTCAACCTGTACTCTCAGGTATGCTATATAACTTTTGGTGTCTTGGCTTAATGAATTTGTTTAAGAT
TTGAACCCAATTGTTTATGCATGATAAAGCCAAAATTAATTTGTCAATTGGGGAGTCTGAAAGTCATGAAATTGTATGATTTCAACAGGGGTGGCTGTTG
GAGCAGGGTGGCAGAGCACTGTAGCGTATGTGAACATAGGGTGTTATTACCTCATAGGTATTCCGGTTGGAATAGTACTTGGTAACATTATTCACTTGCA
AGTCAAGGTAGGCTATTATGTTTTACTTTCCTCATTTTCAAATTACACTTGATTCCTATCCCTTCTCATTCATTGGACAAACTTTCTTCTTTTGTAGGGT
ATTTGGATTGGAATGTTGTTTGGGACACTAATTCAAACTATAATCCTAATTATAATCACCTACAAAACTAATTGGGATGAGCAGGTATTTGATTCATTTT
CCTCTCATTAATCTTTAATGTAAGTTACTATTTGATATAGAACTTGATGAATTCATTATGTATCTGTGTAGGTGATTATAGCCCGTGATCGTATTAATAA
GTGGTCTAAAATGGTCCTTGATCATGAAACAATTACATCAGATAATTAGTTTCATACTCCTATCTATATTGTCATTATTTGTTGTACAACCAAGAGATTG
CTTGAAAGCCATCATAATAAAATATCACTCTTTCTTT

>Glyma.14G032400 | Chr14:2353345..2356426 reverse
GCACTGAAACTAGTTGCGACACTTGTATTGCAGTGCACATAATAACCATGAGCTCTTTGGAGCACCAACCTTTACTACCCAGGCTTGATTCACACTCACA
CATTCAAAACTTGTCATCAGATGCCATTGAAGAATTCTTGGAACATAGGCCTATTGCATTGAGATGGTGGTCAAAGCTTATTGTGTGGGAGTCAAGGCTT
CTCTGGCTCCTCTCTGGGGCTTCTATTGTTGTGTCCATTTTCAATTATATGCTAAGTTTTGTGACCTTGATGTTCACTGGACATTTGGGGTCTCTAGAGC
TTGCTGGAGCATCCGTAGCCAGTGTTGGTATTCAGGGTCTAGCTTATGGTATTATGGTATGCCTCTTTATCTGCGCCACTTAACACACACTTTTGAATAC
ATTTTCTATTAGATAAAACTTAAATGCGGTTCCGTAAGTGCTTTTAGTGTTGTTCTTTTTCTATTAGATAAAATTTAAATGCAGTTCCATAAGTACTTTT
AGTGTTGTTCTCTAATAGAATTAAAGTTTCACTTAAGTAATCTGCATTGGTGATCTAGATCCATTTTTTTTCTACATATAGTTAACTTATATGGAAATTA
TAAAATTTGACAAGTCGCACTTACCATTTAACTGACTCTTCTTATTTAATGAGTTTCACTTATAACTGTGTAGTTTTCAATAAATTTCAATCAAATAACA
TAGAATATATTAAAAGAAGTGTCTCATTTTCAATTATGTGCATATTTCTGACGATTTCATTTTGCTTAAATAGCTGGGGATGGCAAGTGCGGTGCAAACT
GTGTGTGGACAAGCATATGGGGCAAAAAAGCATGGAGCAATGAGCATCATATTGCAAAGAGCAATCATCTTACACATTGGAGCAGCAGTGATTCTCTCAT
TTCTCTATTGGTTCTCTGGGGATTTTCTTAAGGCCATAGGACAGTCAGATAGCATAGCCGAGCGAGGCCAAGTGTTTGCACGCGGAATTATTCTTCAACT
CTATGCATTTGCAATAAGCTGTCCAATGCAGAGGTTCCTCCAAGCACAGAACATTGTGAATCCTCTTGCATATATGTCAGTTGGGGTGTTCCTTGTTCAC
ATTCTCCTCAGTTGGCTAGTTATATATGTTTTGGGTTATGGCCTTCAAGGGGCAGCCCTTACTCTCAGCTTTTCTTGGTGGCTTCTTGTCTTGTTTAATG
GTCTCTACATCATATTTAGTCCAAGATGCAAGGAAACCTGGGCAGGCTTCAGTGTTAAGGCTTTCAAAGGAATTTGGCCTTATTTCAAGCTCACAGTAGC
TTCTGCTGTGATGTTATGGTATGACTATATGTCGATGTCCCCATAATTTTCTCTACATGCATGCATACATAAATGAGTTTAATTTCTTTGTGAAACACAG
TTAAAAAATCAGAAATCAAGGAAGATATAACTTTTAAGTAATGATTATTATATAAGTCAACAAACTTTTCACACATAATTTGTAATTGAATGACAATGTA
AAAACATCTTAACCAGTAAGTGCATGACTATTTAGTACATATTTATTTGGATTTCAATTGAGTGAGCTCAAAAGTACTTGTGATCCCAAATCATTAAAAC
CTTCATTCTTCATTTTGTGCATTGGAAATCGTAAATGTGCATCTCAAAATTACTTTTCCAACTTTTATCCAAACATACCCTACATAAGTATACACATCAC
ACCACATTATTATTTACATCAATTAGTTCTACTAATCTAAAAATGTATCTTAATGTAGTTTGGAGGTATGGTACAATCAGGGGCTGGTGCTTTTATCAGG
GCTGCTCTCCAATCCCACAATCTCACTGGACTCTATTTCCATTTGGTATGTTAAAATTTTGTCTTGGAATAATTTTTCTTGTTATGTGCCAACGTGTTGC
CTAACCATAATATCTTTATCCTAATGTGCTTTCAGTATGAATTACTTGAACTGGGACATGCAATTTATGTTAGGCCTTAGTACAGCAGCCAGGTTTGCAA
CAAAACAATGTGTTTTACATTCAAAAAACTTTCTCAAAAGCATGCTAATGGTAGTTTCCATGATTGCAGTGTCCGAGTCAGCAACGAATTAGGAGCATCA
CATCCAAGAGTAGCAAAATTTTCTGTCTTCGTAGTGAATGGAACCAGCATCCTCATTAGTGTGGTTTTCTGCACAATTATCTTGATATTCCGGGTTTCTT
TGAGCAAACTTTTCACTTCTGACTCTGACGTCATTGATGCTGTGTCTAATTTGACTCCCTTGCTTGCTATTTCTGTTTTCTTTAATGGCATTCAACCTAT
ATTATCAGGTAATAAAGCTATAAAAAGTACACTAGAATAAGCACCAACTTTGGTTTATCACACTTTTGTTAATTTTTCTTGGAAAATTTCAGGGGTGGCA
ATTGGAAGTGGATGGCAAGCATTAGTGGCTTATGTAAACTTGGCTTCTTACTACGTTGTTGGTCTTACTGTTGGATGTGTTCTTGGCTTCAAAACTTCTC
TAGGAGTTGCTGTAAGTTTGGAACATTTCTGTCACTAATTTTTAAGTTTGTAACTGATTAGTTTCATGCAATGCATTTTGTGGTATTTTTTTAGGGAATT
TGGTGGGGAATGATCCTTGGAGTTCTTATACAAACAGTAACTCTAATAATTCTGACTGCCAGAACAAATTGGCAAGCAGAGGTATCAACACTCTACATTA
GAAGTTCTTATACATGTTACATTACATGAATTTTTTGTCACATGTGAAGTGCTGAACCAACTCTTAATGCCTTTGTAGGTTGAAAAAGCTGTTGTTCGCA
TCAATAAATCCGCTGAAAATGACACCTTAGATCAACTGGTTGCTGACACTTAGAGGCATTCGTGTTTTTTCTTCAGGCTTGGTTGAAATGCATTTTAGCT
GCACAGGAATCTTGCTTTGGACTAAAGGAATGTTGTTGATGGGTTTCTTCACCCTATTTAATTTTATGTTTTGTTCACAATTTTGAGAATCTAATAATGA
AACCCAACTTTCACTGCCAGCATAAATATTTGATATATTATTGCGTGGTTGGATATGTGTTTATACAATTTATTATATCTAA

>Glyma.06G305200 | Chr06:49420013..49426780 reverse
ATGGATTCTACATCAGTTCACAAGGACATAGATGAACCTTTGCTGGTCTCAGAAGAACCTTCATCACCACAACCACCTTCTTTTACTCAATCATTCAGCT
CAAGGCATGGATCAGATGGTGAACTTGAGCGAATACTCTCAAACACCAGCGTCCCTTTCGCGAAGCGTATTGGACCGGCAACATGGGTGGAGTTGAAGTT
CTTGTTTCACCTTGCTGCTCCTGCTGTTATCGTCTACCTTATCAACTATGTCATGTCTATGTCCACACAAATCTTTTCAGGCCACCTTGGTAACCTCGAA
CTTGCTGCTGCTTCTCTCGGAAACACTGGCATCCAAGTCTTTGCTTATGGCCTCATGGTAGGGTACTACTTACACACCCTGTTTCCCTCCATCTCACGCT
TCCTAATTATGTTCACAAATTTCATTGTGTTTCTTGATATTAAAATGAAATTATGAACAACCGCGATCGATGCAACCACAGTTGCAGTCTTAGACACTCC
AAAAACCTTGAAGTCGTACAAACCGTTTTTTCCAAAACCTTGATTATGTTCTTGAATTTATGTGCCACTCATATCATCATGCTACTATATTCACAATCAG
TCCAAATATTCTGAGATTTTATACTAACCATGCATAAAATTAGTCTCTTCAGAATGAGAAAATAGTATATACATCAGATTTTACATTATCATTCAATTAC
AAACTGTCATATATTATAAATTTGTTAATCACTTATTATTTTTACCATAGTTTCTGATTGATTAACAAATGTATAGACATTAAACTCATTCATTCATAAA
GAGTATATATATATATATATATATGAATTTAATTAAAATTAGCATGTTTTTCCAATAATTCACTCATGATTTTCACAGTTGGGAATGGGGAGTGCAGTTG
AAACACTATGTGGACAAGCATACGGTGCTAAAAAATTCAACATGTTAGGCATATACTTGCAAAGATCAACGGTGCTTCTCACACTAGCAGGCATTCTTCT
AACCATCATATACATCTTCTCCGAGCCCATCTTAATCTTCCTAGGAGAATCCCCAAGAATCGCGTCGGCAGCAGCACTTTTCGTCTACGGCCTAATCCCT
CAAATCTTCGCCTACGCTATAAACTTCCCAATCCAAAAATTCCTCCAAGCTCAGAGCATAGTGGCTCCAAGTGCATACATTTCAGCAGCAACGTTGTTGG
TCCACCTCGTATTAAGTTATGTTGTTGTGTATAAGGTGGGGCTTGGTCTGTTGGGTGCATCTTTGGTGTTGAGTGTTTCGTGGTGGATCATTGTGATTGC
GCAGTTTGTGTACATTGTGAAGAGTGAGAGGTGTAAGCACACGTGGAGAGGGTTCAGTTTTCAAGCATTTTCGGGGTTAGCAGAGTTTTTTAAGTTGTCT
GCAGCTTCAGCGGTGATGCTGTGCTTAGAAACGTGGTACTTCCAAATTTTGGTTCTGCTTGCAGGATTGCTTCCTCACCCTGAGTTGGCTCTGGATTCTC
TTTCTATTTGGTGAGTCATTCTCTTCACATATACCACTACGAGTCACTCATACCAACACATGCAATATCTAACACACGTACACCTTCAATTATGTCTAAT
TTCATTCAAAATTATAAAATCTGAACACCCATAATTAAAATTTGATGTTTTTCAATAAATTTTAATTAATTAAAAAAATATATTCAAAGATGTGTGATAA
AGTGTGTGTTTCTAGCATTTTTCATGCTACTATTTTCTAAACACTTCCAATAAGTGTTAGAAAATCTTTAATATGTTTACTTTTTTCTTCTTCTTCTTTT
CTACCTAACCTCTATTTCTTTTTATTTTTTTATCACGTTTATTACATATGTGATTTTCTCTTTCTAAATTTATTTATTTTATAAAAAAAATGTTTTTAGT
TTGTATACTTCGATTAAACTTGGTTGACGTGCTTGTGCTTTTTTATTGATAAATGTAATTAAATTTATATTTATCAATACAAAAGTACATGGAGAGGGAT
GAAAAGCTGTATAAAAATTGAAAAAAGAATAAGAAGATTTAAAAGGAAAAGAATTCATTCGAAGTAGAAAAGTTGGGATGATGTCTGGATTATGTATAGG
AAGAGTCTAATTCTTACCTTTTAGATTTAGGAAAAAGAATCTAATTAAAATAATAATGGTGTTTGTCAACATTAAATAAAAATTAAAAAATTGTGATAGT
AATATAAAAGTAAAAAAATAAATGGAAGAAAAAGTTGTATTTGAATCCAATTCCAATTTGTAGGCCATAACTAATCCAAGGATGAAATAGCCTGCCACAT
TGCAACATAATTTGGCTTATGCTTGTTGTACTTATTGAAATTTAAAAACAGAAATCTTCATGGAAAAATTATTGACCCTTATAAAAAGTAGAAAACAAAA
CCCAGTTTTCAATTACGTCACAAATGACTTGTACTTGTCGAGTAGCAACCAAGTAAGTAGTAGTGCAAGAAAGAGATGGGGTTGGTGTCCAAGGACAAAA
TCTATTCCTCATATTGTGCTGCAACCTGCAATATGCAATAGTGACAACACAAAGTCTTTCCGTCGTACATTCTGATATTTTTAAATAAAAAAAATTACAT
TATAAGTTTTATTTAAAGTATTATACACTAAATTATAAAATGATGAGTTAAGTAATAAATTTTATGAAGTTATAAAATTTCATTAATTGGTTTTGTAACT
TAAAAAAATATCATAATACTATTTTTGAAAGGAATATCGAAATGAATTCAATATTCTCTTAATTTATTTTGTTAATAAGCTTTTAGTTATTAAATAAAAA
TAGATATCAAATATTTGTATAGTTCAAAATATTAATCTTATTATTTATTTTATTAAGTATATATAGACATATTTTTATTTTAAATATAGCATTGATGTAT
CCTTAAACGTTACCATTCATGCACACATTTGGTAGTGTCATAAGAAATGAGATTTCAATATTAAGAGAACAAAAAAAAAAGGGAAATAGAGGTTAGGGTT
ATATTTGATAAGATATTTTAGTTAATTTTTAATTTTTATTTATTTATTTAAAACCTCGGTTGTCTGTTTAATTATAAATAATTTTTTTAGTAACTTTTTA
ATATTATTTGAAATAGTATTTTTTAAAATATTAGTTTCTAATTTTTTATATTTCTTTATTTTTTATCTTTAATATATTTATCAAATTTCATGGTTTTTTT
TAAATATATCATGATTTTATTATTTTTTATTGTTTTACACATTTCAACTACTTTAATAGTTAATTTTACTAAATATTTATAATTTAATAAGTTATTTTTT
TAATTTTTAATTAATTATTAAGTTTTTAGTTAATTTTTTTGAACATAACTTACATCATCTAAATGGCCACACCTCTTAAAATTATATGTATAATGAGTTA
GAATAAAATAACTTTATTACTATTTAAATGTTTATTGTAGAATATTTTTTTAAATAAAAATATAATATAAATTTGATAAGCATATTTTATCGAATTTTGT
TTAACACTAACTTATTTTTTAAATATAATCTCGTAATCATAATGTTATTCTAGTTTCTTTATTTTTTCAACTTTTAGTATTTTAAATCTAATTTAAACGA
GCATACAATAAACATGTTTAGTTTCTTATTTTATAACTTAATTCACGAAAAAAATTCCATAAATGAATTTTCACCTAAGTCAACGTTTTAAGTTTTTCAA
ATGAGAATAAAAAAGTAGCATGATGGTGTTGCGTTTCGAAACAAATCTCCTCATATCAACTCGGGACTCAACTCGTGGAATTATTTAAAATATTATTTTT
TCTTTTAGGGTGTGTTTAGAAGAAAAAATAAAAATAATATTAGAAAGAAACTGTGTATTTTTAGTGGGACTCATACGTTGATACTCTATTTTAATTAAAA
AATATATGCATATATGTGGTTTATGTATCCTTATGTGTCACACTCAAATTACTGTAGGTTAATTAAGTTATCTCTGTTGTCTCTTGAATGTAACACTTAG
TCATATCTTGATTAATCTAATTAATGTTCCTGCATGCAACACTAGAAAACGATTATTTTTAATAAAATAATCGTTTATATTTTTAAACTTTTTTTTTTTT
TACAAAAAAGAAGGTGATTATTTTTCTAAAATAAAGCAATTGGTTAAGCAAGTTTTAATTTCTGTTTGATTAACATGTATTTGTTATTGCAGTACCACAT
TTTCTGGATGGGTTTTCATGATCTCAGTTGGATTTAACGCAGCTGCAAGGTTTGATACTCCTTTCTATGGAACTGAAGTTTTGAAATTTTTTCTTTCGTT
TTGCTTTCATTTTAATGCTGTTTTTTTATATTTTAAATTACATAAGCATTTGTTGAAAAATACTCATGTTTATTCTCCTGTGTGCCACGCTGGTTGTCAC
TGTTGTGTTGATGATGAAAAATATTCGTTTCTATGTTTGGTAGAAAGTAATGCACGTTTTGACTATTAATTGACTAATGACTACGATTAATTGTACATCA
TCATTGACCAAATCTAGTGACGAACAACATGTATAGAGGAGGATAATCAATTTCCATAAATTAACATCTACTGATTAGTATTCCATATAATTGAGAGATA
GTGAAAAAAAAGTCAATAATTAACACCCAAGAAACTATCATGCTCTTAAAGGCATAAAGAGAAAATTCTATACTAAAATATATTTTTAGCTAGGTTATTA
CATTATTAAAATACATGAATTGATACAAAATTATTTTAATTTAATTAATGATCCAAAATTCGAGTCATGAATATAAAACTGATATAATTACCATTGAAAA
AAATTGAAGGAAATTAGGATGACATGATAATAATAAAACATGAGTATATGTTTTAACTATTCTTCAGTTGTGAATACACATTACGCCAAAACATGTCCCT
CCTTGTTTCTCCGTTTTTATATGGGAAAGTGAAAAACTTTACGCCAAAACACTTAGTCCTTTAACTGGGTTTTGAACTTATTCCTACGATCCTTTATAAC
ATTAAGTAATTTTTTGCAAAGTAATTTTTGCATTAGATCAATCATTTTATACTAGATTATATTATCAACTTTTTTTATAACAAAAATATTTAAATATAAA
TCATATCATTTTAAAAATTAATTTTAAGCAAAATTAAATTTGTTAACGTTAAATTACACGTATATCATGTCAATAATTTTAAACATTATTTTTGTCTGTC
ATGAATGCAGTGTGAGAGTGAGCAATGAACTAGGGGCAAGAAATCCAAAATCAGCATCATTTTCGGTGATGGTGGTGACATTGATTTCTTTCATAATATC
GGTGATTGTAGCACTTGTGGTGCTGGCAATACGAGATATCATTAGCTATGCTTTCACAGACGGTGAAGAGGTGGCTGCTGCTGTCTCAGATCTTTGTCCT
CTCCTTGCTCTTTCTATTGTTCTCAATGGCATTCAGCCTGTCTTATCTGGTAATATATTCTTTTGACTTTGATATATAATAATATTCATTCCAATCACTA
CAAATAAAAATGTTATGCACATGAATGATTGGATTAAACCATTGATGTAAAAACTATACAGATAATTTCATGAATCATGCATATGACACGAAACCATTGT
ATGTCTTGGGGTCTAGCCTGTACTATATCCTATAAACAGACAATATTATTAGTTTAGAAAATATGTACAATTGATAAATATATAGTATCACATTGTTATG
TATAATACTATCATTGTATATTTATAATATTTATACGACATAAGTATATTTTTAATTATTACAAAATGAATGAATCATGTAGTATAATTATTTATTTTTG
TAATAATTGAATACTTTAAAAACGACATTTGAGGTGTTTAATTTCTAATTGCATGGTTATAATAACGTAAACTCATAAAGGAATTTACTTACTCCAATGA
CAAATTTAAAGAATTACTATCGATCTCAGTTCTAAGTTTTAAAATTATTCTTTCACCGTCAGATTTTAATTTAAATAAATGAATGATTGAGATTAGAAAT
AACCCTTTAAACTTATTCAATTAGTACTAAATCCCTCCCCATAAATTACATTAACGAAAGTTAAAGTTTTCTGTTAACATACGACATTTTTTTTATCAAG
GTGAATGCTAGGAATTTCTGAAACTAATGCATGTATTATAACGTATCTAAAGGGGTGGCTGTTGGATGTGGATGGCAAACTTTTGTTGCATATGTCAACG
TTGGTTGTTATTATGGAATTGGCATACCATTGGGTTCGGTTCTCGGTTTCTATTTCAAACTCAGTGCTAAGGTATGTTACTCGATTGGTGTTGTATACCT
TTCTCATCCAAAAGCTAGCTCTGATTTAATAATGCAAGAAGAGGTATATGCATATATTTAATTTGCATGCACGTATTGAGGACAGTGTCTGTATGCAGGG
AATATGGTTGGGAATGCTAGGTGGCACGGTTTTGCAAACAATTATTTTAGTGTGGGTCACATTTCGAACGGATTGGAATAATGAGGTAAAAAAAAAAAAG
AAAAGAGGTTGTATATGTATATGTATGAAGGGAATGAGCTATATAGTAATTTCTAATCAATATTTCTTGATTGTAGGTTGAAGAAGCAGCAAAGAGGTTG
AACAAGTGGGAGAACAAAACAGAGCCACTTGTGAACTGAAGCTCCTTGTTTCTCAATGTAGCTTAGTCTCACAATTATACAATAATACAAATCAGGCTTT
ACATCTCTGATCACAACTTTTTGTTGTAAATAAGTTATATATAACATTTGGCTAAATTGATAATTTCA

>Glyma.12G099300 | Chr12:8610079..8616394 forward
GACAATATATAATCCCATCCAATCCTTTTGTAGTTGAGCCTCAAAACCTCCTCTGCTGCAGAGGGAAAAAAGGCTTAGTCCACAACATTTTCCAAACATA
AACAAGTTAGTTCCCAAATGGATTCTACATCAGTTCACAAGGACATTGATGAACCTTTGCTGGTCTCAAACGAACCTTCACCCGAGCCACCTTCTTGTAC
TCAATCATTCAGCTCAAAGCATGGATCAGATGGTGAACTTGAGCGAATACTCTCAAACACTAGCGTCCCTTTCGCGAAGCGTCTTGGACCGGCAACATGG
GTGGAGTTGAAGCTCTTGTTTCACCTTGCTGCTCCTGCTGTTATCGTCTACCTTATCAACTATGTCATGTCCATGTCCACACAAATCTTTTCAGGCCACC
TCGGTAACCTTGAACTTGCTGCTGCTTCTCTTGGAAACACCGGCATCCAAGTCTTCGCTTATGGCCTCATGGTACAGTACTACTCACCCTGTTTCTCTCC
ATCTCATTGCTGCTAAATTATGTTCACAAGTTTTAAAATTATGGTCGCAATCGCAATTTAATCGTGTTTCTTGATATTAAGTGAAATTATGAACCAACGT
AACAGATGTGGCCACAATTGCAGTCTCAGACACTCCAAAAACCTTGACGTTGCATTTGAAATGACGTTTACAAACCGTTTTTTAAACATTATGTTCTTGA
ACTAATGTACCACTCATCATGCTATATTCTCAATTAGTCCAAATATTCTAAGATTTTAGAATGATCTTGCATAGAAATTGATTTCTTTAGATAGAGAATA
TAGTATATATGCACCGGTGTAAAAAGATTTTTAAGTTGCCATTTAACTACAAATTATCATTGATAAGTTTGTTAAATAGTCATTTCCAGGGTCAATTCAC
AAATATTTTAAACTCATTCATATATAAGTAGGGAAAGACAATTGGTGTTGGGGGTGTATGAATTCCAATAATTCAGTTATGATTTTCATATTTGGTGATG
TTGTCGAACAGTTAGGAATGGGAAGTGCTGTTGAGACACTATGTGGACAAGCATACGGTGCCAAAAAATTCGACATGTTAGGCATATACCTGCAAAGATC
AACGGTGCTTCTGACGCTAGCAGGCATTATTCTAACCATCATATACATCTTCTCCGAACCCATTCTAATCTTCCTAGGAGAATCCCCAAGAATCGCATCC
GCAGCAGCACTTTTCGTCTACGGCCTAATCCCTCAAATCTTCGCTTACGCTGTAAACTTCCCCATTCAAAAATTCCTCCAAGCCCAGAGCATAGTGGCTC
CAAGCGCATACATTTCAACAGCAACGTTGTTGGTCCATCTTGTGTTGAGTTATTTTGTTGTGTACGAGGTGGGGCTTGGTCTGTTGGGTGCGTCGCTGGT
GTTGAGTGTTTCGTGGTGGATCATTGTGATTGCACAGTTTGTGTACATTGTCAAGAGTGAAAAGTGTAAGCACACTTGGAGAGGGTTCAGTTTTCAAGCG
TTTTCGGGCTTGCCAGAGTTTTTTAAGCTGTCTGCTGCTTCGGCGGTGATGCTCTGCCTAGAGACCTGGTACTTCCAAATTTTGGTTCTGCTTGCAGGGT
TGCTTCCTCACCCTGAGTTGGCTCTGGATTCTCTATCTATTTGGTAAGTTGTTTTTCTTCACATACACTATTTTCTAAACATGTCAGATTAATTATAAGG
AAAATGAATTTTTTTTTTATTCATGACATTAGAATGGAATATCCATTAATATTTGATCACATTTAGTGGATCTTTCATTTTAATTTTTTTTCATTCATAA
AATTCAAATTCAAATTCAAATGTTCAAAGAGATCTATAGGATTACTTTTACCTATTACTTATTAATACTTGATAAAAAAAATAAAAATAAAGATGCATGT
GATAGTCATAAATGATTTTATACTTTTATACAGTAATAAATCATGAAAAGTTGTAACTCATAATAACAATAATTTGTGACGGATGATAATATAAAATATT
TACACTTTAAAACTCTTTAAAATTATTAAACATATATTCACTTTCTTTCCTATCTCAATTCTATTTTACCACTTCTGTGTTGGAAGCAGGATAATTGTGT
GAATAGAAACGTGAGAAACCATGCCTGTAGCAACTTTTAGTTGCCATAACATCGGGAATATATTAGATTTTTCTTTTTGTCTTTTGTTAGAACCATCCTT
CCAAAAGTTTGAATGGAGAGGGATAAAGAGCTGTATAAGAATTAAAAAAAAATAAGAATTAAAAGGGAAAGGATTCATTCAAAGTAGAGAAGTTGTAATG
ATTTTGGATTATGTATAGGAATAATCTAATTCGTTCCTTTTTGGTTTAAGAAGAAGAATCTAATTAAAAAAAAAAATTATGTTTGTCAACATTAAAATGA
AAAACCAAAAAATAAAATAATCGTGATAGTAATATAAAAGTAAAAAAAATAGATATAAGATAAATATAAATGAAAAAGTTGTATTTGAATCCAATTCCAA
CTTGTAAGCCAGGGATTCTATAGAAACCATAGACCACAACCTTTGTGGAGTCTAAATAGTATGTCACATTGCAACATATTTTGGCTTGTTGTACTTTATT
TAAATTTAAAAACAAGAATCTTCATGGAAATTTTTTTGACCCTTATAGGAAGTAGAAAACAGAACCCAGTTTTCAAGCACGTCATAAATGACTTGTATTT
TGTCGAGTAGCAATCAAGTAGTGCAACAAAGAGATGTTTTTGGAATCCTAGGACAAAATCTATTCCCTTAAACGCCGCCATCCAGGCACGCATTTTTTAG
TGTTAAACAAACAATGAGATGAGATTTCAGTATTAATATGAAAAAGAAGGAAATAGGGATTAGATCATCCAAGTGGCGGCACCTCTTAAAATTATATGTA
TAATGAATTAGAATAAAATCACTATATGATTATTTAAATGTTTATATAGCATAATATTTTATTTTATTATTTTAAATAAAAAATAATATAAAATTGATAA
TATATTTTGTTTAACTCTAACTTATTTTAAATATAATCTCGTAATCATATTGTTATTTTGTTTTTTTTTTTTCATTTTTAGCATTTTAAATATAATTTAA
ACAACGTATAATAATCATGTTTAGTTTCTTAATTATTCAATAAATTAATTCACGATTTGAATAAAAAATGCAAAAATAAATTTTTCACCCAAGTCACGGT
TTTACGTTTTCCAAATGAGAAACGAAAAATAGCATGATGGACGATGGTGTTGCGTTTCGAAACAAATCTCCTCATATCAACTTGTATGTGGACTGTACTC
GTTGGGCTATTTAGAATATTCTTTTTCCTTTTATTTAGCCTTTGGGTCCATCAAAATGTATGCCTATGTGGTTTTTGTGTCCTTATGTGACACTCAAATT
ATTGTAGGTTAATTAATTATCTCTGTTCTCTCTTGAATAATAACACTTCGTTATATCTTGATCAATCCAATTAATGTTCCTGCACGTTAGAAAACGATTA
TTTGTAAGAACATAATCGTTTATTTTTAAAAAATTTACAAAAAAGAGTGTGGTAATTTTTCTAAAATAAGGCAATTGGTTGAACAAGTTTTAATTTCTGT
TCGATTAACATGTATTTCTTATTGCAGTACCACAGTGTCTGGATGGGTTTTTATGATCTCAGTTGGATTTAACGCAGCTGCAAGGTTTGATAATCCTTTA
TATAGAACTGAAGTTTTGAATTTATTTCTTTCGTTTTGCTTTTATTTTAATGCTGTTTATTTTGTTTTAAATTACACTAGCATTTGTTGAAAAATATTCA
TGTTTTAAATAGAAAATAAAGAAAATTCTGACAAACATTTTAAATATACAAGTACTAATTAAAAAAAATATTTTTTGGGTTATTATATTGTTAAAATAGT
AGATCAGTTAATGTAAAATTATTTTAGTTTAACTAATAATTTTGAATTCAAATTCTAATTAACTGATATATGATTGAAAATTGTTGAAAGAAATTAGGAT
GACATGATACCAATAAAACATGGTTAACTATTCTTAAGTTGTGCTTGAATACACGTTATGACACAGTTGAATGTCAAAGCAAGTTCCTTCTTATTTCTCT
GTTTTTACATGAGAAAATAAGAAACTTTCGTTCAACGTGTGGTTGGAATAGTTTTCCAAACACACTTAATCCTTTAACTGTGTTTTGTACCTACTCCTAA
AATCCTTTATAACATTAGATTTATTAGTTGCATTTTCACATATTTAATTCTGAGTTGATAAATTAAATCTAAGTTTAATAGTGAATTTGAGTTGAAGCAA
ATTTAAATAATTTTTGCATTAACTTTTTATACTGAATTCTATTATCAACTTTCTTTTATAACAGAAATATTAAAATATAAATCACATTATTTTAAAAATA
ATTTTAAATAAAATTAACTTTGTTAATGTACTATTATATATGTAAATTTTAATAATATTTTTTTGTCTAGCACTTAATAAAAATATTGTTAAAAGTCTTT
TCAAAAAATATTGTTAAATTATTAATAAATGTTACTAATATATTTTTTGTTATTTTATCAGATGTTATATAAAATCTAAAAATATCATAAATTTTTTTTA
ACAACATGAATTATACGTAATATTTGATAAATATTAAAAAATATATTAATAATATTATTTATGATTTTGTCACATTTTTAAATATTGTCAAAAAAATTTA
ATAATATTTATATTAAGTGTTATTAGATAAAAAAAAAAATGTTGTTAAAGATTATATTTATAGTAGTGATGTTCATTGAACACATAACATGTCAATAATT
TTAAACATGATTTTTTGTGTGCCATGCATGCAGTGTGAGAGTGAGCAATGAACTAGGGGCAAGAAATCCAAAATCAGCGTCATTTTCGGTTGTGGTGGTG
ACATTGATTTCTTTCATAATATCAGTTATTGTAGCACTTGTGGTGCTGGCAATAAGAGATGTCATTAGCTATGCTTCACAGACGGTGAAGAGGTGGCTGC
TGCTGTCTCAGATCTTTGTCCTCTACTTGCTCTTTCTATTATTCTCAACGGCATTCAACCTGTCTTATCTGGTAATATCTTCATTTAACTTTCATATTCA
TTCCAATCACTACAAATAAAAATGTGATGCACATCCATGCATGGTTGGATTTGCCTCTCTTCCGCGTGTTTTTTTATCCAATAAGAATTAAACCATTGAT
ATAAAAACTATATATAGACAATTTCATTTTCATGAATCATCCCTACTTACACGAAACCATTCTATATCTAGGGTCTAGCTACACTATATCCTATTAACAG
ACAATATTATTAGTATAGAAAATATGTACAATTTACACACACATTTTTAATTATTACAAAACCATTGAATCATGTGTGATAAATTAATTTGTTTATTTTT
TAAATAAATACTTTAAAAACTATACATGATTTAATTAATTTCTAATCGATTAATAATGCAAACTCATAAATTATGAGGGAGTAATTCTTTAAACTTACTC
TTGGATTAAGTAGATTCCTTCTCTTATTATTTGTAATGCATTAATTCATTATAACGTAATGTAAAGGGGTGGCTGTTGGATGTGGATGGCAAACTTTTGT
TGCGTATGTGAACGTTGGTTGTTATTATGGAATTGGCATACCATTGGGTTCGGTTCTCGGTTTCTATTTCAAACTCAGTGCTAAGGTATGTTACTCGATT
GGTGTTATCTATCTTTCTCATCCAAAAGATAGCTCTGATTTAATAATGCAAGAAGGGGTATATATTTAATTTGCATGCACGTATTGAGGACGGTGTTTGT
GTGCAGGGAATATGGTTGGGAATGCTAGGTGGCACGGTTTTGCAAACAATTATTTTAGTGTGGGTCACATTTGGAACGGATTGGAATAAAGAGGTAAAAA
AGAAGAAGACTATAAACAACCTTCTTCTGTTGTTCTGATCATTCTACTATGTAATGTGTATGAAGTTAACGAGCTAATAATTTCTAACCATAATTTCTTG
AATTAATGTAGGTTGAAGAAGCAGCAAAGAGGTTGAACAAGTGGGAGGACAAAACGGAGCCACTTGTCAATAACTGAGGCTCTTTGTTTCTCAATGTAGC
CTAGCCCACAATTATACAATAACACAAATCGGGCTTTAGATCTCATAGCTTTTTGTTGTAAATAAATTGTAAGATGAGGCCAAATTCATAATTTCACTAC
CAATTTGTTGGTCTAACCCGAATAGTTTGCGTAGTGTTAGTCACGTTGTGTTCACATGTTGGGAGAACAAACATGTTATAAAAATGATCTATTTAACAAT
AAATTTCGTATTCATC

>Glyma.12G194000 | Chr12:35567230..35571578 forward
TGAGTTCCGCCTCCTAGTGGTTGTTGAATATAATATAATATAATATTCTCGTTATATGTGTATATATAAGAGATGGTGTGCCTTACGGCTCCTCGTGGTA
GGTGAGTTGGGACTTGTGGCGGCGAGATTGCTGCTGCTCCAAACGTTACAGGAGAGAAAGCCTTCTTTGCCGTGAATTCGGATCGAGATCAATGGGTTCA
GCGGAGAAAGAGCCACTGTTAACAGCGGAGGGAACTCGATCAAACTCAAAGCACGAGTCCTCCTCCCATGGGCAACTTGAGAGAATTCTCTCCGACGACA
CCCTACCCTTCCTCAACCGTGTGGGACCCGCCACCTGGATCGAGTTGAAGCTCCTCTTCTTCCTCGCCGCCCCTGCCGTCATCGTCTACCTCATCAACTA
CCTCATGTCCATGTCCACCCAAATCTTCTCCGGCCACCTCGGCAACCTCGAACTCGCCGCCGCCTCCCTCGGCAACACCGGCATCCAAATGTTCGCCTAC
GGCCTCATGGTACCACCATTTTCTTTTCAAGTGAATCTCTGCTGATAATTGCGCGTAATTATTGAAAAACACAAAATTATATATATTACTTGCAGTTGGG
TATGGGGAGTGCCGTGGAGACGCTATGCGGACAAGCATTCGGAGCTCAAAAGTACGGGATGTTAGGAGTCTACATGCAGCGGTCAACGATTCTTCTCTCA
CTAGCCGGCGTAGTGTTGACCGTGATATACGTATTCAGCGAACCGATGCTAATATTCCTCGGGGAATCACCGAGAATCGCGTCGGCGGCAGCACTCTTTG
TGTACGGGTTGATCCCACAGATATTCGCATACGCGGCGAACTTCCCGATCCAGAAGTTTCTCCAAGCGCAGAGCATAGTGGCACCGAGCGCGTACATATC
AGCGGCGACGCTGGTGGTCCACCTGGGGATGAGTTGGGTGGCGGTGTACGAGATTGGGTTGGGGCTGTTGGGGGCGTCGCTGGTGCTGAGCCTGTCGTGG
TGGATAATGGTGATTGGACAGTACGTGTACATAGTGAAGAGCGAGAGGTGCAGGCGCACGTGGCAGGGGTTCACGTGGGAGGCGTTTAGCGGTTTGTATG
GGTTCTTCAAGCTCTCCGCTGCGTCCGCGGTCATGCTGTGCCTGGAGACATGGTACTTTCAGATTCTGGTTTTGCTCGCGGGGCTTCTCCCTAATCCTGA
GTTGGCTCTTGATTCCCTTTCTATCTGGTAACTTCTTTTATTTCTTACTTTGCATCTCTCATCGAAATTTCAAATCCATTCATATATAAAAATATACAAC
CTTCTCCAGATTCAAAATATATCAAAATTAACACTTTTTTTCCCCATTATATCTAGCTTTTCAGCATTTCTTATTTAATTTAAGCAGCATATACCCCAAA
ATGAGGTGTGAAATAATCACCATTCAAGTACAATAAGCTGCTTTAGTTTGTGCCATTGTTTGTTCGGAGAAAATAGAGAATGAAAAAAATATGATAGAAA
CATTCTATTTTCATTCTTTGTTTGAAAAAATAAGGATATTAATTATTGTTTTGGAATTCTTAAGAAAAAAAAGGTTTAAAAGGGTTCTTTTTTTATATAA
ATAAATAATTTTTTATAACATGTTTAGTTTTTAAATTAATTTTTTCGTCTAGTCTTAGAAGATTTTTATATAATTTATTTATTTTTCTACTCATCTATGT
AAATTAAACAGTTTTTTTTTTATCTTTCTCTTTATCTTTTTCAATTCGCTCAACCAAATTAGACGGGCACATATTCCTTTTGGTTACCACCGTTTTTGGA
TGCTCACAGAAGCCATTTAGGGAAAAGAAAGGAAAAATAAATAAATTTAAATGGAAGAGTGATTCTTATTTTATTTTTAAAGCTATTTTTTCTTTCATTT
TCCTTTGAATTAAAAAAATATCGCAATCACACTTCTAAAATAAATAATCTTAAAAGAGAAAGAACAAAAAGATAAGATAAGAAGAAAATAGGAGAAGGTG
TTCTGATGTCATGTTACTTGTCAGTATATTTTCTGTCCTGATGTGATTTGAAGGAGCATTCAAACCAGAAATCACACTCTGAAATCCAAACCCAAATATA
AAAATAAATAAACAAACGCGTGCGACTCATGTGAATTATTTAGTCATTGGACCCCACAAAGCAAAATGACTGACTGCCAACTCAGATGAGTTATCCGGGC
AGACACTTTTGAACGTGACGTAAGGAGCATCAGAATTTTGCTACAATTTGATTTGATTTGTCCAATAAATCGTGCGTTTATTTTATTTATTTGTTTTAAT
TAAATTGTTGTTGTTTTTTTAAAATAAAAATTTAAAACCTATGAAACTGACATGGACATCAAACACAACACGAACACGTGAACACCCTGAAATATAGTTC
GTACTACAGGTGTGGTATTGGTGTCGAACACTGACACAGACACGAAATACGATAAAAGACTGGCGTATCTGTACTTCATAGAGTACATTTTTTTAGGATT
CATCTAATCAAACTATGACTCTTGTTATTTGTTATTGCAGCACCACAATTTCTGGATGGGTGTTCATGATCTCTGTTGGATTCAATGCAGCTGCAAGGTA
GTAATTGGATTCCTGTTTTTTATTTATTTATTTCTTGTGAAATTTGCAAGTATTATTATATATTATATACTGTGAGAATATATAATGCAGCGTGAGGGTG
AGCAACGAACTAGGAGCAAGAAGTCCAAAATCAGCATCGTTTTCGGTGGTTGTGGTGACAGTGATTTCTTTCATAATATCAGTAATTGCAGCACTGGTGG
TTCTTGCATTAAGAGATGTCATTAGCTATGCCTTCACAGGGGGTGAAGAGGTTGCTGCTGCAGTTTCAGATCTTTGTCCACTCCTTGCCCTTTCTCTTGT
CCTCAATGGCATTCAACCCGTCTTATCTGGTAATAAATTAAACTCAATTATTTATTCTGTTTTCTCAACTTAATTTAAACAATTCATGAATAATAATCAT
TAGTAATTAATTAAATTGAGTCACATAACTCTGAACTCTTATATATGCATGAATCATAACATATCGTGTATGTATGAATGAAGGGGTGGCTGTTGGATGT
GGATGGCAAGCTTTCGTAGCTTATGTAAACGTGGGTTGTTATTATGGAGTTGGCATACCATTGGGCGCGGTTCTCGGCTTTTATTTTCAATTTGGTGCCA
AGGTACCTAATTTTCGCTTTTCCATTTCATAATTGATATAAAATATATAGTTTTAACTAGACTAAATTATACTAATCACTTTTGTAAAATTATACAATTT
TTATTTTATTTTTTAACATTTCCAATATCCTTCCCTCCTAGTTATAGGAAGAGTGCGGTTTAATATATTTTAAAAAAATTTAAAAGTTAGTATAATATTA
TTTAAAGGAATGTGGTGCATAATTTTCAAATAATTAAGAGGATTAATATAGAGCTATTTTTTTTTAATTTTACGAAACCTAAAAAATAATTAGTATAATT
TACTCTTTTAACTAATGTGCACTCCATCACATACTTTGTTTTATTTAATTGAGTCTTAGAATTCTCACTTTCAAAGTTTTTAGTTTACCAACCGGTAAGG
ATTATTTTTTATGTAACTTTTAATATAATTATTATAAAAACTAATAAATTTTGTCATATGATGTTTTGAAATGATTATTTTATAAAAGCACGTATTATTT
ATTCTTTAATTAATATTTCATATGTTTTTGTATTTAATATAAAAAGTGCATATATTTCAATGCTAAAAGAAGAGGATTTATTTTTTGCTATAATTAGTAT
ATATTTCACTAAACAAATGCAACTTTTATAAACGAGACGGGCTTTTTTGTGGCAGGGAATATGGCTGGGAATGCTAGGTGGCACCGTTATGCAAACAATT
ATTCTACTGTGGGTCACATTTAGAACGGATTGGACTAAGGAGGTAAAAAACCATAGCTGCAATAATTTATTTCCGTAAAACTATGCATGCAATGGGGAAG
TTAATTTTTCTGTCTAATGAGAATTTTCTTCATTGTAGGTAGAGGAAGCAGCCAAGAGGCTGACCAAATGGGAGGACAAGAAGGAACCACTACTAAATTG
AAGAACAACTTGTTTATGTAGGAGAGCAAGAGAGACATAATATTCTCTTTATTATTATTTTTTATTGAAACCAATTTATAAGGACTAATTCTAGAGATTC
AACAAGGTAGTTTAACACTTCGTTGTTCCATTATGAGGTAGGGAATTAAAAATTAAAACTGCCAAGGATTAATTCTAGTTGTCTAAGCAGCAATTTGAAG
TAGAAGGAAAAAGGGAAAAATGTCGTAAATTTGAATTCTTCTACCGATA

>Glyma.15G107100 | Chr15:8394915..8402251 reverse
CCCACACACACACATGATGGAATCCCAGAACCAGAACCTACTACGCCAACCCTTGATAAACTCAACGCATCATCATCATCACTCTGCTGATTCCCGTCTC
GAAGAGGTCCTCTCTGACCCCACCCTACCCTGGTCCAAGCGCATCCTCTCCGCCACATGGATCGAACTCAACCTCCTCTTCCCCCTCGCCGCTCCCGCCA
TCCTCGTTTACGTCTTCAACAACCTCATGTCCAACGTTACTCGCGCCTTCGCCGGCCACCTCGGAAACCTCGAACTCGCCGCTGCCAACCTCGGCAACAG
TGGCATTCAGCTCTTCGCCTATGGCCTAATGGTAAACATTCATTTCATTTCTCATATTAGAAGGTCTATCTCACCTCAGTTGGGTTCCATTTATAGGAAT
AAAAAAAATTAAATGAACATCTAAACTTAAAATTGATTTGCATATGATATATGTTCCACTAGCGAGTAGTCACACACAGAAGACAAAATCCTTGTTGACT
ATTTCAGACTCTATCTATCCAATCATGATGCTATAATTCTTCCAATGAAAACAATTATTATCCTGATCTTGATTATAAAAAAAATGCAAATTGTGTACTT
ATTAACTAAATTAGTTAATTTAAATAAAAAAATATTTTTTTAAAATTATCCTTCATTATAATTTAATATCAAACTTAAAAAAAATTATAATCTTCTTGAA
TGAAGAAAGTTTTGAAGATAATTTCATCAAATAATATTTAAGATTAAAAAAAATTTATCATTTATATTTAAGGTCAGAGAGAATACCCATTGAATATCAA
AACCTAACATAGATACATGCACTTCCGATTTGTTTGTACAGTTGGGTATGGGAAGTGCGGTGGAGACTTTATGTGGACAAGCGTACGGAGCGAACAAATA
TGAAATGCTAGGGATATACATGCAGAGAGCAATTATCGTGCTAACCATAACAGGGATTCCCTTAACGGTGGTGTACATTTTCTGCAAACCAATTCTTCTC
TTGCTGGGGGAGCCACCGGAGGTGGCATCAGTGGCCGCGATGTTCGTGTACGGTCTGATCCCGCAGATCTTCGCTTACGCAGTGAACTTCCCCATACAGA
AGTTTCTGCAGGCGCAGAGCGTGGTGGCGCCGAGCACATACATCTCCGCTGCCACGCTGGTGCTGCACGTGGCGCTGAGCTGGGTGGTGGTGTACAAGTT
AGGGTTTGGGATCATGGGTTCGTCGTTGATGCTGAGTTTGTCTTGGTGGATCATTGTTGGGGCACAGTTTTTGTATGTTGTTAGTGCTTCAAAGTTTAAG
GATACGTGGAGTGGGTTTAGTGTTGAGGCATTCTCTGGACTCTGGGATTTTGTTAAGCTATCGGCTGCGTCCGCGGTGATGCTGTGTTTGGAGACTTGGT
ATTTTCAGGTGCTCGTGCTCATTACTGGCTTGCTTGATAACCCTCAACTCTCTCTTGATTCTATCTCAGTTTGGTAAGTACTAATGCTTTCATTAATCAA
TTACTTTCTTAAACTTGCTTCCTCTCCACCTTATCAGAAACCAAGATATAAAATCATGATCACGGTACGTTTATTACCCAGAAGTTATAAAGTTAACTTG
CTGATAAAAAAAAATTAAGGAGATTCAAGGATTGAAGGAAGAAAAAGGAAAAATTTGTAGTTTCAATTTTTTTTTTCTAACATTCTAACAAAAAAAATAA
CAACTAAACTGATGTTGGCATATAAGAAATTATTTATTATAAAAAAATTAAAGACAATTGTGATTAATACAACTACAATTATAATTGTGTCACCTTGCAA
ACACCTCTAAAACCAGTTAGAACTATGGTCGTAGAATGTCTTTTAAAATCTTATAAGAAAGGATAAAAGTTATATACAATTTCATACATGTTTCATGTTT
GTGTTGTTTTCTAATCAACATTAGAGTTTGTCTGTAAACTACAAAATTGAACTTCAATTTTAATTAAAAAAAAAGAGTACAACTAAAACTTAGGAAATAT
ATCCAAATTTGTCTTAAAAAATCTAATATAAAATTATCATGATCTTAAACAATTTTCATATGACATGTAATAAAATTTTATTAACCTTTTTTATAAATTA
AAAAAACTCAAATACATATATATCACTTAGTGGGGTTAGTTATAATTATAGATAAATGATAATATTAAATCATGTAATAATTTCTCAGAAGACGTACGTT
AAAAATATTAAAGGAAAGAAACACGAGGCAGTGTTGGGCTGAAATAAAACACATGGGAAGGTGAGGTGAGACTGAGAGTGAGACCACTTGTACCATATGT
GTATTTTACTTTGTCGTTCAGTGGTGCAGAGAAAGTCTTGCACTGTGTTTTCCTTTGCCTAATTAGGCCTTTTTCTTTTTCATATGCACAAAAGTCAAAA
CTAATATAGGTACGAGCACTTTACTCTGTCAAGTTCGTTGTGTCGGGACACTGAAAGTCTGAAACTGCCTTTTTTATATAGCTTAAATTAATTGATTTTA
AATCGTGTAAAACGTTCATTTAGATAATACGGTAAAATTTAAACAATATACTTACTCAAATTAATTTAGACCATAATAAGACTGAACAAAAAAATTAGAA
GAGTAAATCAATCAATTTAAAAAAATATATCAAATTGAACTCGAAAAAAAAATGAAGAATCAAATTAAATGTAAAAAACAAATTAAAGGAGAAAAAAAAT
TAATTTAACCCTTTTTAAATATATTTCTATAACATTGTCTTCTATTTTTTATCACGTTAATTATATTATATAATACGTTTTTTTTTCTGGATGAAAAATT
GATATAATTTCTCTTATTAAAATGAAATTACACAATATCAATTCTCCAAACAAGCTCAAGCATGAAGAATATATAATGATCAGTTTTAATACCTCAACGT
ACATAACTAATAACTTAGAGAGGCTAGGCTCGTACCTTATATTAGTTGGATAATTCTAATCCCACGACATTTCTCTTCATATCGTAAATTATTTTTAGAA
TTGAAAAGGGAGATCAGAAAAGACAATGAGGATAAAATAGATGATATTTATGAAAAGAGACAAAAAAAATGGATGGTAAAAAAATTGTAAAATATTGTTG
TAGAAATAGTATTACCCTTTTCACAACACATCCTCACAACATGAACATCCTTACAATAAGGAGAGAAACAGGGAGAAAGAAAGAGAAAAATATTTTTAAA
AAAGTGATATAAAGAACTGAGTTATAAAACAATGTCATGCTTTTTTTATAGTAAAAATAACATCTGTTAATGATTTTTATAGGACCATGGTATAAGGAAT
CTGGTCTGAGATCGTCAAGTAAAAATATTTTTGATTAATGATCGTAAATATTTCTAAAATGTTTAAATTAAAATTGTAGGGTACTCTGGTTAAATTAACC
ATTTTATTTCGTCATCCAAACAAAGCACAATGGATCGAAACGAAATCTTTCAAAACTCAGGAATTAATAAAAGAAATAAAAGAATAGTTGGCACTTGGCC
GCTGTCATGTTGTTATAAGACAAGAAAGGTATTTATCCAAGTTGCCAGTCACATAGTAATGACATTACGTTACGTCTCTTCATAGGTAAGTCGTTGGGTC
ACTTTTAACAATGATAAATAAAAGGATATCATGGTATACTTATGTCCTGATAAAACTCTGAATCGAGTTAAAATTTCTTTCTTCGATTCAAAATCAAAGA
ATTAAAATAAAAAATACTAATTGAACCTTATCGATCCTCTGTAGTTTCAACTTAAATTAAGAAACCTGTATTTAAGTTTATATTACTGGGATTCATACAA
CTTTCAGGATAACTCTGAAGTGTCAAAATCTGAATTTTTAATTACTTGGATTCATACAATTTAGATTAAATAATTTTAACTTCTCAGACTTGAATTTTAA
TATTTGTTTTACACTCTTATCTCTCTATTACGGTGTAAACATTTCCATTATGAAAGGAATAGTTTTGACATCTAATCAATTGTTATAGCCCATAAAAAAG
ACACTGATAAATTGTTATAGCACATCCATTGATGTGCCCCCCTTCACCTAATGATGATATTTTGTTATGGTGTGTGTGATCGAGTTTTAGAAGTATTTCT
TAATATCTTTTATCTTTTAACTTTTTTTTATATCTTTTCTATCTTTTATTAAATTTAGATTTTTTTAGATTTAATTTAATAAATTTTCTCTATTAATAAA
AAATCATGGGTTAGGAATATAATTACTATATGTGCATTGAAGAATAGGGAGAAATATAACCTGTTAAAATAATTTCGTTTTATTTTATCCAATACCCTTT
TCTTTCTATCTAATTTGAAGGAATGGAATAAAATCATCTATCTTTCCCATTTTCATCTCATTTTGTTTTGTATTACACACCACCATTAACTTCTTTCAAC
CATAATTAATCTGAACGTAATATGACGTACAGCATGATTAATCACGTACATTTACTATGGTTATGACTTGTGAGTGCAGCATGGCAATAACAGGGTTGAC
GATGCATATTGGAATTGGATTTAATGCAGCTGCAAGGTTAGTTGGACTAATTATCTATATGCATGGTTTGAATTAATATGTTGCATGTGATAGTTGTAAA
CTGATAATTAGTGCAGCGTGAGGGTGAGTAACGAGTTGGGAGCTGAGCATCCAAAGTCTGCAGCATTCTCAGTGATTGTAGTGAATATGATATCTTTCAT
AATTGCGGTGATAGAAGCAGTGGTGGTCCTTGCACTGCGTCGGGTTGTAAGCTATGCATTCACAGATGGTGAAACCGTCGCTAATGCGGTCTCAGACCTC
TGTCCCTACTTGGCCGTCACTCTCATTCTTAATGGAATCCAACCGGTGTTATCTGGTGAGTAAGTCTATATATTTTTAAGATACATTGGGTGTGTTTGGA
TGGAGAAATTTAAAATTCTGATAAATTTTAAATTTTTAAAATTTTGTATTTGGATTAAAAAAATTAAAGTTAGAAATTTTCAATAATTTAAGGATTTCCG
GAACTTTCAAGGTAGAAGAGATGATGAAAGTTAATAAGGGAATACACAAAGGTTTTAAAATGTTCTTTTATAAAAAAAGAATTTTAATTTCTAACCTTTT
AGAAAGAAATTGAAATTTCACATTTTTAGTGGTTTAAAATTCTATTTTAAAATTTCAAAAATTTAAATTCTTCAAAAAAGACATCCAAACAATGAATTTT
AGATTATAGAAATTCAAATTCTCAGATAAATTACTTTCCTTAGTTAAAATGTTTGGATGGAGAAATTTAAAATTCTGATAAATTTAAAATTTTAAGAATT
TCAAATACTTCAATTGAAATTCTTTTAATTTCAAAATTTTGTGTTTAGATAAAAAAATTAAAATTATGAGGATGAAAAAAAATGAATGAAAAAAGAGAAA
ATATAATTGGTGTGCTAGTTATACGTGTCCCTTTATGTTCACACCCAATCGATATTTTAGGAGCTCGGAGGTGTTTCTTTTGAAAAAGACTGTAAGAAAA
AAAATTTAATTTCTCACCTTTTAGGAAAAAATTGAAATTTTAGATTTTTAGTTGTTTAAAATTCTGTTTTAAAATTCCAAAATTTTAAATTCTTCATAAA
AACATCCAAACAATGAATTCTAAATTACAGAAATTTAAATTTTCTGATAAATTACTTTACTCAATTAAAATTCTCTATTGAAATCTCCCCAAATGTTTTT
CTGTACATTTCTAATTTTTATTTTTAGGAGTGGCTGTTGGGTGCGGATGGCAAGCCATTGTGGCCTATGTAAACGTGGGCTGTTATTACGGGATTGGAAT
TCCGTTGGGCTGTGTTTTGGGCTTCACGTTCGGCTTAGGTGTGCAGGTAATATATTGTTATATTGGGCTTTATCAGTAATTTATTAGTTTATTGCAGGTT
ACATAATTTAAATATATCTTTCTACATTATTATTTATTAATTTACTATTTCCTACGAAAATAATTAATAAAAAATTTCATTAATTATCTTGCTAATATTT
AATGAGTTGAATTAAAATCTATTGAGAGGCCTACGTTCGATCCCTTTGAGCAACGTTTTAAGTGTGGATCAACAAAGCCTGTCTTTGACCTCTCAAATAA
TAATTGATGAAAACTTTTCTTTGATACATAATACAGGGAATATGGTCTGGCATGATCGGGGGAACAATGTTGCAGACCCTTATTTTATTATGGATTACAC
TTCGCACTGATTGGAATAAAGAGGTAATATTACTTTTTCACAAGAAAATATTATGAAGATAGTTTTTCAAACACATTATTTCTCTAATTAATTAAAATTT
ATTAAATAATAAAAAATCAAAAAGAGAAATCATTAAATAGCACGTGAAACTCAATTTTTTTTATCATTTTAATCAAAACTATATAATTATACATAAATAG
TCAAGAACAAAAAAATTGTTATCAGTTATGCTTAAAAAAGTTGTATACATTTTATCTTTTAATCTTGATAGTTTAAATAGAATTTAATGGTCCATTTTGT
GATCTAATTTTCCATAATAATTTGATAGACCATGTGTATCTTTTAAGTATCGACAATAAATATAGTCTATCAAGTTCATAAAAAATAAGTATATATTTAC
TTTTTAGAAAATTTGATAGATCATATTTTTTGAAAAATTCAATAACCGTATGGTCTATCAAGACTATAAGTATATCTACCTGTAAATTTTTAAGCAGGTG
TATCTTTTAACGATAAAATTTACTTCTTTAAATACAAGAAAATATCGACAATAAGATTACCTGACTAAAAATGTCTTATTTATGCAAAGTTTGGTGCGCT
TGATTGTTAGGATAATACCAAAATATTCAATGATTGAAAGTTAATACATAGAAATAGTTATTGAGTGAAAATTAAATTTTATAGTTGGATATTTTTAAAT
TAATTCAATAGTCTTTTACATCAAAATTTCCGTACTTCCTTAATGCAATAGTAAAGTATGATTAAAAGAAAATATAAAAATATTTTATACTAATAGTTAA
TCAAAATTAAGTCAACATCCATATATCGTACATTATGATGCATGCAGGTAAATACAGCAAAGAAACGATTAAATAAGTGGGGGTACAAGAAGGAACCTAA
GATTCAGAGTTGAAATGGTTCTTCTGCTGACCATTGGCCTAACAGATAGTTGTGGGTTCGGATTTCGAGTTCTCACTGGAAAATTAAAAGAAAAGAAAAG
TTGATGAATTATTGTTTTGTTTTTTCTTATGTATGCTTGGAAATTTTGCAAGTAATAATATGCTTCAAATAATTTAAGAGATCAAATAAGTTGTGTTCTT
AATGGCTTTTGAGCAAAGTTGATAAGATGACAAATTG

>Glyma.07G100000 | Chr07:9492032..9497076 forward
GAGAGAATAATGGCCTTATGGAAAGAGGAAGCCAAGAAGCAACTATGGCTGGCAGGGCCAATGGTATTTGTTAGTGTGTTTCAGTATAGTTTACAAGTGA
TATCTCTCGTGTTTGTAGGCCATTTGGATGAGTTGCTTCTGGCTAGTACTTCTTTGGCTACATCATTTGTCAATGCTACTGGTTTCAACGTGTTGGTAAG
TTCACACTATTTTCTAATTTTATGTTATAGTTTTCCTATTTCCAGAATGTGAGATGAGATCTATCCATGCTTGCCGGTGCATATATATGGAAGTGTTCTT
TCTCTGCCACCATTTTTGTTTATAGTATATACCAATACAAAACTTGCATCACTCTTGAAGTTGTATTTTTCTCAAACAGGCCAAATGTAAAAATAGTATG
ACTTTTTATAATTAAGTACAAATTTCATTATTAACGAAGAGGGGAAATGCACGGGACAAGTTTTTAATAAAAAACTTCGTACCCCATTGCCCGGAGGCTC
TTCGCTATGCGAAGGTATGGGGGAGGGATGTTGTACGCAGCCTTACCTTTGCATATGCAAAGAGACTGTTTCCGGATTCGAACCCATGACCGGGACAAGT
TTTTAATAGACACTAGTTTTTTTTTTAGGTGTCAAAGTGTTAAGTATCATTTGACATCAGTCTGTAAGACATATGTTCATTAATTAGACAGTACTCGTCA
AGTGTCTAGCTAGTTTTTTCTGTCTATTCAGATTTACACAAGTAGTTAAGATTGTGGTACGAATTTCCACTTTCTTTATAGGTGTGTGTTCGAATATCTC
TGAGACCATTTTTCCTGTTTGATGAGTGATTCTCACTCGAAAAATCTCTGATAGCTAAAGACTAGTTAAGTAATTCTAATTAAGGGAAATTACTTATAAA
TCAGGCCTGTATTATAATCTTTTAGATAGACCTCTGTTGACAATTCAAATATGTCATTGGTGCCATTGTATTAAGGATGTCTCTCTGTTTTACTACGTTT
ATTATTTAAAAAAAAAAAAAAAAACTAGATTCTTCTCGTATAATTGAGCCCACGCTTTACTTTGTTTGTGGTGTTCATGCCAGAACCCATACTTTTTTTT
GGGTTGAAAACCAAATTATTCCTTTACTTTTTCACAGGGAAACAAGCTAGAAACATTTTCAGCACTACACCTTTCATCAATTTTACTTTGTTAATTATAA
CTTTTTAATTAGTGTCAATCAAATATGAGTAAATTTTCATTAATTGATGCTTAGCTTCAATTGAAACATTTAATCAAGAAATTTTAAGAAAATAAAAAGT
AAGTAAAGAATTTAAATAGACAATCAAATACAGTCTTACTCAGTAAACATGTATTTTTTATATTGTATATTATCATTATTATTATGCACTTTCTTATTCT
TATGTATGATGCTAATCTTTCTGCCTAATGTTCATGGCTCACTTCACTTAGATGGGTATGTCAAGCGCACTAGACACATTTTGTGGTCAAGCATATGGAG
CAAAGCAGTTTCACATGCTTGGTGTACACACCCAAGGTGCCATGTTGGTTCTCACCCTTGTTACCATACCCCTCTCCATTATTTGGGTATTCTTAGGGCC
TATTCTAGTTGCTCTCCATCAAGACAAAGAAATTGCTGCACATGCTCAACTATATGCCAGATACTTGATCCCAAGCCTTTCGGCCAATGGTCTTCTTCGG
TGCATTACTAAGTTCCTACAAACCCAAAACATAGTCTTTCCTATGGTGCTAGCCACTGGACTTACTAGCTTCTTACACTTCTTTCTGTGTTGGGTTTTGG
TTCAAAAAGTTGGGCTTGGCATCAAAGGATCTGCCATTGCAATTTGCATTTCTAATTGGTTTAACACCATAATACTTGCTCTTTATATAAAGCTCTCCCC
TTCGTGCAAAACAACTTGGACTGGTTTCTCAAAGGAATCATTGCATAACATCCCTAAATTTCTCAAACTTGCTTTTCCTTCAACACTCATGGTGTGGTAT
GATATTCTTTTCTTAACCAAAATTTGTTTCCATATAATATAACAACTTGCATTGCAACCAACATAGCAAGATATGATCATTACTTTAGTCAATTGTTTAA
TTCATTACATTAAATTAATCCTAAAAAAAAATGAAAACTAGTCATTTAGTCCGTCATGTTTGTGTCATCAATTTGGTTCTTGAAGCACAAAAATCAATGA
CTTTAATTATCTTAGGGACAAGTTGATGTCACTCATAAATAAAGGACTGATGCAATATATACATGTCATATTTTGGGAAGATTAGATGTACATTATTTTA
ATTTATGTTGTTCTTCGCATATCAGATAGAGATAACTAGCAAAAACTTTTTTTTTTTCCTTAATGTGAATAACTACTCATGCTGAGAAGTTCTAATTGCT
ATACTCCTATTGTATCACTATGTTGCAGCTTAGAATCATGGACGTTTGAAATAATGGTGCTTCTGTCTGGTGCTCTTCCTGATGCGAAATTGCAAACTTC
GATGCTTTCGATATGGTTAGTTTTTCTGCTAATATCTTAGGGGCTATGCCATATGCTATTATATCCATCTTCCTCATTCAGTGTGTGTTTACGGTAAGGT
CAATAAGGCCTTTGCCGCCTTAGGCCATTAATAAGAAAAAGTTTTTGATATTATATATATTTGTTGAGGGACATAAAGTTACAATAACTAATTAAATATA
TTCACAGTAATAAAAATGTGAAGATATCAATGAGATTGCAGTCATTAAATTTTCAATGTTATCCAACCCATGTATGCCTATATATATGAGCGTGCATGAG
TATATGTGCTCGTGCGTTCGTGCGTGCATGATCATTTTTTGGTGTCTGGTCGACTTCGTGGCTCGAGACTTGGCTTGACTACTGTATGTTTTATATATAT
ATATATGCTATTATATTCATCTTTCTCATTTGAAAACCCCTTTCTTCCCTTGTTAATGATCAATTGTATTTTTTTCATCCCAGCCTTAACACATCTGGTA
TATTTTGGATGATACCATTTGGAATTAGTGCTGCTGGAAGGTTAGTCTTTCGTGCTGAACTGTATTGTTTATATTCTATATATTATTTTCCATAACAGAT
TAAAATAAAAATGTGCATGCATGCTATATTACCATATGTAATGTTGCAGTCATATATATTTTTTGCTTATTATTGACCCTAACAAATCTCATTACATGTT
TCCACTTAAACAGTACAAGGATCTCAAATGAATTAGGGGCTGGTAGTCCAAAAGCTGCATATTTAGCAGTTAAAGTCACCATGTTCTTGGCCTCTGCAGT
GGGGATTTTAGAATTTGCTTCCCTTATGATATTATGGAGAGTTTGGGGCCGTGTTTTTACCAATGTACATGAAGTGGTCAAATATGTGACTTCCATGATG
CCACTTGTTGCTAGCTCTACCTTTATAGATTCAATCCAAACAGCATTTCAAGGTATTGAGTAAAATCATTCAATGATATGAGGTTCAAAAATGTCCATTT
TCCCCGGTTGATTAAGTATTCAGGAATTGGATTAAATTGATCATTTCAAGGAAAACTAAATAGTATCATCCAGATCCATCACTTTTCTTATTTTACTATC
ATATTACTAGTTCAATCTCATCTAAATTAAGCGATTTAGATTAAATTTATTTTCAATATTTATTTTTTTATTGATATTGGAAAAAACTGCTGTTGTTATT
TCCTTTAACTGGATTTAGTTTGTTAGTTACTCTATTGTACAATTAACTGTTAATCAGTTAGCTTTCATGATAATATTTCATATTAATGTAACTCAACACC
TGTTCATATTTTTTTATTTGATTTTTTCAGTTTACATTGAATTGAAACGAATTATATATAAATACCCTACTAGTTGAACTAAACACTATCGAATGCAATT
TGAATGCAATCAAATACCTTAAAATCATTTATTGAGGAACCTAAGTGTTTATCTGTAAATGCTTGCGAACTTTGCTAATTCCTTCATTGTGAACAGGTGT
TGCCAGAGGATGTGGTTGGCAGAAACTTGGTGCATATGTCAATCTAGGGTCATATTATTTTTTGGGAGTTCCTTTTTCCGTTGTGTCAGCTTTTGTCTTC
CACATGAAAGGACAGGTAAAATATAACCATTTTATTATTAAAAAAAATCTTATAGTAAATATTAATATCCTTCAGAATTTGGTTTTTAGCTGAAACTAAT
AACATCGTGTAATTTGTATAATCAAGCTCACTTAGTAGCATAAGGCTATTGTTTGTTATTGGTTTGTATCTGGCATGATATATAGTGAACATGCATGTCC
AATTGAAGCTAGTGATTATCTTGATTCTTCATAATTACAGGGGCTATTTTTAGGGATCCTAATAGCACTTACTGTGCAAGTGGTGTGTTTCCTTCTGGTC
ACCTTACGCGCCAATTGGGAGAAAGAAGTAAGTTGTCCTATCTTCAACTCAGTTCTTATTATCAACAACTCTGCATGACTGCATACTAACACAGATAGAA
ATATGATAGGAGAACAAAAAAAATTGGATGCATTCTGGTTGATAGTGATTGATTTAAATACAGATTAGCACTAACAACCAATGGCTACAAAAGATAAAGA
GTGACTAGGAAATAGATAAGAAAAATGAGAACAATTTGATACTCTAGATAAGATAAAAAGTTTGTTACACAACTTTAGGTAAATATTATTATATCTTAAA
TTTTATCCTTTTCTAATTATTCCTTAACTAATAGCAGTGTCTCTTCGTTGTTCAGGCAAAAAAGGCAGCAAAAAGAGTAGGAGGCGATGGAGTCCAACTT
GGGGACCTTCCGTGTGACCAAAATGCCAATACTGCTTGATAGTTGAAGAATTTTAATTCTAGTTTTACGTGTAGAACTAGTGACTTATCTTGGAATAATA
ATGTTGGTTATATGAATCCGAAACTGAGATCAACATCAATGTACCTAGAGCACAACCATAGTTATTCGTTAGCATAACACTACAAAAACAGTAATCCCCG
CTGGAATTCAGTTATCTGATAAATTTTGATTTAGTTTCTTTTAAA

>Glyma.09G178800 | Chr09:40354319..40358609 reverse
AAAATGGCCTTATGGAAAGAGGAAGCAAAGAAGCAACTATGGCTGGCAGGGCCGATGGTATTTGTTAGTGTGTTTCAGTATAGTTTACAAGTCATATCTC
TCATGTTTGTAGGCCATTTGGATGAGTTGCTTCTGGCTAGTGCTTCTTTGGCAACATCATTTGTCAATGCTACTGGTTTCAACGTGTTGGTAAGTTGACA
CTATTTTCTAATTTTATGTTATAGTTTTCCCATTTCCAGAATGTGAGATGAGATCTATCCATGCTTGCATGTATATATATGCATGGAAGTGTTCTTTCTC
TGCCACCATTTTTGTTTATAGTATATACCAATACATGCAAAACTTGCATCGCTCTTGAAGTTGCATTTTTCTCAAACATGACAAATGTAAAACTAGTATA
ACTTTTTCTAATTAAGTACAAATTTGATTATTAACGAAGAGGGGAAATGCACAGGACAAGTTTTTAATACAGACACTCTAGTTGTTTTTCATTAGGTGTC
AAAGTGTCAAGTATCATTTGACATTATCAGTCTATATATCATCTTAATACTTGTAAGACATATATGCATTAATTAGACAGTACTCGTCAAGTGTTTAGCT
AGTTTTTTCTGTCTATCTGAATTTTCGAATCTTTCTAAGACCACTTTTCCCGTTTGGTGAGTGATCCTCACTCGAAAAATCTTTGATGCCTAAAAACTAA
TTTTAAGTAACCGTTATTGAGTGAAATTATTTATTGTTCAGGCCTATATAATAATCTTTTAGATTGACCTCTATTAACAACTCAAGCATGTCATTGGTGT
CGTAGTATTAAGGATGTCTCTCTGTTTTACTACTTTTTCTATAAAAAAAAAAAAAAAAACTAGATTCTTATATATTTGAGCCCTCGCTTTACTTTGTTTT
TGGTGTTCATGCCAGAACCCGTACTTTGTTTTGGGTTGAAAACCAAATTACTTTACTTTTTCACATAGGGAAAACAAGTTATTTTCAGCACTACACCTTT
CATCAATTTTAATCTGTTAATTATAAAATTTTAATTAGTGTCAATCAAATATGAATAAATTTTCATTAACTTATGATTAGCTTCAATTAAAACATTTAAA
TTTTAAGAAAATATAAAGTAAGTAAAGAATTTAAATTGACAATTTAAAATATCAAATATAGTCTTGTTCGGTAAACATGTATTTTTTTATATTGTATATT
ATCATTATTATGCACTTTTTTCTTCTTATGATGCTAATCTTTCTGACATGTTCATGGCTCATTACACTTAGATGGGCATGTCAAGCGCACTAGACACATT
TTGTGGTCAAGCATATGGAGCAAAACAGTTTCACATGCTTGGTGTACACACCCAAGGAGCCATGTTGGTTCTCACCCTTGTTACTATACCCCTCTCCATT
ATTTGGGTATTCTTAGGACCTATTCTAGTTGCTCTCCGTCAAGATAAAGAAATTGCAGCACATGCTCAACTATATGCCAGATACTTGATCCCAAGCCTTT
CTGCTAATGCTCTTCTTCGGTGCATTACTAAGTTCCTACAAACCCAAAACATAGTATTTACTATGGTGCTAGCCAGTGGACTTACTAGCTTACTACACTT
CTTTCTGTGTTGGGCTTTGGTTCAAAAAATTGAGCTTGGTATCAAAGGGTCTGCCATTGCAATTTGCATTTCAAATTGGTTTAATACCATAATACTTGCA
CTTTATATAAAGCTTTCCCCTTCATGCAAAACAACTTGGACTGGTTTCTCAAAGGAATCATTGCATAACATACCAAGATTTCTCAGACTTGCTTTTCCTT
CAACACTCATGGTGTGGTATGATATTCTTTTCTTAACCAAAATTTGTCTCCATAGAATGTACCAACTTGCATTGCAACCAACCTAGCTAGATATAAGCGT
CACTGTAGTCAATTGTTTTTCACTACATTAAATTAATCCTCAAAAAATGTGCAAAACAGTCCTTTAGTCCTTTAATGCCATGTTTGTGTCATCAATTTGG
TTATTGAAGCACAAAAATCAATGACTTTAATTATCATAGGGCCAAGTTGAAGTCACTCATAAATAAAGGACTAATTCAATATGTGGTATATTTTGGGAAG
ATAAGATGTACATTATTTTAATTAATGTTGTTCTTCGCATATCAAATAGAGATAACTAGAGAAAAAAAAACTTATTTATTTTTCCTTAATGTGAATAACT
ACTCATGCTGAGAAGTTGTAATTGCTATACTCCTATTGTATCACTATGTTGCAGTTTAGAATCATGGACGTTTGAAATAATGGTACTTCTGTCCGGTGCT
CTTCCTAATGCGAAATTGCAAACTTCAGTGCTTTCTATATGGTTAGTTTTTTCTATTAATAGCTTAGGAACTATACTACTGTATGTTATAGATATTATTA
TTATTATTATTTATATTTAGTCATAACCTCTAAAATTCACGGTAATAAAAATGTGAAAACATCAATCCAATTGTATCCATTAAATTTTTAATGTCACCCA
ACCCATGCATGTCTACATATATTTATATATGTAAGTGTGTACACACATACACGTGTACGTGTGTGATCAAGTTGATTACTACAAGAGTAAATTTAAAATA
AATAAAAAAACACTTCAAATTATGTTCACTTTTTTTCTCTCTTCTTTTCCTCTTATTTCTATTATTTTATTAAGACTTAAAGTGAATTTTCCAAATGAAT
TAACAAGTAATTGTGTCTCTATTTATATATAGGCTCTATTGCTTCCACGATTGTTTTATATTTTTTGTTTATAATTTATATTGAATAGATCAATTATTTT
ATCTATTAAGAGTGAAATATTATAATTACTTGATTATAAAATATTTTTAAATTATTTTTTAAGCTCAGATAAGTAGTTGATTAGTATGAAAATTAATAAC
TAATATTGCACTATATCTTTTTTAATAAATTTTACCATATGCCCTAATATTTCTAAACACGACCTGTTCTCATTTGAAAATCTCTTTCTTCCCTTGTTAA
TGACCAATTGTATTTTTTTTCATCCCAGCCTTAACACATCTGGTATATTTTGGATGATACCATTTGGAATTAGTGCTGCTGGAAGGTTAGCCTATCATGC
TAAACTGTTTATATTCTATAAAAATGAGCATGCTATATTACCATATGTAATGTTGCAGTCACATATTGTTTTGCTTATTATTGACCATAACAAGTCTCAT
TACATGTTTCCACTTAAACAGTACACGGATCTCAAATGAATTAGGAGCTGGTAGTCCAAAAGCTGCATATTTAGCTGTTAAAGTCACCATGTTCTTGGCC
TCAGCGGTGGGGATTTTAGAATTTGCTTCCCTTATGTTATTATGGAGAGTTTGGGGGCATGTTTTTACCAATGTACATGAAGTGGTCAAATATGTGACTT
CCATGATGCCACTTGTTGCAAGCTCAACCTTTATAGATTCAATCCAAACAGCATTTCAAGGTATTTAGTAAAATCATTCGATGACATGAGGTTCAAAAAT
TTCCATTTTCCCCGGTTGATTGAGTATTCAGGAATTCGATTAAATTGATCATTTCAAGGAAAACAAAATAATCTCATCCAATTTCATCAATTTTCTTATT
CATTATTTTTTATTTTTTATTTGATTTTTACAGTTTACATTGAATTGAAACGAATTATAAATACCCTACTTGTTGATCTAAACACTATACAATGCAATTG
GAATGCAATCTAAAATCTTTAAGGAACCTAAGTGTTTATCCATATATGTTTGTAAACTTTGCTAATTCCTTCATTGTGCACAGGTGTTGCTAGAGGATGT
GGTTGGCAGAAACTTGGTGCATATGTCAATCTAGGATCATATTATTTTTTGGGCGTTCCTTTTTCCGTTGTGTCAGCTTTTGTCTTCCACATGAAAGGAC
AGGTAAAATATAACCATTTTATCATTAAGAAAGATCTTATAGTAAATATCAATATCCTTCAGAATTTGGTTTTTAGCTGAAATTAATTAATAACATTTGT
AATTTATGTAACCAGGCTTACTTAGTAAGGCTATTGATATTTGTTTGTATCTGGCATGATATATAGTGAACAGGCAATGTTCAACTGAAGCTAGTGATTA
TCTTCATTCTTCATAATTACAGGGACTATTTTTAGGGATCTTAATAGCACTTATTGTGCAAGTGGTGTGTTTCCTTCTTGTCACCTTACGCGCCAATTGG
GAGAAAGAAGTAAGCATAGTTGTCTTACCTTCAACTTAGTTTTGTAATCAACCCTGAATCTGGATACTAACAAATTAAGATAGAAATATGA

>Glyma.07G100100 | Chr07:9502651..9508615 forward
ATGGGCATATGGGATAAGGAAATAGCAGATGAAGTAACGAAGCAATTATGGGTGGCAGGGCCCATGATATGTGTGTGTGTCTGTCAGTATAGTTTACAAA
TGATGTCTCTCATGTTTGTAGGCCATTTGGATGAGTTGCTTTTGGCTGGTGCTTCATTGGCTACTTCATTTGTCAATGTTACTGGTTTCAACGTGTTGGT
ATGTTCACAGTATTTTCTAATTTTATGTTATGGTTTACCAACTTTCAGAATGTGTGAGATCTGTGCATGCTTGCATTGTATTATATATGGACTGTGGATA
ACGGAGGTACCTGTGTTCATTCTCTGCCATCATTTTAATTTATACCAATTAAATAAAGGAAATAACAAAATATAATTGTTTGGCTTGAATTAAAACAACA
AAAAGAAGAATTTCAATAGACAATTTAAAATATCGAATATAGTCTTGCTCAGTAAACATGTATTTTAGAAAGAATCAACCCCTTGATACATGAATCATAT
ATATATATATATATATAATCAGTGGCAGAACCAGAAAAAATTGTTAGGGGACAAAATTTATAACATAATAAATAAAATTAATAAAAATCAAATTCTATTA
TTAATCGTTTAAAATTTAGAATAATGAAAAAAAAATTATCATATATCCAATGAAAATAAATATAATTACGTTTGATGGGAAAAAAAACACATGAAAATGA
AATAGATTTCTTTTCAACCATAAGGGTCGTGATGTTTGATAATTCTTGATTTGATAAAAATCTTCCATAATTTCTTTTGTAGTAAATGTTTTTGTAATAT
TCTTTTTAATATATATAGCTAAAGTATCCGGAAGAACTCATCTTGCATTTTGTTGTGAAACTTTGTTTTCACAAGTTTCTTGTCTAAAAAAGATTTTGTA
GAAGCGATTGAATTGGAAAAGTCAAAACAATACAAATTGCATATGTAATCAACTTTGAATGATTTAAAATTACTTTTATTACTTAAAGCTGAGTTAAGAA
GTTCAACTTTAGATTTTTTTCCAAATAAAAACATAAAAATAATATTGAAAAATAATCTAACAAATTAAGATAAATAACAATTATAAAAGTCCAAAGACAA
CCATTTAAAAAATAGAATAACACATAATATTTAAAAATAACAAGTTAAAAGCTAAATAATATAATATTATTCTGTTATATAATATTAATTAACCTAATAT
AATAGCTAAAGAATAACTGTAATATACTAAATGATAAATGTAATCTTGTTTTCTTTATTTTATTCAATATGTAATTTTGATTTTTCTATTTTAAAATTGA
GACATTTGATTCTGTTATTTTATAAAAGTACGTAATTTTCATCTCCAATTGTCTAAAATATGTGGGTGGAGGGAAAATAGTTTCCTACCGTATATAACTC
TTCTTATATCTCTTTTACTTGCATTATACTATATAGTCTTAAATGTAATTTTGGTCCCTCTATTTTACTCAATCCATAATTTTGATTCTTTTATTTTAAA
AATGAAACATTTTATCTTCCTATTTTAAATAATTTATAATTTCAATCTTCTTATTTTAAAATAGAAACATTTGATCCCTATAATTTAGAAAATCTACAAT
TTTAGTTTAATCTTTAATTTTATCTATTTTATTTTTTATTTTCTTACTTTGTAACTAATTAAATCATTTTTTAATGGTACCTTAAATGAATAATTAGGTT
TAGGGTTCAATCGGATAAAAAAACAAATTTAAGAATTTAATCAAAATTGTAAATTTTCTAAAATTAAAGGACTAAACGTTTTTATTTTAAAATGAGAGAA
CTAAAATCTTAAAATTTTTAATATAGAATGACCAAATATGTTAATTTTAAAATGGAGAAATCAAAATTACCGATTGAACAAACGAAAATCAAAATTATAT
TTTAAAATGTCTCAACTTTCTTGTTCTTATGATTCTTAATCTTTATGACTAATGTTCATGGCTCACTTCATTTAGATGGGCATGTCAAGCGCACTAGACA
CATTTTGTGGTCAAGGCAGTATCACATGGTTGGCGTGCACACACAAGGAGCCATGTTGGTTCTCATCCTTGTCACTATACCTGTCTCCATTATTTGGGTA
TTCTTAGGGCCTATTTTAGTTGCTCTTCATCAAGACAAAGAAATTGCAGCACAAGCTCAACAATATGCAAGATTACTGATTCCAAGCCTTTCAGCCAATG
GTCTTCTTCGGTGCATTGTCAAATTCCTACAAACCCAAAGCATAGTCTTTCCTATGGTGATAACCTCAGGACTAACTATAGCCTGTTACACATTCTTCTC
TGTTGGACTTTTGTTCTCAAACTTGGGCTTGGTTTCAAAGGATCTGTCATTGCAATTTGCATTTCAAATTGGTTTAATACCATACTACTTACACTTTATA
TTCGGTTCTCCCCTTCATGCAAAACAACTTGGACTGGTTTCTGAAAGAAATCATTGCATAATATCCCAGAGTTTCTCAAACTTGCTTTTCCTTCTGCACT
CATGGTGTGGTATGATTTTCGTTTCTCAACCAAAACTTCACTTTCATAGTATAATATAACAATTGGTAACCAATATAGGATAGATTAAATTTTTTAATCA
GTCATTTAAGGATACATGGTAGGTGGATATGTGCAGTAATCCCACAATCGGTTCTGGCATTTTATGGGTGTAGGACAAAATATAAATAAGTACCATTATA
TATATACACTTTAAAATATAAATATATGTTAAATTAATGAAATAAACACATAATTTTGTTAAAAATTAAATATTTTATTACATTAAACGAGTAACATAAT
TTTAACAAATTTTAAAAAATATATAGTTCATATTTTTTATTTAATTTAATTTAATTCTATATTATTATCTTTCATCAACAAAATCTCATTATCTCAAATT
AAATCTTACATGTATTTTTTTCTCTCTTTATTTAAAATGAATGAACAGTTAAGAAAATTACAATATGTATAAATATAAATAAAAGAGTACTTTGATCTCA
TTGAGAGTCTAATGATATAGAAGATGCACTTTTAAAGAAAAAGTGATAAATGGTTAAATTGAGAGATGGATGGATTTTAATTTATAGTATAAATAAGAAC
AAATTCTTTTATAATTTGATTTTTAATATGTATTTCTTGTTGAAAAATAAAATCTTTTAATTTATTTATTATCATATGCTAATTACAAGTTTATAAGACA
TAAAGAGTTAGATCTTTTTAGCCTAGGAGGGATCCTGAATAATCCTGTAGATCTCTTTTGGTCATCATAACATACTATTGTGTCATGCCCCGTGAGAATC
TCTCTCGTTCTGTCATTTTTCAGCACAATACAAAAAAAAAAAAAAAACAAAAAAACAAAAGATATATGCATTTAAGTCCACACTTTATCCATAAAATAAA
TTAATCAGCAAAAATTATGCACACCAATCAGTTAGTCCCTTAATATTTTTGCCATCAATTAGGTTCTTGAAGCACAAAAGTAATGAATTTAATTATCTTA
CGATCAATTTGATGTCACTAATAAATAAAGGACTTTAACGTCGGGTAATGTACAATTGTTTTGATTTAATTAATATTGCTCTTCACACTATCACATAGAG
ATTACTTGAAAAAACTTATTTCTTTTCCTAGTGAGTCGAAATGTGAATGACTGATGCTGAGAAATGCTAATTGCTGCTTCTATTCTATCATTATGTTCCA
GCTTAGAAGCATGGACATTTGAAATTATGGTGCTCTTATCTGGTGCTCTTCCTAACGCGAAATTGCAAACTTCAGTGCTTTCTATATGGTTTGTTTTTCA
GTTAAGACCTTCTATGCTACTATTTGTTCTGGATATTAAATAACATATGCTATTATATTTATCATCCCTTGATAATGACCAACTATTCTTTTTTCATCTC
CAGCCTTAATACAACTGGTATATTTTGGATGGTACCATTTGAAGTTAGTGCTGCTGGAAGGTTAGTCTTTCATGCTGAACTGTATTGTTTATATCCCATT
AATATCTAAACTGATTAAAAAAAAAAGAATAAATAATATATCCATTAACAGTGTAAAGAACTTCTACACTGTCATCTTTGTTGAGTTTTACAATAATTTA
TTTATAACTCATACCTACCATAATTGTTTAATGGTTGACAGTGTAAATTTTACACTAATAGTACATGGAAATTAAGCTCTTAAAAAAGTGCATGCTATTA
CCAATTTACCATAAGTAATGTTACAGTCACATGTCTTTTTTGCTTATTATTGACCATAACAAGTCTCATTACATGTTTACACTTGAACAGCACAAGGATC
TCAAATGAATTAGGGGCTGGTCGTGCAAAAGCTGCATATTTAGCCGTTAAAGTCACCATGTTCTTGGCCTCTGCAGTGGGAATTTTGGAATTTGCTGCCC
TTTTGTTGGTACGCAGAGTTTGGGGGCGTGCTTTTACCAATGTACACGAAGTGGTCACGTATGTGACTTCCATGATCCCAATTGTTGCAAGCTCTCCCTT
TATAGATTCAATTCAAACAGCATTTCAAGGTATTGAATAAAATCATTAGATTGGTTTTTAGTATATATATTTATTCATATTTTTATGAGTTGTTTTACAG
TTTACATTCAATTTATAAATACCACCTGTTGAACTGCACAGTAAGAATGCAATCTAATACTACCTTTTAAGTCATTTATTGAGAAACCTTGGTGTTAAAT
TGTATATCTGAACTTTGCTAATTCCTTGATTATAAACAGGAGTTGCCAGAGGATGTGGTTGGCAGAAACTTGGCGCATTCTTTAATCTAGGATCTTATTA
TTTTTTGGGCGTTCCTTTTGCAATTGTGACAGCTTTTGTCCTCCACATGAAAGGACAGGTAAAATCTAACCATTTTGTTATTATTAAAAAAGATCTCACA
ATCAATAATATCCTTAAAAAAAGTAGTTTTTAACTGAGACTAAAAGCATCGTATAATCCATATAATCTAGTGAGATAAGGCTTTTGTTGTGTGTTCCTAT
CCTGCACGGCATTGAACTTGTTCAACTAAGGGTATGTTTGAATTTTTGGTGGGAGGGTCCAAAATTATATTTGAGAGTAAAATCAATTTGAAGTCATGTT
TGATGATCCTCAAAAGTATGTCTAAGAATAAAATTTTGCTTGCAAACTCAGTTTTAGAGAAACAAGACTTAAGATACTTTTGCTAACTCGGTTTACAAAC
TTTATTCCAGACATGCGCTGCACTAAAGTTATAGTAATTGTCATAATTCTTCATAATTACAGGGGTTACTTTTAGGGATCGTATTAGCACTTATTGTTCA
AGTGGTGTGTTTTCTTGTGGTCACCTTACGCACAAATTGGGAGAAAGAAGTAAGTTGTCCTATCTTTAACTCAGTAGTTTATGTTATCTATCAACTATGG
ATACTAACAGCAGAGTCTTCATTGTTCAGGCAAATAAGGCAGCAACAAGAGTAGGAGGCAGTATAGTCCAAGTTGAGGCACTTCAGGGTAACCAAAATGC
TAATACTGCTTGAAAGTTGAAGAACCTTTGCTCTATATTACGTGTAGAGTGAGTTACCTTGGAGCCGAAACTCAGCACCGATGTTACTTATAGCACAACC
AAAATTATTCCTTAGCATGTACTACAAAACAGTAATTCGTGCTGGTATCCAGTTATATCATAAATAGGGATTTGATTTTTTAAAGTGAGGGACACACTTC
TTTTATCTATGGCTACATATATCTTCTATAGAAAAGAATATATTCCAGTCAAGAACATAAAGTGAGATACATTTTAATAAGGAGAAAAAAAAAGTAAATT
TATAATTATTATATGAATATCAATGAATAAAGATGACAGAATTCATATAAACACTTAATTTCGGCCTATCAAGAAAGACCTGCTATAGTCAAAATTTGTG
AAGGAAGACTTACTATGGCAGATGACAGAATAAACACGCAATTTTAGCATATAAAACTGTCATAG

>Glyma.09G178700 | Chr09:40344306..40347537 reverse
ATGAAGCGTCCACTGCCACCATCAGTGAGCAGCGACAATGTAGAGGATAGGAAGATGAAGAAGAAGGTGAAGGCATTAGATACGACGACAATGTGGAGCG
CGTGGAGGGCTAGCATGTTCGATGACAACCTCCTGTTCGAGGTGTTGAAGCACATAGACACTAGCGATGTCGGGATGCATAAACAAGCAGTGACACAAGG
TTCTCACGGTCGGTAGCTTATGTCACCTCCACGCGCTTTATCTCTTCCCTTTCTCCAACCCTCAACAAAAATCATCTTCTCATCAACACTGGCTAAGGTT
ATATTGCAGTAATTTTTGTTCATGAGATTTGAAATTGAGAAAATGACGAAAACATGATGCAGGCGTGGAGATCATTAAGGTAGATCTATTGTCAAACAAA
GTGAAGAAACATGTCATCATCTCAACTCCAGGGAAAAAAGACACCAGAAAAGAAGGTTGAATTGAAGAAACTTAAGGAGAAAAAAACAAAAGAAAAACCT
AAAAAGGTAATTAATTAGTAATTAATCTTCCTTTTTCCCTAATTGAACGAATTTATCTAGCCTTGACGTTCAATCTGTGAGCCTGACAGAAGTAAAGACT
TGGTGACAGTTAAGGGAACGATGAAGGAGATGGTGCCATACCTAAAACCAAATGACTTCATTTTTTGTTGGGTTTTGTTTGTTCAAGTAATCGAATCTGA
GGGGATGATGGATTTTTTGGTTGGGTTGTTGGTGTTCTGGAGACGCGTGAGGTGATTGATGGTGGCGCGTATGGTGTGGAGCTGGTAGAGGAGTTGGTCG
GGGATTGGTGGGGTGGTTTGGGGGGTTGGAGGGTGGAGGAAAGGTGATAGAAGGAGAGGATGTTGGTGGCAACCATGGCCATTAGGACAACCAGGTTCAG
GGTCATAATGGTAAAGGTGAAAAAAAAGATTATATTTTTGTTTTTTTTTTAATCTTATGTTTGATCTCGGTCATACAAAAATCACGTGTGACATGCTTGT
GTGGTTGATTAACGACTAGGTAAGTAGATAACGGATTCTGACTAACGGCAAGTATCTGAGACAAAAAATTTTAAATATTAGATATTCAATCTGAAAGAAA
AAATTATTAAGAATCAAACATAAAACACAAGTATTTATCGGAGATAAAAATATATTTAAGCCTAAATTAAATTAATAAGCAAAAAAAAAATATGTGCAAC
AATCAATTAGTCACTTAATATTTTTGTCATCAATCAAGTTCTTGAAGCACAAAAGTAATCACTTTAATCATCGTTCAATTTGACGTCACTAATAAATAAA
GGGTTAATTTAACATCTGGGTATACTTTCGGGATTAAAATGTAAATTTATTTTAATTTAATTAATATTGTTTTTCACACTGTCACATAGAGATTACTTGA
AAAACTTATTTATTTGTCTAGTGAGTCTTAATGTGAATGACTGATGCTGAGAAATGCTAATTGTTATACTCCTATTGTATCACTATGTTGCAGCTTAGAA
GCATCGACATTTGAAATTATTGTGCTTTTGACTGGTGCTCTTCCTAATTCCAAATTGCAAACTTCAGTGCTTTCTATATGGTTAGTTTCTCAATTAATAC
CTTAGAGGCCATGCTACTATTTGTTTTGGATATTATATAGCGTATGCTATTATATTTATTTTTCTCATTTGAAAACCCATTTTCTTCCCTTGTTAATGAC
CAACTGTACTTTTGGCATCCCCAGCCTTAACACCACCGGTGTATTTTGGATGGTACCATTTGGAGTTAGTGTTGCTGGAAGGTTAGTCTTTCTGGCTGAA
TTGCATTGTTTAATGTTTATATCCCATTAGTATCCAAAACTGATTAAAAATAAATAAATAATCTATGCAAAATGACTTCTACCGTCAGTTTAATCACAAA
CTAACATATGTATTAAGTTTATCATAATTTTTTTTTGTAAAGTCATACCTACCCATAATTATTTATTGATTGCCAGTGTAAAATCTACACCGACAAAGTG
AAAGTACATAAACTCTTAAAAAAAGTGTGCATGCTACTACCAATTTATCATAATAATGTTGCAGTCGCATGTCTTTTCTGGTTATTATTGACCATAACAA
GTCTCATTACATGTTTACACTTGAACAGCATAAGGATCTCAAATGAATTAGGGGATGGTAGTGCAAAAGCTGCATATTTAGCGGTTAAAGTCACCATGTT
TTTGGGCTCTGCAGTGGGGATTTTGGAATTTGCTGTCCTTATGTTGGTACGCAAAGTTTGGGGGCGTGCTTTTACCAACATACATGAAGTGGCCACATAT
GTGACTGCCATTATCCCAATTGTTGCAAGCTCTGCCTTTATAGATTCAATTCAAACAGCATTTCAAGGTATTAAACAAAATCATTAGATTGGTTCTTAAT
ATCTATATTTATTCATATTTTTATTTGTGTTTTTTATAGTTTACATTGAATTGAATCAATTTATAAATACCCTACTTGTTCAACTGCACAGTAAGAATGC
AATCTAATACTACCTATTAAGTCATTTATTGAGGAACCTTGATGTTAAATTGTCTATGTGAACTTTGCTAATTCCTTGATTATAAACAGGAGTTGCCAGA
GGATGTGATAGGCAGAAACTTGGTGCATTAATTAATCTAGGATCTTATTATCTTTTGGGCGTTCCTTTTGCAATTGTGACAGCTTGTGTCCTCCACACGA
AAGGACAGGTAAAATCTAGCCATTTTGTTATTATTAAAAAAGATCTCACAATTAACAATATCCTTAAAAAAGGTAGTTTTTAACAGAGACTAATAGCATC
GTATAACTCTCATAATCCAGTGAGATAAGGCAGTTGTGTGTTGCTATCCTGCATGGCATTGAACTTGTTCAACTGAGGGTATGTTTCTATTTTCAATGAG
AGGATCCAAAAGTTTGAGATCATATTTGATTATCCCTCAAAAGTATGCCTAAGAGTAACATTTTTCTTGCAAACCAAGTTTTAGAAGAACAAGATTTAAG
ATACTTTTGTTAATTCAGTTTGTAAACTTTAATTCAAACATGCACTGAAGTTATAGTAATTTTCTTAATTCTTCATAATTGCATGGGGTTACTTTTAGGG
ATCGTATTAGCACTTATTGTGCAAGTGGTGTGTTTTCTTGTGGTCACCTTACGCACCAAATGGGAGAAAGAAGTAAGTTGTCCTATCTTTAACTCAGTAG
CTATGTTATCATTTATCAACTATGGATACTAA

>Glyma.07G099900 | Chr07:9480608..9484876 forward
AAAAACTGCAACAAATAATTCGCCAATGGTAATGAAAGTAGTAACCAACTTCAAGTAGAATGAGAGATAGAGAGTAGCCAAAATGGGCATATGGAGAAAC
GAGATTTCAGAAGAAGCAAAGAAGCAGCTATGGCTATCAGGGCCAATGGTATTTGTGAGTGTGTTTCAATACAGTTTACAAATGATATCTCTCATGTTTG
TAGGCCATTTGGACGAGTTGCTTCTAGCTGGTGTTTCTCTGGCTACTTCATTTGTAAATGTCACTGGTTTCAGTGTGCTGGTACGTTCACATGATTATAT
TCTTTGTTGTTATATTTCCTAGCTATAAATTGATGTTCACGAGTATTCCATCATTTTCATATACCAAGGCACAAGATAATAATATAATAATTCATATAAC
AAACCTTCTAGCTGTGTTTGAATTAAAGAAGCTCAAAAGTGCATCATGCAAGTTCTTTAGAGATTTTCAGTGGCTCGGAATGAAGCCTAAAAAGCTTCTT
AGCCACACTAACTTGCAAGGGGGTGTTATGTTCAACTGCTACATAATTTAGCATTACTCTAGTAAAATTAACGTTAAGGAAATTTGCCGAAATTTGGAAA
GTGGAAACAGAATAAAAAATGTCAATTTGGGTCAACCTCCAGATGAAATATCAAATTTCAAATTTATATGTATCATTGCTCCATACAAAAATACTATTTG
TTGCTTATGTTGATTTGTTATTTAGCTAGGTATGTCAAGTGCACTTGACACATTTTGTGGTCAATCATATGGAGCACAGCAGTATCATATGGTTGGCATA
CACATGCAGAGAGCCATAGTGATCATCATGCTTGCTACTATACCAATGTCTTTTATTTGGGCATACTTGAGGCCTATTCTGGTTGTTCTGCATCAAGACA
AAACAATTGCAGCACAAGCTCAACTATATGCCACATACTTGATCCCAAGCCTTTCAGCCAACGCTCTTCTTCGGTGCATTACTAAGTTCCTACAAACCCA
GAACATTGTCCTTCCAATGATGCTAGCTTCTGGATTCACTACCTTAGCACATGCCCTTCTCTGTTGGCTTTTGGTTCTAAAATTTGGGCTTGGCATCAAA
GGAGCTGCCATTGCATTTTGCATTTCAAATTGGCTTAACACGGTGCTACTAGCACTTTACATCAGGTTCTCCTCTTCCTGCAAAAGCACTTGGACTGGTT
TCTCAAGGGAATCCTTGCAGAACATTCCTCAATTTCTCAGTCTTGCTTTTCCTTCAGCACTCATGGTCTGGTATGATCTTCGTTATTGAACCAAAATTAA
CCTCTATAAAAAGATAACAACTTGCACACACCCAATTCATGAGCAAATTTTAGTACTCCTAAAGTAACTACTCATTTGATAGTTAGGCCTTGATTGGATA
AACTTCTTCTAAACACACCATTAGTACTCTTATTTTGACATTTTTATTTCATTTCAATCCCCAAAAAAATAGAGAAAAAAAAATAGCTAACATGTCAGGT
GTCACGGTTTTAATGAAAGGAGTTTGTACTTTTTGAGTATTGAATTTTTTTTCTCTAAATTAGTTAATATTCTTCTCCAACATCTAATACATATGCCTTG
AGAAAAACTCATTTCTATTCCTTGTGATTCATAATGTGCATTGGAAAGTCTCATATGGTCTGCCATAGTTCCCAAGGTGCAGCATATAGACTTGTTAGAC
AATTTCACTTAATGCTAATTGGTTGAGATGAATTTTAACATGGCATTAGAGCCAATTAGTCCATCTCCATATATAGCCTATTCTAGTAGTCAGTAGGCAT
CTGTGGGTTGAACAACTTTACTTAATGTCAAATAATTTTAAAATGAAATCTAATAATGTGAATAACCAATGTGATAATATGATTTAATTTCTGATCTCTG
CTACTACTCTTCTACAGCCTAGAACAATGGACTTTTCAGATAATGGTACTCCTATCTGGGGCTCTTCCTAATCCAAAATTGCAAACATCAGTGCTTTCTA
TATGGTTAGTTACTTTTTTTTTAGTTAAGGTCTTAGAGCTATTCCATTTTATGATCAGGATTTTATATGGCATGTCACTACATAGATCTTTTATCTTCTG
GATGCACCATTTTCATGAATTCTTATCAATAACCAATTGGACTTTTCTCATCCCCAGCTTTAATACAACTGGTCTGTTTTGGATGATACCATTTGGGGTC
AGCGTTGCTGCAAGGTTAGTCTTTGATGCTATCTGTTCTACTTATAGTCTATTATTAAAAAAAGAAAAGTATGTTTGTAATTCATAGTTCATGAAGAGTA
GTCAGCTAGTCATGGTCAGAAAATGTGTTTTCTATTACAACCATGAAGAAAAAAAAATTTAATGACTGACTAACTCTTACCACCAAAAAATAAATATTGC
AATATCATGATTTATATATTATTGTTGGTTTACCAAGTCTAATTGCATTTTATATGTTTACTCTCAACAGTACAAGGATATCAAATGAACTAGGGGCTGG
CTGTCCAAAAGCTGCATATTTAGCAGTTAAAGTCACCTTGTTAATGTCCTTCGTGGTTGGGGCTTTAGGATTTATTCTTCTCATGGTCACAAGAAACATT
TGGGGGCATATTTTTACCAATATACCTGAAGTAATCCGTTATGTGGCATCCATGACACCAATTCTTGCAAGCTCTGTCTTTGTAGATTCAATTCAAACAG
CATTATCAGGTATTGAGTAAAGTGATACCAAAAGTTGCACTAAAATTATCTTTTCCTCATAGTTTCTAAGAACCTTATGCATCATAACAGGATGAAGAAA
ATATATTTAAGTACAATGAAACAAATTAAATATTAATAAAAAATTTCAAAAGCAAGTATATGAGGAACATGAATGGATTCCACTTCATCTAGTTCCTATA
AATGACAAGCACTACAGCAGTAGCCAAAAAAAAAGTAAAGGTACTAATATTAAAATTGTGACACTTTGACACGTATGATTATTTTTCTCAATTTAAAGTA
ATTTTTAATTTAAAATAAGAAAAGAATGTTAATTTGAGAGTATTAACTATCAGAGTACATACTGAAAAAATAAAAAATTGTTCGTGTTTTTGATGGGCTA
AGAAATGTTAGGAAGAAGAAAACGCTATAGCCAAGTATAATAACCCACGTGCAGATTAGTTACGTGTAGTTAGTTAATTCAGTTTCAGGTTGTTAGAATT
TGTTAATCCTGTTAGTTAGGTTGGTTATACGTGTAAAGAAAGCTGCAGTATATAAAGGCTGCATGCATAATGAATAAACCATGCAAAAGCTTATTTCCAG
AATTCCATTTCTCTTTTAGGAGGAGCCTGCCTCGAACTAAGGCACACTATCCCCTCTACTCATAACATTTTGTTTACTATATACATATGTGAACTTAACC
TATGCTAATCCTTTGGGGTGTGAAAATTAAAGGTATTGTAAGAGGATGTGGTTGGCAGAAGCTTGGTGCATTTGTCAATCTTGGATCTTATTATCTTGTG
GGGCTTCCCTTTGCAATTGTGTTGGCTTTTGTCCTCCATATCAAAGGAGAGGTAAGATTTAGTCAATCTTGTATCATTTTTTTTATGTACTTAATTAGTA
GTATAATGAATTTGTTTAATTGGAGTTAGTGCTTTTAGGGGCTCCTTTTAGGGATTGTGATTGCACTTACTATGCAAGTGGTAGGTTTCCTTGTCATCAC
GTTGCGCACCAATTGGGAGAAAGAAGTAAGTCTTCGTTCAAATCACTGGTGTTATCAATTTTCAATTTTGGAGGAAATGTAAGATTTGTCGGTGGTTGAG
AAAGAAAGGAATGAGGGAAAGGTCACCCATTCGATCTTCATTTGTCTGCTTATAAAAACTAATAAATAAATAATTAATATTTGTCTATAAAAAAAATTCT
AACTTTGGATCATACCATCAATAACGCAATATCTTTGTATATACTGTTCAGGCAAATAAGGCAGCAAAAAGAATAAGAAGCAACGGAGTGCCAACTGATG
CAAATGCACTGCCAAGTGACCAAAATTAAAATCTTCGACAGTTGAACAGTAATTATGCTTAGTGATTCTGATTATTTAGAAAAGCAGTTTTTAGTGAACA
TTTTTTTTGGTACAACTATTTTGTATATAAAAGGAAAGTGCCCTCGTAGATTCATAATTGGGCTGTTAAAATCAAACCAAAGACTAAATTAGCAGTGGGA
GAATGAATGATTCACAATTTAATAGAATTTCGTATGATATTAATAAAAAAAAATATTATGTTAATCTAA

>Glyma.08G050700 | Chr08:3963090..3965498 forward
AAACCGTTCATCCCTGTTCACGTAGATATATATTCATATTCATACGCGAAAGTGAAAGAGAGACCGAAACAAAACAAGGTGGCAGCAGAGTCTGTAGTCT
GTATGTTTGCATGTATCTGAAGAAAAAATGAGAATTGGGAGAGAAGAAGTGGTGGAAGAAGTGAAGAGGCTGCTATGGTTAGCAGTGCCTCTCTTCTCAG
TTGGAATTTTACAATACATTCTTCAAGCCATCTCTGTCATGTTTATTGGCCATCTTGGTACCCTCCCTCTTTCCGGCGCTTCCATGGCCTCTTCCTTCGC
ATCGGCCACCGGCTTCAACTTATTGTTGGGGCTAGCTACTGCACTGGACACATTTTGTGGTCAATCAAATGGAGCAGGACAATACCATATGCTTGGAATA
CACATGCAGAGATCCATGCTTGTTGTTTTAATGATGAGTGTGTTCCTTTCGATCATCTGGTACAACACAGAACCAATTCTCAAGGCTATGCACCAAGACA
AAGCCATATCCAAGGAAGCCGGCTCATACACCCAGTACATGATCCCGAGTTTATTTGCATACGGTCTTCTTCAGTGCATTCTCAAGTTCTTGCAAACCCA
AAAGATTGTGTTCCCAATGGTGCTAACTTCTGGAATTGCAGCTGTGCTTCATGTCCTTTTGTGTTGGCTTTTGGTTTTCAAGTCAGGGCTTGGAATCAAA
GGAGCAGCCTTGGCCAATTCCATATCGTATTGGATAAATGTAATCTTGATTTCTCTGTATGTGAGGTTCTCTTCTGCATGCAAACACTCTTGGACAGGTT
TTTCCAAGATGGCCCTGCACAACCTTCTTGATTTCCTTAAACTCGCTGCTCCTTCAGCTGTTATGCACTGGTATGGTAAATTCCCTTGCAAAATGATGCT
AATGTGATGCTCTTAACTAGATCTTGTAGATTTCTGATGCAGCATTTCTTCTCTTCTTTCTTTTTTTGTCATGAACATGGAACAGCTTGAAAGTGTGGAC
ATTTGAACTGATGGTCCTCATGTCTGGTCTTCTTCCTAACCCAAAATTAGAAACTTCAGTGCTTTCAATATGGTTTGTGTCTGAACTACATACAAACACA
CAAACTAAGAATATTCTTTGCTTTGCTTTTATTATATATTGATATATTATCACCTAATCTTTCCAGCCTTAATACATTTGGTCTAGCTTGGATGATCCCT
TTCGGATTCAGTGCTGCTGTAAGGTATTTTAAGTATGGGCAATTGCTCTCTTATTCAAATATTAATTCTTTTACCTTTTATTGGATAATTGAAACTGAAT
ATTCTATGTATGCTTTGTTTCACAGTGTACGGGTCTCCAATGAACTGGGGAGTGGAAATCCACAAGCCGCAAGTCTAGCAGTTCGGGTGGTGTTATCAAT
GGCCTTAATTGAAGGGGTCATTTTAGTTTCAGCCATGATTCTACTGCGGAATGTCTGGGGACATGTTTACAGTAATGACAAAGAAGTGATCAGATATGTT
TCATTCATGATGCCAGTTTTGGCCCTTTCTAGCTTTCTAGATGGGATCCAAGGTACACTTTCAGGTATTCCTCGCTAATCCCTCATGTTTATTTTATTTC
CTCCAGGGATAAATATCTGCCACAAAAGAGTATATTAGGAATATCCTAACAGTTTCAATTTTTGTGTACACAACAATCAGGCATTCTTGCAGGATGTGGG
TGGCAGAAGATTGGTGCCTATGTGAATCTTGGCTCATTTTATCTTGTGGGCGTTCCTTGTGCTGTAGTATTAGCGTTTATTGTACACATGAAAGCCAAGG
TTAAGATTTTGTCATCTATCATTTACTACCACTCGCTGAATCTGCCTTCCTTGTTTGTAAATGTTAATAGTTTTCTTCTGTCACACAGGGGCTTTGGATG
GGGATCATTTTTGCATTTATTGTGCAAGTGTCACTTTACATAATCATTACTTTTCGAACTAATTGGGAGGAACAAGTAAGCAATTTCAAAACTCACTCTC
AACGTTCTTCTCAATTCATTTTCCTGTTACTACATTTACACAACTCTTTCTTGTTCTTCATTAGGCAAGGAAAGCTCAAAGGAGAGTTGAACTCATAACC
ATCCCGCCAACTACTAGAGACAGCGTATTACCGCATCAAAAACAGGAACTAGTTCCCTAATCAATTTATGGTTATTGGCTTTATCATTCTGATCCCAAAA
CCTGCCACCGAAGGACTAATGTTGTTGTATACAGCCCCAAATTGGGAACAAGTAATGGAATAAAGATTGGAAGGGCCATGAATTTCAAAGACATACATTT
ATAGAACTTTTGTGCATTGTAACCAGTGGAAGAACAAAAATAACAACTCAAATGACTACCTATTTCAGAATGAATTTCATAAAAACATGGCTTTGGAAAT
GGAAGGATG

>Glyma.09G178900 | Chr09:40368198..40371411 reverse
AAAGCAGGCAGAATGAGAGAGCAAAAAAGCTTGGACAACATCAACAGAAAAATGGGATATTCTTTCTACCTTGCATATCTGTGCACAGAGACTTCAATTA
GTGGTCAAAAGATGGCAGGAAAAACAAAATTGACACAATCAACATTTGAATTTGTGTGGAATAAGGGGAATGAAACACAATGGAGAGGTTGTTCTTTGAG
TGAAAGTACAGCTCGGGGAATCCAATTTCTTCAAAAAGAGTTTGGCAAGGTAAGGCTCCACCATCTAAAAAAAAACTAACTATATAGCATCTTCAATTTA
TCACTTACTCTTCACCACACATAGATACTCACTTGTGCATCATTCTCTAGCACACAACACAAAATATATGAATGGAATCCCATGAATACTGAAAAATCTG
GAGTTAGTTTCCTTATAATGTTGGTTTAAATAAAATACAGTGAGGAGAAAAATAAAATCAAGAATGAAAAGAGTTTCAAGGCAGGAAGTGGTGGAAGAAA
TGAAGAAGCAAGCATGGTTAGCCGGGCCTCTCTTCACTGTTGGTGTGCTGCAGTACAGTTTGCAGGTGATATCTGTTATGTTTGTTGGCCATCTTGGAGA
ATTGCCTCTTTCCGGCGCTTCACTCGCCACTTCCTTTGCATCTGTCACCGGTTTCAATTTGTTGGTAAGGAGGTTGCAGCACTAAATTTGAGTATGTTTG
TTATCCAAAGAGTGTGAGTCTTAATGAAAGGATTTTATCCATTGTTTACACCTTTGATTTATTTAATTCTAAACTAAGAGTAGGCCATTTTTTTGGCTGA
TTACTTCTTATAACTTCTAACAGATGGGTATGGCTAGTGCATTGGATACCTTGTGTGGCCAGTCATTTGGGGCAGGGCAGCACCATATGCTAGGCATACA
AATGCAAAGAGCTACCTTTGTTCTCTCATTTGTAAGTGTCTTTCTAGCAATCATGTTGGTATTCACCAAACATATTCTAGTTGCAATGCACCAGCAAGTT
GCCATAGCTGAGGAAGCCGGGGTTTATGCCATTTACATGATTCCAAGCCTTTTTGCTTATGGTATCTTTCAATGCCTCCTCAAGTTTCTACAAACTCAAA
ACATTGTCTTCCCCATGGTGTTGAGCTCTGCAGTGGTGGCCTTACTTCACATACCTTTATGTTGGGTGTTAGTAATTAAGTCAGGAATTGGAAGTAAGGG
AGCTGCCATAGCAAATTCAGTATCATACTGGTTAAATGTGCTTCTGATAGGATTTTATGTGAAGTTTTCTTCCTCGTGTGCAAAAACATGGACCGGCTTC
TCAGTGAAGGCCTTGCAAAATATCCCAGAATTCCTTAAAATTTCTATTCCTTCAGCTTGTATGCTTTGGTATATTCTTGACCTTGAAGTAACGTTCTCAT
TTTCATCATTACTAAACACTGGTACACCCTTGCTGCATAAGTTATAATGAATGAGAGTTTTCATGTCCTAAAATTCTTATCATGTTTCTGATTCAGTTTA
ACCCTACATGGACAGCTTAAAGGCTTGGACATTTGAACTAATGGTTCTGCTATCTGGCCTTCTTCCAAATCCACAGTTAGAAACTTCAGTGTTATCTATA
TGGTTAGCTCTCGGCACATAACTAAATGCATATACCAATTGCTTTTTCACCCTCTTTCACAAAATAACTTGTCTTCTTTTGAGGCTGACCTTATTAATGT
GTAGTGTGACATTTTCTAATTTTCAGTCTTAACACATTCGTAATTGCATGGATGATTCCCTTTGGATTGAGCTGCGCTGTGAGGTGCCTTCATTCTCTGC
ATTTCCATTTTCCAAACACTTCCATTATGTATATGAATTTTCATTCAATTATAGCATTTGAGGAGCTTAAGGCATGATGTAGAAGCAACTATATATGGTT
TGTTTCCATAAACTTCTCCCTTTACAGCACAAGGGTCTCAAATGAACTAGGGGCTGGTCATCCACAAGCTGCAAGTCTTGCAGTTCGAGTTGCTCTCTTC
CTAGTCCTTGCTGATGGTATTATGATGGTATTAGTCATGATCCTGCTAAGAAAAATATGGGGCAATCTTTACAGCAGTGATACACATGTTATAAAATATG
TAGCAGCCGTGATGCCAATTCTTGCTACATGCAGCTTCCTTGATGGGATTCAAAGTGTTCTTTCAGGTATCCGTGTCAATTTCAGAATTTCCTACGCAAC
TCCTTTATATGTATTTGTGTGCAAATCCTCATTTCCGTTTCACGAAAAGGTTTCTTAATTAGCTTGGATGCACATAGTACATATGTTTCAGTGTTTCAGA
ACATCCAATTTTGATTATAATATTAATAATGTCTATTTATTGGTGGGCAATAAGGCATTGCAAGAGGTTCTGGCTGGCAGAAGATTGGTGCAATTGTGAA
TCTTGGATCATTTTATTTTGTGGGAGTTCCTTCTTCAGTGGTGTTGGCTTTTGTTTTACATATGAAAGGAAAGGTAAATTACACATAATTTACTTGTGGT
CTTTACAATTATTGCAACTTGCAATGGTCTTCAGATAAATTTGGCTAATGCATGTCTTGAAATAGGGCCTTTGGCTGGGGATTGTTTCTGCATTCATTGT
GCAAGTGATACTCTTTGGTGTCATTACAATTAGAACTAGCTGGGACAAAGAAGTAAGGCACCTATTCTATCTCTGTTTGCTGTACTAGCCATAAACTCTT
AACATCCATTCCATTCACTGTTTGCATCTTTTCCTTGTTTAGGCAAATAAGGCAGCAATGAGAGTGAAAGATACCAAAATCCCTCAGGAGTTGCCACAGA
GAGATCCATTTACCATCACAGAAATGAATTGATGTCATCTTTACAGAATTAAAAGACATGAGGCATTAATTTAAATCACCATACAAAATGTCATCTCTCT
CTTTCTCAACACACACGCACACATTACACATATGAGAGTTTCAAAAGAACGAATCTTAACATATAATTCAAGTAGATGGTTAAAATTAAAGCTAAGTAAA
ATAATTTTGACTTCTTGTAATATGGTTGTATGATCAAAACTTACTCTTCTCATCTCTCACACTCTTACTGGATATTAATGATATCAGACAGGGTCATTGG
TCAACTCAGTTAGAGTACTGGTCACTATAAGAACTAAGGAGTCAATAATTGAATTGCATTAATTGTATGATTAAAATATATATATTGCCAAATGTTGGCC
GGACAGTGTGCAAG

>Glyma.08G050500 | Chr08:3957774..3961167 forward
AATCTCTTCCACTTCCAGTGGCAGTACCCTCAGTAGAAGTGTGCCCTGTGAATCTGAATTCTGAACACCAACAATAAATTAAATAGTAATTAGACAGAGT
TGACACTCGAGTATTTTCAAGATAGAAAGCCAGTTTAAGGATTAAGCAAATATCTAAAAGCATGATAACAAGACGAAAGTGTACTATTCTATTCCTATCT
CATTTTCTTATAAATATCTCTTCAACTCTTTTTGTATTATATATACAGTATTGTTTCTTTCTGGTTTCTTTGGCTCGTGTCTTTCCTCTTAAACAACAAT
GGATAGAGAAGATCAAAAGGCATCTCTACTGTCTCAATTGATACATATCACAGTGGAAGATAGACTCAAAGTTGATAACATACAGCGCAACAAGGATAAG
CAGCAGGCAATAGAGAGAGCTGAACTGTATGAAGAAGTGAGGAAGCAGTTATGGCTAGCAGGGCCTTTAATATCTGTTTCTATGTTAAATTATTCCCAAC
AAATTATATCTGTTATGTTTGTGGGGCATCTCGGCCAATTGCCTCTCTCTGGTGCTTCAATGGCTACTTCTTTTGCCTCTGTCACTGGTTTTAGCTTATT
GGTTAGTTGTGACCTGCTTTCCTTGCCTTTGTGTGCCTACTGTGTACTGTTTCTAGCATGTGTAGCTGAACATTACTTATTTATTTTTGTGATCCTAAAT
TCCTTGTGTTCAATGTCTAATAGAGAAGTTACTAAGTTAGTATGCTAGTTAATTAGGTAAGACGAATCTGTCTAGTTATCATTTTCATCATGTAATATAG
CGGCTTATAACTGGTTCAAGTTAAATTTGTTCATCATTACCAATAACAAGTTAATCAGCAAACATGTATATTAATCTACAAATCAAAAATAAAGGAAAGA
ATAAAAGTGATTTCAATTCCAAGCTATAAGACAGGTTTGTACGTAATCAATCAATATTATTGAGTTACTGAGAGTATAGACACCTAATAACTTGATGTTT
AATTTCTTAAAAATTATTTTTTGTCAGTATTATGCCAAACAACTTCCAAATACTAATGTGGTAATATTTAATTCTCTAAGATGTTATTGGTTTTTTTATG
TTGTTCAAATAGGTGGGAATGGCTAGTGCTTTGGATACTTTGTGTGGGCAATCATATGGAGCAAAGCAGCACCATATGTTAGGCATACACATGCAGAGGG
CTATGCTTGTTCTTATGATTGTCAGCATAAACCTTGCATTTATTTGGGCAAATACAAGATCCATTCTGGTTGCCCTTGGTCAAGACCCTGAAATATCTGC
AGAAGCTGGACAATATGCTCAGCTAATGATTCCAAGCCTCTTTGCCTATGGCATTCTTCAGTGCCTTAACAGATTTTTGCAAACCCAAAATATTGTATTT
CCAATGGTGTTCAGCTCTGGAGTCACTACTTTACTGCATATTCTTATATGTTGGACTATGGTATTCAAATCTGGACTTGGAAACAAAGGAGCTGCCATAG
CAAATGCTATATCTTACTGGATAAATGTTTTAATCCTCATACTCTATGTCAAGTTTTCTCCCTCGTGCTCAAAAACTTGGACTGGATTTTCTAAAGAGGC
ATTGCATGGCATCCCTTCTTTTCTTAAACTTGCAATTCCTTCTGCTCTTATGGTTTGGTATAGTCTCTTCATACATTTTTACCAACAATTCTTACTTGTG
AAAGTTACATTTTGTTTCTTTTCTGAATCAGCCAATCCTATACAACTTCGGTTTAGGATAATGATTTTCTTTTTCCATTTTTCATTTATATTATCCGGAA
CAGCTTGGAAATGTGGTCGTTTGAAATGATGGTACTCCTCTCTGGCCTTCTTCCAAATCCAAAATTAGAAACATCAGTGCTTTCTATCTGGTTAGTTAGT
ATCATATTGAACATTAAATGTGTTTTGAGCTGCTACACATGCTTGACAATTTCTGTTTAACCAGTCTGAATACTTCGACAAGTGTTTGGATGATCCCCTT
TGGACTCAGTGGAGCTGTAAGGTGAGTAGCTTTTAAGAGTTTTCAAAAACATGATCAAATTGCTAAATTACTGAGTTGCCAAAGTGTTCTGTCCTCTTCC
TAGCTACTTTGTCTATTAGTACTTGTTTCTTGGTTCTGTACAGATGTTGTAGTAGTAATACGTTGCATGATTCTTTGCAGCACTCGTGTCTCAAACGAAC
TAGGAGCTGGTCATCCACGGGCTGCGCGTTTAGCAGTGTATTTTGTCTTTATAATGGCCATTATTGAGGGAACTTTTGTTGGAGCAGTGATGATAATAAT
ACGCAACATTTGGGGCTATGCATATAGTAATGAAGCGGAAGTAGTCCAATATGTAGCTATCATGTTGCCAATTCTTGCAACATCCATTTTCCTTGATGCA
CTCCAGTGTGTTCTTTCAGGTTTTTCTAGCTTATATTTCTTTTATTTATTGTCCTAATAGATACAGGTCTGAAAATAAAATCTTCCAATACAAAATGTTA
CATAACATCTTTTCACTCGAAGTCGAAAGGTTGCTTGCAATACCGAAATTCTATGATCAAATGTTACATGTCAAAATAAGGTTGCTTGTAACTCATTATT
TCGAAGAAAAAGAATGTACAATTTTGTATGTAGCTAAAGATTTAGACTAATCATCGACTCTAATGCAGGAACAGCTAGAGGATGTGGTTGGCAAAAAAAG
GGTGCTTTCATTAATCTAGGATCATACTATTTAGTTGGGATTCCATCAGCTATCTTGTTTGCTTTCGTCCTGCACATTGGTGGGAAGGTAAAACTAGTCC
ATTTTTCTGAAATCAGTCTTCTTAATTTTGTCATTTTCATATAAAAAAAATGTCATTCTACTTTGTATAGGGGCTTTGGCTGGGGATCATATGTGCTCTA
GTTGTTCAAGTGTCATGTCTTCTGATCATTACAATACGTACTGATTGGGAACAAGAGGTTAACTTTTGCCTTTTCGTTATACCTCCTTCATCTACTTTAA
TATTTTTATTTGATTTGATGAGCTCTGGGGGGTGATATGGTTGTATTTTTCTGGAAATTCCTTTCATTTTCAGGCAAAGAAGGTGAAAGACAGAGTCTAC
GATTCTATGAGAGCTGAGGTCATAGTCTCGTGAACATTATATATTTTAACTGGTGGCACCGTGAAGTGAACATTCCTCAACTGTGCACATCTATATGACT
ATATGTTTGTAGCTTTTAAACAGCGATATAGCATTAAGTCTCTTCAATCTGTAACATCTTTAACCACAAAAAAAAAAAAAGAAAGAAAACCTGTAACATC
TTTGTCTCCTCTGAGTCTGACGAAATTATCAACTTCTGATTCATGCTGGTCGATTCTCTAGTTCGTTCTCAAAATTGTCATATTGTTAATAGTC

>Glyma.09G179000 | Chr09:40373197..40377187 reverse
TTTTTCAGATGTTTTTATAATCTTTTTAACAACACCATGCAATATGCAACATTTTTCTCTGTCTGAGTCTTCCATGTGTTAAGAACTTAAAGAGCAACAA
AGAAGAGAAATGGAGAGAGGAGACCAAAACCAAAGTGCATCTCTTCAGTCTCCATTGATTAAGCACTCACACTCATCATCATCAGGGAGGGGTATTGAGA
GAAGGGAAGTTATAGAAGAAGTGAAGAAGCAGTTATGGCTTGCAGGGCCTTTGATATCAGTGACCCTTTTGAATTTTTGCCTCAGTATCATATCTGTCAT
GTTTGTTGGCCATCTTGGCGAGTTGTCTCTCTCTGGTGCTTCCATGGCCACTTCTTTTGCCTCTGTCACAGGTTTCAGTTTATTGGTATGTTAGTTCTCT
TTTCCTTCATAAGTTTAAACTGATGGATGGGTGGGAACCAAGGTTTTAAATATCAGTCGTGGTCGCGTTACGATTTCATCACGTTTGTTGATATTGTGAA
AAATTGCGGCCAACGCAATTCCAATTGTGGTCATGTTGTGGTGACAACCAGTTAAAAAACCTTAACGTTACGACCTGTTGGAATCCAAATCACGGTCGCG
GATCCTTTTTTAAAACCTTGGTGGGAACAAATGAATGATTTTATTGTACGTCTCTAAAACTTGGGAAGTCAACATCGTTGGTTCAACACACTTGCTTTTT
GCAAATCATCAACTGTTGCAACATGGTGTCAAGAACTCCACTAGCTGTCTATAAGTATAAGCTGGATACACAATTTACTTTTCCATTAACACTTACCTGA
GAAAGAAAAAAAATAAGGTAAAATGCATTGAGCTTCTCCCATAAGCTAAAATCAACTTATGCACCTTGATTTTGGGAGAAGTCAAATAAGAGAATTTTTA
TAAATAAAATAAAATTAAGTGTATAAGTTGATATTAGCTTTTAGGAGAAGCTAAATTCATTCTACCTTTTCTTTTTTTTCCTGGTGCTGAAATTTAAAGT
TTCATTACAAAAATCAGGGATACAAGAATCAAATTCTAGATCACTTGGTCCTAGCTAGAGGCTTTGATTATATGTCATGAATTAACAATTCCTAAAGTTT
AAGCTATTTGGCAAAGACAAATGAATGGTTAGATATGGCTGAGAAAGTCATCATTATTGGTTCACAACAATTGTGCAGGTTGTTACCAATTAGACTTCTG
TGTAGCAGAATGAATATTAATAGTTGGGAGTGTGAAGTGTCTATGGTTTACATTACAAACATCTTTTCAAGGATAATGGATGATGGTTTGCATTACAAAC
ATATCAATTTTTTTTCTTTTCTTTGTTTGGTTTTTGGTTGAAATAGGTAGGAATGGCAAGTTCCTTGGACACGTTTTGTGGCCAGTCATATGGAGCAAAG
CAGTATCACATGTTAGGCATACACTTGCAGAGAGCCATGTTCACTCTCATGATCGTCAGCATTCCGCTTGCAATTATTTGGGCAAACACAAGGTCCATTC
TAACTTTCCTTGGCCAGGATCCTGAAATAGCTGCAGAAGCCGGGAGTTATGCTAGGTTTATGCTTCCAAGCCTTTTTGCTTATGGCCTTCTACAGTGCCT
CAACAGATTTCTACAAACCCAAAATATTGTATTCCCAATGATGTGCAGCTCTGCAATTACCACATTACTACATGTACTTATATGTTGGATTCTAGTTTTC
AAGTCTGGACTTGGGAACAGAGGAGCTGCTGTAGCAAATTCTATATCTTATTGGTTGAATGTTACTATACTCTCACTCTATGTCATGTTTTCTCCTTCAT
GTGCAAAATCTTGGACAGGATTTTCCAAAGAGGCACTGCATAACATTCCCTCATTTGTGAGGCTTGCCATTCCTTCAGCTGTTATGGTTTGGTAATTACT
AATTACATTCTCTTAATACTTAAATACCAACTGTTACAATAGATCAATATTTATAGTTTTGTTATTTTTTTTAACATTGCACTTTTCAATTCTTTTTGAG
ATACTAATGCTGTTTCTAATTTTTTTAAACCATTATCATGGTGAGCAGCTTGGAAATGTGGTCATTTGAATTGATGGTTCTCCTTTCTGGTCTTCTTCCA
AATCCAAAATTGGAAACATCAGTGCTTTCCATCTGGTTGGTTTCATATTTGAGATGCTTTTTGCATTTGTTACAAATGATTGTCTTAATTTTTATTATTT
TCTCAATTATTACACAATACTTGACATTGTTTTTTCACATTCTTGGTTTTGTCAGCTTGAATACTACAGCAGCTGCTTGGATGATCCCCTTTGGACTAAG
TGGAGCAGGAAGGTTGGTATTTCTTTTGAAGTTTAAGTGGCCACATCACCTTTCATATGCTTCTTTAAAAGTTTCCCTTGCTTTACCAATACTGAATTGC
ATGGTTCTTGCAGCATTCGTGTCTCAAATGAACTTGGAGCTGGTCGTCCGTGGAATGCACGTTTAGCAGTGCGTGTTGTCTTAGTACTAGCAATTATTGA
GGGCATCATAGTGGGAACAGTGATGATACTTGTACGCAATATCTGGGGCTATGCATATAGCAATGAAGTAGAAGTGGTCGAATATGTAGCAACTATGTTC
CCAATTCTGGCAGCATCCAACTTTCTGGATGGACTCCAATGTGTTCTTTCAGGTTTTTCTTTTCCCCCATTACTTTTACTTGTCTGCACAGGAGCTATGA
CTCAAAAGCACTGGTATAGGCTGAGAGGCATATTGGAACTTCCAATACATCTCACATACAAAAATGCAAATAGTATACAGTCAATTGACATCTGGTTACT
ATGGGCCAATACCCGGAAAGGAACATGTGTGCATAGTACAAATGAGTGTATGTGAAGCTTTCAGTGAAGCAATTGTAAGATTTGATTTTCTTTAGTTTTA
AATTTCTCCAATGCATATTGCATAAATATGCGAGGCATACTGACAGTTAAGACTTAAACGGAATTAGTTCATTTTGTGGCCAATTTTCTTTAGAACCCAC
ATTTCCTTCTTGCACATCACATAAGCATGAAATAGATGATGTATAACTTTCTTTTTTCACTTTGTCAACAATAATTCAGGCACTGCTAGAGGATGTGGTT
GGCAGAAAATTGGTGCATTTGTCAATCTGGGGTCATACTATATAGTTGGGATTCCATCATCTATCGTGTTTGCTTTTGTATTGCATATTGGTGGGAAGGT
AAACAAAAGTTCATTAGTTTGTACAATAGCCTTGTTGCTAATGGTCAATTCTAAAAAAATTGCAATTGATCTGATTTTGTTTTACATGCTAATTACATCA
TGTAGGGACTCTGGTTGGGGATCATATGTGCACTCATTGTTCAAATGTGTTCTTTAATGATCATAACTATTCGTACTGATTGGGACCAAGAGGTGAATTT
TTTTTTCTTACTCTCAATCGTTTTGTTCCTTTTAATTATGTGGTGTTGAGTGCTGACATGCCTCTATTTTTAACTCCTTCAGGCAAAGAAAGCAACAGAT
AGAGTCTATAATTCAGTAACACTTGAGAGCTTAGTTTCATGAAGATGCAATATCGTTGCAGTTGCACATTCTGAGTTAAATTCTATTTGGATGAAAACAA
TACATGTTTTGGTATCCTTCTTTTGGCAGAAAAGTCTAGGGGCAATTATATATGCGCGAAAGATGATAGATGATGCAGTTGGCCATTGGAAGTGAATAAA
GTGGTTTTATTATTTTATTTTTATGTATGTGTTGGTAGGGGACGTCTTGATGTTTTTTGGAGGGAAAAGATTATGTTCTTGTGTAGAAGATACTAAATGT
GATTTTGATCTAGTTATTGAAAACCTTTCAATTTTTCTCATTGCCCCACTGGTGAGTCTATGGTTGGTTATATTAGGGGTAATTTTGTGACTGTAAACTA
AGTTTCGGATAGAATAGAGTAGTACAATTATTTTATCTTGTTGTAATAAGTCATTTGATGCACTAATGGAATATTTTCTTATTTCATATTC

>Glyma.13G275600 | Chr13:37706574..37710287 forward
ATGCTGAAAAGCAGGAGTTTAGATGGCACAGTAACACACTTCACACCTCTATTAGATGTATCTCATCATAAAGAAAACACAAGACAGTACCGATGGTGGA
ACAGCAAAATCCTGGATTTGGAGGAGGCCAAACATCAATTACTTTTTTCACTACCTATGTTCCTTACAAACTTGTTCTATTACTTAATCGTGTTGGTTTC
TGTAATTTTCGCTGGCCACCTTGGTGATCTTCAGCTTGCTGGTGCTACTCTTGCAAACTCTTGGTTTTCTGTCACTGGTTTGGCTGTCATGGTAAATTAT
ATTCAGAATTTCTTTAGGAATGTAGTGAAATGAATTATCATGTATGATAAGCAATATTGTTGAGTTTTATAATAAGATTGGTGCTAGCTACTTTAACAAT
GAAAAGCAGGAGTTTAGATGGCACAGTAACACACTTCACACCTCTATTAGATGTATCTCATCATAAAGAAAACACAAGACAGTACCGATGGTGGAACAGC
AAAATCCTGGATTTGGAGGAGGCCAAACATCAATTACTTTTTTCACTACCTATGTTCCTTACAAACTTGTTCTATTACTTAATCGTGTTGGTTTCTGTAA
TTTTCGCTGGCCACCTTGGTGATCTTCAGCTTGCTGGTGCTACTCTTGCAAACTCTTGGTTTTCTGTCACTGGTTTGGCTGTCATGGTAAATTATATTCA
GAATTTCTTTAGGAATGTAGTGAAATGAATTATCATGTATGATAAGCAATATTGTTGAGTTTTATAATAAGATTGGTGCTAGCTACTTTAACAATGATGC
AATATGCTAGGAAAATTTAACACCATAACATTTCCTCAAAACTAGTTGACTTGAATAAATGCAATTAACAAGATTGAGTATTCTTACAAGATCAAACACG
ATCATCTTCTTTCCCTTGTAATAGGTTGGTTTGAGTGGGGCATTGGAAACACTCTGTGGACAAGGATTTGGAGCAGAAGAATACCAAATGTTGGGAATTT
ATCTACAAGCCTCTTGCATCATCTCTCTTATTTTTTCCATCATTATATCAATTATTTGGTTCTATACAGAGCCAATCCTTGTATTGCTACATCAATCTCA
AGACATTGCAAGAACAACTTCCCTTTACACCAAGTTTCTTATCCCTGGCTTATTTGCACTTAGCTTCTTGCAAAATATCTTGAGGTTTCTTCAAACACAA
TCTGTTGTAAAGTCATTGGTGGTCTTCTCAGCTATTCCACTTTTGGTTCATATTTTCATTGCATAAGCCTTGATTTTCTGCACAGATCTGAGTTTTATAG
GTGCTCCTGTAGCAGTTTCTATTTCATTATGGATATCCATACCATTGTTGGTCATGTATATCATGTATGCAGAAAGGTTCAGGCAGACTTGGACAGGATT
TTCATTTGAGTCATTCAATTACATTTTCACAGACTTGAAACTAGCTTTGCTCTCAGCAGCAATGGTATGGTATGTCTCTAAAAACCTTACACTTAGCTAA
ACCCCTTTGATGATTGACCTTAGAAAGTTAAATGAAGAGCAGTTTTGTTTGAAAATCTGTTTTCTGTTTTTCATTCTCTCCAATAACTACAAAATTGCAC
ATTCTTTTCACTTTGTTTATTATTTTAATTTTTAATGATTATACAGGAAACATTAAGTGAAAAAAAGGCAGTAATTTTGCAATTATTTATAAAAATAAGA
AAAAAAAATAAAAAAAAAATGTATTTTCTCAAACTCAACATGTTAATAGATTTTAGATGAATAAACCACTAATTTTGTCTCTGATGTATTATCCTTTCTC
CTAAAGTAACTAAATTATTTAAGCGATTCCCCCATTTTTAAACATTCACTACTTTAGTCACCAAGTTCAAGTATTTCAGTAAAGTTCAGGAATCAGTCTG
AAGGTTCTACCAAATACTAATAATAGGAGTACTACTTAGCATGATTTTTTTAATAATTATATAATTACTTAAGTAGTTTTTATATTTAGGGACCACTTAG
GAGAGAAGGTAATTAATACTTAAACGATGAAATTAGTGATTTATTCGATTCGATTCTAGATTGAAGTCTATTTGCAATACATATTCAGAGAATATAGTTG
TAATTATGTATGAGACATTTACCTCATTTATCTCACAATCTGTTCACATTATGCAAGTTTCGAGTATTGGGCTTTTGAGATTATGGTTTTCTTAGCTGGA
TTATTGCCTGACCCAACAATATCGACTTCATTGATTGCAATATGGTATGAGTCTAATCCTTTTCAAGTTATACATGTATTCCTAAAACATAAAAATGTAA
TCATTCTTGTCAAATCTTCAATGATATTTGCAGCACAAACACAGAACTAATTGCCTATCTGATCACTTATGGTCTCAGTGCAGCTGCAAGGTTAGTAAGT
TAAGAATGTTCTCCTTTTGTTTGCATTGTTGTGTATCTTCTGAATCGGCACACATTATACAATTTTTGAATAGCTAAAAAAATTGAGTATGATATTAGTA
CAAGGGTCTCCAATGAATTGGGATCAGGCCACCTGGATCGAGCTAAGCATGCAATGGGTGTCAGTCTCAAGCTCTCTCTCCTCCTTGGTTTATGTTTTGT
TTTGGCACTTGCATTTGGTCACAATATATGGATTCAGATGTTCAGTGATAGTTCTAAAATTAAAGAGGAGTTGGCTTCACTGACACCCTTTCTTTCCATT
TCCATATTACTAGATTCTGTCCAAGGTGTCTTATCAGGTTTGAATAATTCCTCATTTTCCTTTTAACCTTTGTTATATTTGCTTCTTGATCTTTAACTTA
AAAAAATCAATTAGTGGAAATTAAAATACCTTTGATACTGCAGGGGTGATCAGAGGGTGTGGTTGGCAGCACTTGGCTGCCTATGTCAACCTAGCAACTT
TTTATCTTATTGGTTTGCCAATATCAGGCCTTCTTGGATTTAAGTTCAATTTGCAGGTTAAGGTAAAACATATTTGCTCAATATTCTGGTTCACTGTGTT
AATTCATGAGAAATTATTAGCTAGTCCCGGTCTACCAAATATCCATGATCTCGGTTGTTGCAATAGATCGAGATCCCTAACTAATGGGGACAATTTATTT
TAAAATGATAATAATAATAATAATAATAATAATTATTATAAAATGGAATGAAATGTGAGATGAATGGAACAAAAAGCGAGAAAAAAACTAACCAGATAAA
ATTTGACAATAGAATGAGAAATCAGAGATAGTAGATTCAAATATATAAAATTTAGGGGGACAAAATACAAAAACTTTGGATATATACTTATAATTTTCCC
ATCAAAATGAAAGGGTGTATTTATACACCCTTACTTGAATATAATTTAACTATTGTCGATTTGTTCGTCATTCGATCTCTATGTACCATGGTACATGAAA
AAATTAATAACACTAGTTTAAGTAGAAATTAGTTCTGACCATTATAATCCCATAACACCTAATAATTTCTTATTAGTTTACATGACAAGAAGAATATGTT
CTTTTGAATGATGTCTTGTGTATTCTTTTACATCCATTAGTTTAATCGTTTTATGTTCTTTTGGATTTGTTTTAATGTGCAGGGTTTATGGATTGGCCTG
ATTTGTGGGTTGGCTTGTCAAACTGGGACTCTCTCATTTTTGGCATGGCGTGCCAAATGGACTAAACTGAATGTCTCCTTGGACAGAGATGAAATCCAAC
CACCATCTGCTTAA

>Glyma.13G275400 | Chr13:37696340..37699959 forward
TCACGTTTAGCATATAGTTTTATATAATAATAAAGTTGTTTGGCTTATATTAATATCACCAAAAATTCAAAGTGCTTCCTTACACATATATATGGAAAAC
GTGGAGCAGATAAAAGAGGAGATAGAGAAGATGGTGGCAAGTTCTAGTTCAGAAGGAACAGGAGTACCCCTATTGAGAGCATCTGATAATAATGGAAGAG
ATCAAAACACAAGATCCCCCCAAGCTGAGGGATGGTGGAACAAAGTGTTGGACATGGAAGAGGCCAAGCACCAACTCTTGTTTTCACTGCCAATGATTCT
TACCAACTTGTTCTATCACTTGATCATTTTGGTTTCTGTCATGCTTGTTGGTCACCTTGGTGAGCTTCAGCTTGCTGGTGCTACTCTTGCAAATTCATGG
TTCAGTGTCACTGGCGTGGCAGTTATGGTAAATTCCTCTATCTTTTGCTCCCCTTTCTTATGATTTAAGATCAAAACATCATAAATGTTTATAATATAAT
AGCTTATTTTTCTATTATCAATTTAGGGTTTTTTTTAACAAAATTTGTGTTCTTATGGCCTCTTAATCTATGCCTAGTGGGATCGACAAAGCCTAGTGTA
TGACAAGCTTTTCAATATCAGTAGAGTAGTTGCTATGCTACCGGTCTCACAAATTGCAATAAATGTTTGAAAGCCTACATAACCAACTGCCATTAATGTT
TTGTTTCATAACTCATTCAGTAGTATAAAATTGTCTTCACTATGTCTAGAATACCTCTCCCTCCCATTGTAATTTGGTGCAAGATAACCAAATTGGTCCG
AAAATAATTATTATTTTAATTTTTCTTTATAACATTAACTATTTTTTTTTTACTTATATCCCTTATAATATTAATGGTGATCAACAAAATCTATAAATAA
ATTAATGATGATATAAAATTAATTTTATAAAATTATTACTTTTTTTTTTCTTGGTCTCGGTACAAAAAACCTACAACAATAATTATTTTGATATAATGGA
GTGTAATTTTTACAGTAATGATTATACGTTTTTAAAACAGGAAAAAAGATGAGAATCTAAAATTGTGAGTGGAAACAACGTTAGAACATGAAAACAATAT
ACTATGTAAGCCTGTAGGGGAGATTAACAGGGATACAATTTTCTGGCGAAATGTCAAATTATTGTTTCTAATCTTTATTTCTCTATACTTAAGTTCTGAC
ATATAAATGGTAGTCCAAATTTAACATCATAACATTTTCTCAATGCTCATTGAAATGAATGCAACAAGACTAAATATTGTTACTAGATTAATTCACAGCA
TGGAAATTGCATCATAACATCCTTATTTTTGTCCTAGGTTGGTTTGAGTGGGGCACTGGAAACACTCTGCGGACAAGGATTTGGTGCAAAGGAATACCAA
ATGCTGGGAATTTACCTACAAGCCTCTTGCATCATATCTCTTATTTTTTCAATCATTATATCCATTATTTGGTTCTATACAGAACCCATCCTAGTGTTGC
TTCATCAATCTCCCGACATTGCAAGAACAGCTGCACTTTATATGAAGTTTCTTATCCCGGGAGTGTTTGCTTATAGCTTCTTGCAAAACATCTTGAGGTT
TCTCCAGACACAATCTGTAGTAATTCCATTGGTTGTACTCTCTGCTCTTCCAATGCTGGTTCATATTGGTGTTGCATATGGCTTGGTTCAGTGGTCAGGT
CTGAGTTTTACAGGTGCACCAGTTGCTGCTTCTATTTCACTATGGATATCATTGCTATTATTGGCCTTGTATGTAATGTATGCTAAGAAGTTCAAGCAGA
CATGGAAAGGATTTTCAACGCATTCATTTCGTTACGTATTTACAAACATGAGACTAGCTCTGCCCTCTGCTGCAATGGTATGGTATGAATTACTAATAAC
CTTTCACTTAGCTAGCCCTTTTGATTGTTCATCTTGATTGGAAAAACAAGCCAACACAATAACAAACTCAATAATAGAGTAATAGACAGTGGAAATAAAT
TCTCCCAAATTTGCAAAAACCTAGGAGCACCAATAAATTATAGAGCGCAAAATAGAAATTAAGAAAAATCAGAGACACGATTTGTTTAAAGTGAAAAACA
CCTCAATACAAGGGTAAAAACCATGGTTCGTCCAGATTCATATATCATCTTATTACCTGATGTTTATGTTATGCAAGTTTGGAGTATTGGGCTTTTGAAG
TTTTGGTTTTCTTAGCTGGACTAATGCCCGACTCACAGATAACCACTTCGTTGATTGCAATATGGTAAGGTTCTAAATTCTAATAGCTTTATATGTTCGA
AAACAGATTCATATATTTTAATATTGTTAATTAAAATGAAAAAAAAAAGTTTCATATAATTTACTATTGTTAATCTTCAAAAAAATCTGCAGTATAAACA
CAGAATTCATTGCCTACATGATCACTTATGGTCTCAGTGCAGCTGCAAGGTTAGTTGATTGAACACATTCTCCTTTGCTTGCATTGTTGCATATCTTCTG
AATCAACATTCTAGTACATGTGTTGAAAAGTTGAAATTTAACTTTAACAGCACAAGGGTATCCAATGAATTGGGAGCAGGCAACCCGGAACGAGCTAAAC
ATGCGATGAGTGTCACACTGAAGCTCTCTCTCCTCCTTGGATTATGTTTTGTTTTGGCACTTGGATTTGGTCACAATATCTGGATTCAGTTTTTCAGCGA
CAGTTCTACAATTAAAAAGGAGTTTGCTTCAGTGACACCCTTGCTTGCCATTTCTATATTACTTGATGCCATCCAAGGTGTCTTGTCAGGTTTGAATTCC
TCATTTGTCATTTCTTTTAAATTTTGAAACTATAACTTTAACTTAAAAATTCTCAAGGAGGAAATTGTGTTTAATACTGCAGGGGTGTCCAGAGGATGTG
GCTGGCAGCACTTGGCTGCTTACATTAACCTTGCTACATTTTATCTTATTGGTTTGCCAATATCATGTTTCCTAGGATTTAAGACCAATTTGCAGTACAA
GGTAAATAAAATATTTATTGCATAGTCTGGTTATGTTTGTTCTGATATCAATCTTTTTGCATTCAGTTGTTTTATTAATATGATTGTGTTGAATTTGCTT
TATGATATAGGGTTTATGGATTGGTCTGATTTGTGGGTTACTTTGTCAAAGTGGGACTCTCTTCCTTTTCATAAGGCGTGCCAAATGGACTAAATTGGAT
CTCTCTAGGGACAATGATAAAGAACGGCCTCTTGTTGTTTAACTGAATGGAATTGGACTTATGGTAAAAAAAAAAGAAAAAGGTTTGCATTCTATTATGC
AGAAGTTTATCAGATAGGGTGAACACTGAACATGATTTTGGTGAAGCGTTTCTTGCTTATATTCATTTTGAGAAAGTGCTCACACTTTTTTTTATATTTT
ATTTGGGAGTGTTGGTGAGTTGCAGAGAGTAAATCTTGGCATATAATGTAACAGTCTCACGGGAACACATCAAGAATAGATGGGGAGTAATGTATCCCAC
CGTGAAAAGAATACTATAAAAAAACTAGGAAACTACGTGTAATTGACAATGGAGAACCATATGATTGATGATAGATTATTTTCTCGCGTGAGAAGTGGGA
CTCTCGAATTTAAATTTAAA

>Glyma.12G225600 | Chr12:38518185..38519665 reverse
ATGCTGGGAATTTACCTACAAGGCTCTTGCATCATATCTCTTATTTTTTCAATCATTATATCCATTATTTGGTTCTATACAGAGCCCATCCTAGTGTTAC
TTCATCAATCTCCCGACATTGCAAGAACAGCTGCACTTTATATGAAGTTTCTTATCCCGGGAGTGTTTGCATATAGCTTCTTGCAAAATATCTCGAGGTT
TCTCCAGACACAATCTGTGGTAATGCCATTGGTTGCACTCTCAGCTCTTCCACTGTTGATCCATATTGGTATTGCATATGGCTTGGTTCAGTGGCCAGGT
CTGAGTTTTGCAGGTGCACCACTTGCAGCTTCTATTTCACAATGGATATCAATGCTATTATTGGCCTTGTATGTCATGTATGCTAAGAAGTTCAAGCAGG
CATGGCAAGGATTTTCAATGCATTCATTCCATTACGTATTTACAAACATGAAACTGGCCCTGCCCTCTGCTGCAATGGTATGGTATGAATCACTAGCAAC
CTTTCACTTTGCTTACCCTTTTGACTATTGATCTTAGAAAGCTAAATGAGGAGAAATAGCCTAATTTGCAAACATACATGATACATTAATTCAAATTAAG
AGAATATTGTTGTAATTTAATGTAAGATCCTTATATCAACTTACTGTATGTTGTTTTTGTAGCTAAAAAATCTTACTATATGTTGTTTATGTTATGCAAG
TTTGGAGTATTCGGCTTTTGGTTTTGGTTTTCTTGGCTGGACTGTTGCCTGACTCTCAGATAACAACTTCGTTGATTGCAATATGGTATGGTTCTAATAG
CTTGGTATTCAGGTATTCTGTTCCTGAAAAATAAAATAGTTAGTATGATTTATTATTGTTGTAATCTTAAAAAAATCTGCAGTTTAAACACTCAATTCAT
AGCGCACATGGTCCCTGTTGGTCTCGGTGCGGCTGAAAGGTTAGTTGGTCGAACACATTCTCCTTTGCTTGCATTGTTGCATTTCTTCTGAATCAACATT
CTAGTACATGTATTGAAAAGTTCAAATTAAAGAGGAGTTTGCTTCAGTGACACCCTTGCTTGCCATTTCCATAGTACTAGATGCTGTCGAAGGTGTCATA
CAAGGTTTGAATTCCTCATATCTTTTAACCCGCTTTATCTTTTCAACTTGAACATCATTCACTTTAAATTCTTGGTGTGAAAATTGTGTTTAATACTACA
GGGGTGGCCAGAGGATGTGGTTGGCAGCACTCAACTGTTTATATTAAATATTTTGCACATTCTAAAAATCTGATATCTTGATTTTGAGTCTTTTTTTCAT
TTGTAATTTTTCATTATTATGTTTGTCTTGAATTTGCTTTATGATATAGGGTTTATGGATTGGTCTAATTTGTGGGCAGCTCTGTCAAGTTGGGACACTC
TTCCTTTTCTTAAGGCGTGCCAAATGGACTAAATTGAATCTCTCTGGGGTCAAAGATAAAGAGCATCCTCTCGTTGTTTAA

>Glyma.13G275500 | Chr13:37700577..37704898 forward
TGGCCGAGGCCCGAGATCAGACAACCTACTCGTGAAGCATATTCGAATAATGAATATTGTAACTCAAGACTTGGGGATAAATCTACAAAATAAGACAAAG
TAACCCAATGGAAACTATGAAAAAGAAATTCGTTACCTGTGCAACAACCCAAAATCAAATTATTTAGAATCGCCAAGGGAATTATTTGGCATGATAGTAG
ATCATTATTTCGATGCTGCATGATGATTGATCCATAATAGATACAACCACGATGGCGACCAAAGCTGTTCCTCGTATCATATTCTGGGTTTATGGTTAAT
ACAAAAAGCACCTCTGAAACTTTCAGCAGCATTTATCTGTCATAAAGTAACTTTTGGCTCATAGTTTTGTATAATAAAGTCGTTTGGCAGTATATATATA
GCGATCAGCAATCATTCAAGTGCTTCCTCATCAGATATATGCAGATAACGTAGAGCAGATGAAAGAGGAGATAGAGAAGATGGTGGCAAGTAACAGCTCG
GATGATAGCATAGGAACACCTCTAGTAATAAGGGGATCTGATAATAATGGAAGAGATCAAAACACAAGACTCCACCAAGTTGAGGGATGGTGGAACAAAG
TGTTGGACATGGAAGAGGCTAAGTGCCAACTCTTGTTTTCACTGCCAATGATTCTCACCAACACGTTCTATTACTTGATAACTTCGATTTCTGTCATGCT
TGTTGGTCACCTTGGCGAGCTTCAACTTGCTGGCTCTACTCTGGCAAACTCGTGGTTCAATGTCACTGGCTCTGCTGTCATGGTAATTCCTCTCCCACTT
CCTCTTCCTCTTCGATCGCCTTTCTTATAAATCAATATCTATATATTTTATATGATTTCTTTTTATCCTATACAATAAGTCTATAGAATAAAAACCAAAA
ATTAAAAAGAAGTATAATTAAATGGTTGCTTTTTAACTAAAACTTTCTAGTTTTAGCCTCTTAATCTATGCACTCCGTTCATAGGATATCGGATAGAAAA
ATCATGGTACATATGACAAACATTTCAAAATCAATAGTTGCTCTGGTATCAGTTTTAAAAAAAAGCTGTCAATGTTGAAAGCCTATATAACCACTTGCCC
ATTATTATTAATGTTTAACATTTCATTTTGCAGTATTTAGATTTTGTACAGTGTAATTATACTGAAGTGTTACTTATAGTTCTTAATCATTATTTCTTAA
TGCTGAAATATCAATTGTTGTTTCTGTTATGGCTTTTATATACTGTAATGCCTCACATACTGAAATGTCAATTATTGTTTCTAATCTTTATTTCTATACT
TGAATTATGGCTCATCAATGTTGGTGCAAATTTAAAATCATAACATTGTCTCAAAGCTCGTTAAATTGAATGCAAGAAGACTAAATGTTGTTACTAGATT
AAACTCCTGGTTCATACCCTTGTATTCTTATATTAGGTTGGTTTGAGTGGGGCACTGGAAACACTCTGCGGGCAAGGATTTGGTGCAAAGGAATACCAAA
TGCTGGGAATTTACCTACAAGCCTCATGCATCATATCTCTAATTTTTTCCATCATTATATCCATTATTTGGTTCTATACAGAACCTATCCTAGTGTTGCT
TCATCAATCTCATGACATTGCAAGAACAACTGCTCTTTATATGAAGTTTCTTATCCCAGGATTATTTGCATATAGCTTCTTGCAAAATATCTTGAGGTTT
CTCCAGACACAATCTGTGGTAATGCCATTGGTTGCACTCTCAGCTCTTCCATTGTTGATTCATATTGGTATTGCATATGGCTTAGTTCAGTGGCCAGGTC
TGAGTTTCACAGGTGCACCAGTTGCAACTTCTATTTCACAATGGATATCAATGCTATTATTGGCCTTGTATGTCATGTATGCTAAGAAGTTCAAGCAGAC
ATGGCAGGGATTTTCAATGCATTCATTCCATTATGTATTTACAAACATGAAACTGGCCCTGCCCTCTGCTGCAATGGTATGGTATGAATAGTAACTCACT
AGCAACCTTTCACTTTGCTTACCCTTTTGACTATTGATCTTACAAAGCTAAATGAGGAGAAGTAGCCTAATTTGTAAACATACATGATACAGCAATTCAA
ATTAAGAGATTATTGTTGTAATTTAATGTAAGATCCTTATATCATCTTACTATATGTTGTTTTTTGTAGCAAAAAAATCTTACTATATGTTGTTTATGTT
ATGCAAGTTTGGAGTATTGGGCTTTTGAAGTTTTGGTTTTATTGGCTGGTCTGTTGCCTGACTCACAGATAACAACTTCATTGATTGCAATATGGTATGG
TTCTAATAGCTTAGTATTCTGTTCCTGAAAAAAGAAAATAATTAGTATAATTTATTATTGTTATAATCTTCAAAATATCTGCAGTTTAAACACACAATTC
ATTGCGTACATGGTCCCTGTTGGTCTTGGTGCAGCTGGAAGGTTAGTTGGTTGAACACATTCTCCTTTGCATGCATTGTTGCATTTCTTCTGAATCAACA
TTCTAGTACATGTATTGAAAAGTTGAAATTTAACTTTAATAGCACAAGGGTATCCAATGAATTGGGAGCAGGCAACCCAGAACAAGCTAAACATGCAATG
AATGTCACCGTGAAGCTCTCTTTCCTCTTTAGTTTCTGTTTTGCTTTGGCACTTGGATTTGGCCATAATATCTGGATTCAGCTTTTCAGTGGTAGTGCTA
AAATTAAAGAGGAGTTTGCTTCAATGATACCCTTGCTTGCCATTTCCATAGTACTAGATGCTGTCCAAGGTGTCATGCAAGGTTTGAATTCCTGATTTCT
TTTAACCTGTCCTGTTCTATTTTTGCAACTTGAACATTAACTTAAAATTTTCTTGGGGTGAAAATTGTGTTTAATACTACAGGGGTGGCCAGAGGATGTG
GTTGGCAGCACTCAACTGTTTATATTAACCTTGCAACTTTTTATCTTGTTGGTTTACCAATATCATGTCTCCTAGGATTTAAGACCAATTTGCATTATAA
GGTAAATAAAATATTTATAGCACATTCTGGTTATTTTTGTTCTTATATCATGAAGAATCATCTTGATTTTGAAACTTTATTCATTTGTAATGTTTTATTA
TTATGTTAGTCTTGAATCTGCTTTATGTGATATAGGGTTTATGGATTGGTCTGATTTGTGGGCTGCTATGCCAAGTTGTGACTCTCTTCCTTTTCTTAAG
GCTTGCCAAATGGACTAAATTGGATCTCTCTGGGGACAAAGATAAAGACCACCCTCTTGTTGTTTAACTGAATGTCTGCCAATCTGGCACTGAAGTTATG
AACTAAGATGATCACAAATTGGGGTGAACATGATTTTGGTAAAACGTCTCTACTTTCTTGCTTATAGTTTTTATTCATTTTGAGAAAGTGCAAACACTTA
ACCTTTTTGGGGGGAGTATTTGTGAGGCGCAGGCAGTAAATCTTGACATATAATGTAACAAACTCTCCAATTAGGTGGTTTTTATTCCACAATCTCACAA
GAACTCATTTAGATTTTAGAGGAGATAGGCAGAAATGTATCTTAACATAAAAGATTATTATCAACTTAGCACTTAAGTGGACTTAACCTTGGAAAATCAT
TTGATTGATAAGAGATTAATTTCTCGCATAAGTGGGACCTTTATAAAAAATATTCTTTTCCCCACATGCTCCACTTGAAATTACATTTAAATGAAGAAAT
CTCTTTCTTGAATATTTGGCTTATCAATGAAAATATGTGATTGACACAATAGTCCTTGCAAATGTGCTGCTCCTTTATCTCTTCTGATGAAGGGTGTTTA
ATACTGGATGCAATGAGGAGGAGGATTGGTTCAGGTAGGTTACTGAATGGACTCTGCTAACTGGAAGGAGCTAGTGCACAGTCTTCACAACACCCACCAA
GGACTGTAAGAAATGAAAATTGTAATGAAATTTGTAACATTGTTTCCATTCTTTTAGATTTGGACATACTTAGCTAGGACTGAAACTTTAAGCAATTCGA
ACCCTGCTATTGATGTGAATAAGAATTCAATTTGTTACATTTGCTTTCAAAGCATAGAATTATCCTGGTTCAAGTAGGCTATGCTATATGCTAGCGTCAG
GCAGTTTGATCTATTTCCTCCCCTAGCTACGCCCCGTCGTATTCACCATTTACACTTAGTTGTCTTCCTAGTTTAAGCTCTTGAGCACAGCAGTAGTTGA
AATTAAACAGAAAATCAATACAATAGAATCTATAAGTAGGGGTTGGTTTCAGAGAATAATAATAATAATAAGATCATTGCTGTCTCCAAATAGGCAAGAA
GAACTAGAGCAGTACCAGCATA

>Glyma.06G318700 | Chr06:50734144..50738368 reverse
TGAAAGTGAGTGAGATAAAAGAGAACAAATTGATCGATAGAGGCAGTGATGGAAGAAGGTTCAGAGACAGGAAAGTGGGGATGGATGAAAAGGAGGAGAG
CAATGAGGGAAGAGCTGAAGAAGGTGGGTACTATAGCAGCTCCAATGGTGGTTGCAAGTGTGTTGCAATATCTTTTGCAAGTGGTGTCCTTGGTAATGGT
TGGCCATCTTAACCAGCTCTCTCTTTCAACTGTTGCTATTGCAACCTCTCTCACCAATGTTTCTGGGTTTAGTGTTTTGGTAAGTAGGTCTTTGCATGAT
TTAATCATTACATTTTTAATTCATTCTCTTATTGATCCTATATTTATGATATGTTTATAAATTATTTTTGACTTATTTGAATAAGATTTTCAAGAAAACT
AAAGAATAGCTCATAATTTATATGAAAGAAATTTAATTAATAAATGAGTAATAAAATGGATTATTTAAACACGTTTTAAATTATTAGTGCTAATTACGAA
TAAATAAAAATAAATATACAATTTATAAAAGAAATCAAGAAACATTGAGTGTAAAAGAAATTTTCCTTACAGAAAATGAGTTTGGAAACTATGATAAAAC
TAATCTCAAAACCTTTAAGCCTTTATTCTATGTCAAGTTGTACAAATAACAAGGATTGCTCATATGGTATCTAATGGCAGGACAAATCTTCAAACTTACA
GAATATTTATAAAAAAAACTTACATTTATCTTATTTATGGAGACAAATGAGGATTTAAATTTATAACTTGTCATATGTGAGACTAACTTATACTCCACTT
AATCAATCTATTTGTTAATTGTTATATACGAACACTTGATGATTAAAAATTTGGTGCCTTATGTTGCATTAAAAAAACAAAAGGTATCTTAAGTTGTATT
TAAAATGATAGACACCTTTTCATGTTTTGGGTCCAAATTACAGTTTAGTAATGTAGTCTAGTCAACTTAGTCAACGGGTCAATTTAGGCCTTTTGTGTTA
TTTTTTATGAATCATTTTTATGACTATATCTTTTTTTAACCAATTGTCTTAATATTTTATAAAAATAAGAACATTAAATATTTTGAAAATATAAACAATA
TTTAATATTTTTCATCTAATAAATATAGGATCAATAAGAGAATTTGGAGTTATTTAATTTTCTTTCATATTCTTAAAACAAATATTTTTCGAACTTGTGA
AGAATTTTATCATTAATTTTTTGGACTTATATTCTTTCATGACAACTTTCTTATAATTTAAAAGGATGAAAAAATAATTATAACAAAATCTCTAATTGTT
TTCATATGATTTATGATTTTTAATAAGAGAAAAATGAGAATATATATATATATATATATATATATATATATATATATATATATATTCTCCTTTTGTTACC
TTTGTATCAACGATTTAGATGTTCTGGGAGAATTTTCTTCGAAAAAGAGTTTTAGAATTGCGTAAGTACTCTTAATTTGTTTTATATGACATAATCAATT
TGATCAATTGTTTTTTTGAGAAATTCTATAGTACTCTTAAAAATTTAAAGATATTAGTATAGTAATAGAGAAACTCTTTCTCTCCTTGACTCCTTCTTGG
TGTACTCAAATTCATTTAACTATTGACACCAAAACCAACTATACTAAAGCCTTTAGATTTCTCCAAGTACGATAATAACTCTCTTATTTTTTGTCACAAA
ATAACAAAACAACATTTTAAGGGAAGATGTGTTCATAAGGAGTAGTATTATGGAGCATTCTTATGTCAAATTTGTAAAACCAAATCGTTTGAAATATAAT
GTGCATGCTCATTTAGTTATATTTATGTCAATATTGAAGTCAGGGATGGCTGGTGGATTGGAAACTTTAGGTGGCCAAGCTTTCGGGGCAGGGCAATATG
AAAAATTTGGACAATATACTTACACTGCCGTAATCTCCCTTTCTTTGATTTGTTTCCCAATCACAATTCTATGGACTTTCATGGACAAAATATTAACACT
CTTAGGCCAAGACCCAACAATTTCCCTTGAAGCTCGTAAATATGCCATTTGGCTAATACCTGCTCTATTTGGTTCCGCAATTCTCAAACCTCTAACACGA
TTTTTCCAGACCCAGAGTTTGATTTCTCCCATGATTCTAACCTCTGCCATAGCTTTGTGCTTCCACGGAGCAACATGTTGGACCCTTGTATTTAAATTGG
AGTTGGGACATGTTGGTGCTGCAATCTCCTTCAGCCTTTGTGTTTGGTTTAATGTGATGTTGCTTTTGTCCTTTGTGAGGTATTCCTCTGCTTGTGAGAA
AACACGCATCCCTTTCTCCAAGAATGCTTTGGTTGGTGTTGGAGACTTCTTTCGCTTTGCTGTCCCAGCAGCAGTCATGGTTTGGTAACTATTCTTTAGC
TTTACTATCCATACACTTTTTCTTCAGAAACATTTTATTAATTAAAATAGCTACAACATATCTTAATTAGTCGACTAGTTTGACAAGATTTCCCCGCACC
ATCAATTATAATACACAAAATTTTATCACTTTCAATCAATAAAAAAGAGTATGTTACTAGTATTTCTCTAATGATTTGCCAACATGGTCACTTATCTGTC
ATACAATTTTTCGTCTTACAGTCTTAAATGGTGGGCCTGCGAGATACTTGTTTTGCTAGCCGGACTTTTTCCAAATCCAAAGTTGGAGACATCAGTCCTA
TCTATATGGTAATGCTTAATTTGATTTTTTTGAAGAAATTAAGCTAACATTATTTAGATGAAAACGAAATTAACCACTTTTTTTTTTTTTAATATTGCCA
GCTTGACAATCTCTACATTACATTTCACCATACCTTATGGGTTTGGGGCTGCTGCTAGGTTTGTTTCTCAATCAAACTCTAAGGTCATTTTATTTCCATT
TGCAAATATTAGGATTGAAGCATAGTAAAATGTGACACATTAATTCTATGAGATAGCACTAGAGTTTCGAATGAATTAGGAGCTGGGAATCCACAAGCAG
TTCGTGTGGCTGTTTCAGCAACGATGTTCCTTGCAGTCACAGAGGGTCTAATTGTAAGTGCAACACTATTCGGCTGCAGACATATCTTGGGTTATGCCTA
TAGCGATGATCGTATGGTTGTTCATTACGTGGCTGTTATGACCCCTCTGCTTTGTCTCTCTATTTTTACTGACAGCTTACAAGGAGTTCTTTCAGGTAAA
TTATTCTCCTTTATAATTTTATGCTTGTTACTTTTGAATACTGCACAGTAAAATAAAGCTAACAAGAAAAATGCTAGAAATATACTCTTCCATTCACCTC
ACTAAATATGTTGTAAGTCATATTCTTTATTTGATGGGGATAACATTCCTATATTGATTAGAGTGGTTTAAATTTTAACTAGTAAGAGAAAATTTGTTAA
AATATGTTACTTGTAGAAGTAATATGTTACCAATGCATTTGAATGGTACTTGTAGAAGTAATATGCATTTGGTGTGTTAATTGCCTCTTATATTTTCTTG
GTAACTATCATTCTTATTTAGCTAACCTGCTACGGAAATTAATTAATTAATTAATCAGGGGTTGCTAGAGGAAGTGGGTGGCAACATCTAGGAGCCTATG
TCAATCTGGGAGCATTTTATCTGGTAGGAATTCCTGTAGGTATCGTACTTGGCTTTGTTGCACATTTAAGAGCAAAGGGCCTCTGGATTGGAATAGTCAC
TGGCTCCATTGTCCAATCGATTCTCCTTTCCCTTGTTACTGCTCTTACCAACTGGAAGAAACAGGTTCACGATTTATAAATTTGACACTATCCTTAATAA
CCCAATATGATTTCTTTGTTGCAATGCAGAAATATTGTATGTTTGGTGTTAATTAGGTGTCGGATTTGTGCAATTTTTGTAGGCAATGATGGCAAGGGAG
AGAATCTTTGATGTTAAACCACCAGACGAAAATGAATCAAATCACATGACGAGTGCTTAAAGCTAGTTGGTCAGCTGTGTCTAACATAGACAGAGATATA
CATACATGATATATAATTTTGGTTTTCCGTTGGAAATCTGTCACCAAAGTATGGTTTTCGCAAAGAAATGTTATGAGACACCTACATCAACATCGAGAGA
GACAAAGAAATAAATGAAAGCAAAGACATAAATATGTAATAGATAATATAATGTTACAAGAAAAATAGAGAAACACACCAGGAATGTACGTCAAACAAGT
AGTGTTTGTAATGATGGAGTGTCTA

>Glyma.18G293100 | Chr18:57124192..57128652 forward
AAAGAAAGATAAGATATATAGAAGAAAAGGTAATATATAACACTTGAAAGTGAGTGACAAAAAGAGAACAAATTGATCGTAGAAAACTGATAGAGGCAGT
GATGGAAGAAGGTTCAGAGACAGGAAAGTGGGGATGGATGAAAAGGAGGAGAGCAATGAGGGAAGAGCTGAAGAAGGTGGGTACTATAGCAGCTCCAATG
GTGGTTGCAAGTGTGTTGCAATATCTTTTGCAAGTGGTGTCATTGGTAATGGTTGGCCATCTTAACCAGCTCTCTCTTTCAAGTGTTGCTATTGCAATCT
CTCTCACCAATGTTTCTGGGTTTAGTGTTTTGGTAAGTAGGCCTTTGATTTAATCATTACATTTTTAATTCATTCTTTGTTTCTATTTTATGATTATGTT
AAAATCACTTTTAAACAAACTTATTCAAACAAAAACTGATTTTAATTATAATAAATTATAACAATAACCAATTATACATATACAATTAATTACTAAAATA
ATAATACATTTTTGCAGTTGTCTCAAATACGTACATCCTAAGAGTGTCTTTTGGAAGATTTATTTACAACTTATTTGAATTTATTTTATGCTATAAATTT
TTATGTAGGAGTAAGTATTAATAAAAAAAAACTTGTAAAAATAACTTATGATATGTTTATAAATTATTTTTTAACTTATTTGAATAAGATTTTCAAGAAA
ACTAAAGAATAGCTCATAATTTATATGAAAGAAATTTAATTAATAAATGAGTAATAAAATGGATTATTTAAACACGTTTTAAATTATTAGTGCTAATTAC
GAATAAATAAAAATAAATATACAATTTATAAAAGAAATCAAGAAACATTGAGTAAAAGAAATTTTCTTTTCAGAAAATGAGTTTGGAAACTATGATAAAA
CTAATCTCAAAACCTTTAAGCCTTTATTATATGTCAAGTTGTACAAATAACAAGGATTGCTCATATGGTATCTAATGGCAGGACAAATCTTCAAACTTAC
AGAATATTTATAAAAAATACTTACATTTATCTTATTTATGGAGACAAATGAGGATTTAAATTTATAACTTGTCATATGTGAGATTAACTTATACTCCACT
TAATCAATCTATTCGTTATATATGAACACTTGATGATTAAAAATTTGGTGCCTTATGTTGCATTAAAAAAACAAAAAGTATCTTAAGTTGTACTTTTACA
TGATAGACACATTTTTATGTTTTGGGTCCAAATTACAGTACTGTGGTCTAGTCAACTTAGTCAACGGGTCAATTTAAGCCTTTTGTATTATTTTTTATGA
ATCATTTTTACGACTATATCTTTTTTTAACCAATTGTCTTAATATTTTATAAAAATAAGTACATTAAATATTTTGAAAATATAAACAATATTTAATATTT
TTCATCTAATAAATATAGGATCAATAAGAGAATTTGGAGTTATTTAATTTTCTTTCATATTCTTAAAACAAATATTTTTCGAACTTGTGAAGAATTTTGT
CATTAATTTTTTGGACTTATATTCTTTCATGACAACTTTCTTATAATTTAAAAGGATGAAAAAATAATTATAACAAAATCTCTAATTGTTTTCATATGAT
TTATGATTTTTAATAAGAGAAAAATGAGAATCTATATATATATATATATATTATTTATTTTTTTCTCCTTTTGTTACCTTTGTATCAAGGATTTAGATGT
TCTGGGAGAATTTTCTTCGAAAAAGAGTTTTAGAAATGCGTAAGTACTCTTAATTTTTTTTATATGACATAATCAATTTGATCAATTGTTTTTTTGAGAA
ATTCAATAGTACTCTTAAAAATTTAAAGATATTAGTATAGTAATAGAGAAACTCTTTCTCTCCTTGACTCCTTCTTGGTGTACTCAAATTCATTTAACTA
TTGACACCAAAACCAACTATACTAAAGCCTTTAGATTTCTCCAAGTACGATAATAACTCTATTATTTTTTGTCACAAAATAATAAAACAACATTTTAAGG
GAAGATGTGTTCATAAGGAGTAGTATTATGGAGCATTCTTATGTCAAATTTGTAAAACCAAATTGTTTGAAATATAATGTGCATGCTCATTTAGTTATAT
TTATGTCAATATTGAAGTCAGGGATGGCTGGTGGTTTGGAAACTTTATGTGGCCAAGCTTTCGGGGCAGGGCAATATGAAAAATTTGGACAATATACTTA
CACTGCCGTAATCTCCCTTTCTTTGATTTGTTTCCCAATCACAATTCTATGGACTTTCATGGACAAAATATTAACACTCTTAGGCCAAGACCCAACAATT
TCCCTTGAAGCTCGTAAATATGCCATTTGGCTAATACCTGCTCTATTTGGTTCCGCAATTCTCAAACCTCTAACACGATTTTTCCAGACCCAGAGTTTGA
TTTCTCCCATGATTCTAACCTCTGCCATAGCTTTGTGCTTCCACGGAGCAACATGCTGGACCCTTGTATTTAAATTGGAGTTGGGACATGTTGGTGCTGC
AATCTCCTTCAGCCTTTGTGTTTGGTTTAATGTGATGTTGCTTTTGTCCTTTGTGAGGTATTCCTCTGCTTGTGAGAAAACACGCATCCCTTTCTCCAAG
AATGCTTTGGTTGGTGTTGGAGTCTTCTTTCGCTTTGCTGTCCCAGCAGCAGTCATGGTTTGGTAACTATTCTTTAGCTTTACTATCCATACACCTTTTC
TTCAGAAACATTTTATTAATTAAAATAGCTACAACATATCTTAATTAGTCGAGTAGTTTGACAAGATTTCCCCGCACCATCAATTATAATACACAAAATT
TTATCACTTTCAATAAATTTCAATCAATAAAAAAGAGTATGTTACTAGTATTTCTCTAATGATTTGCCAACATGGTCACTTATCTGTCATACAATTTTTG
GTCTCAGTCTTAAATGGTGGGCCTGCGAGATACTTGTTTTGCTAGCTGGACTTTTTCCAAACCCAAAGTTGGAGACATCAGTCCTATCTATATGGTAATG
CTTAATTTGATTTTTGAAGAAATTAAGCTAAGATTATTTAGATGAAAACGAAATTAACCACTTTTTTTTTTAATATTGCCAGCTTGACAATCTCTACATT
ACATTTCACCATACCTTATGGGTTTGGGGCTGCTGCTAGGTTTGTTTCTCAATCAAACTGTATGGTCATTTTATTTCCATTGGCTAATATTAGCATTGAA
GCATAGTAAAATGTGACACATTAATTGTATGTGATAGCACTAGAGTTTCGAATGAATTAGGAGCTGGGAATCCACAAGCAGTTCGTGTGGCTGTTTCAGC
AACGATGTTCCTTGCAGTCACAGAGGGTCTAATTGTAAGTGCAACACTATTCGGCTGCAGACATCTCTTGGGTTATGCCTATAGCGATGATCGTATGGTT
GTTCATTACGTGGCTGTTATGACCCCTCTGCTTTGTCTCTCTATTTTTACTGACAGCTTACAAGGAGTTCTTTCAGGTAAATTATTCTCCTTTATAATTT
TATGCTTGTTACTTTTGAATACTGCACAGTAAAATAAAGCTAACAAGAAAAATGCTAGAAATATACTCTTCCATTAGAGTAAAATTCACCTCACTAAATA
TGTTGTAAGTCACATTCTTTATTTGATGGGGATAACATTCCTATATTGATTAGAGTGGTTTAAATTTTAACTAATTAAGAGAAAATTTGTTAAAATATGT
TACTTGTAGAAGTAATATGTTACCGATGCATTTGAATGGTACTTGTAGAAGTAATATTCATTTGGTGTGTTAATTGCCTCTTATATTTTGTCGGTAACTA
TCATTCTTATTTAGCTAACCTGCTACAATTAATTAATTAATCAGGGGTTGCTAGAGGAAGTGGGTGGCAACATCTAGGAGCCTATGTCAATCTGGGAGCA
TTTTATCTGGTAGGAATTCCTGTAGGTATCGTACTTGGCTTTGTTGCACATTTAAGAGCAAAGGGCCTCTGGATTGGAATAGTCACTGGCTCCATTGTCC
AATCGATTCTCCTTTCCCTTGTTACTGCTCTTACCAACTGGAAGAAACAGGTTCACGATTTATAAATTTGACACTATCCTTAATAACCCAATATGATTTC
TTTGTTGCAATGTAGAAATATTGTATGTTTGGTGTTAATTAGGTGTCGGATTTGTGCAATTTTTGTAGGCAATGATGGCAAGGGAGAGAATATTTGATGC
TAAACCACCAGACGAAAATGAATCGAATCACATGACGAGTGCTTAAAGCTAGTTGGTACAGAGATATACAATCTGTCACGAAAGTATGGTTTTCGCAAAG
AAATGTTATGAGACACCTACATCAACATCGAGAGAGACAAAGAAATAAATGAAAGCAAAGACATAAATATGTAATAGATAATATAATGTTACAAGAAAAA
TAGAGAAACACACGAGGAATGTCAAACAACTAGTGTTTGTAATAATGGATGGAGTGTCTAT

>Glyma.02G090100 | Chr02:7925640..7933611 reverse
ATTATATTTTTTTAATCCATACAATTCGAATCAAAATTTTAATTTAAAGAGATTAATCATTCAATATCAGATAAATCACTCAGATATATATATGTATGTA
TATGTATATATATCAAATCTCAATGAAGCATCTGCAGATTGATCCCGTCTATGATTTAGTTTTTAATAATATATATGTAGGTTTGTATAAAATATTTTGA
TACATTGATTATACAAGTTGTTAGGTATACATTATATAAATATGGCGTACAAATATATTTTGATTTGCTATTGGATGATTATGTTCTAAATTTTGACATA
TACACTGCTTTAAGTTATAGAATGTTTACAAAATTACGGTCTAAAAATCATTTAACATTTGTTCTGCAACTTAAACTGAAATAGAAATTGCAAGTTGTAC
GTTCAAAATATCTACATTAATATTTTGTTGAAATGTGTATAGATTGTCGTTACTAGTTTGTATGTTTATTTGCTTGTTATTTATTAAGGTATAGTTGCGT
GGGTGACTGAAAAAGATAACGCAAAGTTGTGTTTAGTTGTGATGATACAAAAGAGGACAAAGGTAGAGGCAGAGCCAAAGAAGGATAAGATCGACATATA
GAAGAAAAGGTAATAATATATAACACTTGAAAGTGAGTGATATAAAAGAGTAAAAATTGATCGTACAAAAATGATAAAAGGGTGCAGTGATGGAAGAAAG
TTCGACAGGAAAGTGGGGATGGATGAAAATGAGGGAAGAGCTGAAGAAGGTGGGTACTATAGCAGCTCCAATGGCGGTTTCAAGTGTGTTACAATACCTT
TTGCCAGTGGTGTCCTTAGTAATGGTTGGCCATCTTAACCAGCTCTCTCTTTCAAGTGTTGCTATTGCAACCTCTCTCACCAATGTTTCTGGGTTTAGTG
TTTTGGTAAGTAGGCCTTTGATTTAATTTTTAATTCATTGTTACTATTTTTTTTATTACCCTGTTCAAAGAAACAAATTCATTGTTAATTACTTTTAGGC
TTGTCAACAATCTTGCTCTACTCAATTAATCTATTCGGGTTGAACAAATGGTAAGTTAAACGAACACATGACGATAAAATTTTGGTGTCTTATTGCATTA
AAAAAAATTATGATCTTAAGCTGTCTTTTGTATTGTTTTTTATGAATCATGTGTAAGACTATATCTTCTTACATGATTCTTTTACCAATTGTCTTAACAT
TTTATCAAAAAATAATAAAAAAGTACATTAAATATGTTGAAAATATAAATAATATTTAATATTTTTCATCAAATAAATATAGGATCAATAAGAGAATTTG
AAATTATTTAATTTTCTTTCATATTCTTGAAACAAATATTTTTTGGAACTATGAAAAATTTTATCATTAGTTTTTTGGACTTATATTCTTTCATTACAAT
TTTCTTAAAAGGATAAAAAAAAATAGTAGTAACAAAATCTCTATTTTTTTCATATGATTTTGTTGGACAAGTAGCCTCAATAACTTAAGAGGGAGTGAAT
TAAGTCTTACAAAATTTTTCACTAACAAACTTTTAATCTCCTTTTAAATGATAGGCTCAAAATTCAAAAGAAGAAGCAACAATCAATTTAATAATGATTT
TTAAACGTGCAAGATAAAATTGATTGCAATAAAATAAATAAGATGAGGGAACAGAAAATTGCAAACTTGATTTATACTGGTTCGGCCACTTCCCGTGCCT
ACGTCCAGTCCTCAAGCAATTCACTTAAGATTTTCTACTATCTCTGTAAATCCTTTATAGACTTTGAACACACCTTAGGATCCCTCACCCTTGTGTTTAA
GATTCTCCAAGAGACAATCAGTCTCTTGATTACAATTCTCAAAGTCCAAGAGACAATCAGTCTCTTGATTACAACTGAGTTTCTGAGATGAATAGAAAGA
TTTCTCTCCTTTAGAGTAGATGATACAAATTGAAGATTCTAGAGAAATTTCTCTCTTTTAGAGATGATAATACAAATTGAAGTTCTTGGATAAATTCTCA
ATAGATTTGTAAGTGTTTGTCCAATAGTTGTTGAGAGAACATTTAACAATTAAGTTCTCTTAGAATATTTCTCTCTTACTTTTTGAAGTCAAACACACAT
TTATATAGGCTCATCGTGCCTTTGCAAAATGATTTGAAGATATGTGTCTTTTCAAAAAGTTTTTCTGAAATTTTCTCATTGGTAATCGATTACAGGATAC
TCGTAATCAATTGCACAGTTACATTTTGATGGGTCATGACTTTTTAAATTGAATTTAAGGAGTTCCGTTACTGGTAATCGATTACACACCAATAGTAATC
GATTATAACTTTTAAATTCAAATTTTAAAATCCTTTTTAAAAGCTAATTTGCAAACTTGTTTTCTGGTAATCAATTACACTGTCTGGTAATAGATTACCA
GTGTCTTGATTTATTGGAAACAATGTGTTTGAGACAAAAGCTTATCAACTAGTGAGATTCTTTGAGACCTTATCTTTTTCTTGATCTTGTATGCTTAATC
TTGAATTAATCTTGAAGCAATGCTTAATCTTTGAATGTTTGTTGAAGCAATCTTATTTGATTATACTTTTGGCATCACTAAAACCCTGTATTCATATATT
TACATTCTCCACCTTTTTGATGATGACAATCATTATCAAGTGAATTCTTTTCAGCATCATCCAAACCTGTATGATTAACATATTTATGATTTTTAATAAG
AGAAAAAGAGAGAAAAAAATGAGAATCTAAAATCCAAATATATTTTTGTTTATCTATTTTTTTCTCCTTTTGTTACCTTCGAATCAACGATTTAGATGTT
TTGGACGAATTCTCTTCGAAAAAGAGTTTTCGAATTGAGCAAGTATTCTTAAATTTTTTTATATGACATAATCAACTTGATCAATTATTTTTTTTTTTTG
AGAAATTCTATAGTACTCTTAAAAATTTAAAGATATTATTCTACTAATAGAGAAACTCTTTCCCTCCTTGATTCTTTCTCTTGGTGTACTCAAATTCATT
TAACTATTGACCCAAAAAACAACTATACTAAAGCCTTTAGATTTCTCTGAGTACAATAACAACTCTCTTTTTTTTGTCACAAAATAACAAAAGACGTTTC
AAGGGAAAATGTGTTCATAAGAAATAGTATTATGGAGCAATCTTATGTGAAATTTGTAAAACCAAATTGTTTGAAATATGACTCATGACTTCTATTTATG
TCAATATTGCAGTCAGGGATGGCTGGTGGATTGGAAACTTTATGTGGCCAAGCTTTCGGGGCAGGGCAATATGAAAAATTTGGACTATATACTTACACTG
CCATAATATCCCTTTCTTTGGTTTGTTTCCCGATCACAATTCTATGGATTTTCAATGACAAAATATTAACTCTATTAGGCCAAGACCCAACAATATCCCT
TGAAGTTCGTAAATATGCCATTTGGCTAATACCTGCTCTATTTGGTTCCGCAATTCTCAAACCTCTAACACGATTTTTCCAGACCCAGAGTTTGATTTCT
CCCATGATTCTAACCTCCGCTATAGCTTTGTGCTTCCACGTAGTAACATGTTGGACCCTTGTTTTTAAACTGGGGTTGGGACATGTTGGTGCCGCAATCT
CCTTCAGCCTTTGCGTTTGGTTTAATGTGATAATGCTTTTGTCCTTTGTGAGGTATTCCTCTGCTTGTGAGAAAACACGCATCTCTTTCTCCAAGAATGC
TTTGGTTGGTGTTGGAGAGTTCTTTCGCTTTGCTGTCCCAGCAGCAGTCATGGTTTGGTAACTATTCTCTCGCTTTGCTATCCATACACTTTTTTTTAAG
AATATTTTATTAATTAAAATAAATCTCCTTATACTATTTTTTATTTCTGCCAATACAAAAAAAAAAAGTCTTGAGTATTTTGAGAAGATTTTGCAACACG
ATCATAATGATTTGGCCAACATGGTCACTTATCTGTCATACAGTCTTAAGTGGTGGGCCTGCGAGATACTTGTTTTGTTAGCTGGACTTTTTCCAAACCC
AAAGTTGGAGACATCAGTCCTATCTATATGGTAATGCTTAATTTGATATTTGAAGAAAGCCCATCTTTATTTAGATGAAAACGAAATTAACCACTTTTTT
TTTTTTTTTTAATATTGCCAGCTTAACAATCTCTACGTTGCATTTCACCATACCTTATGGGTTTGGGGCTGCTGCTAGGTTTGTTTCTTACTTAATCAAA
CTATATGGTAATTTTTTTTTCCATTTGCTAATAGTAAAAAGTGACACATTAATTAATACTATGTGATAGCACTAGAGTTTCGAATGAATTAGGAGCTGGG
AATCCACAAGCAGTTCATGTGGCTGTTTCTGCAACGATGTTCCTTGCAGTCACAGAGGGTTTCATTGTAAGTGCAACACTATTCGGCTGCAGACATATCT
TGGGTTATGCCTATAGTGATGACCGTATGGTTGTTCATTATGTGGCTGTTATGATCCCTCTGCTCTGTCTCTCTATTTTTACTGACAGCTTACAAGGAGT
TCTTTCAGGTTAATTATTCTCTCCTATTCTTTTTTTGCTTGTTGCTTTTTGAAAATTAAATTGTACGTTGGTGCTTTCAACCAACTAGGTTACTCTTGAA
TGCTGCATAGTAAAATAAAGCTAACAAGAACAAAAAATTGCTAGAAATACACTCTTTTTTGGTTAAACTAGAAGAAATGCACCACTCTTTGATACTTTTA
ACAGACGCAGCCTCTCCCATAATCATTAGAGTAAAATTCATCTCACTAAATATGTTGTAAGTCACATTCTTTATTTGATGGGGGTAACATTCCTAAATTG
TTTGGAGTGGTTTAAATTTTAACTAGTAAGAAAGAATTTGTTAAAAAAATAAGTGTTAAAAAATATGTTATCAACATTTCTAAATTGTTAGGATGGAGTC
ACCTAAATTTCAAAAGCGGGTTTGTTAAAAAGATAACGTTAGAAAATGTGTTACCAATGCATTTGGTGTGTTATATTTTGTCAGTAACTATCATTATTAT
TTTGCTAACCTGCAACGAAAATAAATAAGTAAATAGATAAAATAATCAGGGGTTGCTAGAGGAAGTGGGTGGCAACATCTAGGAGCCTATGTCAATCTGG
GAGCGTTTTATCTGGTAGGAATTCCTGTGGGTATCCTACTTGGCTTTGTTGCACATTTCAGAGCAAAGGGCCTCTGGATTGGAATTGTCACTGGCTCCAT
TGTCCAATCGATTCTCCTTTCCCTTATTACTGCTCTTACCAACTGGAAAAAACAGGTTCACAATTGATAAATTTGACACTATCCTTAATTATCCAATATG
CTTTCTTTTTTGCAATGTAGAAATATTGTATGTTTGGTGTTAATTAGGTGTCGGATTTGTGCAATTTTTGTAGGCAATTATGGCAAGGGAAAGAGTATTT
GATGCTAAACCACCAGACGTAAATGGATCATATCACATGACGAGTGCTTAAAGCTAGTTTGGTCAGCTGTGTCTCACATAGACATAGATATATAATTTTG
ATTTTCCGTAGGAAATAAGTCACCAAACTATGGTTTCGCAAAGAAATGTTACGAGACACCTACGTCAACATCTAGAGAGCCAAAGAAATAAATGAAAGCA
AAGAAATAAGTAACAGATAATATAAATGTTACAAGAAAAATAATGGAGTGTTTGTAATAATGGAGTGTCCATGCATGACTGTTTTATTATATTATTCTAA
TTTGGTCAAAATGCTGGATCAACTCCAATTAGAGATTATTAAAACTATGGGCGCAAGTTCACATTGGTTAATTATATGTCTGCTATATGATACCCAGTAG
GAATGGCGACCGATCTTATCCATAGTAAGCTATTTTAACTCGTGAAACATGATTGGACTCATCCAAATAAATTATGGAAGATGGTGGCATTTCTAAATCC
AACTCATCAGAGGTATTTGGCATATAATATACATTGATTAACTTTAAAAATGACCTGCTTTGGTGTTTTATTCTTTTTTCTCTTACTCCTTTAAATATTT
CTTTTTGTTCTTTAAATATTTATTGATTGACAAATATGTTAAACAGAAAACTTAAAACAATGGTTAAAACAACAAAACTAAAAATATTGTTAAAAAAGAA
AATTTTATTTAGGTCAACAATATTTTTCAACTATACATTTCAATTTCTCTTTAAAAACACCCTTACTTTTTATGGTGAGATAATTATTGTTTGCACAGTA
TTTTAATCAACTTCTCTAATATAAATTTGAGTTTTCATTTTCAAACTTGTAGATGTACGTCTTATACTGAATTTTCAATTTTCTTGTAAGAAATAGCTAC
TTATTTTTATATTTTCAACTATTTTAAAGTATCAAATAGCTACTGATCAGTTTGGGCTATTCATAAAAGAAATTTGATGAGTAACTTCTTAACTTCAAGC
CCACAAGTACAGGGGTGTATGTAGATGTTGGAAGTTGCATCACATAATTCAATCAAATTTAAATAGGTTAGTTTGAGTTGATTTTTTAAAAAAGAAAAAA
AATTAATTAACTCCAACCAATTTAGCTTGTTTGTAAACAATTTTATTTGAATTGAACTAATGAGTAAAATATTTTAATTTGCCTATTAACATATTTAAAA
TTTAATATTTTTCACTATTACATACAATGCAAGTTCTTCTAGGACACTACATTAAGGACGGTTCTACAAGAGGGACGGTTCCAAAAACTGAATGATATTT
TTGTAAATAATCATAATTGTTCAATGATGATTCTCACGAAATTGTCTTAAAAACATATTTTCAAATATAAATTATTTTTTTCAAAACCGTTGTTAAAATG
GGATACTCAACATCTCATTTTTTACTGGACCCGTTGCGAATGTGCCTTCAATTTTTATTTTCACTCTGGCTCTACTCGTTTTCATTCCCCTCTTTCTCTC
GTGCTCACATACCTCACTCTTATGGTCTCTCTGCCAAGGTAAGAACCCTCCTCCAAACACCTTCACTGCCCTCCTCCAACATCCCCGTCATTTCCCAAAT
TAATAGAAAGTAAGAAACATAAATGAGTTTTCCTTTCATTTTGTTCTTCAACAGAGCTTGTATCATCTTTTTCCTTAAATTCATATCAAAGATATTCATT
AAGCTAAGAAACTCTTTCCAAGCCATGTGTTACTTAAGATGACAGATAATGTTATTTTATATGGAGAACCATTAAATATTATTATAGTTACTCTTATAAT
TAGGATTGTTACTCTTATATCCATGTGTAGAAACAATACGATCATAAAACCATAATAGATCTATGAATTATAACAATTGAAGCTTGAACTAATGGTGTAA
AAACAGAAGCCGCAAAAAGGTTATAACTCTAAAACAAAGGAAAAAAAAAAAAGATTAAAAATAGCTATCACCATGATAGAAAGGATATAGTAAAAAATAT
AATGACAACAATGTCTGGAATATTTTTCTTATTTTATAGGAAAAAAAGAATGAAACCAATGCCTATTTGGTATTAACTCATTTGAGTAGTTTTACTTTTT
ATTTTTACTTTGCTAACAATGCTTGCAGGATAAAGGTAAAAAAATAATGAAGCTTTGTTATATGTAGTTTAGATAATTAATTTGCTTATTTACAGTATGA
TTATTCAAAAGCATTTAATTTGCAGGTATTGAACCTAACCAAAGCATATGTTGAAGGGCTAGGAGAAGGTGGTTTTGGATAAGTTTTTAAGGGGTGGATC
AATGAGAATTCATTGACTGCTACCAAACTTGGCACTAGCATTGTTATTGCTATTAAAAGACTCAATCAAGATGGTATCCAGGGTCATAGGGAGTGATCCG
TAAGTTTAATCTAATCCTCTAGCTTTGTTCACACTTCATACTAATTAAGAAATAAATGTTACATGTGTTACACCATAGTAAGCACAATGCATGGTTAATG
AGTACACTGCTAGCTAAAATATTCACAAACTATGCTAGTCAATTTAGACTCTGCTTACCATCTGTGTCGGAG

>Glyma.18G110800 | Chr18:13125204..13125542 forward
ATGGATAAATTGCTTGTTCTTTTGGGGCAAGATCATGCTATTTCACTAGTAGCTGGAAACTACTGCATTTGGCTCATTCCTGCACTCTTTGGTTATGTTG
TTCTTCAAGCTTTGGTTCGTTATTTTCAGACTCAGAGCTTGATCTTTCCAATGCTTGTAACCTCAGTTGTTGTTTTAGTTTTGCATATACCTATTTGTTG
GGTACTAGTGTTTGAACTGGGACTTGGACAAAATGAAGCGGCCTTATCCATTGGAATTTCGTATTGGCTCAGTGTCATGTTACTTATAGTTTACACAGTA
TTATCTGTCATGTCAGAAAACTTAGGTAGCTTTAGGTAG

>Glyma.18G293300 | Chr18:57136449..57140783 forward
ATGGAGGAGACCTTATTGTTGCCAAAAGAAAACAAGAGGGTGAGCTCAAACTCAAAGAGTAGTAGTAGTGGTAGTGGATTTGTCCAAGAATTTAAGAAGG
TGAGTTTGATGGCAGCACCAATGGTTGTGGTCTCAGTGTCACAGTTTTTGCTACAGGTGGTGTCATTGATGATGGCTGGTCGGTCATCTTGGCGAACTCT
CCCTTGCAGGTATTGCATTGGCCACTTCCTTTGCAGATGTCACTGGCTTTAACATACTTGTATGTTCCCTCCTTTCCTTTCCTCATTTTTACATCCAAAT
AATCGAGAGAAGTTAGTTTCTTAAATAAATTTATGTAATTTGAAGGATTAGTCTTAAAAGATAATATTCTTATTCTTAAAAGATAAATCAAAGTAGACAA
GCAGTCAAAACTTACTAGTTTCTACAAATTCTCGTGATGGAAAAATAATATTTTTTTGCTAGGACTCTGTGATCTCTATATGCTGAAAGAATAATGTTAA
CTCGTTGGCAAGTGAACCAAATCGTCACAAGTAGTAAAGTTCTCGGAAGTCCGAATGTCGAATCCACAGGGACTTTATTTTTACTTAAATTAATGCAAAT
CCAATTTAAAAGCAAGAGATAAGAATTTAAAATAAAAGATAAAGAAAGATAGAAGATAAGATATTTAAAGATAAGATTAGAAGATAAAAGAAAAGATAAA
AGATTGAAAGATCAAAATTGAAGATAAAATATTTAAATTGAGATATGATAAAGATAAGAATAACTACTTCTAAGAAAACCCAACAGTAAAATAAGGAATA
ACTAACAAGTCTAATTAGCCTAGATATATGGATTCTGGATTTGTCTCTTATCAATACCAGTGAACTAATTCTGCCCCACATCTATCCATCTACTTGCCCC
TGATGCCTCACGATGACAAGCCTACCTTAATTACCTATCTTCCAAATGCCCTTTGCGAAGACTCAATAACTAAGATGCTTTAAGATTACATTCTACATGT
TTGCTAAAGCATGGGCATTGAGGCATTAAGTCATGCCAACCCAATGATTTCTTTCTTTAATTATTCTATTAAGCGTTACCCTCTCCCGAGTGGCCTAACC
CTTAAAACCGATTCACGCATTCATTTCTTATCCCTAATTAGGCTTTACCCTCTCCCGAGTGGCCTAAAGACTAACAGAAAATAAGGTCCGAGATGCAGAA
TAAGCAAGAAAGAAAATCAGATATAAGAATCTGGAAGAAAAATCCTCTAAAAGATAACCCGATAATTTTCTCCTTTTAGTGTTCTCTTAAGCGTTATCCT
CTCCTGAGTGGCCTAACCCTTATTAGTCATTCATGGTCATGAGGGCTACAATGAGAGGTGAAAGAAAGAAAGGGAGTAAATGAAACCCCTAGGAGAGGGG
GTTCTGTGGTGGCGTCTGTCCCTTGGACTGACTTTCTATTTATAGCTGTTGAAGTGGGCTTTGGGCCTTCATAGGCGCGCTTAACGCGTGACGCGCGCTT
AGCACGCGTGTTGAGTTGGACCTGCTTCAGATTTTCTTCTTTTCTTCAATTTTCCTGCCCTTTTTGCTTGTTACACCTCCAATTTTTATATCTGCAACCA
AAAATTCAATTTAATTAATTTTCTAACTTTTAATCACAAACAACTGCTAAATAATTAATTTCAGGCCAATATTTGACTAATTTTCTACTATCAAAATACA
ATTATTTAGCAGTTATCAAATAAATTTAACGTCTTCCATTCAGCCTCGTGAGTAATTTTATATATTAATAAATGTATTGCTTATTTTTTTTTGGTTAAAA
TTGTAAATGTATTACTTTGAAATACTTACATATGTTTTTTGACTAGCTGTATAGCTTATATATTGATCTGACATATTTTCTAGCTGTCAATGCTATTTTA
GGGGTGCAATATTGCAATCACACATATTGGAACTTGAGTGTGTTTGTTTGTTTACTAAATGTGGTATTTGTTATGAACTGGCATGCTTAGACAATCTCCA
ATACATGATTTTTCTTTAGGTTCTTAAGTGGATTCCATTTGTACACATAGTGCATGATAACAACAGTTTTCTGCTTTCTTTAGTGTCATAGTGCCTTGGT
AGTTGACTGAACATGAAATGTAATGCAGATGGGAATGGCAGGTGCTTTGGAAACTCAATGTGCCCAATCATTTGGAACCGAGCAGTTTCATAAGCTTGGA
AACTATGTCTTCTGTGCAATACTCTTTCTCATTTTAAGCAGTGCCCCAAAATCTATCCTATGGATTTTCATGGATAAACTGCTTGTTCTTTTGGGGCAAG
ATCATGCTATTTCACTAGTAGCTGGAAACTACTGCATTTGGCTCATTCCTGCACTCTTTGGTTATGCTGTTCTTCAAGCTTTGGTTCGTTATTTTCAGAC
TCAGAGCTTGATCTTTCCAATGCTTGTAACCTCAGTTGTTGTTTTAGTTTTGCATATACCTATTTGTTGGGTACTAGTGTTTGAACTGGGACTTGGACAA
AATGAAGCGGCCTTATCCATTGGAATTTCGTATTGGCTCAGTGTCATGTTACTTATAGTTTACACAGTATTATCCGTCATGTCAGAAAACTAAGGTAGCT
TTAGGTAGCAATGCTTTAAGAAGCATCAAAGAGTTTTTCTTCTTAGCCATCCCATCTGCACTTATGATTTGGTAAGTTTCTAGTTTTGCCCCTGTATGAT
TGGAGAAATTATTACATGGTTTAGGATTACCATGGTCCAAGACCACCATGATTCCAACTATTTTTCCATGTGACATGTATACCGAGTTTAATTAACTCTT
TTTCGTTAATTGAAAATCTCAATTATATATCATGTAAAGATTGATCGAGACCATGGATATGTGGTGGTTCCAAACCATGTAACAATTTTTTGTATAATTG
GGGAGGCCAATGACTCGATGCTGCTTCTTCTCTATACTTTTTGTTTGTAACATATTTCATCTTTTTTTTTTTTTGAAAATAACATGTTTCTATCTTAATG
GATACATTCAGTTGAGTGGTGGTCGGTCATTAGAGCTACTAGTAATACTTGCTGGTCTTTTACCAAATCCCAAACTTGAAACTTCAGTTCTATCAATCTG
GTGTGTTTCTCCATGATCCTGTTATGTAAATATTTAAAAGTTCATTACCTGATAACTCAGGGATTCATCTGACTTATGTTTTCTTTGAGCAGCCTTAAAA
TTTGCAACTTGCACTATTTCATTCCATATGGGACCGGTGCTGCAGTAAGGTATGCTCTATATACACACACATTGATGTATTATGTAGGAGGCTTATATTG
TCGTGTGAGGAGATAATTTGATCATATCGCTGTATTGGATGGTCAAGATTAGATTTTAGAAAAGCAATCTTAACCATGCATATATAAAATATTATATGGT
CAGGATTGAAGTTTTCATGTTCTTACTAATACTCCTCATGTTATGCATCTTACTTTTGTGATTGTTATACACACTAGTGGAAACATCAAGCACATAATAA
AAGAATATCCTTCTATAATTTTGTAGTAGTCGGGTTTCAAACGAATTAGGGGCAGGAAGACCACAGGCAGCTCGAGAGGCTGTTTTTGCTGTAATTGTTC
TAACATTCACGGATGCAATTGTCTTTAGCTCTGTTCTATTTTGCTTCCGTCATGTGCTGGGATTTGCATTTAGCAATGAGATGGAAGTTGTACATTCTGT
TGCAAAGATAGTTCCAGTGCTTTGTCTCTCTTTCAGTGTGGATGGCTTTCTAGGAGTTTTGTGTGGTAAGTTTTTCACATTGACCGTTATAAATGTGCTG
TTTGAATAAATTTCTCATTAAACACTTTTAAGAGAAAAAAATAAGATGGTAAAATATATTGAGTTTCTCCCATAAGTTAAAATCAACTTATACACCTTAC
CTTTTGAAGGAATTAAATGAGAGAATTTTTACAAAAGTTAAGTCCATAAATTGATTTTAACTTATGAGAGAAGCTCAATTTATTTTATTTTTTTATTTTC
TTCTTCTCTAAGTGTTTATGGAGAAATCTATAAAAACAAAGCCACATGCTTGCCATTTCATGATTGTGCAATGAAAATACATTTTTAAAAGGGAATGACA
ATCATTTCACTCATTGATAACAACAGCTCTATGGTCAAAGCTAGTAGATATATTGTGGCAAATAAACATTAGGTTTGCAGCTCTCAGTTGTGTGTTCAAT
GAATCAAGAATTGTAAGAGGAAGTAGATTGCAGAAGATGGGGGCAATTAGCAATCTTGTAGCATATTATGCTGTGGGAATCCCTGTGTCTCTCGTGTTTG
GTTTTGGTCTTAATTTCTATGGGAAAGGCCTTTAG

>Glyma.16G149100 | Chr16:30978703..30980584 reverse
ATGGAGGAGACCTTATTGTTGCCAAAAGAAAACAAGAGGGTGAGCTCAAACTCAATGAGTAGTAGTAGTTGTAGTGGATTTGTCCAAGAATTTAAGAAGG
TGAGTTTGATGGCAGCACCAATGGTTGTGGTCTCAGTGTCACAGTTTTTGCTACAGGTGGTGTCATTGATGATGGCTGGTCGGTCATCTTGGCGAACTCT
CCCTTGCAGGTATTGCATTGGCCACTTCCTTTGCAGATGTCACTGGCTTTAGCATACTTGTATGTTCCCTCCTTTCCTTTCCTCATTTTTACATCCAAAT
AATCGAGAGAAGTTAGTTTCTTAAATAAATTTATGTAATTTGAAGGATTTGTCTTAAAAGATAATATTCTTATTCTTAAAAGATAAATCAAAGTAGACAA
GCAGTCAAAACTTACTAGTTTCTACAAATTCTCGTGATGGAAAAATATTATTTTTTTGCTAGGACTCTGTGATCTCTATATGCTTAAAGAATAAATTTAA
CGTCTTCCATTCAGCCTCGTGAGTAATTTTATATATTAATAAATGTATTGCTTATTTTTTTTTGGTTAAAATTGTAAATGTATTACTTTGAAATACTTAC
ATATGTTTTTTGACTAGTTGTATAGCTTATCTATTGATCTGACATATTTTCTAGCTGTCAATGCTATTTTAGGGGTGCAATATTGCAATCACACATATTG
AAACTTGAGTGTGTTTGTTTGTTTACTAAATGTGGTATTTGTTATGAACTGGCATGCTTAGACAATCTCCAATACATGATTTTTCTTTAGGTTCTTAAGT
GGATTCCATTTGTACACATAGTGCATGATAACAACAGTTTTCTGCTTTCTTTAGTGTCATAGTGCCTTGGTAGTTAACTGAACATGAAATGTAATGCAGA
TGGGAATGGCAGGTGCTTTGGAAACTCAATGTGGCCAATCATTTGGAACCGAGCAGTTTCATAAGCTTGGAAACTATGTCTTCTGTGCAATACTCTTTCT
CATTTTAAGCAGTGCCCCAATATCTATCCTATGGATTTTCATGGATAAACTGCTTGTTCTTTTGGGGCAAGATCATGCTATTTCACTAGTAGCTGGAAAC
TACTGCATTTGGCTCATTCCTACACTCTTTGGTTATTCAAGGTTTGGTTCGTTATTTTCAGACTCAGAGCTTGATCTTTCCAATGCTTGTAACCTCAGTT
GTTGTTTTAGTTTTGCATATACCTATTTGTTGGGTACTAGTGTTTGAACTGGGACTTGGACAAAATGAAGCGGCCTTATCCATTGGAATTTCGTATTGGC
TCAGTGTCATGTTACTTATAGTTTACACAGTATTATCCGTCATGTCAGAAAACTAAGGTAGCTTTAGGTAGCAATGCTTTAAGAAGCATCAAAGAGTTTT
TCTTCTTAGCCATCCCATCTGCACTTATGATTTGGTAAGTTTCTAGTTTTGCCCCTATATGATTGGAGAAATTATTACATGGTTTAGGACTACCATGGTC
CAAGACCACCATTGATTTCAACTATTTTTCCATGTGACATGTAACCGAGTTTAATTAACTCATTTTCGTTAATTGAAAATCTCAATTATATATCATGTAA
AGATTGTTCGAGACCATGGATATGTGGTGGTTCCAAACCATGTAACAATTTCTTGTATAATTGGGGAGGCCAATGACTCGATGCTGCTTCTTCTCTTTAC
TTTTTGTTTGTAACATGTTTCTATCTTCTTCTTTTTTTTTAAAAATAACATGTTTCTATCTTAATGGATACATTCAGTTGAGTGGTGGTCGGTCATTAGA
GCTACTAGTAATATTTGCTGGACTTTTACCAAATCCCAAACTTGAAACTTCAGTTCTATCAATCTGGTGTGTTTCTCCATGA

>Glyma.02G089900 | Chr02:7903639..7908241 reverse
GGAAAGAAAATGCCCACGTTGTACACGAAACATCAGTCTCACTAGTGTTATCTTACCACCTTTCTTCACCTTCTCGTATATCCTTGATTTGGCTTTAACC
ACAGACACACGTGGGAACTCCGAAAGAAAGAAAGAAAGAAATGGAGGAGACATTGTTGCCAAAAGAAAACAAGAGGGTGACCTTAACAAACTCAAAGAGT
AGTAGTGGCTTTGTCCAAGAGTTGAAGAATGTGAGTTTGATGGCAGCACCAATGGTGGTGGTCTCAGTGTCACAGTTTTTGCTGCAAGTGGTGTCATTGA
TGATGGCTGGTCATCTTGGTGAACTCTCCCTTGCAGGGGTTGCATTGGCTACTTCCTTTGCTGATGTCACTGGCTTTAGCATACTTGTATGTTCCCTCCT
TTCCCTTCTTATTTTCACATACAACTTACAGACCATTCAAATGCATTTTCCTTCTCTTTTAGCAAATCAACTTTTAGCCATTTTTTTGGTACAGGATATC
AACAGATTTACTATAACTAATCTCTGTAAAAAAAATATTTTTTTTAATTATGTTTTTTTCATGATTCAAATAAATAGACTAAGAAAAGAAAATAAATTAC
ATTTTTTATAGGCTAAAATTAATTTTTATATTATTTAATGTTTATAGAAATTTTTTCCTTTAACTTTTCTAAAAACTGAAGGTATAAACCGATTTTTTTT
AGTTCATGAAAGAATTTTTTTTTACAAATACTTAATAAAATATTTACCTAAAAATAGTATAAGCCAAGCTCACTTATGAAACATCATCTACGTAGTCTTA
AATTATCATCTAAGTAGTTGAGAAAACTCGGTCTCTTAAATAAATTTATGTAATTTGAAGGATTAGTGTTTGACTATCTGCCGAATAATATTCTTAAATC
AAAGTAGACAAGCAGTCAAAACTCACTAGTTTCCACTAATTCATGTGATGGATAAAGATTATTTTTTTGCTCGGACTCTGTGATCTATATATATGCTGAA
AGAATAAATTAACATCAGCCTCGTGCGTAATTTTATATATTTATAATTTTTTTTGACGAAATACTTTCTTTTATTAAAATTATAAATTTATTACTTTGAA
ATAGTTATATGTTTTTTTACTAGCTGTATAGCTTATATCTTGATCCGACATATTTTCTAGCTGTCAATGCAATACTGCAATCACACATATTGGAACTTGA
GTGTGTTTGTTTGCTTACGATTTGTGGTATTTGGTATGAACTGGGATGCTTAGACAAGTTAGACCATCTCCAATACTGCCTTGGTATTAGTTGATTGAAC
ATGAAATGTAATGCAGATGGGAATGGCTGGTGCTTTGGAAACTCAATGTGGCCAATCATTTGGAGCAGAGCAATTTCATAAGCTTGGAAACTATGTCTTC
TGTGCAATACTCTCTCTCATTTTAAGTAGTGTCCCAATATCTATCATATGGATTTTCATGGATAAACTGCTTATTCTTTTGGGGCAAGACCATGCTATTT
CACTAATAGCAGGAAACTACTGCATTTGGCTCATTCCTGCACTCTTTGGTTATGCTGTTCTTCAAGCTTTGGTTCGTTATTTTCAGACTCAGAGCTTGAT
CTTTCCAATGTTGGTAACCTCAGTTGTTGTTTTAGTTTTGCACATACCTATTTGTTGGGTACTAGTGTTTGGACTGGGACTTGGACAAAATGGAGCAGCA
ATATCCATTGGAATTTCATATTGGCTCAGTGTCATGTTACTTTTAATTTACACAAAGTATTATCCATCATGTCAGAAAACTAAGATAGCCTTAGGTAGCA
ATGCTTTAAGAAGCATCAAGGAGTTCTTCTTCTTAGCCATCCCATCTGCACTTATGATTTGGTAAGTTTCTAGTTTTACACCTGTATGATTAGAGAAATT
ATTACATGGTTTAGGACTACCATGGTCCAAGACCACCATATGACATGCAACCGAGTTTTATTAACTCTTTTTTGTTAATTGAAAATCTTGATTATATATC
ATGTAAAGATTGTCGAGGCCATGAACATGTGGTGGTTCCAAAGTCCAACCCATGTAATAATTTCTTGTATGATTGGGGAGGTCAGTGACTCAATGCTGCT
TCTCTTTACTTTTTGTTTGTAACATTTTTCTATCTTAATGGATACATACAGTTTTGAGTGGTGGTCATTTGAGCTGGTAGTAATACTTGCTGGACTTTTA
CCAAATCCCAAACTTGAAACTTCCGTTCTATCAATCTGGTGTGTTTCTCCGTGATCCAGTTATGTAAATATTTAAAAGTTCATTACCTGATAACTCAGGG
ATTCATCTGGCTTATGTTTTCCTTGAGCAGCCTTAACATTTGCACCTTGCACTATTTCATTCCATATGGAACCGGTGCTGCAGTAAGGTAAGCTCTATAT
ACACACATTGATGTATTATGTGGGGCTTATGTTGTCGTATGAGGAGTTAATTTTGATCATATCGCTGTATTGGATGGTCCAGATTAGATTTAAGAAAAAC
AATCTTAACCAAGCATATAATATTATATGGTCAGGATTAAAGTTTTTCATGTTCTCACTAAATACTCCTCATGTTATGTATCTTACTTTTGTGTTTCTTA
TCTCACCAGGTATCGGTCCAACAACCAACCATAAGTTAGATTTAAAATAAATTTTAAGAGAAATATTTTAATGATAAAATTATATATTAGACGTTTTTAT
CAACATATATTTCATTTTTTTAATAAATGTGACATTTTTGCTTTATAAAATTAAAAGAAAAAATATTTTTATTGGTGAGCCTAAAGTTTATACTTTAGTT
GTTTTACCCGTGAAAAAATCAACCACATTACAAAGCATACTTCTATAATTTTGTAGTACCCGGGTTTCGAATGAATTAGGGGCACGAAGACCACAGGCAG
CGCGAGAGGCTGTTTTTGCTGTAATTGTTCTAGCATTCACGGATGCAGTTGTCTTTAGCTCTGTTCTCTTTTGCTTCCGTCATGTGTTGGGATTTGCATT
TAGCAATGAGATGGAAGTTGTACATTATGTTGCAAAGATAGTTCCTGTGCTTTGTCTCTCTTTCATGGTGGATGGCTTTCTAGGAGTTTTGTGTGGTAAG
CTTTCCACATTGAACGTTATAAATGCCCTATTTGAATGAACTTCTCATTAAGAACTTATAGGATAAAAAAAATTAAAAAGTAAAACAGGTTGAGTTTCTT
TTATAAGTTAAAATCAACTTATACTTCTCACCTTTTGAAGAGATATATTAAAAGAGAGTTTCTATCATAGTTAAGTGCATAAATTGATTTAACTTATGAA
AAAAGCTAAACTCATTTCTTCTTCTTCTTCTTCGAAAATACATTTTTAAAAGGGAATGACAATCACTTCACTCAATGATAACAACAGCTCTATGGTGAAA
GCTAGTAGATATGGTGGCAAATAAACATTAGGTTTGTGGCTCTCAGTTGTGTGTTCAATGAATCAGGAATTGTCAGAGGCAGTGGATGGCAGAAGATAGG
GGCAATTACCAATCTTGTAGCATATTATGCCGTGGGAATCCCTGTGTCTCTCTTGTTTGGTTTTGGTCTTAACTTCAATGGTAAAGGCCTTTGGATTGGA
ATATTAACCGGATCTACACTACAAACAATAATACTGGCTTTGCTGACAGCATTTACAAACTGGGAAAAACAGGTTCTTAATTCTGTGCTTTACCTGTTGT
TTTATGTTCTGACTTACTCAAAAAATCAGTAAATAATAATAAGAATTAACATAGAAAAATAATGAATTGATGGAATGAAATGATATCAATTGTCCTTAAT
TTTTTGTTTTGTTTGAAATGAGATGATCATGAAATGAAATGGAATTCAAACTTATTGGAATGCATGTCACTAATACCCCTTATTTTCTTCTCCTGTTTTG
GAGGTATGAGATGAAATAGAATGAAACTAACTTATTGTTAACTAGTTTCTTTACCATTCGGTATATTTCCTAAATAATGAAAAAAAAAATTATTTCATTT
CATCATAAAATATTTTTAAACAACATTATTATAATTTTGTTATATTTCATTTCCTAAGTACACTACATCCAATGATCCATTGCATTCCATTTTGTACTAC
CAATAGACATCAAAACATAGTCTACATTAACTTCTAGGTTTAATGTACAGTTGCAGTATCCTTTATTTTTTCTGCTTCATGGATATTTAGGGAAGATAGC
AATAAATCTTATGGAAAGTGTGAGCAGTTCTTATGTTACTAGAAGATTTACAAAGTTTGTTAAACTTGCTATAATTTGCAGGCATCTTTGGCAATTGAGA
GATTATCCGAGCCTGATGAGACTGTTTTTTAAGGTCATAACGGGTTAGTTTGCGCTTGAAAATGAAGGAAGAACAAAGGTTTTGTAACCCTGTTAGTGAA
ATATTTACTGCTTATGAAACCTTGTTTATTTTATTTTGTTATGCGTAAAAATTAAAAACAGATACAACAATTCACGTATTCCTGTTGAAGTAATTAAGCT
AGACTCTTTATTGAAACCTTATTTATTAATTGTAGCTTTTCATTTTCATCGGTGTTGTATGTTCGACGCATGCAAAAAAGTGCACCCAGTTAAAATATGA
TAA

>Glyma.02G090000 | Chr02:7919496..7922905 reverse
TGTTCTATATAACTCGTGTTGTTGATCATCTTGTTGTCTAACATCTGTTTCATGAACGTTGCTTTGCATAGAAAAACTGAGAGAGATGATGGGTAAGGAG
GAAGCAACACCGCTTCTAAGAAAGAGTGAGGTGGCACCATTAGAGGATGATGATGCATTTTGTGTAGAGCTTAAAAGGGTAGGCTCCATGGCAGCTCCAA
TGGTGGCTGCGAATATGTGTCAGTACCTTCTACAAGTTGTGTCACTGATGATGGTGGGACATCTTGGTGTACTTGTCTCCTTCTCTGGGGTTGCAATTGC
CACTTCTTTTGCTGAAGTTACAGGCTTTTGTGTTCTTGTAAAGTCTTCTTTCATCTCCTCACTTTTTTTGTTCCATGCACCTGCCTGTATTTGCTTTAGT
CCACTTTCTGGTTCTTTAATTAGGAAACTTTCTCTGATTGATTCGGTTATTTAGTTAGTACATAGCACTAACATTCCTGAAGCCAAATATGACTTGCGAA
AATTATGATAAAAGACCGTATATTTTGATCGTTAGTTGGTTCACTTTGATTTATCTATTAATGTTGTTAGTTAGTATGCATAAAGTTTCACTTGGAATTA
GTACATGGACACTTCATGAAAGCCTTTCAAAGCATATTAAAAGAAAATTTTGATACTTCTTAGTTAACAGGTGCAGGTGATCATGTGATTACTGAGATGA
TCTAGGATATTGTTTTCCTTGTTTTGAAGATAGGATCCTTTATTCTTCTTCTTCTTTTTTTTTTTTTGAAACAAGTAAATAGTCTATGTGTGTAAAGGAT
TTTTAAATTGTGATTCAATCACAAGCCGCCATGGTAAATTTGTTAACTTTACAATAAATATCTTAAAAGTCAATCCATAGGATTGAGACACTGGTGTAAG
GTTCCAGTGCTGTATTCATGAAACGATCTATTTAGCAAAAGAACCAATGTTCAATTCAATTCCTTCCGTGTGTAAAATTTCCTTAAGCCAACAGTAACCA
TGCACTAAAGGTGTGATTAATCTTTGGCTCAGAAGGTTAGGGGACATCCCGCCATCTATGATCTAGCACAAAAAAAAAATATTTTTGTTTGGTTTAGCTG
ATGAAACTTCCTCTTAGTCCAATGTCAAACTTTTGATACAGGATATGCACTAAAGAGTTGAATTGAAAGTACCTTTTATTTAAATTTTTAGTTTAGCTTA
AATCACTTTAAATTTGCTTGTACAGTTCTAAATTAATCTCCACCCTGAATTCTTGTTGACTCTGAGTAGATTGCTAAATGGCTTGCAGATGGGAATGGCT
GGTGCATTGGAAACTTTATGTGGCCAAACCTATGGTGCAGAAGAATTTTCAGAGATTGGAAACTACACTTTTTGTGCAATTGTGACTTTGCTTTTGGTTT
GTCTCCCCATATCTATGCTGTGGATATTCGTGGATAAAATACTATTGTTGTTTGGTCAAGACCCTGAAATTTCTCATGTAGCTCATGAGTACTGCATATA
CTCCATCCCTGCATTGTATGGCTTTGCTGTTCTTCAATGTCAGATTCGCTACTTCCAGACTCAGAGTATGATCTTTCCCATGGTTTTCAGCTCAATTGCG
GTTCTGTGTTTGCATGTTCCTATTTGTTGGGCTTTGGTATTCAAATTAGCCCTAGGACATGTTGGAGCAGCATATGCTATTGGAATTTCATATTGGTTAA
ATGTCATTGGGCTTGGAATTTATATGAATTTTTCTCCAGCATGTGAGAAAACCAAAATTGTGTTTTCTTTTAATGCTTTATTAAGCATTCCAGAGTTCTG
CCAATTTGCTATTCCTTCTGGACTGATGTTTTGGTAATGTATTATGACATTGCACAATTGTTTTTTCAGTTTAGTCATCTCCTAATTGATTTTCTTAATT
GTTTCATTGTTCATAGTTTTGAAATGTGGTCGTTTGAGCTACTTACATTATTTGCTGGGCTTTTACCTAATCCACAACTTCAAACCTCAGTTCTTTCTGT
CTGGTGAGAGGATTAATTTTTATTAATTATTGAAGAGTAGGGCTGACAACACACTTTATACTATTTGATTAAAATTTATTAGAAATTATAAAATCATGAA
TGAAAGCTCATTACATAAGTAGTGGGACCTATATAAATTTATGATTTTTAATAAATTTTGACCAATAATAAAGACTATGTTTAAAATAATGTTAGAAAGT
ATGTTGCTAGCATTTCTCATTATTGAAATAATTTGTGAGGGGTTTTTCTAACATGTTTCAGACTTTTTCAGCCTTAACACAACCACATTGCACTACATCA
TTCCATATGCTGTTGGAGCTTCTGCAAGGTTTGGTCCAAATTTGTTTCTTCATTTCTATACACAGGATCAATGGCACTGTAGAATTGTCTACTGATAAAT
CATGATCTTATGAATTTCGCTTTGCAGTACTCGCATTTCGAATGAATTAGGAGCAGGGAATCCAAAGGCAGCTCAAGGTATTGTTCGTGTTATTGTGATT
ATTGGAATTGTTGATGGGGTTATTGTCAGCATTTTCTTCGTTTGTTGTAGGCATATATTAGGATATGCTTATAGCAACGACAAGGAAGTTGTAGATTATG
TTTCAGATATTGTTCCCATTCTTTGTGGGTCTTTTACTGCAGATAGTCTAATAGGAGCTCTTTCTGGTGAGTTTTTATTTGCTGAAATAGTCTATGTTAT
TCTAGCATCATTAATTGAGCACAAATATTAGCAATAAAATGGATATTCATATGTGCAGGGATTGCAAGAGGTGGAGGATTTCAGCAAATAGGAGCTTATG
TGAACCTTGGAGCCTATTATCTTGTGGGAGTTCCTTTAGCATTCTTATTGGGTTTTGTCCTACATTTCAATGCCAAGGGACTATGGATGGGAAGTCTTAC
AGGATCTGTTCTACAAGTGATTATTCTGACTGTTGTAACAGTGTTAACAGATTGGCAGAAAGAGGTTGGTTTTTACTTTTTTAACAGTGTAACAGTTTTA
ACTTCTCCATTGTTAATAATTGCACGTGTCATTTGTTTTTTTGCTTTCCTTCTAGTTTTGAGCCCTTTTTGTTGCCTTTGCTTGATGAATCCAATGGATA
ACAATTGACTGTTAATTAAAGTGGCTCTAAATGATTGTATTTTGTGGTTACTGCAGGCAACAAAAGCAAGGGTGAGAATAGTTGAGAAGTCAATTAAAGC
TCATAATGGTTCAGTCTGAAATTTTAAATTTTGCAATTATAGTAGAAGAATAAAAGAGTGGAAGAAAAAGGAAGTAATGACTAATTTTCTGTCAGATTCT
GGTTTTGCTATTATTTCTATTTTGGTGAAGTAAACAGATAAAAGTTATTATGTCTATGTATAATGTTGTGGAATGCAAAGTGGTGGGAGCCTCTATGGTT
TTGCAGATTC

>Glyma.18G293200 | Chr18:57131714..57135856 forward
ACCATTGTTAAGAAAGAGTGAGGTGGCACCATTAGAGGATGATGATGCATTTTGTGTAGAGCTTAAAAGGGTAGGCTCCATGGCAGCTCCAATGCTGGCT
GCGAATATGTGTCAGTACCTTCTACAAGTTGTGTCACTGATGATGGTGGGACATCTTGGGCTTCTTGTCTCATTCTCTGGGGTTGCAATTGCCATCTCTT
TTGCTGAAGTTACCGGCTTTTGTGTTCTTGTAAAGTCTTCTTTGATACTTTTTTTTTCTTCCATGCACCTACCTATATTTGCTTTAGTCCATTCGCCATA
TTTTTCTAATTTATACTTGTACATAGTATCTTTTCTAAAATAGTGTTTGTGAAGATTCAAGAGTGATTCAGATTAAGCTTCTTTTTCTCTCTTTTATCTT
TTCAAAACTATCTCTGATTCATTCAGTTATTCAGTTAGTACATGGTACTAAAATACCTGTAGCCAGGTCACTCGCCAAAAGTATAATAAAAGACCATATA
TATATATATATATATATATATTGATCATTAGTTGATTCACTTTGATTTATCTAATTTTTTTGTTAATTAATATGAATAAAGTTTCACTTGGAAAGCCATT
TAGTACATTGGCACCTCATCAAAGCCTTTCGAAGCAGATTAAAAGAAAATAAATGGTACTTCTTAGTTAGCAGGTGCGGGTGATGATGTGATTAGTGAAA
TGATCTAGGAGATTGTCTTCTTTGTTTTGAAGATAGGATCCTTTTTATTTATTTATTTTGAAACAAGTAAATAGTGAATTTGTGTAAAGGATTTTTATAC
TCGTTGAATCACAAATTGACATGTTAAATTTTTCACACTAGTGCATAGAAATTTAATTCTTTCAAAATATACATTGTTGTTTGACTTAGCTGATAAAACT
TTCTCTTAGTCCAATGTCAAACTTTTGATAAAGGATCTGCAAGAAAGAGTTGAGTTGAAATTCGCCCTTTATTCAAGTTTCTAGTTTAGCCTAAGTCACT
TTAAATTTTCTGGTACAGTTCTAAATTAATCTCCACCCTGAATTCTTGATGACTCTGACATGATTGCTAAATGGCTTGCAGATGGGAATGGCTGGTGCAT
TGGAAACTTTATGTGGCCAAACCTATGGTGCAGAAGAATTTACAGAGATTGGAAACTACACTTTTTGTGCAATTGTCACTTTGCTTTTGGTTTGTCTCCC
CATATCTCTGCTGTGGATATTCATGGATAAAATACTATTGCTGTTTGGTCAAGACCCTGAAATTTCTCATGTAGCTCACAAGTACTGCATATGCTCCATC
CCTGCACTGTATGGATTTGCTGTTCTTCAATGTCAGATCCGCTATTTCCAGACTCAGAGTATGATCTTTCCCATGGTTTTCAGCTCAATTGCGGTTCTGT
GTTTGCATGTTCCTATTTGTTGGGGTCTGGTATTCAAATTGGGGCTGGGACATGTTGGAGCAGCATATGCTATTGGAATTTCATATTGGTTAAATGTCAT
TGGGCTTGGAATTTATATGAATTATTCTCCAGCATGTGAGAAGACCAAAATTGTGTTTTCATTTAATGCTTTACTAAGCATTCCAGAGTTCTGCCAATTT
GCTATTCCTTCTGGGCTGATGTTTTGGTAATGTATTATGACATTGCACAATGGTTTTTTCAGTTTAGTCATCTCCTATTTGATTTTCTTAATTGTTTCAT
TGTTCATAGTTTTGAAATGTGGTCATTTGAGCTACTTACATTATTTGCTGGGCTTTTACCTAATCCACAACTTCAAACCTCAGTTCTTTCTGTCTGGTGA
GAGGAATAATTTTTATTAATTATTGAAGAATAGGGCTGACAACACACTATCTAACACACTTTATACTATTTGATTAAAATTTATTAGAGATTATAAAATC
ATGAATGAAAGCTCATTACATAAGTAGTGGTACCTATATAAATTTATGATTTCTAATAAATTTTGACTAATAACAAAGAGTGTGTTTAAAATAGTGTTAG
AAAGTGTGTTAATAGCATTTTTCATCATTGAAATAATTCGTGAAGTGTTTTTCTAACATATTTTTAACTTTTTCAGCCTTAACACAACAACATTGCACTA
CATCATTCCATATGCTGTTGGAGCTTCTGCAAGGTTAGGTCCAAATTTGTTTCTTCATTTCTATACACAAGATCAATGGCACTGCACAATTGTCTGCTGA
CAAATCACGATCTTATGAATTTTACTTTGCAGTACTCGCATTTCGAATGAATTAGGAGCAGGGAATCCAAAGGCAGCTCAAGGTATTGTTCGTGTTGTTG
TGATTCTTGGAATTGTCGATGGGGTTATTGTCAGCACTTTCTTCGTTTGTTGTAGGCATATATTAGGATATGCTTATAGCAATGACAAGGAAGTTGTAGA
TTATGTTTCAGACATTGTTCCCATTCTTTGTGGATCTTTTACTGCAGATAGTCTAATAGGAGCTCTTTCCGGTGAGCTTTATGTACTGAAATAGTCTATG
TTCTTCTAGCATGCTTAATTGAGGACAAATAGTAGCAATAAAATGGATATTTGTATGTGCAGGGATTGCAAGAGGTGGTGGATTTCAGCAAATAGGGGCT
TATGTGAACCTTGGAGCCTATTATCTTGTGGGAGTTCCTTTGGCATTCTTATTGGGTTTTGTTCTACATTTCAATGCCAAGGGACTCTGGATGGGAAGTC
TTACAGGGTCTGTTCTACAAGTGATTATTCTGACTGTTGTAACAGTGTTAACAGATTGGCAGAAAGAGGTTGGTTTATACTTTCTTATGTGACTTCTCCA
TTGCTAATAATTGCTTGAGTCATTTGTTCTTTTTCTTTTCCAACTAGTTTTTGAGCCCTTTTCGTTGCCTTTGCTTGATGAATCCAATGGATGTCTGTTA
ATTAAAGTGGCTCTAAATGATTATCTTTTGTGGTTACTGCAGGCAACGAAAGCAAGGGAGAGAATAGTTGAGAAGTCAATTAAAGTTCATAATGATTCAG
TGATATGATCCAAGGATCCAATCACTATAATAACTTGGGAAGCCTAAAACATGGGATGTTTAGAAGGAAGTCTAGCCTATAAACAATAGGAACTTTGTAG
CTTTGAGGCATATTGGAGTCTGAATGAAAGTTTGAGTTTTCTACCTTTTGTGAGAGCTTCCCTTGGTTCTAGGCAAAGAACTAAATTTGACCTTATCAAG
GTAACTTGTGGCGATCAAATCTATGACTTATCACCCATCTTCTCCCCTATTTCTTGAGTGCGGCGTCAATATCCTTTTCTAAAAGAAAACTAAATCAATT
TCTTAGTATGAAAACTTCAACCCGATCCGTCTCGTTTCCGTGTTCATATCATTCAGTGAGAAATTTTTTAAATATAGTAGAAGAATGAAAGAGTGGAAAA
AAAAGGAAGTACTGACTAATTTTCTGTTAGATTCTGGTTTTGCTATTGTTTCTATTTTTTGGTGAAGTAAACAGATAAAAGTTATTATGTCTGTATATAT
GTGATTTAAGAACCGGAATTGTGCCTATGAAGTGTAAGGGTAGCACATAAGTCAGAACTTAGAAAATTATTCATGACATGCTGCAGTTACATGGAGGTGG
ATCCGGGCTTCCATTTTAGTATCTCTTTCATGTTCTGTGTCGAGGCAAGGAATTGATCTTCAGATTGAGGGTGTGTTTTGTTTGGAGTGTGTTAACAAGG
GGCTATTTCTATAAGGTTGGTCCTTCATTGCGGTACAGGGTTGGTGATAACGTGGATTTTTTATTGTTGTTGGATGAAAAGTCGACATGGATCTTTATAT
TGTTGTGGGATGAAAAGTGGTGGGAGCCTCTACGGTTTTGCAGATTCAATGGAATTTAATAGTATCGAGGTGGAAGATGTATGTTTTATAGTCTTGAGTT
AATTACAACCAAATAAGCTTTGGAAGTAATGGTAACTATAATGGTAATTACGTTGGGTGTGACATATAGTTGTTTTATGTTTGTTAGGGGGGTGGATGAA
CTATTGTTTTGCATTGCTTTTTTTTTTTTTTAGCTTTCGTTATTACAAATCTTTGTACTGTTGTTGACATATTTGGAGTGATCACATCTTGTGGTGTATC
CAAATCTGTTTTATTTTAATACAAGTTTCTTTGGCTTTTTTTC

>Glyma.05G001700 | Chr05:130310..133384 forward
CTGGGTTGACCACAAAATAATTGTTTGGTAAAAAGAGGTAGAAGTAGAAGAAAGAAGCACCACTTTCTCTTTCTCTTCCTATATATTCCTCTCTTCACAA
CACACATTGTGGCATGAACATTGACCAGTGTTGACAATATGGAAAGCTCCAGAGAGTTGGCAGCACCACTGCTAGTGCTGAGAAAGAGTGGCGAGCAAGA
GAATAATAATGGGGTGGAGAGTACATTTTGCCAAGAGTTCAAGAGGGTAAGCTCCATGGCGGCTCCAATGGTGGCTGTCACGGTGTCGCAGTACCTTCTA
CAAGTTGTGTCTTTGATGATGGTGGGACATCTTGGGATACTGGTCTCCTTCTCTGGAGTTGCTATTGCTACCTCTTTTGCTGAAGTCACCGGATTTAGTG
TCCTTGTAAATATATACAACTCCTCCTTTTTCTCTCTTTTTCATTCTATATATGCTATTGCTACCCTTTCTTGTTTTTCTCAGTTTTCATTGAACTTTAA
AAAGGTGCCATTTAGCTTTTGCTTGCATTCAACTATAGCATCTTTTAAACATATAAGCTTGTATTCTATTGGAAAGCTATTTTCTGATTCTTGGGCAGAG
GGCTCTTGATCCAGTTAAGGTTATCATTTATGTTGATTCTTAATAAATTTACCAGTTAACTAATTAATAAGCCATATTTTGGGCTGGATTGGATTTTAAT
ATAGAAAATAAATAGCATTCCATTGGTTATTTGTGTTTGATACAGTTCAGTTTGGTTGATAACCCTTCTTTTGAAACTGGATCTGTACAAACAGTTGAAA
AGGTTGAGAACTTAAAAAATTGCCCTCATTCTACATAACAATATTTTGGCTAGTTTAGCCATTAATATTAGTCTTTGTGTGCATATATTTATATATTATT
AATTAATGCTACCCTACCCTGTTGAAATGCCAAAGTCAATCTGATTGACTGATGAGTACTGTGTTGCAGTTGGGAATGTCCGGTGCATTGGAAACTTTAT
GTGGGCAAACCTATGGTGCGGAGGAGTATAGGAAGTTTGGAAACTACATTTGGTGTGCGATTGTGACTCTGACATTGGTTTGTCTCCCGATATCTCTGGT
GTGGATATTCACTGATAAAATACTCATGCTGTTTAGTCAAGACCCTGAAATTTCTCATGCAGCTCGTGAGTACTGCATATACCTTATTCCAGCTCTATTT
GGCCATGCTGTTCTTCAAGCTCTCACTCGCTACTTCCAGACTCAGAGTATGATCTTTCCCATGGTTTTCAGCTCAATCACCGCACTGTGTTTGCATGTTC
CTATTTGTTGGGGTCTGGTTTTTAAGTTGGGACTGAGACACATTGGAGCTGCATTAGCCATTGGAGTCTCGTATTGGTTGAATGTGGTTTGGCTTGCAAT
TTATATGATCTTCTCTCCGGCTTGTCAAAAGACCAAGATTGTCTTTTCTAGTAACGCTTTACTTAGCATTCCAGAGTTCTTGAAATTAGCTATCCCTTCC
GGACTCATGTTTTGGTAAATTTGTTTATTTCTTATATAACATTTTTAATTCTCCTAATTAATTAATTAATTACATTTTCACATTAATTTCTATGTATGTA
TATAGTTTTGAATGGTGGTCCTTTGAGGTGCTTACATTACTTGCGGGGATTTTGCCTAATCCTCAACTCGAAACCGCAGTTCTGTCAGTCTGGTGTGTGG
ATTAAATTTCTTTTCCATTATTATTACTCAAAGGCAAATGTTAACCGGTGCTCTAAAGGCATGAGTTAATAGTGCGCTAAGGATATTGGTTAAGAAATTA
AAATAAAAATATTTTTATTGAGAGATGAAAAATTTCACTGTTCATGATCTTTTTCACACTCTTCATTTAGTTCTTTAACCAATGTCCCTAAGAATACCCT
TGCTGAAATAACTCGTGATCTTCTAATTAACTTTTTGATCATTCATACGCTTCTTGCAGCCTTAACACAACCACATTGCACTACTTTATTCCTTATGCTG
TTGGAGCCTCTGCAAGGTAAAAAAGGTTTGGTCCAAATTTGCCTTTTTTTGGGGCTTCCAACACACTTAATCTGATGATGGATGTTTGCAGCACTCGGGT
TTCAAATGAACTGGGGGCAGGGAATCCAAAGACAGCAAAAGGTGCAGTTCGAGTGGTTGTGATTCTCGGGGTGGCAGAGGCAGTTATTGTGAGCAGTGTC
TTCATTAGTTGCCGACATGTATTAGGATACGCGTACAGCAATGATAAGGAGGTAATCGATTACGTTGCAGAGATGGCTCCACTCTTGTGTGTGTCTGTTA
CTGCCGATAGTCTAATTGGAGCACTATCAGGGATTGCGAGAGGGGGAGGATTCCAGGAAATAGGGGCCTATGTGAACCTGGGAGCCTATTATCTTGTGGG
AATTCCAATGGGTTTGTTATTGGGTTTTCATCTGCAACTAAGAGCCAAGGGCCTTTGGATGGGAACTCTATCAGGCTCTCTTACACAAGTCATTATTCTT
GCTATTGTAACAGCACTAACAGATTGGCACAAAGAGGTTCGTTCATTCCATACCTTACATTACGTAACCTGTACAAATATCACAAATGCTAACCCGTGTT
CTTAGAGTATTAGTTAAAGAACTAAAAATGAAAAATATTTATTATGTGGATCATAGGAGAGCCTAAAAAAATCATTGAAAACATAATATTCTTAAGACAC
TAGCTAGTTATTATTTGCCACAAATTCAATTAAATGACTGTATTTTGTGGTTGCTGCAGGCAACTAAAGCGAGGGAGAGAGTAGTTGAGAACTCAATTAA
AGTTCACTATGAAGCTTAAGAAAACTGAAAAATAAATGATGTTTTGGTTTCTGCATGGACTGTCATCTGCGGTGGAGTGGAAAGATAGAGTCATATAGAG
GATAGTGAGTGTGGCCCAGTTGTATCTGTTTTTTTGGTGAAGAGATTTAATTATGTTAACATACACAACTTGTTGGTGTGAATATGTGATGGGATTTTTG
TACTGGATTTGTGCCTCTGGCGTCTGATAAATCAGATGCCACAAGTTTAATGAATATGCTTCAGCCTTCAGTTAC

>Glyma.19G001600 | Chr19:117544..120775 forward
AGCTGGTTTGACCACAAAATAATGGTTTGGTAAAAAGAGGTAGAAGTAGAAGAAAGAAGCTCCACTTTCTCTTTCTCTTCCTATATATTCCTCTCTTCAC
AGCACATCACAGCACATTGACCAGTGTTGAGAATATATGGAAAGCTCTAGAGAGATGAATAAGGAGTTGGCAGCACCACTGCTAGTGCCAAGAAAGAGTG
GTGATGGGCAAGAGAATAATAATAATAATGGAGTGGAGGTGGTGGCATCATCATCAGAGAGTACATTTTGCCAAGAGTTGAAGAGAGTAAGCTCCATGGC
GGCTCCAATGGTGGCAGTCACGGTGTCTCAGTACCTTCTACAAGTTGTTTCTTTGATGATGGTGGGACATTTTGGGATACTGGTCTCCTTCTCTGGGGTT
GCTATTGCTACCTCTTTTGCTGAAGTCACCGGATTCAGCGTCCTTGTAAATATATACAACTAACTCCTCTTTATGATTCTATATGTGCACTACCCTCTGT
TGTTTTTCTCATTTTTAGTTCAACTTTTCGATTCAACTATAGCATCTTTTAAACATATATATTATAAGCTTGTATTCTATTTTATTGGAAAGCTAGCTAT
TTTCTGATTCTTGGGTGAGGGCTCTTGATACAGTTAATTAGTACCTAGTATTAGCATACTACTAACCGGATCACATGTAACAGTACAACGGGTTAACATT
TAGGTTGATTCTTAATTGATTTATCATCTACATCCAAAAAGCCTTATTCTTTGTAAGAAGCTTCTACAGTTTGGAAAGAAGTTTTGATTCTTTAAAAAAG
AAGAATCTACTTCAACCGATATGCCCTAGGCACGGCATTAACAAGTTAGCCAGATTTTGGGTTGGTGGGGTGGCATGGATTGGATTTTAATATAGAAAAT
AGCATTCTATTGGTTATTTATTTGTGTTTGATACAGTTCAGTTTGGTTGATAAGCCTTCTTTTGAAACTGGATCTGTACAAACAGTTGAAAGTTGAGAAG
TTAAAAAATTGCCCTCATTCAACCCATATCGAATGTTAGTTTTTAGTTTTTGTTAACAGGAAGATTCGATTTCCTTCATCCTTTATCAACAAGCCTACCT
TATATCTTCCGCGTGTCTGTGTGTTTGTATATATCCTTTTGAAATGCCTAGTGAATCTGAGTACTGTGGTTGCAGTTGGGAATGTCCGGTGCATTGGAAA
CTTTATGTGGGCAAACCTATGGTGCGGAAGAGTATAGGAAGTTTGGAAACTACACTTGGTGTGCGATTGTGACTCTGACATTGGTTTGTCTCCCGATATC
TCTGGTGTGGATATTCACTGATAAAATACTGTTGCTGTTTAGTCAAGACCCTGAGATTTCTCATGCAGCTCGTGAGTACTGCATATACCTCATTCCAGCT
CTATTTGGTCATGCTGTTCTTCAAGCTCTCACTCGCTACTTCCAGACTCAGAGTATGATCTTTCCCATGGTTTTCAGCTCAATCACCGCACTGTGTTTGC
ATGTTCCTATTTGTTGGGGTCTGGTTTTTAAGTTGGGACTGGGACACGTTGGAGCTGCATTAGCCATTGGAGTCTCGTATTGGTTGAATGTGGTTTGGCT
TGCAATTTATATGATCTACTCTCCGGCTTGTCAAAAAACCAAGATTGTCTTTTCTAGTAATGCTTTACTTAGCATTCCAGAGTTCTTGAAATTAGCTATC
CCTTCTGGACTAATGTTTTGGTAAATATGTTTGTCTGATTGTTATATAACTTTGCATCCATCTCCTGCAACATTTTTTTAATTTTCTTCTGCTAATTACA
TTTTCACATTAATTTCTATCTATGTATGTATATAGTTTTGAATGGTGGTCCTTTGAGGTGCTTACATTACTTGCGGGAATTTTGCCTAATCCTCAACTTG
AAACCGCAGTTCTGTCAATCTGGTGTGTGGAATTCATTTCATTTCCATCATTATTGTTGAGAGGCTAAAATATATTTTACCTCATATTAAAATGATCATT
TTTGTTTTAAGTCCCTAAATTTTTCAGTTGTAATCGGAAAAAAAAAATTATTTATTGGTGACTCAAAATAAAAATTGGTCATTTATTACCTGACTTAAAA
CATATTTAAACCTTAAGTGAAACAACTCATATAGTTAAGTTTGATCGATCATACGCTTTTTTGCAGCCTTAACACAACCACATTGCACTACTTTATTCCT
TATGCCGTGGGAGCCTCTGCAAGGTAAAGGTTTGGTCCAAATTTGCCTTTTTTTCATGCTTCCAACACAGTAATTAGTTATTTTGATGATGGATGTTTGT
TTTGTTTTGTTTTGCAGCACTCGGGTTTCAAATGAACTAGGGGCAGGGAATCCAAAGACAGCAAAAGGTGCCGTTCGTGTGGTAGTGATTCTCGGGGTGG
CAGAGGCAGCTATTGTGAGCACTGTGTTCATTAGTTGCCGACATGTATTAGGATACGCGTACAGCAATGATAAGGAGGTAATCGATTACGTTGCAGAGAT
GGCTCCACTCTTGTGTGTGTCTGTTACTGCCGATAGTCTAATTGGAGCACTATCAGGGATTGCGAGAGGGGGAGGATTTCAGGAAATAGGGGCTTATGTG
AACCTGGGAGCCTATTATCTTGTGGGAATTCCAATGGGTTTGTTATTGGGTTTTCATCTGCAACTAAGAGCCAAGGGCCTTTGGATGGGAACTCTATCAG
GCTCTCTTACACAAGTCATTATTCTTGCTATTGTAACAGCACTAATAGATTGGCAAAAAGAGGTTCGTTCGTTTTAAGTTTTCATTTTTACATCCTTTGC
ATGTGTTGTGGAATGCTTCTGAATAACTGTATTTTGTGGTGGTTTGTTTCAGGCAACTAAAGCCAGGGAGAGAGTAGTTGAGAACTCAATTAAAGCTCAC
ATTTGATGTATTCCTATCAAGTTTAAGAAAACTGAAAAATAAATGACTGCAAGTGCTCACAATCGAAGTCTTTTTGGTTTCTGCATTGACTGTGATCAGC
AGTGGAGTGGAAAGATAGTGAGTGTGTGTCAGGTTTCATTAATTATGTTTTTGGTTCATTTTTTGTGTGTTTGTGAAGAGATTTAATCATGTTAACATAG
ACAACTTGGTGTGAATATGTGATGGGATTTTTGTACTGGATTTAATCATGTTAACATAGACAGCCTTCAGTTACATGTTTCACTAGTTTTTTTTATTGAC
TAGTTAATAGTAGCAATAGGATTTTATTTTTT

>Glyma.10G267800 | Chr10:49058164..49060854 forward
ATGAAAAGAGTCGGTTATCTTGTAGGGCCTATGATTACTGTCACTTTGTCACAGTATTTTTTACAGATTATTTCAATGGTGATGGTTGGTCACTTGGGTA
AACTTGCTCTCTCTAGCACAGCCATTGCCATCTCTCTCTGTGCTGTATCTGGCTTCAGCCTTATTGTAAGTGTCTTCCTTCCCCATCTCACTGCTTTCTT
CTTTAGTTTCAAGACCTACAATTGCATTGCACCCGATCAATATGAAAAAAGTTATTTATCAAAGAAGGGAAAACTATATATATATATAAAAAAAAAGTTG
AGATGAGTAAAAACACAAATTGCTTTTTGTTCCGGTTTAATTAGTAGTCTTGAATTCATGTAAGATTGTATCCCGTTAAAAATACTTGCAGGAAGAAATC
TTTGGAGTTAGTTGCTAGTTTTTTATGCTCTGGTTGTGCGATTAATTTTCCAAACTCGCTGTCTTTTTTGTACATGTCAAACAGTTTGCAATGTCATGTG
CACTGGAAACTCAATGTGGGCAAGCATACGGAGCACATCAATATCGAAAATTTGGTGTTCAAATGTACACTGCTATTGTCTCTCTTACTTTAGCCTGTCT
TCCTTTGAGTCCTTTGTGGGTATACTTGGGGAAGATACTCATTTTTCTTGGCCAAGACCCTTTGATTTCTCAAGAAGCTGGAAAATTTGCTTTGTGCATG
ACCCCTGCTCTCTTTGATTATGCAACACTTCAGGCCTTGGTTCGATACTTTCTGATGCAAAGTTTGACCCCAAATGAGAAGACCTGTATATCTCACTTAT
TTAAGTGAGATTAGAGGGTGAGATACAGTGATAGGGACTATTTGATAGGAAAAACAGTTAAGAAAGAAGGAGAGAGAAAATTATGGTGATTTTGTTTTTC
AGATTATAACTGCGGATTTGTTCACCGTTAAATCAGGTTGATTTTTGGATAGCAAATTCTACGCGCGTGATATTTCAAATTGTTCGATTGGATCAGGAAA
AAGATATCTAGAGAGAGATAAGTGTTTCGCATTATCTGTTCTATATTTTGAAGTTTGGAGGTTCTCTTGTATCTTCATTGATTATATCGGAGTTATTTAT
TTGGTCTAGACGGCCCGTGATTTTTACCCTTGCATTGAGGGATTTTTCACTTTAAACACAAATGGTGTCTTTCTTATCTTTAATTTCTATTTTGTGCTCT
ATACTTTATCCTCACAGATTCTGTAAGTTTGGGAGGATTTATTTCCACTGCCTATTACTCTGTTATAGAGTTTGTTATTGTGTTAGCCTATTTCTCCTTC
AGAGTTCCCTTGTCATAAGTTCCTCCATTACTCTTTGCTTCCATGTAGCTTTCTGTTGGCTACTGGTTTTTAAATTTGGATTTGGTAACTTAGGAGCAGC
ATTTTTTATTGGTACTTCATACTGGCTGAACGTGATTTTACTTGTATTATACATGAAATTCTCTATTGAATGTAAAAAGACTTGGGTACCAATTTCAACG
GAACTATTCCATGGCATTGGGGAGTTCTTCCGCTGTGCTATTCCTTCAGCTGGAATGATTTGGTGAATTTCTTCCTCTTATATTTTATCATTTATGTTGT
ATTTTTATTTCCTCTTTAATTTTCATCCATGTTTTTATTTTATTTTCATATTTGGTCATTGCAGCCTTGAATGGTGGTCATTTGAGTTACTATTCTTGCT
TTCTGGTCTTCTACCAAATCCAGAACTTGAAACTTCAGTCTTATCCATATGGTATATTATATGTACAACTTTTCTTAGTCCTTCAGACATGAATGACTGA
CTCTGTGTGTTTTAAAATCTCTATTGAAATTGTTATCCATACCCAAATATTGATTTACATTCTTGGAACTTGGAAGTAGTGCTAATCTGTTGTTTAAATG
TTAACGGATTTTCTGATCAGTTTATCAGTCACCACAACAATCTACACAATCCCAGAAGCAATTGGCTCAGCAGCAAGGTTTTCATTCACATAACTCCTTA
GTCCTAACCTCATCAGTTTGTGCCTATTTGTATTCATGTACAATAATAGTAAATGATTTTTTTTTCTTCTTTTTGGGTTGGTTACTCAGCACTAGAGTTT
CAAATGCATTAGGTGGTGGAAGTCCACAATTGGCACAAGTGTCTGTTTCTGCTGCTATGACTCTTGCAGCATCCGCGGCCATTCTGGTGAGCTCAATCAT
ATTTGCTTGCAGGCAGGTTGTAGGTTATGCATTTAGCAATGAGCTGGACGTGGTGGATTATTTCACAGAAATGGTTCCTCTTTTAAGTATATCTGTTATA
CTGGACACCTTACATGATACCCTCTCAGGTTTGGTTCTTTCTTTTCTCTATTTTTCAATGCATATCACTCTTACGCAATGTAGTCTGAAATTGTGCCATA
AAATATAATACTGTAAGGTAATACTTGTTCATTATAGGTATTGCTAGAGGATGTGGGTGGCAGCACAGAGGAGCATATGTAAACCTTGACGCTTATTATG
TTGTGGGAATTCCAATTGCTGCTATATTGGGTTTCTGCCTACAATTAAGAGGAAAAGGCCTTTGGATTGGAATACTGACTGGTGCCTTCTGCCAAACAGT
TATGGTATCCCTCATTACAAGTTGTACAAACTGGGAAAAACAGGTTTGCCTCCACTTCCAAGTCCATGCTTTGCACTTGGCACAGTCCTAA

>Glyma.20G123300 | Chr20:36600865..36604507 reverse
AACAGTTTTCCAGATTAAGTAATGATATTTTGGTTGAGAAACAAAAATTTCCGGATTCGGGAAGTATCCTAAAAGATGTGGATTCCACATTATTTTTCAT
CATTCTTGAGGAAAGAAGAGCAACCAAACGGCGGAAGGAAGATGAATCTCACATTTTTCGTTTCGTCTCCCTTCTTTCCTTCCTTTTTGGCTGGTACGAA
GGAGAGAGAGGCAGAGTATGTGATGAGATGGAGTGTGTTTGGTGAAGAAATGAAAAGAGTGGGTTATCTAGCAGGTCCTATGATCAATGTGACTTTGTCA
CAGTATTTTCTACAAATAATATCAATGATGATGGTTGGTCACTTGGGTAAACTTGTTCTCTCTAGCACAGCCATTGCCATCTCTCTCTGTGCTGTCTCTG
GCTTCAGTCTTATTGTAAGTCTCTTCCTTTCCCATCTCAACCTTCAATTTTACAAAAACTACACTTGCATTGCACATTTGTACACATTGGGTAATCTTGA
GTAGTGCCTTGTATGGTTATGTACAAAAAACATTTTCTATAAAAAAAAGTTTGATACTTAATTTAGGCCTATTAAAAAAATCATTTCTTTTCTTTGATGC
TTCTTGCTTTTATTCACATTGTTATTATGTGTCTTTAATGATCAATGACACACGGGAGTGTTCTTCCAAATTTGTGGTCACTGTAAATAAATACTGAGTA
TAACTCATTCCACATTTGTCATTGTGAACTATTTGACGATGAATTAGATTCATTTTAAGGGAACCAACCTTCTTGAACTTAATACTCTTTCTCTTGATAA
ACTACATTTACTATCAAAATGGACAAGGAAATGATGAGATTTTGAAATCGTTTAAATGTGATATAGTAATCATGCTTACATGTTATGTATAGTTTTTTTA
AGGAGATGTTTTTTTTTTTTCTGTCATGTGGAAGAATTTAATCTTTGTTTATGTTATAAACTATACTTATTTGTGTTATTTTAACGTTTATACAGTTTGG
TCATGTTTATATATAATTCTATTGTTTTATGTACGTATATTTTTTAAAATTTATATTAACGTATATGTATCCTATGCAATCTACATGGAACAGTTGCTTA
TCAGAGGGAGGGAAAACTATAAAATAAAAAAATGAAAGGAATGATGAAGATGAATACAAAACAGTGATTAGTCTTTTAATTTTGATAATTAGTAAAATTT
AATTATCCTTTTTAGATGTCCTGTATAATAAAAGGAAGAACTCAGAATTAGCTGCTACTTCGTAAGTTCTGGTTCTGCTATTAATTTTTCAACTCACACG
GGGATATTGAATTGGATGCTTGCTAGGATACTTGGTACATCTTGTGACTTATATTTGAGATTTGAGAATTAATTTTACTTTGTCTTTTATATATATCAAA
CAGTTTGGAATGTCATGTGCACTGGAAACTCAATGTGGGCAAGCATATGGAGCACAGCAATATCGAAAATTTGGTGTTCAAATATACACTGCTATTGTCT
CTCTTACTTTAGCTTGTCTTCCTCTGACTCTTTTGTGGGTCTACTTGGGGAAGATACTCATTTTTCTTGGCCAAGATCCTTTGATTTCACAAGAAGCTGG
AAAATTTGCTTTGTGCATGATCCCTGCTCTCTTTGCTTATGCAACACTTCAGGCCTTGGTTCGATACTTTCTGATGCAAAGTTTGACCAGTCCCCTCTTC
ATAAGTTCCTCCATTACTCTTTGCTTCCACGTAGCTTTTTGCTGGCTACTGGTTTTTAAATGTGGATTTGGTAACTTAGGAGCAGCATTTTCTATTGGTA
CTTCATACTGGCTGAACGTGGTTTTACTTGGGTTATATATGAAATTCTCTACTGAATGTGAAAAGACTCGGGTACCAATTTCAATGGAACTATTCCATGG
GATTGGGGAGTTCTTCCGCTGTGCTATTCCTTCAGCTGGAATGATTTGGTGAGTATCCTCCTCTTATGTTTGATCTTTTACGCTGTGTTTTTATTTCCTA
TTTAATTTTCAACCATTTTTTGTTTTTTTACATATTTGGTCATTGCAGCCTTGAATGGTGGTCATTTGAGTTGCTAACCTTGCTTTCTGGTCTTCTACCA
AATCCAGAGCTTGAAACTTCAGTCTTATCCATATGGTATGTACAACTATTTTTAGTCTTTCAGACATGAATGACTTTGCGTGTTTTAAAATCTCTGTTGA
AATTGTTATTCATACCCAAATCTCTGTTGTTTAAATGTTATCTACTTTCTGATTAGTTTATCAGTCACCACAACAATCTACACAATCCCAGAAGCAATTG
GCTCAGCAGCAAGGTTTTCATTCACATAAGTCCTAACCTCATTAATTTGTGCCTATTCGTATTCATGTTCAACAATAGTAAAGGATTTCTTTTTTCTCTT
TTGGGTGGGTTACTCAGCACTAGAGTTTCAAATGCATTAGGTGCTGGAAGTCCACAATCAGCCCAATTGTCTGTTTCTGCTGCTATGACACTTGCAGCAT
CCGCGGCCATTCTGGTGAGCTCAATCATATTTGCTTGCAGGCAGGTTGTAGGTTATGTATTTAGCAGTGAGCTGGACGTGGTGGATTATTTCACAGACAT
GGTTCCTCTTTTATGTCTCTCTGTTATACTGGACACCTTACATGGTACCCTATCAGGTTTGGTTCTTTCTTTTCTCTATTTTTCAATGCAAATCAGTCTT
ATGCAATGGACTAATGCAGTGTGAATTGTGCCATAAAATATAATTCTGTAAGGTAATACTTGTTCATTATAGGTATTGCTAGAGGATGTGGTTGGCAGCA
CTTAGGAGCATATGTAAACCTTGGAGCTTATTATGTTGTGGGAATTCCAATTGCTGCTATGTTGGGTTTCTGGGTACAATTAAGAGGAAAAGGCCTGTGG
ATTGGAATCCTGACTGGTGCCTTCTGCCAAACAGTTATGCTATCTCTCATTACAAGTTGTACAAATTGGGAGAAACAGGTTGCTTCCGCTTCCAAGTCTA
AGCTTTGCACTTGGCACAGTCCTAAAACACCATTTTCACAATGTTATGTCTTCTGATATATCAAACCAAACCTTTTGAATTTAATGTTTAAACTAGAGAA
ATTATTTTTTCAGTCAAAGAAAAGTAGCATCTTAACTCATGCAGTCCTATTCAGTTTTGAACAAGCACTACTAGTACGTATTTTTATAGCACATATGTAC
TACTAGTAGTAGTACTGACTGAAGAGTACTCATTGCTCGAGTGTCCTGAGATTGCAGGCAATTAAAGCAAGGGAAAGGACATTTCAGAGAAGTTTTGCTG
TAGAAGATGGATTAGTTTTAGCAAATGGAGAAAATTCCTTAAACCTGTCAGCCTGTTTAGTTGGTATTGGTAAATCATCTAACTTTGGGGGCACAAAAGA
TCAAAATTTAGAGTTAGAGGAGGGAAAAGTTCTCATAACGAGACAAGTGTAATGGTATTGATTTGTTATTTTTTCTCAATCCAATTTTAGTTAAGGTTCT
GTAACGTGAAGTAGAGGCAACTGGATGAATTCACGAGCAGCACTTCAATTTCCAATAACATCTTTGTATATAACAATGATAAATTAGAGTTCTTCCACTC
AATATGCAATGTAACCGAGCAGATTTCTGGATTTGTCAAACAA

>Glyma.20G123400 | Chr20:36606303..36608942 reverse
TTTCGACAAGGTGCAGTGATTCTGTGACCCTTTGGAGAGGATATGGAAAATAGTCTCTTAGATAAGGACTCAGAGCCGGAGAATCCTTCAGCTTCAGCCA
TAACATGGACTGTCTTTTCTCAGGAAATGAAAAGAGTGGGTTATCTAGCAGCTCCTATGATTACTGTGACTTTGTCACAGTATTTTCTACAGATTATTTC
AATGATGATGGTTGGTCACTTGGGTAAGCTTGCTCTCTCTAGCACAGCCATTGCCATCTCTCTCTGTGCTGTCTCTGGCTTCAGTCTTATTGTAAGTCTC
TTCCTTTCCCATCTCACTGCTTTCTTCTTAAGCTTTGAGACCTACTACTGCATTGAACCCAAATTATCAAAGGATGGAAAATTATTAAAAACAAAAGAGA
TGAAGATGAATTAAAACACAAAATGGCTTAAGTTCGAGTAGGATTCTCTTATGTGAAGATACCTATGGTCTCTAAAAAAATTATAAGAATTAATTGCACT
TTGTCTTTTTTGTACATGTCAAACAGTTTGGAATGTCATGTGCACTGGAAACTCAATGTGGGCAAGCATATGGAGCACAGCAATATCGAAAATTTGGTGT
TCAAATATACACTGCTATTGTCTCTCTTACTTTAGCTTGTCTTCCTCTGACTCTTTTCTGGGTCTACTTGGAGAAGATACTCATATTTCTTGGCCAAGAC
CCTTCGATTTCACAAGAAGCTGGAAAATTTGCTTTGTGCATGATCCCTGCTCTCTTTGCTTATGCAACACTTCAGGCCTTGATTCGATTCTTTTTGATGC
AAAGTTTGATCAGTCCCCTTGTCATAAGTTCCTCCATTACTCTTTGCTTCCATGTGGCTTTCAGTTGGTTAATGGTCTTTAAATCTGGATTTGGTAACTT
GGGAGCAGCATTTTCAATTGGTACTTCATACTGGCTGAATGTGATTTTACTTGGCTTATATATGAAATTCTCTACTGAATGTGAAAGGACTCGAGTCCCA
ATTTCAATGGAACTATTCCATGGGATTGGTGAATTCTTCACCTATGCTATTCCTTCAGCTGGAATGGTTTGGTGAGTTTCTTCCTCTTATATTTTATCTT
TTGTGTTGTGTTTTTATTTCCCATTTAATTTTCAACCATGGTATGTTTCTTACTTATTTGATCGTTGCAGCCTTGAGTGGTGGTCATTTGAGCTGCTAAC
CTTGCTTTCTGGTCTTCTACCAAATCCAGAGCTTGAAACTTCAGTCTTATCCATATGGTATGTACAACTTATCCTAGTCCTTCAGACATGAATGGCTTTG
TGTTTTAAAATCTGTGTTGAAATTGTTACTCAGCCAAATTTTGATTTCCATTCATGCAAGTGTTGCTAATTTGTTGTTATTGTTAACTATGTTCTGACCA
GTTTATCCATCATCACAACTATCTACACAATCCCAGAAGCAATTGGCTCAGCAGCAAGGTTTTGATTCACACAAGTCCTAAGCTCATCAATTTGTGCATA
TTCGTATTCATGTAGAATGATATTATAGTAAATGAATTTTTTCCTCTTTCTGATTGGTTTCTCTTTTGGGCGGGTTGTTCAGCACTAGAGTTTCAAATGC
ATTAGGAGCTGGAAGTCCACAATCAGCACGAGTATCTGTTTCTGCTGCCATGACTCTAGCAGTTTCTGAGGCCATTCTGGTGAGCTCAATCATTTTTGCT
AGCAGGCAGGTTTTAGGTTATGTATTTAGCAATGAGCAGGATGTGGTGGATTATGTCACAGATATGGTTCCTCTTTTAAGTATATCTGTTATAGTAGACA
CGTTACACGGTACCCTTTCAGGTTAGTTCTTGTTTGAACTATTTTTCAATGCACATTACTCTTCTTAAGCAATGGAATCTGAGTTATGCCTTCATTATAG
GTATTGCTAGAGGATGTGGGTGGCAGCACATAGGAGCATATGTAAACCTTTGAGCTTATTATGTTGTGGGAATTCCAATGGCTGCCATATTGGGTTTCTG
GCTACAATTAAGAGGAAAAGGCCTTTGGATTGGAATACTGACTGGTGCCTTCTGCCAAACAATTATGCTATCTCTCATCACAAGTTGTACAAACTGGGAA
AAACAGGTTTGCCTCCACTTCCAAGTCTTTATGGCTCTGCACTTGGCACAGTCTAAAAAAATCATTTTCTCAATGTTATATTTTCTCATATATCAAATAA
TTTTTTTTTGAATTTAAAGTTTAAGCTAGAGAAATCATCTCTATAGTCAAAGAAAAGTAGCATCTTAACCCATGCAATCTTTTTAAGATTCAGTTTTGAA
CATGTATTACAATACGTTTTTTTAGAGCTCACATGTACCACTAACTGAAATGTACTCATTTGTCGAATGTCTGAGATTGCAGGCAATTAAAGCAAGGGAA
AGGATATTTCAGAGAAGTTTTTCTGTAGAAGATGGATTAGTTTAAGCAAATGGAGAAGATTCTTTAAAGTTGTTAGCTTGTTTAGTTGGGTATTGGTAAA
ACATCTAGCTTTGGTTGCAGAAAACATCAAATTTTTCAGTTGCTTAAAGAGTTAGAGGAACAAAATGTTTTCATAAAAGGACAAGTGTAAGAATATTGAT
ATGTTATTTTTTCTCAATCCAATTTTGGTTAAGGTTCTGT

>Glyma.10G267700 | Chr10:49053473..49057599 forward
TTGAAAACCCGAAGGAGGACCAAACCAAATCAAAAGATCTTGCTTCTTTCTCTCTCTAGCATGGAAGAAAGTCTAGTAAAGAAACATGAGCAAGAAAGAG
TAACATGGGGTGTTTATAGTGAAGAAATGAGAAGGGTATGCCATATAGCAGGGCCTATGGTGGCAGTGGTTTCTTCACAATATTTGTTGCAAGTTGTCTC
AACTATGATAGTTGGTCATTTGGGTGAACTCTATCTCTCTAGCGCCGCCCTTGCCATTTCTCTATCAGGGGTCACTGGTTTCAGTCTTCTTGTAAGCTCC
TACTTGCTTTCCACTACTTTTTTTTTCATTTCTTCCAAAGTTGCTCCATATATAGCTAATTTATTTGATTTCCTTATTACTATTAGGTTTTAATATCATT
TTACTTATAAAGTACTACATCATATTCTTTTTGAGTCTTTATAACTATTTACTTACTGTATGTATGGGAAATTAAAAATAGGGCATTTTATTGAGTATAC
AAGATAAAAATAAAACCTTTTTATGAACAAAAAGACCGGCTATCGCCACCATTGCTGATTTGTTGCTGTAAACGATGTGATGACCAATTAGAACGTATAC
CAATATTTTTAATGATTCGACGTGACACTCTCAGTTGTACTACTTTTCAAACGGGCACGTCACTTTGAACTTTGAACCAAAAAAGATATGCACTCTGTAA
TTCTGTATATGTAAAACACCCATTTTCACATTTCAATTGTATGATTTTATTACGTCATATCATCCGTCTTATAAAAAGTATTAATAATATATTTTAAATT
TATTTATAATTAGAAGGGGAGCTCACTAAATAAAAAATAAGGCTTACAAATTTTCATAATTTCAAACAATAATAAAAATGTATTTCCAGCATTTCTTTTA
TCACTACTATTGTCTTCTATTCCTTTGTCTCTGACGCTATACCAGAATGTCCGCCGAAGACTCGTTGCACTACGCCGACCAGAAGCACCATCACAATGCG
GTCTTCTATTGCATATATAATAAAAGTTACTCACCTTTTGGATAGATTCATGCATATAGGTTGGATCGAGTGGAAAGAAATAAAAATATATAAATTTAAA
TTAAAGTAAAATATAAAAATAAGTTTTATATTATTTTACTGTTTATTTATCTTATTTTTTTAAGTTCTTTTTCTTTCAAACAAACACACCCTTAACTTTT
TTTTAAGAGAGCTTGTAAAAAAAAACTCATAACATGTTTCTAACATCTTTTTTAGAAAAATAATTTATGCTATGAAAATATATTATCACTTTTACAAAAA
TAGTTTAATGTATTTTCTCTTTTAATTATGGAAATAACATATACATAAGTAATTACATAATAAGTATTTATTCATCAAATTCTTAATTAAGAGGAAAAAA
GAAGCAACACGCGTATAGTTTCTACCATCCGTACCTACGAAGAAAACAAAAATAAACGAGATCAACGAAGCACTAGCTGCTATATATTTAAACTACGATA
TTATATAACCTACTCTGGTGAAGATCTGGATACTATGCACAAGGTTTTGGGTTCTAACCTTGTTGCTACCAATCTACCATTGTACACATTAAAAAACCTG
TGATATTAAATTATTGGCAAGGTTCCAATTCACTCACTCTAGAAGGCAAACAAAATAAGATGCTTATCCTGTTATTACTAAATGCTTTTTCTGATAATGG
TGATATTACTAAATGCTTGCATTTAGTGACTAGTTTATCTTCTTTATAAACTATTTCAAGCGTGTTATGCTGCTAAAAATTACCATTATTTCCTTTCCTT
GTAACTTGGTGCATTGATAATTTGCATATGCTGATAGATGGGAATGGCTAGTGGACTGGAAACAATTTGTGGACAGGCTTATGGAGGCCAACAATATCAA
AGAATTGGAATACAAACATACACTGCTATATTCTCTCTCATTTTGGTTTCTATTCCGGTGTCTCTCCTTTGGATCAACATGGAAACCATACTAGTTTTCA
TAGGCCAGGACCCTCTAATTTCCCATGAAGCAGGGAAGTTCACAATTTGGCTTGTTCCAGCACTTTTTGCATATGCAATTCTACAGCCGTTAGTTCGATA
TTTCCAAATCCAAAGTTTGCTTCTTCCCATGTTTGCAAGTTCTTGTGTCACACTCATTATTCATGTACCTCTTTGTTGGGCCTTGGTATTCAAGACAAGT
CTGAGTAATGTTGGTGGAGCATTGGCTGTGAGCATTTCAATATGGTCAAATGTGATTTTTCTTGTGTTATACATGAGATACTCTTCTGCATGTGCAAAAA
CCCGCGCACCGATTTCTATGGAGCTGTTCAAAGGAATGTGGGAGTTCTTTCGTTTCGCTATCCCTTCAGCAGTAATGGTTTGGTGATTATTCTTCCTGTT
CAATTAATATTCTACATATTCATGTTATCAGATAACAAAAATTTTCTATAATATGTATTGTATTGTTTTTCCAAAAAGAGTATTTAACATAATTTTTGTA
TTCATGTTGATGATGGTAGCCTTGAATGGTGGTCATATGAGCTGCTTGTATTGCTGTCAGGACTCTTACCAAATCCACAACTTGAAACTTCAGTTCTGTC
CGTTTGGTATGTATGTTACATTATTTTGGTTTTCCATAAACTGCAACTAATGATCTACAATTTCATTTTGTACCAATCTATTTCAGAGATTTTATAAATT
ATTGTGATTGAACTATTTTTTTGTCATTTTTCTACAGCCTCAACACCATTGCAACTCTCTATACGATACCCTTTGGAATTGGTGCAGCAGCAAGGTTGCA
TCATAGACACACTCATTCTTCATAGTTTAAATGTTACACTAGTACCCAGCTGCCTTGAGCATGCATCTAAACTAATTATCTTTTCTCCTCTCTGATTATA
GCACAAGGGTTTCAAATGAATTAGGAGCTGGGAATTCACATGCTGCACGTGTTGCCGTGTTAGCAGCAATGTCTCTTGCAGTAATTGAGACAAGTATAGT
AAGTGCAACACTCTTCGCCTGCCGCAATGTTTTTGGTTATATTTTCAGCAATGAGAAGGAAGTTGTTGATTATGTCACTGCCATGGCTCCTCTGGTATGT
ATATCTGTTATACTAGACAGCATACAAGGTGTTCTAACAGGTATTCTTTCTCTCAATGGCCATCCTTAAGTGAATTTTATATAGAAGAAAAGCTTTCTCT
CAATTTGTTTAATTATTTGGTTCAGGGATTGCTAGAGGTTGTGGATGGCAACATTTAGGGGTTTATGTCAATCTAGGGGCCTTCTACCTTTGTGGGATTC
CAATGGCTGCCTTATTAGCATTTTTGGTGCGATTGGGAGGAAAAGGACTATGGATTGGCATACAAAGTGGTGCTTTTGTTCAATGTATTCTACTTTCTAT
CATAACAGGTTGCATAAATTGGGAAAAGCAGGTATAACCTCTAATCTTTGTATTCTTGAAGAGTGTTAGTAACACACTTTTTTTATATACATCCTTAATA
AATTTTAACTAATAACAAAGTGAGTCCAAAAGAGTGTATTAATGTGTTAACTTTTCTCTTGTCTTGGAACTCCAAACTTCAATTTTGTTGGTTGCACGAA
GTTAAAAGGATAATAAAGTACAGTAATTCATCTTCTGGCAGCCACTTTATTTTGCTTCAATAAATCTTAAAGCCCTTGTGAAGGTAGTATCCCTTTTAGA
CATTTTGTTTCTATTGTTTTGTTTTACTATGCAGGCAATCAAGGCAAGAAAGAGGTTATTTGATGAAAAAATTTCAGCAGACAATATATTGGTATGAGTA
TGACAAGCCCTCAAAGGCCAATTTTCTAAAGATGGGGACTATTTTCCGTGAGTGAGGAGTAGGAGTTTAGTTTTTGGTTTTTTGGAAGTGGTTAGCTATT
TGAGCTTGTACCTTCGTGCATCTGATTAAAGAATAATAGTAGCATTTTTTGTCATGATAATAGGATTAAACGTTTAGCAATGTTTCGAATGCAAAATGTA
ATTTTGAGCTATGCCAACTATTTTTCATTTAATAGCCGATCCTTGATAGTTCCCGTCTTATACATTTAACTTTTTTTTGGTATAATTAGTAGGTATCTCA
TAATTGTCATTTAACACCTAACTAAAT

>Glyma.20G123500 | Chr20:36610054..36614582 reverse
AACAAGTTTCCAACCCCGAAGGAGGACCAAATCAAACAAAGAAAGAAGGTCTTGTTTCTTTCGTTTCTTTGTTTCTCTTTCTCTAGCTAGCTAAGTCCGT
TCGTGATCGAAAAGAGAAAAAGATCATGGAAGAAAGTCTAGTAAAAAAACATGAAGAAGATAGAGTAGTAAGATGGGGTGTTTATAGTGAAGAAATGAGA
AGGATCTGTGAAATAGCAGGGCCTATGGTGGCAGTGGTTTCTTCCCAATATTTGTTGCAAGTTGTGTCAACCATGATAGTTGGTCATTTAGGTGAACTCT
ATCTCTCTAGCGCAGCCTTAGCCATTTCCCTATCAGGGGTCACTGGTTTCAGTCTTCATGTAAGCTCCTACTCTTACACTTCTTTTATTTTGCATATCAC
TCATTTCTTCTAAAGTTTGCTCCATGGCTAGCTTCTTCTCTCATTATTTCCTTATTACCTTTAGGTTTGAGTAACATTTCACTTGGAAAATACATCATAT
TCTTTTTTTTAAGTCTTCATGAAAAATTTATTCACTTAATTTATGGGGATTTTTTTTTATTACTTTAAAAACACGGCATTTTGTCGACCATTGAGTACAC
AAGGATGTTAATGACTAATTAAAACGTATACCAACATTTTTAATGATTCGACGTGATTCTCTGACTTGTACCTTTCTAGTGGGCACTACAGTTCTTTTAT
ACAGGACGGATCCAGAAGTGGATAAAGGGGAGGATCTATCTAATTTTTAATAAATAATAAGTTTAACAAAAAATATTTAAATATATGATGTACTAGTAGA
AGAAAATTAAACATATGTTAAACGAAAAAGGAATTTAAAATTTAGATATATTAATTTTATGTGCAATGAGTCACTGTTGTTTACATATATGAATTTGTTA
GCATATATATATTTTTAATATAAATACATGAGTAAGTTATAATTAAAAATTATCTTAAAATATGTTAATGATATAATTTATTAACAGACTGATATTAAAA
ATAAAAATAAAAGTGTATTTATGAGAAAAAATTGAACTAAAAAAGAAATGCACACTGTAAATATAAGCACCCATTTCACATTTTTTTTTTGTATTTTTAT
GATTTTATTACGTCATATCGTCCATCTTATAAAAGTATTAATAATATATTTTTCTAACACATTTTTTAAATTTATTTATAATTATAAGGGGAGCTCACAA
AATAAAAAAACAAGGCTTACAAATTTTCAATAATTTGAAACAATAATAAAATATATATAATAAAAATGTATTTCTAGCATTTTTTTTATCACTATTCCTT
TCTCTCTCTCACGCTATACCAGAATGTCCGCCTAACATTGTGTTTGACTCATTGCACTAGGCCGACCAGAAGCACCATCACATGTGAGTGAGACTGTAGC
AGCACAGGAAAATGCGGTCTTTGCTCTAGTTTGCCATTGATATTAATATTAAAAAGATGACAGAATGTTCTATTGCATATGTAAAAGTTGCTTACCTTTT
CAACAGATTCATGCATTTAACCTTAAGTGATGAGATCAGTACCTAGACAAATATTTAAGAGATCGTGCTTGTAAAAATAACTCATGACATGTTTATTAAC
CTTTTTATAGAAGAATAATTTATGAAACTAAATTGTCTTTTGTCACTTTTAGAAATTTTTTTAGTGTAATTTCTTTTTTAATTATAGAAATAGCATATAC
ATAAGTATTTATTCATCAAATTCTTAAGTATTAAATATGTATTTAGTTCAAGTAAGTTAGTATTTTTTTTAAGTCTATGTAAGTTTATTATTTTTCTAAT
TTTAGTTTTTGAAAGTTTTTTATTTTTTATTTTTAATTCTTATAAGTTTGTGTTTTTTTAAGTTTAGTTTTTAAGATTCTAATTTTTTTTATTTATAGTT
CCATAAATTCGTGTTTAAAAAAGAAACAAAATGTTTTAGGACGATCCGAATTAACAAAATGCAAACATGAACTAAAATTAAAAAAAAAAAATCAACTTAC
AAGCGTTTAAAAAATAAAAATAAAAAAGCTATGTCAAGCATGTTATCCTGCTAAAAATCATCATTATTTTCTTCCCTTGTAACTTAGGGCATTGATAAAT
TGCATATGCTAACAGATGGGAATGGCTAGTGGACTGGAAACAATTTGTGGACAGGCTTATGGAGCTCAACAATATCAAAGAATTGGAATGCAAACATACA
CTGCTATATTCTCTCTCATATTGGTTTCTATTCCCGTGTCTATCCTTTGGATCAACATGGAAAGCATACTAGTTTTCATAGGCCAGGACCCTCTAATTTC
GCATGAAGCAGGGAAGTTCACAATTTGGCTTGTTCCAGCACTTTTTGCATATGCAATTCTTCAGCCGTTAGTTCGATATTTCCAAGTCCAAAGTTTGCTT
CTTCCCATGTTTGCAAGTTCTTGTGTCACACTCATTATTCATGTACCTCTTTGTTGGGCCTTGGTATTCAAGACAAGACTGAGTAATGTTGGTGGAGCAT
TGGCAGTGAGCATTTCAATATGGTCAAATGTGATTTTTCTTGGATTGTACATGAGATACTCTTCTGCATGTGCAAAAACCCGCGCACCCATTTCTATGGA
GCTGTTCAAAGGAATGTGGGAGTTCTTTCGTTTTGCTATCCCTTCGGCAGTAATGGTTTGGTGATTATTCTTCCTGTTCAATCAATATTCTACATATTCA
TGTTATCAGATAACAAAAAGTTTCCTTGTCCAACAAATTTTCTATAATGTATTGTATTGTTTTTCCAAAACTAGTATTTAACATAATTTTTGTATTCATG
TTGATGATGGTAGCCTTGAATGGTGGTCATATGAGCTGCTTGTATTGCTGTCAGGGCTGTTACCAAATCCACAACTTGAAACTTCAGTTCTATCTGTTTG
GTATGTATGCTCCATTATTTTGGTTTTCTTTATAACCCATATAAGACTTTTTGGTCCCTTTATAAACCGAAACTCATGATCTACAATTTCATTTTGCTAC
CAATCTTCAATTTCTAAGATTTTATAAATTGATGTGATAGAACTAATTTTTTTTGTCATTTTTCCACAGTCTAAACACCATTTCAACTCTCTATATGATT
CCCTTTGGAATTGGCGCTGCAGCAAGGTTACATCATAGACATACTCATTCTACATAATTTACACTTTACATGTTACACAACCTAGCTACCCTAAGCATGC
ATCTAAACTAATTATCTTTTCTCCTCTCTGATTATAGCACAAGGGTTTCAAATGAACTAGGAGCTGGGAATTCACATGCTGCACGTGTTGCCGTGTTAGC
AGCAATGTCTCTTGCAGTAATTGAGACAAGTATAGTAAGCGCAACACTCTTTGCCTGCCGCAATGTTTATGGTTATATTTTCAGCAATGAGAAGGAAGTT
ATTGATTATGTCACTGTCATGGCTCCTCTGGTATGTATATCTATTATACTAGACAGCATACAAGGTGTTCTCACAGGTAATCTTTCTCTCAATGGCTATC
CTAAAGTAAATTCTATATAGAAGAAAAGCTTTCTCTCAATTTGTTTAATTCTTTGGTTCAGGGATTGCTAGAGGTTGTGGATGGCAACATTTAGGGGTTT
TTGTCAATCTAGGGGCCTTCTACCTTTGTGGGATTCCAATGGCTGCCTTATTGGCATTTTTGGTGAGACTGGGAGGACAAGGACTTTGGATTGGTATACA
AAGTGGTGCTTTTGTTCAAACTCTTCTACTTTCTATCATAACAGGTTGCATAAATTGGGAAAAGCAGGTATACCCTCTAATCTTTGTATTCTCCAGGAGT
GTTACTAACAAACTTTTTTTATATACATTCTTAGTTTCTAATAAATTCTAACTAATAACAAAGTGAGTCCAAAAGAGTGTATTAATATGTTGTTAACATT
TCTCGTCATAAAACTTCAATTTTGTTATGTTTGGTCGCACGAAGTTAAAAGGATAATAAAGTATACTAGTTCATCTTCTTTCAGCCACTTTTTTGCTTTA
GTAAATCTTAAAGCCCTTGTGAAGGTAGTATCCCTTTTAGACATTTTGTTTCAATTGTTTTGTTTTACTATGCAGGCAATCAAGGCAAGAAAGAGGTTAT
TTGATGATCAATTTTCTGCTGACAATATATTGGTATGAGTATGACAAGCTCTTAATTAAAGGCCAATTTTCTAAAGATGACTATTCTCCGTGACTGAGTA
GGAGTTTACTCCATTCATTATAAGAAATGAGTTTTTGGCTTTTTGAGGTGGTTAGCTATATGAGCTTATACCTCCGTGGAGAATATCGTAGCATTTTTGG
TCATGATAACATGATAAACGTTTAGCAATGTTTGCTATACTAGCGCTGTGCTGCTTGGAATGCAAAATGTCATTTTGAGCTATTCAACTCTGTTTCATTT
TCCCTAAAAAAAACTGTTTCATTTAATTGCCGATCCTTGATAGTTCCCGTCTTATACATTTAACCTTTTCTTTTTTGTATAATTAGTAGGTATTTTTGAC
TCCCAACTAATTTTTCTCTCAACTTATAA

>Glyma.04G102100 | Chr04:9415963..9417864 forward
TTAGCTTGCTCAAAATCATCATTATTTTACTCTCCAATAACTTTGGTGCATTCTTAATTTGCACATGTTAATAGATGGGAATGGCTAGTGGACTGGAAAC
TATTTTTTGACAAGCTTATGGAGCTCAACAATATAAAAAAAATTGGAGTGCAAACATACACTGCTATATTCGCTCTCACATTCGTTTGTCTTCCTTTCAC
TTTCCTTTGGATCAACAGGGAAAAGATACTAGTTTTCACAGGTCAGGACCCTCTAATTGCAAAAGAAGCAGGAAAATTCATAATTTGGCTTATTCCAGCA
CTTTTTGCATATGCAATTCTACAACCATTAGTTCGATATTTTAAAGTGTAAAGTTTGCTTCTTCCGATGCTTATAACATCTTGTGTCACTCTATGTGTCC
GCATACCCCTTTGTTGGGTTTTGGTGTTCAAGACTAGACAGAATAATGTTGGTGGAGCATTAGCAATGAGCATTTCAATATGGTCAAATGTGTTTTTTCA
TGGATTATACATGAGATATTCTCCTACATGTGCAAAAACTGGTGCACCCATTTTTATGGAATTGTTCCAAAGACTTTGGGAGTTCTTTCGTTTTGCTATC
CCTTCTGCAGTAATGATTTGGTGATTTATCCTTCTGTCCACTTGAAGTTGTATATATTCAGTTTATGAAATTATAAATTGCTTCCTTTTCTAAAAAAATT
CTATAATGTATTTTTTTCATGTTGATGATTGCAGCCTTGAATGGTGGCCATTTGAGCTGATTATCTTGTTGTCTGGGCTGTTACTAAATCCACAACTTGA
AACTTCTGTTTTATCCGTTTGGTATGTTTGCTACATTCTTTTTTTTTTAATCTTTCTTTTCAGCATGTAAGATTTTTCTGGCCCTCCTTGAACTGCAACT
AACGACCTAACAATTTTATGAGATTTTGTAATTAATGTGGGCCTGCTAATTCTTCATCATTTTTCTACAGTCTTAACACCACTTCAACTCTCTATGCAAT
ACCTTTTGGAATTGGTGCTGCAGCAAGCTGACATCATAAACATGTCCTTTCTAAATATTTTACATGTCCTAAGCTATGTAGATAGCCACCTTAATTTGCA
CATATACCTAAACTAGTTATTCATTTCCTCTCTGATTGTAGCACAAGAATTTCAAATGAATTAGGAGTGGGGAATCCACGTGGTGCCCGTGTTTCTGTGA
GGGCTGCAATGCCTTTTGCGGTCGTTGAGACAACTATAGTTAGCGGAACCCTCTTTGCTTGTCGCCATGTTTTTGGCTATATTTTTAGCAATGAGAAGGA
AGTTGTAGATTCTGTCACTCTCATGGCTCCTTTGGTTTGCATATGGGTTATACTTGACAACATACAAGGTGTTCTCGCAGGTAAACTTCCTTCTTGTCTA
TTTACATTTTATATTGAAGAGAATTATTCTCTCAATTTGATTTACAATTCTTTGGCTTAGGGGTTGCTAGAGGTTGTTGATGGCAACACATAGGGGTTTA
TGTCAATATAGGAGCATTTTACCTATGCGGGATTCCAATGGCAGTCTTATTGTCATTTTTGGCAAAACTAAGAGGAAAAGGACTTTGGATTGGTGTACAA
GTTGGTTCTTTTGTTGAATGTGTTCTACTTTCTACCATAACCAGTTGCATAAATTGGGAACAACGGGTATTTATCATTTAAAAGGACAATAAAATATTGT
CCTCCAATGGATCTTTTAGATATCTCAAATGTATAATTTTAGTCTTTCAAGTTCTAATTCAGTCAATTAAGTTTTCACATAGCACAATTATGTGATTTTA
ATAAATATTTATGGATACCATATATACTTGTAAATATTGACAAAAATATGTAAATATAATATTTATCCAACAAAATATACTAAAGGATTATTGTCTTTAC
CC

>Glyma.06G103400 | Chr06:8233858..8237566 forward
AGAGGCAGCGCAGAAGTTCTTTCATACAGACTCAGATATTTTTTACATTACACTCTTTGTCTCGATTGGGTTGTTAGTAGTAATAAGAAAGAAAAGTATG
GAAGAGAATCTATTAGTATTAGCAAAAGGAAGTGGGGAGGAGCAGAAAGTTGCATGGGAAGGTTTGGGTGAGGAAATGAAGAGGATGATTGACATAGCAG
GGCCTATGGTGGTTGTGACCGCTTCCCAGCGTTTGTTGCAGGTTGTGTCCGTCATGATGGTTGGTCACCTCAACGATGACCTCTTTCTCTCCAGCGCCGC
CTTAGCCATCTCTCTTACTGCTGTCACGGGTTTCAGTTTTCTTGTAAGCTATATACTCAATCTTATGTTGTTTTACTCCAATGCTAAATTTGTTTTTTCC
TGTAAAATTAATAAGGTATAGATTTCGTTGCTGTAAAAATTTTCATTTGATTTCGCTTCTCACAAAATTAAAATATATCAAGTTAGATTTGATTGACTTT
CATACCTACATGACACTCTCATTCATATGCTATCAGTCTCTCATTGGTGCATGCATTTGTTACTCTCCTTGTGATTTATGGACAAATTGCAAAAGAAAAC
GCTGTAATTTTAAAAATAATAAAAAGTGAAGAGAAACGGGTGGTCCACCATCTCTGCTCTCTGCGCCGTCTTCTTCAGTACCACCACCTCCTTGTCCACC
ACCTTCATGGCGTAGAATTAGGTGTCGTTGTCACCGTCGTGGCAGAGGCAACAGAGGTAGACGGTGCCGATGCGGCGGAGGATGGCAGAGTAGGCGAAGT
CGGAGGAGCGATGCGGCTTCACGAAGAGGCTCTCCGGGGAAGAGGAGGAGGGAGAGACAACTAAAGTTGGTGTTGCAAGCAATTTCGTTGCTCATTGATG
TCCGTTGAGTGGAGGAATTCATAGTCTCTGATTTCATCCTAGAATCGCGTCCACCAACTCTCTCCATTAGCCTCCTGGAACCGCACCACGGCTCATAGAA
ATTATAAAGGTTGAAGTAAGAGAGAGAAAGAGAGAGAGAGAGGAGGAGGAGGAGGAGGAGGCATTTTTGGAGAAGGAAGGTTATGTGGTATTTATGGGAA
AGGTGTGGTGGCAAAGGAATATTGTGTCGAATTGACAATTGGGACCGAACGTGTGTGGGAGGAAATGAGAGTGGGGTGAATTCTGGGGTGGGATCGAAGG
TTACTCCATTTTGTTCCAGCTATGGTGGTTGAAGTTTGAAGAAGATGAGGAAGGTTAGGGAAAAGGGATTTAAAATTTTTGACAATTTCGGAGAGGGGTG
GGGGTTTCAAAGCGGGAATTGTGACCTTTTTTTTTTTAAAAAAAAAAACTTAAGGATAGTAGCCGATAATTGGATCATTGTAGGAATTAAACATAGGCAT
GAAAGTCAATCAAGTCTAATTCCGGAAAAAATAATATTTTAATTTTAGAAGGATAAAAGCATATGAAAATTTTTACTGGAATAAAATTCATATTTTGATA
ATTTTATCTAGACAGAAAATATATTTTAACATTTATATATTATGTTTTGGTTGCATTTCTCATTACTTAACATCAATCTAGAGTGTTGATTAAGTTTATC
TTTATCAACTATTTTTCACATGTTAGCTTGTTCAAAATCATCATTATTTTATTCTCCGAAAACTTAGTGCATTCTTAATTTGCACATGTTAATAGATGGG
AATGGCTAGTGGACTGGAAACTATTTGTGGACAAGCTTATGGAGCTCAACAACATAAAAAAATTGGAGTGCAAACATACACTGCTATATTCGCTCTCACA
TTCGTTTGTCTTCCCTTCACTTTTCTTTGGATCAACATGGAAAAGATACTAGTTTTCATAGGTCAGGACCCTCTAATTGCAAAAGAAGCAGGAAAATTCA
TAATTTGGCTTATTCCAGCACTTTTTGCATATGCAATTCTACAACCATTAGTTAGATATTTTCAAATGCAAAGTTTGCTTCTTCCCATGCTTATGACATC
TTGTGTCACTCTATGTGTCCACATACCCCTTTGTTGGGTTTTGGTGTTCAAGACTAGACTGAATAATGTTGGTGGAGCATTAGCAATGAGTATTTCAACA
TGGTCAAATGTGATTTTTCTTGGATTATACATGAGATACTCTCCTAGATGTGCAAAAACTCGTGCACCCATTTCTATGGAATTGTTCCAAGGACTTCGGG
AGTTCTTTCGCTTTGCTATACCTTCTGCAGTGATGATTTGGTGATTTATCCTTCTGTCCACTTGAAGTTGTATATATTCATTTTATGAAATTATAAATTT
CTTCCTTTTCCAAAAAACAATGGTATAATGTATTTTTTTTTTCATGTTGATTGCAGCCTTGAATGGTGGTCATTTGAGTTGATTATCTTGTTGTCTGGGC
TGTTACTAAATCCACAACTTGAAACTTCTGTTTTATCCATTTGGTATGTTTGCTTCATTTTTTTTTCTTTTCAACATGTAAGACTTTTCTGGCCCTCCTT
GCACTGCAACTCACGACCTAACAATTTTATGAGATTTTGTAAATTAATGTGGTCAAGCTAATTCTTCATCATTTTTCTACAGTCTTAACACCACTTCAAT
TCTCTATGCAATACCTTTTGGAATTGGTGCTGCAGCAAGGTGACATCATAAACATGTTATTTCTAAATATTTTACATGTCGTAAGCTATGTGTCTATCTA
CCTTAATTTGCACATATACCTAAACTAATTTAATCATCCATTTTCCTCTATGATTGTAGCACAAGGATTTCAAATGAATTAGGAGCGGGGAATCCACATG
GTGCCTGTGTTTCTGTGTTGGCTGCAATATCTTTTGCAATCATTGAGACAACTGTAGTTAGCGGAACCCTCTTTGCTTGTCGCCATGTTTTTGGTTATGT
TTTTAGCAATGAGAAGGAAGTTGTAGATTATGTCACTGTCATGGCTCCTTTGGTTTGCATATCGGTTATACTTGACAACATACAAGGTGTTCTCGCAGGT
AAACTTTCTTCTTGTCTATTTACATTTTATATTGAAGAGAATTATTCTCTCAATTTGATTTACAATTCTTTGGCTTAGGGGTTGCTAGAGGTTGTGGATG
GCAACATATAGGGGTTTATGTCAATATAGGAGCATTTTACCTATGCGGGATTCCAATGGCTATCTTATTATCATTTTTTGCAAAAATGAGAGGAAAAGGA
CTTTGGATTGGTGTACAAGTTGGTTCTTTTGCTCAATGTGTTCTACTTTCTACCATAACAAGTTGCATAAATTGGGAACAACAGGTATTTATCATTTAAA
AGGACAATAAAATATGGAATACTTTGTCTGGCTACAATTTTTTATTTTGCTTGATAAAGTTGAATACCTTACTGGGGGTACCGCTCTAGGCATTTTATTT
CGATTGCGTGGTCTTGCTAATGCAGACAATCAAGGCAAGAAAGAGATTATTTGGTAGTGAATTTTCAGCAGACGATAGACTGATATGAGAAGCCCATGAT
CAAAGGCCCATTCTCTAATGATGGTGACAGCTTTCCATGAGTGAGTCAAGTGGGAACTCAATCTTCATTGGCAGTTTGAAAGTAATGCTTGATGATATTT
TGACTTTTTTCTTTATCTTATTGTTGTTGTTTCTCGTTAAAATAAAATAAAAATGAATATATGGAAAGCCTCTGAAATAAATGCATAATTTTTTAGAGAC
CAAAAAGAA

>Glyma.10G267300 | Chr10:49038069..49040697 reverse
TCAAATAGTAGTATAGGAATTAGAAGTAGGAAAGAAAGAAAAGCATGGAAGAAAATCTAATAATAACAAAACAAAGTGAGAAGCAGAGAGTTACATGGGA
TGGTTTGGGTGAAGAAATGAAGAGGATCATTCGAGTAGCAGGGCCTATGGTGTTTGTGTATGCTTCCCAGAATCTGTTGCAGGTTGTGTCCATCATGATG
ATTGGTCATCTCAATGACGAGCTTTTTCTTTCCGGCGCCGCCTTAGCCATCTCTCTTGCCACCGTCACTGGTTTCAGCCTTCTTGTAAGCATGCTAAACT
CAATCTTGCTTGCATGCCTTGTTTGACTTTGACTCCTCACAATTATATGTTTTTCTTTATGAATTTAATGATCATGCACTTACAGTACATTAGTTATGAA
GTACAAATTTAACCATGCTAGAAAGTATTGATAAGCCACTGAACCAACACAAATGAAGTTGAATGCTCATGTTTAGAGTGGTCAAGCTTATCTTCCTTAA
CAAGTTTTCTAATATGTTAGCTTGTTCAAAATTTGCACATGCTAATAGACGGGAATGGCTAGTGGACTTGAAACTATATGTGGACAAGCTTACGGAGCTC
GACAATATCAAAAAACTGGAGTGCAAACATACACTGCTATATTCTCTCTCACATGTGTTTGTCTTCCCCTGACTATAATTTGGATCAGCTTGGAAAATAT
ACTAGTTTTCATAGGCCAAGACCCTCTCATTGCACACGAAGCAGGAAACTTTATAATTTGGCTTCTTCCAGCACTTTTTGCATATGCAATTCTGCAGCCG
TTAGTTCGATATTTTCAAATGCAAAGTCTGCTTCTTCCCATGCTTGCAACTTCTTGTGTCACTCTTTGTCTCCACATACCCCTTTGTTGGGCTTTGGTGT
TCAAGACTGAACTGAGTAATGTTGGTGGAGCATTAGCAATGAGCATTTCAATATGGTTAAATGTGATTTTTCTCGTATTATACATGAGATACTCTCCTGC
ATGTGAAAAAACCCGTGCACCAGTGTCTATGGAGCTGTTCCAAGGAATTTGGGAGTTTTTTCGCTTTGCTATCCCTTCTGCAGTGATGATTTGGTGATTC
ATCTTCCCATTCACTTCAAGTTTTATATATTCATTTTATCCCATTATAAATTGTTTAATTTTCCCAAAAATAAAATGTATAATGTAACATTTTTCTTTTC
ATGATGATGACTGTAGCCTTGAATGGTGGTCATTTGAGCTGCTTATCTTACTGTCTGGGTTGTTACCAAATCCACAACTTGAAACTTCAGTTCTATCCAT
TTGGTATGTTTGCTACATTCTTTTATTTATCAACGTAACATAGAGGATAGGACTTTTTAGGCCCTAGTTGATTTTCAACTCACCATCTAACAATCTTATG
AGATACTATGAGTAACATGATTGTGCAAATTGTTTGACATTTATCTCAACAGTCTCAACACCATTTCCACACTCTATGCAATAGCTTTTGGAATTGCTGC
TGCAGCAAGGTGAAAGTGAACCCATAAACATGTTCTTTCTAAAAAATTTACATGTGACAATTTATATATCTGAACTAGTTATCCTTTCTCCGCTTTGATT
GTAGCACAAGGATTTCAAATGAATTAGGTGCAGGGAATCCACATTCTGCCCGTGTTGCTGTGTTGGCTTCAATGTCTTTTGCAATCATGGAGGCAACTAT
AATTAGCGGAATCCTCTTTGTCTGTCGCCATGTTTTCGGTTATACTTTCAGTAACAAGAAGGAAGTTGTTGATTATGTCACTGTTATGGCTCCTCTGGTT
TGCATATCTGTTATACTGGACAATATACAAGGTGTTCTCGCAGGTAAAATTCCCACTTCTCCATTGTTAAGTAAATTTTATACATAAAAGAAGCATCGTC
TCAATTTGTTTAACAATACTTTTGCTCAGGGATTGCTAGAGGTTGTGGATGGCAACACATAGGGGTTTATGTGAATCTAGGGGCATTCTACCTCTGCGGG
ATTCCTGTTGCTGCATCATTGGCATTTTTGGCTAAAATGAGTGGAAAAGGACTTTGGATTGGTTTACAAGTCGGTGCCTTTGTTCAATGTGCTCTACTTT
CTACCGTAACAAGTTGCACAAACTGGGAACAACAGGTATATATGCTCTCTAACGGTCTAACCTTTATCTTAAAACTCCAAAACTAAGGATATGGTATTTT
ACTATATTTGTTTCACGAATTTAAAAGCATCTAATTTTTCGTCTGGCCCACTTTGATTTTGCTTAATAAAGTTTAATGCCCTTGTTGAGGTGGTATCCTA
TTAGGCCTTTTGTTTAAATTGTATGGTGTTTCTAATGCAGGCAATGAAGGCAAGAAAGCGGTTATTTGATAGTGAAATTTCAGCAGAAAATATACTGGTA
TGAGAGCCCATGATCAAAAAGGCTAATTTTTTAAGATGGAACAACTCTTCATGAGTGAGAAGTAGGAGCTCAATCTCTTCATTAGTAGTTTGAAACTTGA
AAGCTATGGTTAATGAGACTTTCTTTAGTTCGTTATTATTGTTGTTTGTTAAATTAAAATATATTTTCTGAATTCATTGAGCTCCCCTAGTTCTAAAATA
TGCATTAATTTTTTTAAGGGCAAAAGGAA

>Glyma.10G267400 | Chr10:49041854..49048547 forward
TTTTCATCTCTCATCGTATTCTTCTTCTATGGCTCTCACTCTCGGATGCATCAACTTTCCCTGTCATCAACAAAAATCAATCTCAAACCAAAACTTCCCT
AATCTTCTCCTATGATTTACTCTTCTTCTTTATCTCATCTTTTCTCCTAACCTTCTTTTTTTTTTTTTAGTTTATTATGTGAATTATGCCAGGGAAAATA
TATCTCTATACAAAATTTTGTTCTGGTTGGGAAGAAGGAACCCTACCCATCTCCATATTCAAGGAAGTAAGTTATTATTTATTTTGATTAAACTATATTG
TTGAAATTGAAAATTCTACGTATCTCTAATTTTAATTAAAAGGAAAATTTCTAAAATAATTGAATGCTGTTTTTTCTTTCTGTATAATGCTGGTATGGTC
CTACTTTTGGTCTTTTCCTGTGTGGACTTGGGACTAATGTGTTAGCAGCCTTGCTTTAATAGTGTTGTGAATTGTAATCAGAGTATAATGTTCTTTTTTA
ATGATTTAATTTAATTATACTCGCAAGATATCAGTGTTGATATTGTATTAACTACATATGATGTTCTTAAAAAGAATTTGTCCCTTGATGCATGATATTT
TCTGAGATTTTAGAAAAGGTTTGATTTAAGGAGTGCCATATTTCCCCCATTGTTATTATATCATATGTAAGATATTTTACCTCTACATCTGAGAATGGCA
AAATGAGTTTATACGAGTTTCACTTTCACCATATTTTTTCTTCATCAAATTTTGATTTTGTAACTTTTTGAGGTGTTATATTTTCTTTATGATTTGTAAT
TTTTTTAAACCTTTACAATAAACCATGCATATTATGTATTAGAAAACAAAATTCGATGGGAAATATTTGAGAACCACTCCTCCCCCGAATAGCTTGAAAG
GCTCTGATATCGTGATTTGGAAGGGGTCTAGTGATGGGAAATTTACTGGATAATAATGCTCTGTGCAATGATCCATTGTATTCTGCCATCTGGAATTGAA
GAGGTCCAGAGAGGGTGTGTGTTTTACTCTGGAAAATTGGAGTTAATGCGCTTTTGACAAATGAGAATAAGTGCTCCTGCCACCTAACTTTAGATGCTGC
TTGCTGAAGGTGTGGTGCTGATTCAGAAAATAATGACCATGGCTTTAGACACTGCCCCATTATTCGAGCCATATGGTACTTGATTCTTCCTCATTCTCTG
CACTCAAACTTCTTTCTTTAGGAGTTTAAGAGTAGACTTCTTGAGAATGTCACTAGTAACTTGATGGTTAATGGCAGAAATTGGTGTATGATTTTTGCTG
TGGCTTTGAACTACATTTGGAGATATAGAAACAAAGGAATCTTTGATAATAATTCCTTGTTACAGCTGATTGATATTGCAAATCAGATTTTGCTCCAGGT
TGATGCTATCTCAGTGAGTTTGCAGTGCCAATAGAGTTTGGTTCCTCAGATTGTTAATCCTGTGACTAGCTCTAGCATTCAGTGGTTCCCTCCTCCAACA
AATCAGTTCAAATTGAACTGTGATCCAGCTGTTCATACAGCTTCGAGAAAGCCAGCAGCTGGAGGAGTCCTTCGTGATCACACTGGATTGGTTTTATTCC
CCTTCGTTGCGGACATTGGTTGCTGTTCTGTTTTGGCAGCCGAACTTTGGGCAATTTTTCTTGGCTTGCAGCTTGCTTGGAGTCGTGGATTCAAGGAGTT
CATAGTTGAGTCAGCTTGCTGGTCATTGAGCTTATTAAGGAGTGTCCCGATACTCATCCTTGTTTCATGTTGATCAAGAACATTAATGAATTTGAGGTCA
AAGGAGGAGTTTTTGTCTGGTCTCACGCGCTGTGAGAGGGCAACCAGGTCGCAGACAAGCTTGCTGGCTTTGATTTATCTTTAGATGGCCGCTGTTCGAT
TTTTGACTTTGTTCCGTTTTTTTTTATGAAATGTTGTTGTAGCAGATATATCCTCAACCGTGTTCCCTTGTGGTTTTTAGTTGTATTGTTTGGGGCTTAG
GCCCCTATATTCCACAAAAAAAAAAAGATCTCTGTATGTTGCATTTGCTTAAATTTAAGAAAACTCTTCCAGAAAGAACTCTATTAAATGCTTTTGTGCT
TAACAAAAACATACCGATCATCAATAGTAATTTCAATGCTTCTGATTTTTGTTCAGATATTTGAATGATAATGTAGTTATCATATTTATATCTATAAATT
AAACTAGCCATCTAGCTTTTGAGAGCTTCAATCTCTACTTAGTACTCTCTTTATGATTTCTCATTTGAATTCTTATCTCTTATGCAATTCTGTACTTCAC
ACACTTGCTTTGCAAAGTTGCGTCACTCTCTCTGTGAAGAAATTCTGAAGTTGCTTCACTCTCTGTGAAGAAATTACACTTACTCTACGAAAAAATTACC
AACGCCACTTTTTTCCTTCTCCTTCAAACAAATTTTCTCAATGTTCACTCATACCCTTATTCTTCAACTGAAAATCTCCCAGCTCTCCTAATCATCTTCC
CTTATCTTCCTTAAATTGTGGATTTTGCCTTTTTTTATGTCAGTTTTCTCTTACTTCTGAAATCTTCAACTGCCTCTACTCTAAGGTACATTACACATTC
ACCATTCCATGCCCTCTTTCTTTCTTTGCTCTTCTATTATTTTCTCTCATGTTTTGTTTCCTTTTTTGATTTTGGGTTTTGATAACAACACCAATAGCTA
ATAGAGACATGCGTTTATTTTTGGCTCCAATATGTTATATCACCAATGTTTCACTCTCTCTCTCACTAATTTTTATTTAAAGTCAGTGTTACTTTTTCTT
TTCTTCAAAATCAAAAGCAAAACTTCAAACATATGTTCCTTTTCATGTTTCACACTCTGTCTCGCGTTTCAGTGTTTGCCAGTGTGTATTTCCAGAAGAA
TTTATGTTGTAATTTTGTATTTGAAGAAGAAAATCAATCTTTGATTATACTCTACTGCTCTATTTCAGATTTGTTTTGCTTTTAAAACTTTTGTGTATAG
GTTTAATGGTAAATTTTTATTGCATCATAGTATTTCTTAGATGCATTTAGTCTTTACTGTTATTGCTTATGTGGTTATATGTTACAGATTTGAATTGGTT
TATTTTGAAATTGATTTTAAAAATTCTTGGATTTGAAATTTGACTCATGCATATTCAAACTCCCAAGTTTCTCAATTTTGCAGAATGCTTTGAGAACTGT
GTTATTGTGGAAGCTTGATAATGTAGAAAAGAATGACAAATGCCAAATAACAATGATATCCACATGGGTAACTGACTTATACTTGGACAAGGGTTGACTA
CATTCTTTAATCTTAAACACTTGGAAGGAAAATATAGTTTCCCACTTCTTCCATTATCTCATTTTCTATTACATCGGCATGATGAAACCTTGTATATTAT
ACTCTAAAATTTAGAATCATTCTTAATTTGGTATAATTATACTAAATATTTTACTGTTCGATATGGGAAATGCACTGTATCACGGATAACTGCAAAACGA
TGAGGAAGTAAAGGGGTTTGGAAAACAAATACTCTTTTGTCACATTCTTTTCTGTGTGCTATAAGAGCAAATAAAGTGATATAAAACAGAAAATTTTATT
GTGTTGGTTGCTAATGCACTCATACTAAAAAAAGAGGTGTGAGTGCATTAACATATATATATATATATAATTTCTAAAGTAGTTTTTACATTTTATTGTC
AATCAAGGATTTACATACTGATTGCTCATTATCTTATTTTTCTCTATCCAGTCTTCCTCAACACATGGTGGTTCATAAATTCACTAAAAAGAGAAAGAAT
GTGGTCATAGTATATGATTGATTGAGAGGCAGAGACAATCTTTCATATTAGCAACTCAAATCTTTCACGCACTCTATCTCTCGATTGGACTCGTCGTACT
AGTAGGATAGAAAACCATGGAAGAGAATCTATTAGCAAAACAAAGAGAGAAGCAGAAAGTTACATGGGATGGTTTGGGTGAAGAAATGAAGAGGATCATT
TGCATAGCAGTGCCTATGGTAATTGTGACTGCTACCCAGTATTTATTGCAGGTTGTGTCAATCATGATGGTTGGTCACCTCAATAACAATCTCTATCTCT
CTGGCGCCGCCTTAGCCATCTCTCTTGCCACCGTTACTGGTTTCAGTGTTCTTGTAAGCTAAACTCAATATTGTACTGTTTGACTTTGATTCCTCATAGT
TATATGCTTTTTCTTGCATCCCATTCCTTAATATCAATCTACATCAAGACCATAATGTCCCACACAACAACAATATTAAATATTAATAGGGTAAACCACT
GTTTTAGTCCTTGAAAGTGTATGGGGCTGTCACATTAGTCTCTGAAATTAAGAAAATTTTAAATAAATCTCTAAAAGTAATATTTATCAATCACATCAGT
CCAAAATTAAAAAAAAAATGTGACTTAAGTAATAGTATTAACATATATTAAGGGTCAAAATGACAATAAGTTGTTAAACTTAGAGACTTAAACAACTGAA
AATTTTAGGAACTTATTTGAAATTTCCTTAGTTTTAAAGACTAATGTGACAACAAATAGTAGTTATATGCTTTTAGTGACCAAAATAGTAGTTTACCATA
TTACTTTTTGTTATCTAATTAGAACTAAATTCTTGCATTATTTACAAGTACGAATTCAATGATGATTAAAGGTATTAAAAAGCAATGAGTCAACATGAAT
GAAATCGAATGCTTATGTTTAGAGTGATTAAGTTTATATTCTTTATCAACTAATTCTAACATGTTAGCTTGCTCAAAATCATCACGATGGTATTCCCCAA
GTAACATAGTGCATTCGTAATTTGCACATGCTAACAGGCAGGAATGGCTAGTGGACTGGAAACTATTTGTGGACAGGCTTATGGAGCTCAGCAATATGAA
AAAGTTGGAGTGCAAACATACACTGCTATATTCTCTCTCACAGTCGTTTGTCTTCCCCTAACTTTCATTTGGATCAGCATGGAAAAGATCCTAGTTTTCA
TAGGCCAGGACCCTCTAATTGCACAAGAAGCAGGAAAATTCTTAATCTGGCTTGTTCCCGCACTTTTTGCACATGCAATTATGCAGCCGTTTGTTCGATA
TTTTCAAATGCAAAGTCTGCTTCTTCCCATGCTTATAAGTTCTTGTGTCACTCTTTGTATCCATATACCCCTTTGTTGGGCTTTGGTGTTCCAGACTGGA
ATGAATAATATTGGTGGAGCATTAGCAATGAGCATTTCAATATGGTTAAATGTGACTTTTCTTGGATTATACATGAGATACTCTCCTGCTTGTGCAAAAA
CCCGTGCACCCATTTCTATGGAGCTGTTCCAAGGAATTTGGGAGTTCTTTCGCTTTGCTATCCCTTCTGCAGTGATGATTTGGTGATTCATCTTCACTTT
CACTTCAAGTTGTATATATATTCCTTTTTTCCAATTATAAATTGTTTATTTTTCCAGAAATATCTGATCTAATGTTTTTTTTTTCATGTAATGTTCATTT
TAGCCTTGAATGGTGGTCGTTTGAGCTGCTTATCTTGCTGTCTGGGCTGCTACCGAATCCACAACTTGAAACATCAGTTCTATCCATTTGGTATGTTTGC
TATATTTTCTCTTCTTTTCTACATAACATACTATACTTTTAAGGTTGTCCTTGAACGACAACTCACAATCTAACATTTTTAAATTAACGTGATTGTGCTA
ATTCATTGTCATTTTAATACAGTCTCAACACCATTAGCACTCTCTTTTCAATACCTTTTGGAATTGCTGCTGCTGCAAGGTGAATAATAAACATGTTATT
TCTAAATATTATACATGCCACAATCTATATACACCTAGCAACCTTTGGACGTATATCTAAACTGATTATCTTTTCCCCTCTCTGATTGTGTAGCACAAGG
ATTTCAAATGAATTAGGAGCAGGGAATCCACATGCTGCCCATGTTGCTGTGTTGGCTGCAATGTCTTTTGCAATCATGGAGACAGCTATAGTTAGCGGAA
CCCTCTTTGTCTGTCGCCATGATTTTGGTTATATTTTCAGCAATGAGAAGGAAGTTGTTGATTATGTCACTGTCATGGCTCCTCTGATTTGCATATCTGT
TATACTGGACAGCATACAAGGCGTTCTCGCTGGTAAAATTCTGTCTTCTCTATTGTTAGGTAAATTTTATATAGAAGAGAAACGTTGTCTCAATTTGTTT
TATAATACTTCGGCTCAGGGGTTGCTAGAGGTTGTGGATGGCAACACATAGGGGTTTATGTGAATCTAGGGGCATTCTATCTTTGTGGGATTCCTGTTGC
TGCCACACTGGCATTTTTGGCTAAAATGAGAGGAAAAGGACTTTGGATTGGTGTACAAGTCGGTGCTTTTGTTCAATGTATTCTATTTTCTACCATAACA
AGTTGCATAAATTGGGAACAACAGGTATATATGTCCTCTAACCTTTGTCATAAAACCCCAAAATTTAATATTCTTGTTTAACAAATTTAAAAGGACAAGT
ATATAATTCTTTGCCTGGCTACCACTTTAGGCATTTTGTTGAATTGTGTGGTCTTACTGAATACCAATGCAGGCTATCAAGGCAAGAAAAAGGTTATTTG
ATAGCGAAATTTCAGCAGACAATAGACTCGTATAAGAAACCCATGATCAAAGGCCGATTTTCTAATGACATGAACAACTCTCCATGAATGAGAA

>Glyma.10G267600 | Chr10:49049665..49051372 forward
TCACTTCCAAAGGTCCTAAGTTTCTAATTTCTAGTGAAACTAAAACAAGAAAAATCCACAAGAAAACATTGGTCTCATTCTCTACCCCGCTAAGCCTTAT
TATTGCAGTAGCCCTGAATCGTGAGAACTGCTGCTTCTCCGACAACCGTGGCACTGCCGCCGTGAGAGAGGAGATATGTTTCTGCTTCTTCGGTGACGAC
TGGCGCCTGTAACTCGTCTCCTTCCTTCATCTGTCTCTTTCAAACGAGCCTATCACTTATTTCCGTCGTTTATACTTTATACCCGACCATAGCCATATAT
AGGGAGACTCATATCTCTCACACTGTGTTGGGTTAGTAGGTCAGAAAAAACATGCAAGATAATCTATTATTGTCATCACCAAAACAAGGTGCAACAGAGA
GTTACGTAGGATGGTTATGCAGAAGAAATGAAGAGGATCATTTCAGGCCCTCCTTGAACTGGAGCTCATAATCTAACAATCTTGTGAGATTTTGTAAATT
AACATGATTGTGCTAATCCTTTGTCATTTTTCTACAGTCTCAACACCATTGCAACTCTCTTTGCAATACCTCTTGGAATTTCTTCTGCAGCTAGATGACA
CCATAAACATGTTCTTTCTAAATACTTTACATGTTACAATTTATATAGCTAGCTAGCTACCTTTGGACGTATAGGTATATCTAAACCAATTATCTTTTTT
CCTCTCTAATTATAGCACAAGAATTTTAAATGAATTAGGAGCAGGCAATCCACATGCTGCCCGTGTTGCTGGGTTGGTCTCAATGTCTTTTACAATCACG
GAGGCAACTATAGTTAGCGGAACCCTCTTTGCCTGTCGCCATGTTTTTGGTTATATTTTCAGCAATGAGAAGAAAGTTGTAGATTATGTCACTGTCATGG
CTCCTCTAATTTGCATATCTGTTATACTGGACAGCATACAAGGTGTTCTCGCAGGTAAACTTCCCTCTTATCTATTTATTGTTGACTAAATTTTATATAG
AATTTGTTTAACAATACTTTGGCTCAGGGGTTGCTAGAGGTTGTGGATGGCAACACATAGGGGTTTATGTCAATCTAGTGGCCTACTATCTCTGTGGGAT
TCCTGTTGCTGCCTCTCTGGCATTTTTGGAAAAAATGAGAGGAAAAGGACTTTGGATTGGTGTACAAGTCGGTGCTTTTGTTCAATGTGTTCTACTTTCT
ATCATAACAAGTTGCATAAATTGGGAACAGCAGGTATATATATGCTCTCTAATCTTTGTCATAATTAACCTCCAAACTTAAATTTGGTTATTTAGTTTAC
TACTTTTAACGAATTTAAAGTAGCTAATTCTTAGTCTTGCTACCATTTTGATTTTGCTTAATAAAGTTGAATGCCCTGTTGAGGAGGTATCCGGTTAGGC
CTTTTGTTTCAATTGTGGTGTTATTAATGCAGGCAATCAAGGCAAGAAAGAGGTTATTTGATAGTGAATTTCCAGCGGACAATAGACTGGTATGAGAAGT
AGAAGAGCCTCTTTATTGGCAGCTTGAAAGTAATGTTTGATGAGATTTTGTTTTTTTTTTCTTTAGCTCATTATTGTTTTTTTTTTCTTTAGCTCATTAT
TGTTTCTTTTTTTTTTGTCATTATTATGGTTTCTTGTTAAATTAAAAACGTATTTTTCGAATATATTTACCTCCTCTAATTCTAATATATGCATAATTTT
TTGGGGAC

>Glyma.05G054000 | Chr05:4903827..4905404 reverse
ATGTCAGCAACATCTAAAGAAGGAGAGAACTTAAGCACAAACGACAGGGCCAACCGACACCCCTCAACTCCTTCATCGCACACTAAAAAATGCCCTTTGA
ATGCCAATTTTGTGAAGATGGTGGTAGAAGAGCTAAGAGTCCAACGAGGGATAGCCCTTCCACTTGTGGCCATGAACTTGGCTTGGTTCGCCAAGTTAGC
CATAACAACAGCTTTTCTCGGCCACCTTGGGGAGCTGAACTTAGCCGGTGGCGCACTCGGGTTCAGTTTTGCTAATGTCTCCGGCTTCGCTGTCTTGAAC
GGTCTAAGCGGCGCCATGGAACCCATTTGTGGACAAGCACATGGAGCCAAGAACGTGAGGCTCCTTCACAAGACCCTTCTCATGACAACCCTCTTGTTGC
TTTTGGTAACACTTCCCTTATCTTTCTTGTGGCTTAACGTTGACAAGATTTTGATTCTTTTTGGCCAACAACAAGAGATCTCAATAGTGGCCAAAACCTA
TGTTTCCTATCTCATACCTGACTTGTTTATCAAGGCACTCCTTTGTCCCTTAAAAGCCTACCTCAGCTCTCAGTGCATGACTCTTCCCACCATGTTTAGT
TCTGCTGTGGCACTAGCCTTTCACATACCCGTTAACATACTACTCTCCAAAACCATGGGACTCCGAGGAGTTTCAATCGCGGTTTGGGTAACCGACCTCA
TTGTTATGGTTATGCTTGCAATTTATGTTGTGGTTCTTGAGAGAAGAAATGGGAGCATGTTGTGGAAGGAAGGAGGGTGGTGGGATCAGAACATGATGGA
TTGGAGTAGGCTAATCAAGCTTAGTGGATCATGCTGCCTCAACACGTGCCTTGAGTGGTGGTGTTATGAGATTCTACTTTTCCTCACTGGCCACTTGGCA
AATGCAAAACAAGCAGTGGGGGTTCTGGCCATAGTGTTAAACTTTGACTATTTGCTTTATGCAGTGATGCTTTCTCTAGCCACTAGTGTTTCCACTCGTG
TGTCCAATGAACTTGGTGCTAACCGTGCTGGTCAAGCTTATAAATCAGCACGTGTGTCTTTGGCAGTGAGTGTTATATCGGGTTGCATTGGAGGTTCCAT
CATGGTGGCTGCTAGAGGGGTTTGGGGGAATTTGTTTAGTCATCAAAAAGGGGTTGTAAAGGGTGTGGAGAAGGCAATGTTGCTGATGGCTCTGGTCGAA
GTGTTTAATTTTCCTGTGACGGTTTGTGGAGGCATAGTACGAGGAACTGGACGACCCTGGCTAAGCATGTATGCTAGTCTTGGTGGGTTCTATTTCCTGA
CACTACCATTGGGTGTGGTTTTTGCCTTCAAGCTTCGGTTTGGACTTGGTGGACTCCTTATAGGACTCCTGATTGGTATTGCTGCTTGCTTGGTTCTGTT
GTTAACATTTATTGTGAGAATAAATTGGGTGGAAGAAGCTACCAAGGCACAAACGTTTGTATGTAACGCACAGGTACATGAACAAGTTTCAAGAAATGAG
GTAAATGAACTTACTGAAAACCCTGAAGGTGATCAAGTGTGCAAAGAAACCTCTATCACCCCATATTCATGTATATAA

>Glyma.17G136400 | Chr17:11018767..11020308 reverse
ATGTCAGCAACATCTAAAGGAGAGAGCATAACCGCAAACGACTGGTCCAACCGGCAACCCTCATTTCCTTCATCACACACCCAAAAATGCCCTTCCAATG
CCAACAATTTTGTGAAGATGGTGGTAGAAGAGCTAAGAGTCCAACGAGGGATAGCCCTTCCACTTGTGCCCATGAACTTGGCTTGGTTTGCCAAGTTAGC
CATAACAACAGCTTTTCTTGGCCACCTTGGGGAGCTGAACTTAGCCGGTGGCGCGCTCGGGTTCAGTTTTGCTAACGTCACCGGCTTCTCTGTCTTGAAC
GGTCTAAGCGGCGCCATGGAACCAATCTGTGGACAAGCACATGGAGCCAAGAACGCGAGGCTTCTCCACAAGACCCTTCTCATGACAACCCTCTTGTTGC
TTTTGGTAACACTTCCCTTATCTTTCTTGTGGCTTAACCTTGGCAAGATTTTGATTCTTTTTGGCCAACAACAAGAGATCTCAACCGTTGCCAAAACCTA
TGTTTCCAATCTCATACCTGACTTGTTTATCAAGGCACTCCTTTGTCCCTTAAAAGCCTACCTCAGCTCTCATTGCGTGACTCTTCCCACCATGTTTAGT
TCTGCAGTGGCACTAGCCTTTCACATACCCGTCAACATAGTACTCTCCAAAACCATGGGACTCCGAGGAGTTGCAATTGCGGTTTGGATAACCGATCTCA
TGGTTATGGTTATGCTGGCGATTTATGTTGTGGTTCTTGAGAGAAGAAGTGAGGGCCTGTTGTGGAAGGAAGGAGGGTGGTGGGATCAGAACATGATGGA
TTGGATTAGGCTAATCAAGCTTAGTGGATCATGCTGCCTCAACACGTGCCTTGAGTGGTGGTGTTATGAGATTCTACTTTTCCTTACTGGCCACCTAGCA
AATGCAAAACAAGCAGTGGGGGTTCTAGCCATAGTGTAGAACTTTGACTATTTGCATTACGCAGTGATGATTTCTCTAGCCACTAGTGTTTCCACTCGTG
TGTCCAATGAGCTTGGTGCTAACCGTGCCGGTCAAGCTTGTGAATCAGCACGTGTGTCTTTGGCACTGAGTGTTGTATCTGGTTGTATCGGTGGTTCCAC
GATGGTGGCTGCAAGAGGGGTTTGGGGGGATTTGTTTAGTCATGATAAAGGGGTTGTAAAGGGTGTGAAGAAGGCAATGTTGTTGATGGCTCTGGTGGAA
GTGTTTAATTTTCCTGTGACGGTTTGTGGAGGCATAGTACGAGGAACTGGACGACCCCGGCTAGGCATGTATGCTAGTCTTGGTGGGTTCTATTTCCTGA
CACTGCCGTTGGGTGTGGTTTTTGCCTTCAAGCTTCGGTTAGGACTTGCTGGATTCACTATAGGACTCTTGATTGGTATCGTTGCTTGCTTGATTTTGTT
GTTGACGTTTATTGTGAGAATAAATTGGGTGCAAGAAGCTACCAAGGCACAAACGTTTGTATGTATCGCACAGGTACAGGAACAAGTTCCAAGATATGAG
GTAAATGAACTTGTTGAAAACCATGAGAATGATCAAGTGTGA

>Glyma.05G054100 | Chr05:4910436..4911476 forward
ATGAACTTGGCTAGGTTCGCCAAGACAGCCATAACAACAGCTTTTCTTGGCCACCTTGGGGAGCTGAACTTAGCCGGTGGCGCACTCGGGTTCAGTTTTG
CTAATGTCACTGGCTTCTCTGTCTTGAACGGTCTAAGAGGCGCCATGGAAACCAAGAACGTGCGGCTCCTTCACAAGACCCTTCTCATGACAACCCTCTT
GTTGCTTTTGGTAACACTTCCCTTGTCTTTCTTGTGGCTTAAGGTTGACAAGATTTTGATCCGTTTTGGCCAACAACAAGAGATCTCAATAGTGGCCAAA
ACATATGTTTCCTATCTCACACCTGACTTGTTGGTCACTTCACTCCTTTGTCCCTTAAAAGCCTACCTCAGCTCTCAGTGCATGACTCTTCCCACCATGT
TTAGTTCTGCTGTGACACTAGCCTTTCACATACCCGTCAACATACTACTCTCCAAGACCATGCGACTCCGAGGTGTTTCAATTGCGGTTTGGATCAACGA
TCTCATGGTTATGGTTATGCTGGCGATATATGTTGTGATTCTTGAGAGAAGAAACGGGAGCATGTTGTGGAAGGAAGGAGGGTGGTGGGATCAGAACATG
ATGGATTGGATTAGGCTAATCAAGCTTAGTGGATCATGCTGCCTCAACACGTGCCTTGAGTGGTGGTGTTATGAGATTCTAGTCTTGCTCACTGGCCACT
TGGCAAATGCAAAACAAGCAGTGGGGGTTCTAGCCTTAGTGTTGAACTTTGACTATTTGCTTTACTCGGTGATGCTTTCTTTAGCCACTTGTGTTTCCAC
TCGTAGTGCTGGTTAAGCTTATAAATCAGCACGTGTGCCTTTGGCAGTGAGTGTTATATCGGTTTGCATTGGAGGTTCCATGATGGTGGCTGCTAGAAGG
GTTTGAGGGAATTTGTTTAGCCATGTTAAAGGGGTTGTAAAGGGTTTGAAGAAGACAATGTTGGTGATTGCTCTAGTGGAAGTGTTTAATTTTCCTGTGA
CGGTTTGTGGAGGAATGGTGCGAGGGACTGCAAGACCATGA

>Glyma.17G136500 | Chr17:11025191..11027406 forward
GATTGGTTGCTTAACCAATCTTATATAAAACAAAAGAAACACGCTAGTGAAACTTCACAACACAACAATCATACCATAACATTATCTCAGCAAAAATGTC
AGCAACATCTAAACAAGTAGAGAACCTAAGCACAAACGATAAGGCTAACCATCGGCCCTCATCTCCTTCATCACACACTAAAAAATGCCCTTTCAATGCA
AATTTCATAAAGATGGTACTAGAAGAGCTAAGAGTTCAACGAGGGATAGCCCTTCCTCTTGTGGCCATGAACTTGGCTTGGTTTGCCAAGACAGCCATAA
CAACAGCTTTTCTTGGCCACCTTGGGGAGCTGAACTTAGCCGGTGGCGCTCTCGGGTTCAGTTTTGCTAATATCACCGGCTTCTCCGTCTTGAACGGTCT
AAGCGGCGCCATGGAACCAATCTGTGGGCAAGCGCATGGAGCCAAGAACGTGAGGCTCCTCCACAAGACCCTTCTCATGACAACCCTCTTGTTGCTTTTG
GCATCACTCCCCATATCTTTCATGTGGTTGAACGTTGACAAGATTTTGATTTGTTTTGGCCAACAACAAGAGATCTCAACCGTGGCCAAAACCTATGTTT
CCTATCTCATACCTGACTTGTTGGTCACTTCACTCCTTTGTCCCTTAAAAACCTACCTCAGCTCTCAGTGCATGACTCTTCCCACCATGTTTAGTTCTGC
TGTGGCACTAGCCTTTCACATACCCGTTAACATAGTACTCTCCAAAACCATGGGACTCCGAGGAGTTTCGATTGCGGTTTGGATAACCGATCTCATGGTT
ATGGTTATGCTGGCGGTTTATGTTGTGGTTCTTGAGAGAAGAAATGAGGGCATGTTGTGGAAGGAAGGAGGGTGGTGGGATCAGAATGTGATGGATTGGA
TTAGGCTAATGAAGCTTAGTGGGTCATGCTGCCTCAACACGTGCCTTGAGTGGTGGTGTTACGAGATTCTAGTTTTGCTCACTGGCCACTTGGCAAACGC
AAAACAAGCCCTGGGGGTTCTGGCCATTGTGTTGAACTTTGACTATTTGCTTTACTCAGTGATGCTTTCTCTAGCCACGTGTGTTTCCACTCGTGTGTCC
AATGAGCTTGGTGCTAATAGTGCTGGTCAAGCTTATAAATCAGCACGTGTGTCTTTGGCAGTGGGTGTTATATCGGGTTGTATCGGTGGTTCCATGATGG
TGGCTTCTAGAGGGGTTTGGGGGAATTTGTTTAGCCATGATAAAGGGGTTGTAAAGGGTGTGAAGAAGACAATGTTTTTGATGGCTCTGGTGGAAGTGTT
TAATTTTCCTGTGACGGTTTGTGGAGGCATAGTGCGAGGGACTGCAAGACCATGGCTAGGAATGTATGCCAATATTGGTGGATTCTACTTCTTGGCCTTA
CCATTGGGTGTGGTTTTTGCCTTCAAGCTTCGTTTAGGACTTGCTGGACTCATTATAGGATTCTTGATTGGTGTTGTTGCATGCTTGATTTTGTTGTTAA
CATTTATTGTGAGAATAAATTGGGTGCAAGAAGCTACCAAGGCACAAATGTTAGTATGTATTCCCGCACAGGTACAGGAACAAGTTCCAAGAAATCAGGT
AAATGAACTTGTACGTTGAAAGAAACCTCTATGGCTCTATCACCCCAAATTCATATATAACAACACTTGGGGTGAGAAGAATTAGACATGTACAATATGC
TAAGTCCCTTCTCAATCTCACTAGTATTTGTGATCTCGGGTGTTTTCTGAATCATATCATTGCTAGTTGTAATTGTAGTAACGGATGCAGTGGAACTTCA
GATTTGAAAATTAGAAGATTAATGCATTTTAAATCAATAGGTAGAACAAGACGTTGGTCTACGTACTAATGTTATAATTGTGTCAAATGATCATAATGCA
ATCAAGGCAAAACTATTCTATTCATACATCTAAAAGCAAAGCATCAATTACCCATCATCAGAGATTCAGAAATTTAGGAGTACTGCAGCTCTTGTGTTTC
TTAAATCTCAATATCAAAGCCCCGTTCGTTTACTGAGAAAGATAACGGAAAAGAGAAGAAGTAAACAGTAAAGCTTGTAATCAGGGGATTCTGCTTCAAA
CCAAATGCCAAGAAGTATATGGGTTGTCCCTTCCATCATTTGCAAGGCATAGATGAAATTTATATTACGAAGTAATTATTTATGAAATTAAGACCACTAA
CCAATCTAAAGCAAAA

>Glyma.01G213000 | Chr01:54426718..54428490 reverse
ATGTCAGCAACCTTAACATGTAAACTAGGAGACAACAACCTAAGCTTAAACGATAAAAACGACCAACCCCCTTCACCTCCTCAGCCCCTACAAAAATGCC
CTGCCAATATGGTTTCGATGGTGGTCTCAGAGCTAAGAATTCAACGAGGAATAGCCCTTCCAATGGTGGCCATGAATTTGGCTTGGTTTGCCAAGACAGC
CATCACAACAGCATTTTTAGGCCGTCTTGGGGAGCTTAGTTTAGCAGGTGGAGCTCTCGGCTTCACTTTTGCTAATGTCACTGGCTTCTCTGTCCTCAAT
GGTCTATGTGGTGCCATGGAACCCATATGTGGACAGGCTCATGGTGCCAAAAACTTCAGGCTCCTTCACAAGACCCTTCTCATGGCAATCTCGTTGTTGC
TATTGGTATCACTTCCCATCACTTTCTTGTGGCTTAATGTTGACAAGATTTTGATCCTTTTTGGCCAGCAACAAGACATCTCCACTGTTGCCAGGACCTA
TGTTTCATGTCTCATTCCTGACTTGTTTGTTGCCTCACTGTTCTGTCCCTTAAAAGCCTACTTGAGTTGTCAGAGCATAACTCTTCCCACCATGTTTAGT
TCTGCTGTGGCACTAGCCTTCCACATACCAATTAACATAGTGCTCTCAAGGACCATGGGCCTCAGAGGAGTTTCCATGGCAGTTTGGATAACTGATCTTA
TTGTCGTGGTTTTGCTAGCCATTTATGTTTTAATTCTTGAGAACAAAAAGGAAAGCATGTGGAAGGAAGGAGGGTGGTGGGATCAGAGCATTGAAGATTG
GATAAGGCTGCTCAAGCTTTGTGGATCATGCTGCCTCAACACATGCCTTGAGTGGTGGTGTTATGAGATTCTAGTTTTACTTACTGGCCACCTCACAAAT
GCTAAGCAAGCAGTGGGAGTTTTGGCCATTGTGCTAAACTTCGACTATTTGCTTTTCTCAGTGATGCTGTCACTAGCCACTTGTGTTTCCACACGTGTCT
CAAATGAGCTTGGTGCGAACCAAGCTGGTCTTGCTTACCGATCAGCATGTGTGTCTCTGGCATTGGGCTTTATCTCTGGTTGCATAGGTAGCTTGGTGAT
GGTGGCTGCCAGGGGAATTTGGGGGCCACTGTTTAGCCATGATATGGCAATTATAAAGGGAGTAAAGAAGACAATGTTGCTGATGGCTCTGGTTGAAGTG
TTTAATTTTCCGCTGGCAGTTTGTGGAGGCATAGTTCGAGGGACAGCACGACCCTGGTTGGGCATGTACGCGAATCTAGGTGGATTTTATTTCCTTGCTC
TGCCACTTGGTGTTGTTTTTGCCTTCAAGCTCTGTCTTGGGCTTGTTGGACTCCTCATTGGACTTCTGACTGGTATTGTTACCTGCTTGACGCTGTTATT
GGTATTTATTGCGCGGTTAAATTGGGTGGAGGAAGCTGCCAAGGCACAAACACTAACAGGCCAAGAGCAGGTTAAGGAACTTTCCAAATATGACGCAGAA
GAACGAATTGACGCCCATGAAAAAGATGTAGTGTAAAATGAATATTAGAAATTATTATCATCTCCATCTAACCAAGTTCATACGTATCTCCAAGCACCAG
GTCACGATGAAAAAGACATGGCTTAACCGTGTGCACAAATTGAAAGCGGGAGAAAGGTCATACATACAATAGCTTAATTGCCTGTCATCTGTCAAACCCA
TTTTTAGAGTTTCAGTGCTCAAGGACTTTTCCTGAAAATCACTTAGAAGCAACCGACATGGTTTATCTGTCGT

>Glyma.11G028900 | Chr11:2090136..2091568 forward
ATGGGCTTCCGATCTGGTTTCGATGAGCTAAGAGTTCAACGAGGAATAGCCCTTCCAATGGTGGCCATGAATTTGGCTTGGTTTGCCAAGACAGCCATCA
CAACAGCATTTTTAGGCCGTCTTGGGGAGCTTAGTTTAGCAGGTGGAGCTCTCGGCTTCACTTTTGCTAATGTCACTGGCTTCTCTGTCCTCAATGGTCT
ATGTGGTGCCATGGAGCCCATATATGTGGACAGGCTCATGGTGCCAAAAACTTCAGACTCCTTCACAAGACTCTTCTCATGACAATCTCATTGTTGCTAT
TGGTATCACTTCCCATCACTTTCTTGTGGCTTAATGTTGACAAAATCTTGATCCTTTTTGGCCAACAACAAGACATCTCCACTGTGGCCAGGACCTATGT
TTCATGTCTCATACCTGACTTGTTTGTTGCCTCACTCTTCTGTCCCTTAAAAGCCTACTTGAGCTCTCAAACCATAACTCTTCCCACCATGTTTAGTTCT
GCTGTGGCACTAGCCTTCCACATACCAATTAACATAGTGCTCTCAAGGACTATGGGCCTCAGAGGAATTTCGATGGCAGTTTGGATAACTGATCTTATTG
TTGTTGTCTTGTTAGCCATTTATGTTTTAATTCTTGAGAGGAAAAAGGAAAGCATGTGGAAGGAAGGAGGGTGGTGGGATCAGAGCATTGAAGATTGGAT
AAGGCTGCTCAAGCTTTGTGGATCATGCTGCCTCAACACATGCCTTGAGTGGTGGTGCTATGAGATTCTAGTTTTGCTTACTGGCCACCTCACAAATGCT
AAGCAAGCAGTGGGAGTTTTGGCCATTGTGCTAAACTTCGACTACTTGCTTTTCTCAGTGATGCTGTCACTAGCCACTTGTGTTTGCACACGTGTCTCGA
ACGAGCTTGGTGCCAACCAAGCTGGCCTTGCTTACCGATCAGCGTGTGTGTCTCTGGCATTAGGTTTTATCTCTGGTTGCATAGGTAGCTTGGTGATGGT
TGCTGCGAGGGGAATTTGGGGGCCACTGTTTAGCCATGATGTGGGAATTATAAAGGGAGTAAAGAAGACAATGTTGCTGATGGGTCTGGTGGAAGTGTTT
AATTTTCCGTTGGCAGTTTGTGGAGGCATAGTTAGAGGGACAGCACGACCCTGGTTGGGAATGTACGCGAATCTAGGTGGATTTTATTTCCTGGCCCTAC
CACTTGGTGTTGTTTCTGCCTTCAAGCTCCGTCTTGGTCTTGTTGGACTCTTCATTGGACTTCTTACTGGTATTGTTACCTGCCTGACATTGTTATTGGT
ATTTATTGCGCGGTTAAATTGGGTGGAAGAAGCTGCCCAGGCACAAACACTAACAGGCCAAGAGCAGGTTAAGGAACTTTCAAAATATGACGCAGAAGAA
CTAATTGACGCCCATAAAAAAGATGTAGTGTAA

>Glyma.01G132200 | Chr01:44951519..44957755 forward
ATGTGCACTGGTGCTGAATCTCGAGGAGATATAAATGGTGACCCTGTGATTTCAAAGGTTGGAGGAGACTATGAGGATAGAATGAGAACCATAGAATTGG
AAGGTGGAGAAGAAACAGCTTGCAACTGGGGCACATTTGTTCGAGGGCTTCTTCAAGGCATACAGCATCATCTAACTGCCCTTAATCTCCCTAGTCTTTC
TGCTTCTGAGGTAGGTATCTTCTTTCTATCTCTGAAGTGAGTTTGGTTCTGCAAGCTTTTTTCTTTCTTGCTGGTTTTCTAGCTTCTTTTAGTTGAAAAC
CTCATCATACAAATTTAATAAAAGAAAATGATGTATGAACAATCCATGAAATAAACATTCAAAAGAGAGGTACAAGTGAGAAAACTGTAAATATATAGGC
GATATGGTGTAATAAGAGAGAAATAAAAAATAATAATAAATATTAACAAGATATTTACCCAATATATACAATTAAGGTCTCTTTTTTTAGTAGTCGTAGT
TACTCTCATACTTAATGGTAGGAAGGAAAGTTTTAAGAAACTGTATTGATATTTTATACAATTGTATTGTGAAGTAAAAAAAAAAAAAAATGATATGTTG
CAAAATAATAAGATTATGTTCTCGTGAAAGGAAGAAAATGCCGAGACATCTATGCAGACAATATTCTTGGAGCAAGTATTTCTTTTATTGGTTTGAGCTT
TTTGGCATTTTATAGTATAAGCCGCAGTTTCAGTTAGGCAACCGAAAATATGTTTAAAAAATATGTAGTTTTTATTATTTTTTTTGGAAAAGAACAGCTT
TTTAAGAAGAAATCAGAAAGTTTGTAACAAAAACTTTTGAACAGCTCAAAATAAGTTAATTAAGCACACACTTTTTTTAGTTTAATTTGAATCTTTTTAA
TCCCTTTAATTAATTTACAAATCAAAGTGATTATATATTAATTAAGGTTTCTTTGGAATTTGTTAAAAAAATACATTGAATTATGTTTAAAAGATATTTT
TAAATTCATTTAGGCGTGTATAAAGACATTTAAATTTCATAAAATAAAGTATATAATATATTAAAGGAAAATATTATCTTTTGATGACTTAAATAAGGAA
ATAGTTGTGACCATATCTTAAGTGACATTTTCTTATGTAAGGTATTTTAAGCTTCATTATCCATCATGTTGGGAGTTTTCCATTGTTCTCGATGCTGAAC
CCCTCTATTATCAATACTAAATTGATCTTTTACCCATCTAACATTTATCTACAGTACAGAGTACGGACATAAATTGTTTGCTTGGCAATTTAAATCTATT
GTGCTAATAATTGAGGCATGCACACGTGGATGACCCTCTTTTTTTCTTTTCTTCTATTCTTTTTGCTTAACTTAATCATTGAATATAGATCTGTACGTAG
TTTAAATAATTATTTATTAAATAATTATAAGTTAAATTATAAAATCATTAAAATAAATTAAAACTTGACAATTTTTCTTTACAATATATAGATATATAAA
TAAGTTTAGTAAAAATTTATAAATATTATAAATCACTTTTATAAATCTTAATAAACTCTTTCAAATATCATATATCTAAACCCTAAATAAAAGACATAAG
TCATTTGATGCTTTATTTGCTTTCTTTCCTATTCTTTCACTTAAAAATGGTACACTGGAACCGAATGGTTGATGATGTACGTACAAATTCACAATAGTTC
TTGCCCTAAAATATTGTCTTTTGAGTTGTTCATACCGAAAACAAAGAATAAATAGTCAAACACTTGCTTTGTAAGTATTGAAGTTTGGGCTACATATGTA
TATGGTCATTTTTCAACAATGACGACCAATTCATTTTGTTTGTTATATTACTAGTCACTTGTAGCATAAATTGCGTAATTCTCTGTACATAACCTTTGAT
TGTGTTCTTGACTTGGTTAAGTATAGATATTGGATTTGATTGTCTAATAGCATAAAGAATTTATCTTACTCTTTATATGAATGGTTAAGGGATCTCATGG
ATTGTTTGAAAGGATTATAGGGAGTCAACTTTTGGAAGGAAACACTACAGCTATACAATAATATATATAGTTGCTGTTAGCAGGAATATAATTGATATAC
TGATTTTGATTCGGATGAAAGATGGTGGACAATAATGCTGACTTTTATATATGGGCTGGGATAGTCCTTGTACTCATATTTAATATATTTATCAATTTTG
GCTAACAAAAAAAAAATGAGTACGAGAGAATTATCAAAGTACCAAAAATCTAAAAACAAAAGACGACAAATAAATTTCTTTATATATTTTTTAATTTTTA
AAATAAACAATGACATGTGTTTTTTTAAATTTCTAAATATACATTTACTAAAATTTATTTAATGTTGAAAAACCATTGACATATCATTATTTACTTTAAG
ATTTTGACGGTAAAAATACAATCAACATAATATAAATTTCATCTTAAGATAGTTTTTTTGGATAAACAATTAATAGGAAAAATTCATCTTCTTAGGATAT
GTATGAACACTTTGCTAGTTGCAGTTTTGTCCCTGCTATACTAAGAAGAACATGATAGTGTTCAAGAAGAACAGGGATATCATCTATCTTAGCTTTCTTA
TCATGCCATATATTTCAACATGGTATGGGAACTGAAAATCTGAAATATGTTACCATGACTTAGGTTATATTATGACCACCTGCTCTCTTTGCTCATCATT
GTTTGATCCTTGATTAAAACTGTGTTTGGGGTCAATATGATATAATTTGTATTCTTCCACTTCATTCAGATGAATCGAGACCGAAAATATGGAGGCTCTG
TCTCTTAGAAATCACTTGTGTCTGTTTCACACCACAAATCCTTTTTGCTATATTGCATGGCACTTTTCAGTGTCGTGTCCTGATCATTAATTGATCTTTA
TCATAACATTGCACAAAATTTGGTATCGAGTGAGAGTGAAAGATCATACATGTAACTACTAATCGCTATACATAATTAAGAATATCTTTAAGAATATCTA
TTGGTGTTAAATGAATTATGAATACGACCGTGATGTTCAGAAGTACGTCTATAAAGCATAATTCATTATTCAATAGAGTTTTGGGAAATAATTCTTAAAC
ACCCTTTGAATAGGAAGAGAAAAAATAATTCTTAAACACCTTTGAAGGAAGAGAAAAAAAAAATAGAAGTGCCGGTGAAATAATTTTTATTTATAGGTGT
TTAAATATTACAATTCTAGAATTTTATATATATTATGCAGAACCTCGTGATATATAATATAGATTATTTAAAAATATTATTCTTTTCAAAACATTAATAA
AAAATAATTATTTTTTTCATAATAACCTAAACTACAGTATACGCGCACGTCTGTGAGACATGCTCATAATCATAATACCGCAGTTTCTGTTGGCGGCTTG
TCCTAAACTAGAGCCTGTGTCTGTTAGATCTGAAAGCATTTTGACATTTTGTCATCTGTTTTTAGTTTTCACTTTTTTTTTCTTCCTTTTTCCTGCTACA
TCATTAAATATATAACACCGTCATATTTTTATACACCAACAGAATTATGACAGTAACTTTTCTTTTCAACCACTTGCCTAATGCCTATGCTTTTTCTTGC
TAGGAACCTGACTAATTAAATTGAAAAACTCACGAGCTTCAATTCCATGTTCCAACTTTTTTTTTTCTTTTCTATACTCGTTATTTTATCAATTCTATGT
CTTTACACTTTCTAAAAAAAAATATTTGTAACCTTTTTTTTTCTTTATTGAATAAAAATCGGAGAGAAAAGGATCATGTGAACCTTGTTTAGATGAGTGT
GTTGATATTTTACAAGTTATGAGGTGTTTTCTTATTTATTTATTATAGTTTTGAGGTGTTAAAGTGTATTTATCTATACTAAGGAAACAGGCACTTTGTG
CATAATTGTTTTTCAATGTTTTTTTTTTAAAATAAAAAGAAAGATAACAATAGAAATTAGTAGATAGTTTGTGAGGATAGTTTTAGAAATAAAATAATAA
CTTATAAATGTGTATATTAAAACATCATATTTTTTAAATATTATATATATATATAATTATAGATTAGTTGATATGCATTGTCTAACTCAAGTATTCTTTT
AAGGGTTTTATTGGTATTTTTGTGTTTCTAATTTGATGTGGATCGATTTATGAAAAGTATTAAATCTGAAACAATTCAATCCAATCTCATAAGGTATAGA
CTGACTTGATTATTTTGGTTTTGGTCTCATTTTTTAAGGGATAATATTTTATGTTTTAGTTAACAAACAAATAAAATTTTTAAGGTTATATTTTTTCACT
CATGCATGTGAATTACACAATCTGAAATTACTCTTGTATTCAGAAATTATGCGCACAGAGCCACACAAATAAAAAATAAATTATTCTAGTAAAAATCGGT
TCAATTTTCTAGAAACAATTCGGTTGATTGAATGGATTTTGTAAAACAGTGCACTTTATTTTTTTCAAACTAATTCAGATAAAAACCAGTTTGATTACAA
TCCGGTTTGGTCCAAAGTAATATTTTGAGACACCCTTACATATATACTCCACACATCATGTTATGCATTCATAAGTGAGTTACCAAACTCTTAAATCAAT
TGACATAAGATTATTTTAGGTTATATTTGAAGGAAATTTTATCGCTTATATTAATCTTATCCTTTTCAAACAAGATTGTCTAGTATGGTCTATAAAAAAA
ATTATGTTCATACTTGAAAAATAGAAATTGGGGTTGAAGAAATTGGTTCAAAAGTGAAAGATCTCATTATTGTATGGTAACAAAATATGCATGTTAACTT
TGCTAATGTGAAAAATGCAATTGTATGCAGTTGAAAGAAGAGTTGCAATCTCTTGCAAAGGTTGCATGTCCCATAATAATGACTAGCCTAATGATGTACT
CTCGATCTGCTGTGTCCATGTTGTTCTTGGGTCGGCAAGGGAAAGTAGAGCTAGCAGGAGGCTCACTAGCACTTGGATTTGCCAACATCACAGCCAATTC
AGTCCTCAAAGGCCTCACTATGGGAATGGACCCAATTTGTTGCCAAGCATATGGAGCCAAGAGATGGTCAGTTCTCAACCAAACCTTTCTCAGAACTCTG
TGTCTCCTCCTACTTGTTGCCATACCAATTTCAATTTTATGGCTAAACATGGAACCAATCCTTCAGATGCTAGGTCAAGACCCTGAAGTCACAAAAGTTG
CCCAAGTTTACATGGTCTTCTCTATTCCAGAGTTGCTAGCTCAGGCTCATCTCAATCCATTGAGGTCCTTCCTTAGAACTCAAGGCTTAACCACCCCAGT
TACTATAGCTGCTTCTTGTGCAGCACTCTTGCACCTTCCCATCAACTATTTCTTGGCCACATACTTAAACTTGGGAGTAAAGGGTATTGCATTAGCCACT
GGTTTGAACTCAATAAACATGACTTTGGGTTTGTTGCTCTATATTTTGTTCTCAAAGAAACCACTGAAGCCTTGGCAAGGTGCTACACTTCTCTCAGCCT
TTCATGGATGGAAACCATTGCTGAGTTTGGCATTGCCTAGTTGCATCTCAGTGTGCTTGGAGTGGTGGTGGTATGAGATAATGCTCTTTCTGTGTGGCCT
GTTGAGCAATCCACAAGCCACAGTTGCTACCATGGGGATCCTCATTCAAACACTAGGGTTCTTGTATGTGTTCCCATTCTCACTAAGCATAGCCTTGACA
ACAAGAATTGGTCACTCTTTAGGTGCAGGGCAAGCATCCAAGGCCCAAAGCACAGCCATAATAGGGTTTCTCACAGCATTCACCTTGGGCCTCACTGCCT
TCATTTTGCTGTTTCTTGTGAGGAAATCATGGGGGAAACTTTTCACTAATGAGACACAAATTGTTGAGCTGGTCACAACCATACTTCCCATTCTAGGCTT
GTGTGAGGTTAGCAATTGGCCTCAAACAGTTTCATGTGGGATTTTGTCAGGCACTGCTAGGCCTTATTTGGGTGCCAGAATAAACTTGTGTGCATTTTAT
CTAGTTGGATTGCCAGTTTCTGTTTTTGCTACATTCATATATAAATATGAATTGGTGGGTTTGTGGTCTGGAATGGTGGCTGCACAGGCTTCATGTCTTT
GTATGATGGTTTACACATTGATTCAAACAGATTGGGAGCAGCAATGTAAGAGGGCAGTGGAGCTAGCTCAGAAAACAACAGAGCGGGAAAATAAAAATGA
TGATGAGGAAAGTGGGTTGCTTGGTTCTGATCAATGA

>Glyma.03G036200 | Chr03:4360966..4367363 reverse
GTTCTTTTGCAGTTACACTTAAGGAGAGTCGCAAACCTTAACTACGACATTCTTCAGTGACACAGTACACTCATCTTTCTTGAGATACCATACCAAGGTC
TGATCAACATTTTTTTTTTAACCTAAGTTTTAATTATATCTACTCAATCATTTAAAATCCAAGTTGATAATTTTTTTTCCTTCTCATCACCACTTTTAAG
ACCATATTAGTTTATTTTTTTATTTTTTTCCCTTCGTCTCTTGGGGGGAGGAAAGGATCAATGTGTTTGTCTCTGAAGTGTTTAATTATATTTATATGTA
TGCATTGGTTTTCAAATTTGTACTTTAGATAAACATGTGCACTGGTGCTGAATCTCGAGGAGATATAAATGGTGATCCTGTGATTTCAAAGGTTGGAGGA
GGAGACTATGAGGATAGAATGAGAACCATAGAATTGGAAGGTGGTGGAGAAGAAAGAGCTTGCAACTGTGGCACATTTGTTCAAGGGCTTCTTCAAGGCA
TACACCATCATCTCACTGCCCTTAATCTCCCTACCCTTTCTGCTTCTGAGGTTGGTATAATCCCTCTCACTGTTCTTTTGTTCTTCAAACCTTTTCTATC
TTGCTGTTTTTCTAACTTCTTTTTGCTTTAAAAGGTGAAAGTTAGTATAAATATATTTGAAAACCTCATCATACAAATGAAAATGATGTATGAACACTCC
ATGAAATAAACATTCAAAACAGAGGTAGAAGTTGTAAATATACAGGAAATATAGCGTAATAAGAAAGAGAGAAATACAAAATAATAATTAATATTAGCAA
AATATTTACTCAATTAAGGTATCCAAATATACACTACCAAATATGCATTTGACATCGTCCAAATTGACATTGGTTATATTCACACTTAAGGCCTTTTTTT
TTTAATAGTGATAGTTATTCTCATTCTCATACTTGATGGTAGGAAGGAAAGTTTTAAGAAACTATATTGATATTGTATAATTGTATTGTGAAGTAAAAAA
CAAATGATATGATGCAAACTAATAAAGATTATGTTCACGTGAAAGGAGGAAAACGCCGAGACATCTATGCAGACAATTTTCTTGGAGCAAGTATTTCTTT
AATTGGTTTGAGCTTTTTGTCATTTTATTATAGTGATAAGCGGCAGTTTCAGTTAGGCAATCGAAAATATGTTGGAAAAAAATGTAGTTTTTTTTTTTTT
TTGGAAAGAACAGCTTTTCAAGAAGAAATCAGAAAATTTTGTAACAAAAAATTTTGAACAGCTCAAAATAAGTTAATTAAGCACATACTTCTTTTTAGTT
TAATTTAAATTAAATCTTTTTAATCCCTTTAGTTTAGGCACAAATCAAAGTGAGTATTAGTTAATGTTTCTTTAGAATTTGTTAAAAAATTGTCATTGAA
TTATATTTAAAAGATATTTTTAAATTCATTTAGACGTATATGAAGACATTTAAATTCTATAAAATAAATTACACAATATATTAAAAGAAAATATTATCTT
TTGATGACTTAAATAAGAAATAGTGTTGACCATATCTTAAGTGACCCTTTTATGTAAGGTATTTTAAGCTTCATTATTCCATCATGTAGGGAATTTTCCA
TTATTCTGGATGCTGAAACCTTCTATTATCAATACTAAATTGATCTTTTAAATAACCCATCTAACCTTTATCTACAGTATAGAGTACAGACATAAATTGC
TTGCTTGTCAATTTAAATCTATTGTGCTAATAATTGAGGCATGCACACGTGGGTGACCCTCTTTTTTTTTCTTTTTTCTTGTAACTTAATCATTGATTTC
TCTTCTCGTTAAAGTGGAATATAGATCTGTAGTTTAAATAGTAACTATTTATTTAATAAATTTTTATCAAATAAATAATAAGTTAAATTATAAAATCATT
ATAATAAATTAAAACTTGACAATTTTTCTTTACAATATGTAGATAAATGGGCTTAGTAAAAATTTATAAGGATATTATAAATCACTTTTATAAATATAAA
TAAACTCTTTTAAATACCATATATCTAAACCCTAAATAAAAAACATAGGTCGTTTGATGCTTTATTTGCTTTCTTTCCTAATCTTTCACTTAAAAATGGT
ACAAATTCACAATAGTTGATGATGTACGTACAAATTCACAATAGTTCTTGCCCTAAAATATTAGATTAATTGTTCATACTGAAAACAAAGAATAAACAAT
CAAGCACTTGCTTTGTAAGTAAGTTTGTGCTACATTTACATATGCATGGTCATTGTTCAACAATATGACGACCAATTCATTTTGTTTGTAATACTACTAG
TCACTTGTAGCAAAAATTGCGTAATTCTCTGTACATAACCTTTGATTGTGTTCTTGACTTTGGTTAAGTATAGATATTGGATTTCATTGTCTAATAGCAT
AAAGAATTTCTCTTACTCTTTTTCTGAATGGTTAAGGAATCTCAAGGATTGTTTGAAAGGGTTATAGGGATTCAGCTTTTTGAATGAAATACACTACAGC
TCTACAAGAATATAATTGATGTTAGCAAGAATATAATATCATAAAAAGAATTAAGCACTAACCATGTAAAAAAGTTTACGCAATTATTCAATCATAATTT
AATATATGTGATAAATTAATTTACTTTTAAAATAATTATTTTAAAATAATTCATAAAAAATAATTTCTTTACTACTCCATATACGCGTATTCTTTTCCGG
TTAACAAAAAAATCGAGTAGGACAGAATTATGAAAGTACCAAAAATCTAAACACAAAAGACAAATAAAATTCTTTATATATTTTTTAATTTTCAAATTTA
ACAATGGCATACTGTTTAATGTGTTTTTTTAATTTCTCAATATACATTTAACAAAATTTATCTAATGTTGAAAAACCATTGACATATCATTATATACTTT
AAGATTTTGACGGTAAAAATACTCGACATAAAGAAATTTCTTCCTGAGATATATTTTTTTGGATAAACAAAAGAATTTATTTAATAGGAAAATTTTCATT
TCAGGATATGTATGAACACGTACTTTGCTACTTACGTTGCAGTTTTGTCCCTGCTATACTAAGAAGAACATGATAGTTTTCAAGTAGAACTGGAATATAA
TCTATCTTGGCTTTCTTCTCATGTCATATATTTCAACAATCTATACTAATTAATTAATCTTGCTTGAGGCTAGTCAGTGATATATGGGAACTGAAAATCT
GAAATATGTTACCATGCCTTAGGTTATTATGGCCACGCACCTCCTCTCTTGCTCATCATCATTGTTTGATCCTGAGTAATTGCAAATGTGTCTGGGGTCA
ATATGATATAATTTGTATTCTTCTGCTTCATCCAGATGAATCGAGACCGAAAATATGGAGGCTCTGTCTCTTAGAAATCACTTGTGTCTGTTTCACACCA
CAAATCCTTTTTTGCCATATTGCATGGCACTTTTCAGTGTCGTGTCGTAATTATTATTATTATTATTATTATTATTATTATTATTATTATTATTATTATT
ATCATTATTATTATTTTACCTTTATCATAACATAGCACAAAATTTGGTATCGACTATCAAGTGAGATTCGAAAATCATTCATGGAACTGCTAATCGCTGA
TATACATATAATTAATAATTTTTTTACTACATAATTAAGAATATCTTGATTTTGTGCTAAATGAATTATGAATATAACCGTGATGTTCAACATTACTACG
TCTATAAAGCATAATTCATTGTTTAGTAGAATTTTGGTAAATAATTCTTGGACATTCTTTGGTGTAAAAGGTAACAAAAATAGAGAAGATATTTCTTATT
GATGAGTGTTCAAATATTTCTACCCTAAAATTTTATATAAAATATGCAGAACCTCGTGGTATAGAATATATAGATTATTTTTTAAAAACAATATTATGTT
TTCAAAAGCTTTTTCAAAACATTAATAAAAAAATATTTTTTTCTTCATAATAACCTAAACTATATGCGCACGTCTGTGAGACATGATCATAATCATAATA
CCGCAGTTTCTGTTGGCGGCTTGTCCTAAACTAGACCCTATGTCTGTTAGATCTGGAAGCATTTTTACATTTTGTCATTTTTTCACCTTTTTTTTAGCTT
CCTTTTTCCAGCTACACCATTAATATATAACACCTTCATATTTTATACACCAACAGAATTATGGCAGTAACTTTTCTTTTCAACCACTTGCCTATGCTTT
TCTTGCTAGGAACCTGACTAATTAAATTGAACAATTTACAAGCTTCGATGTTCCAACTTTTTTTCTTTTCTACTCATTATTTTATCAATTCTTTTAAAAC
GTCTTTACACTTCTGAACAAAAAAAAATAAAAATTAACCCCTTTTTCTTTCTCTGTCAGGTGATTTGACATTTTACAGTGCTGTTAAAGAAATTATGTAA
AACAAACTTTTTTCTTTATGAATAAAAATCGGGGAGAGACGGATCTCATCTGAACCTTGTTTTGATATTTTACAAGTTTTGACGTGTTAAAGTGTATCGA
TATTTTTTTATCAGCATTTTCCATAAGTTGACGTGCATTTTCTAATTCAAGTATTCTTTTAAGGGTTTTATGAGTGTTATTTTGTGTTTCTAATTTGATT
TGATCCATTTGTGAAAAATATTAAATATGAAACAATTCAATCCAATCTTATAAAGGAATAGATTGAATTGATTATTTTAGTTTTTGTCTTATTTTTTAAG
GGATAATCTTTTTATTTTTTATTTAACAAACAAAAAAATTATTAATTAAACATTGATTAAAAAAATGCGTTCATACTTGCAAAATAGAAATTGGGGTTGT
AAAAATTGGTTCTAATTTGAAAGATCTCATTATTCTATGGTAACAAAATATAATTTTTTATGAAAAAATGATAATTTGTTAGTTTTGATAAAAATATCAG
TTGGAGAAATTCCTTCGTCATTCTCCGATCACTGAAATCTCCACAAAATATGTATGTTAACTTGGCTAATATGAAAAATGCAATTTGTATGCAGTTGAAA
GAAGAGTTGCAATCTCTTGCAAAGGTTGCATGCCCCATAATAATGACTAGCCTAATGATGTACTCTCGATCTGCTGTGTCCATGTTGTTCTTGGGTCGGC
AAGGGAAAGTAGAGCTAGCAGGAGGCTCACTAGCACTTGGATTTGCCAACATCACTGCAAATTCAGTCCTCAAAGGCCTCACTATGGGAATGGACCCAAT
TTGTTGCCAAGCATATGGTGCAAAGAGATGGTCAGTTCTCAACCAAACCTTTCTCAGAACTCTATGTCTCCTCCTACTTGTTGCCATACCAATTTCAGTT
TTATGGCTAAACATGGAACCAATCCTTCAAATGCTAGGTCAAGACCCTGAAGTCACAAAAGTTGCCCAAGTTTACATGGTCTTCTCTATTCCAGAGTTGC
TAGCTCAGGCTCATCTTAATCCATTGAGGTCTTTCCTTAGAACTCAAGGCTTAACCACCCCAGTTACTATAGCTGCTTCTTGTGCAGCACTCTTGCACCT
TCCTATTAACTATTTCTTGGCCACATACTTAAACTTGGGGGTAAAGGGTATTGCATTAGCCACTGGTTTGAACTCAATAAACATGACTTTGGGTTTGTTG
CTATATATTTTGTTCTCAAAGAAACCACTTAAGCCTTGGCAAGGTGCTACACTTCTCTCAGCCTTTCATGGATGGAAACCACTGCTGAGTTTGGCATTGC
CTAGTTGCATCTCAGTGTGCTTGGAGTGGTGGTGGTATGAGATAATGTTATTTCTGTGCGGCTTGCTGAGCAATCCACAAGCCACAGTTGCTACCATGGG
GATCCTCATTCAAACACTAGGGTTCTTGTATGTGTTCCCATTTTCACTAAGCATAGCATTGACAACAAGAATTGGTCACTCACTAGGTGCAGGGCAAGCA
TCCAAGGCCCAAAGCACAGCCATGATAGGGTTTTTCACAGCATTCACCTTGGGGATCACTGCCTTCATTTTGCTGTTTTTTGTGAGGAAATCATGGGGTA
AACTTTTCACTAATGAGACACAAATTATTGAGTTGGTCACCACCATACTTCCCATTCTAGGGTTGTGTGAGGTTAGCAATTGGCCCCAAACAGTTTCATG
TGGGATTTTGTCAGGCACTGCTAGGCCTTATTTGGGTGCAAGAATAAACTTGTGTGCATTTTATCTAGTTGGATTGCCAGTTTCTGTTTTTGCTACATTC
ATATACAAGTATGAATTGGTGGGTTTGTGGTCTGGAATGGTGGCTGCACAGGCTTCATGTCTTTGTATGATGGTTTACACATTGATTCAAACAGATTGGG
GGCAACAATGTAAGAGGGCTTTGGAGCTAGCTCAGAAAGCAACAGAGCAGGAAAATAAAAATGATGAGGAAAGTGGGTTGCTTGGTTCTGATCAATGA

>Glyma.09G276100 | Chr09:49139110..49143810 reverse
TTTCTACCAGTCCCCAAACACCTTTCTGCAGTATCCATCTTCTCTTACAATACACATTGTTTAAGAGGGGCTGAGAGGCCTAACTATAACCTTGAATGTT
GGTACACATCGTGATTTTTGATATCAAGGTTTGGTTCATCTTCTTCGTCTTTTTTTTTTAATTATTATTTTGTTTTATGTATTTATTTATATATGATATA
TAGCTAGCTCCATGGGCAACAAAATCTTATTTTATGCATTCCATCAGCAAATTAAAAACAAGCTAACTCCATTTCCAACAAGTGTTTTTTAACCCCCTTA
ACATTACTTTTAAGGCCTTAAGTTAGTTTTTGTGTTTATTTTTTTTTCGTCTCTTAGGGGTTTGGAAGGATCGATATGCTTTCGTTTCCAATTACATTCT
CGGTTTTGTTAAATTAATTCTCAAGAGTTAGCCCATTAATATTATCTTACCAAATTTTCAAACATACCAAATGGTTATTAAATTTACCTTTTCACATAAG
CACTGATTCTTTAACAAATACTAGTATATATTTTGCTGAGAAAAATATTAGAAAAGTAATAATGTAAAATTAATAACCTATGTTTAGAAGATAATAATAT
AGTTTAGTTACTATATATATCTATAAGCTCATTAATCCATCTCTGAAATTGGTGTTATCTGTCCCCTTTAGCAAGAACTGTAACAAAAAGATAAACATGT
GCACTGGTGCTGAATCTCGAGGAATTGTAGATGGTAATAACCCCGTGACTAATTCAAAGGTTGGAGGAGTGTACCATGAGATCAACGGAATGAGAAGCAC
AGAAGGTGGTGATGGTGAAGGAAAAAGAAACAGTGGTTGTTCTGGCAGATTCATTCAAGGCCTTCATCAACACTTGCATCAACTCCTCAAGGCACTTTTC
CCTACCCTTTCTCTCTCTGAGGTTCGTAACTATATCTCTAGCTTCCTCTGTGTGGTTTTTGTTCTGAAAGTTCTTTTTGGGTCAAAAAGTGAAAATAAAT
CAAATAGACTGATCCTGTCAATGTTGTACGGAAAGAAGCTATAATAAATAACTATTTAGGATGTAAAGATGTTTTTGTTTTTAAAATACGTTACTCCTGC
CTCGTGGACATGTTGTTTAGATGATGATGCTACTGATGGGTGAAGGGGTTTCATCATGTTATTTAGATATATCTATATCATATATATTCTATCAATACAT
TTTTTTTTTAATCCATCACTTTCTTTTATATCATATAATCTATTATATGTATTTGATTTTGTGATAAGTTAAAGAAAATTTGACTAAATTTTAGTATAAT
TTCGGTATTATTACATATTTTACATGATTTTTGAAAGTTTATCGTGTAACCAAACATATTATATTATATTTTTTTTCTTATCGATAAATATTAATTTTTA
TTAATAAAAAGATTAAATCCTTGATTTCTTTTTTTTTCTTCTTTAATCATTTAACTTATTTTATATCTTCATTTTGTCTCCATATGTTGAATAGAGTACA
GTCTCCACTCAACTTTTCTCTATCTTCTTATTCTCTTCCTCAATTATGAAAAAGAAAAATAATATTTATAATGCAAGTTTTTAGTATGAAAACTGCACGA
AACCACTTTTCATTCAACTTTTTTTTATAAATTTATTCATAAGCAATTGTAGAAAAAGAAAATAAGAAAGAAATGTAGAATAAGAATGAAATTCTTCATC
GCCAATGACTGCTTGATCACGGACCTCGGTTATAATTTCCTCTATAGAAAATGTGTTTTTAGCTTTTATATTTGGATTGAGAAAACAGCCACAAGTGCTC
CAATGCTTTTGATTATAAAATAGTAAATTGATTGGCAATGCATAACATAACCTTTGTTTAGTTAGCTAAGGTGGTCGAATCATACATGAACCGCATTTTA
AGGTGGAAGCCGTTGTTGTTAATCTAGACTCAGTGTGTGTCGTTTAGGCGACCAATAATGATATTGGCATATATTTTACTTTTAGAAAGAAAAAAATTCA
CGGACTTTAAGGTGAACTCATTTGAATTTGCTAAAAAAACAAACAAGAATTTGTCCCGTTTTAGGGTGTATTTTTAAATACATAAATACAGATAACATTA
AAAAAAGAACTACAATAAGGTACGCATTATTTAATGACTTTCATCAAAATCTATGAAAAGTTTATGATGATAAATTACATGCAATTATCATTAGTGATAT
TAAACGAGTTAGTCAATTTATGTAAATCAATAGTATATTAATTTGGATTTGAGATGGAAACACCGGCACCAAAAATCTTCTTCCAACCCAGATTGCAAAT
TGAAACTATACCAGCCCACACAACCTCCATCCTAAAGCATTCTTCTCCTGTTACTCAACTTTTTTATTCTAGGATACGTAATCACGTTTTTCTTGTGCCT
TTTAGAAATTTTATAAGATTATAATTTTCTCAATAAATAGTTTTCTTGAATTAAAACTGATGACTTTATGTATATTACTCAAGTCCTTTGACCACTAAAC
TGACTATCCTAATGAGTTTTTCCCCGTAATTAGTTTATTGAGACATGTTATATTAGAGTGAAATTTGTAATTATTTTTTAAAAAAAAATTAAGGTGAAGC
ATATTTATTTTTATGTTATCTTAAAAAATTACAAATATTGCCCACTTTAATTTTTAAACAGTAAGCGCATTAGTCTCTAAAAATGACTAATAGTACTCAG
GTTTATAATTTCTAGAGAAGAAATTAATATTTTTAAAATCAAATATGACTGGCAACCAATGAAAATTTATGAAGATTGAATAAAATTTAAAATAGAAAAC
AAAAAAGAAAACATTCCAATTCAAATATCTTTTTATCAATTATTATTAAGTTTTGACTAATTTAATTAATTTTTAACAATTTTTATTCCACTAAATTTCT
GGGTGCGCAAACCGATAACCTATCTTTGCTATAATGGTTTAAAACTCTAAATAACCATAGATGTGACTGAAGTTTTACATTTTTAATGTTGACGTGTTGG
TGTTAATTTTTCAGGAAGAGAATTGAGAGTAGTTATTCCTGAAAGAGTAATTATATAACACTTATTTTCTAAAAACAATTTTGTTATTCATATCTGGTAA
AAAATAATTAAGCTACTATTATCATCATGTGTAACACATAGTATTTTGCTGACACATGAAATTAATGCACTTGCATGCAGGTGAAAGAAGAGTTGAAATC
ACTAGCCAACATTGCATGTCCAATGATGATGACCAACGTGCTTTTGTATTCTCGATCCGCTATTTCCATGCTTTACTTGGGTCGCCAAGGGAAAGTGGAG
CTAGCAGGAGGATCACTTGCAATAGGGTTTGCCAACATCACTGCCAATTCATTTCTCAAGGGTCTCACCATGGGAATGGACCCAATTTGTTGCCAAGCAT
ATGGTGCAAAAAGATGGTCAGTTTTAAGCCAAACTTTTTGCAAAACCTTGTGCCTCCTCCTCCTTGTAGCCATCCCCATTTCACTCCTTTGGCTCAACAT
GGCACCTCTCCTTCATTGGCTAGGGCAAGACCCTGAAGTCACAAAAGTTGCACAAGTTTACATGGTCTTCTCTATCCCAGAATTGCTTGCTCAAGTTCAT
CTCAACCCTTTAAGGTCTTTTTTAAGAACACAAGGGTTAACAACACCATTAACTATTGCAGCTTCTTTTGCTGCCATCTTACACCTTCCAATAAACTATT
TCTTGGCTACATATTTGGAATTAGGAGTAAAAGGCATTGCATTAGCCACAGGTTTGAACTCCATAAACATGATACTAGGGTTGGTGCTTTATCTTGTTGT
GTCAGAGAAACCCTTAAAGCCTTGGGAAGGTGTTACTATTCTCTCTTCCTTTCATGATTGGAGACCATTGCTAACCCTAGCATTGCCTAGTTGCATCTCA
GTGTGCTTGGAGTGGTGGTGCTATGAGATAATGCTCTTCCTATGTGGCTTGTTGAGTAATCCACAAACCACAATTGCTACCATGGGTGTACTAATTCAAA
CAACTGGGTTCTTATATGTGTTCCCATTTTCACTAAGTGCAGCATTGACGACACAAATTGGTCACTCCTTAGGTGCAGGTCAACCTTCACGTGCACAAAA
CACAGCCAAAATAGGACTCTTCATTGCATTTGCATTAGGTGTCTCAGCCTTTGTTTTCTTATTGTTTGTGAGGAATGTGTGGGGGAAACTCTTCACAAAC
GAGACACAAATTGTTGACATGGTCACAGCCATACTTCCAATTTTAGGGTTGTGTGAGATTGGAAACTGGCCTCAAACTGCTGCGTGTGGGATCTTATCAG
GGACTGCACGGCCTTATGTGGGTGCAAGAATAAACTTGTGTGCTTTTTACTTGATTGGATTGCCGGTTGCGATTTTTGCTGCTTTCATGCACAGGTACCA
ATTGAGGGGCTTGTGGTTTGGAATGCTAGCAGCACAAATTTCGTGTTTTTGTATGATGGTTTACACGTTGGTTCAGACGGATTGGGGGCACCAAAGTAGA
AGAGCTGAGCAATTGGCTCAAACAACAGATGAGGAAAATGTAAATAATGATGAGAAAAGTGGGCTCCTTGATTCTGATTTATGAGATTCATTTAAATCAA
TTTTGTGCTTAGTCACTAGCCAGTTACGATATAAGTGAGGTAATTCTCACCTTATAAATCAGTTTTGTAGAATTAAATTAGAGATGTAACTCACCTTCTA
A

>Glyma.18G214800 | Chr18:50165772..50170225 reverse
AACATTAGAAAAGAAATAATGTAAAATAACCTATGTTTAGAAGATAATAATATAGTTTAGTTTAGTCTATAATATATATAAGCACATTATTAATCAATCT
CTGAAATTGGTGCTGAATCTCGAGGAATTGTAGATGGTAATAACCCCGTGAAAAATTCAAAGGTTGGAGGAGTGTACCATGAGATCAACAGAATGAGAAG
CACAAAAGGTGGCGATGGTGAAGGAAAAAGAAACTGTGGTTGTTGTGGCAGATTCATTGAAGGCCTTCATCAACACTTACATCAATTCCTTAAGGCACTT
TTCCCTACCCTTTCTCTCTCTGAGGTTTGTAAAATATCTCTTCTTGTATGTGTGTTTTAGTTCTGCAACTGCAAGTTCTTCTGCTGCTATGATTCCACTT
TTAAAAGATTGTTTTATTTTTCCTTTTTGGGTCAAAAAGTGAAAATGTTTGAATATATCATAATTTTTAGAATTTAATAAAATAAACTGATCCTGTCAAT
GTTGTACGGAAAAGAGATTATAATAAATAATTATTTAGCATTGTAAAGATTTATTTTAATATGTTGCTCCTGCCTCTCGTGGCCATGGTTGTTAAGATGC
TGATGGATGGAGGGTTTCATCATGTTATTTTCTCAAGTCATGATGCATAGATATCTCTATATATCATATGTATACAACACACTTTATTAAATATATACTA
GTAATTTTTTTATCTTTCACTTTTTATCACATTATATAATAAAATTTATTATGTGTTTGATTTTGTGTTGAATTAAAGAAAATTTGACTAAATTTTGGTA
TCATTTCGGTATTTTATGCAATTTTTGTAAGGTTTATCGCATACCAAACATGTTGTATATCTATATTTTTCTCTCATTCCATCTCCAGTTGTCGAATAGA
GTATAGTCTCCACCAATCTTTTTCTTGATTTTTTCATTTTCCCTCACAGATACTTGTGGAAATTTTTTATTTCACTCTATCACTACTCTTGTATTTTTAT
CATAAACAAGTTTCATTAGTTCACTCTCTTTTGTTAATTATTTTCAAACAACAATTTTCATAACAAATAGTCATTCTCTATCAAAAAAGAAAAAGTAAAA
AACCATCAAGCAATATTAGCTGAGAGTTACAACTTACAATGTTTGAAATTCTTCATCTATAGAAAATGGCTTTTTCCATTTTATATTTTGGATTGAGAAA
GTAGTGAGCACAAGTGCACCAAGGGCTTTGATTATAAAATAGTAAATTGGTTGGAAATACATATAACATAACTTTCGTCTAGTTGGCTAAGTTGGTCGAA
TCATACAAGAACCACATTTTAAGGTGGAAGCCGTTGTTGTTACTCTCAATGTGTATCGTTTAGGCAATCAATATTGATATTGGCATATGTAGTAGTATTT
TACTTTTATAATTTTTTTTTCACGGAATTTAAGGTAAACTCATTTGAATTAATTAAAAAGCAAACAAAAATTTGTCCCGTTTTAGGGTTTATTTTTTTAA
ATACATATATACAGATAACATTAAAAAAACTACACTAAGGTACGTATTACTTAACGACTTTCATCAAAATCTATAATTTTATGATGATAAATTAAATGCA
ATTATCATTAGTGATATTTAAAAGAGTCAGTCAATATATGTAACTCAATAGTATATTAATTTGTGCAAATTGAAACTATACCAACCGTGGGCAGAAGAGT
CGCGATCTCTAAAGTGACTAGTTTTAAAGAAATTGCCAAGAGTCACAAACTTTAACTTGAGTCATCGTATGACTATGAATATGTGATCATGGCACGAATT
TCAACTAACTGATTTTCATTCAATCATTCTTAACATCTTGCTAAGAGNNNNNNNNNNNNNNNNNNNNNNNNNNNNNNNNNNNNNNNNNNNNNNNNNNNNN
NNNNNNNNNNNNNNNNNNNNNNNNNNNNNNNNNNNNNNNNNNNNNNNACCCCCCCCGCCAATCCTAAAGCATCTGTTACTCCCCATTTTGATTTTAGGAT
ACGTGATCCCGTTTTTCTTATGCCTTTTACAAAATTTATAAGTTTATAATTTTCTCAATAAATAGTTTTTTATATATATATATACAGTTGTTGCACTTGA
GTCTGAAACTTCATGTATATTACTCAAAATCCTCTAAGCACTATCCTAGTGAGTTTTTCTTGGTAATTAGTTTATTGAGACATTAGAGTCAAATTTAATT
TTTTTTCTTAAAAAATAAGGTGAGAAATATTTATTTTGTGATTGATCTTTTAAAAATGACAAATAGTGTTCATATTAATTTTTAAACAGTTAGCTCATTA
GTCTCTAAAAATGACTAATATTACTCACGTTTATAATTTCTAGAGATGAAATTAATCTATATTATTCTAAAAATTGTTCAGCCACCCATCACATTTACAC
GTGTTGCTTTTTTTGTGCAATATAGGTCACTTTTTTTTTTCTCTCTCTCTCTTTTTTACTGTTGACTGCAATTAATTGCTGTTTCTATTTTTTCACTTTT
ACGGCACTTGCTCTTACTGGTTTAAAACTCTAAATATCTTTGTTATTATGGTTTAAAACTCTAAATAACCATAGATGGGACAGAAGTTTTACATTTTTAA
TGTTGACGTGTTGGTGTCAAATTTTTAGGAAACGAATTTGAGAGTTTATGCTTGAAAGAGTAATGATATACCGTAACACTTATTTTTTCAAAATAATTTT
CTTATTCAAAACTGGTAAAAATAAGCTATTGTTAATTTATCATCATAATGTGTAAAACACATTTTGCTGATGTATATCAAATATGCACTTGAATTGCATG
CAGGTGAAAGAAGAGTTGAAATCACTAGCCAACATTGCATGTCCAATGATGATGACCAACGTGCTTCTGTACTCTCGATCCGCCATTTCCATGCTTTTCT
TGGGTCGCCAAGGGAAAGTGGAGCTAGCAGGAGGATCACTTGCAATAGGGTTTGCCAACATCACTGCCAATTCATTCCTCAAGGGTCTCACCATGGGAAT
GGACCCAATTTGTTGCCAAGCATATGGTGCAAAAAGATGGTCAGTTCTAAGCCAAACTTTTTGCAAAACCTTGTGCCTCCTCCTCCTTGTTGCCATCCCC
ATTTCACTCCTTTGGCTCAACATGGCACCCCTCCTTCATTGGCTTGGGCAGGACCCTGAAGTCACAAAAGTTGCACAAGTTTACATGGTCTTCTCTATCC
CAGAATTGCTTGCTCAAGTTCATCTCAACCCTTTAAGGTCTTTTTTAAGAACACAAGGGTTAACAACACCATTAACTATTGCTGCTTCTTTTGCTGCCAT
CTTACACCTTCCAATAAACTACTTCTTGGCTACATATTTGGAGTTAGGAGTAAAAGGCATTGCATTAGCCACAGGTTTGAATTCCATAAACATGATACTA
GGGTTGGTGCTTTATCTTCTTGTGTCAAAGAAACCCTTAAAACCTTGGGAAGGTGCTACTATTCTCTCTTCCTTCCATGATTGGAGACCATTGCTAACTC
TAGCATTGCCTAGTTGCATCTCAGTGTGCTTGGAGTGGTGGTGCTATGAGATAATGCTCTTCCTATGTGGCCTATTGAGTAATCCACAAACCACAGTTGC
TACCATGGGTGTACTAATTCAAACAACTGGGTTCTTATATGTGTTCCCATTTTCACTGAGTGCTGCATTGACAACACAAATTGGCCACTCCTTAGGTGCA
GGTCAACCTTCACGTGCACAAATCACAGCCACAATTGGACTCTTCATTGCATTTGCATTAGGGTTCTCAGCCTTTGTTTTCTTGCTGATTGTGAGGAACG
TGTGGGGGAAACTCTTCACAAACGAGACACAAATTGTTGACATGGTCACAACCATACTTCCAATTTTAGGGTTGTGTGAGATTGGAAACTGGCCTCAAAC
TGCTGCGTGTGGGATCTTGTCTGGGACTGCACGGCCTTATGTGGGTGCAAGGATAAACTTGTGTGCTTTTTACTTGATTGGATTGCCGGTTGCAGTTTTT
GCTGCTTTCATGCACAGGTACCAATTGAGGGGCTTGTGGTTTGGAATGTTGGCAGCGCAAATTTCATGTTTTTGTATGATGGTTTACACGTTGGTTCAAA
CGGATTGGGGGCACCAAAGTAGAAGAGCTGAGCAATTGGCTCAAGCAACTGATGAGGAAATTAGTGGGTTCCTTGATTCTGATATGTGAGATGTTTAAAT
CAATTTTGTGCAGTGATAGTCACTAGTCACTAGAACTACACGACCAGTTACAAGGCATTAATTCTAGTTATTGAGATTTCTCATCATTGCTCATTAATTA
GTCATTCATTATGGTAGGAAGTAAGAACATATTTTCTTCGTAATATTCAATTAGATGTACATAAGAAAAGCTTTGTTAGTGAACTCATTCACGCATGATG
AATTTTTTAAATAACAATACATCATAATTAGGTGGGTACAAATACCTGAGCTCA

>Glyma.01G007100 | Chr01:707570..708601 reverse
ATGGCACAAACCTACTTGCTCTTCCTCCTCCCCGACCTCCTTATCAACTCTTTCCTCCACCCAATCAGAGTCTACCTTCGCGCCCAGAACGTCACCCACC
CTGTCACCTTAGCGTCCCTCGCCGGCACCCTCCTCCACGTGGCCTTTAACCTGGCTCTCGTCGAGCGCGGCCTGGGTGGCGTCGCCGCCGCCGCCGCCGC
CTCCAGCTTTTCCATTCTATGTTTGCTGGTTCTCTATTTGTGGATAAGTGGGGTCCACCTGGCCACGTGGACCGCGCCGAGCCGGGAGTGTTTAACCTGC
TGGGAGCCGCTAATTCGGCTCGCGGCGCCGAGCTGTGTCTCCGTTTGTTTGGAGTGGTGGTGGTATGAGATCATGATTTTGTTGTGTGGGTTGTTGGTGG
ACCCCACTGCCAGCGTGGCGGCTATGGGGATTTTAATCCAGACTACGTCCTTGATCTACGTTTTTCCCTCCTCGCTGGGGCTTGCGGTGTCTACGCGCGT
GGGAAACGAGCTTGGCGCCAACAGGGGACGACGCGCCAGGATGTCGGCGGTGGTGGCGGTGTTCTTTGCCGCCGTAATGGGGTTCTCCGCGGTGGTTTTT
GCGACGGCGATGCGGAGGAGGTGGGGGAGGATGTTCACCGGAGACGAGGGGATTCTGCGGCTGACGGGGGCGGCGCTACCGATTCTGGGGCTGTGCGAGC
TGGGAAACTGCCCGCAGACGGTGGGGTGCGGGGTGGTGAGGGGGACGGCGAGGCCGAACGTGGCGGCGAATGTGAACCTGGGAGCGTTTTATCTGGTGGG
GATGCCCGTGGCAGTTGGGCTTGCGTTCTGGCTCGAGGTTGGGTTCTGTGGGCTCTGGCTGGGCCTATTGTCGGCCCAGGTTTGTTGTGCGGGCCTCATG
TTGTATATGATTGGGACCACCGATTGGGAGTACCAGGCCTGTCGGGCCCAGTTACTTACGGCCCTTGATGAAGGATCCGACGGCCACAAACAACCGTTGA
TTGCCACTTTGGACAACAACAACTACTCTTGA

>Glyma.07G124600 | Chr07:14837991..14840026 forward
GGGTCACTTACACATAGTATTTTAAACAGGAAAAACACGCTCGAATTCAAATCCTCCTCAATTCTATTTATACTCGCCCATCCTTCTCACTTCTTCACTC
CCACGCAGTAATTAGTTTCTTTCTTCATTTATCACTCTGCTCCTAGCTCCCACCGCTTTCCACCCTTTAAAAAAAAACAGAGTCCCCTCTCCTCCTCCTC
CTCCTCCTCCTCTTTGTTTCAGAACACACTGTCTAGTGTATGTGTATCGCAGTTTTCACAACACGCACTCTTCCTTCATCATATTATTCAAAATCATTCA
TAAACACGTACATATATACCCTCTTTTGAGTCTTCATTTCATAACAACGAAGGAAGACGTGTAGTAGTAGTAGTAGTAGTCGTCGTCATAAAATAGAAGA
ACCAAGAACATGTGCGAATCAAAATCAGAGCAGAGGCATGATGATAAGGAAAAGCAGACCGAAGAATTAGGTGGTGTGTTAACAAGACGTTCGACAATAA
TGTGGGATGTGATAGGAGAAGCGAAATCCCTGTGGGAGTTGGCATTCCCGACAGCACTCACTGGTCTCATTTTCTACGCGCGTTCCATGGTCTCCATGCT
GTTCCTCGGCCACCTAGGAGACACCGAACTGGCCGCAGGTTCCTTAGCCATCGCCTTCGCCAACATCACCGGTTACTCCGTTCTCTCTGGTCTCTCCTTA
GGAATGGAACCTCTTTGTTCCCAAGCCTTCGGAGCTAAGCGTCCTAAACTCCTCTCTCTCACCCTCCAACGCTGCGTCATCTTCTTATTATTCTCTTCAA
TCCCCATTTCGCTCTTGTGGCTTAACATGTCTAAAGTCTTTATCCTACTCCACCAACACACACACATCACCCAAATGGCACAAACCTATTTAGTTTTCCT
TCTCCCCGACCTCGTCACCAACTCCTTCCTCCACCCAATCAGAGTCTACCTTCGCGCCCAGAACATCACCCACCCGGTAACGTTAGCGTCCCTCGCCGGC
ACTCTCCTCCACGTTCCATTCAACTTGCTCCTCGTCCAGCGCGGCCTGCCCGGCGTCGCCGCCGCCTCCGCCGCCTCCAGTTTTTCCATTCTAAGTCTGC
TGGTCCTCTACGTGTGGATAAGTGGGGTCCACCTGGCCACGTGGACCGCGCCGAGCCGGGAGTGTTTCGGCGGCTGGGAGCCGCTGCTTAGGCTTGCGGC
GCCGAGCTGCGTCTCGGTTTGTTTGGAATGGTGGTGGTATGAGATTATGATTTTGTTGTGTGGGGTTTTGGTGGACCCCACTGCCAGCGTGGCGGCTATG
GGGATTTTTAATCCAGACGACGTCGTTGATCTATGTCTTCCCCTCGTCTCTGGGATTTGCGGTTTCCACGCGCGTGGGGAACCAGCTTGGCGCGAACAGG
GTCCACGCGCCAGGATGTCGGCGGTGGTAGCGGTGTTCTTCGCCGCAGTGATGGGCTTCTCCGCGGTGGTTTTTGCGACGGCGATGCGGAGGAGGTGGGG
GAGGATGTTCACCGGGGACGAGGGGATTCTGCGGCTGACGGCAGCGGCGCTGCCGATTCTGGGGTTGTGCGAGCTCGGAAACTGCCCGCAGACGGTGGGA
TGCGGGGTGGTGAGGGGGACGGCGCGGCCGAACGTGGCGGCGAACGTGAACCTGGGAGCGTTTTATCTGGTGGGGATGCCGGTGGCGGTTGGGCTTGCGT
TCTGGCTCGAGGTTGGGTTCTGTGGGCTCTGGCTGGGCCTGTTGTCGGCCCAGGTTTGTTGCGCGGGCCTAATGTTGTATATGATTGGGACCACCGATTG
GGAGTACCAGGCCTGCCGGGCCCAGTTACTCACGGCCCTTGATCAAGGATCCGACGGCCACAAACAACCGTTGCTTGCCGCTGTGGACAACAACAACTCT
TGATAAAAAAAAAAAAAAAAATTATGATGATTGTTGATTGACGGTGTAATATTCTTTTCTTTTTGTAATAATTCTTATATTTATTAAAGGGAATTAATTT
GTGAAATACAGTTATTATTATTCTTCTTTTATTCAC

>Glyma.05G226400 | Chr05:40480968..40482410 reverse
ATGGAGGGAGCAGCAGAAAAGGAGAGGCTGCAGTGTTGGAGCATAAGGAGAGAAGTGAAAGCGGTGGTGGAGCTGGCGTTTCCGATAGGAATAACGGCGT
TGATATTCTACGCGCGTTCCATGGTATCGATGCTGTTCCTGGGACACCTGGGAGAGTTAGAACTGGCAGCAGGGTCCCTAGGAATGGCGTTCGCGAACAT
CACAGGTTATTCGGTGCTGTCGGGTCTAGCACTTGGGATGGAACCGATGTGCTCGCAAGCCTTCGGAGCAAAACGCGTGAAGGTGTTGTCGTTGACCCTC
CACCGCTGCGTCATGTTCCTATTAGTCTGCTCCATCCCAATATCACTCCTTTGGCTCAACATGTCCAGCATCCTCCTTCTCTTGCACCAGGACCCAAACA
TCACCCTCATGGCCCACACCTACCTCATCTTCTCCCTCCCTGACCTCCTCACCCACTCCTTCCTTCACCCAATCCGCATTTACCTTCGCGCCCAGGGCGT
CACCCACCCGGTCACCTTAGCCTCCCTCGCCGGAACCCTCCTCCACCTCCCCTTCAACTACCTCCTCGTCACGCGCCTCCGCCTCGGCCTCGCCGGTGTC
GCCGCTGCCTCCGCCGCTTCCAACCTCTCCATCCTCCTCTTCCTCGGAGCCGCCGTTTGTTTCACCGGCCTGCACTGCGCCGCCCCGAGCCGAGACTGTT
TCAGCGGCTGGAAGCCGCTGCTTCGGCTCGCCGCGCCGAGCTGCGTCTCTGTTTGCTTGGAGTGGTGGTGGTATGAGATCATGATCATTCTTTGCGGGAT
TCTCGTGGACCCCACCGCTACCGTTGCTTCCATGGGAATACTCATTCAAACCACTTCACTCATCTATGTTTTCCCCTCCTCGCTTGGTTTCGCGGTTTCC
ACACGCGTCGGGAACGAGCTCGGAGCGAATCGTCCTTCTCGAGCCAAATTATCCGCGGTGGTTTCTGTTTTTCTCGCCGCGATTATGGGGTTCTCGGCGA
TGTTTTTCGCCGTCGGGATGCGGCGGCGGTGGGGGAGGATGTTCACCGCCGACGAGGACATAATAAGGATCACGTCGATGGCGCTGCCGATCCTCGGGAT
TTGTGAACTCGGGAACTGTCCGCAGACGGTGGGGTGCGGCATCGTTAGAGGGATGGCGCGGCCGAACACGGCGGCCAATGTGAACCTGGGCGCGTTCTAT
TTGGTCGGGATGCCCGTGGCGGTGGGGCTTGGGTTCTGGTTTGACGTTGGGTTCTGTGGGCTGTGGCTGGGCCTGCTCTCGGCCCAGGTGTGTTGCGCGG
GACTTATGTTGTATGTGATTGGGACCACCGATTGGGAGTTTGAAGCCCATCGGGCCCAGTGGCTGACGTTGGTCGAAGATGGAGTGATGGACGGACAGAA
ACAGCCGTTGACAAGCGTTGTTACCGAGGCTCCCTCGACTTGA

>Glyma.08G033200 | Chr08:2649700..2651523 reverse
TCTCATTGAAACGCGTTAATTAGTTTCGTGTATGTATCTAGCTAGCTAGCTAATCCCACAGACTCAATCAGTCTTTACTGAGAAGAAGACAAGAGAGAAG
AGAGGGGGTGGTGTTTAGGAGAGTGAGAGTGAGAGTGCAAGTTTGAGTTTGAGAGGATCCATTAAGATGGGATCAAAAACAGAGGAAGTAGTAGATTACA
CGTTGATGGAGGGAGCAGTAGAAAAAGAGGGGCAGCACTGTTGGAGCATAAGGAGAGAAGTGAAAGCGGTGGGGGAGCTGGCGTTTCCGATAGCCTTAAC
GGCGCTGATATTCTACGCGCGTTCCATGGTATCGATGCTGTTCCTGGGACACCTAGGAGAGCTAGAACTGGCAGCAGGGTCCCTAGGAATGGCGTTCGCG
AACATCACAGGCTATTCGGTGCTGTCGGGTCTAGCACTCGGGATGGAACCGCTATGCTCCCAAGCATTCGGCGCAAAACGCGTGAATGTGTTGTCGTTGA
CCCTCCACCGCTGCGTCATGTTCCTGTTACTCTGCTCCATCCCAATATCACTCCTCTGGCTCAACATGTCCAACATCCTCGTTGATCTCTTGCACCAGGA
CCCCAACATCACCCTAATGGCCCACACCTACCTCCTCTTCTCCCTCCCCGACCTCCTCACCCACTCCTTCCTCCACCCAATCCGCATTTACCTTCGCGCC
CAGGGCGTCACCCACCCTGTCACCTTAGCTTCCCTCGCCGGAACCCTCCTCCACCTCCCCTTCAACTACCTCCTCGTCACGCGCCTCCGCCTCGGCCTCG
CCGGTGTCGCCGCTGCCTCCGCCGCTTCCAACCTCTCCATCCTCCTCTTCCTCGGTGCCGCCGTCTTCTTTTCCGGCTTGCACTGCTCCGCGCCGAGCCG
AGAATGTCTCAGCGGCTGGAAGCCGCTCCTTCGGCTCGCTGCGCCTAGCTGCGTCTCCGTTTGCTTGGAGTGGTGGTGGTATGAGATCATGATCATTCTT
TGCGGGCTTCTCGTGGACCCCACCGCTACCGTTGCTTCCATGGGGATACTCATTCAAATCACTTCACTCATCTATGTTTTTCCCTCCTCGCTTGGTTTCG
CGGTTTCGACTCGCGTCGGGAACGCGCTGGGAGCCAATCGTCCCTCTCGGGCCAAACTATCCGCGGTGGTTTCTGTTTTTCTCGCAGCGATTATGGGATT
CTCGGCGATGTTTTTCGCCGTCGGGATGCGGCGGCGGTGGGGGACGATGTTCACCGCCGACGAGGACATATTAAGGATCACGTCGATGGCGCTGCCGATC
CTCGGAATTTGCGAACTCGGGAACTGTCCGCAGACGGTGGGGTGCGGCGTCGTTAGAGGGACGGCGCGGCCGAACACGGCGGCGAATGTGAACCTGGGCG
CGTTCTATTTGGTCGGGATGCCCGTGGCAGTTGGACTTGGGTTCTGGTTTGACGTTGGATTCTGTGGGCTATGGCTGGGCCTGCTCTCGGCCCAGGTGTG
TTGCGCGGGTCTTATGTTGTATGTGATTGGGACCACCGATTGGGAGTTTGAAGCCCATCGGGCCCAGTTGCTGACGTTGGTCGAAGATGGAGTGATGGAC
GGACAGAAACAGCCGTTGACGGGTGTTGTTACTGAGACTCCCTCGTCTTGATCGTTTGTTTGATCAAAAGCAACAACACCCCGTGCTGATCCTGATGGGG
TTATTTTGTAATTTACTAAGGTCAAAGAAAGTGAATTATAACTATATTTATTTTTTTTCGCCTCTTTTTTTTTTTTTTCCCACCCATATGGGAACTGGAT
ACATATACAGAGGATAACTTAAAG

>Glyma.02G075300 | Chr02:6597633..6602520 reverse
TTTTCTAATTCTTGCTGCTTTCTATCATGGGATAATAGATAGATATGATAATCAAACATTGTAGACCCTCTTTTGTTTCTATCTCCTTTTCTTTCTTTCA
CTCTCCCCTATATGAAGCTACTACTACTATTGCTCTATAGGTGTTGTCAGAAGTAGTCTTTCTTCTTCTCTTCTAACTTCTTCACCACCCTTTTGCAGAA
CAACAACGCATACATATATTGGTCAAGAAGAATGGGTGATAACAAGGACCGGGATTTCTTTTCCCACAAATTCCCCACAACCTCTCAGGTAAACTCATGT
TGCTAGTTGCTACTTCTCTCTATGGAAACGCATTTGTAAGAGAAAAAAAGAGTAGTTTCTTTTTAAGAAACCCTACCATGATGCTAAAATCAAGTCAAGG
TTTTAAAAACCGGTTCGCACGATCTTGGTCCCATCATTTAAGGTTTTTTGGAGTGTCTGCAACTGTAACACGATCATATAGACTGCAGACCACATTTGTC
TGTAATTTCTCAACATCAAAGAACGTGACAAAAAAATTGTGACGGTAGCCGCAATTTAAAAGCTTGATGCTAGTAAAAAGAAAAAGACTATTGTGTTAAA
AGTAAGTTTTTTTTCCCCTTCTTATTTGTGTTCTAATCATGTGATGTTGTTGTTGGCAAGTAGGTGATGGAAGAGATGAAGGAACTGTGGGGCATGGCTC
TACCCATAACAGCTATGAACATGTTGGTATTTGTGAGGGCAGTGGTTTCTGTACTCTTTTTGGGTAGGCTTGGAAGCCTGGAGCTCGCAGGTGGTGCACT
TTCCATAGGCTTCACCAACATAACAGGGTACTCTGTTCTCGTGGGTCTTGCAGCAGGCCTAGAACCTGTGTGCAGCCAAGCCTATGGTAGCAAAAACTGG
GACCTTCTCTCACTCTCTCTCCAACGCATGGTCCTCATCCTCCTCATGGCAATAGTCCCCATAAGCCTCCTATGGCTTAACCTAGAAAGGATCATGCTTT
TCATGGGCCAAGACAGTGCCATCACAGGAATGGCATCACTCTACTGCTTCTACTCCCTCCCAGACCTTTTAACAAACACCTTGCTTCAACCCTTAAGGGT
GTTTTTGAGGTCACAAAAAGTGACCAAGCCTATGATGTATTGCTCCCTTGTGGCTGTTCTGTTCCATGTGCCATTGAACTATCTTTTGGTGGTTGTGATG
GGGCTTGGGGTGCCAGGGGTGGCCATGGCTTCGGTGATGACTAATCTGAACATGGTGGTGCTGATGGCAGGGTATGTGTGTGTGTGCAGGAAGAGGGAGG
TGGTGGTGAAGTGGGGGTGTTGGGGTGTGGGTGGGGGAGTGGTGTGTTCTGGGTTGGGGCAGTTGATGGGTTTTGCTGTGCCTAGTTGCCTAATGATATG
TTTGGAGTGGTGGTGGTACGAGATTGTCACTGTGCTGGCTGGGTACTTGCCACGTCCAACACTGGCTGTGGCTGCCACTGGTATTCTCATTCAGACAACT
AGCATGATGTACACAGTCCCAATGGCACTTGCAGGCTGTGTTTCTGCCAGGGTGAGTCTCCCTCTCTCTCTCTCTCTCTCTCTCTATAGACTATACTACA
CTTTGTATTCATTGATACTCCTCCTAAACCTAGGGAGAGGTATTTAGTCAAATTTTTACATGCTCACCCAAATGTATATTATATTTGGATTAAAGTTTGC
ATAACTGAGTTTTTAAGAGTAACTCAAAGATGGAAAACCAATCATAGCCTCAGAATTGTCTGATGAAGATTTGGTCAGATGGTTCATATTTTATTTTTTT
AAAAACACTCAAAATTTGAACCATTTGATCTTTTATCTGACGGTTATAAATACAATGACGGCTTAAGTGTGTATAAATTTAACTACATTAGATAGGAAAT
ATAATGTGATAAGTAGAAAAAAGAGAAATGAAAGATGTCAACTGAAAGTAAGAATATTATTATTCTATTTTAATAACATAGTGTTCACAAAGAGGATAAG
AAAACTTTTTTGACCATGTTGGGTTTTCTTTTTATTCTAGTTTTGAGTGGTTAATAGTTCATTTTTTTTACCCTTTTTAACCAGTTGGGTTGGTTGAGAC
AGAAGAAGATAGGGACTAGAGAGTGGTGTGCAGACAGTGCAGGTAGCTTGGCTGGTTTGTTTTTTTTTTTTTTCTCACTTTTTGTGTCTGTGAGATTTGG
TGTTAGAATTTGAGCAGAAAGGAACCATTACTTTTAGCAATTTGGCTTATGTGGAATGGGCAGCAGGGATAAGTTCAAATTTGCAATTTATTTTTTGGTT
TTAAATTTAATGTATCTGGCCTATCATAAATGGTAGTAAATACTATGATAGGCTAGTTTATTAGCTTACTTTTTTGTTTACTTGCTTTATTTTGCAAGAA
TGGAAGTAAATACTAGGCATATTTTGATTATATATTTATTTATTTATTTGGTTATACTAGACAAGTGTATATATAACCCATGACTGTTTCCAAAATATTT
GGCTTGCTTTTATTCTTTCATCATATTATTTCTATCTTTTGTTTATCTAAACACTTCAAACATAGTATTGATTGAGCTATACACATGTTTCTTTATATAA
GATGCTTTACATTTTCTTTGACAAAACATATTTGGAAGGCATTAAATTGTCTTCATCCACATGTGTGCATTTTAACTTTTGTCGTGAAAACAATCCTAAA
CAAACCACTAGGCTTAGCTTTAACCTTTGAGGCAAGGACATAAATGGTCAATTATAATAACAAGTTTTTTTTCCTCACAATACAATATTATTGATTTGTT
AAAGAAGGTCCATAACAATAGGTTGGAGTGATGATGATGAAGAAGAAGATAGTTGTAGTAGTAGGTTGGATCATATTTTATTTTTATTTATTTTTTCTTT
CTCACTGTTTTAATGGTTTGTTAGAGATCATGAGAAATTAGTGGCAACCGTAGGTCTCTTCCCTACCTCTATCTGATCAGATGCTAAATACGCGTGTGGA
GGTTGTGTACTGCTATTTTTCTTTACAGTTTACACCCTGAATGTCTCATCTTTTTCTTCTTTTTGGGCTAAAACGTGAGCCCCCTTAGTTTCTGTTTGCT
GAGGTATAAAAAATTTGTTGCATGATTGCGTCAAGTACATGGACCCAGTTAGGTCACACATGCAATGTGACTGGACAAAACTGAAAACTAATTAATTACT
TCTAAGTAAGTGCATTTCTGCTAGGCTTGCATACAAGGCCCATCATAGATTTCCATTTGACCAAATAATTTAACCATCAAATCGCCATTGGACTCCACTC
TTCTGGTGGGACTCATCAAACAGTTCGGTACTTGATAACGTATCTTGGTACATTTCTGTTTGCACCACAAGTTAACTTGCACATAACATTGGTATGCATG
ATGTACACTTTGAATTATACCCACATGAAATTTGCCATGAAGCACTTAATAAGGCTATGCTTGTATATTAGAGTTACAAAAGTAATTCTGTGAATTTTAA
TATACAATTGTGAACGTGTATAAAACAAAAGAAAGAAAAACTTATTTTAAAAGTAAAAGTACTTTTAAATTTATCTAACTGAAATTCAAACATATACTAA
ACAGTTTCAGATTTTCAATGCAATTAATGCAGGTAGGGAATGAGCTTGGAGCTGGAAAACCATACAAGGCAAAGCTAGCAGCAGTGGTTGCCTTAGGATG
TGCTTTTGTGATAGGCTTCATCAATGTGACATGGACTGTGATTCTGGGTCAAAGATGGGCTGGGCTTTTCACCAATGATGAGCCCGTCAAAGCGTTGGTT
GCATCAGTTATGCCAATTATGGGCTTGTGTGAGCTTGGAAACTGCCCACAAACCACGGGCTGTGGGATCCTTCGTGGCATGGCCCGGCCTGGTATTGGGG
CTCACATAAACCTGGGCTCTTTCTACTTTGTGGGCACCCCGGTGGCAGTGGGCCTGGCATTTTGGTTCAAGGTTGGGTTCAGTGGGCTTTGGTTTGGGCT
TCTGTCGGCCCAGGTTGCATGTGCTGTGTCAATCCTCTATGTTGTGTTGGTGAGGACAGATTGGGAAGCTGAGGCTTTGAAGGCTGAGAAGCTCACAAGG
ATTGAAATGGGTAGTTGCAATGGGCTTAGGAATAAGGAAAATGAGAGAGATGAAGAAAGAAAAGGGTTGTTGGTGAATGGAAATGGGAACAACAAAGATG
ACATTTGCTAAGAAAAAAGACACCACACTAGAGAAATGAAATGACACCAATTATGATTTATGACATTGGGATTTTGTTTGGTGGGTAGGATCAATGGGGT
TGGAGGCTTGTGAGTGGTTGCTTTATGATAATTTTGAGGAGGGCTTATCTTATTTTTTTAAAATTTAATTTAATTTGCTTGGGAAACCATTCCAATCCAA
ATCTCATTTTCGATACCAATTGTTTACATATTTTAGCACTTTTCTCTCACCTTCTCTTGTGTTCCTAATGGGCAAGTTTTGTTTTGGGTATTGTGATGAA
AAAGTAGCTTTTGCTGTGTTGTGAGTGACAAGTGCATGGAGATGTGACTGGATGTGAGAATCTGGCATCTGACATTTGATAATATTTTAGATGTAAGGCT
GAGACGTAAGAGACAGAGATTAAACTGAAAGCAGCTTTGCCTGAAAGCTGTTGCACACAATGCAGCACTTGATAATTATTCAAGCCGTGTATAACTCGCG
TCAGAACTCGACCTGTGCTAAACGCCAACAATTTGATTCTCCACAGTCCACACTGCTGACTCATGATCAATTTAGATCGTGACTTTGATCTAAATTGAAG
TTTACCTGTTATTAAGAGTTGGTTCTACTTGTTTGACAAATCGTTTTTCAGTTGAATTGAATGAAGTTTATTCCAAAGTTATTTCTTG

>Glyma.16G157300 | Chr16:31794899..31800043 reverse
TTTTGACAAATACTTAGAATTAGTGAAAGACTGAAACTTTTTTCAAGTATTTGAGCCATACTATACCACACTGTTTATGTTGGTTGCTCTACTTAAGTGC
CCCCCTTTTCCCTCATAGCATTTCTTTCTTGTCACACACCCTTTGCTTCACCCCTTTTTTCAGTTTTTAGAGAGAAGGAGCATAAGGAGGAGAAAAAAAA
GGTTGGTTTGGTTGTTACATACAGCATGCCCTTTTCTCATTCTTGCTTTCATGGGATTAATAGATAGATAGATATGATAATGAAACATTGTAGACCCTAT
TTTTATTTTGTTTTCATCTCCTTTTCTTTCTTCACTCTCCCCTATATGAAGCTACTACTACTATTGCTCAATAGGTTTTGTCAAAAGTAGTCTTTCTTCT
CTTCTAAGTTCTTTCAACACTGTTTGATTCACCACCCTTTTGCAGAACAACAACACATACATATATTGGTCAAGAAGAATGGGAGATAACAAGGACCATG
ATTTCTTTTCCCACAAATTCCCCACAACCTCTCAGGTAAACTCATGTTGCTACTTACTTCTCCATGGAAACACATTTTGTAAGAGAAAAAGAGTAGTTTC
TTTTAGAGATACTCGACCATGATGCTAAAATCAAGTCAAGGCTTTTTAAAAACCGGTTCGCGCGATTTCGTTCGCAACAATAAGGTTTTTCGAGTGTCTG
CTACTGCAAAACAACCTGTGGTGATCACATAGACCTTACCACATTTGTGTGTATGTAATTTCCCACAATATCAAAGAACATGACGAAATTATTGTGACCT
TAACCGTAATTTAGAACCTTGATGCCAGTAAAAAGAAAAAGACTATTGTTTTAAAAATGAGTTTTTTTTTTATCCTTTCTTATTTGTGTTCTAGTCATGT
GATGTTGTGTTGCCAAGTAGGTGATGGAAGAGATGAAGGAGCTGTGGGGCATGGCTCTACCCATCACAGCTATGAACATGTTGGTGTTTGTGAGAGCAGT
GGTTTCTGTTCTCTTTTTGGGTAGGCTTGGAAGCCTAGAGCTAGCAGGTGGTGCACTTTCCATAGGCTTCACCAACATAACAGGGTACTCTGTTCTTGTG
GGTCTTGCAGCAGGCCTAGAACCTGTGTGCAGCCAAGCCTTTGGTAGCAAAAACTGGGACCTTCTCTCTCTCTCTCTCCAACGAATGGTCCTCATCCTCC
TCATGGCAATAGTCCCCATAAGCCTCCTGTGGCTGAACCTCGAAAGGATCATGCTTTTCATGGGCCAAGACAGTGCCATCACAGGAATGGCATCACTCTA
CTGCTTCTACTCTCTCCCAGACCTTTTAACAAACACCTTACTACAACCCTTAAGGGTGTTTTTGAGGTCACAAAAAGTGACCAAACCTATGATGTATTGC
TCCCTTGTGGCTGTTTTGTTCCATGTGCCATTGAACTATCTTTTGGTGGTGGTGATGGGGCTTGGGGTACCAGGGGTGGCCATGGCTTCAGTGATGACTA
ATCTAAACATGGTGGTGCTGATGGCAGGGTATGTGTGCGTGTGCAGGAAGAGGGAGGTGGTGCTGAAGTGGCGGTGTGGTGGTGGGGGAGTGGTGTGCTC
TGGGTTGGGGCAGTTGATGGGGTTTGCTGTGCCTAGTTGCCTTATGATATGTTTGGAGTGGTGGTGGTACGAGATTGTGACTGTGCTGGCTGGGTACTTG
CCACGTCCAACACTGGCTGTGGCTGCCACTGGTATTCTCATTCAGACAACTAGCATGATGTACACTGTCCCAATGGCACTTGCAGGCTGTGTTTCTGCCA
GGGTGAGTCTCTCTCTCTCTCTCTCTCTCTCTCTCCACTATACTATATACTACACTATATTGTATTCAAAATATTCATGGATACTCTCCCTATACCTAGG
GAGAGATATCTAGTCAAACTTTCATTGTTCACACGTATTCATCGATTCAGTTAATGTTTTGATTAAAATTTGCAAATTGAATTTATAAAAACAACTTAAG
TCTTAGTTTTGCAAAACTGAATGGTCGGACAAATTTTTTTTAGTGCATACATACTTTTGGAGGTAATTACATATGTCACCAAAATGAGTTTATCATCGAA
ACTTAGTTTGGAGCTCCCAAGATGGAAAACCAATCACAGCTTCAGAATTATCTCATGATGATTTGATAAGATGTTTCATATTTTTCTTTAAAAATTAGGG
TTAAAAAGTCCATTTAAAATTTGAATTGTTTGATCTTTATTCGACTGTTATAAATACAGTGACTGTGTAAGTGTGTACAAACTTTACAAACTTAACTGCA
TTAGATAGGTCATATAATATCATCAGCAGTAAGCGAGAAAAATGCAATATGTCAATTGAAAATATGAAATATACAAGTGTTCAGTTATCATTGTCCTATT
TAAATAACACAGTGTTCCCAAAGAGGATAGGAAGACTTGTTTGACCATGTTCAGTTCATTTTTTTACCTTTTTTAACCAGTTGGGTTGGTTGAGACAGGG
GAAGATAGGGACTAGAGAGTGGTGTGCAGAGTGCAGGTGGCTTGGCTAGTTTGTTCTTTTCCCACTTTTTGTGTCTGTGATTTGGTGTTAGAACTTGAGA
GCAGGAAGGAACCATTTACTTTTAGCAAGTTGGCTTATGTGGAATGGGGTTTGAAGGCAAAGCAGCAGGGGATAAATTCAAATTTGCAATTTCTTATTTG
GTTTTAAATTTAATGTATCTGGCCTATCATAAATGGTAGTAAATACTAGTACTATGATAGGTTGATCTATTAGCTTACTTTTTTGTGTACTTGCATTATT
TTGCAAAAATGGAAGTAAATACTAGGCATATTTTGATTTTTTTTGGTTTATTATTTATTTATTTATTGGGTTACACTAGAAAAGTGTATATATAACCCAT
GACTGCTTCCAAAATACTTGGCTTGCTCTTATATTCTTTCATAATGTTATTTCTATTTTTTGTTTACCTAAACACTTAAAACATAGTATTGATTGAGCTG
TACACATGTTTCTTTATATAAGATGCTTTACATTTTCTTTGACAAAACATATTTGGAAGGCATTAAATTGTCTTCATCCACATGTGCATTTTAACTTTTG
TGGTGAAAACAATCCTAAACAAACCACCAAGCATAGCTTTAACCTTTGAGGCAGGGACATAAAAGATACAATAAATAATATAAAGTTTGATTTTGTTTTA
TTTTTATTTTTTTCACAATATTATTGATTTGTTAAAGGAGGTGTATAAAAATAGGTTGGAATCATGATGATGGTGTTGATAAATAATGATGATGATGATC
ACGACCAGGAAGATAGTTGTAGTAGTAGGTTGGATCATTTTTTATTTTATTTTTATTTTTTCCACTGTTTCAATGGTTTGTTAGAGATCATGAGAAATTA
CTGGCAACCGTAGGTCTTTTCCCTATCTCTATCTGATCAGACGAGACATTCAGGGTGTGTAAACTGTAAAGAAAAATAGCAGTACATGGACCCAGTTAGG
TCACACTTGCAATGTGACCAGACAAAATTAAAAACTAATTAATTACTTCTAAGTAAGTGCATTTCTGCTAGGCTTGCATACAAGGTCCATCATAGATTTC
CATTTGACCACATAATTTAACCATCAAATCGCCAATGGACTCCACTCTTCTGGTGGGACCCATCAAACTAGTTGGTGTACTTGATAACTTGGTATATTTC
TGTTTGCTGCACCAAAAGTAAACTTGCACATAACATTGGTATGATGTACAATTTGAATTATACCCACGTGAAATTTGCCACGAAGCACTTAGAATAAGGC
TATGCTTGTATATTAGAGTTACAGAATACAATTGTGAATTTGTATAAAATAAAGAAAGAAAAATTTATTGTTTTAAAAGTAAAAAAACTTTTATTTTTTT
AAACTGAAATCCAAGCATACACTAAGGAATTTCACATTTTCAATGCAATTAATGCAGGTAGGGAATGAGCTTGGAGCTGGGAAACCATACAAGGCAAAAC
TAGCAGCAACGGTTGCCTTAGGATGTGCTTTTGTGATAGGCTTCATCAATGTTACATGGACTGTGATATTGGGTCAAAGGTGGGCTGGGCTTTTCACCAA
CGACGAGCCCGTAAAAGCCTTGGTTGCATCAGTCATGCCGATTATGGGCCTGTGTGAGCTTGGGAACTGTCCACAAACCACGGGCTGTGGGATCCTTCGT
GGCACGGCCCGACCTGGTATTGGGGCCCACATAAACCTAGGCTCATTCTACTTTGTGGGCACCCCAGTGGCCGTGGGCCTGGCCTTTTGGTTCAAGGTGG
GGTTCAGTGGGCTTTGGTTTGGGCTTCTGTCGGCCCAGGTGGCATGTGCTGTGTCAATCCTCTATGTTGTGTTGGTGAGGACAGATTGGGAAGCTGAGGC
TTTGAAGGCTGAGAAGCTCACTAGGATTGAAATGGGTAGTTGCAATGGGCTTAGGAATAAGGAAAATGAGAGAGATGAAGAAAGCAAAGGGCTCTTGGTG
AATGGAAATGGGAACAACAAAGATGACACATGCTAAGAAAAAAGATACCACACACTAGAGAAATGAAATGACACCAATTATGACATTGGGATTTTCTTTG
TTGGGTAGGATCAATGGGGTTGGAGGCTTGTGAGTTAACTGCTTTATGATAATTTTGAGGAGGGCTTGTCTTTTTTATTTTTAATTTAATTTTATTTGCT
TGGGGAAACCATTCCAATCCAAATCTCATTTTCTATACCAATTGCTACATATATATATATTTAGCACTTTTGTCTCCCTCTCTCTGGTGTTCCTAATGGG
CAAGTTTTGTTTTGGGTGTTGAGATGAAAAAGTAGCTTTTGCTGTGTTGTGTTGTGAGTGACAAGTGCAAGGAGATGTGGATGGGATGTGAGAATCTGGC
ATCTGACATTTGATAATATTTTAGATGTAAGGCTGAGAGACATAGAGATTAAACTGAAAGCAGCTTTGCCTGAAAGCTGTTGCACATAATGCAGCACTTA
TAATAATAATTATTCAAGCTGTGTATAACTTGCGTCAGAACTTGACCTGTGCTAAACGCCAAAAATTTGATTCTCCACAGTTCACATTGCTGACTCATGA
TCAATTTAGATTGTGACTATCTTCTAAGTTGAAGTTTACTTGTTA

>Glyma.07G246100 | Chr07:42499296..42501951 forward
CACTTTGCTTGCTTCCTCTCCTCGATATCTAACTCCTCTTGGTCCCGCTCACTTTTGTTTTTTGAAGGAGTGTGTTTTTCGGAGGAGAGGAATATATATA
TCAAACACCCCCACCACTATCCCCTTGTGTTCCTCATAATTTTTTCAACACACAAAGCTAGAAACAAGAAAAAAAAAAGAAAAAGGAAAACTAACCAAAA
GGTTTAAGATGCTTGCAGAACAGAAATCCCAAAACACATACCCGACAGTCACCGAGGTAGGAAACTTAATTTACCATTCCATCCTTTCACAACCTTCCTC
TCCTTTTCTATGATTTCCACATACGTACACCTACACTAAATAATATGGTCCACTGAGTTTTAATTAATCTAGGAAAATACTTGTCATCCACTTCCATTCT
CTTCCATCTGAATCCTGTCCTATTTTCTGATAAATCATCATTATCTCAATTAATCTTTACATCATAATAGAATGGATTATTATTTTTTTTTTTAGACCAT
GAACATATAGTTCATTCAAAGTATATACCTAATTGTTTTTCTCACTTTCTTCTTTATTTCTCTTTCTTTCCTGCCACATCACTTGTTACATATATCATAT
TTTCTTTCTTTTAACTTCCCATTTATCTCTATTTTTCCAATCAGTCTATTCCAATGTAGGTAGACGAATATCATTACTCTTCATTTATGACCTTCAACCA
GCCAATGTAAAAGTTAACTTGTTGATATACCAAATATGGATCACTCACGTACGTTTGTATTTTATTTACCATCAATGCAGGTATTGGAGGAAGCAAAGAG
AATGACAGACATAGGGTTCCCAATAGCAGCCATGAGCCTTGTGGGGTACCTCAAAAACATGACCTTAGTTGTGTGCATGGGAAGGTTGGGAAGCCTGGAG
TTAGCTGGAGGTTCTTTGGCCATAGGCTTCACCAACATAACCGGTTATTCTGTCCTCTCAGGTCTAGCCATGGGCATGGAGCCACTCTGCACCCAAGCCT
TCGGTTCTCGAAACTTCTCCTTACTCTCTCTAACTTTGCAGAGAACAATTCTCATGCTACTGCTATTTTCTCTCCCCATTTCTCTTCTTTGGCTCAACCT
CGAATCTCTCATGCTTTGTCTTCGTCAGAATCCAGACATAACCCGAGTAGCAACCCTCTATTGCTGCTTCGCCATTCCAGACCTCATTGCCAATTGCTTC
CTCCACCCTCTTCGTATCTACCTACGCAGCAAAGGCACAACTTGGCCACTGTTGTGGTGCACTTTACTATCCATTCTTCTACACCTTCCAACCCTCACTT
TTCTAACCTTCAAACTTAACCTTGGCGTACCGGGAATTGCTATTTCATCCTTTGTAGCCAATTTTAGCAACCTTTTCTTCCTTCTTTTGTACATGTTCTA
TACGCGTGTCCGCAAGGAGTCCTTACACGTGCCGTTACTAATGCCTCGTCACATGTCACATAATGTAACAACATGTAGCAGCACTAGTACTATTGCCAAG
GAATGGGGTGTGCTAATGAAATTTTCGATACAAAGTTGTCTAGCTGTTTGCTTGGAATGGTGGTGGTACGAGTTGATGACAATTTCAGCAGGTTACCTTC
ATAACCCTCGTGTTTCTCTAGCCACTGCTGGCATAGTGATACAAACCACGTCTCTTATGTACACTTTGCCAACGGCATTAAGTGCATCGGTGTCAACCAG
AGTAGGCAATGAACTTGGAGCGGGGCAAGGTGAAAGGGCAAGATTGTCAACGGTGGTAGCTATAGGATTAGCACTTGTAAGCTCAATTTTTGGTTTGTTG
TGGACCACAATGGGAAGAGAAAGGTGGGGGAGAGTGTTCACTAGTGATAGTGAGGTGTTACAATTGACCATGGCAGTGTTACCTATAATTGGACTGTGTG
AGTTAGCCAATTGTCCACAAACCACAAGCTGTGGAATCCTCAGAGGAAGTGCTAGGCCTGGTGTTGGGGCTGTTATAAACTTTTGCTCATTTTATTTGGT
AGGAGCACCAGTGGCTATTGTTTTGGCCTTTTATTGGAAACTTGGGATGGTGGGTCTTTGTTATGGGTTGCTGGCAGCACAGATCGCATGTGTGGTGTCA
ATTTTTGTTGTGGTGTACAAAACCGATTGGGAAAGAGAGTCATTGAAGGCTACATGCTTAGTGGGAAAGAGTTCATGTGGCACATTTGCATATGAGGACC
AAACTGCCGTCAAATGTGAAGGTGTTGTCTTTCTTCACAACTCCGAAAAGTGAAGAAGATACGCCCCCGATTTACCCTAGTGTTGAAGAAATATACATCA
TAAAGTAGTTATGGCATTACTCAAAGCAAAGTTCAACCTAAAATTATTATGATTCTTTTAGCTAGTATTGGAAAAAAAAATTATTCACACCCCAAATCTT
ACATCCTTCCCCTGTATTTGTGAGTGGATTCTCACGTATAAATACATAAATGTAGGAGGAGGATCTAAGATTTTAGAGAGTGAATTTTTTTTTCATTGTG
ATTATACGTATGCATTTACATAACCAATTGTGATGTTGCATTTTTTTTTCCTTGCGGCCTCAGTGCAAGGTCTAGATTGATGGTGCCATGATATACGTTT
GTGTAATTGTAAAAGTAAGGTTTATCTAATTTGTACTTGATTAATATAACAGATCG

>Glyma.17G027900 | Chr17:2085066..2087848 reverse
TTCCTCTCCTCGATTTCTAACTCCTCCAGGTCCCGCTCACTTGTGTTTTTTGAAGGAGTGTGTTTTTCGGAGGAGAGGAATATATAAAACACCCCCACCA
CTATCGATCCCCTTGTGTTCCTCGTAATTTTTTCAATTCTTTCTCCAAACACAAACAGCTAGAAACAAGAAGAAAAAAAAACAAAAAAGAAGGAATACTA
GCCAAAAGGTCTAAGATGCTTGCAGAACAGAAATTCCAAAAGACATACCCGACAGTCACCGAGGTAAGAAATTTAATTTACCATTTCATCCTTTCACAAC
CTTCTTCTCCGTTTCTATGATTTCCACACACACATACACGTCCACTAAATAATGTGGTGCACTGAGTTTTATTTAGGAAAATACTTATCATCCACTTCCA
TTCTCTTCCATGTCCTATTTTCTGAGTGAGAAATTACCATTATCTCAATTAATCTTTACATCATAATGGAATGGAATATTATTAAAAATAAATTGTAGAT
TAAAAAACTTAAATAATGTCATATGCATGGGACCATGAAGACATGGACATGGTGATCAAGAAGTATATATCTAATTATTTCTGTCATTTTCTTCTTATTT
TTCTTTCTTTTCTATCACATTACATATTACATGGCGGATAATAAAAAAAACCATTAATATTTTATATTACATCTATCCCTTTTTGGATAGACAAACATCA
TTACTCTTCATTTATGACCTTCAATCAGCCAATGTAAAACCATGATATGATGTACCAAAGAACAATAATATACAAACATTCACTTATTTTAAAAATTTCA
TAAAAATCTCTCATTTTTTTATCTTTGTTTTCCTATTACATCGTAAATTTTATAATACTTATATTATTATTGTTGTGTGTCTCTAGGTATTTTATAGCAT
AATGGGTGTTGATGAAAGATTTTTCTATACCAAATTAATATGGATCACTGACGTCTGTATTTTATTTACCATGAATGCAGGTATTGGAAGAAGTAAGGAG
AATGACAGACATAGGGTTCCCAATAGCAGCCATGAGTCTTGTGGGTTACCTCAAAAACATGACCTTAGTTGTGTGCATGGGAAGATTGGGAAGCCTGGAA
TTAGCCGGAGGTTCTTTGGCCATAGGCTTGACCAACATAACCGGTTACTCTGTCCTCTCAGGTCTAGCCATGGGCATGGAACCCCTCTGCACCCAAGCCT
TCGGTTCTCGAAACTTGTCCTTACTCTCTCTAACTTTGCAGAGAACAATTCTCATGTTACTGCTATTTTCTCTCCCCATTTCTCTTCTTTGGCTCAACCT
CGAATCTCTCATGCTATGCCTTCGCCAGAATCCAGACATAACCCGAGTAGCAACCCTCTATTGCCGCTTCGCAATTCCAGACCTCATTGCCAATAGCTTC
CTCCACCCTCTTCGAATCTTCCTACGTAGTAAAGGCACAACTTGGCCACTGTTGTGGTGCACTTTACTATCCATTCTTCTACACCTTCCAACCCTCACTT
TTCTAACCTTCAAACTTAACCTTGGCGTGCCGGGAATCGCTATCTCATCCTTTGTTGCCAATTTTAGCAACCTTTTCTTCCTTCTTTTGTACATGTTCTA
TACACGTGTCCCCAAGGAGTCCTTACACGTGTCTTTACTAATGTCACATAATAATTTAATAATAACATGTAGCAGCAGTACTAGTACTATTGCCAAGGAA
TGGGGTATGCTAATGAAATTTTCGATACAAAGTTGTCTAGCTGTTTGCTTGGAATGGTGGTGGTACGAGTTGATGACAATTTCAGCAGGTTACCTTGATA
ACCCTCGTGTTGCTCTAGCCACTGCTGGCATAGTGATACAAACCACGTCTCTTATGTACACTTTGCCAACGGCACTAAGTGCATCAGTGTCAACCAGAGT
AGGAAATGAACTTGGAGCGGGGCAGGGTGAAAGGGCAAATTTGTCAACGGTGGTGGCAATAGGATTAGCACTTGTAAGCTCAATATTTGGTTTGTTGTGG
ACCACAATGGGAAGAGAAAGGTGGGGGAGAGTGTTCACTAGTGATAGCGAGGTGTTACAATTGACCGTGGCAGTGTTACCTATAATTGGACTGTGTGAGT
TAGCCAATTGTCCGCAAACCACAAGCTGTGGAATCCTTAGAGGAAGTGCTAGGCCTGGTATTGGGGCTGTTATTAACTTTTGCTCATTTTACTTGGTAGG
AGCACCAGTGGCAATTGTTTTAGCCTTCTATTGGAAACTCGGGATGGTTGGTCTGTGTTATGGGTTGCTGGCAGCACAAATCGCATGTGTGGTGTCAATT
TTTGGTGTGGTATATAAGACTGATTGGGAAAGAGAGTCATTGAAGGCTAGATGCTTGGTGGGAAAGGCTTCATGTGGCACATTTGCATATGATGAGGACC
AAACTGATGCACTCAAATGTGAAGAAGGTGTTGTCTTTCTCAACTCCCAAAAGTGAACAAGATACGTCCCCGATTCACACTAGTGTTGAAGAAATATAGA
CCATAAAGCAAGCGTATGGCATTACTCAAAGCAAACTTCAACCTAAAATTATTATGATTCTTTTAGCTAGTATTGGGAAAATTTATTTTTATTCACACTC
CAAATCTTACATCCTTCCCCTGTATTTGTGTGTGATTATACGTGTGCATTTACATAACCAATTGTGATCATATATTTTTTTTCCTTGCAGCCTTGCAAGG
TCTAGGAAGATGGTGTCATGATATATAATTGATATGTAATTGTAATAGTAAGGTTTATCTAATTTGTGCTTGATTAGTATAAC

>Glyma.09G042700 | Chr09:3653337..3655456 forward
TTTTCTTGTTCCCTCCCTTCTCTTCCTGGTCCCCTCTCACTTTGTACTTGAGGAGTGTTTTGGGGAGAAAAAAAGACACCCCACTATTTCATTAATTTTT
GTTTTTTTATCATATTTTTTTAGAATATTTAAAATGTGAGTTTAAGTTTAATTCAATTTCAAAAGTTAAGTGTGATTTGAATTTGTTTCAATAATATTAT
CTCACACCACACGGAAACAAGAAAGGGAAAAATAAAACGTTTAAGATGGTTGCTGAGGGGAAATCCCAAAAGCCATACCCAACAGCTGCTGAGGTACGAG
CAAACGAGACCTCACACATCCTTTCAGAACCTTCCATGTTTCTCACTCTTAATTTCACTTCTTCATCTCACTTAGACTGTTGTCTATTTTTTTTATTAGC
AACATATACTACTTCCGGTCTTATTTATAAAAAATAAGTTTTAATGTAGTTTATAAATATATTATAACTTATTTCTTATAAATAGGATCCTAGATAATAT
TATAGTCTATAGTATAATATAAGCTCATTTATTCATACAATAACATTAACTAGTTTCATTGTGTTTAATTTGTTGCTATAGGTAGTGGAAGAGCTAAAGA
GAATGGGGGACATAGGAGTTCCAATAGCAGCCATGAGCCTTGCGGGATACATCAAAAACATGGTCTTAGTTGTGTGCATGGGAAGGTTGGGAAGCCTTGA
GCTAGCAGGTGGTGCTTTGGCCATAGGCTTCACCAACATAACAGGTTTCTCAGTTCTCTCAGGTTTAGCCATGGGAATGGAACCTCTTTGCACACAAGCC
TTCGGTTCAAGAAACTTCTCTTTGGTCTCTCTCACTTTACAAAGGACAATTCTCATGTTATTGGCAGCTTCACTACCCATTTCTCTCTTGTGGCTAAAGC
TCGAGCCTCTCATGTTATGGCTTCACCAAAACCCTGAAATAACCAAAGTGGCAAGTGTCTATTGCTTCTTCTCAATCCCTGACCTAATTGCAAATAGCTT
CCTTCACCCAATTCGAATCTATTTACGTAGCAAAGGGACAACTTGGCCTCTATTGTGGTGCACTTTGCTTTCCATTCTCATACACATCCCCATTGTGGCT
TTTTTTACCTTCAAACTTCATCTTGGTGTGCCCGGGATTGCTATGTCAGCTTTTGTTGCCAACTTCAACACCCTTTTTTTCCTCCTATCATACATGCTCT
ACATGCGTGTCTCAAAGGGATCACTTTCCATGCCTTTACTATTATCGTCAACCTGCCAACTGCCATCACCATCACCACAACAACAACAACAACAACATCA
TCATCAAGACCAAACCTCATTAAAAACAACAACGCTAGGAAAAGAGTGGGGCATGCTAATTAGGTTTTCCATTCAGAGTTGCTTAGGAGTGTGCTTGGAG
TGGTGGTGGTACGAGTTCATGACGATTCTCGCAGGCTACCTGTATAACCCACGTGTTGCTTTAGCAACTGCTGGCATAGTGATACAAACAACATCTCTCA
TGTACACTTTACCAACGGCACTAAGTGCTTCAGTGTCAACTAGGGTTGGGAATGAGCTTGGGGCAGGTCAACCCGAGAGGGCAAAATTGTCAACAATTGT
GGCAATAGGACTGTCACTTGCAAGTTCAATATTAGGGTTGTTGTGGACCACAATAGGGAGGGAGAGATGGGGGAGGGTGTTCACAAGTGATAGTGAGGTG
TTGGAACTAACAATGAGTGTGTTGCCCATAATTGGGGTTTGTGAGTTAGCAAATTGCCCTCAAACCACAAGCTGTGGAATCCTTAGAGGGAGTGCTAGGC
CTGGTGTTGGGGCAGGGATAAATTTTTACTCATTTTACCTTGTGGGGGCACCAGTGGCAATAGTGATAGCATTTGTTTGGAAACTAGGGTTGGTGGGGCT
TTGCTATGGCTTGTTGGCAGCACAGATAGCATGTGTGGTCTCTATTCTAGTTGTGGTATACAACACAGATTGGGAAAGGGAGTCTATGAAGGCAAAAAGC
TTGGTGGGAATATTTAAGAGTTCATGTGATCATGATCATCATTATGGAGACCAAACAGTGAAATGTGAAGAAGGTCTCTTGTCTTTCTCAATGAGAAAGA
ATAGCGACCAAAAAAAGTGA

>Glyma.15G150300 | Chr15:12416065..12418420 reverse
TGTTCCCTCCCTTCTCTTCCTCTGGTCCCCTCTCACTTTGTACTTGGAGGAGTGTTTTGGGGAGAAAAAAAAAAAAAGACACCCACTATTTCAATAATTT
TTGTTTCTCTTTATCACATACCTCAAACCACACGCAAACAAGAAAGAGGAAAAAAAAAAACGTTTAAGATGGTTGCGGAGGAGAAATCCCAAAAGACATA
CCCGCCAACTGCTGAGGTACGAACAAACGAGACCTTACATACATCCTTTCAGAACCTTCCACCCTCGTCCATGATTTTCACTCTTAATCTCACTTGTTTA
TCACTTATACTATAGTTCATATTATTTTATATCAACATATTATAGTTGCGACCATATCAATATTAATGCACCAGATTCTATCAAATATATTATCGTCTAT
ATTGTAATATAAGCACATTCATTTATACAATAACATTAACTATTTTCATTGTGTTTAATTTGTTTGCTATAGGTAGTGGATGAGCTTAAGAGAATGGGGG
ACATAGGAGTTCCAATAGCAGCCATGAGCCTTGTGGGATACATAAAAAACATGGTCTTAGTTGTGTGCATGGGAAGGTTGGGAAGCCTTGAGCTAGCAGG
TGGTGCTTTGGCCATAGGCTTCACCAACATAACCGGTTTCTCAGTCCTCTCAGGTTTAGCCATGGGAATGGAACCTCTTTGCACTCAAGCCTTCGGTTCA
AGAAACTTCTCTTTGGTCTCTCTCACTCTACAAAGGACAATTATCATGTTATTGGTAGCTTCACTACCCATTTCTCTCTTGTGGCTCAAGCTCGAGCCAC
TCATGTTATGGCTTCACCAAAACCCTGAGATAACCAAAGTGGCAAGTGTTTATTGCTTCTTCTCAATCCCTGACCTAATTGCTAACAGCCTCCTTCACCC
GATTCGAATCTATTTACGTAGCAAAGGGACAACTTGGCCTCTATTGTGGTGCACTTTGCTTTCCATTCTCATACACATCCCCATCGTGGCTTTTTTGACC
TTCAAACTCCACCTTGGTGTGCCCGGGATTGCTATGTCAGCTTTTGTTGCCAACTTCAACACCCTTTTCTTCCTCCTATCATACATGCTCTACATGCGCG
TCTCTAAGGGGTCACTTTCCATGCCTTTACTAATATCATCTCGTCCCTTGTCGTCGTCACCACGACAACATCATCACCAAGACCAAACCTCATTAAAAAC
AACAACAACACTAGGAAAAGAGTGGGGCATGCTAATTAGGTTTTCCATTCAGAGTTGCTTAGGGGTGTGCTTGGAGTGGTGGTGGTACGAGTTCATGACG
ATTCTCGCGGGCTACCTGCATAACCCGCGAGTTGCTTTAGCCACTGCTGGCATAGTGATACAAACAACATCTCTCATGTACACTTTACCAACGGCACTGA
GTGCTTCAGTGTCAACTAGGGTTGGGAACGAGCTTGGGGCAGGTCAACCTGAGAGGGCAAGGTTGTCAACAATTGTGGCAATAGGAATGTCACTTGCAAG
TTCAATATTAGGGTTGTTGTGGACCACAATAGGGAGAAATAGATGGGGGAGGGTGTTCACAAGTGATAGTGAGGTGTTGGAACTAACCATGAGTGTGTTG
CCCATAATTGGGGTTTGTGAGTTAGCAAATTGCCCTCAAACCACAAGCTGTGGAATCCTTAGAGGGAGTGCTAGGCCTGGTGTTGGGGCAGGGATAAATT
TCTACTCATTTTACCTTGTGGGGGCACCAGTGGCCATAGTGATGGCATTTGTTTGGAAACTAGGGTTGGTGGGGCTTTGCTATGGCTTATTGGCAGCACA
GATAGCATGCGCGGTCTCTATTCTAGTTGTGGTATACAACACAGATTGGGAAAGGGAGTCTTTGAAGGCAAAAAGCTTGGTGGGAATTTATAAGAGTTCA
TGTGATGATCAACATCATGGAGACCAAACAGTCAAATGTGAAGAAGGTGTTGTCTTTCTCAATGAGAAAGAATAGCGACCCAAAAAAAGTGATGCCTACA
[truncated: 353,267 more chars]
