# Supplementary material for: Genome-wide analysis of MATE transporters and expression patterns of a subgroup of MATE genes in response to aluminum toxicity in soybean
Source: BMC Genomics. 2016 Mar 11;17:223. doi: 10.1186/s12864-016-2559-8 (PMC4788864; doi:10.1186/s12864-016-2559-8)
Supplement: Additional file 8: — The 1500 bp upstream sequences of the eight soybean C4-3 MATE genes. (DOC 43 kb) [file 12864_2016_2559_MOESM8_ESM.doc]

**Additional file 8. The 1500 bp upstream sequences of the eight soybean C4-3 *MATE* genes.**

>GmMATE13

TAAAATTGGTGAATATTTTACACGAAATTCAAAAATATAATCTACTTATTTTTGTAGCTA

TTTATTTAAATTAATTATTCATATTTTCTTATAAGTATAAATAATTAATCAATTAATATT

TCAAAGTTTAATTTAATTTAAACTATATATATATATTAAATAAAATAGATGATAGTTATT

TTTACCTTCATCCAAATTATATATAAAATGATTATATATATATATATATATATATATATA

TATATATATATATTAAACTATATATATATATATATATATATATATATATTCTTATCCTTG

AAAATGATTTTAAGTAACTAAAAGTAGGAAAAATTATTTTAAAAAATAAAGTATTTAGTA

TTCCTTTATATATCTTAAATCATATATATTTTATTTTTTCATAAAATTTTTCCAATTCAA

GCTGTTTAAAATTTACAATTTAGAGATAATGTTAACAAATTAAACCCATAAATAAGACAA

AAGCGTAACAAATCAATAATATATTATGTATACTATTTTTCTTTTATCCTTCATGGATCA

CATGCCTTTATTAATTTCCATTATTAAATTTAGAAATAATAATTAGTATTTGAGTAATTT

CTTATTTTATCATTGAAAAACATTTGAAATAGTCTTTTGGTCCTTAAAATATCAATGACT

TCGATTTCATATTCAAATGTAATATCTAATTACCAATAAAAAATATAACTTGTATATTTT

TAAAAAATATTTATTATGAATTTTAATTATAATATAGTTTACAATTTTTTTTACAAAATA

TAGTTTACAAATTTAAAAATAATTTTTTACTATGAATTATGATTATAATTATATACAAAT

AAATATTTATATTGTACAATGCGCTTAAATACTAGTTATAGTATAAAGTTGAATAAGATA

ATATTCATTAAACCTTTTAAGATCTGGGTTTTGCACAACTGAGGGAGGTGAACCTATATG

GTGTCGAAAGGATAGAACCTGTTGTTCAAGATATACTTTTAATTATAATATAGTTTACAA

ATTTAAAAATAATTTTTTACTATGAATTATGATTATAATTAGATACAAATAAATATTTAT

ATTATAATATAAAGTTGAATAAAATAGTATTCATTAAACCTTGTATAATCTGGGTATTGC

ACAACTGAGGGAGGTGAACCTATATGGTGTCGAAAGGACATAACCTGTTTGTTCATTATT

TTAAGTTGATTAATTTCCGTTTTTCATAGATTGCATTGTAGGATATAATATAATATGATA

TGATTTTCCCCTTTGCTTCAAGGATGTTGCTTCATTCATTAATTGGGTTCTCTATAAGAA

AAAGTATGGAGTCATGAAAGTCTTTTTTATTTCTTTTAGTTGTGATGTATTCACATGTTT

ATCTTTCGTGCCAAAGTGGTGGTGATTGTTGGTGATGGTTGATCAAATACTGAATATTGA

AAAGAAAAGTGGTGGACTTGTGTAAGCAAATTCCATTGCGAGAAGTAGCTGTTCTCAGGA

>GmMATE47

TAAAAATAATTTTGTAATAAAATTTAATGTAAAATTCCCCATCCATAGCAAGAATTACTT

CTTTCGTTTCCAATCTAACATAAGTTTGGTTCAACAATCAAATATGATTCTTTTTTTATG

ATAAAATAATGTTAATTAGAAGAAGACTTCGAACTTGAGTTTTAACAACGTAAGAACCAT

AATGCGTATCCACTATGCAAATACACATTAGTTATGCAGCAAGATTCCTACGGAGGCTTT

TTTTGTCTATATTTCTATTTTCTTCAACTTGTTTAGTTCTCTTTTTTAAATAATAAAAAA

AAAACTGAGAGAATCCTGCAATTCGTTAAGCATTTGTTTACGTCACTCGATGCTTCACCT

TTCGTGTCCATTCTTGACGTGAACAAAAAATTAAATAAATCATTACTTTATCATCAAGAT

CAGGTTCATGACTCAAGTTAATTATGTGGTCATCAATTCTCGTACCATCAATATTTTAAG

CTTTGATCACTGATATTATGCTCTCTATAATCCGGTTTATGACACCCTTTATCTATATCA

GGGAGATGGTACAGTTTCTTTTTCTTTCTTCTTTTTTTTTTTCTTGAATATATGTAACTT

CCATGTTTGTTAACATTTTCAGTGATCAACTCTCTTCACAAATATCTGAACTCTATTAAT

TTAATCAATATTTGGGATTACGCAGTAAATTAATTCATTGGTAGAGTAGAGTTGCAAATT

TAGCGCATATAATCTTGCTTGTTTAGCAAAAAAAATATTGTATAAAGAGGGACATAATAA

GCTAAGTGTAAAAATTAAAATAGAAAGCATTTATCTTTCCTTGATTTGCATGAGGTCCAT

ACTCTATAGAGTAGTACCCCCCAGCAATGTAAGTTCTCTATTCTAAAGATCCTTTTATAG

TTTGCATGCTGGATTTCAAGAATCATGATATTTCAAGAATCATGATATAAAATAAAATAA

AAACAATACACATTCTTGTTTAGTATAAAGTTCAATTGATATAACTTTTCTGTTATGTAG

CTATCGGGGGTTTAATTAACTAGGGACGGTTGTTGCACTATCATTTGTCACATTCTTCCA

AGTTCTGCCTACGCATCTTTTAACCTTTTTGCATCTGATGGTTAATTAAGTTAAAATTAG

GCAGGGTTACTTTATGAGAAAATGACGTTAAAACACATCTGAGGAAGGCTCAAATTAGAA

TCGTGACGAAACCCATAATTATGCCACTATTGTCCATTACTTTTTCAACACTACATGATT

TCTGCAACACTTTAATTAACATTTGGCTTTTGCATATCTACAATCACTATAAATAGGTTT

CACTGATCTTATAGTGATCTTTCAATGCTAGAAAAGTAGCACATTATTATATCCGCATGG

TGATCAATATCAAACAAGCTTATTTTTTTATTTTGATGGTACAAAACATTGAATGTTGTT

ATCAAACTCAAAAATTGGTACTCTTCATGATTGAGTTGTCATTTTACTATTTAGGATATC

>GmMATE58

ACATATCACTATCAATTGAACGCAATATGCATGCCTAACGTGGTCCACAGTCACAAATTT

TAAACATGAAGAGGAGTTACAATTTGCATTTTAAACCCTGTGAAGAAGAAGACAATCACT

TGGAGGATCTACGGCACATTTGTGGCTATACAACCGATTGCATAGTTATAATTTTCTTCT

TAAGCGGTAATTAACAATCTCTTCATTAAAACTTTTTATTCTTTTAGTTTGTACACATAT

GTTATGAATGTGATGAAATTCAACAAACTCCCTTTAGCAATTATGCTCATGGATAATACA

TCACTTTTATTAATAGTAATCCCAAATTGGCAAACGAGATAGTTAGATTAAAGGAGTGCA

TGAGATGTTTTCTATCAAATGAGGGATTCAAGTTATATCTATTCAAGATATGCTCTGCCT

GTATCGATTTAATTTTAATTCAACTAGATATAAACCTATGTATTATGTAGGTATCTTAAT

TTTTTTAATGAATTACATTAATAACAATAAAATACATTAAAAAAATAAAAGCTGCATGGA

AATAATTATCTTTTTACTTAGAACATAAAAAAATTGGTTTAATGGTATTTAGAAAAATAA

TATATGAAATTTGTGTGATAACCTTATTGCAGTAAAAAAAATGGTTGATTATACTTGCGT

TCAATACTTCTAAATCTTTTTTTTTCTTTTTTTTCCACTATATCTATATTTATTATCACC

AATGTTTAAGTATCCTAACTATTAATAATTTGTATACATCAAATAAAGTAAAGAAAAGAT

TGTGACTTCTAGTCCTCATATATGTTGGTCACTCAAGAGCTATGAAAGAGATAAATCAAA

GAGAATGATTTATATGAAGACAGCTCATGAAAGAAAACTGGTAAATGAGATTAAAGTATA

AACTAACTTGACAACTAAGATATCAATTTCAAAGCTTGCGCTTTTATAAATTATATATTA

GATATTTCATCTCCTGGTTTTTATACAAATTTCATGTTGCTATTTAGTTTTGATGTTCAA

CATACTCAATTGCAGCTGAAGATCGATCAACAATGCCACACTCATATACTTATCCAATGT

CAAGAAGTCTGGGTTTAGAAGGAAACAATTGCTTTATTTGAAGATAAATTAATCCTTCAC

TGCAAACATAGGTAAGTAAAAGCTTTATTTTTTTATAACATTGCACCACACTATCTTAAT

ATATATGATGTAATAACACATAATATATTGTTAGTTATTGTGTCATATATGTTTTAAATA

TTTAGTTGCTAGTTTTAAACACATTTATATTTCCATTCTCCTAATCTCATGTATTCACAT

GTTTATCTTTCGTGTCAAAGTGGGGATGATTGTAGATCAAATTCTTTCATGATGTTCTCT

TTGACTGTTTCTAGTTTTCATTGGTGTTGGTTGATCAACATTCAACTACTAAACATTGGG

AAGAAAGGTGGTGAACTTTTGTAAGCAAATTTCGTTGAGAGGAACTAGTCGTTCTCAGGA

>GmMATE74

AAAAATAAAAAATTATTAATTTAATTAATTTTAAAACTATTGAGTTTTATAAATCAATTG

ACGTGAAATTATACTAAATTAATAGGTGGTCTAATGTTTGAGTAATAATTATGTATTTTT

GTTAAATATTCGAAATCACAGTTTTATTATTTATAATAATCCTATTGAAACAAAATTTCC

TCCTATAAATGATACCTATTGTTTATGCAAAAAATATTAAATTACTCTGCATGTCCGTGA

ATTATTTTTCTAATTACATACTTGAACTAGAAATTGTTTTTAATTAGGTCCCAATTGTGA

TCTAATTAAAAAATAAATACAAATTCAAAAACCTAATTAAAAAATAATTCAATAACCTAT

TTAAAAATGACAAATAATTCATAAACCTTGTTGAAAATTGTAAAGTCTTTGCAGTGCATT

ATTTTCACCTCTACATGTCAACACTCGCCTCTGTAGACTCGATATTCTTAACTAGGTGAG

ACAGTGACTTGCATAGACTTAAATAATTTTTAATTAGGTCTTTTTAACTATTTGTTATTT

TTAAATAGCACTTTAAACTATTTTTTTAATTAGATCCTAAATTTATATTTATTTTTTTAA

TTAGGTTACAATAAGATCTAATTATAAAACAAGTACAAATACAAAAGTCCAATTAAAATA

AAAACTTATAAACCTAATAATAAACTGTGAAATAGTTCAAGGATTTGCAGAATAATTTGA

CCTGAAACAAAATTATGGGTCTTACTACGCGCACTATGTAAATTGTTGGTACACCCAACA

TAATTTTCACAATTCCCATTTTGTCCTTCCGTAGAAGGGATATGGATTGTGAAGGGATAT

GGATTGTCTAATCTGTAAGTGGTTGAAACAATTTTTTTTTTCAAAAATATATTTTACGAA

TTAATTTAAAATTAATAGTTCAAGTAGAAATTGAATATGTGATTTTTGTATTATTAACAT

GACACTCTAACCAACTGAATTAATAGTTAAATTATATTATAAAATAATTAATGTCATTAT

ATATAATATTAAAATTTTTAACATATATTTAATGCACATGTAAATTTAAATAATTAATTT

TGTAATAATTAATTTTGATCTAATATGTATGTAAATGCAATATTGCTTAGTTATTTAGCT

CTTCAATATCTTCAATGACTCCTATAAAAACCATCACTAGCTTCTCTAGTGATCTTTCAA

CGCCAAAAAGGTACCACATTACTATATTCTTACCTTTTATTATTTCTAGTTGCCAACCTT

GATTGCATGCGGCGTGACCATCATATATATATATATATATATACATTCCTTCTTAGTTTT

GATTAATGTGAACATTATATAAACATTAATATTGGACGATCTCAAACTCAAATTATGATT

AACTTGGCAGGTACATAGTTGAGGATTGACCTTAATTATCGTGAACGATCTTAATTTCTT

CTTTTGATCAATTGTTTCAAGCACTTCAGTGAAGATCTCTTGTGTGAGATTCACAATCC

>GmMATE75

ATTATGTATGTGACTAGTAATTGACTTACTTGATTGAAATCATTGCATGTTAGGATTAGA

AACAAAACCCTATATTAAGGCCAAGACCAGATCCTTATTTCCAAAATCATGTTCAAATAC

GCGGTTTCTAAAATATTAAAAAAAAACTTGTTGAAATCATATGTACATATACAATTTCAA

CTTTTAATAATAAAAAAATTAAAAAATTGAAATCATATGTGCATATACGATTTCATTGTA

ACAATTTTTTGAAAAATAAAATCGTACTTCGAAGCACGATTTTTTCAAGGTGAAATCGTA

TTTCAAAGTATGATTTATTCATTTTCGTAATTTTTAAAAATATACCCATTATGATAATTT

TTAAAAAAAATTACCCCTTTATGGTGCAAGAGCCCTCGTTTCTTGTTGCCCCGTTGGTGT

ATATATATATATATATATATATATATATATATATATATATATATATATATATATATATAT

ATATATATATATATATATATATGCACGCTAACTAGATTATATGATTTTCTTAAAAATAAA

AAATAAAAATAAGTGTATGATATAATGGTTGTATATCAACTAAAAGAGAGACGATATCAT

AATTTAGAGGGGGAGGGGGGAAATGCCCCTGTATCATATAGGTATGGCTTGGTTGTGTAG

AATATATGGAATGAAAAAAAAAATTAACAAAAACATTTGAATTAAAATAGTGTAAAAAAG

ATGTTTGATCTATATTAAATAGTAAAAAAAATTTGTCCAACCCTTTCTCATCAATTTCGT

CTCAAGCAAACCAAGGGATATATAACGTGATCCCGTCCCTATTTGTAGTTTGTACTATAT

GCTCGATCGGCTCTTACAATTCTGTATATTTCAGTTCTAACTGGGGAGCCATTTGCATAT

TTCTCGATGCATGCATATATAAAATAATAATATACATTTTCGTTTGTTTCTACTTGATAG

TTGTTACGAAGTTTCTATTATGTTGCTATCAAGGATATATAGGGACCTAACTGAGGACGG

TGCTTACTGTAGTGAGGATCTGTCACGATCTTCATTCAACTAACCCATCTTTGAATATTT

AGATAGCATGCCATGGATCCTTCTCTAGCCTACATATTTTTTTATAGCAATTTAATGTCA

TCAAGTGTTCTTAATTAGTATACCAATTAAAAATAAAAAATAGAAAACTTTTATTAAAAA

AATTATCAATACACCTAGCTGAAATTTCCGTAGCATCTCCAAATGTACTGATTCCATAAT

TAGTGTGTGATCACTGATTATTAGCAAGAAATTATTTTAAGTTATAAGGGTTTCTTTATA

CTTAGAAGATGAAATCTATAGTTTAACTGAGGAAGGTTCCAACTATAAAACAAAACAAAG

TAATGATGACTGTAACGTTTACCAGGCTTATTTAGTTGGCTCTTCGATATCCCCAATGAT

TCCTATAAAAACCCATCACTTCTCTAGTGATCTTTCAAAGCCAAAAAAGGTACCACATT

>GmMATE79

TCAAATGAAAGTGAAAACATTTCTATTTAAATTAGATTCTAAATTCAATGCATTTATGTA

AATATATATATATAAAAAAATGTAAGGTGTTTGTATCATTGAAGCTTCATAAATCATAAT

AATGCAGGTTATGACCTAGGCCGGGAGGTTCCAAAATTCTCATTCTCACCTATCATATTC

ATATCAGCAAGTGACGTAACGAATTAGTCTAACTTTACATGGAAAAGCATTTTTAATTTT

TTCCTTTCTTCTTTTTTTTGGTACAGATTTTTCCTTTCTTTTATGAAAAGTGTCAACACA

ATCATTTTAACATGTTTTCTCTCATTAATTAAAATTTATTGAAATTAAAATGTATAAAAT

TATAAAAGAAGTTATAAAATAAGTTTGTAAAAATTATAATTTTTAATAAATTTTTACTAA

TAGAAAACGTGTGTTTAAAATAGTTGTAATGCTTTTCTAACATTTCTCTTCTTTTATCAT

GTTGGATTGGAGGACATTCCAAAGCAGAATGTGGCAGACACGTGGCAATCGTTGGTAAGC

GTGCCACATGTTTTAACGTGGCAGACACTGAGAGACTTTTGACGTGTATGGACCGCTTCT

ATGGATTATAGTACTGCCACATGTTTTAACAATTCATGGTACACAAGAGCTACTAAGATC

AATGAAGCTCCTTCTACTTCTTTTAAGTGTCCCAAAGAAGCTGAAAGTTGAACCTGGCTA

GCTGGGCCCGCATGATGTCTGTGTTCAACTTTTTTTCCACCACCATATATTATTGCTTGT

TACCTCAAACTTCTCTTGTCCTGTTCTCAGTGTTCCATTATCATTCTTTCAGTCGCATGC

ATATTTTATGTGATATATATGGACAATTGAACATGCTATGGTTGTTGTTTGTTCTCCCCC

AAGTTAAAGGGTGTTTTTATTTCAAAGCTAGTATTAAAAGCCTAAGTGCGGAGTTTCAAA

CCTTGAATTTCAGGTGAATCACATTTTTGGGTAGCAGGGAAATCTGAGTTGCAGTTTATT

TGTCTTACTAGTGATTGGTTGGTTTGGCTTTCTTTCAGAGCCCAAATGTCCAAGTTTGAA

TGTTACTAACTACTATTAAGCAACCGGATTTGGAAAAACATCTCCTGTTTTTTTAAGGGG

AACTCACTTTACAGTTTACAGTGATTCTCATGTGGGTAAACCCTTTTTGTGTGTTTTAAT

TACGTACTTTTTTTCATTCTTTATTATTATAGTCAGTGGTTTTGCTCATACATAGTGAGA

AGGACTATTCTATGCCAAGAGGGGAAGAATATAAAAGGGCATCTCCTTTACCAACTTTTT

CATCACTGTGGAGTCTATCCCTATTCCATGATGATAATGGTATTCCATTATGATTTGACT

TGATTGGTTGGTTGGTCTGTATTTAACCACATAAATAACGAGATATTATGCAGTCTCAAT

ACATTGGAGCCGAAATTATGATGTCTTTAAGTGTTAATGCTCAAGTTAAAAGTTTAATTC

> GmMATE84

GATTGCATTAAATCCATAAAGCCTTTTTTGTCGGTCTTTTGATAATATTATGACTTTAGT

AGAAACGTAACTAGTCCTACTACGTCCTCGTGTCATTAAATCAACATTCTATTAAATTAA

ATAAAAAAATAGAAAACTGTTCAGATATACTTTTTGACATTTTTATTTTAAATATAAATA

TTAAAAAATAATCTGTATTAATTAGAATTCATGTAAAATTTCATTCAATAATATAAACGT

CAAATAATAATATATATCCGTATGATACAAGGTACTGTTTTTTCAATGCATTTATTATTA

GACTATTTTTATTATTATTTTACTTAATAAAAAATAGAAGTCATAAACTATATATATATA

TATATATATATATATATATATATATATATATATATATATATATATATATATATATATATT

GTACAGTGCTTGTATCATTGATGCTACATAAATCATAATAATGCAGGTTATGGCCTAGGC

CTCATTCTGATCTATCATATTCATATCAGAAACTAGCAAGTGACGTAAAGAATTAGTCGA

ACTTTACATGGAAAAGCATTTATATGAGTTTTACTTTATTGTGTGAAGAGTTTTAACACA

TGATCTTAAACAGGTTTTCTCTTATTAAATAATAAAATTGAAATCTATAGAGTTATAAAA

GAAATTTATTAAATAATGAAAATTATATTTTTTTTTTGTAATTTTTAACAAACTTTTACC

AATAAAAAAGTGTGTTTAAAATAATGTTTTTCTAATTATTATGTTGGACTTGGAGGACAT

TCCACAGCGGAATGTGACAGACACGTGGCAATCGTTGGTAAGCATGCAGTGCCACTTCGA

GACTTTTGACGTCTATGGATTATAGTACTGCCACATGTTTTTGGCAATTTATGGTACACA

AGAGCTCCTTCTACTTCTTACCATATTATTGCTTCCTACCTCAACTCAAACGTCTCTTGT

TCTGTTCTCAGAGTTCCATTATCATGCTATGGTTGTTTTTCGCCCCCAAGTTAAGGGTGT

TTTATTTCAAAGCTAGTATTAAAGCCTGACTGTGGAGTTTCAAAGCCTGAATTGCAGTTT

ATTTGTCTTACTTGTGATGGGTTGGTTTGACTCTCTTTCAAATCCCAAATGTCCAAGTTT

GAATTAAGGGGAACTCACTTTACAGTGATTTCTCATGGGGGTAAACCCTCTTTGTGTGTG

TTAATCAAGTACTTTTTTTCATTCTTTATTATTATAGTTAGTGGTTTTGCTCATACATAG

TGAGAAGGACTATTCTATGCAAGAGGGGAAGAATATTAAAGGGCATCTCCTTTACCAACT

TTTTCATCACTGTGGAGTCTATCCCTATTCCATGATGTTATTCCATTATGATTTGACTTG

ATTGGTTGGTCTGTGTTTAACCACATAAATACCGAGATAGTATTATGCAGTCTCTCTACT

TTGGGGCCGAAATTATTATGATGTCTTTAACTGTTAATGCTCAAGTTAAAAGTTTAACGC

> GmMATE87

GATTCCTATGGAAGCGTTTCTGTCTTATTTCTACTTGCTTTAACTTTAATTAGTTCTTTCTTGTTTTGAATTTCTTTTAAGAATAAAACTGACATGTTCA

GCGTGGAATTGCACACCCGATCTCAGCAAATCCTGCAATTCGTTAAGCATTTGTTTATGTTACTCGATGCTTCACTTTCTGATGCCCATTCTACATGTGA

ACAAAAAATTAAATAAATCATTACTTTATCTTCAAATTCAGGTTCATGACTCATGTTAATTATGTGGTCGTTTCTATTCCATATGAAACGTTGATCACTG

ATATTATGCAATAAATTTCCTTATGACATGCTTTATCAGGGAGATGGTTGGCATTTTTTCTTGAATACATGTAACTTCCCTATGCTGTTTAACATTTTTT

CAGTTCTCAATGTTCCGCACAAATATTTGAACTCTATTAATTTAATCAATATATGGGATTACCTAAGTCAAGTAGGTAAATTAACTCATTGGTAGAGCTG

CAAATTTAGCTACATATAATCTTGCTTGTTTGGCAAGTTATTGTAAAAATTATTGCATTTAACTCATTGCATATATGCTAAGTCTTCTAAAAATTAAAAT

ACATAAATGTTCCGCACAAATATTTGAACTCTATTAATTTAATCAATATATGGGATTACCTAAGCCAAGTAGGTAAATTAACTCATTGGTAGAGCTGCAA

ATTTAGCTACATATAATCTTGCTTGTTTGGCAAGTTATTGTAAAAATTATTGCATTTAACTCATTGCATATATGCTAAGTCTTCTAAAAATTAAAATACA

TAGCATTTAGCTTTCCTTGATTTGCATGAGGTCCATATTCTAAAGAACCTTTTACAGTTTGCATGGTGGATTTCAAAAATCATGATACAAAATAATAAAA

AAAAACAATACACATTCTTTTTTAGTATAAAGTTCAATTAATATAACTTTTCTTTTATGTAGCTATCAGGGGTTTAATTAACTGGGGACGGTTGTTGCAC

TATCATTTGTCACATTCTTCCAAGTTCTGCTTACGCATCTTTTAACATTTTTTGCATCTGATGGTTAATTAGTTAAAATTAGGCATGCTTACTTTATGAG

AAAATGACGTTAAAACACATCTGAGGAAGGCTCAGACTATAATCGTGACGAAACCCATAATTATATGTTACTACTGTCTGTTACTTTTTCAACCCTCCAT

GATTTCTGCAACACTTTAATTAACATTTGGCTTTTGAATATCAGCAATGATTATAAATAGTTCACTGGTCTTATAGTTGATCTCTCAATGCTAGAAAAGT

AGCACATTATATCTCTGCATGGTGATCAATATAAAACAAGTTTATTTTTTTATTTTGATGGTACAAAACATTGAATGTTGTTGTCAAACTCAAAAATTGG

CAGATTGTTGAGATTTGGCTAACTCATCCCGAAGGGAAATACGTTCCTTTTCGCACTATCCTCTTCTTGATTGAGTTGTTATTTTACTATTTAGGATATC
