# Supplementary material for: Genome-wide analysis of MATE transporters and expression patterns of a subgroup of MATE genes in response to aluminum toxicity in soybean
Source: BMC Genomics. 2016 Mar 11;17:223. doi: 10.1186/s12864-016-2559-8 (PMC4788864; doi:10.1186/s12864-016-2559-8)
Supplement: Additional file 9: Table S7. — Details of cis-acting elements in the 1500 bp upstream of the eight soybean C4-3 MATE genes. (DOC 33 kb) [file 12864_2016_2559_MOESM9_ESM.doc]

**Table S7. Details of *cis*-acting elements in the 1500 bp upstream of the eight soybean C4-3 *MATE* genes.**

| Cis element | Sequence | Function |
| --- | --- | --- |
| ABRE | ACGTGGC | cis-acting element involved in abscisic acid-responsiveness |
| ARE | TGGTTT | cis-acting regulatory element essential for anaerobic induction |
| CGTCA-motif | CGTCA | methyl jasmonate (MeJA)-responsiveness |
| HSE | AAAAAATTTC | heat stress-responsiveness |
| LTR | CCGAAA | cis-acting element involved in low-temperature responsiveness |
| MBS | CAACTG | MYB binding site involved in drought-inducibility |
| TCA-element | CCATCTTTTT | cis-acting element involved in salicylic acid responsiveness, stress response |
| TC-rich repeats | ATTCTCTAAC | cis-acting element involved in defenses and stress-responsiveness |
| TGACG-motif | TGACG | involved in transcriptional activation of several genes by auxin and/or salicylic acid |
| WUN-motif | AAATTTCCT | wound-responsive element |
| W1-box | TTGACC | fungal elicitor responsive element, enhancing gene expression in seeds |
