# Supplementary figures and images for: Genome-wide analysis of MATE transporters and expression patterns of a subgroup of MATE genes in response to aluminum toxicity in soybean
Source: BMC Genomics. 2016 Mar 11;17:223. doi: 10.1186/s12864-016-2559-8 (PMC4788864; doi:10.1186/s12864-016-2559-8)

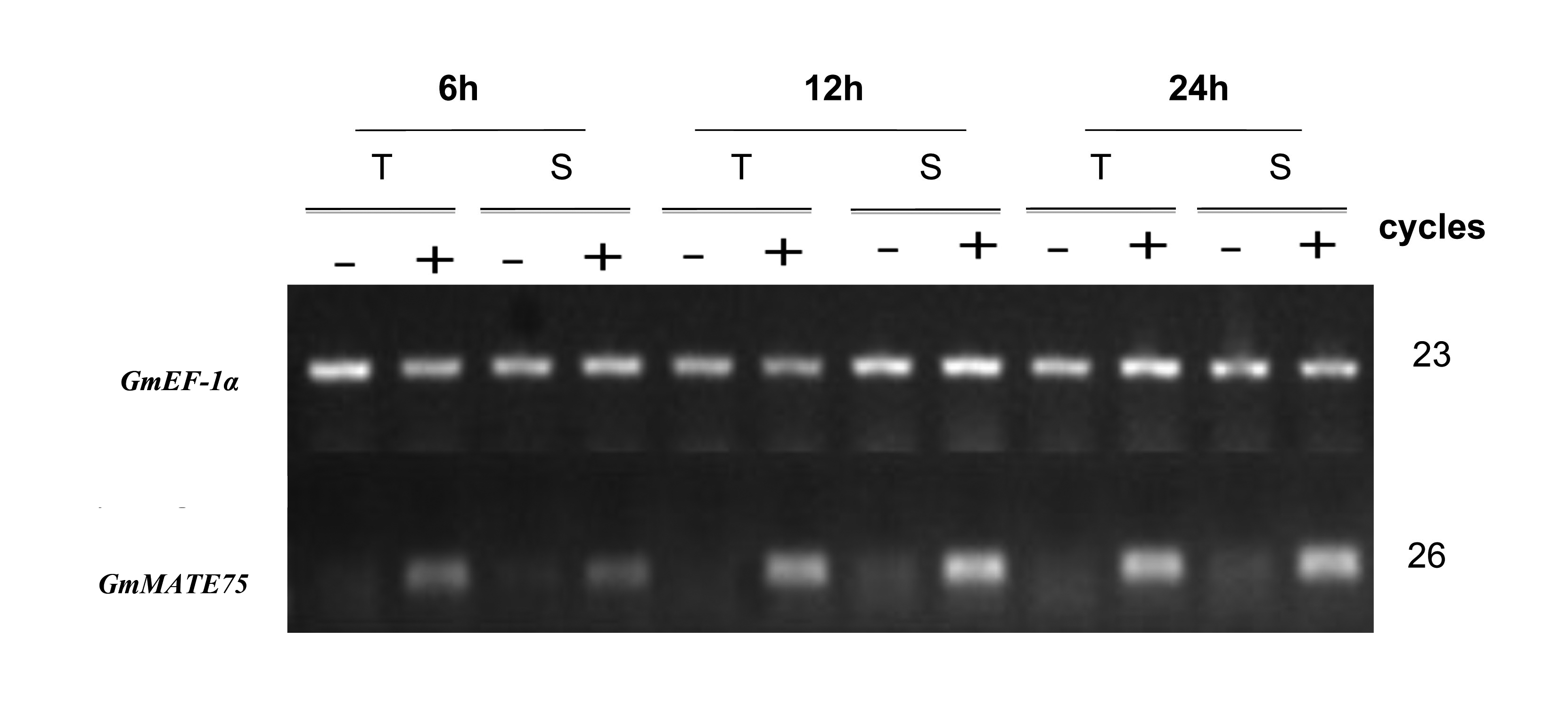

Supplement: Additional file 10: Figure S1. — Semi-quantitative RT-PCR of the candidate MATE gene for Al tolerance in soybean. The semi-quantitative RT-PCR was performed using the RNA from soybean root tips (0–2 mm). - represents control plants (0 μM AlCl3) while + represents plants treated with 25 μM AlCl3. GmEF-1α was used as the internal control. T: aluminum-tolerant cultivar, KF; S: aluminum-sensitive cultivar, GF. The number of PCR cycles is shown on the right. (PNG 569 kb) [file 12864_2016_2559_MOESM10_ESM.png]
