# Supplementary material for: Genome-wide analysis of MATE transporters and expression patterns of a subgroup of MATE genes in response to aluminum toxicity in soybean
Source: BMC Genomics. 2016 Mar 11;17:223. doi: 10.1186/s12864-016-2559-8 (PMC4788864; doi:10.1186/s12864-016-2559-8)

**Figure S2. The amplicon specificities of 15 pairs of primers for qRT-PCR in this study.**

A.GmMATE13


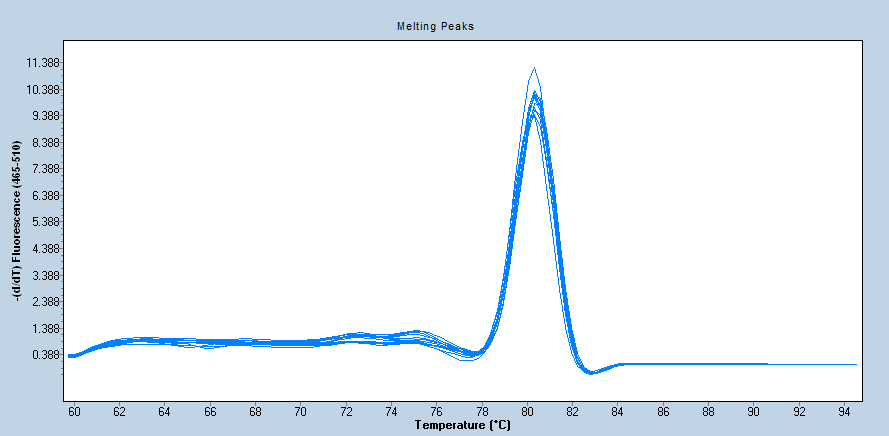


B.GmMATE36


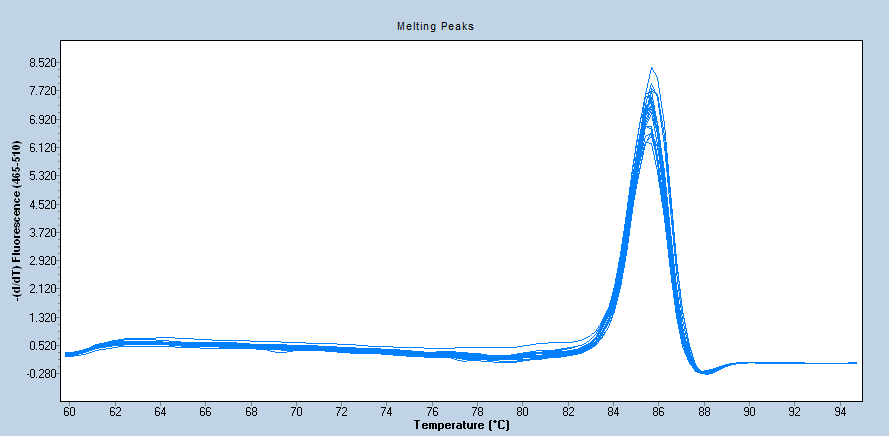


C.GmMATE47


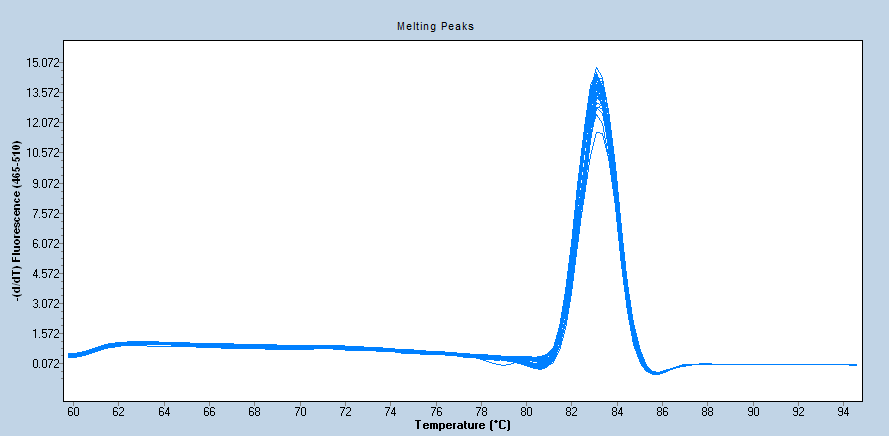


D. GmMATE49


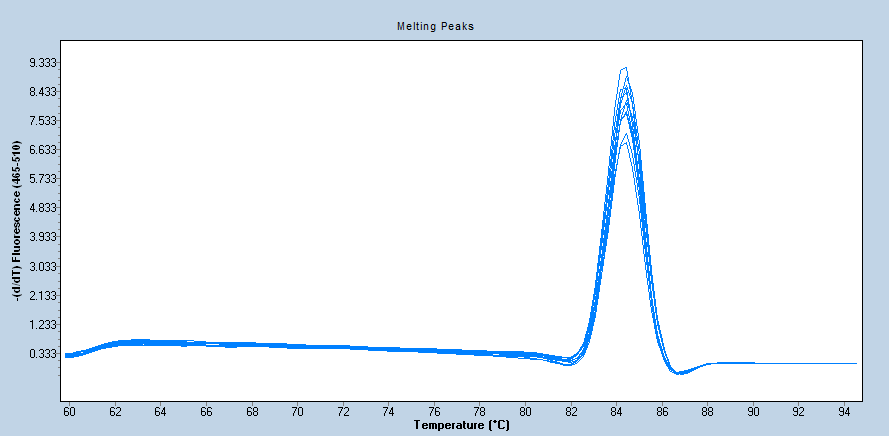


E.GmMATE58


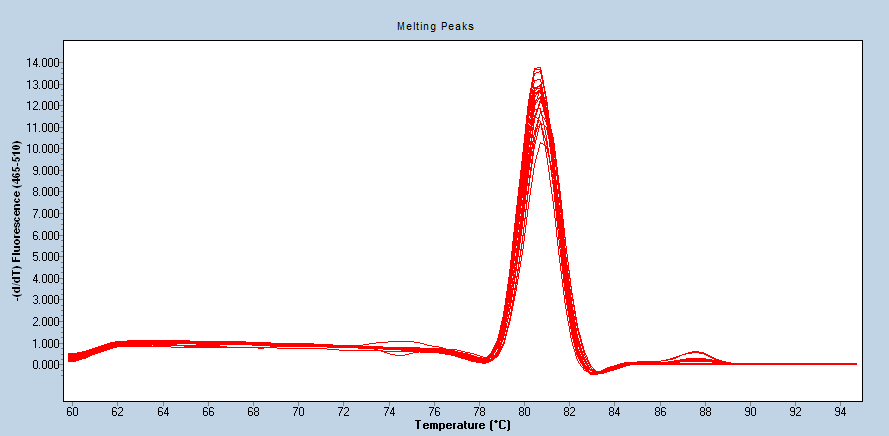


F.GmMATE74


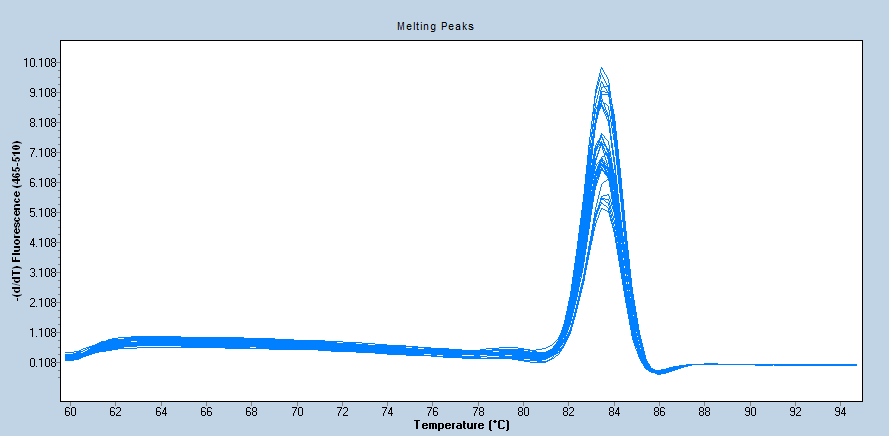


G. GmMATE75


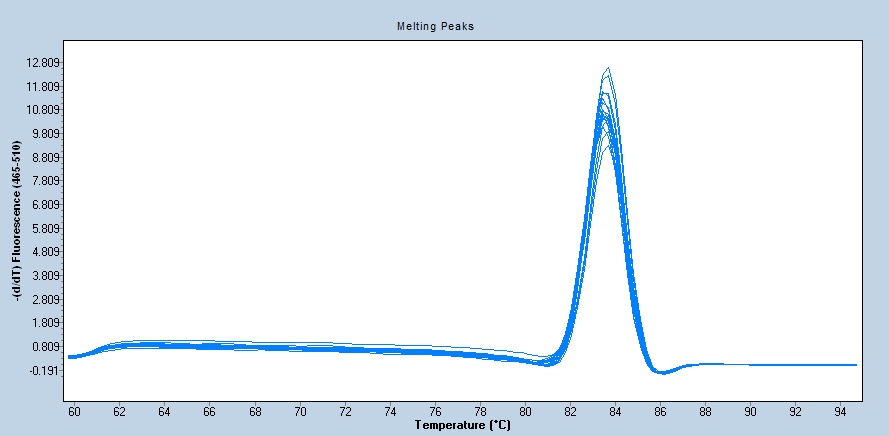


H.GmMATE79


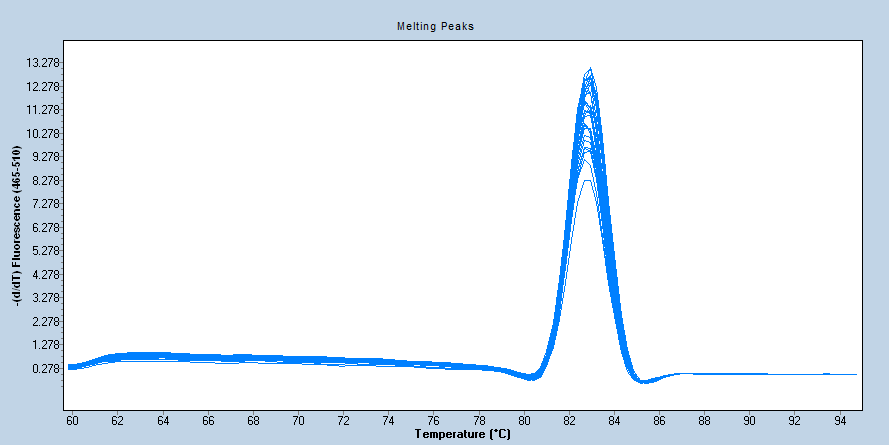


I.GmMATE84


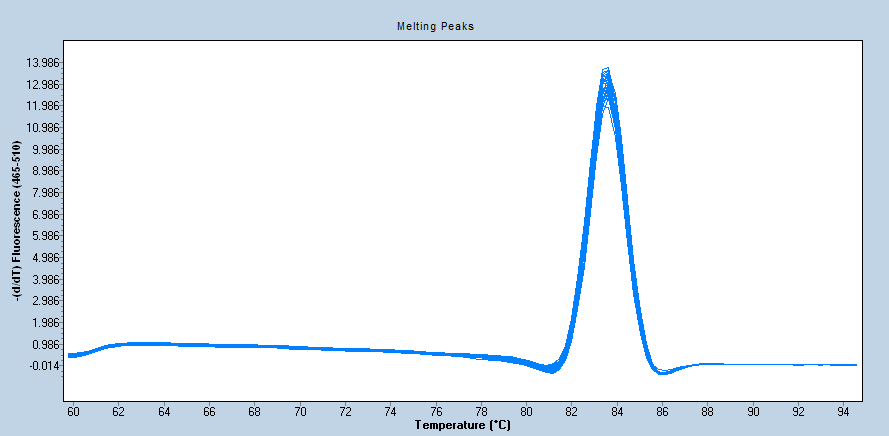


J. GmMATE86


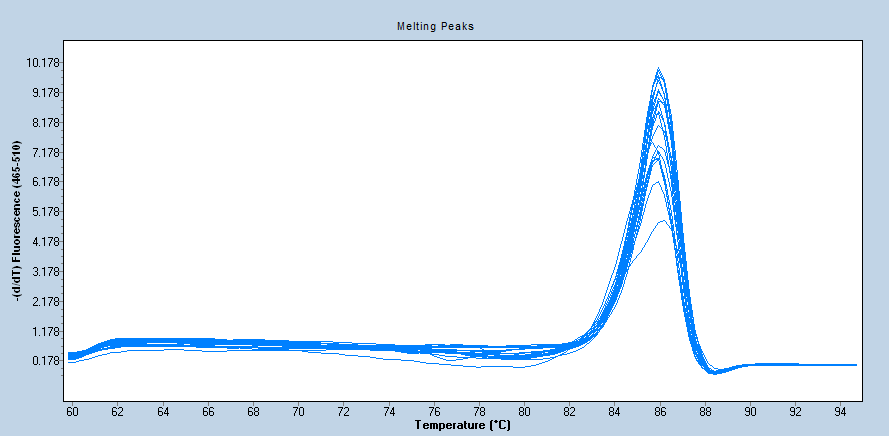


L.GmMATE87


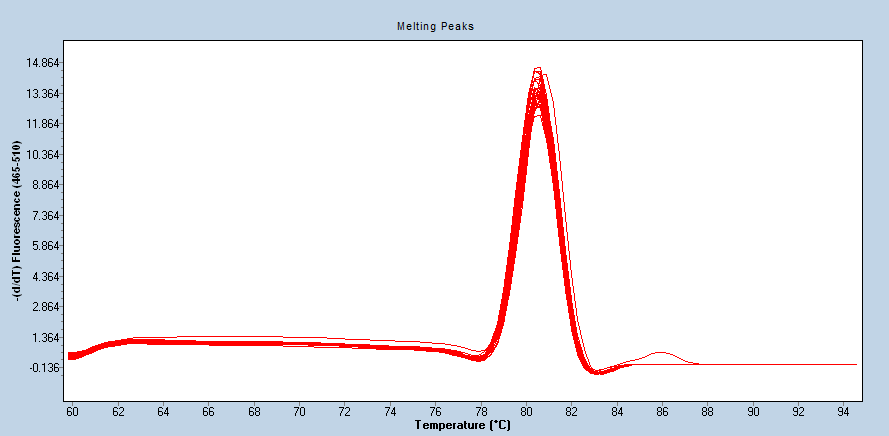


M. GmMATE91


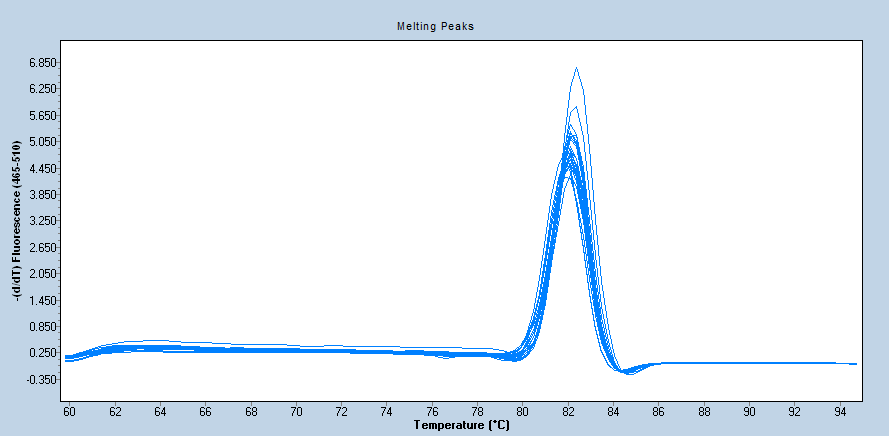


N. GmMATE93


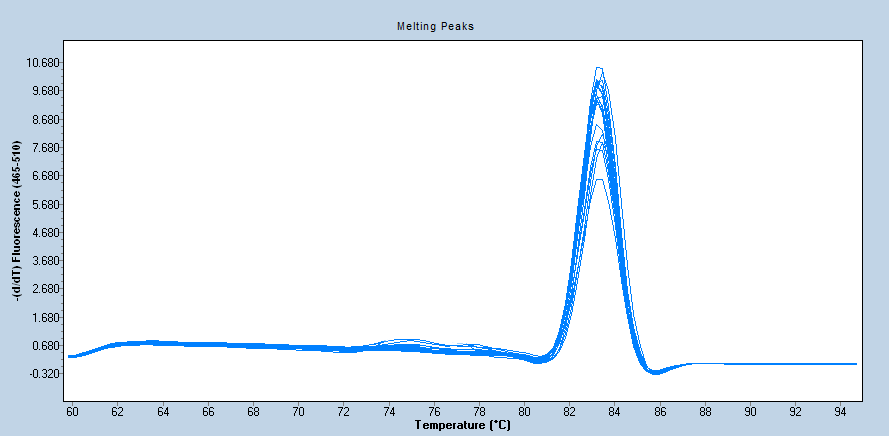


O. GmMATE117


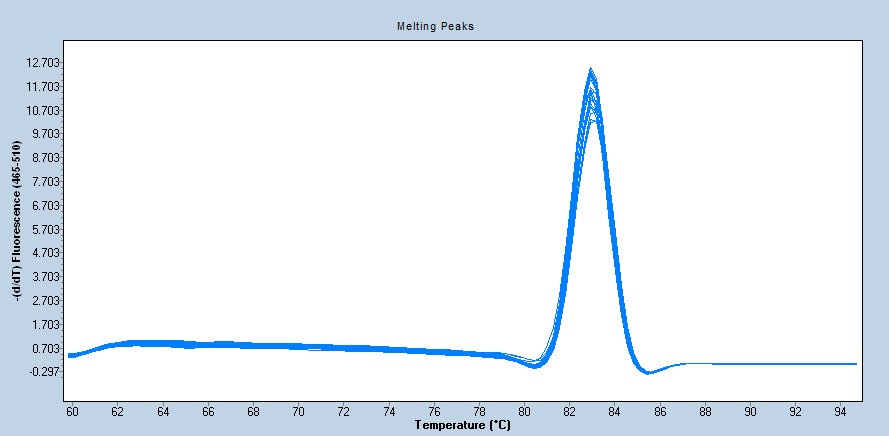


P.GmEF-1ɑ


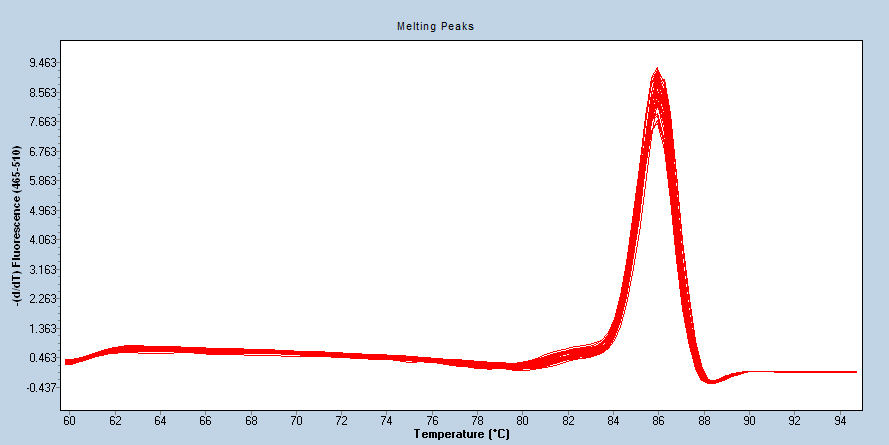

Supplement: Additional file 12: Figure S2. — The amplicon specificities of 15 pairs of primers for qRT-PCR in this study. (DOC 220 kb) [file 12864_2016_2559_MOESM12_ESM.doc]
